# Supplementary material for: Model for predicting drug resistance based on the clinical profile of tuberculosis patients using machine learning techniques
Source: PeerJ Comput Sci. 2024 Oct 14;10:e2246. doi: 10.7717/peerj-cs.2246 (PMC11623081; doi:10.7717/peerj-cs.2246)
Supplement: Supplemental Information 2 [file peerj-cs-10-2246-s002.zip › code/EDA/chi2/Tuberculosis_chi2_and_plot_1.pdf]

Resultados

Tabelas de Contingência

Tabelas de Contingência

		Status_Resistencia		
testesensibilidade		0	1	Total
N	Observado	1467	686	2153
	% em linha	68.1 %	31.9 %	100.0 %
S	Observado	54	1847	1901
	% em linha	2.8 %	97.2 %	100.0 %
Total	Observado	1521	2533	4054
	% em linha	37.5 %	62.5 %	100.0 %

Testes χ^2

	Valor	gl	p
χ^2	1836	1	< .001
N	4054		

Survey Plots

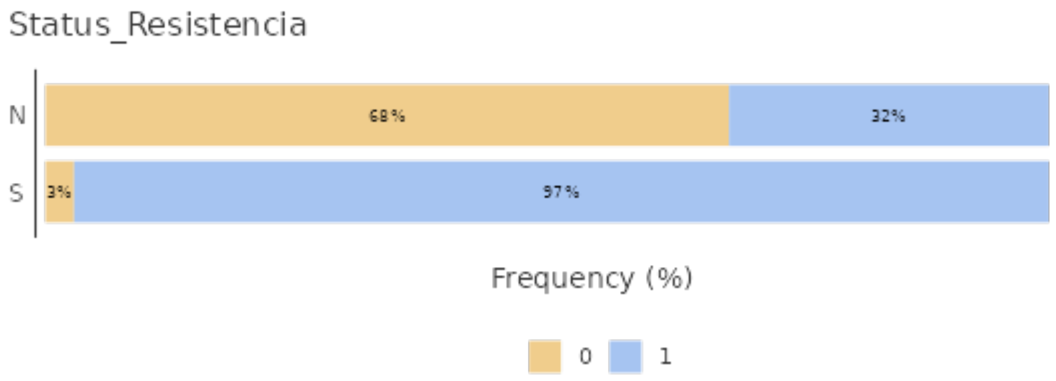

Tabelas de Contingência

Tabelas de Contingência

cultEsc		Status_Resistencia 0	Status_Resistencia 1	Total
And	Observado	127	54	181
	% em linha	70.2 %	29.8 %	100.0 %
N/realiz	Observado	7773	2095	9868
	% em linha	78.8 %	21.2 %	100.0 %
Neg	Observado	2041	896	2937
	% em linha	69.5 %	30.5 %	100.0 %
Pos	Observado	2251	9930	12181
	% em linha	18.5 %	81.5 %	100.0 %
Total	Observado	12192	12975	25167
	% em linha	48.4 %	51.6 %	100.0 %

Testes χ^2

	Valor	gl	p
χ^2	8568	3	< .001
N	25167		

Survey Plots

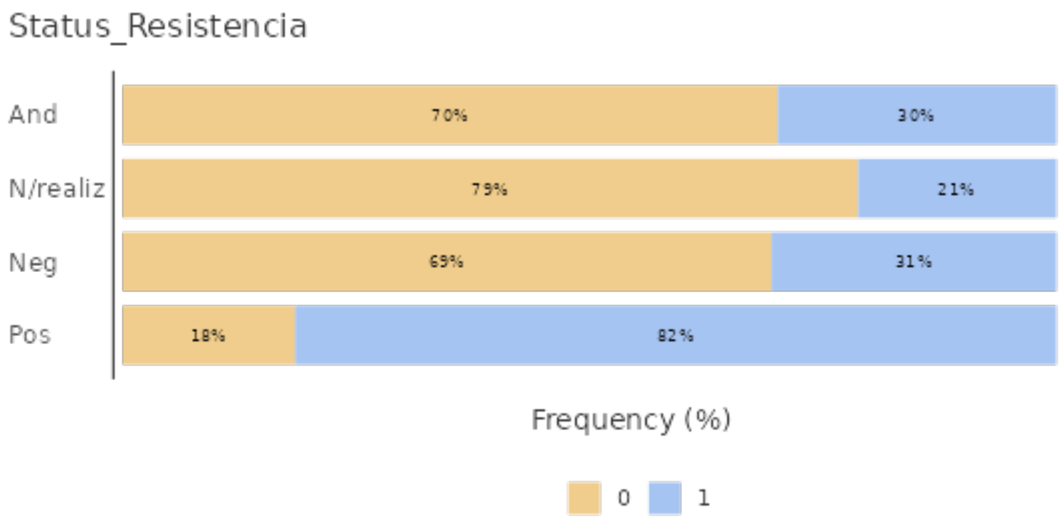

Tabelas de Contingência

Tabelas de Contingência

CULTOUTRO		Status_Resistencia 0	1	Total
And	Observado	36	17	53
	% em linha	67.9 %	32.1 %	100.0 %
N/realiz	Observado	9788	9547	19335
	% em linha	50.6 %	49.4 %	100.0 %
Neg	Observado	476	292	768
	% em linha	62.0 %	38.0 %	100.0 %
Pos	Observado	298	1068	1366
	% em linha	21.8 %	78.2 %	100.0 %
Total	Observado	10598	10924	21522
	% em linha	49.2 %	50.8 %	100.0 %

Testes χ^2

	Valor	gl	p
χ^2	483	3	< .001
N	21522		

Survey Plots

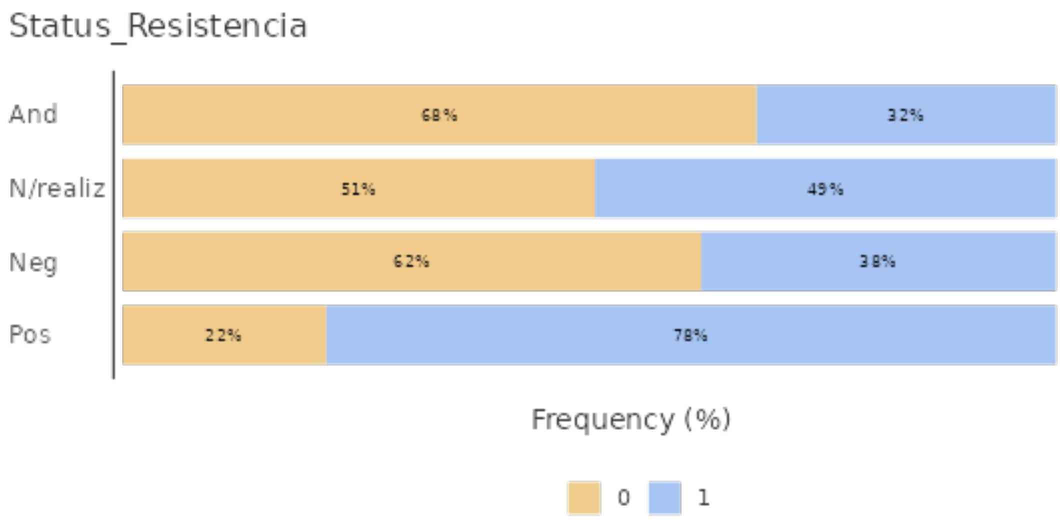

Tabelas de Contingência

Tabelas de Contingência

mtvInter1		Status_Resistencia 0	Status_Resistencia 1	Total
Abscesso	Observado	12	22	34
	% em linha	35.3 %	64.7 %	100.0 %
Aids	Observado	78	257	335
	% em linha	23.3 %	76.7 %	100.0 %
Caquexia	Observado	80	147	227
	% em linha	35.2 %	64.8 %	100.0 %
Causas Sociais	Observado	149	522	671
	% em linha	22.2 %	77.8 %	100.0 %
Diabetes	Observado	10	17	27
	% em linha	37.0 %	63.0 %	100.0 %
Elucidacao Diagnostica	Observado	1252	1747	2999
	% em linha	41.7 %	58.3 %	100.0 %
Hemoptise	Observado	132	146	278
	% em linha	47.5 %	52.5 %	100.0 %
Insuficiencia Respiratoria Aguda	Observado	641	817	1458
	% em linha	44.0 %	56.0 %	100.0 %
Intolerancia Medicamentosa	Observado	18	36	54
	% em linha	33.3 %	66.7 %	100.0 %
Meningite	Observado	24	22	46
	% em linha	52.2 %	47.8 %	100.0 %
Nao Adesao ao Tratamento	Observado	32	75	107
	% em linha	29.9 %	70.1 %	100.0 %
Outros	Observado	442	528	970
	% em linha	45.6 %	54.4 %	100.0 %
TB Miliar	Observado	14	20	34
	% em linha	41.2 %	58.8 %	100.0 %
Total	Observado	2884	4356	7240
	% em linha	39.8 %	60.2 %	100.0 %

Testes χ^2

	Valor	gl	p
χ^2	171	12	< .001
N	7240		

Survey Plots

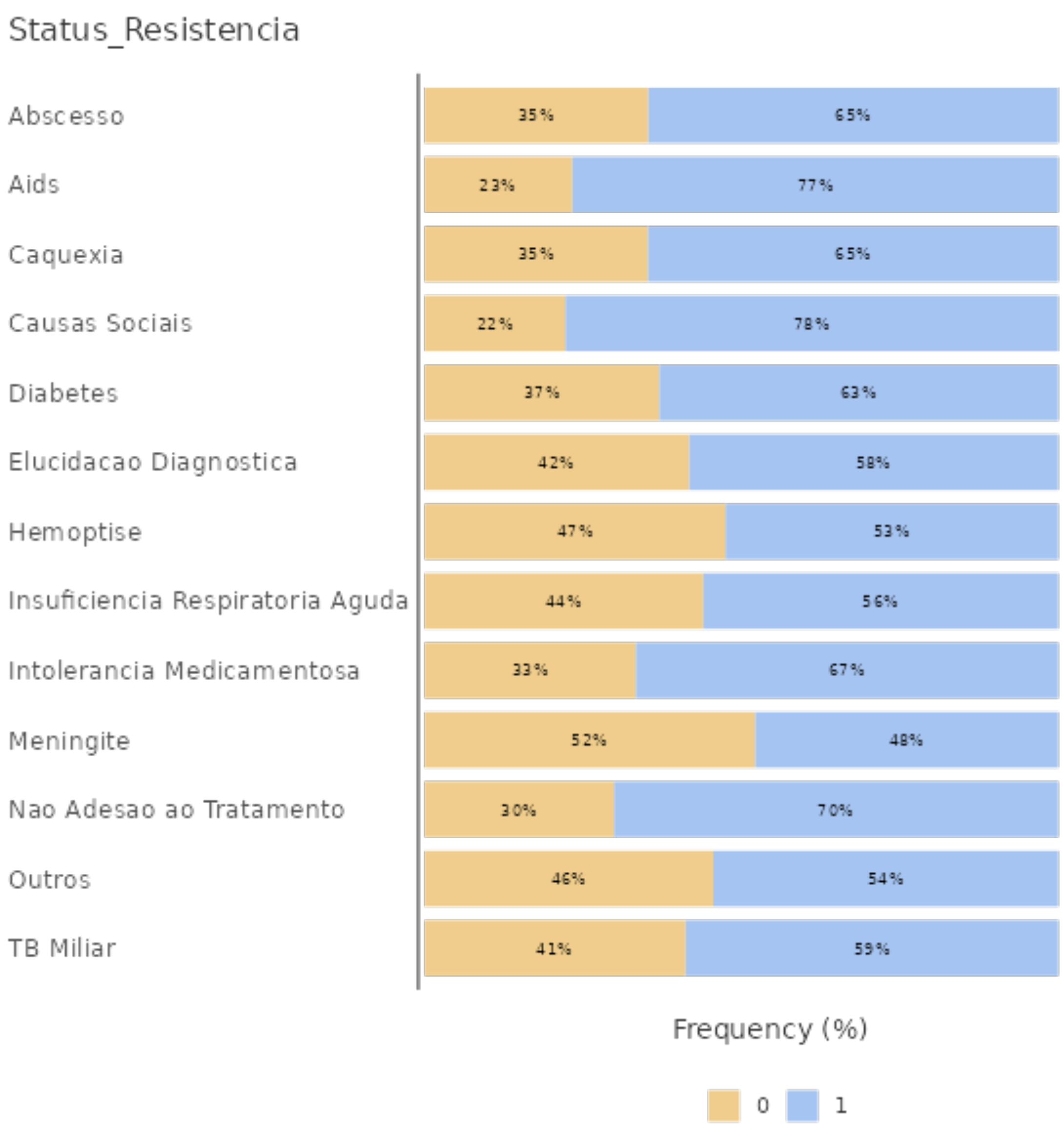

Tabelas de Contingência

Tabelas de Contingência

aids		Status_Resistencia 0	1	Total
N	Observado	12394	11279	23673
	% em linha	52.4 %	47.6 %	100.0 %
S	Observado	913	2028	2941
	% em linha	31.0 %	69.0 %	100.0 %
Total	Observado	13307	13307	26614
	% em linha	50.0 %	50.0 %	100.0 %

Testes χ²

	Valor	gl	p
χ^2	475	1	< .001
N	26614		

Survey Plots

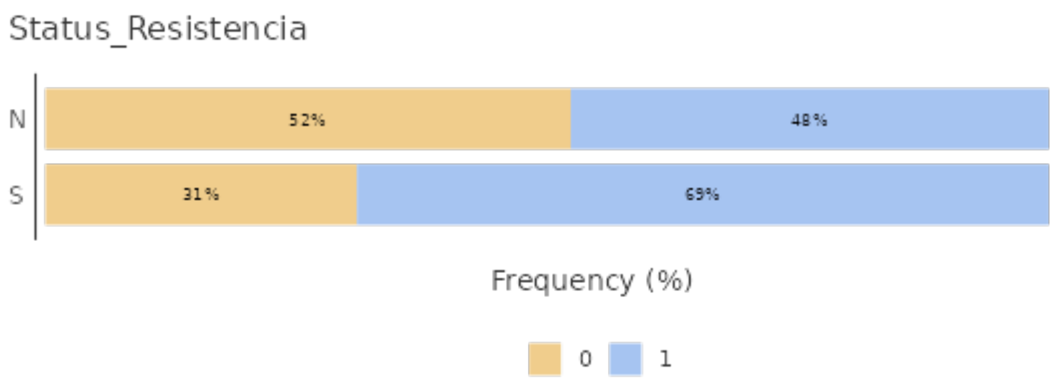

Tabelas de Contingência

Tabelas de Contingência

		Status_Resistencia		
DROGADICAO		0	1	Total
N	Observado	12099	10537	22636
	% em linha	53.5 %	46.5 %	100.0 %
S	Observado	1208	2770	3978
	% em linha	30.4 %	69.6 %	100.0 %
Total	Observado	13307	13307	26614
	% em linha	50.0 %	50.0 %	100.0 %

Testes χ²

	Valor	gl	p
χ^2	721	1	< .001
N	26614		

Survey Plots

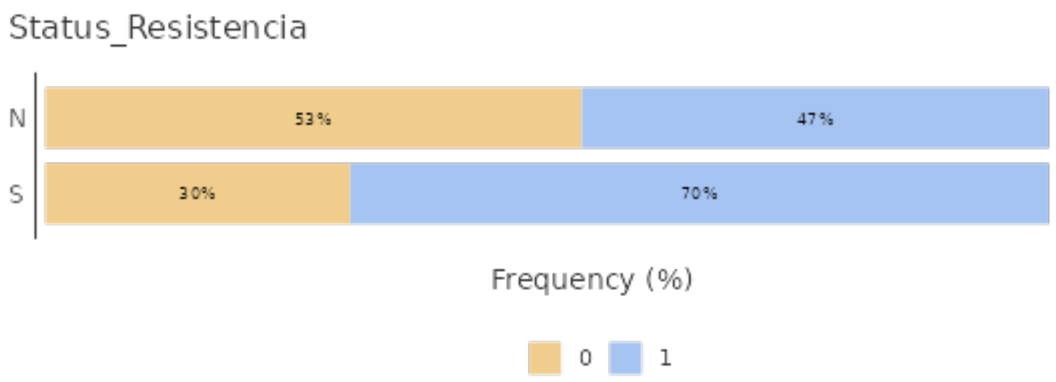

Tabelas de Contingência

Tabelas de Contingência

esqIni		Status_Resistencia 0	1	Total
MR	Observado	0	19	19
	% em linha	0.0 %	100.0 %	100.0 %
OUTROS	Observado	288	493	781
	% em linha	36.9 %	63.1 %	100.0 %
RHZ	Observado	1283	516	1799
	% em linha	71.3 %	28.7 %	100.0 %
RHZE	Observado	11727	12278	24005
	% em linha	48.9 %	51.1 %	100.0 %
Total	Observado	13298	13306	26604
	% em linha	50.0 %	50.0 %	100.0 %

Testes χ^2

	Valor	gl	p
χ^2	412	3	< .001
N	26604		

Survey Plots

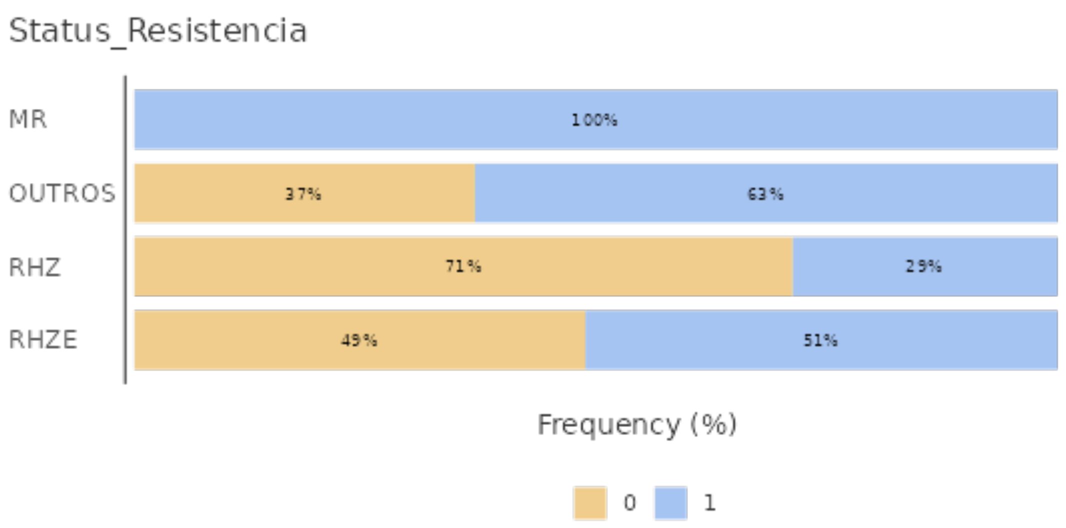

^ MR possui poucas amostras.

Tabelas de Contingência

Tabelas de Contingência

tipoCaso		Status_Resistencia 0	1	Total
Novo	Observado	12780	12124	24904
	% em linha	51.3 %	48.7 %	100.0 %
Recidiva	Observado	317	576	893
	% em linha	35.5 %	64.5 %	100.0 %
Retr Aband	Observado	209	482	691
	% em linha	30.2 %	69.8 %	100.0 %
Retrat apos falencia/resistencia	Observado	1	124	125
	% em linha	0.8 %	99.2 %	100.0 %
Retrat apos mud esquema int/tox	Observado	0	1	1
	% em linha	0.0 %	100.0 %	100.0 %
Total	Observado	13307	13307	26614
	% em linha	50.0 %	50.0 %	100.0 %

Testes χ^2

	Valor	gl	p
χ^2	322	4	< .001
Teste Exato de Fisher			< .001 [a]
N	26614		

[a] Monte Carlo simulation

Survey Plots

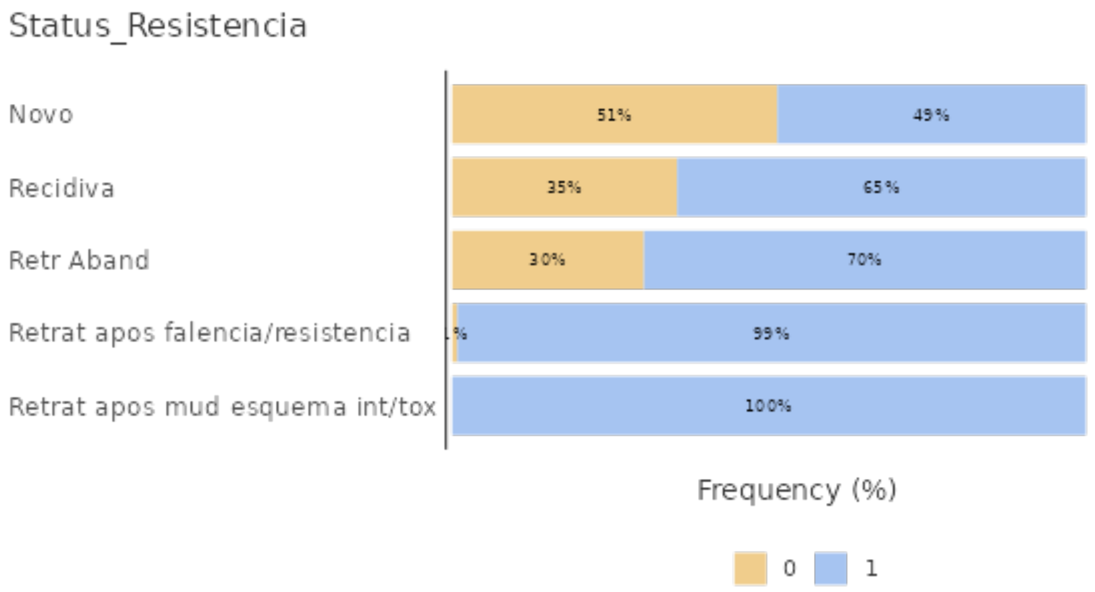

Tabelas de Contingência

Tabelas de Contingência

		Status_Resistencia		
idade		0	1	Total
0-22	Observado	3441	2740	6181
	% em linha	55.7 %	44.3 %	100.0 %
23-39	Observado	3294	3816	7110
	% em linha	46.3 %	53.7 %	100.0 %
40-54	Observado	3255	3692	6947
	% em linha	46.9 %	53.1 %	100.0 %
Mais de 54	Observado	3317	3059	6376
	% em linha	52.0 %	48.0 %	100.0 %
Total	Observado	13307	13307	26614
	% em linha	50.0 %	50.0 %	100.0 %

Testes χ^2

	Valor	gl	p
χ^2	156	3	< .001
N	26614		

Survey Plots

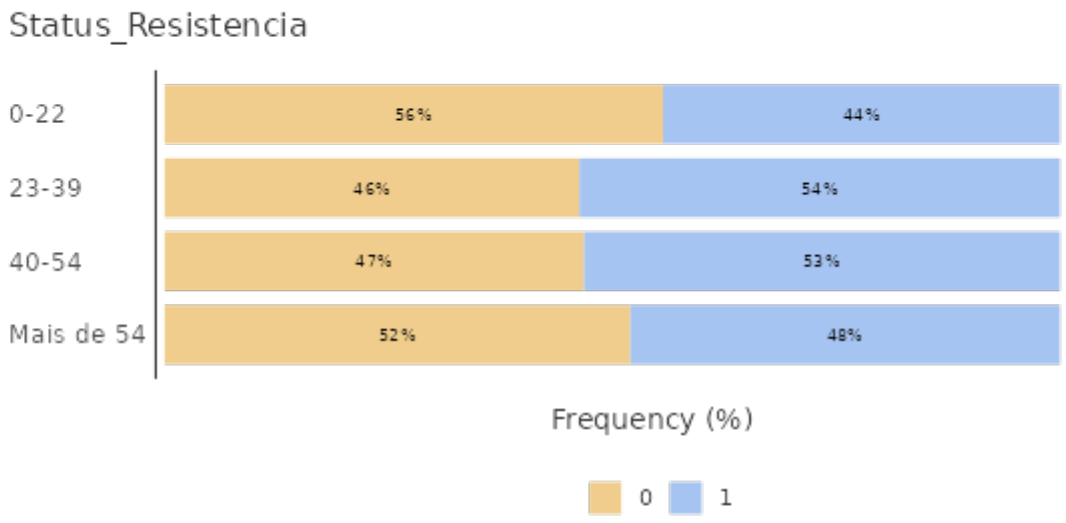

Tabelas de Contingência

Tabelas de Contingência

sitAtual		Status_Resistencia 0	1	Total
Abandono	Observado	1426	2127	3553
	% em linha	40.1 %	59.9 %	100.0 %
Cura	Observado	11881	11180	23061
	% em linha	51.5 %	48.5 %	100.0 %
Total	Observado	13307	13307	26614
	% em linha	50.0 %	50.0 %	100.0 %

Testes χ^2

	Valor	gl	p
χ^2	160	1	< .001
N	26614		

Survey Plots

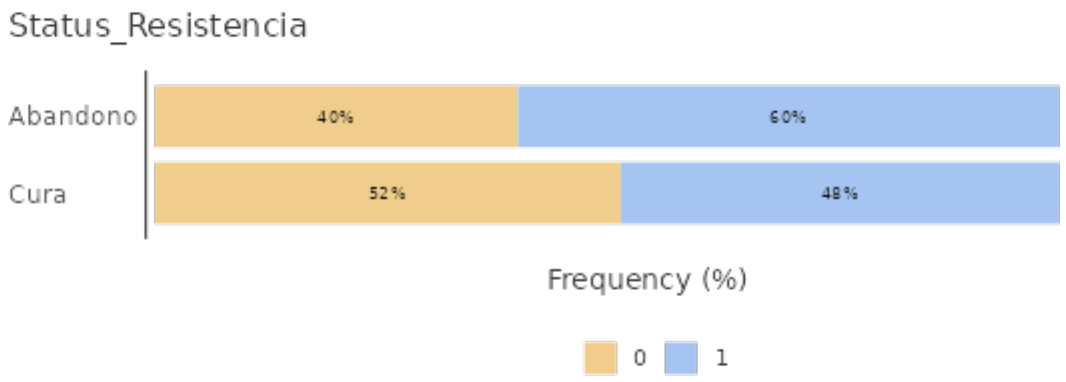

Tabelas de Contingência

Tabelas de Contingência

TIPOCUP		Status_Resistencia 0	Status_Resistencia 1	Total
Aposentado	Observado	849	712	1561
	% em linha	54.4 %	45.6 %	100.0 %
Desempregado	Observado	1637	2581	4218
	% em linha	38.8 %	61.2 %	100.0 %
Dona de Casa	Observado	1128	896	2024
	% em linha	55.7 %	44.3 %	100.0 %
Outra	Observado	7987	7377	15364
	% em linha	52.0 %	48.0 %	100.0 %
Profissional de Saude	Observado	178	244	422
	% em linha	42.2 %	57.8 %	100.0 %
Total	Observado	11779	11810	23589
	% em linha	49.9 %	50.1 %	100.0 %

Testes χ^2

	Valor	gl	p
χ^2	284	4	$< .001$
N	23589		

Survey Plots

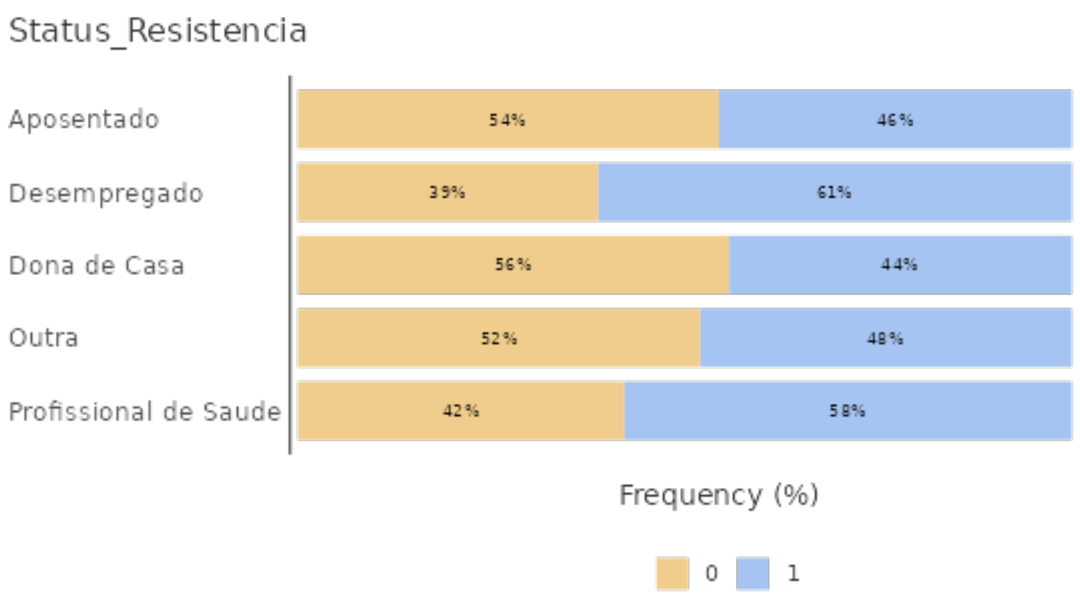

Tabelas de Contingência

Tabelas de Contingência

TABAGISMO		Status_Resistencia 0	Status_Resistencia 1	Total
N	Observado	12238	11324	23562
	% em linha	51.9 %	48.1 %	100.0 %
S	Observado	1069	1983	3052
	% em linha	35.0 %	65.0 %	100.0 %
Total	Observado	13307	13307	26614
	% em linha	50.0 %	50.0 %	100.0 %

Testes χ^2

	Valor	gl	p
χ^2	309	1	< .001
N	26614		

Survey Plots

Status_Resistencia

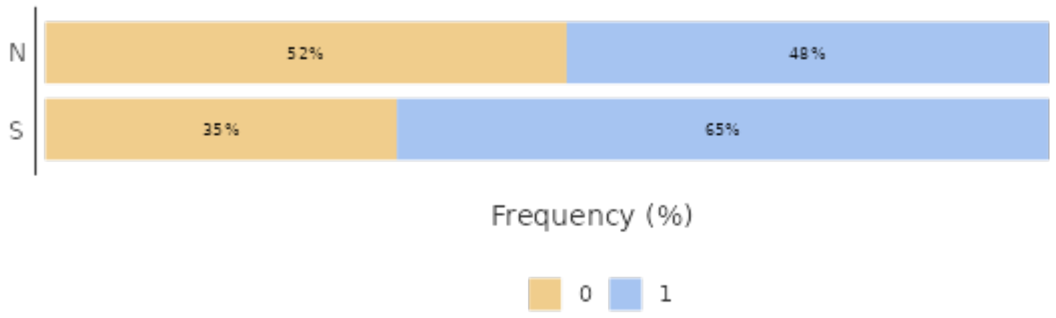

Referências

[1] The jamovi project (2022). *jamovi*. (Version 2.3) [Computer Software]. Retrieved from https://www.jamovi.org.

[2] R Core Team (2021). *R: A Language and environment for statistical computing*. (Version 4.1) [Computer software]. Retrieved from https://cran.r-project.org. (R packages retrieved from MRAN snapshot 2022-01-01).
