# Supplementary material for: Model for predicting drug resistance based on the clinical profile of tuberculosis patients using machine learning techniques
Source: PeerJ Comput Sci. 2024 Oct 14;10:e2246. doi: 10.7717/peerj-cs.2246 (PMC11623081; doi:10.7717/peerj-cs.2246)
Supplement: Supplemental Information 2 [file peerj-cs-10-2246-s002.zip › code/EDA/chi2/Tuberculosis_chi2_and_plot_2.pdf]

Resultados

Tabelas de Contingência

Tabelas de Contingência

FORMACLIN1		Status_Resistencia 0	Status_Resistencia 1	Total
Ganglionar Periferica	Observado	486	173	659
	% em linha	73.7 %	26.3 %	100.0 %
Genital	Observado	23	2	25
	% em linha	92.0 %	8.0 %	100.0 %
Intestinal	Observado	47	16	63
	% em linha	74.6 %	25.4 %	100.0 %
Laringea	Observado	10	2	12
	% em linha	83.3 %	16.7 %	100.0 %
Meningea	Observado	126	53	179
	% em linha	70.4 %	29.6 %	100.0 %
Miliar	Observado	107	57	164
	% em linha	65.2 %	34.8 %	100.0 %
Multiplos Orgaos	Observado	30	40	70
	% em linha	42.9 %	57.1 %	100.0 %
Oftalmica	Observado	141	10	151
	% em linha	93.4 %	6.6 %	100.0 %
Ossea	Observado	108	43	151
	% em linha	71.5 %	28.5 %	100.0 %
Outras	Observado	114	35	149
	% em linha	76.5 %	23.5 %	100.0 %
Pele	Observado	57	11	68
	% em linha	83.8 %	16.2 %	100.0 %
Pleural	Observado	1201	240	1441
	% em linha	83.3 %	16.7 %	100.0 %
Pul	Observado	10804	12586	23390
	% em linha	46.2 %	53.8 %	100.0 %
Vias Urinarias	Observado	53	39	92
	% em linha	57.6 %	42.4 %	100.0 %
Total	Observado	13307	13307	26614
	% em linha	50.0 %	50.0 %	100.0 %

Testes χ^2

	Valor	gl	p
χ^2	1227	13	< .001
N	26614		

Survey Plots

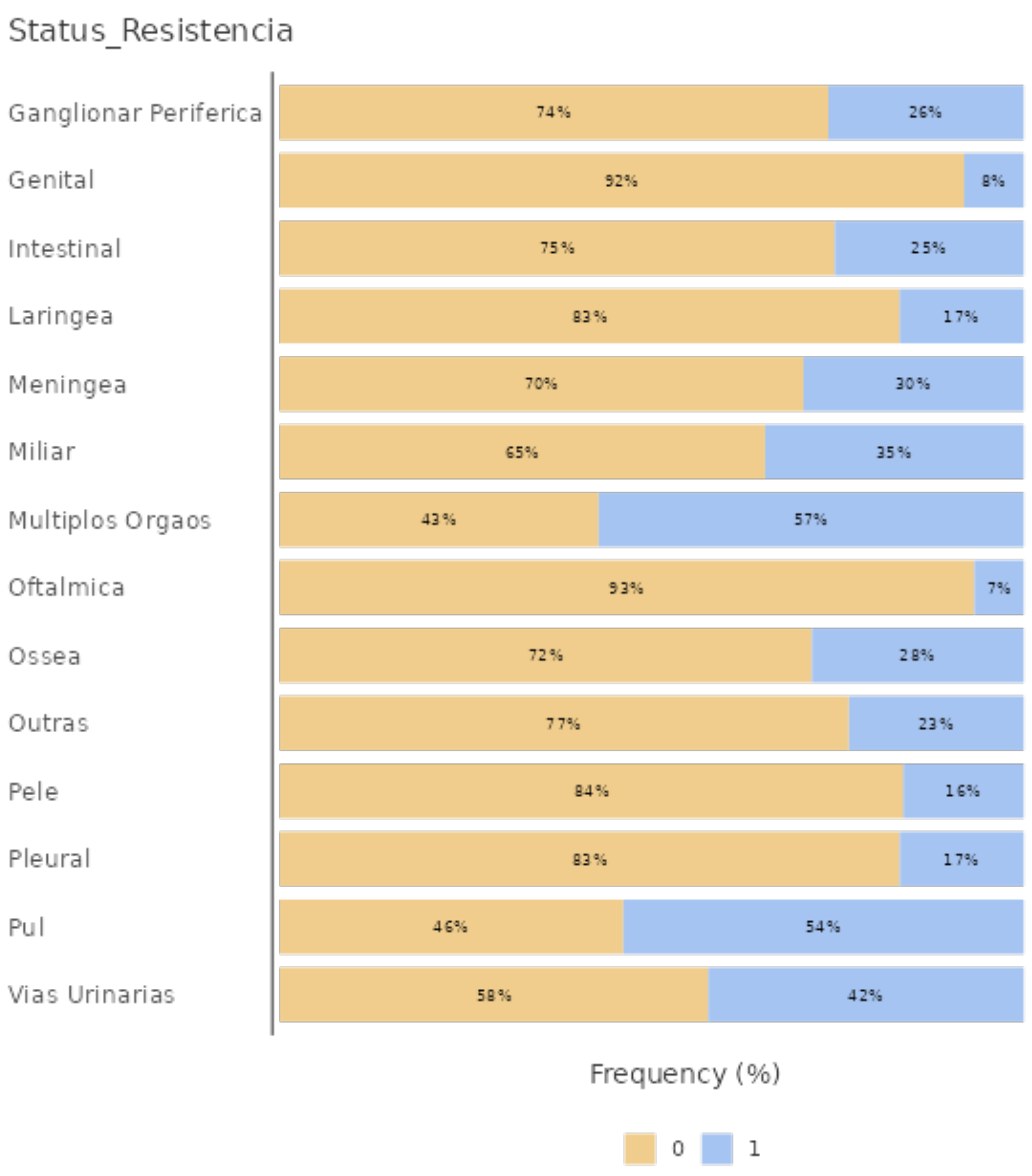

Tabelas de Contingência

Tabelas de Contingência

classif		Status_Resistencia 0	1	Total
Dissem	Observado	30	40	70
	% em linha	42.9 %	57.1 %	100.0 %
Ext	Observado	2473	678	3151
	% em linha	78.5 %	21.5 %	100.0 %
P+E	Observado	400	773	1173
	% em linha	34.1 %	65.9 %	100.0 %
Pul	Observado	10404	11816	22220
	% em linha	46.8 %	53.2 %	100.0 %
Total	Observado	13307	13307	26614
	% em linha	50.0 %	50.0 %	100.0 %

Testes χ^2

	Valor	gl	p
χ^2	1232	3	< .001
N	26614		

Survey Plots

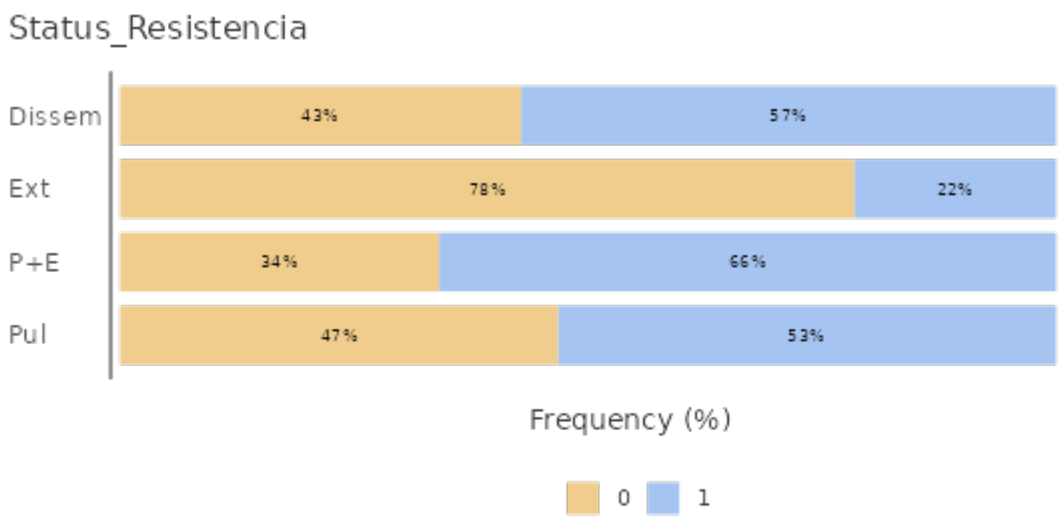

Tabelas de Contingência

Tabelas de Contingência

bac		Status_Resistencia 0	1	Total
And	Observado	32	4	36
	% em linha	88.9 %	11.1 %	100.0 %
N/realiz	Observado	2503	1291	3794
	% em linha	66.0 %	34.0 %	100.0 %
Neg	Observado	3390	2668	6058
	% em linha	56.0 %	44.0 %	100.0 %
Pos	Observado	7265	9279	16544
	% em linha	43.9 %	56.1 %	100.0 %
Total	Observado	13190	13242	26432
	% em linha	49.9 %	50.1 %	100.0 %

Testes χ^2

	Valor	gl	p
χ^2	740	3	< .001
N	26432		

Survey Plots

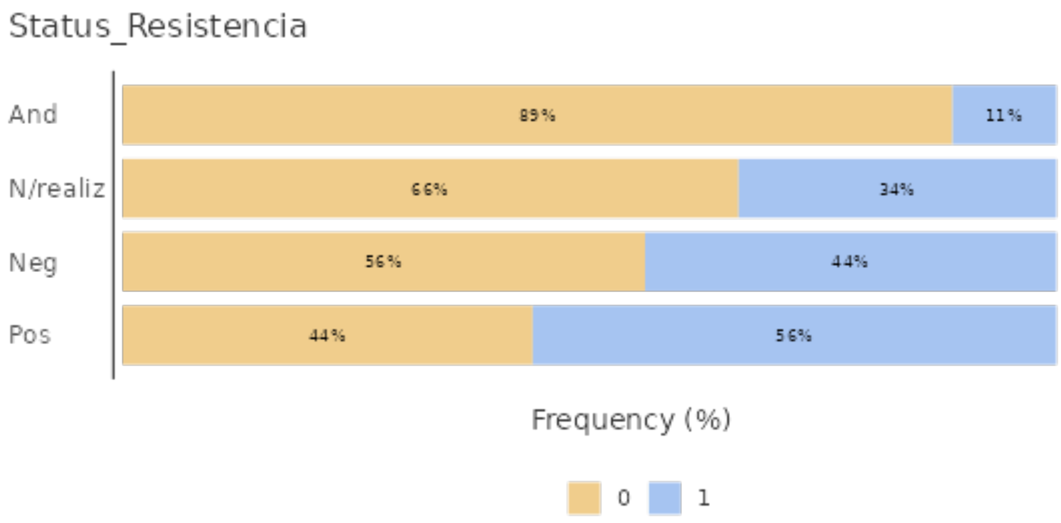

Tabelas de Contingência

Tabelas de Contingência

instTrat		Status_Resistencia		
		0	1	Total
Albergue	Observado	68	266	334
	% em linha	20.4 %	79.6 %	100.0 %
Asilo	Observado	20	4	24
	% em linha	83.3 %	16.7 %	100.0 %
Casa de Apoio/Recuperacao	Observado	1	4	5
	% em linha	20.0 %	80.0 %	100.0 %
Hosp. Psiq.	Observado	20	33	53
	% em linha	37.7 %	62.3 %	100.0 %
Instit. Penal	Observado	96	496	592
	% em linha	16.2 %	83.8 %	100.0 %
N	Observado	12655	11518	24173
	% em linha	52.4 %	47.6 %	100.0 %
Orfanato	Observado	2	0	2
	% em linha	100.0 %	0.0 %	100.0 %
Total	Observado	12862	12321	25183
	% em linha	51.1 %	48.9 %	100.0 %

Testes χ^2

	Valor	gl	p
χ^2	447	6	< .001
Teste Exato de Fisher			< .001 [a]
N	25183		

[a] Monte Carlo simulation

Survey Plots

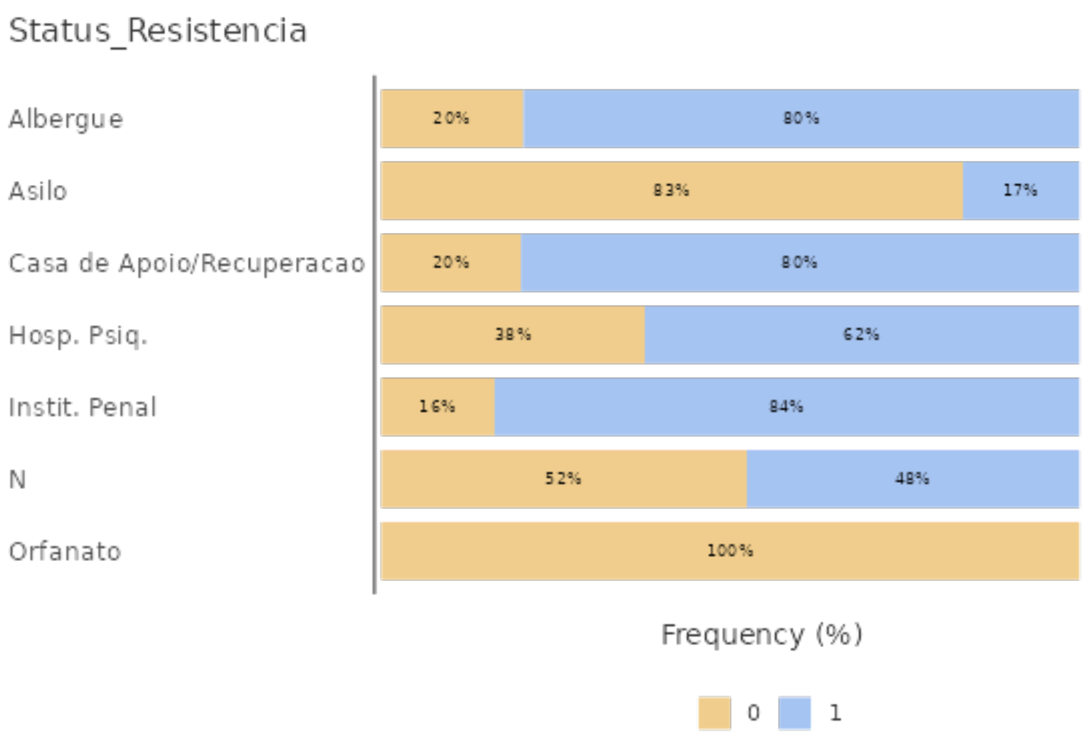

Tabelas de Contingência

Tabelas de Contingência

RX		Status_Resistencia 0	1	Total
N/realiz	Observado	1528	1804	3332
	% em linha	45.9 %	54.1 %	100.0 %
Normal	Observado	1025	640	1665
	% em linha	61.6 %	38.4 %	100.0 %
Outra Patologia	Observado	120	95	215
	% em linha	55.8 %	44.2 %	100.0 %
Susp TB	Observado	7879	7723	15602
	% em linha	50.5 %	49.5 %	100.0 %
Susp c/cavid	Observado	2117	2407	4524
	% em linha	46.8 %	53.2 %	100.0 %
Total	Observado	12669	12669	25338
	% em linha	50.0 %	50.0 %	100.0 %

Testes χ^2

	Valor	gl	p
χ^2	135	4	< .001
N	25338		

Survey Plots

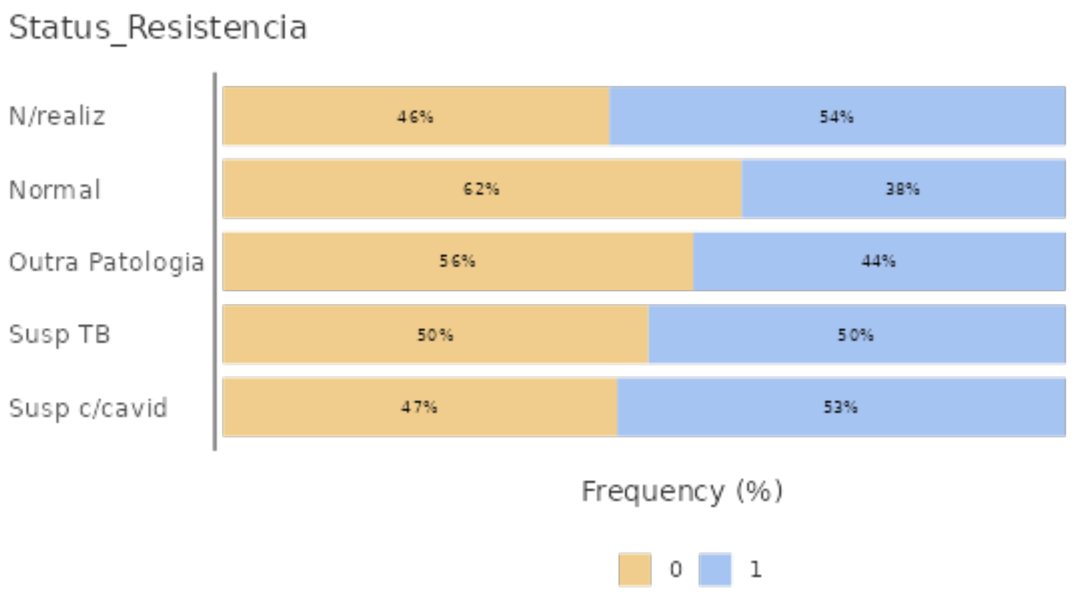

Tabelas de Contingência

Tabelas de Contingência

		Status_Resistencia		
**aids**		**0**	**1**	**Total**
N	Observado	12394	11279	23673
	% em linha	52.4 %	47.6 %	100.0 %
S	Observado	913	2028	2941
	% em linha	31.0 %	69.0 %	100.0 %
Total	Observado	13307	13307	26614
	% em linha	50.0 %	50.0 %	100.0 %

Testes χ^2

	**Valor**	**gl**	**p**
χ^2	475	1	$< .001$
N	26614		

Survey Plots

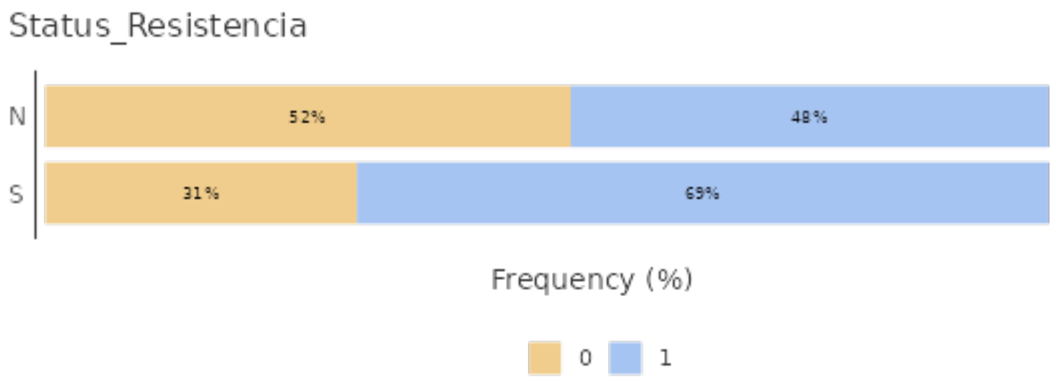

Tabelas de Contingência

Tabelas de Contingência

descoberta		Status_Resistencia		
		0	1	Total
Busca Ativa em Instituicao	Observado	172	433	605
	% em linha	28.4 %	71.6 %	100.0 %
Busca Ativa na Comunidade	Observado	352	419	771
	% em linha	45.7 %	54.3 %	100.0 %
Demanda Ambulatorial	Observado	6843	6395	13238
	% em linha	51.7 %	48.3 %	100.0 %
Elucidacao Diagn. em Internacao	Observado	2383	2540	4923
	% em linha	48.4 %	51.6 %	100.0 %
Investigacao de Contatos	Observado	480	315	795
	% em linha	60.4 %	39.6 %	100.0 %
Urgencia / Emergencia	Observado	2859	3071	5930
	% em linha	48.2 %	51.8 %	100.0 %
Total	Observado	13089	13173	26262
	% em linha	49.8 %	50.2 %	100.0 %

Testes χ^2

	Valor	gl	p
χ^2	180	5	< .001
N	26262		

Survey Plots

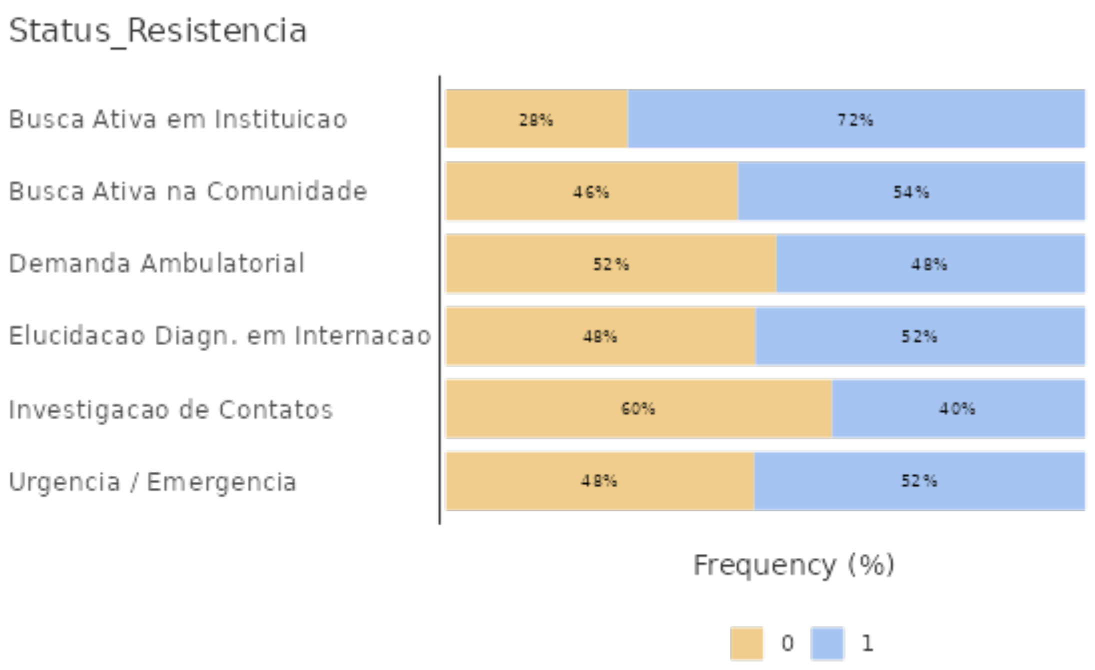

Tabelas de Contingência

Tabelas de Contingência

		Status_Resistencia		
hiv		0	1	Total
And	Observado	86	54	140
	% em linha	61.4 %	38.6 %	100.0 %
N/realiz	Observado	1259	884	2143
	% em linha	58.7 %	41.3 %	100.0 %
Neg	Observado	10724	10069	20793
	% em linha	51.6 %	48.4 %	100.0 %
Pos	Observado	1004	2164	3168
	% em linha	31.7 %	68.3 %	100.0 %
Total	Observado	13073	13171	26244
	% em linha	49.8 %	50.2 %	100.0 %

Testes χ^2

	Valor	gl	p
χ^2	518	3	< .001
N	26244		

Survey Plots

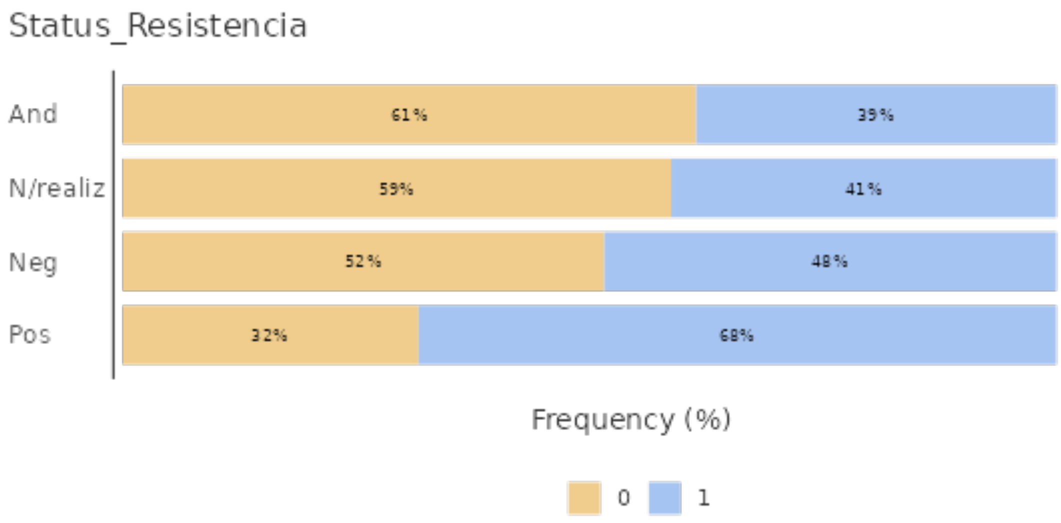

Tabelas de Contingência

Tabelas de Contingência

sexo		Status_Resistencia 0	1	Total
F	Observado	4604	3562	8166
	% em linha	56.4 %	43.6 %	100.0 %
M	Observado	8703	9745	18448
	% em linha	47.2 %	52.8 %	100.0 %
Total	Observado	13307	13307	26614
	% em linha	50.0 %	50.0 %	100.0 %

Testes χ^2

	Valor	gl	p
χ^2	192	1	< .001
N	26614		

Survey Plots

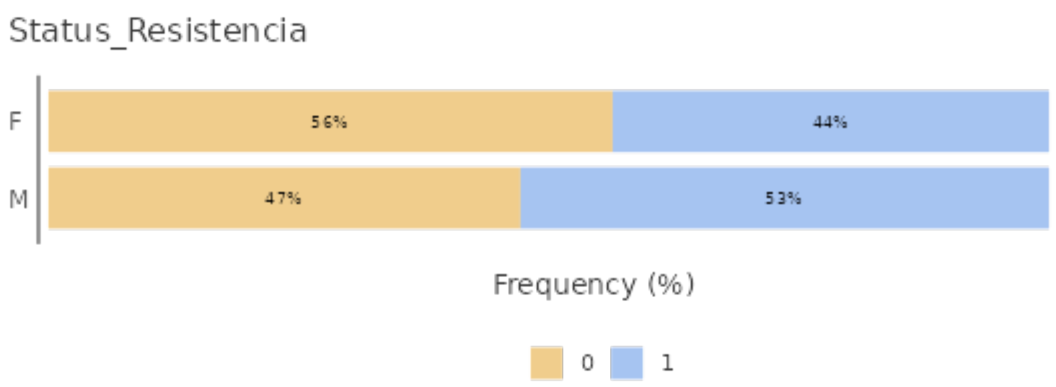

Tabelas de Contingência

Tabelas de Contingência

faixaEtaria		Status_Resistencia 0	1	Total
01_04	Observado	166	25	191
	% em linha	86.9 %	13.1 %	100.0 %
05_09	Observado	138	25	163
	% em linha	84.7 %	15.3 %	100.0 %
10_14	Observado	225	137	362
	% em linha	62.2 %	37.8 %	100.0 %
15_19	Observado	942	647	1589
	% em linha	59.3 %	40.7 %	100.0 %
20_29	Observado	3187	3299	6486
	% em linha	49.1 %	50.9 %	100.0 %
30_39	Observado	2761	3328	6089
	% em linha	45.3 %	54.7 %	100.0 %
40_49	Observado	2512	2763	5275
	% em linha	47.6 %	52.4 %	100.0 %
50_59	Observado	1855	1920	3775
	% em linha	49.1 %	50.9 %	100.0 %
60_69	Observado	956	806	1762
	% em linha	54.3 %	45.7 %	100.0 %
70_79	Observado	376	263	639
	% em linha	58.8 %	41.2 %	100.0 %
Maior de 80 anos	Observado	130	70	200
	% em linha	65.0 %	35.0 %	100.0 %
Menor de 1 ano	Observado	59	24	83
	% em linha	71.1 %	28.9 %	100.0 %
Total	Observado	13307	13307	26614
	% em linha	50.0 %	50.0 %	100.0 %

Testes χ^2

	Valor	gl	p
χ^2	392	11	< .001
N	26614		

Survey Plots

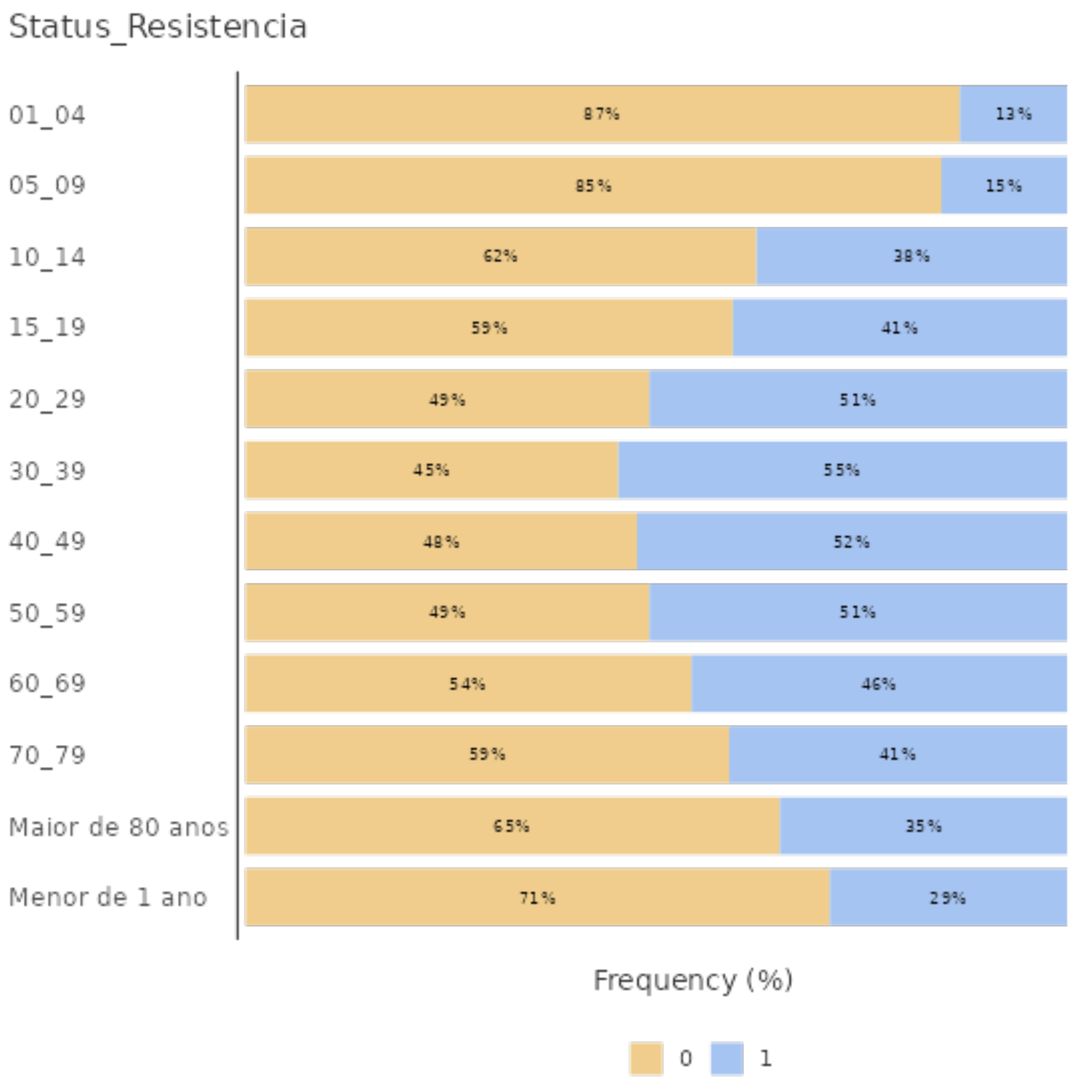

Tabelas de Contingência

Tabelas de Contingência

ALCOOLISMO		Status_Resistencia 0	Status_Resistencia 1	Total
N	Observado	11477	10106	21583
	% em linha	53.2 %	46.8 %	100.0 %
S	Observado	1830	3201	5031
	% em linha	36.4 %	63.6 %	100.0 %
Total	Observado	13307	13307	26614
	% em linha	50.0 %	50.0 %	100.0 %

Testes χ^2

	Valor	gl	p
χ^2	461	1	< .001
N	26614		

Survey Plots

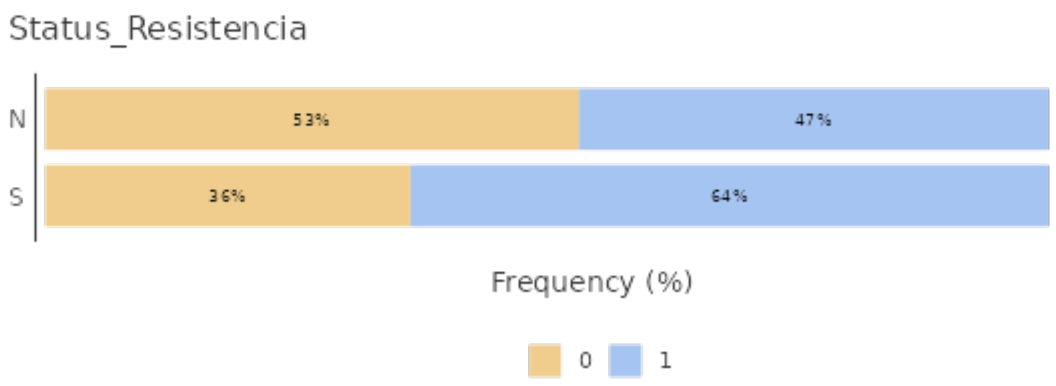

Tabelas de Contingência

Tabelas de Contingência

HISTOPATOL		Status_Resistencia 0	Status_Resistencia 1	Total
BAAR pos	Observado	315	263	578
	% em linha	54.5 %	45.5 %	100.0 %
N/realiz	Observado	9468	10403	19871
	% em linha	47.6 %	52.4 %	100.0 %
Sugestivo TB	Observado	969	360	1329
	% em linha	72.9 %	27.1 %	100.0 %
Total	Observado	10752	11026	21778
	% em linha	49.4 %	50.6 %	100.0 %

Testes χ^2

	Valor	gl	p
χ^2	324	2	< .001
N	21778		

Survey Plots

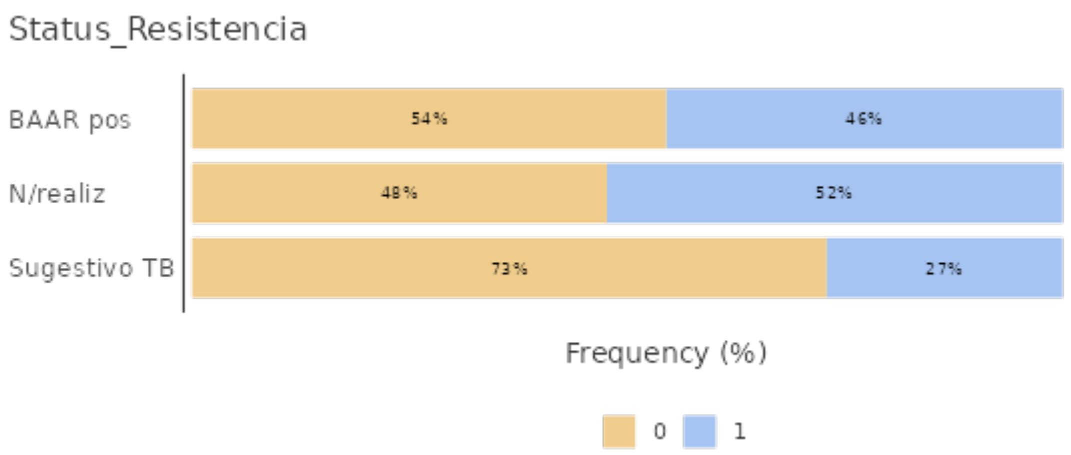

Referências

[1] The jamovi project (2022). *jamovi*. (Version 2.3) [Computer Software]. Retrieved from https://www.jamovi.org.

[2] R Core Team (2021). *R: A Language and environment for statistical computing*. (Version 4.1) [Computer software]. Retrieved from https://cran.r-project.org. (R packages retrieved from MRAN snapshot 2022-01-01).
