# Supplementary material for: Model for predicting drug resistance based on the clinical profile of tuberculosis patients using machine learning techniques
Source: PeerJ Comput Sci. 2024 Oct 14;10:e2246. doi: 10.7717/peerj-cs.2246 (PMC11623081; doi:10.7717/peerj-cs.2246)
Supplement: Supplemental Information 2 [file peerj-cs-10-2246-s002.zip › code/EDA/false_negative_1.html]

Pandas Profiling Report 

Toggle navigationPandas Profiling Report

- Overview
- Variables
- Correlations
- Missing values
- Sample

# Overview

- Overview
- Alerts 38
- Reproduction

Dataset statistics

|  |  |
| --- | --- |
| Number of variables | 28 |
| Number of observations | 3 |
| Missing cells | 0 |
| Missing cells (%) | 0.0% |
| Duplicate rows | 0 |
| Duplicate rows (%) | 0.0% |
| Total size in memory | 781.0 B |
| Average record size in memory | 260.3 B |

Variable types

|  |  |
| --- | --- |
| Categorical | 24 |
| Boolean | 4 |

Alerts

| `tipoCaso` has constant value "Novo" | Constant |
| `cultEsc` has constant value "N/realiz" | Constant |
| `NECROP` has constant value "N/realiz" | Constant |
| `hiv` has constant value "Pos" | Constant |
| `aids` has constant value "S" | Constant |
| `DIABETES` has constant value "False" | Constant |
| `ALCOOLISMO` has constant value "False" | Constant |
| `MENTAL` has constant value "False" | Constant |
| `DROGADICAO` has constant value "False" | Constant |
| `motMudEsquema` has constant value "Nulo" | Constant |
| `HISTOPATOL` has constant value "N/realiz" | Constant |
| `Status_Resistencia` has constant value "1" | Constant |
| `Cluster` has constant value "0" | Constant |
| `faixaEtaria` is highly overall correlated with `sexo` and 13 other fields | High correlation |
| `sexo` is highly overall correlated with `faixaEtaria` and 4 other fields | High correlation |
| `ESCOLARID` is highly overall correlated with `faixaEtaria` and 4 other fields | High correlation |
| `TIPOCUP` is highly overall correlated with `faixaEtaria` and 4 other fields | High correlation |
| `sitAtual` is highly overall correlated with `faixaEtaria` and 4 other fields | High correlation |
| `FORMACLIN1` is highly overall correlated with `faixaEtaria` and 4 other fields | High correlation |
| `classif` is highly overall correlated with `faixaEtaria` and 13 other fields | High correlation |
| `descoberta` is highly overall correlated with `faixaEtaria` and 4 other fields | High correlation |
| `bac` is highly overall correlated with `faixaEtaria` and 13 other fields | High correlation |
| `BACOUTRO` is highly overall correlated with `faixaEtaria` and 4 other fields | High correlation |
| `RX` is highly overall correlated with `faixaEtaria` and 4 other fields | High correlation |
| `TABAGISMO` is highly overall correlated with `faixaEtaria` and 4 other fields | High correlation |
| `tipoTrat` is highly overall correlated with `faixaEtaria` and 4 other fields | High correlation |
| `idade` is highly overall correlated with `faixaEtaria` and 13 other fields | High correlation |
| `Probabilidade` is highly overall correlated with `faixaEtaria` and 13 other fields | High correlation |
| `faixaEtaria` is uniformly distributed | Uniform |
| `classif` is uniformly distributed | Uniform |
| `bac` is uniformly distributed | Uniform |
| `idade` is uniformly distributed | Uniform |
| `Probabilidade` is uniformly distributed | Uniform |
| `faixaEtaria` has unique values | Unique |
| `classif` has unique values | Unique |
| `bac` has unique values | Unique |
| `idade` has unique values | Unique |
| `Probabilidade` has unique values | Unique |

Reproduction

|  |  |
| --- | --- |
| Analysis started | 2023-10-31 19:30:00.620074 |
| Analysis finished | 2023-10-31 19:30:03.300165 |
| Duration | 2.68 seconds |
| Software version | pandas-profiling v3.6.6 |
| Download configuration | config.json |

# Variables

Select ColumnsfaixaEtariasexoESCOLARIDTIPOCUPsitAtualtipoCasoFORMACLIN1classifdescobertabacBACOUTROcultEscRXNECROPhivaidsDIABETESALCOOLISMOMENTALDROGADICAOTABAGISMOmotMudEsquematipoTratidadeHISTOPATOLStatus\_ResistenciaClusterProbabilidade

faixaEtaria  
Categorical

`HIGH CORRELATION`  `UNIFORM`  `UNIQUE`

|  |  |
| --- | --- |
| Distinct | 3 |
| Distinct (%) | 100.0% |
| Missing | 0 |
| Missing (%) | 0.0% |
| Memory size | 48.0 B |

|  |  |
| --- | --- |
| 20\_29 | 1 |
| 40\_49 | 1 |
| 30\_39 | 1 |

More details

- Overview
- Categories
- Words
- Characters

Length

|  |  |
| --- | --- |
| Max length | 5 |
| Median length | 5 |
| Mean length | 5 |
| Min length | 5 |

Characters and Unicode

|  |  |
| --- | --- |
| Total characters | 15 |
| Distinct characters | 6 |
| Distinct categories | 2 ? |
| Distinct scripts | 1 ? |
| Distinct blocks | 1 ? |

The Unicode Standard assigns character properties to each code point, which can be used to analyse textual variables.

Unique

|  |  |
| --- | --- |
| Unique | 3 ? |
| Unique (%) | 100.0% |

Sample

|  |  |
| --- | --- |
| 1st row | 20\_29 |
| 2nd row | 40\_49 |
| 3rd row | 30\_39 |

#### Common Values

| Value | Count | Frequency (%) |
| --- | --- | --- |
| 20\_29 | 1 | 33.3% |
| 40\_49 | 1 | 33.3% |
| 30\_39 | 1 | 33.3% |

#### Length

xml version="1.0" encoding="utf-8" standalone="no"?2023-10-31T16:30:03.814173image/svg+xmlMatplotlib v3.6.0, https://matplotlib.org/ 

Histogram of lengths of the category

#### Common Values (Plot)

xml version="1.0" encoding="utf-8" standalone="no"?2023-10-31T16:30:03.956420image/svg+xmlMatplotlib v3.6.0, https://matplotlib.org/

| Value | Count | Frequency (%) |
| --- | --- | --- |
| 20\_29 | 1 | 33.3% |
| 40\_49 | 1 | 33.3% |
| 30\_39 | 1 | 33.3% |

- Characters
- Categories
- Scripts
- Blocks

#### Most occurring characters

| Value | Count | Frequency (%) |
| --- | --- | --- |
| 0 | 3 | 20.0% |
| \_ | 3 | 20.0% |
| 9 | 3 | 20.0% |
| 2 | 2 | 13.3% |
| 4 | 2 | 13.3% |
| 3 | 2 | 13.3% |

#### Most occurring categories

| Value | Count | Frequency (%) |
| --- | --- | --- |
| Decimal Number | 12 | 80.0% |
| Connector Punctuation | 3 | 20.0% |

#### Most frequent character per category

##### *Decimal Number*

| Value | Count | Frequency (%) |
| --- | --- | --- |
| 0 | 3 | 25.0% |
| 9 | 3 | 25.0% |
| 2 | 2 | 16.7% |
| 4 | 2 | 16.7% |
| 3 | 2 | 16.7% |

##### *Connector Punctuation*

| Value | Count | Frequency (%) |
| --- | --- | --- |
| \_ | 3 | 100.0% |

#### Most occurring scripts

| Value | Count | Frequency (%) |
| --- | --- | --- |
| Common | 15 | 100.0% |

#### Most frequent character per script

##### *Common*

| Value | Count | Frequency (%) |
| --- | --- | --- |
| 0 | 3 | 20.0% |
| \_ | 3 | 20.0% |
| 9 | 3 | 20.0% |
| 2 | 2 | 13.3% |
| 4 | 2 | 13.3% |
| 3 | 2 | 13.3% |

#### Most occurring blocks

| Value | Count | Frequency (%) |
| --- | --- | --- |
| ASCII | 15 | 100.0% |

#### Most frequent character per block

##### *ASCII*

| Value | Count | Frequency (%) |
| --- | --- | --- |
| 0 | 3 | 20.0% |
| \_ | 3 | 20.0% |
| 9 | 3 | 20.0% |
| 2 | 2 | 13.3% |
| 4 | 2 | 13.3% |
| 3 | 2 | 13.3% |

sexo  
Categorical

|  |  |
| --- | --- |
| Distinct | 2 |
| Distinct (%) | 66.7% |
| Missing | 0 |
| Missing (%) | 0.0% |
| Memory size | 151.0 B |

|  |  |
| --- | --- |
| F | 2 |
| M | 1 |

More details

- Overview
- Categories
- Words
- Characters

Length

|  |  |
| --- | --- |
| Max length | 1 |
| Median length | 1 |
| Mean length | 1 |
| Min length | 1 |

Characters and Unicode

|  |  |
| --- | --- |
| Total characters | 3 |
| Distinct characters | 2 |
| Distinct categories | 1 ? |
| Distinct scripts | 1 ? |
| Distinct blocks | 1 ? |

The Unicode Standard assigns character properties to each code point, which can be used to analyse textual variables.

Unique

|  |  |
| --- | --- |
| Unique | 1 ? |
| Unique (%) | 33.3% |

Sample

|  |  |
| --- | --- |
| 1st row | F |
| 2nd row | M |
| 3rd row | F |

#### Common Values

| Value | Count | Frequency (%) |
| --- | --- | --- |
| F | 2 | 66.7% |
| M | 1 | 33.3% |

#### Length

xml version="1.0" encoding="utf-8" standalone="no"?2023-10-31T16:30:04.078051image/svg+xmlMatplotlib v3.6.0, https://matplotlib.org/ 

Histogram of lengths of the category

#### Common Values (Plot)

xml version="1.0" encoding="utf-8" standalone="no"?2023-10-31T16:30:04.214025image/svg+xmlMatplotlib v3.6.0, https://matplotlib.org/

| Value | Count | Frequency (%) |
| --- | --- | --- |
| f | 2 | 66.7% |
| m | 1 | 33.3% |

- Characters
- Categories
- Scripts
- Blocks

#### Most occurring characters

| Value | Count | Frequency (%) |
| --- | --- | --- |
| F | 2 | 66.7% |
| M | 1 | 33.3% |

#### Most occurring categories

| Value | Count | Frequency (%) |
| --- | --- | --- |
| Uppercase Letter | 3 | 100.0% |

#### Most frequent character per category

##### *Uppercase Letter*

| Value | Count | Frequency (%) |
| --- | --- | --- |
| F | 2 | 66.7% |
| M | 1 | 33.3% |

#### Most occurring scripts

| Value | Count | Frequency (%) |
| --- | --- | --- |
| Latin | 3 | 100.0% |

#### Most frequent character per script

##### *Latin*

| Value | Count | Frequency (%) |
| --- | --- | --- |
| F | 2 | 66.7% |
| M | 1 | 33.3% |

#### Most occurring blocks

| Value | Count | Frequency (%) |
| --- | --- | --- |
| ASCII | 3 | 100.0% |

#### Most frequent character per block

##### *ASCII*

| Value | Count | Frequency (%) |
| --- | --- | --- |
| F | 2 | 66.7% |
| M | 1 | 33.3% |

ESCOLARID  
Categorical

|  |  |
| --- | --- |
| Distinct | 2 |
| Distinct (%) | 66.7% |
| Missing | 0 |
| Missing (%) | 0.0% |
| Memory size | 48.0 B |

|  |  |
| --- | --- |
| De 4 a 7 anos | 2 |
| De 8 a 11 anos | 1 |

More details

- Overview
- Categories
- Words
- Characters

Length

|  |  |
| --- | --- |
| Max length | 14 |
| Median length | 13 |
| Mean length | 13.333333 |
| Min length | 13 |

Characters and Unicode

|  |  |
| --- | --- |
| Total characters | 40 |
| Distinct characters | 11 |
| Distinct categories | 4 ? |
| Distinct scripts | 2 ? |
| Distinct blocks | 1 ? |

The Unicode Standard assigns character properties to each code point, which can be used to analyse textual variables.

Unique

|  |  |
| --- | --- |
| Unique | 1 ? |
| Unique (%) | 33.3% |

Sample

|  |  |
| --- | --- |
| 1st row | De 4 a 7 anos |
| 2nd row | De 8 a 11 anos |
| 3rd row | De 4 a 7 anos |

#### Common Values

| Value | Count | Frequency (%) |
| --- | --- | --- |
| De 4 a 7 anos | 2 | 66.7% |
| De 8 a 11 anos | 1 | 33.3% |

#### Length

xml version="1.0" encoding="utf-8" standalone="no"?2023-10-31T16:30:04.329359image/svg+xmlMatplotlib v3.6.0, https://matplotlib.org/ 

Histogram of lengths of the category

#### Common Values (Plot)

xml version="1.0" encoding="utf-8" standalone="no"?2023-10-31T16:30:04.485124image/svg+xmlMatplotlib v3.6.0, https://matplotlib.org/

| Value | Count | Frequency (%) |
| --- | --- | --- |
| de | 3 | 20.0% |
| a | 3 | 20.0% |
| anos | 3 | 20.0% |
| 4 | 2 | 13.3% |
| 7 | 2 | 13.3% |
| 8 | 1 | 6.7% |
| 11 | 1 | 6.7% |

- Characters
- Categories
- Scripts
- Blocks

#### Most occurring characters

| Value | Count | Frequency (%) |
| --- | --- | --- |
|  | 12 | 30.0% |
| a | 6 | 15.0% |
| D | 3 | 7.5% |
| e | 3 | 7.5% |
| n | 3 | 7.5% |
| o | 3 | 7.5% |
| s | 3 | 7.5% |
| 4 | 2 | 5.0% |
| 7 | 2 | 5.0% |
| 1 | 2 | 5.0% |

#### Most occurring categories

| Value | Count | Frequency (%) |
| --- | --- | --- |
| Lowercase Letter | 18 | 45.0% |
| Space Separator | 12 | 30.0% |
| Decimal Number | 7 | 17.5% |
| Uppercase Letter | 3 | 7.5% |

#### Most frequent character per category

##### *Lowercase Letter*

| Value | Count | Frequency (%) |
| --- | --- | --- |
| a | 6 | 33.3% |
| e | 3 | 16.7% |
| n | 3 | 16.7% |
| o | 3 | 16.7% |
| s | 3 | 16.7% |

##### *Decimal Number*

| Value | Count | Frequency (%) |
| --- | --- | --- |
| 4 | 2 | 28.6% |
| 7 | 2 | 28.6% |
| 1 | 2 | 28.6% |
| 8 | 1 | 14.3% |

##### *Space Separator*

| Value | Count | Frequency (%) |
| --- | --- | --- |
|  | 12 | 100.0% |

##### *Uppercase Letter*

| Value | Count | Frequency (%) |
| --- | --- | --- |
| D | 3 | 100.0% |

#### Most occurring scripts

| Value | Count | Frequency (%) |
| --- | --- | --- |
| Latin | 21 | 52.5% |
| Common | 19 | 47.5% |

#### Most frequent character per script

##### *Latin*

| Value | Count | Frequency (%) |
| --- | --- | --- |
| a | 6 | 28.6% |
| D | 3 | 14.3% |
| e | 3 | 14.3% |
| n | 3 | 14.3% |
| o | 3 | 14.3% |
| s | 3 | 14.3% |

##### *Common*

| Value | Count | Frequency (%) |
| --- | --- | --- |
|  | 12 | 63.2% |
| 4 | 2 | 10.5% |
| 7 | 2 | 10.5% |
| 1 | 2 | 10.5% |
| 8 | 1 | 5.3% |

#### Most occurring blocks

| Value | Count | Frequency (%) |
| --- | --- | --- |
| ASCII | 40 | 100.0% |

#### Most frequent character per block

##### *ASCII*

| Value | Count | Frequency (%) |
| --- | --- | --- |
|  | 12 | 30.0% |
| a | 6 | 15.0% |
| D | 3 | 7.5% |
| e | 3 | 7.5% |
| n | 3 | 7.5% |
| o | 3 | 7.5% |
| s | 3 | 7.5% |
| 4 | 2 | 5.0% |
| 7 | 2 | 5.0% |
| 1 | 2 | 5.0% |

TIPOCUP  
Categorical

|  |  |
| --- | --- |
| Distinct | 2 |
| Distinct (%) | 66.7% |
| Missing | 0 |
| Missing (%) | 0.0% |
| Memory size | 48.0 B |

|  |  |
| --- | --- |
| Desempregado | 2 |
| Dona de Casa | 1 |

More details

- Overview
- Categories
- Words
- Characters

Length

|  |  |
| --- | --- |
| Max length | 12 |
| Median length | 12 |
| Mean length | 12 |
| Min length | 12 |

Characters and Unicode

|  |  |
| --- | --- |
| Total characters | 36 |
| Distinct characters | 13 |
| Distinct categories | 3 ? |
| Distinct scripts | 2 ? |
| Distinct blocks | 1 ? |

The Unicode Standard assigns character properties to each code point, which can be used to analyse textual variables.

Unique

|  |  |
| --- | --- |
| Unique | 1 ? |
| Unique (%) | 33.3% |

Sample

|  |  |
| --- | --- |
| 1st row | Dona de Casa |
| 2nd row | Desempregado |
| 3rd row | Desempregado |

#### Common Values

| Value | Count | Frequency (%) |
| --- | --- | --- |
| Desempregado | 2 | 66.7% |
| Dona de Casa | 1 | 33.3% |

#### Length

xml version="1.0" encoding="utf-8" standalone="no"?2023-10-31T16:30:04.621313image/svg+xmlMatplotlib v3.6.0, https://matplotlib.org/ 

Histogram of lengths of the category

#### Common Values (Plot)

xml version="1.0" encoding="utf-8" standalone="no"?2023-10-31T16:30:04.816391image/svg+xmlMatplotlib v3.6.0, https://matplotlib.org/

| Value | Count | Frequency (%) |
| --- | --- | --- |
| desempregado | 2 | 40.0% |
| dona | 1 | 20.0% |
| de | 1 | 20.0% |
| casa | 1 | 20.0% |

- Characters
- Categories
- Scripts
- Blocks

#### Most occurring characters

| Value | Count | Frequency (%) |
| --- | --- | --- |
| e | 7 | 19.4% |
| a | 5 | 13.9% |
| D | 3 | 8.3% |
| s | 3 | 8.3% |
| d | 3 | 8.3% |
| o | 3 | 8.3% |
| m | 2 | 5.6% |
| p | 2 | 5.6% |
| r | 2 | 5.6% |
| g | 2 | 5.6% |
| Other values (3) | 4 | 11.1% |

#### Most occurring categories

| Value | Count | Frequency (%) |
| --- | --- | --- |
| Lowercase Letter | 30 | 83.3% |
| Uppercase Letter | 4 | 11.1% |
| Space Separator | 2 | 5.6% |

#### Most frequent character per category

##### *Lowercase Letter*

| Value | Count | Frequency (%) |
| --- | --- | --- |
| e | 7 | 23.3% |
| a | 5 | 16.7% |
| s | 3 | 10.0% |
| d | 3 | 10.0% |
| o | 3 | 10.0% |
| m | 2 | 6.7% |
| p | 2 | 6.7% |
| r | 2 | 6.7% |
| g | 2 | 6.7% |
| n | 1 | 3.3% |

##### *Uppercase Letter*

| Value | Count | Frequency (%) |
| --- | --- | --- |
| D | 3 | 75.0% |
| C | 1 | 25.0% |

##### *Space Separator*

| Value | Count | Frequency (%) |
| --- | --- | --- |
|  | 2 | 100.0% |

#### Most occurring scripts

| Value | Count | Frequency (%) |
| --- | --- | --- |
| Latin | 34 | 94.4% |
| Common | 2 | 5.6% |

#### Most frequent character per script

##### *Latin*

| Value | Count | Frequency (%) |
| --- | --- | --- |
| e | 7 | 20.6% |
| a | 5 | 14.7% |
| D | 3 | 8.8% |
| s | 3 | 8.8% |
| d | 3 | 8.8% |
| o | 3 | 8.8% |
| m | 2 | 5.9% |
| p | 2 | 5.9% |
| r | 2 | 5.9% |
| g | 2 | 5.9% |
| Other values (2) | 2 | 5.9% |

##### *Common*

| Value | Count | Frequency (%) |
| --- | --- | --- |
|  | 2 | 100.0% |

#### Most occurring blocks

| Value | Count | Frequency (%) |
| --- | --- | --- |
| ASCII | 36 | 100.0% |

#### Most frequent character per block

##### *ASCII*

| Value | Count | Frequency (%) |
| --- | --- | --- |
| e | 7 | 19.4% |
| a | 5 | 13.9% |
| D | 3 | 8.3% |
| s | 3 | 8.3% |
| d | 3 | 8.3% |
| o | 3 | 8.3% |
| m | 2 | 5.6% |
| p | 2 | 5.6% |
| r | 2 | 5.6% |
| g | 2 | 5.6% |
| Other values (3) | 4 | 11.1% |

sitAtual  
Categorical

|  |  |
| --- | --- |
| Distinct | 2 |
| Distinct (%) | 66.7% |
| Missing | 0 |
| Missing (%) | 0.0% |
| Memory size | 48.0 B |

|  |  |
| --- | --- |
| Cura | 2 |
| Abandono | 1 |

More details

- Overview
- Categories
- Words
- Characters

Length

|  |  |
| --- | --- |
| Max length | 8 |
| Median length | 4 |
| Mean length | 5.3333333 |
| Min length | 4 |

Characters and Unicode

|  |  |
| --- | --- |
| Total characters | 16 |
| Distinct characters | 9 |
| Distinct categories | 2 ? |
| Distinct scripts | 1 ? |
| Distinct blocks | 1 ? |

The Unicode Standard assigns character properties to each code point, which can be used to analyse textual variables.

Unique

|  |  |
| --- | --- |
| Unique | 1 ? |
| Unique (%) | 33.3% |

Sample

|  |  |
| --- | --- |
| 1st row | Abandono |
| 2nd row | Cura |
| 3rd row | Cura |

#### Common Values

| Value | Count | Frequency (%) |
| --- | --- | --- |
| Cura | 2 | 66.7% |
| Abandono | 1 | 33.3% |

#### Length

xml version="1.0" encoding="utf-8" standalone="no"?2023-10-31T16:30:04.951653image/svg+xmlMatplotlib v3.6.0, https://matplotlib.org/ 

Histogram of lengths of the category

#### Common Values (Plot)

xml version="1.0" encoding="utf-8" standalone="no"?2023-10-31T16:30:05.102072image/svg+xmlMatplotlib v3.6.0, https://matplotlib.org/

| Value | Count | Frequency (%) |
| --- | --- | --- |
| cura | 2 | 66.7% |
| abandono | 1 | 33.3% |

- Characters
- Categories
- Scripts
- Blocks

#### Most occurring characters

| Value | Count | Frequency (%) |
| --- | --- | --- |
| a | 3 | 18.8% |
| C | 2 | 12.5% |
| u | 2 | 12.5% |
| r | 2 | 12.5% |
| n | 2 | 12.5% |
| o | 2 | 12.5% |
| A | 1 | 6.2% |
| b | 1 | 6.2% |
| d | 1 | 6.2% |

#### Most occurring categories

| Value | Count | Frequency (%) |
| --- | --- | --- |
| Lowercase Letter | 13 | 81.2% |
| Uppercase Letter | 3 | 18.8% |

#### Most frequent character per category

##### *Lowercase Letter*

| Value | Count | Frequency (%) |
| --- | --- | --- |
| a | 3 | 23.1% |
| u | 2 | 15.4% |
| r | 2 | 15.4% |
| n | 2 | 15.4% |
| o | 2 | 15.4% |
| b | 1 | 7.7% |
| d | 1 | 7.7% |

##### *Uppercase Letter*

| Value | Count | Frequency (%) |
| --- | --- | --- |
| C | 2 | 66.7% |
| A | 1 | 33.3% |

#### Most occurring scripts

| Value | Count | Frequency (%) |
| --- | --- | --- |
| Latin | 16 | 100.0% |

#### Most frequent character per script

##### *Latin*

| Value | Count | Frequency (%) |
| --- | --- | --- |
| a | 3 | 18.8% |
| C | 2 | 12.5% |
| u | 2 | 12.5% |
| r | 2 | 12.5% |
| n | 2 | 12.5% |
| o | 2 | 12.5% |
| A | 1 | 6.2% |
| b | 1 | 6.2% |
| d | 1 | 6.2% |

#### Most occurring blocks

| Value | Count | Frequency (%) |
| --- | --- | --- |
| ASCII | 16 | 100.0% |

#### Most frequent character per block

##### *ASCII*

| Value | Count | Frequency (%) |
| --- | --- | --- |
| a | 3 | 18.8% |
| C | 2 | 12.5% |
| u | 2 | 12.5% |
| r | 2 | 12.5% |
| n | 2 | 12.5% |
| o | 2 | 12.5% |
| A | 1 | 6.2% |
| b | 1 | 6.2% |
| d | 1 | 6.2% |

tipoCaso  
Categorical

|  |  |
| --- | --- |
| Distinct | 1 |
| Distinct (%) | 33.3% |
| Missing | 0 |
| Missing (%) | 0.0% |
| Memory size | 48.0 B |

|  |  |
| --- | --- |
| Novo | 3 |

More details

- Overview
- Categories
- Words
- Characters

Length

|  |  |
| --- | --- |
| Max length | 4 |
| Median length | 4 |
| Mean length | 4 |
| Min length | 4 |

Characters and Unicode

|  |  |
| --- | --- |
| Total characters | 12 |
| Distinct characters | 3 |
| Distinct categories | 2 ? |
| Distinct scripts | 1 ? |
| Distinct blocks | 1 ? |

The Unicode Standard assigns character properties to each code point, which can be used to analyse textual variables.

Unique

|  |  |
| --- | --- |
| Unique | 0 ? |
| Unique (%) | 0.0% |

Sample

|  |  |
| --- | --- |
| 1st row | Novo |
| 2nd row | Novo |
| 3rd row | Novo |

#### Common Values

| Value | Count | Frequency (%) |
| --- | --- | --- |
| Novo | 3 | 100.0% |

#### Length

xml version="1.0" encoding="utf-8" standalone="no"?2023-10-31T16:30:05.219629image/svg+xmlMatplotlib v3.6.0, https://matplotlib.org/ 

Histogram of lengths of the category

#### Common Values (Plot)

xml version="1.0" encoding="utf-8" standalone="no"?2023-10-31T16:30:05.352726image/svg+xmlMatplotlib v3.6.0, https://matplotlib.org/

| Value | Count | Frequency (%) |
| --- | --- | --- |
| novo | 3 | 100.0% |

- Characters
- Categories
- Scripts
- Blocks

#### Most occurring characters

| Value | Count | Frequency (%) |
| --- | --- | --- |
| o | 6 | 50.0% |
| N | 3 | 25.0% |
| v | 3 | 25.0% |

#### Most occurring categories

| Value | Count | Frequency (%) |
| --- | --- | --- |
| Lowercase Letter | 9 | 75.0% |
| Uppercase Letter | 3 | 25.0% |

#### Most frequent character per category

##### *Lowercase Letter*

| Value | Count | Frequency (%) |
| --- | --- | --- |
| o | 6 | 66.7% |
| v | 3 | 33.3% |

##### *Uppercase Letter*

| Value | Count | Frequency (%) |
| --- | --- | --- |
| N | 3 | 100.0% |

#### Most occurring scripts

| Value | Count | Frequency (%) |
| --- | --- | --- |
| Latin | 12 | 100.0% |

#### Most frequent character per script

##### *Latin*

| Value | Count | Frequency (%) |
| --- | --- | --- |
| o | 6 | 50.0% |
| N | 3 | 25.0% |
| v | 3 | 25.0% |

#### Most occurring blocks

| Value | Count | Frequency (%) |
| --- | --- | --- |
| ASCII | 12 | 100.0% |

#### Most frequent character per block

##### *ASCII*

| Value | Count | Frequency (%) |
| --- | --- | --- |
| o | 6 | 50.0% |
| N | 3 | 25.0% |
| v | 3 | 25.0% |

FORMACLIN1  
Categorical

|  |  |
| --- | --- |
| Distinct | 2 |
| Distinct (%) | 66.7% |
| Missing | 0 |
| Missing (%) | 0.0% |
| Memory size | 48.0 B |

|  |  |
| --- | --- |
| Pul | 2 |
| Pleural | 1 |

More details

- Overview
- Categories
- Words
- Characters

Length

|  |  |
| --- | --- |
| Max length | 7 |
| Median length | 3 |
| Mean length | 4.3333333 |
| Min length | 3 |

Characters and Unicode

|  |  |
| --- | --- |
| Total characters | 13 |
| Distinct characters | 6 |
| Distinct categories | 2 ? |
| Distinct scripts | 1 ? |
| Distinct blocks | 1 ? |

The Unicode Standard assigns character properties to each code point, which can be used to analyse textual variables.

Unique

|  |  |
| --- | --- |
| Unique | 1 ? |
| Unique (%) | 33.3% |

Sample

|  |  |
| --- | --- |
| 1st row | Pul |
| 2nd row | Pleural |
| 3rd row | Pul |

#### Common Values

| Value | Count | Frequency (%) |
| --- | --- | --- |
| Pul | 2 | 66.7% |
| Pleural | 1 | 33.3% |

#### Length

xml version="1.0" encoding="utf-8" standalone="no"?2023-10-31T16:30:05.469513image/svg+xmlMatplotlib v3.6.0, https://matplotlib.org/ 

Histogram of lengths of the category

#### Common Values (Plot)

xml version="1.0" encoding="utf-8" standalone="no"?2023-10-31T16:30:05.620438image/svg+xmlMatplotlib v3.6.0, https://matplotlib.org/

| Value | Count | Frequency (%) |
| --- | --- | --- |
| pul | 2 | 66.7% |
| pleural | 1 | 33.3% |

- Characters
- Categories
- Scripts
- Blocks

#### Most occurring characters

| Value | Count | Frequency (%) |
| --- | --- | --- |
| l | 4 | 30.8% |
| P | 3 | 23.1% |
| u | 3 | 23.1% |
| e | 1 | 7.7% |
| r | 1 | 7.7% |
| a | 1 | 7.7% |

#### Most occurring categories

| Value | Count | Frequency (%) |
| --- | --- | --- |
| Lowercase Letter | 10 | 76.9% |
| Uppercase Letter | 3 | 23.1% |

#### Most frequent character per category

##### *Lowercase Letter*

| Value | Count | Frequency (%) |
| --- | --- | --- |
| l | 4 | 40.0% |
| u | 3 | 30.0% |
| e | 1 | 10.0% |
| r | 1 | 10.0% |
| a | 1 | 10.0% |

##### *Uppercase Letter*

| Value | Count | Frequency (%) |
| --- | --- | --- |
| P | 3 | 100.0% |

#### Most occurring scripts

| Value | Count | Frequency (%) |
| --- | --- | --- |
| Latin | 13 | 100.0% |

#### Most frequent character per script

##### *Latin*

| Value | Count | Frequency (%) |
| --- | --- | --- |
| l | 4 | 30.8% |
| P | 3 | 23.1% |
| u | 3 | 23.1% |
| e | 1 | 7.7% |
| r | 1 | 7.7% |
| a | 1 | 7.7% |

#### Most occurring blocks

| Value | Count | Frequency (%) |
| --- | --- | --- |
| ASCII | 13 | 100.0% |

#### Most frequent character per block

##### *ASCII*

| Value | Count | Frequency (%) |
| --- | --- | --- |
| l | 4 | 30.8% |
| P | 3 | 23.1% |
| u | 3 | 23.1% |
| e | 1 | 7.7% |
| r | 1 | 7.7% |
| a | 1 | 7.7% |

classif  
Categorical

`HIGH CORRELATION`  `UNIFORM`  `UNIQUE`

|  |  |
| --- | --- |
| Distinct | 3 |
| Distinct (%) | 100.0% |
| Missing | 0 |
| Missing (%) | 0.0% |
| Memory size | 48.0 B |

|  |  |
| --- | --- |
| P+E | 1 |
| Ext | 1 |
| Pul | 1 |

More details

- Overview
- Categories
- Words
- Characters

Length

|  |  |
| --- | --- |
| Max length | 3 |
| Median length | 3 |
| Mean length | 3 |
| Min length | 3 |

Characters and Unicode

|  |  |
| --- | --- |
| Total characters | 9 |
| Distinct characters | 7 |
| Distinct categories | 3 ? |
| Distinct scripts | 2 ? |
| Distinct blocks | 1 ? |

The Unicode Standard assigns character properties to each code point, which can be used to analyse textual variables.

Unique

|  |  |
| --- | --- |
| Unique | 3 ? |
| Unique (%) | 100.0% |

Sample

|  |  |
| --- | --- |
| 1st row | P+E |
| 2nd row | Ext |
| 3rd row | Pul |

#### Common Values

| Value | Count | Frequency (%) |
| --- | --- | --- |
| P+E | 1 | 33.3% |
| Ext | 1 | 33.3% |
| Pul | 1 | 33.3% |

#### Length

xml version="1.0" encoding="utf-8" standalone="no"?2023-10-31T16:30:05.734921image/svg+xmlMatplotlib v3.6.0, https://matplotlib.org/ 

Histogram of lengths of the category

#### Common Values (Plot)

xml version="1.0" encoding="utf-8" standalone="no"?2023-10-31T16:30:05.879287image/svg+xmlMatplotlib v3.6.0, https://matplotlib.org/

| Value | Count | Frequency (%) |
| --- | --- | --- |
| p+e | 1 | 33.3% |
| ext | 1 | 33.3% |
| pul | 1 | 33.3% |

- Characters
- Categories
- Scripts
- Blocks

#### Most occurring characters

| Value | Count | Frequency (%) |
| --- | --- | --- |
| P | 2 | 22.2% |
| E | 2 | 22.2% |
| + | 1 | 11.1% |
| x | 1 | 11.1% |
| t | 1 | 11.1% |
| u | 1 | 11.1% |
| l | 1 | 11.1% |

#### Most occurring categories

| Value | Count | Frequency (%) |
| --- | --- | --- |
| Uppercase Letter | 4 | 44.4% |
| Lowercase Letter | 4 | 44.4% |
| Math Symbol | 1 | 11.1% |

#### Most frequent character per category

##### *Lowercase Letter*

| Value | Count | Frequency (%) |
| --- | --- | --- |
| x | 1 | 25.0% |
| t | 1 | 25.0% |
| u | 1 | 25.0% |
| l | 1 | 25.0% |

##### *Uppercase Letter*

| Value | Count | Frequency (%) |
| --- | --- | --- |
| P | 2 | 50.0% |
| E | 2 | 50.0% |

##### *Math Symbol*

| Value | Count | Frequency (%) |
| --- | --- | --- |
| + | 1 | 100.0% |

#### Most occurring scripts

| Value | Count | Frequency (%) |
| --- | --- | --- |
| Latin | 8 | 88.9% |
| Common | 1 | 11.1% |

#### Most frequent character per script

##### *Latin*

| Value | Count | Frequency (%) |
| --- | --- | --- |
| P | 2 | 25.0% |
| E | 2 | 25.0% |
| x | 1 | 12.5% |
| t | 1 | 12.5% |
| u | 1 | 12.5% |
| l | 1 | 12.5% |

##### *Common*

| Value | Count | Frequency (%) |
| --- | --- | --- |
| + | 1 | 100.0% |

#### Most occurring blocks

| Value | Count | Frequency (%) |
| --- | --- | --- |
| ASCII | 9 | 100.0% |

#### Most frequent character per block

##### *ASCII*

| Value | Count | Frequency (%) |
| --- | --- | --- |
| P | 2 | 22.2% |
| E | 2 | 22.2% |
| + | 1 | 11.1% |
| x | 1 | 11.1% |
| t | 1 | 11.1% |
| u | 1 | 11.1% |
| l | 1 | 11.1% |

descoberta  
Categorical

|  |  |
| --- | --- |
| Distinct | 2 |
| Distinct (%) | 66.7% |
| Missing | 0 |
| Missing (%) | 0.0% |
| Memory size | 48.0 B |

|  |  |
| --- | --- |
| Elucidacao Diagn. em Internacao | 2 |
| Urgencia / Emergencia | 1 |

More details

- Overview
- Categories
- Words
- Characters

Length

|  |  |
| --- | --- |
| Max length | 31 |
| Median length | 31 |
| Mean length | 27.666667 |
| Min length | 21 |

Characters and Unicode

|  |  |
| --- | --- |
| Total characters | 83 |
| Distinct characters | 20 |
| Distinct categories | 4 ? |
| Distinct scripts | 2 ? |
| Distinct blocks | 1 ? |

The Unicode Standard assigns character properties to each code point, which can be used to analyse textual variables.

Unique

|  |  |
| --- | --- |
| Unique | 1 ? |
| Unique (%) | 33.3% |

Sample

|  |  |
| --- | --- |
| 1st row | Urgencia / Emergencia |
| 2nd row | Elucidacao Diagn. em Internacao |
| 3rd row | Elucidacao Diagn. em Internacao |

#### Common Values

| Value | Count | Frequency (%) |
| --- | --- | --- |
| Elucidacao Diagn. em Internacao | 2 | 66.7% |
| Urgencia / Emergencia | 1 | 33.3% |

#### Length

xml version="1.0" encoding="utf-8" standalone="no"?2023-10-31T16:30:05.997752image/svg+xmlMatplotlib v3.6.0, https://matplotlib.org/ 

Histogram of lengths of the category

#### Common Values (Plot)

xml version="1.0" encoding="utf-8" standalone="no"?2023-10-31T16:30:06.130798image/svg+xmlMatplotlib v3.6.0, https://matplotlib.org/

| Value | Count | Frequency (%) |
| --- | --- | --- |
| elucidacao | 2 | 18.2% |
| diagn | 2 | 18.2% |
| em | 2 | 18.2% |
| internacao | 2 | 18.2% |
| urgencia | 1 | 9.1% |
|  | 1 | 9.1% |
| emergencia | 1 | 9.1% |

- Characters
- Categories
- Scripts
- Blocks

#### Most occurring characters

| Value | Count | Frequency (%) |
| --- | --- | --- |
| a | 12 | 14.5% |
| n | 8 | 9.6% |
| c | 8 | 9.6% |
|  | 8 | 9.6% |
| e | 7 | 8.4% |
| i | 6 | 7.2% |
| g | 4 | 4.8% |
| r | 4 | 4.8% |
| o | 4 | 4.8% |
| m | 3 | 3.6% |
| Other values (10) | 19 | 22.9% |

#### Most occurring categories

| Value | Count | Frequency (%) |
| --- | --- | --- |
| Lowercase Letter | 64 | 77.1% |
| Space Separator | 8 | 9.6% |
| Uppercase Letter | 8 | 9.6% |
| Other Punctuation | 3 | 3.6% |

#### Most frequent character per category

##### *Lowercase Letter*

| Value | Count | Frequency (%) |
| --- | --- | --- |
| a | 12 | 18.8% |
| n | 8 | 12.5% |
| c | 8 | 12.5% |
| e | 7 | 10.9% |
| i | 6 | 9.4% |
| g | 4 | 6.2% |
| r | 4 | 6.2% |
| o | 4 | 6.2% |
| m | 3 | 4.7% |
| l | 2 | 3.1% |
| Other values (3) | 6 | 9.4% |

##### *Uppercase Letter*

| Value | Count | Frequency (%) |
| --- | --- | --- |
| E | 3 | 37.5% |
| D | 2 | 25.0% |
| I | 2 | 25.0% |
| U | 1 | 12.5% |

##### *Other Punctuation*

| Value | Count | Frequency (%) |
| --- | --- | --- |
| . | 2 | 66.7% |
| / | 1 | 33.3% |

##### *Space Separator*

| Value | Count | Frequency (%) |
| --- | --- | --- |
|  | 8 | 100.0% |

#### Most occurring scripts

| Value | Count | Frequency (%) |
| --- | --- | --- |
| Latin | 72 | 86.7% |
| Common | 11 | 13.3% |

#### Most frequent character per script

##### *Latin*

| Value | Count | Frequency (%) |
| --- | --- | --- |
| a | 12 | 16.7% |
| n | 8 | 11.1% |
| c | 8 | 11.1% |
| e | 7 | 9.7% |
| i | 6 | 8.3% |
| g | 4 | 5.6% |
| r | 4 | 5.6% |
| o | 4 | 5.6% |
| m | 3 | 4.2% |
| E | 3 | 4.2% |
| Other values (7) | 13 | 18.1% |

##### *Common*

| Value | Count | Frequency (%) |
| --- | --- | --- |
|  | 8 | 72.7% |
| . | 2 | 18.2% |
| / | 1 | 9.1% |

#### Most occurring blocks

| Value | Count | Frequency (%) |
| --- | --- | --- |
| ASCII | 83 | 100.0% |

#### Most frequent character per block

##### *ASCII*

| Value | Count | Frequency (%) |
| --- | --- | --- |
| a | 12 | 14.5% |
| n | 8 | 9.6% |
| c | 8 | 9.6% |
|  | 8 | 9.6% |
| e | 7 | 8.4% |
| i | 6 | 7.2% |
| g | 4 | 4.8% |
| r | 4 | 4.8% |
| o | 4 | 4.8% |
| m | 3 | 3.6% |
| Other values (10) | 19 | 22.9% |

bac  
Categorical

`HIGH CORRELATION`  `UNIFORM`  `UNIQUE`

|  |  |
| --- | --- |
| Distinct | 3 |
| Distinct (%) | 100.0% |
| Missing | 0 |
| Missing (%) | 0.0% |
| Memory size | 48.0 B |

|  |  |
| --- | --- |
| N/realiz | 1 |
| Neg | 1 |
| Pos | 1 |

More details

- Overview
- Categories
- Words
- Characters

Length

|  |  |
| --- | --- |
| Max length | 8 |
| Median length | 3 |
| Mean length | 4.6666667 |
| Min length | 3 |

Characters and Unicode

|  |  |
| --- | --- |
| Total characters | 14 |
| Distinct characters | 12 |
| Distinct categories | 3 ? |
| Distinct scripts | 2 ? |
| Distinct blocks | 1 ? |

The Unicode Standard assigns character properties to each code point, which can be used to analyse textual variables.

Unique

|  |  |
| --- | --- |
| Unique | 3 ? |
| Unique (%) | 100.0% |

Sample

|  |  |
| --- | --- |
| 1st row | N/realiz |
| 2nd row | Neg |
| 3rd row | Pos |

#### Common Values

| Value | Count | Frequency (%) |
| --- | --- | --- |
| N/realiz | 1 | 33.3% |
| Neg | 1 | 33.3% |
| Pos | 1 | 33.3% |

#### Length

xml version="1.0" encoding="utf-8" standalone="no"?2023-10-31T16:30:06.248893image/svg+xmlMatplotlib v3.6.0, https://matplotlib.org/ 

Histogram of lengths of the category

#### Common Values (Plot)

xml version="1.0" encoding="utf-8" standalone="no"?2023-10-31T16:30:06.393365image/svg+xmlMatplotlib v3.6.0, https://matplotlib.org/

| Value | Count | Frequency (%) |
| --- | --- | --- |
| n/realiz | 1 | 33.3% |
| neg | 1 | 33.3% |
| pos | 1 | 33.3% |

- Characters
- Categories
- Scripts
- Blocks

#### Most occurring characters

| Value | Count | Frequency (%) |
| --- | --- | --- |
| N | 2 | 14.3% |
| e | 2 | 14.3% |
| / | 1 | 7.1% |
| r | 1 | 7.1% |
| a | 1 | 7.1% |
| l | 1 | 7.1% |
| i | 1 | 7.1% |
| z | 1 | 7.1% |
| g | 1 | 7.1% |
| P | 1 | 7.1% |
| Other values (2) | 2 | 14.3% |

#### Most occurring categories

| Value | Count | Frequency (%) |
| --- | --- | --- |
| Lowercase Letter | 10 | 71.4% |
| Uppercase Letter | 3 | 21.4% |
| Other Punctuation | 1 | 7.1% |

#### Most frequent character per category

##### *Lowercase Letter*

| Value | Count | Frequency (%) |
| --- | --- | --- |
| e | 2 | 20.0% |
| r | 1 | 10.0% |
| a | 1 | 10.0% |
| l | 1 | 10.0% |
| i | 1 | 10.0% |
| z | 1 | 10.0% |
| g | 1 | 10.0% |
| o | 1 | 10.0% |
| s | 1 | 10.0% |

##### *Uppercase Letter*

| Value | Count | Frequency (%) |
| --- | --- | --- |
| N | 2 | 66.7% |
| P | 1 | 33.3% |

##### *Other Punctuation*

| Value | Count | Frequency (%) |
| --- | --- | --- |
| / | 1 | 100.0% |

#### Most occurring scripts

| Value | Count | Frequency (%) |
| --- | --- | --- |
| Latin | 13 | 92.9% |
| Common | 1 | 7.1% |

#### Most frequent character per script

##### *Latin*

| Value | Count | Frequency (%) |
| --- | --- | --- |
| N | 2 | 15.4% |
| e | 2 | 15.4% |
| r | 1 | 7.7% |
| a | 1 | 7.7% |
| l | 1 | 7.7% |
| i | 1 | 7.7% |
| z | 1 | 7.7% |
| g | 1 | 7.7% |
| P | 1 | 7.7% |
| o | 1 | 7.7% |

##### *Common*

| Value | Count | Frequency (%) |
| --- | --- | --- |
| / | 1 | 100.0% |

#### Most occurring blocks

| Value | Count | Frequency (%) |
| --- | --- | --- |
| ASCII | 14 | 100.0% |

#### Most frequent character per block

##### *ASCII*

| Value | Count | Frequency (%) |
| --- | --- | --- |
| N | 2 | 14.3% |
| e | 2 | 14.3% |
| / | 1 | 7.1% |
| r | 1 | 7.1% |
| a | 1 | 7.1% |
| l | 1 | 7.1% |
| i | 1 | 7.1% |
| z | 1 | 7.1% |
| g | 1 | 7.1% |
| P | 1 | 7.1% |
| Other values (2) | 2 | 14.3% |

BACOUTRO  
Categorical

|  |  |
| --- | --- |
| Distinct | 2 |
| Distinct (%) | 66.7% |
| Missing | 0 |
| Missing (%) | 0.0% |
| Memory size | 48.0 B |

|  |  |
| --- | --- |
| N/realiz | 2 |
| Pos | 1 |

More details

- Overview
- Categories
- Words
- Characters

Length

|  |  |
| --- | --- |
| Max length | 8 |
| Median length | 8 |
| Mean length | 6.3333333 |
| Min length | 3 |

Characters and Unicode

|  |  |
| --- | --- |
| Total characters | 19 |
| Distinct characters | 11 |
| Distinct categories | 3 ? |
| Distinct scripts | 2 ? |
| Distinct blocks | 1 ? |

The Unicode Standard assigns character properties to each code point, which can be used to analyse textual variables.

Unique

|  |  |
| --- | --- |
| Unique | 1 ? |
| Unique (%) | 33.3% |

Sample

|  |  |
| --- | --- |
| 1st row | Pos |
| 2nd row | N/realiz |
| 3rd row | N/realiz |

#### Common Values

| Value | Count | Frequency (%) |
| --- | --- | --- |
| N/realiz | 2 | 66.7% |
| Pos | 1 | 33.3% |

#### Length

xml version="1.0" encoding="utf-8" standalone="no"?2023-10-31T16:30:06.513191image/svg+xmlMatplotlib v3.6.0, https://matplotlib.org/ 

Histogram of lengths of the category

#### Common Values (Plot)

xml version="1.0" encoding="utf-8" standalone="no"?2023-10-31T16:30:06.651648image/svg+xmlMatplotlib v3.6.0, https://matplotlib.org/

| Value | Count | Frequency (%) |
| --- | --- | --- |
| n/realiz | 2 | 66.7% |
| pos | 1 | 33.3% |

- Characters
- Categories
- Scripts
- Blocks

#### Most occurring characters

| Value | Count | Frequency (%) |
| --- | --- | --- |
| N | 2 | 10.5% |
| / | 2 | 10.5% |
| r | 2 | 10.5% |
| e | 2 | 10.5% |
| a | 2 | 10.5% |
| l | 2 | 10.5% |
| i | 2 | 10.5% |
| z | 2 | 10.5% |
| P | 1 | 5.3% |
| o | 1 | 5.3% |

#### Most occurring categories

| Value | Count | Frequency (%) |
| --- | --- | --- |
| Lowercase Letter | 14 | 73.7% |
| Uppercase Letter | 3 | 15.8% |
| Other Punctuation | 2 | 10.5% |

#### Most frequent character per category

##### *Lowercase Letter*

| Value | Count | Frequency (%) |
| --- | --- | --- |
| r | 2 | 14.3% |
| e | 2 | 14.3% |
| a | 2 | 14.3% |
| l | 2 | 14.3% |
| i | 2 | 14.3% |
| z | 2 | 14.3% |
| o | 1 | 7.1% |
| s | 1 | 7.1% |

##### *Uppercase Letter*

| Value | Count | Frequency (%) |
| --- | --- | --- |
| N | 2 | 66.7% |
| P | 1 | 33.3% |

##### *Other Punctuation*

| Value | Count | Frequency (%) |
| --- | --- | --- |
| / | 2 | 100.0% |

#### Most occurring scripts

| Value | Count | Frequency (%) |
| --- | --- | --- |
| Latin | 17 | 89.5% |
| Common | 2 | 10.5% |

#### Most frequent character per script

##### *Latin*

| Value | Count | Frequency (%) |
| --- | --- | --- |
| N | 2 | 11.8% |
| r | 2 | 11.8% |
| e | 2 | 11.8% |
| a | 2 | 11.8% |
| l | 2 | 11.8% |
| i | 2 | 11.8% |
| z | 2 | 11.8% |
| P | 1 | 5.9% |
| o | 1 | 5.9% |
| s | 1 | 5.9% |

##### *Common*

| Value | Count | Frequency (%) |
| --- | --- | --- |
| / | 2 | 100.0% |

#### Most occurring blocks

| Value | Count | Frequency (%) |
| --- | --- | --- |
| ASCII | 19 | 100.0% |

#### Most frequent character per block

##### *ASCII*

| Value | Count | Frequency (%) |
| --- | --- | --- |
| N | 2 | 10.5% |
| / | 2 | 10.5% |
| r | 2 | 10.5% |
| e | 2 | 10.5% |
| a | 2 | 10.5% |
| l | 2 | 10.5% |
| i | 2 | 10.5% |
| z | 2 | 10.5% |
| P | 1 | 5.3% |
| o | 1 | 5.3% |

cultEsc  
Categorical

|  |  |
| --- | --- |
| Distinct | 1 |
| Distinct (%) | 33.3% |
| Missing | 0 |
| Missing (%) | 0.0% |
| Memory size | 48.0 B |

|  |  |
| --- | --- |
| N/realiz | 3 |

More details

- Overview
- Categories
- Words
- Characters

Length

|  |  |
| --- | --- |
| Max length | 8 |
| Median length | 8 |
| Mean length | 8 |
| Min length | 8 |

Characters and Unicode

|  |  |
| --- | --- |
| Total characters | 24 |
| Distinct characters | 8 |
| Distinct categories | 3 ? |
| Distinct scripts | 2 ? |
| Distinct blocks | 1 ? |

The Unicode Standard assigns character properties to each code point, which can be used to analyse textual variables.

Unique

|  |  |
| --- | --- |
| Unique | 0 ? |
| Unique (%) | 0.0% |

Sample

|  |  |
| --- | --- |
| 1st row | N/realiz |
| 2nd row | N/realiz |
| 3rd row | N/realiz |

#### Common Values

| Value | Count | Frequency (%) |
| --- | --- | --- |
| N/realiz | 3 | 100.0% |

#### Length

xml version="1.0" encoding="utf-8" standalone="no"?2023-10-31T16:30:06.768332image/svg+xmlMatplotlib v3.6.0, https://matplotlib.org/ 

Histogram of lengths of the category

#### Common Values (Plot)

xml version="1.0" encoding="utf-8" standalone="no"?2023-10-31T16:30:06.900252image/svg+xmlMatplotlib v3.6.0, https://matplotlib.org/

| Value | Count | Frequency (%) |
| --- | --- | --- |
| n/realiz | 3 | 100.0% |

- Characters
- Categories
- Scripts
- Blocks

#### Most occurring characters

| Value | Count | Frequency (%) |
| --- | --- | --- |
| N | 3 | 12.5% |
| / | 3 | 12.5% |
| r | 3 | 12.5% |
| e | 3 | 12.5% |
| a | 3 | 12.5% |
| l | 3 | 12.5% |
| i | 3 | 12.5% |
| z | 3 | 12.5% |

#### Most occurring categories

| Value | Count | Frequency (%) |
| --- | --- | --- |
| Lowercase Letter | 18 | 75.0% |
| Uppercase Letter | 3 | 12.5% |
| Other Punctuation | 3 | 12.5% |

#### Most frequent character per category

##### *Lowercase Letter*

| Value | Count | Frequency (%) |
| --- | --- | --- |
| r | 3 | 16.7% |
| e | 3 | 16.7% |
| a | 3 | 16.7% |
| l | 3 | 16.7% |
| i | 3 | 16.7% |
| z | 3 | 16.7% |

##### *Uppercase Letter*

| Value | Count | Frequency (%) |
| --- | --- | --- |
| N | 3 | 100.0% |

##### *Other Punctuation*

| Value | Count | Frequency (%) |
| --- | --- | --- |
| / | 3 | 100.0% |

#### Most occurring scripts

| Value | Count | Frequency (%) |
| --- | --- | --- |
| Latin | 21 | 87.5% |
| Common | 3 | 12.5% |

#### Most frequent character per script

##### *Latin*

| Value | Count | Frequency (%) |
| --- | --- | --- |
| N | 3 | 14.3% |
| r | 3 | 14.3% |
| e | 3 | 14.3% |
| a | 3 | 14.3% |
| l | 3 | 14.3% |
| i | 3 | 14.3% |
| z | 3 | 14.3% |

##### *Common*

| Value | Count | Frequency (%) |
| --- | --- | --- |
| / | 3 | 100.0% |

#### Most occurring blocks

| Value | Count | Frequency (%) |
| --- | --- | --- |
| ASCII | 24 | 100.0% |

#### Most frequent character per block

##### *ASCII*

| Value | Count | Frequency (%) |
| --- | --- | --- |
| N | 3 | 12.5% |
| / | 3 | 12.5% |
| r | 3 | 12.5% |
| e | 3 | 12.5% |
| a | 3 | 12.5% |
| l | 3 | 12.5% |
| i | 3 | 12.5% |
| z | 3 | 12.5% |

RX  
Categorical

|  |  |
| --- | --- |
| Distinct | 2 |
| Distinct (%) | 66.7% |
| Missing | 0 |
| Missing (%) | 0.0% |
| Memory size | 48.0 B |

|  |  |
| --- | --- |
| Susp TB | 2 |
| Susp c/cavid | 1 |

More details

- Overview
- Categories
- Words
- Characters

Length

|  |  |
| --- | --- |
| Max length | 12 |
| Median length | 7 |
| Mean length | 8.6666667 |
| Min length | 7 |

Characters and Unicode

|  |  |
| --- | --- |
| Total characters | 26 |
| Distinct characters | 13 |
| Distinct categories | 4 ? |
| Distinct scripts | 2 ? |
| Distinct blocks | 1 ? |

The Unicode Standard assigns character properties to each code point, which can be used to analyse textual variables.

Unique

|  |  |
| --- | --- |
| Unique | 1 ? |
| Unique (%) | 33.3% |

Sample

|  |  |
| --- | --- |
| 1st row | Susp TB |
| 2nd row | Susp TB |
| 3rd row | Susp c/cavid |

#### Common Values

| Value | Count | Frequency (%) |
| --- | --- | --- |
| Susp TB | 2 | 66.7% |
| Susp c/cavid | 1 | 33.3% |

#### Length

xml version="1.0" encoding="utf-8" standalone="no"?2023-10-31T16:30:07.004063image/svg+xmlMatplotlib v3.6.0, https://matplotlib.org/ 

Histogram of lengths of the category

#### Common Values (Plot)

xml version="1.0" encoding="utf-8" standalone="no"?2023-10-31T16:30:07.143193image/svg+xmlMatplotlib v3.6.0, https://matplotlib.org/

| Value | Count | Frequency (%) |
| --- | --- | --- |
| susp | 3 | 50.0% |
| tb | 2 | 33.3% |
| c/cavid | 1 | 16.7% |

- Characters
- Categories
- Scripts
- Blocks

#### Most occurring characters

| Value | Count | Frequency (%) |
| --- | --- | --- |
| S | 3 | 11.5% |
| u | 3 | 11.5% |
| s | 3 | 11.5% |
| p | 3 | 11.5% |
|  | 3 | 11.5% |
| T | 2 | 7.7% |
| B | 2 | 7.7% |
| c | 2 | 7.7% |
| / | 1 | 3.8% |
| a | 1 | 3.8% |
| Other values (3) | 3 | 11.5% |

#### Most occurring categories

| Value | Count | Frequency (%) |
| --- | --- | --- |
| Lowercase Letter | 15 | 57.7% |
| Uppercase Letter | 7 | 26.9% |
| Space Separator | 3 | 11.5% |
| Other Punctuation | 1 | 3.8% |

#### Most frequent character per category

##### *Lowercase Letter*

| Value | Count | Frequency (%) |
| --- | --- | --- |
| u | 3 | 20.0% |
| s | 3 | 20.0% |
| p | 3 | 20.0% |
| c | 2 | 13.3% |
| a | 1 | 6.7% |
| v | 1 | 6.7% |
| i | 1 | 6.7% |
| d | 1 | 6.7% |

##### *Uppercase Letter*

| Value | Count | Frequency (%) |
| --- | --- | --- |
| S | 3 | 42.9% |
| T | 2 | 28.6% |
| B | 2 | 28.6% |

##### *Space Separator*

| Value | Count | Frequency (%) |
| --- | --- | --- |
|  | 3 | 100.0% |

##### *Other Punctuation*

| Value | Count | Frequency (%) |
| --- | --- | --- |
| / | 1 | 100.0% |

#### Most occurring scripts

| Value | Count | Frequency (%) |
| --- | --- | --- |
| Latin | 22 | 84.6% |
| Common | 4 | 15.4% |

#### Most frequent character per script

##### *Latin*

| Value | Count | Frequency (%) |
| --- | --- | --- |
| S | 3 | 13.6% |
| u | 3 | 13.6% |
| s | 3 | 13.6% |
| p | 3 | 13.6% |
| T | 2 | 9.1% |
| B | 2 | 9.1% |
| c | 2 | 9.1% |
| a | 1 | 4.5% |
| v | 1 | 4.5% |
| i | 1 | 4.5% |

##### *Common*

| Value | Count | Frequency (%) |
| --- | --- | --- |
|  | 3 | 75.0% |
| / | 1 | 25.0% |

#### Most occurring blocks

| Value | Count | Frequency (%) |
| --- | --- | --- |
| ASCII | 26 | 100.0% |

#### Most frequent character per block

##### *ASCII*

| Value | Count | Frequency (%) |
| --- | --- | --- |
| S | 3 | 11.5% |
| u | 3 | 11.5% |
| s | 3 | 11.5% |
| p | 3 | 11.5% |
|  | 3 | 11.5% |
| T | 2 | 7.7% |
| B | 2 | 7.7% |
| c | 2 | 7.7% |
| / | 1 | 3.8% |
| a | 1 | 3.8% |
| Other values (3) | 3 | 11.5% |

NECROP  
Categorical

|  |  |
| --- | --- |
| Distinct | 1 |
| Distinct (%) | 33.3% |
| Missing | 0 |
| Missing (%) | 0.0% |
| Memory size | 48.0 B |

|  |  |
| --- | --- |
| N/realiz | 3 |

More details

- Overview
- Categories
- Words
- Characters

Length

|  |  |
| --- | --- |
| Max length | 8 |
| Median length | 8 |
| Mean length | 8 |
| Min length | 8 |

Characters and Unicode

|  |  |
| --- | --- |
| Total characters | 24 |
| Distinct characters | 8 |
| Distinct categories | 3 ? |
| Distinct scripts | 2 ? |
| Distinct blocks | 1 ? |

The Unicode Standard assigns character properties to each code point, which can be used to analyse textual variables.

Unique

|  |  |
| --- | --- |
| Unique | 0 ? |
| Unique (%) | 0.0% |

Sample

|  |  |
| --- | --- |
| 1st row | N/realiz |
| 2nd row | N/realiz |
| 3rd row | N/realiz |

#### Common Values

| Value | Count | Frequency (%) |
| --- | --- | --- |
| N/realiz | 3 | 100.0% |

#### Length

xml version="1.0" encoding="utf-8" standalone="no"?2023-10-31T16:30:07.256854image/svg+xmlMatplotlib v3.6.0, https://matplotlib.org/ 

Histogram of lengths of the category

#### Common Values (Plot)

xml version="1.0" encoding="utf-8" standalone="no"?2023-10-31T16:30:07.392210image/svg+xmlMatplotlib v3.6.0, https://matplotlib.org/

| Value | Count | Frequency (%) |
| --- | --- | --- |
| n/realiz | 3 | 100.0% |

- Characters
- Categories
- Scripts
- Blocks

#### Most occurring characters

| Value | Count | Frequency (%) |
| --- | --- | --- |
| N | 3 | 12.5% |
| / | 3 | 12.5% |
| r | 3 | 12.5% |
| e | 3 | 12.5% |
| a | 3 | 12.5% |
| l | 3 | 12.5% |
| i | 3 | 12.5% |
| z | 3 | 12.5% |

#### Most occurring categories

| Value | Count | Frequency (%) |
| --- | --- | --- |
| Lowercase Letter | 18 | 75.0% |
| Uppercase Letter | 3 | 12.5% |
| Other Punctuation | 3 | 12.5% |

#### Most frequent character per category

##### *Lowercase Letter*

| Value | Count | Frequency (%) |
| --- | --- | --- |
| r | 3 | 16.7% |
| e | 3 | 16.7% |
| a | 3 | 16.7% |
| l | 3 | 16.7% |
| i | 3 | 16.7% |
| z | 3 | 16.7% |

##### *Uppercase Letter*

| Value | Count | Frequency (%) |
| --- | --- | --- |
| N | 3 | 100.0% |

##### *Other Punctuation*

| Value | Count | Frequency (%) |
| --- | --- | --- |
| / | 3 | 100.0% |

#### Most occurring scripts

| Value | Count | Frequency (%) |
| --- | --- | --- |
| Latin | 21 | 87.5% |
| Common | 3 | 12.5% |

#### Most frequent character per script

##### *Latin*

| Value | Count | Frequency (%) |
| --- | --- | --- |
| N | 3 | 14.3% |
| r | 3 | 14.3% |
| e | 3 | 14.3% |
| a | 3 | 14.3% |
| l | 3 | 14.3% |
| i | 3 | 14.3% |
| z | 3 | 14.3% |

##### *Common*

| Value | Count | Frequency (%) |
| --- | --- | --- |
| / | 3 | 100.0% |

#### Most occurring blocks

| Value | Count | Frequency (%) |
| --- | --- | --- |
| ASCII | 24 | 100.0% |

#### Most frequent character per block

##### *ASCII*

| Value | Count | Frequency (%) |
| --- | --- | --- |
| N | 3 | 12.5% |
| / | 3 | 12.5% |
| r | 3 | 12.5% |
| e | 3 | 12.5% |
| a | 3 | 12.5% |
| l | 3 | 12.5% |
| i | 3 | 12.5% |
| z | 3 | 12.5% |

hiv  
Categorical

|  |  |
| --- | --- |
| Distinct | 1 |
| Distinct (%) | 33.3% |
| Missing | 0 |
| Missing (%) | 0.0% |
| Memory size | 48.0 B |

|  |  |
| --- | --- |
| Pos | 3 |

More details

- Overview
- Categories
- Words
- Characters

Length

|  |  |
| --- | --- |
| Max length | 3 |
| Median length | 3 |
| Mean length | 3 |
| Min length | 3 |

Characters and Unicode

|  |  |
| --- | --- |
| Total characters | 9 |
| Distinct characters | 3 |
| Distinct categories | 2 ? |
| Distinct scripts | 1 ? |
| Distinct blocks | 1 ? |

The Unicode Standard assigns character properties to each code point, which can be used to analyse textual variables.

Unique

|  |  |
| --- | --- |
| Unique | 0 ? |
| Unique (%) | 0.0% |

Sample

|  |  |
| --- | --- |
| 1st row | Pos |
| 2nd row | Pos |
| 3rd row | Pos |

#### Common Values

| Value | Count | Frequency (%) |
| --- | --- | --- |
| Pos | 3 | 100.0% |

#### Length

xml version="1.0" encoding="utf-8" standalone="no"?2023-10-31T16:30:07.499933image/svg+xmlMatplotlib v3.6.0, https://matplotlib.org/ 

Histogram of lengths of the category

#### Common Values (Plot)

xml version="1.0" encoding="utf-8" standalone="no"?2023-10-31T16:30:07.648524image/svg+xmlMatplotlib v3.6.0, https://matplotlib.org/

| Value | Count | Frequency (%) |
| --- | --- | --- |
| pos | 3 | 100.0% |

- Characters
- Categories
- Scripts
- Blocks

#### Most occurring characters

| Value | Count | Frequency (%) |
| --- | --- | --- |
| P | 3 | 33.3% |
| o | 3 | 33.3% |
| s | 3 | 33.3% |

#### Most occurring categories

| Value | Count | Frequency (%) |
| --- | --- | --- |
| Lowercase Letter | 6 | 66.7% |
| Uppercase Letter | 3 | 33.3% |

#### Most frequent character per category

##### *Lowercase Letter*

| Value | Count | Frequency (%) |
| --- | --- | --- |
| o | 3 | 50.0% |
| s | 3 | 50.0% |

##### *Uppercase Letter*

| Value | Count | Frequency (%) |
| --- | --- | --- |
| P | 3 | 100.0% |

#### Most occurring scripts

| Value | Count | Frequency (%) |
| --- | --- | --- |
| Latin | 9 | 100.0% |

#### Most frequent character per script

##### *Latin*

| Value | Count | Frequency (%) |
| --- | --- | --- |
| P | 3 | 33.3% |
| o | 3 | 33.3% |
| s | 3 | 33.3% |

#### Most occurring blocks

| Value | Count | Frequency (%) |
| --- | --- | --- |
| ASCII | 9 | 100.0% |

#### Most frequent character per block

##### *ASCII*

| Value | Count | Frequency (%) |
| --- | --- | --- |
| P | 3 | 33.3% |
| o | 3 | 33.3% |
| s | 3 | 33.3% |

aids  
Categorical

|  |  |
| --- | --- |
| Distinct | 1 |
| Distinct (%) | 33.3% |
| Missing | 0 |
| Missing (%) | 0.0% |
| Memory size | 48.0 B |

|  |  |
| --- | --- |
| S | 3 |

More details

- Overview
- Categories
- Words
- Characters

Length

|  |  |
| --- | --- |
| Max length | 1 |
| Median length | 1 |
| Mean length | 1 |
| Min length | 1 |

Characters and Unicode

|  |  |
| --- | --- |
| Total characters | 3 |
| Distinct characters | 1 |
| Distinct categories | 1 ? |
| Distinct scripts | 1 ? |
| Distinct blocks | 1 ? |

The Unicode Standard assigns character properties to each code point, which can be used to analyse textual variables.

Unique

|  |  |
| --- | --- |
| Unique | 0 ? |
| Unique (%) | 0.0% |

Sample

|  |  |
| --- | --- |
| 1st row | S |
| 2nd row | S |
| 3rd row | S |

#### Common Values

| Value | Count | Frequency (%) |
| --- | --- | --- |
| S | 3 | 100.0% |

#### Length

xml version="1.0" encoding="utf-8" standalone="no"?2023-10-31T16:30:07.754459image/svg+xmlMatplotlib v3.6.0, https://matplotlib.org/ 

Histogram of lengths of the category

#### Common Values (Plot)

xml version="1.0" encoding="utf-8" standalone="no"?2023-10-31T16:30:07.890081image/svg+xmlMatplotlib v3.6.0, https://matplotlib.org/

| Value | Count | Frequency (%) |
| --- | --- | --- |
| s | 3 | 100.0% |

- Characters
- Categories
- Scripts
- Blocks

#### Most occurring characters

| Value | Count | Frequency (%) |
| --- | --- | --- |
| S | 3 | 100.0% |

#### Most occurring categories

| Value | Count | Frequency (%) |
| --- | --- | --- |
| Uppercase Letter | 3 | 100.0% |

#### Most frequent character per category

##### *Uppercase Letter*

| Value | Count | Frequency (%) |
| --- | --- | --- |
| S | 3 | 100.0% |

#### Most occurring scripts

| Value | Count | Frequency (%) |
| --- | --- | --- |
| Latin | 3 | 100.0% |

#### Most frequent character per script

##### *Latin*

| Value | Count | Frequency (%) |
| --- | --- | --- |
| S | 3 | 100.0% |

#### Most occurring blocks

| Value | Count | Frequency (%) |
| --- | --- | --- |
| ASCII | 3 | 100.0% |

#### Most frequent character per block

##### *ASCII*

| Value | Count | Frequency (%) |
| --- | --- | --- |
| S | 3 | 100.0% |

DIABETES  
Boolean

|  |  |
| --- | --- |
| Distinct | 1 |
| Distinct (%) | 33.3% |
| Missing | 0 |
| Missing (%) | 0.0% |
| Memory size | 27.0 B |

|  |  |
| --- | --- |
| False | 3 |

More details

- Common Values (Table)
- Common Values (Plot)

| Value | Count | Frequency (%) |
| --- | --- | --- |
| False | 3 | 100.0% |

xml version="1.0" encoding="utf-8" standalone="no"?2023-10-31T16:30:08.002398image/svg+xmlMatplotlib v3.6.0, https://matplotlib.org/

ALCOOLISMO  
Boolean

|  |  |
| --- | --- |
| Distinct | 1 |
| Distinct (%) | 33.3% |
| Missing | 0 |
| Missing (%) | 0.0% |
| Memory size | 27.0 B |

|  |  |
| --- | --- |
| False | 3 |

More details

- Common Values (Table)
- Common Values (Plot)

| Value | Count | Frequency (%) |
| --- | --- | --- |
| False | 3 | 100.0% |

xml version="1.0" encoding="utf-8" standalone="no"?2023-10-31T16:30:08.123497image/svg+xmlMatplotlib v3.6.0, https://matplotlib.org/

MENTAL  
Boolean

|  |  |
| --- | --- |
| Distinct | 1 |
| Distinct (%) | 33.3% |
| Missing | 0 |
| Missing (%) | 0.0% |
| Memory size | 27.0 B |

|  |  |
| --- | --- |
| False | 3 |

More details

- Common Values (Table)
- Common Values (Plot)

| Value | Count | Frequency (%) |
| --- | --- | --- |
| False | 3 | 100.0% |

xml version="1.0" encoding="utf-8" standalone="no"?2023-10-31T16:30:08.236134image/svg+xmlMatplotlib v3.6.0, https://matplotlib.org/

DROGADICAO  
Boolean

|  |  |
| --- | --- |
| Distinct | 1 |
| Distinct (%) | 33.3% |
| Missing | 0 |
| Missing (%) | 0.0% |
| Memory size | 27.0 B |

|  |  |
| --- | --- |
| False | 3 |

More details

- Common Values (Table)
- Common Values (Plot)

| Value | Count | Frequency (%) |
| --- | --- | --- |
| False | 3 | 100.0% |

xml version="1.0" encoding="utf-8" standalone="no"?2023-10-31T16:30:08.351913image/svg+xmlMatplotlib v3.6.0, https://matplotlib.org/

TABAGISMO  
Categorical

|  |  |
| --- | --- |
| Distinct | 2 |
| Distinct (%) | 66.7% |
| Missing | 0 |
| Missing (%) | 0.0% |
| Memory size | 48.0 B |

|  |  |
| --- | --- |
| N | 2 |
| S | 1 |

More details

- Overview
- Categories
- Words
- Characters

Length

|  |  |
| --- | --- |
| Max length | 1 |
| Median length | 1 |
| Mean length | 1 |
| Min length | 1 |

Characters and Unicode

|  |  |
| --- | --- |
| Total characters | 3 |
| Distinct characters | 2 |
| Distinct categories | 1 ? |
| Distinct scripts | 1 ? |
| Distinct blocks | 1 ? |

The Unicode Standard assigns character properties to each code point, which can be used to analyse textual variables.

Unique

|  |  |
| --- | --- |
| Unique | 1 ? |
| Unique (%) | 33.3% |

Sample

|  |  |
| --- | --- |
| 1st row | N |
| 2nd row | N |
| 3rd row | S |

#### Common Values

| Value | Count | Frequency (%) |
| --- | --- | --- |
| N | 2 | 66.7% |
| S | 1 | 33.3% |

#### Length

xml version="1.0" encoding="utf-8" standalone="no"?2023-10-31T16:30:08.462973image/svg+xmlMatplotlib v3.6.0, https://matplotlib.org/ 

Histogram of lengths of the category

#### Common Values (Plot)

xml version="1.0" encoding="utf-8" standalone="no"?2023-10-31T16:30:08.599623image/svg+xmlMatplotlib v3.6.0, https://matplotlib.org/

| Value | Count | Frequency (%) |
| --- | --- | --- |
| n | 2 | 66.7% |
| s | 1 | 33.3% |

- Characters
- Categories
- Scripts
- Blocks

#### Most occurring characters

| Value | Count | Frequency (%) |
| --- | --- | --- |
| N | 2 | 66.7% |
| S | 1 | 33.3% |

#### Most occurring categories

| Value | Count | Frequency (%) |
| --- | --- | --- |
| Uppercase Letter | 3 | 100.0% |

#### Most frequent character per category

##### *Uppercase Letter*

| Value | Count | Frequency (%) |
| --- | --- | --- |
| N | 2 | 66.7% |
| S | 1 | 33.3% |

#### Most occurring scripts

| Value | Count | Frequency (%) |
| --- | --- | --- |
| Latin | 3 | 100.0% |

#### Most frequent character per script

##### *Latin*

| Value | Count | Frequency (%) |
| --- | --- | --- |
| N | 2 | 66.7% |
| S | 1 | 33.3% |

#### Most occurring blocks

| Value | Count | Frequency (%) |
| --- | --- | --- |
| ASCII | 3 | 100.0% |

#### Most frequent character per block

##### *ASCII*

| Value | Count | Frequency (%) |
| --- | --- | --- |
| N | 2 | 66.7% |
| S | 1 | 33.3% |

motMudEsquema  
Categorical

|  |  |
| --- | --- |
| Distinct | 1 |
| Distinct (%) | 33.3% |
| Missing | 0 |
| Missing (%) | 0.0% |
| Memory size | 48.0 B |

|  |  |
| --- | --- |
| Nulo | 3 |

More details

- Overview
- Categories
- Words
- Characters

Length

|  |  |
| --- | --- |
| Max length | 4 |
| Median length | 4 |
| Mean length | 4 |
| Min length | 4 |

Characters and Unicode

|  |  |
| --- | --- |
| Total characters | 12 |
| Distinct characters | 4 |
| Distinct categories | 2 ? |
| Distinct scripts | 1 ? |
| Distinct blocks | 1 ? |

The Unicode Standard assigns character properties to each code point, which can be used to analyse textual variables.

Unique

|  |  |
| --- | --- |
| Unique | 0 ? |
| Unique (%) | 0.0% |

Sample

|  |  |
| --- | --- |
| 1st row | Nulo |
| 2nd row | Nulo |
| 3rd row | Nulo |

#### Common Values

| Value | Count | Frequency (%) |
| --- | --- | --- |
| Nulo | 3 | 100.0% |

#### Length

xml version="1.0" encoding="utf-8" standalone="no"?2023-10-31T16:30:08.716057image/svg+xmlMatplotlib v3.6.0, https://matplotlib.org/ 

Histogram of lengths of the category

#### Common Values (Plot)

xml version="1.0" encoding="utf-8" standalone="no"?2023-10-31T16:30:08.846502image/svg+xmlMatplotlib v3.6.0, https://matplotlib.org/

| Value | Count | Frequency (%) |
| --- | --- | --- |
| nulo | 3 | 100.0% |

- Characters
- Categories
- Scripts
- Blocks

#### Most occurring characters

| Value | Count | Frequency (%) |
| --- | --- | --- |
| N | 3 | 25.0% |
| u | 3 | 25.0% |
| l | 3 | 25.0% |
| o | 3 | 25.0% |

#### Most occurring categories

| Value | Count | Frequency (%) |
| --- | --- | --- |
| Lowercase Letter | 9 | 75.0% |
| Uppercase Letter | 3 | 25.0% |

#### Most frequent character per category

##### *Lowercase Letter*

| Value | Count | Frequency (%) |
| --- | --- | --- |
| u | 3 | 33.3% |
| l | 3 | 33.3% |
| o | 3 | 33.3% |

##### *Uppercase Letter*

| Value | Count | Frequency (%) |
| --- | --- | --- |
| N | 3 | 100.0% |

#### Most occurring scripts

| Value | Count | Frequency (%) |
| --- | --- | --- |
| Latin | 12 | 100.0% |

#### Most frequent character per script

##### *Latin*

| Value | Count | Frequency (%) |
| --- | --- | --- |
| N | 3 | 25.0% |
| u | 3 | 25.0% |
| l | 3 | 25.0% |
| o | 3 | 25.0% |

#### Most occurring blocks

| Value | Count | Frequency (%) |
| --- | --- | --- |
| ASCII | 12 | 100.0% |

#### Most frequent character per block

##### *ASCII*

| Value | Count | Frequency (%) |
| --- | --- | --- |
| N | 3 | 25.0% |
| u | 3 | 25.0% |
| l | 3 | 25.0% |
| o | 3 | 25.0% |

tipoTrat  
Categorical

|  |  |
| --- | --- |
| Distinct | 2 |
| Distinct (%) | 66.7% |
| Missing | 0 |
| Missing (%) | 0.0% |
| Memory size | 48.0 B |

|  |  |
| --- | --- |
| Supervisionado | 2 |
| Auto-Administrado | 1 |

More details

- Overview
- Categories
- Words
- Characters

Length

|  |  |
| --- | --- |
| Max length | 17 |
| Median length | 14 |
| Mean length | 15 |
| Min length | 14 |

Characters and Unicode

|  |  |
| --- | --- |
| Total characters | 45 |
| Distinct characters | 16 |
| Distinct categories | 3 ? |
| Distinct scripts | 2 ? |
| Distinct blocks | 1 ? |

The Unicode Standard assigns character properties to each code point, which can be used to analyse textual variables.

Unique

|  |  |
| --- | --- |
| Unique | 1 ? |
| Unique (%) | 33.3% |

Sample

|  |  |
| --- | --- |
| 1st row | Auto-Administrado |
| 2nd row | Supervisionado |
| 3rd row | Supervisionado |

#### Common Values

| Value | Count | Frequency (%) |
| --- | --- | --- |
| Supervisionado | 2 | 66.7% |
| Auto-Administrado | 1 | 33.3% |

#### Length

xml version="1.0" encoding="utf-8" standalone="no"?2023-10-31T16:30:08.959880image/svg+xmlMatplotlib v3.6.0, https://matplotlib.org/ 

Histogram of lengths of the category

#### Common Values (Plot)

xml version="1.0" encoding="utf-8" standalone="no"?2023-10-31T16:30:09.109543image/svg+xmlMatplotlib v3.6.0, https://matplotlib.org/

| Value | Count | Frequency (%) |
| --- | --- | --- |
| supervisionado | 2 | 66.7% |
| auto-administrado | 1 | 33.3% |

- Characters
- Categories
- Scripts
- Blocks

#### Most occurring characters

| Value | Count | Frequency (%) |
| --- | --- | --- |
| i | 6 | 13.3% |
| o | 6 | 13.3% |
| d | 4 | 8.9% |
| u | 3 | 6.7% |
| r | 3 | 6.7% |
| s | 3 | 6.7% |
| n | 3 | 6.7% |
| a | 3 | 6.7% |
| S | 2 | 4.4% |
| p | 2 | 4.4% |
| Other values (6) | 10 | 22.2% |

#### Most occurring categories

| Value | Count | Frequency (%) |
| --- | --- | --- |
| Lowercase Letter | 40 | 88.9% |
| Uppercase Letter | 4 | 8.9% |
| Dash Punctuation | 1 | 2.2% |

#### Most frequent character per category

##### *Lowercase Letter*

| Value | Count | Frequency (%) |
| --- | --- | --- |
| i | 6 | 15.0% |
| o | 6 | 15.0% |
| d | 4 | 10.0% |
| u | 3 | 7.5% |
| r | 3 | 7.5% |
| s | 3 | 7.5% |
| n | 3 | 7.5% |
| a | 3 | 7.5% |
| p | 2 | 5.0% |
| e | 2 | 5.0% |
| Other values (3) | 5 | 12.5% |

##### *Uppercase Letter*

| Value | Count | Frequency (%) |
| --- | --- | --- |
| S | 2 | 50.0% |
| A | 2 | 50.0% |

##### *Dash Punctuation*

| Value | Count | Frequency (%) |
| --- | --- | --- |
| - | 1 | 100.0% |

#### Most occurring scripts

| Value | Count | Frequency (%) |
| --- | --- | --- |
| Latin | 44 | 97.8% |
| Common | 1 | 2.2% |

#### Most frequent character per script

##### *Latin*

| Value | Count | Frequency (%) |
| --- | --- | --- |
| i | 6 | 13.6% |
| o | 6 | 13.6% |
| d | 4 | 9.1% |
| u | 3 | 6.8% |
| r | 3 | 6.8% |
| s | 3 | 6.8% |
| n | 3 | 6.8% |
| a | 3 | 6.8% |
| S | 2 | 4.5% |
| p | 2 | 4.5% |
| Other values (5) | 9 | 20.5% |

##### *Common*

| Value | Count | Frequency (%) |
| --- | --- | --- |
| - | 1 | 100.0% |

#### Most occurring blocks

| Value | Count | Frequency (%) |
| --- | --- | --- |
| ASCII | 45 | 100.0% |

#### Most frequent character per block

##### *ASCII*

| Value | Count | Frequency (%) |
| --- | --- | --- |
| i | 6 | 13.3% |
| o | 6 | 13.3% |
| d | 4 | 8.9% |
| u | 3 | 6.7% |
| r | 3 | 6.7% |
| s | 3 | 6.7% |
| n | 3 | 6.7% |
| a | 3 | 6.7% |
| S | 2 | 4.4% |
| p | 2 | 4.4% |
| Other values (6) | 10 | 22.2% |

idade  
Categorical

`HIGH CORRELATION`  `UNIFORM`  `UNIQUE`

|  |  |
| --- | --- |
| Distinct | 3 |
| Distinct (%) | 100.0% |
| Missing | 0 |
| Missing (%) | 0.0% |
| Memory size | 48.0 B |

|  |  |
| --- | --- |
| 0\_22 | 1 |
| 40\_54 | 1 |
| 23\_39 | 1 |

More details

- Overview
- Categories
- Words
- Characters

Length

|  |  |
| --- | --- |
| Max length | 5 |
| Median length | 5 |
| Mean length | 4.6666667 |
| Min length | 4 |

Characters and Unicode

|  |  |
| --- | --- |
| Total characters | 14 |
| Distinct characters | 7 |
| Distinct categories | 2 ? |
| Distinct scripts | 1 ? |
| Distinct blocks | 1 ? |

The Unicode Standard assigns character properties to each code point, which can be used to analyse textual variables.

Unique

|  |  |
| --- | --- |
| Unique | 3 ? |
| Unique (%) | 100.0% |

Sample

|  |  |
| --- | --- |
| 1st row | 0\_22 |
| 2nd row | 40\_54 |
| 3rd row | 23\_39 |

#### Common Values

| Value | Count | Frequency (%) |
| --- | --- | --- |
| 0\_22 | 1 | 33.3% |
| 40\_54 | 1 | 33.3% |
| 23\_39 | 1 | 33.3% |

#### Length

xml version="1.0" encoding="utf-8" standalone="no"?2023-10-31T16:30:09.228696image/svg+xmlMatplotlib v3.6.0, https://matplotlib.org/ 

Histogram of lengths of the category

#### Common Values (Plot)

xml version="1.0" encoding="utf-8" standalone="no"?2023-10-31T16:30:09.379882image/svg+xmlMatplotlib v3.6.0, https://matplotlib.org/

| Value | Count | Frequency (%) |
| --- | --- | --- |
| 0\_22 | 1 | 33.3% |
| 40\_54 | 1 | 33.3% |
| 23\_39 | 1 | 33.3% |

- Characters
- Categories
- Scripts
- Blocks

#### Most occurring characters

| Value | Count | Frequency (%) |
| --- | --- | --- |
| \_ | 3 | 21.4% |
| 2 | 3 | 21.4% |
| 0 | 2 | 14.3% |
| 4 | 2 | 14.3% |
| 3 | 2 | 14.3% |
| 5 | 1 | 7.1% |
| 9 | 1 | 7.1% |

#### Most occurring categories

| Value | Count | Frequency (%) |
| --- | --- | --- |
| Decimal Number | 11 | 78.6% |
| Connector Punctuation | 3 | 21.4% |

#### Most frequent character per category

##### *Decimal Number*

| Value | Count | Frequency (%) |
| --- | --- | --- |
| 2 | 3 | 27.3% |
| 0 | 2 | 18.2% |
| 4 | 2 | 18.2% |
| 3 | 2 | 18.2% |
| 5 | 1 | 9.1% |
| 9 | 1 | 9.1% |

##### *Connector Punctuation*

| Value | Count | Frequency (%) |
| --- | --- | --- |
| \_ | 3 | 100.0% |

#### Most occurring scripts

| Value | Count | Frequency (%) |
| --- | --- | --- |
| Common | 14 | 100.0% |

#### Most frequent character per script

##### *Common*

| Value | Count | Frequency (%) |
| --- | --- | --- |
| \_ | 3 | 21.4% |
| 2 | 3 | 21.4% |
| 0 | 2 | 14.3% |
| 4 | 2 | 14.3% |
| 3 | 2 | 14.3% |
| 5 | 1 | 7.1% |
| 9 | 1 | 7.1% |

#### Most occurring blocks

| Value | Count | Frequency (%) |
| --- | --- | --- |
| ASCII | 14 | 100.0% |

#### Most frequent character per block

##### *ASCII*

| Value | Count | Frequency (%) |
| --- | --- | --- |
| \_ | 3 | 21.4% |
| 2 | 3 | 21.4% |
| 0 | 2 | 14.3% |
| 4 | 2 | 14.3% |
| 3 | 2 | 14.3% |
| 5 | 1 | 7.1% |
| 9 | 1 | 7.1% |

HISTOPATOL  
Categorical

|  |  |
| --- | --- |
| Distinct | 1 |
| Distinct (%) | 33.3% |
| Missing | 0 |
| Missing (%) | 0.0% |
| Memory size | 48.0 B |

|  |  |
| --- | --- |
| N/realiz | 3 |

More details

- Overview
- Categories
- Words
- Characters

Length

|  |  |
| --- | --- |
| Max length | 8 |
| Median length | 8 |
| Mean length | 8 |
| Min length | 8 |

Characters and Unicode

|  |  |
| --- | --- |
| Total characters | 24 |
| Distinct characters | 8 |
| Distinct categories | 3 ? |
| Distinct scripts | 2 ? |
| Distinct blocks | 1 ? |

The Unicode Standard assigns character properties to each code point, which can be used to analyse textual variables.

Unique

|  |  |
| --- | --- |
| Unique | 0 ? |
| Unique (%) | 0.0% |

Sample

|  |  |
| --- | --- |
| 1st row | N/realiz |
| 2nd row | N/realiz |
| 3rd row | N/realiz |

#### Common Values

| Value | Count | Frequency (%) |
| --- | --- | --- |
| N/realiz | 3 | 100.0% |

#### Length

xml version="1.0" encoding="utf-8" standalone="no"?2023-10-31T16:30:09.506478image/svg+xmlMatplotlib v3.6.0, https://matplotlib.org/ 

Histogram of lengths of the category

#### Common Values (Plot)

xml version="1.0" encoding="utf-8" standalone="no"?2023-10-31T16:30:09.647341image/svg+xmlMatplotlib v3.6.0, https://matplotlib.org/

| Value | Count | Frequency (%) |
| --- | --- | --- |
| n/realiz | 3 | 100.0% |

- Characters
- Categories
- Scripts
- Blocks

#### Most occurring characters

| Value | Count | Frequency (%) |
| --- | --- | --- |
| N | 3 | 12.5% |
| / | 3 | 12.5% |
| r | 3 | 12.5% |
| e | 3 | 12.5% |
| a | 3 | 12.5% |
| l | 3 | 12.5% |
| i | 3 | 12.5% |
| z | 3 | 12.5% |

#### Most occurring categories

| Value | Count | Frequency (%) |
| --- | --- | --- |
| Lowercase Letter | 18 | 75.0% |
| Uppercase Letter | 3 | 12.5% |
| Other Punctuation | 3 | 12.5% |

#### Most frequent character per category

##### *Lowercase Letter*

| Value | Count | Frequency (%) |
| --- | --- | --- |
| r | 3 | 16.7% |
| e | 3 | 16.7% |
| a | 3 | 16.7% |
| l | 3 | 16.7% |
| i | 3 | 16.7% |
| z | 3 | 16.7% |

##### *Uppercase Letter*

| Value | Count | Frequency (%) |
| --- | --- | --- |
| N | 3 | 100.0% |

##### *Other Punctuation*

| Value | Count | Frequency (%) |
| --- | --- | --- |
| / | 3 | 100.0% |

#### Most occurring scripts

| Value | Count | Frequency (%) |
| --- | --- | --- |
| Latin | 21 | 87.5% |
| Common | 3 | 12.5% |

#### Most frequent character per script

##### *Latin*

| Value | Count | Frequency (%) |
| --- | --- | --- |
| N | 3 | 14.3% |
| r | 3 | 14.3% |
| e | 3 | 14.3% |
| a | 3 | 14.3% |
| l | 3 | 14.3% |
| i | 3 | 14.3% |
| z | 3 | 14.3% |

##### *Common*

| Value | Count | Frequency (%) |
| --- | --- | --- |
| / | 3 | 100.0% |

#### Most occurring blocks

| Value | Count | Frequency (%) |
| --- | --- | --- |
| ASCII | 24 | 100.0% |

#### Most frequent character per block

##### *ASCII*

| Value | Count | Frequency (%) |
| --- | --- | --- |
| N | 3 | 12.5% |
| / | 3 | 12.5% |
| r | 3 | 12.5% |
| e | 3 | 12.5% |
| a | 3 | 12.5% |
| l | 3 | 12.5% |
| i | 3 | 12.5% |
| z | 3 | 12.5% |

Status\_Resistencia  
Categorical

|  |  |
| --- | --- |
| Distinct | 1 |
| Distinct (%) | 33.3% |
| Missing | 0 |
| Missing (%) | 0.0% |
| Memory size | 48.0 B |

|  |  |
| --- | --- |
| 1 | 3 |

More details

- Overview
- Categories
- Words
- Characters

Length

|  |  |
| --- | --- |
| Max length | 1 |
| Median length | 1 |
| Mean length | 1 |
| Min length | 1 |

Characters and Unicode

|  |  |
| --- | --- |
| Total characters | 3 |
| Distinct characters | 1 |
| Distinct categories | 1 ? |
| Distinct scripts | 1 ? |
| Distinct blocks | 1 ? |

The Unicode Standard assigns character properties to each code point, which can be used to analyse textual variables.

Unique

|  |  |
| --- | --- |
| Unique | 0 ? |
| Unique (%) | 0.0% |

Sample

|  |  |
| --- | --- |
| 1st row | 1 |
| 2nd row | 1 |
| 3rd row | 1 |

#### Common Values

| Value | Count | Frequency (%) |
| --- | --- | --- |
| 1 | 3 | 100.0% |

#### Length

xml version="1.0" encoding="utf-8" standalone="no"?2023-10-31T16:30:09.761207image/svg+xmlMatplotlib v3.6.0, https://matplotlib.org/ 

Histogram of lengths of the category

#### Common Values (Plot)

xml version="1.0" encoding="utf-8" standalone="no"?2023-10-31T16:30:09.900504image/svg+xmlMatplotlib v3.6.0, https://matplotlib.org/

| Value | Count | Frequency (%) |
| --- | --- | --- |
| 1 | 3 | 100.0% |

- Characters
- Categories
- Scripts
- Blocks

#### Most occurring characters

| Value | Count | Frequency (%) |
| --- | --- | --- |
| 1 | 3 | 100.0% |

#### Most occurring categories

| Value | Count | Frequency (%) |
| --- | --- | --- |
| Decimal Number | 3 | 100.0% |

#### Most frequent character per category

##### *Decimal Number*

| Value | Count | Frequency (%) |
| --- | --- | --- |
| 1 | 3 | 100.0% |

#### Most occurring scripts

| Value | Count | Frequency (%) |
| --- | --- | --- |
| Common | 3 | 100.0% |

#### Most frequent character per script

##### *Common*

| Value | Count | Frequency (%) |
| --- | --- | --- |
| 1 | 3 | 100.0% |

#### Most occurring blocks

| Value | Count | Frequency (%) |
| --- | --- | --- |
| ASCII | 3 | 100.0% |

#### Most frequent character per block

##### *ASCII*

| Value | Count | Frequency (%) |
| --- | --- | --- |
| 1 | 3 | 100.0% |

Cluster  
Categorical

|  |  |
| --- | --- |
| Distinct | 1 |
| Distinct (%) | 33.3% |
| Missing | 0 |
| Missing (%) | 0.0% |
| Memory size | 48.0 B |

|  |  |
| --- | --- |
| 0 | 3 |

More details

- Overview
- Categories
- Words
- Characters

Length

|  |  |
| --- | --- |
| Max length | 1 |
| Median length | 1 |
| Mean length | 1 |
| Min length | 1 |

Characters and Unicode

|  |  |
| --- | --- |
| Total characters | 3 |
| Distinct characters | 1 |
| Distinct categories | 1 ? |
| Distinct scripts | 1 ? |
| Distinct blocks | 1 ? |

The Unicode Standard assigns character properties to each code point, which can be used to analyse textual variables.

Unique

|  |  |
| --- | --- |
| Unique | 0 ? |
| Unique (%) | 0.0% |

Sample

|  |  |
| --- | --- |
| 1st row | 0 |
| 2nd row | 0 |
| 3rd row | 0 |

#### Common Values

| Value | Count | Frequency (%) |
| --- | --- | --- |
| 0 | 3 | 100.0% |

#### Length

xml version="1.0" encoding="utf-8" standalone="no"?2023-10-31T16:30:10.005128image/svg+xmlMatplotlib v3.6.0, https://matplotlib.org/ 

Histogram of lengths of the category

#### Common Values (Plot)

xml version="1.0" encoding="utf-8" standalone="no"?2023-10-31T16:30:10.135100image/svg+xmlMatplotlib v3.6.0, https://matplotlib.org/

| Value | Count | Frequency (%) |
| --- | --- | --- |
| 0 | 3 | 100.0% |

- Characters
- Categories
- Scripts
- Blocks

#### Most occurring characters

| Value | Count | Frequency (%) |
| --- | --- | --- |
| 0 | 3 | 100.0% |

#### Most occurring categories

| Value | Count | Frequency (%) |
| --- | --- | --- |
| Decimal Number | 3 | 100.0% |

#### Most frequent character per category

##### *Decimal Number*

| Value | Count | Frequency (%) |
| --- | --- | --- |
| 0 | 3 | 100.0% |

#### Most occurring scripts

| Value | Count | Frequency (%) |
| --- | --- | --- |
| Common | 3 | 100.0% |

#### Most frequent character per script

##### *Common*

| Value | Count | Frequency (%) |
| --- | --- | --- |
| 0 | 3 | 100.0% |

#### Most occurring blocks

| Value | Count | Frequency (%) |
| --- | --- | --- |
| ASCII | 3 | 100.0% |

#### Most frequent character per block

##### *ASCII*

| Value | Count | Frequency (%) |
| --- | --- | --- |
| 0 | 3 | 100.0% |

Probabilidade  
Categorical

`HIGH CORRELATION`  `UNIFORM`  `UNIQUE`

|  |  |
| --- | --- |
| Distinct | 3 |
| Distinct (%) | 100.0% |
| Missing | 0 |
| Missing (%) | 0.0% |
| Memory size | 48.0 B |

|  |  |
| --- | --- |
| 0.39424201040664203 | 1 |
| 0.28457588664308664 | 1 |
| 0.4416289673559519 | 1 |

More details

- Overview
- Categories
- Words
- Characters

Length

|  |  |
| --- | --- |
| Max length | 19 |
| Median length | 19 |
| Mean length | 18.666667 |
| Min length | 18 |

Characters and Unicode

|  |  |
| --- | --- |
| Total characters | 56 |
| Distinct characters | 11 |
| Distinct categories | 2 ? |
| Distinct scripts | 1 ? |
| Distinct blocks | 1 ? |

The Unicode Standard assigns character properties to each code point, which can be used to analyse textual variables.

Unique

|  |  |
| --- | --- |
| Unique | 3 ? |
| Unique (%) | 100.0% |

Sample

|  |  |
| --- | --- |
| 1st row | 0.39424201040664203 |
| 2nd row | 0.28457588664308664 |
| 3rd row | 0.4416289673559519 |

#### Common Values

| Value | Count | Frequency (%) |
| --- | --- | --- |
| 0.39424201040664203 | 1 | 33.3% |
| 0.28457588664308664 | 1 | 33.3% |
| 0.4416289673559519 | 1 | 33.3% |

#### Length

xml version="1.0" encoding="utf-8" standalone="no"?2023-10-31T16:30:10.559540image/svg+xmlMatplotlib v3.6.0, https://matplotlib.org/ 

Histogram of lengths of the category

#### Common Values (Plot)

xml version="1.0" encoding="utf-8" standalone="no"?2023-10-31T16:30:10.709774image/svg+xmlMatplotlib v3.6.0, https://matplotlib.org/

| Value | Count | Frequency (%) |
| --- | --- | --- |
| 0.39424201040664203 | 1 | 33.3% |
| 0.28457588664308664 | 1 | 33.3% |
| 0.4416289673559519 | 1 | 33.3% |

- Characters
- Categories
- Scripts
- Blocks

#### Most occurring characters

| Value | Count | Frequency (%) |
| --- | --- | --- |
| 4 | 9 | 16.1% |
| 0 | 8 | 14.3% |
| 6 | 8 | 14.3% |
| 2 | 5 | 8.9% |
| 8 | 5 | 8.9% |
| 5 | 5 | 8.9% |
| 3 | 4 | 7.1% |
| 9 | 4 | 7.1% |
| . | 3 | 5.4% |
| 1 | 3 | 5.4% |

#### Most occurring categories

| Value | Count | Frequency (%) |
| --- | --- | --- |
| Decimal Number | 53 | 94.6% |
| Other Punctuation | 3 | 5.4% |

#### Most frequent character per category

##### *Decimal Number*

| Value | Count | Frequency (%) |
| --- | --- | --- |
| 4 | 9 | 17.0% |
| 0 | 8 | 15.1% |
| 6 | 8 | 15.1% |
| 2 | 5 | 9.4% |
| 8 | 5 | 9.4% |
| 5 | 5 | 9.4% |
| 3 | 4 | 7.5% |
| 9 | 4 | 7.5% |
| 1 | 3 | 5.7% |
| 7 | 2 | 3.8% |

##### *Other Punctuation*

| Value | Count | Frequency (%) |
| --- | --- | --- |
| . | 3 | 100.0% |

#### Most occurring scripts

| Value | Count | Frequency (%) |
| --- | --- | --- |
| Common | 56 | 100.0% |

#### Most frequent character per script

##### *Common*

| Value | Count | Frequency (%) |
| --- | --- | --- |
| 4 | 9 | 16.1% |
| 0 | 8 | 14.3% |
| 6 | 8 | 14.3% |
| 2 | 5 | 8.9% |
| 8 | 5 | 8.9% |
| 5 | 5 | 8.9% |
| 3 | 4 | 7.1% |
| 9 | 4 | 7.1% |
| . | 3 | 5.4% |
| 1 | 3 | 5.4% |

#### Most occurring blocks

| Value | Count | Frequency (%) |
| --- | --- | --- |
| ASCII | 56 | 100.0% |

#### Most frequent character per block

##### *ASCII*

| Value | Count | Frequency (%) |
| --- | --- | --- |
| 4 | 9 | 16.1% |
| 0 | 8 | 14.3% |
| 6 | 8 | 14.3% |
| 2 | 5 | 8.9% |
| 8 | 5 | 8.9% |
| 5 | 5 | 8.9% |
| 3 | 4 | 7.1% |
| 9 | 4 | 7.1% |
| . | 3 | 5.4% |
| 1 | 3 | 5.4% |

# Correlations

- Auto

- Heatmap
- Table

xml version="1.0" encoding="utf-8" standalone="no"?2023-10-31T16:30:10.854188image/svg+xmlMatplotlib v3.6.0, https://matplotlib.org/

|  | faixaEtaria | sexo | ESCOLARID | TIPOCUP | sitAtual | FORMACLIN1 | classif | descoberta | bac | BACOUTRO | RX | TABAGISMO | tipoTrat | idade | Probabilidade |
| --- | --- | --- | --- | --- | --- | --- | --- | --- | --- | --- | --- | --- | --- | --- | --- |
| faixaEtaria | 1.000 | 1.000 | 1.000 | 1.000 | 1.000 | 1.000 | 1.000 | 1.000 | 1.000 | 1.000 | 1.000 | 1.000 | 1.000 | 1.000 | 1.000 |
| sexo | 1.000 | 1.000 | 0.000 | 0.000 | 0.000 | 0.000 | 1.000 | 0.000 | 1.000 | 0.000 | 0.000 | 0.000 | 0.000 | 1.000 | 1.000 |
| ESCOLARID | 1.000 | 0.000 | 1.000 | 0.000 | 0.000 | 0.000 | 1.000 | 0.000 | 1.000 | 0.000 | 0.000 | 0.000 | 0.000 | 1.000 | 1.000 |
| TIPOCUP | 1.000 | 0.000 | 0.000 | 1.000 | 0.000 | 0.000 | 1.000 | 0.000 | 1.000 | 0.000 | 0.000 | 0.000 | 0.000 | 1.000 | 1.000 |
| sitAtual | 1.000 | 0.000 | 0.000 | 0.000 | 1.000 | 0.000 | 1.000 | 0.000 | 1.000 | 0.000 | 0.000 | 0.000 | 0.000 | 1.000 | 1.000 |
| FORMACLIN1 | 1.000 | 0.000 | 0.000 | 0.000 | 0.000 | 1.000 | 1.000 | 0.000 | 1.000 | 0.000 | 0.000 | 0.000 | 0.000 | 1.000 | 1.000 |
| classif | 1.000 | 1.000 | 1.000 | 1.000 | 1.000 | 1.000 | 1.000 | 1.000 | 1.000 | 1.000 | 1.000 | 1.000 | 1.000 | 1.000 | 1.000 |
| descoberta | 1.000 | 0.000 | 0.000 | 0.000 | 0.000 | 0.000 | 1.000 | 1.000 | 1.000 | 0.000 | 0.000 | 0.000 | 0.000 | 1.000 | 1.000 |
| bac | 1.000 | 1.000 | 1.000 | 1.000 | 1.000 | 1.000 | 1.000 | 1.000 | 1.000 | 1.000 | 1.000 | 1.000 | 1.000 | 1.000 | 1.000 |
| BACOUTRO | 1.000 | 0.000 | 0.000 | 0.000 | 0.000 | 0.000 | 1.000 | 0.000 | 1.000 | 1.000 | 0.000 | 0.000 | 0.000 | 1.000 | 1.000 |
| RX | 1.000 | 0.000 | 0.000 | 0.000 | 0.000 | 0.000 | 1.000 | 0.000 | 1.000 | 0.000 | 1.000 | 0.000 | 0.000 | 1.000 | 1.000 |
| TABAGISMO | 1.000 | 0.000 | 0.000 | 0.000 | 0.000 | 0.000 | 1.000 | 0.000 | 1.000 | 0.000 | 0.000 | 1.000 | 0.000 | 1.000 | 1.000 |
| tipoTrat | 1.000 | 0.000 | 0.000 | 0.000 | 0.000 | 0.000 | 1.000 | 0.000 | 1.000 | 0.000 | 0.000 | 0.000 | 1.000 | 1.000 | 1.000 |
| idade | 1.000 | 1.000 | 1.000 | 1.000 | 1.000 | 1.000 | 1.000 | 1.000 | 1.000 | 1.000 | 1.000 | 1.000 | 1.000 | 1.000 | 1.000 |
| Probabilidade | 1.000 | 1.000 | 1.000 | 1.000 | 1.000 | 1.000 | 1.000 | 1.000 | 1.000 | 1.000 | 1.000 | 1.000 | 1.000 | 1.000 | 1.000 |

# Missing values

- Count
- Matrix

xml version="1.0" encoding="utf-8" standalone="no"?2023-10-31T16:30:02.588618image/svg+xmlMatplotlib v3.6.0, https://matplotlib.org/ 

A simple visualization of nullity by column.

xml version="1.0" encoding="utf-8" standalone="no"?2023-10-31T16:30:03.105251image/svg+xmlMatplotlib v3.6.0, https://matplotlib.org/ 

Nullity matrix is a data-dense display which lets you quickly visually pick out patterns in data completion.

# Sample

- First rows
- Last rows

|  | faixaEtaria | sexo | ESCOLARID | TIPOCUP | sitAtual | tipoCaso | FORMACLIN1 | classif | descoberta | bac | BACOUTRO | cultEsc | RX | NECROP | hiv | aids | DIABETES | ALCOOLISMO | MENTAL | DROGADICAO | TABAGISMO | motMudEsquema | tipoTrat | idade | HISTOPATOL | Status\_Resistencia | Cluster | Probabilidade |
| --- | --- | --- | --- | --- | --- | --- | --- | --- | --- | --- | --- | --- | --- | --- | --- | --- | --- | --- | --- | --- | --- | --- | --- | --- | --- | --- | --- | --- |
| 462 | 20\_29 | F | De 4 a 7 anos | Dona de Casa | Abandono | Novo | Pul | P+E | Urgencia / Emergencia | N/realiz | Pos | N/realiz | Susp TB | N/realiz | Pos | S | N | N | N | N | N | Nulo | Auto-Administrado | 0\_22 | N/realiz | 1 | 0 | 0.394242 |
| 331 | 40\_49 | M | De 8 a 11 anos | Desempregado | Cura | Novo | Pleural | Ext | Elucidacao Diagn. em Internacao | Neg | N/realiz | N/realiz | Susp TB | N/realiz | Pos | S | N | N | N | N | N | Nulo | Supervisionado | 40\_54 | N/realiz | 1 | 0 | 0.284576 |
| 884 | 30\_39 | F | De 4 a 7 anos | Desempregado | Cura | Novo | Pul | Pul | Elucidacao Diagn. em Internacao | Pos | N/realiz | N/realiz | Susp c/cavid | N/realiz | Pos | S | N | N | N | N | S | Nulo | Supervisionado | 23\_39 | N/realiz | 1 | 0 | 0.441629 |

|  | faixaEtaria | sexo | ESCOLARID | TIPOCUP | sitAtual | tipoCaso | FORMACLIN1 | classif | descoberta | bac | BACOUTRO | cultEsc | RX | NECROP | hiv | aids | DIABETES | ALCOOLISMO | MENTAL | DROGADICAO | TABAGISMO | motMudEsquema | tipoTrat | idade | HISTOPATOL | Status\_Resistencia | Cluster | Probabilidade |
| --- | --- | --- | --- | --- | --- | --- | --- | --- | --- | --- | --- | --- | --- | --- | --- | --- | --- | --- | --- | --- | --- | --- | --- | --- | --- | --- | --- | --- |
| 462 | 20\_29 | F | De 4 a 7 anos | Dona de Casa | Abandono | Novo | Pul | P+E | Urgencia / Emergencia | N/realiz | Pos | N/realiz | Susp TB | N/realiz | Pos | S | N | N | N | N | N | Nulo | Auto-Administrado | 0\_22 | N/realiz | 1 | 0 | 0.394242 |
| 331 | 40\_49 | M | De 8 a 11 anos | Desempregado | Cura | Novo | Pleural | Ext | Elucidacao Diagn. em Internacao | Neg | N/realiz | N/realiz | Susp TB | N/realiz | Pos | S | N | N | N | N | N | Nulo | Supervisionado | 40\_54 | N/realiz | 1 | 0 | 0.284576 |
| 884 | 30\_39 | F | De 4 a 7 anos | Desempregado | Cura | Novo | Pul | Pul | Elucidacao Diagn. em Internacao | Pos | N/realiz | N/realiz | Susp c/cavid | N/realiz | Pos | S | N | N | N | N | S | Nulo | Supervisionado | 23\_39 | N/realiz | 1 | 0 | 0.441629 |

Report generated by YData.

 
