# Supplementary material for: Model for predicting drug resistance based on the clinical profile of tuberculosis patients using machine learning techniques
Source: PeerJ Comput Sci. 2024 Oct 14;10:e2246. doi: 10.7717/peerj-cs.2246 (PMC11623081; doi:10.7717/peerj-cs.2246)
Supplement: Supplemental Information 2 [file peerj-cs-10-2246-s002.zip › code/EDA/false_negative_2.html]

Pandas Profiling Report 

Toggle navigationPandas Profiling Report

- Overview
- Variables
- Correlations
- Missing values
- Sample

# Overview

- Overview
- Alerts 32
- Reproduction

Dataset statistics

|  |  |
| --- | --- |
| Number of variables | 28 |
| Number of observations | 4 |
| Missing cells | 0 |
| Missing cells (%) | 0.0% |
| Duplicate rows | 0 |
| Duplicate rows (%) | 0.0% |
| Total size in memory | 1000.0 B |
| Average record size in memory | 250.0 B |

Variable types

|  |  |
| --- | --- |
| Categorical | 23 |
| Boolean | 5 |

Alerts

| `sitAtual` has constant value "Cura" | Constant |
| `FORMACLIN1` has constant value "Pul" | Constant |
| `classif` has constant value "Pul" | Constant |
| `bac` has constant value "Pos" | Constant |
| `BACOUTRO` has constant value "N/realiz" | Constant |
| `NECROP` has constant value "N/realiz" | Constant |
| `hiv` has constant value "Neg" | Constant |
| `aids` has constant value "False" | Constant |
| `DIABETES` has constant value "False" | Constant |
| `ALCOOLISMO` has constant value "False" | Constant |
| `MENTAL` has constant value "False" | Constant |
| `TABAGISMO` has constant value "False" | Constant |
| `tipoTrat` has constant value "Supervisionado" | Constant |
| `HISTOPATOL` has constant value "N/realiz" | Constant |
| `Status_Resistencia` has constant value "1" | Constant |
| `Cluster` has constant value "1" | Constant |
| `faixaEtaria` is highly overall correlated with `sexo` and 6 other fields | High correlation |
| `sexo` is highly overall correlated with `faixaEtaria` and 3 other fields | High correlation |
| `ESCOLARID` is highly overall correlated with `faixaEtaria` and 10 other fields | High correlation |
| `TIPOCUP` is highly overall correlated with `faixaEtaria` and 4 other fields | High correlation |
| `tipoCaso` is highly overall correlated with `faixaEtaria` and 3 other fields | High correlation |
| `descoberta` is highly overall correlated with `ESCOLARID` and 5 other fields | High correlation |
| `cultEsc` is highly overall correlated with `ESCOLARID` and 1 other fields | High correlation |
| `RX` is highly overall correlated with `ESCOLARID` and 2 other fields | High correlation |
| `DROGADICAO` is highly overall correlated with `faixaEtaria` and 4 other fields | High correlation |
| `motMudEsquema` is highly overall correlated with `ESCOLARID` and 2 other fields | High correlation |
| `idade` is highly overall correlated with `faixaEtaria` and 6 other fields | High correlation |
| `Probabilidade` is highly overall correlated with `faixaEtaria` and 10 other fields | High correlation |
| `ESCOLARID` is uniformly distributed | Uniform |
| `Probabilidade` is uniformly distributed | Uniform |
| `ESCOLARID` has unique values | Unique |
| `Probabilidade` has unique values | Unique |

Reproduction

|  |  |
| --- | --- |
| Analysis started | 2023-10-31 19:38:54.519391 |
| Analysis finished | 2023-10-31 19:38:56.599240 |
| Duration | 2.08 seconds |
| Software version | pandas-profiling v3.6.6 |
| Download configuration | config.json |

# Variables

Select ColumnsfaixaEtariasexoESCOLARIDTIPOCUPsitAtualtipoCasoFORMACLIN1classifdescobertabacBACOUTROcultEscRXNECROPhivaidsDIABETESALCOOLISMOMENTALDROGADICAOTABAGISMOmotMudEsquematipoTratidadeHISTOPATOLStatus\_ResistenciaClusterProbabilidade

faixaEtaria  
Categorical

|  |  |
| --- | --- |
| Distinct | 3 |
| Distinct (%) | 75.0% |
| Missing | 0 |
| Missing (%) | 0.0% |
| Memory size | 64.0 B |

|  |  |
| --- | --- |
| 40\_49 | 2 |
| 20\_29 | 1 |
| 50\_59 | 1 |

More details

- Overview
- Categories
- Words
- Characters

Length

|  |  |
| --- | --- |
| Max length | 5 |
| Median length | 5 |
| Mean length | 5 |
| Min length | 5 |

Characters and Unicode

|  |  |
| --- | --- |
| Total characters | 20 |
| Distinct characters | 6 |
| Distinct categories | 2 ? |
| Distinct scripts | 1 ? |
| Distinct blocks | 1 ? |

The Unicode Standard assigns character properties to each code point, which can be used to analyse textual variables.

Unique

|  |  |
| --- | --- |
| Unique | 2 ? |
| Unique (%) | 50.0% |

Sample

|  |  |
| --- | --- |
| 1st row | 20\_29 |
| 2nd row | 40\_49 |
| 3rd row | 40\_49 |
| 4th row | 50\_59 |

#### Common Values

| Value | Count | Frequency (%) |
| --- | --- | --- |
| 40\_49 | 2 | 50.0% |
| 20\_29 | 1 | 25.0% |
| 50\_59 | 1 | 25.0% |

#### Length

xml version="1.0" encoding="utf-8" standalone="no"?2023-10-31T16:38:56.661952image/svg+xmlMatplotlib v3.6.0, https://matplotlib.org/ 

Histogram of lengths of the category

#### Common Values (Plot)

xml version="1.0" encoding="utf-8" standalone="no"?2023-10-31T16:38:56.809106image/svg+xmlMatplotlib v3.6.0, https://matplotlib.org/

| Value | Count | Frequency (%) |
| --- | --- | --- |
| 40\_49 | 2 | 50.0% |
| 20\_29 | 1 | 25.0% |
| 50\_59 | 1 | 25.0% |

- Characters
- Categories
- Scripts
- Blocks

#### Most occurring characters

| Value | Count | Frequency (%) |
| --- | --- | --- |
| 4 | 4 | 20.0% |
| 0 | 4 | 20.0% |
| \_ | 4 | 20.0% |
| 9 | 4 | 20.0% |
| 2 | 2 | 10.0% |
| 5 | 2 | 10.0% |

#### Most occurring categories

| Value | Count | Frequency (%) |
| --- | --- | --- |
| Decimal Number | 16 | 80.0% |
| Connector Punctuation | 4 | 20.0% |

#### Most frequent character per category

##### *Decimal Number*

| Value | Count | Frequency (%) |
| --- | --- | --- |
| 4 | 4 | 25.0% |
| 0 | 4 | 25.0% |
| 9 | 4 | 25.0% |
| 2 | 2 | 12.5% |
| 5 | 2 | 12.5% |

##### *Connector Punctuation*

| Value | Count | Frequency (%) |
| --- | --- | --- |
| \_ | 4 | 100.0% |

#### Most occurring scripts

| Value | Count | Frequency (%) |
| --- | --- | --- |
| Common | 20 | 100.0% |

#### Most frequent character per script

##### *Common*

| Value | Count | Frequency (%) |
| --- | --- | --- |
| 4 | 4 | 20.0% |
| 0 | 4 | 20.0% |
| \_ | 4 | 20.0% |
| 9 | 4 | 20.0% |
| 2 | 2 | 10.0% |
| 5 | 2 | 10.0% |

#### Most occurring blocks

| Value | Count | Frequency (%) |
| --- | --- | --- |
| ASCII | 20 | 100.0% |

#### Most frequent character per block

##### *ASCII*

| Value | Count | Frequency (%) |
| --- | --- | --- |
| 4 | 4 | 20.0% |
| 0 | 4 | 20.0% |
| \_ | 4 | 20.0% |
| 9 | 4 | 20.0% |
| 2 | 2 | 10.0% |
| 5 | 2 | 10.0% |

sexo  
Categorical

|  |  |
| --- | --- |
| Distinct | 2 |
| Distinct (%) | 50.0% |
| Missing | 0 |
| Missing (%) | 0.0% |
| Memory size | 160.0 B |

|  |  |
| --- | --- |
| F | 3 |
| M | 1 |

More details

- Overview
- Categories
- Words
- Characters

Length

|  |  |
| --- | --- |
| Max length | 1 |
| Median length | 1 |
| Mean length | 1 |
| Min length | 1 |

Characters and Unicode

|  |  |
| --- | --- |
| Total characters | 4 |
| Distinct characters | 2 |
| Distinct categories | 1 ? |
| Distinct scripts | 1 ? |
| Distinct blocks | 1 ? |

The Unicode Standard assigns character properties to each code point, which can be used to analyse textual variables.

Unique

|  |  |
| --- | --- |
| Unique | 1 ? |
| Unique (%) | 25.0% |

Sample

|  |  |
| --- | --- |
| 1st row | F |
| 2nd row | F |
| 3rd row | F |
| 4th row | M |

#### Common Values

| Value | Count | Frequency (%) |
| --- | --- | --- |
| F | 3 | 75.0% |
| M | 1 | 25.0% |

#### Length

xml version="1.0" encoding="utf-8" standalone="no"?2023-10-31T16:38:56.928841image/svg+xmlMatplotlib v3.6.0, https://matplotlib.org/ 

Histogram of lengths of the category

#### Common Values (Plot)

xml version="1.0" encoding="utf-8" standalone="no"?2023-10-31T16:38:57.068447image/svg+xmlMatplotlib v3.6.0, https://matplotlib.org/

| Value | Count | Frequency (%) |
| --- | --- | --- |
| f | 3 | 75.0% |
| m | 1 | 25.0% |

- Characters
- Categories
- Scripts
- Blocks

#### Most occurring characters

| Value | Count | Frequency (%) |
| --- | --- | --- |
| F | 3 | 75.0% |
| M | 1 | 25.0% |

#### Most occurring categories

| Value | Count | Frequency (%) |
| --- | --- | --- |
| Uppercase Letter | 4 | 100.0% |

#### Most frequent character per category

##### *Uppercase Letter*

| Value | Count | Frequency (%) |
| --- | --- | --- |
| F | 3 | 75.0% |
| M | 1 | 25.0% |

#### Most occurring scripts

| Value | Count | Frequency (%) |
| --- | --- | --- |
| Latin | 4 | 100.0% |

#### Most frequent character per script

##### *Latin*

| Value | Count | Frequency (%) |
| --- | --- | --- |
| F | 3 | 75.0% |
| M | 1 | 25.0% |

#### Most occurring blocks

| Value | Count | Frequency (%) |
| --- | --- | --- |
| ASCII | 4 | 100.0% |

#### Most frequent character per block

##### *ASCII*

| Value | Count | Frequency (%) |
| --- | --- | --- |
| F | 3 | 75.0% |
| M | 1 | 25.0% |

ESCOLARID  
Categorical

`HIGH CORRELATION`  `UNIFORM`  `UNIQUE`

|  |  |
| --- | --- |
| Distinct | 4 |
| Distinct (%) | 100.0% |
| Missing | 0 |
| Missing (%) | 0.0% |
| Memory size | 64.0 B |

|  |  |
| --- | --- |
| De 4 a 7 anos | 1 |
| De 8 a 11 anos | 1 |
| De 1 a 3 anos | 1 |
| De 12 a 14 anos | 1 |

More details

- Overview
- Categories
- Words
- Characters

Length

|  |  |
| --- | --- |
| Max length | 15 |
| Median length | 14.5 |
| Mean length | 13.75 |
| Min length | 13 |

Characters and Unicode

|  |  |
| --- | --- |
| Total characters | 55 |
| Distinct characters | 13 |
| Distinct categories | 4 ? |
| Distinct scripts | 2 ? |
| Distinct blocks | 1 ? |

The Unicode Standard assigns character properties to each code point, which can be used to analyse textual variables.

Unique

|  |  |
| --- | --- |
| Unique | 4 ? |
| Unique (%) | 100.0% |

Sample

|  |  |
| --- | --- |
| 1st row | De 4 a 7 anos |
| 2nd row | De 8 a 11 anos |
| 3rd row | De 1 a 3 anos |
| 4th row | De 12 a 14 anos |

#### Common Values

| Value | Count | Frequency (%) |
| --- | --- | --- |
| De 4 a 7 anos | 1 | 25.0% |
| De 8 a 11 anos | 1 | 25.0% |
| De 1 a 3 anos | 1 | 25.0% |
| De 12 a 14 anos | 1 | 25.0% |

#### Length

xml version="1.0" encoding="utf-8" standalone="no"?2023-10-31T16:38:57.194671image/svg+xmlMatplotlib v3.6.0, https://matplotlib.org/ 

Histogram of lengths of the category

#### Common Values (Plot)

xml version="1.0" encoding="utf-8" standalone="no"?2023-10-31T16:38:57.364625image/svg+xmlMatplotlib v3.6.0, https://matplotlib.org/

| Value | Count | Frequency (%) |
| --- | --- | --- |
| de | 4 | 20.0% |
| a | 4 | 20.0% |
| anos | 4 | 20.0% |
| 4 | 1 | 5.0% |
| 7 | 1 | 5.0% |
| 8 | 1 | 5.0% |
| 11 | 1 | 5.0% |
| 1 | 1 | 5.0% |
| 3 | 1 | 5.0% |
| 12 | 1 | 5.0% |

- Characters
- Categories
- Scripts
- Blocks

#### Most occurring characters

| Value | Count | Frequency (%) |
| --- | --- | --- |
|  | 16 | 29.1% |
| a | 8 | 14.5% |
| 1 | 5 | 9.1% |
| D | 4 | 7.3% |
| e | 4 | 7.3% |
| n | 4 | 7.3% |
| o | 4 | 7.3% |
| s | 4 | 7.3% |
| 4 | 2 | 3.6% |
| 7 | 1 | 1.8% |
| Other values (3) | 3 | 5.5% |

#### Most occurring categories

| Value | Count | Frequency (%) |
| --- | --- | --- |
| Lowercase Letter | 24 | 43.6% |
| Space Separator | 16 | 29.1% |
| Decimal Number | 11 | 20.0% |
| Uppercase Letter | 4 | 7.3% |

#### Most frequent character per category

##### *Decimal Number*

| Value | Count | Frequency (%) |
| --- | --- | --- |
| 1 | 5 | 45.5% |
| 4 | 2 | 18.2% |
| 7 | 1 | 9.1% |
| 8 | 1 | 9.1% |
| 3 | 1 | 9.1% |
| 2 | 1 | 9.1% |

##### *Lowercase Letter*

| Value | Count | Frequency (%) |
| --- | --- | --- |
| a | 8 | 33.3% |
| e | 4 | 16.7% |
| n | 4 | 16.7% |
| o | 4 | 16.7% |
| s | 4 | 16.7% |

##### *Space Separator*

| Value | Count | Frequency (%) |
| --- | --- | --- |
|  | 16 | 100.0% |

##### *Uppercase Letter*

| Value | Count | Frequency (%) |
| --- | --- | --- |
| D | 4 | 100.0% |

#### Most occurring scripts

| Value | Count | Frequency (%) |
| --- | --- | --- |
| Latin | 28 | 50.9% |
| Common | 27 | 49.1% |

#### Most frequent character per script

##### *Common*

| Value | Count | Frequency (%) |
| --- | --- | --- |
|  | 16 | 59.3% |
| 1 | 5 | 18.5% |
| 4 | 2 | 7.4% |
| 7 | 1 | 3.7% |
| 8 | 1 | 3.7% |
| 3 | 1 | 3.7% |
| 2 | 1 | 3.7% |

##### *Latin*

| Value | Count | Frequency (%) |
| --- | --- | --- |
| a | 8 | 28.6% |
| D | 4 | 14.3% |
| e | 4 | 14.3% |
| n | 4 | 14.3% |
| o | 4 | 14.3% |
| s | 4 | 14.3% |

#### Most occurring blocks

| Value | Count | Frequency (%) |
| --- | --- | --- |
| ASCII | 55 | 100.0% |

#### Most frequent character per block

##### *ASCII*

| Value | Count | Frequency (%) |
| --- | --- | --- |
|  | 16 | 29.1% |
| a | 8 | 14.5% |
| 1 | 5 | 9.1% |
| D | 4 | 7.3% |
| e | 4 | 7.3% |
| n | 4 | 7.3% |
| o | 4 | 7.3% |
| s | 4 | 7.3% |
| 4 | 2 | 3.6% |
| 7 | 1 | 1.8% |
| Other values (3) | 3 | 5.5% |

TIPOCUP  
Categorical

|  |  |
| --- | --- |
| Distinct | 2 |
| Distinct (%) | 50.0% |
| Missing | 0 |
| Missing (%) | 0.0% |
| Memory size | 64.0 B |

|  |  |
| --- | --- |
| Outra | 3 |
| Desempregado | 1 |

More details

- Overview
- Categories
- Words
- Characters

Length

|  |  |
| --- | --- |
| Max length | 12 |
| Median length | 5 |
| Mean length | 6.75 |
| Min length | 5 |

Characters and Unicode

|  |  |
| --- | --- |
| Total characters | 27 |
| Distinct characters | 13 |
| Distinct categories | 2 ? |
| Distinct scripts | 1 ? |
| Distinct blocks | 1 ? |

The Unicode Standard assigns character properties to each code point, which can be used to analyse textual variables.

Unique

|  |  |
| --- | --- |
| Unique | 1 ? |
| Unique (%) | 25.0% |

Sample

|  |  |
| --- | --- |
| 1st row | Desempregado |
| 2nd row | Outra |
| 3rd row | Outra |
| 4th row | Outra |

#### Common Values

| Value | Count | Frequency (%) |
| --- | --- | --- |
| Outra | 3 | 75.0% |
| Desempregado | 1 | 25.0% |

#### Length

xml version="1.0" encoding="utf-8" standalone="no"?2023-10-31T16:38:57.511708image/svg+xmlMatplotlib v3.6.0, https://matplotlib.org/ 

Histogram of lengths of the category

#### Common Values (Plot)

xml version="1.0" encoding="utf-8" standalone="no"?2023-10-31T16:38:57.674484image/svg+xmlMatplotlib v3.6.0, https://matplotlib.org/

| Value | Count | Frequency (%) |
| --- | --- | --- |
| outra | 3 | 75.0% |
| desempregado | 1 | 25.0% |

- Characters
- Categories
- Scripts
- Blocks

#### Most occurring characters

| Value | Count | Frequency (%) |
| --- | --- | --- |
| r | 4 | 14.8% |
| a | 4 | 14.8% |
| O | 3 | 11.1% |
| u | 3 | 11.1% |
| t | 3 | 11.1% |
| e | 3 | 11.1% |
| D | 1 | 3.7% |
| s | 1 | 3.7% |
| m | 1 | 3.7% |
| p | 1 | 3.7% |
| Other values (3) | 3 | 11.1% |

#### Most occurring categories

| Value | Count | Frequency (%) |
| --- | --- | --- |
| Lowercase Letter | 23 | 85.2% |
| Uppercase Letter | 4 | 14.8% |

#### Most frequent character per category

##### *Lowercase Letter*

| Value | Count | Frequency (%) |
| --- | --- | --- |
| r | 4 | 17.4% |
| a | 4 | 17.4% |
| u | 3 | 13.0% |
| t | 3 | 13.0% |
| e | 3 | 13.0% |
| s | 1 | 4.3% |
| m | 1 | 4.3% |
| p | 1 | 4.3% |
| g | 1 | 4.3% |
| d | 1 | 4.3% |

##### *Uppercase Letter*

| Value | Count | Frequency (%) |
| --- | --- | --- |
| O | 3 | 75.0% |
| D | 1 | 25.0% |

#### Most occurring scripts

| Value | Count | Frequency (%) |
| --- | --- | --- |
| Latin | 27 | 100.0% |

#### Most frequent character per script

##### *Latin*

| Value | Count | Frequency (%) |
| --- | --- | --- |
| r | 4 | 14.8% |
| a | 4 | 14.8% |
| O | 3 | 11.1% |
| u | 3 | 11.1% |
| t | 3 | 11.1% |
| e | 3 | 11.1% |
| D | 1 | 3.7% |
| s | 1 | 3.7% |
| m | 1 | 3.7% |
| p | 1 | 3.7% |
| Other values (3) | 3 | 11.1% |

#### Most occurring blocks

| Value | Count | Frequency (%) |
| --- | --- | --- |
| ASCII | 27 | 100.0% |

#### Most frequent character per block

##### *ASCII*

| Value | Count | Frequency (%) |
| --- | --- | --- |
| r | 4 | 14.8% |
| a | 4 | 14.8% |
| O | 3 | 11.1% |
| u | 3 | 11.1% |
| t | 3 | 11.1% |
| e | 3 | 11.1% |
| D | 1 | 3.7% |
| s | 1 | 3.7% |
| m | 1 | 3.7% |
| p | 1 | 3.7% |
| Other values (3) | 3 | 11.1% |

sitAtual  
Categorical

|  |  |
| --- | --- |
| Distinct | 1 |
| Distinct (%) | 25.0% |
| Missing | 0 |
| Missing (%) | 0.0% |
| Memory size | 64.0 B |

|  |  |
| --- | --- |
| Cura | 4 |

More details

- Overview
- Categories
- Words
- Characters

Length

|  |  |
| --- | --- |
| Max length | 4 |
| Median length | 4 |
| Mean length | 4 |
| Min length | 4 |

Characters and Unicode

|  |  |
| --- | --- |
| Total characters | 16 |
| Distinct characters | 4 |
| Distinct categories | 2 ? |
| Distinct scripts | 1 ? |
| Distinct blocks | 1 ? |

The Unicode Standard assigns character properties to each code point, which can be used to analyse textual variables.

Unique

|  |  |
| --- | --- |
| Unique | 0 ? |
| Unique (%) | 0.0% |

Sample

|  |  |
| --- | --- |
| 1st row | Cura |
| 2nd row | Cura |
| 3rd row | Cura |
| 4th row | Cura |

#### Common Values

| Value | Count | Frequency (%) |
| --- | --- | --- |
| Cura | 4 | 100.0% |

#### Length

xml version="1.0" encoding="utf-8" standalone="no"?2023-10-31T16:38:57.827949image/svg+xmlMatplotlib v3.6.0, https://matplotlib.org/ 

Histogram of lengths of the category

#### Common Values (Plot)

xml version="1.0" encoding="utf-8" standalone="no"?2023-10-31T16:38:57.978283image/svg+xmlMatplotlib v3.6.0, https://matplotlib.org/

| Value | Count | Frequency (%) |
| --- | --- | --- |
| cura | 4 | 100.0% |

- Characters
- Categories
- Scripts
- Blocks

#### Most occurring characters

| Value | Count | Frequency (%) |
| --- | --- | --- |
| C | 4 | 25.0% |
| u | 4 | 25.0% |
| r | 4 | 25.0% |
| a | 4 | 25.0% |

#### Most occurring categories

| Value | Count | Frequency (%) |
| --- | --- | --- |
| Lowercase Letter | 12 | 75.0% |
| Uppercase Letter | 4 | 25.0% |

#### Most frequent character per category

##### *Lowercase Letter*

| Value | Count | Frequency (%) |
| --- | --- | --- |
| u | 4 | 33.3% |
| r | 4 | 33.3% |
| a | 4 | 33.3% |

##### *Uppercase Letter*

| Value | Count | Frequency (%) |
| --- | --- | --- |
| C | 4 | 100.0% |

#### Most occurring scripts

| Value | Count | Frequency (%) |
| --- | --- | --- |
| Latin | 16 | 100.0% |

#### Most frequent character per script

##### *Latin*

| Value | Count | Frequency (%) |
| --- | --- | --- |
| C | 4 | 25.0% |
| u | 4 | 25.0% |
| r | 4 | 25.0% |
| a | 4 | 25.0% |

#### Most occurring blocks

| Value | Count | Frequency (%) |
| --- | --- | --- |
| ASCII | 16 | 100.0% |

#### Most frequent character per block

##### *ASCII*

| Value | Count | Frequency (%) |
| --- | --- | --- |
| C | 4 | 25.0% |
| u | 4 | 25.0% |
| r | 4 | 25.0% |
| a | 4 | 25.0% |

tipoCaso  
Categorical

|  |  |
| --- | --- |
| Distinct | 2 |
| Distinct (%) | 50.0% |
| Missing | 0 |
| Missing (%) | 0.0% |
| Memory size | 64.0 B |

|  |  |
| --- | --- |
| Novo | 3 |
| Recidiva | 1 |

More details

- Overview
- Categories
- Words
- Characters

Length

|  |  |
| --- | --- |
| Max length | 8 |
| Median length | 4 |
| Mean length | 5 |
| Min length | 4 |

Characters and Unicode

|  |  |
| --- | --- |
| Total characters | 20 |
| Distinct characters | 9 |
| Distinct categories | 2 ? |
| Distinct scripts | 1 ? |
| Distinct blocks | 1 ? |

The Unicode Standard assigns character properties to each code point, which can be used to analyse textual variables.

Unique

|  |  |
| --- | --- |
| Unique | 1 ? |
| Unique (%) | 25.0% |

Sample

|  |  |
| --- | --- |
| 1st row | Novo |
| 2nd row | Novo |
| 3rd row | Novo |
| 4th row | Recidiva |

#### Common Values

| Value | Count | Frequency (%) |
| --- | --- | --- |
| Novo | 3 | 75.0% |
| Recidiva | 1 | 25.0% |

#### Length

xml version="1.0" encoding="utf-8" standalone="no"?2023-10-31T16:38:58.104156image/svg+xmlMatplotlib v3.6.0, https://matplotlib.org/ 

Histogram of lengths of the category

#### Common Values (Plot)

xml version="1.0" encoding="utf-8" standalone="no"?2023-10-31T16:38:58.266200image/svg+xmlMatplotlib v3.6.0, https://matplotlib.org/

| Value | Count | Frequency (%) |
| --- | --- | --- |
| novo | 3 | 75.0% |
| recidiva | 1 | 25.0% |

- Characters
- Categories
- Scripts
- Blocks

#### Most occurring characters

| Value | Count | Frequency (%) |
| --- | --- | --- |
| o | 6 | 30.0% |
| v | 4 | 20.0% |
| N | 3 | 15.0% |
| i | 2 | 10.0% |
| R | 1 | 5.0% |
| e | 1 | 5.0% |
| c | 1 | 5.0% |
| d | 1 | 5.0% |
| a | 1 | 5.0% |

#### Most occurring categories

| Value | Count | Frequency (%) |
| --- | --- | --- |
| Lowercase Letter | 16 | 80.0% |
| Uppercase Letter | 4 | 20.0% |

#### Most frequent character per category

##### *Lowercase Letter*

| Value | Count | Frequency (%) |
| --- | --- | --- |
| o | 6 | 37.5% |
| v | 4 | 25.0% |
| i | 2 | 12.5% |
| e | 1 | 6.2% |
| c | 1 | 6.2% |
| d | 1 | 6.2% |
| a | 1 | 6.2% |

##### *Uppercase Letter*

| Value | Count | Frequency (%) |
| --- | --- | --- |
| N | 3 | 75.0% |
| R | 1 | 25.0% |

#### Most occurring scripts

| Value | Count | Frequency (%) |
| --- | --- | --- |
| Latin | 20 | 100.0% |

#### Most frequent character per script

##### *Latin*

| Value | Count | Frequency (%) |
| --- | --- | --- |
| o | 6 | 30.0% |
| v | 4 | 20.0% |
| N | 3 | 15.0% |
| i | 2 | 10.0% |
| R | 1 | 5.0% |
| e | 1 | 5.0% |
| c | 1 | 5.0% |
| d | 1 | 5.0% |
| a | 1 | 5.0% |

#### Most occurring blocks

| Value | Count | Frequency (%) |
| --- | --- | --- |
| ASCII | 20 | 100.0% |

#### Most frequent character per block

##### *ASCII*

| Value | Count | Frequency (%) |
| --- | --- | --- |
| o | 6 | 30.0% |
| v | 4 | 20.0% |
| N | 3 | 15.0% |
| i | 2 | 10.0% |
| R | 1 | 5.0% |
| e | 1 | 5.0% |
| c | 1 | 5.0% |
| d | 1 | 5.0% |
| a | 1 | 5.0% |

FORMACLIN1  
Categorical

|  |  |
| --- | --- |
| Distinct | 1 |
| Distinct (%) | 25.0% |
| Missing | 0 |
| Missing (%) | 0.0% |
| Memory size | 64.0 B |

|  |  |
| --- | --- |
| Pul | 4 |

More details

- Overview
- Categories
- Words
- Characters

Length

|  |  |
| --- | --- |
| Max length | 3 |
| Median length | 3 |
| Mean length | 3 |
| Min length | 3 |

Characters and Unicode

|  |  |
| --- | --- |
| Total characters | 12 |
| Distinct characters | 3 |
| Distinct categories | 2 ? |
| Distinct scripts | 1 ? |
| Distinct blocks | 1 ? |

The Unicode Standard assigns character properties to each code point, which can be used to analyse textual variables.

Unique

|  |  |
| --- | --- |
| Unique | 0 ? |
| Unique (%) | 0.0% |

Sample

|  |  |
| --- | --- |
| 1st row | Pul |
| 2nd row | Pul |
| 3rd row | Pul |
| 4th row | Pul |

#### Common Values

| Value | Count | Frequency (%) |
| --- | --- | --- |
| Pul | 4 | 100.0% |

#### Length

xml version="1.0" encoding="utf-8" standalone="no"?2023-10-31T16:38:58.379493image/svg+xmlMatplotlib v3.6.0, https://matplotlib.org/ 

Histogram of lengths of the category

#### Common Values (Plot)

xml version="1.0" encoding="utf-8" standalone="no"?2023-10-31T16:38:58.513684image/svg+xmlMatplotlib v3.6.0, https://matplotlib.org/

| Value | Count | Frequency (%) |
| --- | --- | --- |
| pul | 4 | 100.0% |

- Characters
- Categories
- Scripts
- Blocks

#### Most occurring characters

| Value | Count | Frequency (%) |
| --- | --- | --- |
| P | 4 | 33.3% |
| u | 4 | 33.3% |
| l | 4 | 33.3% |

#### Most occurring categories

| Value | Count | Frequency (%) |
| --- | --- | --- |
| Lowercase Letter | 8 | 66.7% |
| Uppercase Letter | 4 | 33.3% |

#### Most frequent character per category

##### *Lowercase Letter*

| Value | Count | Frequency (%) |
| --- | --- | --- |
| u | 4 | 50.0% |
| l | 4 | 50.0% |

##### *Uppercase Letter*

| Value | Count | Frequency (%) |
| --- | --- | --- |
| P | 4 | 100.0% |

#### Most occurring scripts

| Value | Count | Frequency (%) |
| --- | --- | --- |
| Latin | 12 | 100.0% |

#### Most frequent character per script

##### *Latin*

| Value | Count | Frequency (%) |
| --- | --- | --- |
| P | 4 | 33.3% |
| u | 4 | 33.3% |
| l | 4 | 33.3% |

#### Most occurring blocks

| Value | Count | Frequency (%) |
| --- | --- | --- |
| ASCII | 12 | 100.0% |

#### Most frequent character per block

##### *ASCII*

| Value | Count | Frequency (%) |
| --- | --- | --- |
| P | 4 | 33.3% |
| u | 4 | 33.3% |
| l | 4 | 33.3% |

classif  
Categorical

|  |  |
| --- | --- |
| Distinct | 1 |
| Distinct (%) | 25.0% |
| Missing | 0 |
| Missing (%) | 0.0% |
| Memory size | 64.0 B |

|  |  |
| --- | --- |
| Pul | 4 |

More details

- Overview
- Categories
- Words
- Characters

Length

|  |  |
| --- | --- |
| Max length | 3 |
| Median length | 3 |
| Mean length | 3 |
| Min length | 3 |

Characters and Unicode

|  |  |
| --- | --- |
| Total characters | 12 |
| Distinct characters | 3 |
| Distinct categories | 2 ? |
| Distinct scripts | 1 ? |
| Distinct blocks | 1 ? |

The Unicode Standard assigns character properties to each code point, which can be used to analyse textual variables.

Unique

|  |  |
| --- | --- |
| Unique | 0 ? |
| Unique (%) | 0.0% |

Sample

|  |  |
| --- | --- |
| 1st row | Pul |
| 2nd row | Pul |
| 3rd row | Pul |
| 4th row | Pul |

#### Common Values

| Value | Count | Frequency (%) |
| --- | --- | --- |
| Pul | 4 | 100.0% |

#### Length

xml version="1.0" encoding="utf-8" standalone="no"?2023-10-31T16:38:58.620394image/svg+xmlMatplotlib v3.6.0, https://matplotlib.org/ 

Histogram of lengths of the category

#### Common Values (Plot)

xml version="1.0" encoding="utf-8" standalone="no"?2023-10-31T16:38:58.755550image/svg+xmlMatplotlib v3.6.0, https://matplotlib.org/

| Value | Count | Frequency (%) |
| --- | --- | --- |
| pul | 4 | 100.0% |

- Characters
- Categories
- Scripts
- Blocks

#### Most occurring characters

| Value | Count | Frequency (%) |
| --- | --- | --- |
| P | 4 | 33.3% |
| u | 4 | 33.3% |
| l | 4 | 33.3% |

#### Most occurring categories

| Value | Count | Frequency (%) |
| --- | --- | --- |
| Lowercase Letter | 8 | 66.7% |
| Uppercase Letter | 4 | 33.3% |

#### Most frequent character per category

##### *Lowercase Letter*

| Value | Count | Frequency (%) |
| --- | --- | --- |
| u | 4 | 50.0% |
| l | 4 | 50.0% |

##### *Uppercase Letter*

| Value | Count | Frequency (%) |
| --- | --- | --- |
| P | 4 | 100.0% |

#### Most occurring scripts

| Value | Count | Frequency (%) |
| --- | --- | --- |
| Latin | 12 | 100.0% |

#### Most frequent character per script

##### *Latin*

| Value | Count | Frequency (%) |
| --- | --- | --- |
| P | 4 | 33.3% |
| u | 4 | 33.3% |
| l | 4 | 33.3% |

#### Most occurring blocks

| Value | Count | Frequency (%) |
| --- | --- | --- |
| ASCII | 12 | 100.0% |

#### Most frequent character per block

##### *ASCII*

| Value | Count | Frequency (%) |
| --- | --- | --- |
| P | 4 | 33.3% |
| u | 4 | 33.3% |
| l | 4 | 33.3% |

descoberta  
Categorical

|  |  |
| --- | --- |
| Distinct | 3 |
| Distinct (%) | 75.0% |
| Missing | 0 |
| Missing (%) | 0.0% |
| Memory size | 64.0 B |

|  |  |
| --- | --- |
| Urgencia / Emergencia | 2 |
| Elucidacao Diagn. em Internacao | 1 |
| Investigacao de Contatos | 1 |

More details

- Overview
- Categories
- Words
- Characters

Length

|  |  |
| --- | --- |
| Max length | 31 |
| Median length | 27.5 |
| Mean length | 24.25 |
| Min length | 21 |

Characters and Unicode

|  |  |
| --- | --- |
| Total characters | 97 |
| Distinct characters | 23 |
| Distinct categories | 4 ? |
| Distinct scripts | 2 ? |
| Distinct blocks | 1 ? |

The Unicode Standard assigns character properties to each code point, which can be used to analyse textual variables.

Unique

|  |  |
| --- | --- |
| Unique | 2 ? |
| Unique (%) | 50.0% |

Sample

|  |  |
| --- | --- |
| 1st row | Elucidacao Diagn. em Internacao |
| 2nd row | Urgencia / Emergencia |
| 3rd row | Investigacao de Contatos |
| 4th row | Urgencia / Emergencia |

#### Common Values

| Value | Count | Frequency (%) |
| --- | --- | --- |
| Urgencia / Emergencia | 2 | 50.0% |
| Elucidacao Diagn. em Internacao | 1 | 25.0% |
| Investigacao de Contatos | 1 | 25.0% |

#### Length

xml version="1.0" encoding="utf-8" standalone="no"?2023-10-31T16:38:58.865268image/svg+xmlMatplotlib v3.6.0, https://matplotlib.org/ 

Histogram of lengths of the category

#### Common Values (Plot)

xml version="1.0" encoding="utf-8" standalone="no"?2023-10-31T16:38:59.018678image/svg+xmlMatplotlib v3.6.0, https://matplotlib.org/

| Value | Count | Frequency (%) |
| --- | --- | --- |
| urgencia | 2 | 15.4% |
|  | 2 | 15.4% |
| emergencia | 2 | 15.4% |
| elucidacao | 1 | 7.7% |
| diagn | 1 | 7.7% |
| em | 1 | 7.7% |
| internacao | 1 | 7.7% |
| investigacao | 1 | 7.7% |
| de | 1 | 7.7% |
| contatos | 1 | 7.7% |

- Characters
- Categories
- Scripts
- Blocks

#### Most occurring characters

| Value | Count | Frequency (%) |
| --- | --- | --- |
| a | 12 | 12.4% |
| e | 10 | 10.3% |
| n | 9 | 9.3% |
|  | 9 | 9.3% |
| c | 8 | 8.2% |
| i | 7 | 7.2% |
| g | 6 | 6.2% |
| o | 5 | 5.2% |
| r | 5 | 5.2% |
| t | 4 | 4.1% |
| Other values (13) | 22 | 22.7% |

#### Most occurring categories

| Value | Count | Frequency (%) |
| --- | --- | --- |
| Lowercase Letter | 76 | 78.4% |
| Space Separator | 9 | 9.3% |
| Uppercase Letter | 9 | 9.3% |
| Other Punctuation | 3 | 3.1% |

#### Most frequent character per category

##### *Lowercase Letter*

| Value | Count | Frequency (%) |
| --- | --- | --- |
| a | 12 | 15.8% |
| e | 10 | 13.2% |
| n | 9 | 11.8% |
| c | 8 | 10.5% |
| i | 7 | 9.2% |
| g | 6 | 7.9% |
| o | 5 | 6.6% |
| r | 5 | 6.6% |
| t | 4 | 5.3% |
| m | 3 | 3.9% |
| Other values (5) | 7 | 9.2% |

##### *Uppercase Letter*

| Value | Count | Frequency (%) |
| --- | --- | --- |
| E | 3 | 33.3% |
| I | 2 | 22.2% |
| U | 2 | 22.2% |
| D | 1 | 11.1% |
| C | 1 | 11.1% |

##### *Other Punctuation*

| Value | Count | Frequency (%) |
| --- | --- | --- |
| / | 2 | 66.7% |
| . | 1 | 33.3% |

##### *Space Separator*

| Value | Count | Frequency (%) |
| --- | --- | --- |
|  | 9 | 100.0% |

#### Most occurring scripts

| Value | Count | Frequency (%) |
| --- | --- | --- |
| Latin | 85 | 87.6% |
| Common | 12 | 12.4% |

#### Most frequent character per script

##### *Latin*

| Value | Count | Frequency (%) |
| --- | --- | --- |
| a | 12 | 14.1% |
| e | 10 | 11.8% |
| n | 9 | 10.6% |
| c | 8 | 9.4% |
| i | 7 | 8.2% |
| g | 6 | 7.1% |
| o | 5 | 5.9% |
| r | 5 | 5.9% |
| t | 4 | 4.7% |
| m | 3 | 3.5% |
| Other values (10) | 16 | 18.8% |

##### *Common*

| Value | Count | Frequency (%) |
| --- | --- | --- |
|  | 9 | 75.0% |
| / | 2 | 16.7% |
| . | 1 | 8.3% |

#### Most occurring blocks

| Value | Count | Frequency (%) |
| --- | --- | --- |
| ASCII | 97 | 100.0% |

#### Most frequent character per block

##### *ASCII*

| Value | Count | Frequency (%) |
| --- | --- | --- |
| a | 12 | 12.4% |
| e | 10 | 10.3% |
| n | 9 | 9.3% |
|  | 9 | 9.3% |
| c | 8 | 8.2% |
| i | 7 | 7.2% |
| g | 6 | 6.2% |
| o | 5 | 5.2% |
| r | 5 | 5.2% |
| t | 4 | 4.1% |
| Other values (13) | 22 | 22.7% |

bac  
Categorical

|  |  |
| --- | --- |
| Distinct | 1 |
| Distinct (%) | 25.0% |
| Missing | 0 |
| Missing (%) | 0.0% |
| Memory size | 64.0 B |

|  |  |
| --- | --- |
| Pos | 4 |

More details

- Overview
- Categories
- Words
- Characters

Length

|  |  |
| --- | --- |
| Max length | 3 |
| Median length | 3 |
| Mean length | 3 |
| Min length | 3 |

Characters and Unicode

|  |  |
| --- | --- |
| Total characters | 12 |
| Distinct characters | 3 |
| Distinct categories | 2 ? |
| Distinct scripts | 1 ? |
| Distinct blocks | 1 ? |

The Unicode Standard assigns character properties to each code point, which can be used to analyse textual variables.

Unique

|  |  |
| --- | --- |
| Unique | 0 ? |
| Unique (%) | 0.0% |

Sample

|  |  |
| --- | --- |
| 1st row | Pos |
| 2nd row | Pos |
| 3rd row | Pos |
| 4th row | Pos |

#### Common Values

| Value | Count | Frequency (%) |
| --- | --- | --- |
| Pos | 4 | 100.0% |

#### Length

xml version="1.0" encoding="utf-8" standalone="no"?2023-10-31T16:38:59.145280image/svg+xmlMatplotlib v3.6.0, https://matplotlib.org/ 

Histogram of lengths of the category

#### Common Values (Plot)

xml version="1.0" encoding="utf-8" standalone="no"?2023-10-31T16:38:59.278097image/svg+xmlMatplotlib v3.6.0, https://matplotlib.org/

| Value | Count | Frequency (%) |
| --- | --- | --- |
| pos | 4 | 100.0% |

- Characters
- Categories
- Scripts
- Blocks

#### Most occurring characters

| Value | Count | Frequency (%) |
| --- | --- | --- |
| P | 4 | 33.3% |
| o | 4 | 33.3% |
| s | 4 | 33.3% |

#### Most occurring categories

| Value | Count | Frequency (%) |
| --- | --- | --- |
| Lowercase Letter | 8 | 66.7% |
| Uppercase Letter | 4 | 33.3% |

#### Most frequent character per category

##### *Lowercase Letter*

| Value | Count | Frequency (%) |
| --- | --- | --- |
| o | 4 | 50.0% |
| s | 4 | 50.0% |

##### *Uppercase Letter*

| Value | Count | Frequency (%) |
| --- | --- | --- |
| P | 4 | 100.0% |

#### Most occurring scripts

| Value | Count | Frequency (%) |
| --- | --- | --- |
| Latin | 12 | 100.0% |

#### Most frequent character per script

##### *Latin*

| Value | Count | Frequency (%) |
| --- | --- | --- |
| P | 4 | 33.3% |
| o | 4 | 33.3% |
| s | 4 | 33.3% |

#### Most occurring blocks

| Value | Count | Frequency (%) |
| --- | --- | --- |
| ASCII | 12 | 100.0% |

#### Most frequent character per block

##### *ASCII*

| Value | Count | Frequency (%) |
| --- | --- | --- |
| P | 4 | 33.3% |
| o | 4 | 33.3% |
| s | 4 | 33.3% |

BACOUTRO  
Categorical

|  |  |
| --- | --- |
| Distinct | 1 |
| Distinct (%) | 25.0% |
| Missing | 0 |
| Missing (%) | 0.0% |
| Memory size | 64.0 B |

|  |  |
| --- | --- |
| N/realiz | 4 |

More details

- Overview
- Categories
- Words
- Characters

Length

|  |  |
| --- | --- |
| Max length | 8 |
| Median length | 8 |
| Mean length | 8 |
| Min length | 8 |

Characters and Unicode

|  |  |
| --- | --- |
| Total characters | 32 |
| Distinct characters | 8 |
| Distinct categories | 3 ? |
| Distinct scripts | 2 ? |
| Distinct blocks | 1 ? |

The Unicode Standard assigns character properties to each code point, which can be used to analyse textual variables.

Unique

|  |  |
| --- | --- |
| Unique | 0 ? |
| Unique (%) | 0.0% |

Sample

|  |  |
| --- | --- |
| 1st row | N/realiz |
| 2nd row | N/realiz |
| 3rd row | N/realiz |
| 4th row | N/realiz |

#### Common Values

| Value | Count | Frequency (%) |
| --- | --- | --- |
| N/realiz | 4 | 100.0% |

#### Length

xml version="1.0" encoding="utf-8" standalone="no"?2023-10-31T16:38:59.386512image/svg+xmlMatplotlib v3.6.0, https://matplotlib.org/ 

Histogram of lengths of the category

#### Common Values (Plot)

xml version="1.0" encoding="utf-8" standalone="no"?2023-10-31T16:38:59.522830image/svg+xmlMatplotlib v3.6.0, https://matplotlib.org/

| Value | Count | Frequency (%) |
| --- | --- | --- |
| n/realiz | 4 | 100.0% |

- Characters
- Categories
- Scripts
- Blocks

#### Most occurring characters

| Value | Count | Frequency (%) |
| --- | --- | --- |
| N | 4 | 12.5% |
| / | 4 | 12.5% |
| r | 4 | 12.5% |
| e | 4 | 12.5% |
| a | 4 | 12.5% |
| l | 4 | 12.5% |
| i | 4 | 12.5% |
| z | 4 | 12.5% |

#### Most occurring categories

| Value | Count | Frequency (%) |
| --- | --- | --- |
| Lowercase Letter | 24 | 75.0% |
| Uppercase Letter | 4 | 12.5% |
| Other Punctuation | 4 | 12.5% |

#### Most frequent character per category

##### *Lowercase Letter*

| Value | Count | Frequency (%) |
| --- | --- | --- |
| r | 4 | 16.7% |
| e | 4 | 16.7% |
| a | 4 | 16.7% |
| l | 4 | 16.7% |
| i | 4 | 16.7% |
| z | 4 | 16.7% |

##### *Uppercase Letter*

| Value | Count | Frequency (%) |
| --- | --- | --- |
| N | 4 | 100.0% |

##### *Other Punctuation*

| Value | Count | Frequency (%) |
| --- | --- | --- |
| / | 4 | 100.0% |

#### Most occurring scripts

| Value | Count | Frequency (%) |
| --- | --- | --- |
| Latin | 28 | 87.5% |
| Common | 4 | 12.5% |

#### Most frequent character per script

##### *Latin*

| Value | Count | Frequency (%) |
| --- | --- | --- |
| N | 4 | 14.3% |
| r | 4 | 14.3% |
| e | 4 | 14.3% |
| a | 4 | 14.3% |
| l | 4 | 14.3% |
| i | 4 | 14.3% |
| z | 4 | 14.3% |

##### *Common*

| Value | Count | Frequency (%) |
| --- | --- | --- |
| / | 4 | 100.0% |

#### Most occurring blocks

| Value | Count | Frequency (%) |
| --- | --- | --- |
| ASCII | 32 | 100.0% |

#### Most frequent character per block

##### *ASCII*

| Value | Count | Frequency (%) |
| --- | --- | --- |
| N | 4 | 12.5% |
| / | 4 | 12.5% |
| r | 4 | 12.5% |
| e | 4 | 12.5% |
| a | 4 | 12.5% |
| l | 4 | 12.5% |
| i | 4 | 12.5% |
| z | 4 | 12.5% |

cultEsc  
Categorical

|  |  |
| --- | --- |
| Distinct | 2 |
| Distinct (%) | 50.0% |
| Missing | 0 |
| Missing (%) | 0.0% |
| Memory size | 64.0 B |

|  |  |
| --- | --- |
| N/realiz | 3 |
| Neg | 1 |

More details

- Overview
- Categories
- Words
- Characters

Length

|  |  |
| --- | --- |
| Max length | 8 |
| Median length | 8 |
| Mean length | 6.75 |
| Min length | 3 |

Characters and Unicode

|  |  |
| --- | --- |
| Total characters | 27 |
| Distinct characters | 9 |
| Distinct categories | 3 ? |
| Distinct scripts | 2 ? |
| Distinct blocks | 1 ? |

The Unicode Standard assigns character properties to each code point, which can be used to analyse textual variables.

Unique

|  |  |
| --- | --- |
| Unique | 1 ? |
| Unique (%) | 25.0% |

Sample

|  |  |
| --- | --- |
| 1st row | N/realiz |
| 2nd row | Neg |
| 3rd row | N/realiz |
| 4th row | N/realiz |

#### Common Values

| Value | Count | Frequency (%) |
| --- | --- | --- |
| N/realiz | 3 | 75.0% |
| Neg | 1 | 25.0% |

#### Length

xml version="1.0" encoding="utf-8" standalone="no"?2023-10-31T16:38:59.640189image/svg+xmlMatplotlib v3.6.0, https://matplotlib.org/ 

Histogram of lengths of the category

#### Common Values (Plot)

xml version="1.0" encoding="utf-8" standalone="no"?2023-10-31T16:38:59.787212image/svg+xmlMatplotlib v3.6.0, https://matplotlib.org/

| Value | Count | Frequency (%) |
| --- | --- | --- |
| n/realiz | 3 | 75.0% |
| neg | 1 | 25.0% |

- Characters
- Categories
- Scripts
- Blocks

#### Most occurring characters

| Value | Count | Frequency (%) |
| --- | --- | --- |
| N | 4 | 14.8% |
| e | 4 | 14.8% |
| / | 3 | 11.1% |
| r | 3 | 11.1% |
| a | 3 | 11.1% |
| l | 3 | 11.1% |
| i | 3 | 11.1% |
| z | 3 | 11.1% |
| g | 1 | 3.7% |

#### Most occurring categories

| Value | Count | Frequency (%) |
| --- | --- | --- |
| Lowercase Letter | 20 | 74.1% |
| Uppercase Letter | 4 | 14.8% |
| Other Punctuation | 3 | 11.1% |

#### Most frequent character per category

##### *Lowercase Letter*

| Value | Count | Frequency (%) |
| --- | --- | --- |
| e | 4 | 20.0% |
| r | 3 | 15.0% |
| a | 3 | 15.0% |
| l | 3 | 15.0% |
| i | 3 | 15.0% |
| z | 3 | 15.0% |
| g | 1 | 5.0% |

##### *Uppercase Letter*

| Value | Count | Frequency (%) |
| --- | --- | --- |
| N | 4 | 100.0% |

##### *Other Punctuation*

| Value | Count | Frequency (%) |
| --- | --- | --- |
| / | 3 | 100.0% |

#### Most occurring scripts

| Value | Count | Frequency (%) |
| --- | --- | --- |
| Latin | 24 | 88.9% |
| Common | 3 | 11.1% |

#### Most frequent character per script

##### *Latin*

| Value | Count | Frequency (%) |
| --- | --- | --- |
| N | 4 | 16.7% |
| e | 4 | 16.7% |
| r | 3 | 12.5% |
| a | 3 | 12.5% |
| l | 3 | 12.5% |
| i | 3 | 12.5% |
| z | 3 | 12.5% |
| g | 1 | 4.2% |

##### *Common*

| Value | Count | Frequency (%) |
| --- | --- | --- |
| / | 3 | 100.0% |

#### Most occurring blocks

| Value | Count | Frequency (%) |
| --- | --- | --- |
| ASCII | 27 | 100.0% |

#### Most frequent character per block

##### *ASCII*

| Value | Count | Frequency (%) |
| --- | --- | --- |
| N | 4 | 14.8% |
| e | 4 | 14.8% |
| / | 3 | 11.1% |
| r | 3 | 11.1% |
| a | 3 | 11.1% |
| l | 3 | 11.1% |
| i | 3 | 11.1% |
| z | 3 | 11.1% |
| g | 1 | 3.7% |

RX  
Categorical

|  |  |
| --- | --- |
| Distinct | 2 |
| Distinct (%) | 50.0% |
| Missing | 0 |
| Missing (%) | 0.0% |
| Memory size | 64.0 B |

|  |  |
| --- | --- |
| Susp TB | 3 |
| N/realiz | 1 |

More details

- Overview
- Categories
- Words
- Characters

Length

|  |  |
| --- | --- |
| Max length | 8 |
| Median length | 7 |
| Mean length | 7.25 |
| Min length | 7 |

Characters and Unicode

|  |  |
| --- | --- |
| Total characters | 29 |
| Distinct characters | 15 |
| Distinct categories | 4 ? |
| Distinct scripts | 2 ? |
| Distinct blocks | 1 ? |

The Unicode Standard assigns character properties to each code point, which can be used to analyse textual variables.

Unique

|  |  |
| --- | --- |
| Unique | 1 ? |
| Unique (%) | 25.0% |

Sample

|  |  |
| --- | --- |
| 1st row | Susp TB |
| 2nd row | Susp TB |
| 3rd row | N/realiz |
| 4th row | Susp TB |

#### Common Values

| Value | Count | Frequency (%) |
| --- | --- | --- |
| Susp TB | 3 | 75.0% |
| N/realiz | 1 | 25.0% |

#### Length

xml version="1.0" encoding="utf-8" standalone="no"?2023-10-31T16:38:59.911568image/svg+xmlMatplotlib v3.6.0, https://matplotlib.org/ 

Histogram of lengths of the category

#### Common Values (Plot)

xml version="1.0" encoding="utf-8" standalone="no"?2023-10-31T16:39:00.060916image/svg+xmlMatplotlib v3.6.0, https://matplotlib.org/

| Value | Count | Frequency (%) |
| --- | --- | --- |
| susp | 3 | 42.9% |
| tb | 3 | 42.9% |
| n/realiz | 1 | 14.3% |

- Characters
- Categories
- Scripts
- Blocks

#### Most occurring characters

| Value | Count | Frequency (%) |
| --- | --- | --- |
| S | 3 | 10.3% |
| u | 3 | 10.3% |
| s | 3 | 10.3% |
| p | 3 | 10.3% |
|  | 3 | 10.3% |
| T | 3 | 10.3% |
| B | 3 | 10.3% |
| N | 1 | 3.4% |
| / | 1 | 3.4% |
| r | 1 | 3.4% |
| Other values (5) | 5 | 17.2% |

#### Most occurring categories

| Value | Count | Frequency (%) |
| --- | --- | --- |
| Lowercase Letter | 15 | 51.7% |
| Uppercase Letter | 10 | 34.5% |
| Space Separator | 3 | 10.3% |
| Other Punctuation | 1 | 3.4% |

#### Most frequent character per category

##### *Lowercase Letter*

| Value | Count | Frequency (%) |
| --- | --- | --- |
| u | 3 | 20.0% |
| s | 3 | 20.0% |
| p | 3 | 20.0% |
| r | 1 | 6.7% |
| e | 1 | 6.7% |
| a | 1 | 6.7% |
| l | 1 | 6.7% |
| i | 1 | 6.7% |
| z | 1 | 6.7% |

##### *Uppercase Letter*

| Value | Count | Frequency (%) |
| --- | --- | --- |
| S | 3 | 30.0% |
| T | 3 | 30.0% |
| B | 3 | 30.0% |
| N | 1 | 10.0% |

##### *Space Separator*

| Value | Count | Frequency (%) |
| --- | --- | --- |
|  | 3 | 100.0% |

##### *Other Punctuation*

| Value | Count | Frequency (%) |
| --- | --- | --- |
| / | 1 | 100.0% |

#### Most occurring scripts

| Value | Count | Frequency (%) |
| --- | --- | --- |
| Latin | 25 | 86.2% |
| Common | 4 | 13.8% |

#### Most frequent character per script

##### *Latin*

| Value | Count | Frequency (%) |
| --- | --- | --- |
| S | 3 | 12.0% |
| u | 3 | 12.0% |
| s | 3 | 12.0% |
| p | 3 | 12.0% |
| T | 3 | 12.0% |
| B | 3 | 12.0% |
| N | 1 | 4.0% |
| r | 1 | 4.0% |
| e | 1 | 4.0% |
| a | 1 | 4.0% |
| Other values (3) | 3 | 12.0% |

##### *Common*

| Value | Count | Frequency (%) |
| --- | --- | --- |
|  | 3 | 75.0% |
| / | 1 | 25.0% |

#### Most occurring blocks

| Value | Count | Frequency (%) |
| --- | --- | --- |
| ASCII | 29 | 100.0% |

#### Most frequent character per block

##### *ASCII*

| Value | Count | Frequency (%) |
| --- | --- | --- |
| S | 3 | 10.3% |
| u | 3 | 10.3% |
| s | 3 | 10.3% |
| p | 3 | 10.3% |
|  | 3 | 10.3% |
| T | 3 | 10.3% |
| B | 3 | 10.3% |
| N | 1 | 3.4% |
| / | 1 | 3.4% |
| r | 1 | 3.4% |
| Other values (5) | 5 | 17.2% |

NECROP  
Categorical

|  |  |
| --- | --- |
| Distinct | 1 |
| Distinct (%) | 25.0% |
| Missing | 0 |
| Missing (%) | 0.0% |
| Memory size | 64.0 B |

|  |  |
| --- | --- |
| N/realiz | 4 |

More details

- Overview
- Categories
- Words
- Characters

Length

|  |  |
| --- | --- |
| Max length | 8 |
| Median length | 8 |
| Mean length | 8 |
| Min length | 8 |

Characters and Unicode

|  |  |
| --- | --- |
| Total characters | 32 |
| Distinct characters | 8 |
| Distinct categories | 3 ? |
| Distinct scripts | 2 ? |
| Distinct blocks | 1 ? |

The Unicode Standard assigns character properties to each code point, which can be used to analyse textual variables.

Unique

|  |  |
| --- | --- |
| Unique | 0 ? |
| Unique (%) | 0.0% |

Sample

|  |  |
| --- | --- |
| 1st row | N/realiz |
| 2nd row | N/realiz |
| 3rd row | N/realiz |
| 4th row | N/realiz |

#### Common Values

| Value | Count | Frequency (%) |
| --- | --- | --- |
| N/realiz | 4 | 100.0% |

#### Length

xml version="1.0" encoding="utf-8" standalone="no"?2023-10-31T16:39:00.178111image/svg+xmlMatplotlib v3.6.0, https://matplotlib.org/ 

Histogram of lengths of the category

#### Common Values (Plot)

xml version="1.0" encoding="utf-8" standalone="no"?2023-10-31T16:39:00.311564image/svg+xmlMatplotlib v3.6.0, https://matplotlib.org/

| Value | Count | Frequency (%) |
| --- | --- | --- |
| n/realiz | 4 | 100.0% |

- Characters
- Categories
- Scripts
- Blocks

#### Most occurring characters

| Value | Count | Frequency (%) |
| --- | --- | --- |
| N | 4 | 12.5% |
| / | 4 | 12.5% |
| r | 4 | 12.5% |
| e | 4 | 12.5% |
| a | 4 | 12.5% |
| l | 4 | 12.5% |
| i | 4 | 12.5% |
| z | 4 | 12.5% |

#### Most occurring categories

| Value | Count | Frequency (%) |
| --- | --- | --- |
| Lowercase Letter | 24 | 75.0% |
| Uppercase Letter | 4 | 12.5% |
| Other Punctuation | 4 | 12.5% |

#### Most frequent character per category

##### *Lowercase Letter*

| Value | Count | Frequency (%) |
| --- | --- | --- |
| r | 4 | 16.7% |
| e | 4 | 16.7% |
| a | 4 | 16.7% |
| l | 4 | 16.7% |
| i | 4 | 16.7% |
| z | 4 | 16.7% |

##### *Uppercase Letter*

| Value | Count | Frequency (%) |
| --- | --- | --- |
| N | 4 | 100.0% |

##### *Other Punctuation*

| Value | Count | Frequency (%) |
| --- | --- | --- |
| / | 4 | 100.0% |

#### Most occurring scripts

| Value | Count | Frequency (%) |
| --- | --- | --- |
| Latin | 28 | 87.5% |
| Common | 4 | 12.5% |

#### Most frequent character per script

##### *Latin*

| Value | Count | Frequency (%) |
| --- | --- | --- |
| N | 4 | 14.3% |
| r | 4 | 14.3% |
| e | 4 | 14.3% |
| a | 4 | 14.3% |
| l | 4 | 14.3% |
| i | 4 | 14.3% |
| z | 4 | 14.3% |

##### *Common*

| Value | Count | Frequency (%) |
| --- | --- | --- |
| / | 4 | 100.0% |

#### Most occurring blocks

| Value | Count | Frequency (%) |
| --- | --- | --- |
| ASCII | 32 | 100.0% |

#### Most frequent character per block

##### *ASCII*

| Value | Count | Frequency (%) |
| --- | --- | --- |
| N | 4 | 12.5% |
| / | 4 | 12.5% |
| r | 4 | 12.5% |
| e | 4 | 12.5% |
| a | 4 | 12.5% |
| l | 4 | 12.5% |
| i | 4 | 12.5% |
| z | 4 | 12.5% |

hiv  
Categorical

|  |  |
| --- | --- |
| Distinct | 1 |
| Distinct (%) | 25.0% |
| Missing | 0 |
| Missing (%) | 0.0% |
| Memory size | 64.0 B |

|  |  |
| --- | --- |
| Neg | 4 |

More details

- Overview
- Categories
- Words
- Characters

Length

|  |  |
| --- | --- |
| Max length | 3 |
| Median length | 3 |
| Mean length | 3 |
| Min length | 3 |

Characters and Unicode

|  |  |
| --- | --- |
| Total characters | 12 |
| Distinct characters | 3 |
| Distinct categories | 2 ? |
| Distinct scripts | 1 ? |
| Distinct blocks | 1 ? |

The Unicode Standard assigns character properties to each code point, which can be used to analyse textual variables.

Unique

|  |  |
| --- | --- |
| Unique | 0 ? |
| Unique (%) | 0.0% |

Sample

|  |  |
| --- | --- |
| 1st row | Neg |
| 2nd row | Neg |
| 3rd row | Neg |
| 4th row | Neg |

#### Common Values

| Value | Count | Frequency (%) |
| --- | --- | --- |
| Neg | 4 | 100.0% |

#### Length

xml version="1.0" encoding="utf-8" standalone="no"?2023-10-31T16:39:00.419205image/svg+xmlMatplotlib v3.6.0, https://matplotlib.org/ 

Histogram of lengths of the category

#### Common Values (Plot)

xml version="1.0" encoding="utf-8" standalone="no"?2023-10-31T16:39:00.553017image/svg+xmlMatplotlib v3.6.0, https://matplotlib.org/

| Value | Count | Frequency (%) |
| --- | --- | --- |
| neg | 4 | 100.0% |

- Characters
- Categories
- Scripts
- Blocks

#### Most occurring characters

| Value | Count | Frequency (%) |
| --- | --- | --- |
| N | 4 | 33.3% |
| e | 4 | 33.3% |
| g | 4 | 33.3% |

#### Most occurring categories

| Value | Count | Frequency (%) |
| --- | --- | --- |
| Lowercase Letter | 8 | 66.7% |
| Uppercase Letter | 4 | 33.3% |

#### Most frequent character per category

##### *Lowercase Letter*

| Value | Count | Frequency (%) |
| --- | --- | --- |
| e | 4 | 50.0% |
| g | 4 | 50.0% |

##### *Uppercase Letter*

| Value | Count | Frequency (%) |
| --- | --- | --- |
| N | 4 | 100.0% |

#### Most occurring scripts

| Value | Count | Frequency (%) |
| --- | --- | --- |
| Latin | 12 | 100.0% |

#### Most frequent character per script

##### *Latin*

| Value | Count | Frequency (%) |
| --- | --- | --- |
| N | 4 | 33.3% |
| e | 4 | 33.3% |
| g | 4 | 33.3% |

#### Most occurring blocks

| Value | Count | Frequency (%) |
| --- | --- | --- |
| ASCII | 12 | 100.0% |

#### Most frequent character per block

##### *ASCII*

| Value | Count | Frequency (%) |
| --- | --- | --- |
| N | 4 | 33.3% |
| e | 4 | 33.3% |
| g | 4 | 33.3% |

aids  
Boolean

|  |  |
| --- | --- |
| Distinct | 1 |
| Distinct (%) | 25.0% |
| Missing | 0 |
| Missing (%) | 0.0% |
| Memory size | 36.0 B |

|  |  |
| --- | --- |
| False | 4 |

More details

- Common Values (Table)
- Common Values (Plot)

| Value | Count | Frequency (%) |
| --- | --- | --- |
| False | 4 | 100.0% |

xml version="1.0" encoding="utf-8" standalone="no"?2023-10-31T16:39:00.674445image/svg+xmlMatplotlib v3.6.0, https://matplotlib.org/

DIABETES  
Boolean

|  |  |
| --- | --- |
| Distinct | 1 |
| Distinct (%) | 25.0% |
| Missing | 0 |
| Missing (%) | 0.0% |
| Memory size | 36.0 B |

|  |  |
| --- | --- |
| False | 4 |

More details

- Common Values (Table)
- Common Values (Plot)

| Value | Count | Frequency (%) |
| --- | --- | --- |
| False | 4 | 100.0% |

xml version="1.0" encoding="utf-8" standalone="no"?2023-10-31T16:39:00.795715image/svg+xmlMatplotlib v3.6.0, https://matplotlib.org/

ALCOOLISMO  
Boolean

|  |  |
| --- | --- |
| Distinct | 1 |
| Distinct (%) | 25.0% |
| Missing | 0 |
| Missing (%) | 0.0% |
| Memory size | 36.0 B |

|  |  |
| --- | --- |
| False | 4 |

More details

- Common Values (Table)
- Common Values (Plot)

| Value | Count | Frequency (%) |
| --- | --- | --- |
| False | 4 | 100.0% |

xml version="1.0" encoding="utf-8" standalone="no"?2023-10-31T16:39:00.915744image/svg+xmlMatplotlib v3.6.0, https://matplotlib.org/

MENTAL  
Boolean

|  |  |
| --- | --- |
| Distinct | 1 |
| Distinct (%) | 25.0% |
| Missing | 0 |
| Missing (%) | 0.0% |
| Memory size | 36.0 B |

|  |  |
| --- | --- |
| False | 4 |

More details

- Common Values (Table)
- Common Values (Plot)

| Value | Count | Frequency (%) |
| --- | --- | --- |
| False | 4 | 100.0% |

xml version="1.0" encoding="utf-8" standalone="no"?2023-10-31T16:39:01.037663image/svg+xmlMatplotlib v3.6.0, https://matplotlib.org/

DROGADICAO  
Categorical

|  |  |
| --- | --- |
| Distinct | 2 |
| Distinct (%) | 50.0% |
| Missing | 0 |
| Missing (%) | 0.0% |
| Memory size | 64.0 B |

|  |  |
| --- | --- |
| N | 3 |
| S | 1 |

More details

- Overview
- Categories
- Words
- Characters

Length

|  |  |
| --- | --- |
| Max length | 1 |
| Median length | 1 |
| Mean length | 1 |
| Min length | 1 |

Characters and Unicode

|  |  |
| --- | --- |
| Total characters | 4 |
| Distinct characters | 2 |
| Distinct categories | 1 ? |
| Distinct scripts | 1 ? |
| Distinct blocks | 1 ? |

The Unicode Standard assigns character properties to each code point, which can be used to analyse textual variables.

Unique

|  |  |
| --- | --- |
| Unique | 1 ? |
| Unique (%) | 25.0% |

Sample

|  |  |
| --- | --- |
| 1st row | S |
| 2nd row | N |
| 3rd row | N |
| 4th row | N |

#### Common Values

| Value | Count | Frequency (%) |
| --- | --- | --- |
| N | 3 | 75.0% |
| S | 1 | 25.0% |

#### Length

xml version="1.0" encoding="utf-8" standalone="no"?2023-10-31T16:39:01.145799image/svg+xmlMatplotlib v3.6.0, https://matplotlib.org/ 

Histogram of lengths of the category

#### Common Values (Plot)

xml version="1.0" encoding="utf-8" standalone="no"?2023-10-31T16:39:01.285105image/svg+xmlMatplotlib v3.6.0, https://matplotlib.org/

| Value | Count | Frequency (%) |
| --- | --- | --- |
| n | 3 | 75.0% |
| s | 1 | 25.0% |

- Characters
- Categories
- Scripts
- Blocks

#### Most occurring characters

| Value | Count | Frequency (%) |
| --- | --- | --- |
| N | 3 | 75.0% |
| S | 1 | 25.0% |

#### Most occurring categories

| Value | Count | Frequency (%) |
| --- | --- | --- |
| Uppercase Letter | 4 | 100.0% |

#### Most frequent character per category

##### *Uppercase Letter*

| Value | Count | Frequency (%) |
| --- | --- | --- |
| N | 3 | 75.0% |
| S | 1 | 25.0% |

#### Most occurring scripts

| Value | Count | Frequency (%) |
| --- | --- | --- |
| Latin | 4 | 100.0% |

#### Most frequent character per script

##### *Latin*

| Value | Count | Frequency (%) |
| --- | --- | --- |
| N | 3 | 75.0% |
| S | 1 | 25.0% |

#### Most occurring blocks

| Value | Count | Frequency (%) |
| --- | --- | --- |
| ASCII | 4 | 100.0% |

#### Most frequent character per block

##### *ASCII*

| Value | Count | Frequency (%) |
| --- | --- | --- |
| N | 3 | 75.0% |
| S | 1 | 25.0% |

TABAGISMO  
Boolean

|  |  |
| --- | --- |
| Distinct | 1 |
| Distinct (%) | 25.0% |
| Missing | 0 |
| Missing (%) | 0.0% |
| Memory size | 36.0 B |

|  |  |
| --- | --- |
| False | 4 |

More details

- Common Values (Table)
- Common Values (Plot)

| Value | Count | Frequency (%) |
| --- | --- | --- |
| False | 4 | 100.0% |

xml version="1.0" encoding="utf-8" standalone="no"?2023-10-31T16:39:01.414276image/svg+xmlMatplotlib v3.6.0, https://matplotlib.org/

motMudEsquema  
Categorical

|  |  |
| --- | --- |
| Distinct | 2 |
| Distinct (%) | 50.0% |
| Missing | 0 |
| Missing (%) | 0.0% |
| Memory size | 64.0 B |

|  |  |
| --- | --- |
| Nulo | 3 |
| Intolerancia/Toxicidade | 1 |

More details

- Overview
- Categories
- Words
- Characters

Length

|  |  |
| --- | --- |
| Max length | 23 |
| Median length | 4 |
| Mean length | 8.75 |
| Min length | 4 |

Characters and Unicode

|  |  |
| --- | --- |
| Total characters | 35 |
| Distinct characters | 16 |
| Distinct categories | 3 ? |
| Distinct scripts | 2 ? |
| Distinct blocks | 1 ? |

The Unicode Standard assigns character properties to each code point, which can be used to analyse textual variables.

Unique

|  |  |
| --- | --- |
| Unique | 1 ? |
| Unique (%) | 25.0% |

Sample

|  |  |
| --- | --- |
| 1st row | Nulo |
| 2nd row | Nulo |
| 3rd row | Intolerancia/Toxicidade |
| 4th row | Nulo |

#### Common Values

| Value | Count | Frequency (%) |
| --- | --- | --- |
| Nulo | 3 | 75.0% |
| Intolerancia/Toxicidade | 1 | 25.0% |

#### Length

xml version="1.0" encoding="utf-8" standalone="no"?2023-10-31T16:39:01.541220image/svg+xmlMatplotlib v3.6.0, https://matplotlib.org/ 

Histogram of lengths of the category

#### Common Values (Plot)

xml version="1.0" encoding="utf-8" standalone="no"?2023-10-31T16:39:01.702532image/svg+xmlMatplotlib v3.6.0, https://matplotlib.org/

| Value | Count | Frequency (%) |
| --- | --- | --- |
| nulo | 3 | 75.0% |
| intolerancia/toxicidade | 1 | 25.0% |

- Characters
- Categories
- Scripts
- Blocks

#### Most occurring characters

| Value | Count | Frequency (%) |
| --- | --- | --- |
| o | 5 | 14.3% |
| l | 4 | 11.4% |
| N | 3 | 8.6% |
| u | 3 | 8.6% |
| a | 3 | 8.6% |
| i | 3 | 8.6% |
| n | 2 | 5.7% |
| e | 2 | 5.7% |
| c | 2 | 5.7% |
| d | 2 | 5.7% |
| Other values (6) | 6 | 17.1% |

#### Most occurring categories

| Value | Count | Frequency (%) |
| --- | --- | --- |
| Lowercase Letter | 29 | 82.9% |
| Uppercase Letter | 5 | 14.3% |
| Other Punctuation | 1 | 2.9% |

#### Most frequent character per category

##### *Lowercase Letter*

| Value | Count | Frequency (%) |
| --- | --- | --- |
| o | 5 | 17.2% |
| l | 4 | 13.8% |
| u | 3 | 10.3% |
| a | 3 | 10.3% |
| i | 3 | 10.3% |
| n | 2 | 6.9% |
| e | 2 | 6.9% |
| c | 2 | 6.9% |
| d | 2 | 6.9% |
| t | 1 | 3.4% |
| Other values (2) | 2 | 6.9% |

##### *Uppercase Letter*

| Value | Count | Frequency (%) |
| --- | --- | --- |
| N | 3 | 60.0% |
| I | 1 | 20.0% |
| T | 1 | 20.0% |

##### *Other Punctuation*

| Value | Count | Frequency (%) |
| --- | --- | --- |
| / | 1 | 100.0% |

#### Most occurring scripts

| Value | Count | Frequency (%) |
| --- | --- | --- |
| Latin | 34 | 97.1% |
| Common | 1 | 2.9% |

#### Most frequent character per script

##### *Latin*

| Value | Count | Frequency (%) |
| --- | --- | --- |
| o | 5 | 14.7% |
| l | 4 | 11.8% |
| N | 3 | 8.8% |
| u | 3 | 8.8% |
| a | 3 | 8.8% |
| i | 3 | 8.8% |
| n | 2 | 5.9% |
| e | 2 | 5.9% |
| c | 2 | 5.9% |
| d | 2 | 5.9% |
| Other values (5) | 5 | 14.7% |

##### *Common*

| Value | Count | Frequency (%) |
| --- | --- | --- |
| / | 1 | 100.0% |

#### Most occurring blocks

| Value | Count | Frequency (%) |
| --- | --- | --- |
| ASCII | 35 | 100.0% |

#### Most frequent character per block

##### *ASCII*

| Value | Count | Frequency (%) |
| --- | --- | --- |
| o | 5 | 14.3% |
| l | 4 | 11.4% |
| N | 3 | 8.6% |
| u | 3 | 8.6% |
| a | 3 | 8.6% |
| i | 3 | 8.6% |
| n | 2 | 5.7% |
| e | 2 | 5.7% |
| c | 2 | 5.7% |
| d | 2 | 5.7% |
| Other values (6) | 6 | 17.1% |

tipoTrat  
Categorical

|  |  |
| --- | --- |
| Distinct | 1 |
| Distinct (%) | 25.0% |
| Missing | 0 |
| Missing (%) | 0.0% |
| Memory size | 64.0 B |

|  |  |
| --- | --- |
| Supervisionado | 4 |

More details

- Overview
- Categories
- Words
- Characters

Length

|  |  |
| --- | --- |
| Max length | 14 |
| Median length | 14 |
| Mean length | 14 |
| Min length | 14 |

Characters and Unicode

|  |  |
| --- | --- |
| Total characters | 56 |
| Distinct characters | 12 |
| Distinct categories | 2 ? |
| Distinct scripts | 1 ? |
| Distinct blocks | 1 ? |

The Unicode Standard assigns character properties to each code point, which can be used to analyse textual variables.

Unique

|  |  |
| --- | --- |
| Unique | 0 ? |
| Unique (%) | 0.0% |

Sample

|  |  |
| --- | --- |
| 1st row | Supervisionado |
| 2nd row | Supervisionado |
| 3rd row | Supervisionado |
| 4th row | Supervisionado |

#### Common Values

| Value | Count | Frequency (%) |
| --- | --- | --- |
| Supervisionado | 4 | 100.0% |

#### Length

xml version="1.0" encoding="utf-8" standalone="no"?2023-10-31T16:39:01.820823image/svg+xmlMatplotlib v3.6.0, https://matplotlib.org/ 

Histogram of lengths of the category

#### Common Values (Plot)

xml version="1.0" encoding="utf-8" standalone="no"?2023-10-31T16:39:01.956427image/svg+xmlMatplotlib v3.6.0, https://matplotlib.org/

| Value | Count | Frequency (%) |
| --- | --- | --- |
| supervisionado | 4 | 100.0% |

- Characters
- Categories
- Scripts
- Blocks

#### Most occurring characters

| Value | Count | Frequency (%) |
| --- | --- | --- |
| i | 8 | 14.3% |
| o | 8 | 14.3% |
| S | 4 | 7.1% |
| u | 4 | 7.1% |
| p | 4 | 7.1% |
| e | 4 | 7.1% |
| r | 4 | 7.1% |
| v | 4 | 7.1% |
| s | 4 | 7.1% |
| n | 4 | 7.1% |
| Other values (2) | 8 | 14.3% |

#### Most occurring categories

| Value | Count | Frequency (%) |
| --- | --- | --- |
| Lowercase Letter | 52 | 92.9% |
| Uppercase Letter | 4 | 7.1% |

#### Most frequent character per category

##### *Lowercase Letter*

| Value | Count | Frequency (%) |
| --- | --- | --- |
| i | 8 | 15.4% |
| o | 8 | 15.4% |
| u | 4 | 7.7% |
| p | 4 | 7.7% |
| e | 4 | 7.7% |
| r | 4 | 7.7% |
| v | 4 | 7.7% |
| s | 4 | 7.7% |
| n | 4 | 7.7% |
| a | 4 | 7.7% |

##### *Uppercase Letter*

| Value | Count | Frequency (%) |
| --- | --- | --- |
| S | 4 | 100.0% |

#### Most occurring scripts

| Value | Count | Frequency (%) |
| --- | --- | --- |
| Latin | 56 | 100.0% |

#### Most frequent character per script

##### *Latin*

| Value | Count | Frequency (%) |
| --- | --- | --- |
| i | 8 | 14.3% |
| o | 8 | 14.3% |
| S | 4 | 7.1% |
| u | 4 | 7.1% |
| p | 4 | 7.1% |
| e | 4 | 7.1% |
| r | 4 | 7.1% |
| v | 4 | 7.1% |
| s | 4 | 7.1% |
| n | 4 | 7.1% |
| Other values (2) | 8 | 14.3% |

#### Most occurring blocks

| Value | Count | Frequency (%) |
| --- | --- | --- |
| ASCII | 56 | 100.0% |

#### Most frequent character per block

##### *ASCII*

| Value | Count | Frequency (%) |
| --- | --- | --- |
| i | 8 | 14.3% |
| o | 8 | 14.3% |
| S | 4 | 7.1% |
| u | 4 | 7.1% |
| p | 4 | 7.1% |
| e | 4 | 7.1% |
| r | 4 | 7.1% |
| v | 4 | 7.1% |
| s | 4 | 7.1% |
| n | 4 | 7.1% |
| Other values (2) | 8 | 14.3% |

idade  
Categorical

|  |  |
| --- | --- |
| Distinct | 3 |
| Distinct (%) | 75.0% |
| Missing | 0 |
| Missing (%) | 0.0% |
| Memory size | 64.0 B |

|  |  |
| --- | --- |
| 40\_54 | 2 |
| 23\_39 | 1 |
| Mais de 54 | 1 |

More details

- Overview
- Categories
- Words
- Characters

Length

|  |  |
| --- | --- |
| Max length | 10 |
| Median length | 5 |
| Mean length | 6.25 |
| Min length | 5 |

Characters and Unicode

|  |  |
| --- | --- |
| Total characters | 25 |
| Distinct characters | 14 |
| Distinct categories | 5 ? |
| Distinct scripts | 2 ? |
| Distinct blocks | 1 ? |

The Unicode Standard assigns character properties to each code point, which can be used to analyse textual variables.

Unique

|  |  |
| --- | --- |
| Unique | 2 ? |
| Unique (%) | 50.0% |

Sample

|  |  |
| --- | --- |
| 1st row | 23\_39 |
| 2nd row | 40\_54 |
| 3rd row | 40\_54 |
| 4th row | Mais de 54 |

#### Common Values

| Value | Count | Frequency (%) |
| --- | --- | --- |
| 40\_54 | 2 | 50.0% |
| 23\_39 | 1 | 25.0% |
| Mais de 54 | 1 | 25.0% |

#### Length

xml version="1.0" encoding="utf-8" standalone="no"?2023-10-31T16:39:02.072318image/svg+xmlMatplotlib v3.6.0, https://matplotlib.org/ 

Histogram of lengths of the category

#### Common Values (Plot)

xml version="1.0" encoding="utf-8" standalone="no"?2023-10-31T16:39:02.227623image/svg+xmlMatplotlib v3.6.0, https://matplotlib.org/

| Value | Count | Frequency (%) |
| --- | --- | --- |
| 40\_54 | 2 | 33.3% |
| 23\_39 | 1 | 16.7% |
| mais | 1 | 16.7% |
| de | 1 | 16.7% |
| 54 | 1 | 16.7% |

- Characters
- Categories
- Scripts
- Blocks

#### Most occurring characters

| Value | Count | Frequency (%) |
| --- | --- | --- |
| 4 | 5 | 20.0% |
| \_ | 3 | 12.0% |
| 5 | 3 | 12.0% |
| 0 | 2 | 8.0% |
| 3 | 2 | 8.0% |
|  | 2 | 8.0% |
| 2 | 1 | 4.0% |
| 9 | 1 | 4.0% |
| M | 1 | 4.0% |
| a | 1 | 4.0% |
| Other values (4) | 4 | 16.0% |

#### Most occurring categories

| Value | Count | Frequency (%) |
| --- | --- | --- |
| Decimal Number | 14 | 56.0% |
| Lowercase Letter | 5 | 20.0% |
| Connector Punctuation | 3 | 12.0% |
| Space Separator | 2 | 8.0% |
| Uppercase Letter | 1 | 4.0% |

#### Most frequent character per category

##### *Decimal Number*

| Value | Count | Frequency (%) |
| --- | --- | --- |
| 4 | 5 | 35.7% |
| 5 | 3 | 21.4% |
| 0 | 2 | 14.3% |
| 3 | 2 | 14.3% |
| 2 | 1 | 7.1% |
| 9 | 1 | 7.1% |

##### *Lowercase Letter*

| Value | Count | Frequency (%) |
| --- | --- | --- |
| a | 1 | 20.0% |
| i | 1 | 20.0% |
| s | 1 | 20.0% |
| d | 1 | 20.0% |
| e | 1 | 20.0% |

##### *Connector Punctuation*

| Value | Count | Frequency (%) |
| --- | --- | --- |
| \_ | 3 | 100.0% |

##### *Space Separator*

| Value | Count | Frequency (%) |
| --- | --- | --- |
|  | 2 | 100.0% |

##### *Uppercase Letter*

| Value | Count | Frequency (%) |
| --- | --- | --- |
| M | 1 | 100.0% |

#### Most occurring scripts

| Value | Count | Frequency (%) |
| --- | --- | --- |
| Common | 19 | 76.0% |
| Latin | 6 | 24.0% |

#### Most frequent character per script

##### *Common*

| Value | Count | Frequency (%) |
| --- | --- | --- |
| 4 | 5 | 26.3% |
| \_ | 3 | 15.8% |
| 5 | 3 | 15.8% |
| 0 | 2 | 10.5% |
| 3 | 2 | 10.5% |
|  | 2 | 10.5% |
| 2 | 1 | 5.3% |
| 9 | 1 | 5.3% |

##### *Latin*

| Value | Count | Frequency (%) |
| --- | --- | --- |
| M | 1 | 16.7% |
| a | 1 | 16.7% |
| i | 1 | 16.7% |
| s | 1 | 16.7% |
| d | 1 | 16.7% |
| e | 1 | 16.7% |

#### Most occurring blocks

| Value | Count | Frequency (%) |
| --- | --- | --- |
| ASCII | 25 | 100.0% |

#### Most frequent character per block

##### *ASCII*

| Value | Count | Frequency (%) |
| --- | --- | --- |
| 4 | 5 | 20.0% |
| \_ | 3 | 12.0% |
| 5 | 3 | 12.0% |
| 0 | 2 | 8.0% |
| 3 | 2 | 8.0% |
|  | 2 | 8.0% |
| 2 | 1 | 4.0% |
| 9 | 1 | 4.0% |
| M | 1 | 4.0% |
| a | 1 | 4.0% |
| Other values (4) | 4 | 16.0% |

HISTOPATOL  
Categorical

|  |  |
| --- | --- |
| Distinct | 1 |
| Distinct (%) | 25.0% |
| Missing | 0 |
| Missing (%) | 0.0% |
| Memory size | 64.0 B |

|  |  |
| --- | --- |
| N/realiz | 4 |

More details

- Overview
- Categories
- Words
- Characters

Length

|  |  |
| --- | --- |
| Max length | 8 |
| Median length | 8 |
| Mean length | 8 |
| Min length | 8 |

Characters and Unicode

|  |  |
| --- | --- |
| Total characters | 32 |
| Distinct characters | 8 |
| Distinct categories | 3 ? |
| Distinct scripts | 2 ? |
| Distinct blocks | 1 ? |

The Unicode Standard assigns character properties to each code point, which can be used to analyse textual variables.

Unique

|  |  |
| --- | --- |
| Unique | 0 ? |
| Unique (%) | 0.0% |

Sample

|  |  |
| --- | --- |
| 1st row | N/realiz |
| 2nd row | N/realiz |
| 3rd row | N/realiz |
| 4th row | N/realiz |

#### Common Values

| Value | Count | Frequency (%) |
| --- | --- | --- |
| N/realiz | 4 | 100.0% |

#### Length

xml version="1.0" encoding="utf-8" standalone="no"?2023-10-31T16:39:02.350989image/svg+xmlMatplotlib v3.6.0, https://matplotlib.org/ 

Histogram of lengths of the category

#### Common Values (Plot)

xml version="1.0" encoding="utf-8" standalone="no"?2023-10-31T16:39:02.490971image/svg+xmlMatplotlib v3.6.0, https://matplotlib.org/

| Value | Count | Frequency (%) |
| --- | --- | --- |
| n/realiz | 4 | 100.0% |

- Characters
- Categories
- Scripts
- Blocks

#### Most occurring characters

| Value | Count | Frequency (%) |
| --- | --- | --- |
| N | 4 | 12.5% |
| / | 4 | 12.5% |
| r | 4 | 12.5% |
| e | 4 | 12.5% |
| a | 4 | 12.5% |
| l | 4 | 12.5% |
| i | 4 | 12.5% |
| z | 4 | 12.5% |

#### Most occurring categories

| Value | Count | Frequency (%) |
| --- | --- | --- |
| Lowercase Letter | 24 | 75.0% |
| Uppercase Letter | 4 | 12.5% |
| Other Punctuation | 4 | 12.5% |

#### Most frequent character per category

##### *Lowercase Letter*

| Value | Count | Frequency (%) |
| --- | --- | --- |
| r | 4 | 16.7% |
| e | 4 | 16.7% |
| a | 4 | 16.7% |
| l | 4 | 16.7% |
| i | 4 | 16.7% |
| z | 4 | 16.7% |

##### *Uppercase Letter*

| Value | Count | Frequency (%) |
| --- | --- | --- |
| N | 4 | 100.0% |

##### *Other Punctuation*

| Value | Count | Frequency (%) |
| --- | --- | --- |
| / | 4 | 100.0% |

#### Most occurring scripts

| Value | Count | Frequency (%) |
| --- | --- | --- |
| Latin | 28 | 87.5% |
| Common | 4 | 12.5% |

#### Most frequent character per script

##### *Latin*

| Value | Count | Frequency (%) |
| --- | --- | --- |
| N | 4 | 14.3% |
| r | 4 | 14.3% |
| e | 4 | 14.3% |
| a | 4 | 14.3% |
| l | 4 | 14.3% |
| i | 4 | 14.3% |
| z | 4 | 14.3% |

##### *Common*

| Value | Count | Frequency (%) |
| --- | --- | --- |
| / | 4 | 100.0% |

#### Most occurring blocks

| Value | Count | Frequency (%) |
| --- | --- | --- |
| ASCII | 32 | 100.0% |

#### Most frequent character per block

##### *ASCII*

| Value | Count | Frequency (%) |
| --- | --- | --- |
| N | 4 | 12.5% |
| / | 4 | 12.5% |
| r | 4 | 12.5% |
| e | 4 | 12.5% |
| a | 4 | 12.5% |
| l | 4 | 12.5% |
| i | 4 | 12.5% |
| z | 4 | 12.5% |

Status\_Resistencia  
Categorical

|  |  |
| --- | --- |
| Distinct | 1 |
| Distinct (%) | 25.0% |
| Missing | 0 |
| Missing (%) | 0.0% |
| Memory size | 64.0 B |

|  |  |
| --- | --- |
| 1 | 4 |

More details

- Overview
- Categories
- Words
- Characters

Length

|  |  |
| --- | --- |
| Max length | 1 |
| Median length | 1 |
| Mean length | 1 |
| Min length | 1 |

Characters and Unicode

|  |  |
| --- | --- |
| Total characters | 4 |
| Distinct characters | 1 |
| Distinct categories | 1 ? |
| Distinct scripts | 1 ? |
| Distinct blocks | 1 ? |

The Unicode Standard assigns character properties to each code point, which can be used to analyse textual variables.

Unique

|  |  |
| --- | --- |
| Unique | 0 ? |
| Unique (%) | 0.0% |

Sample

|  |  |
| --- | --- |
| 1st row | 1 |
| 2nd row | 1 |
| 3rd row | 1 |
| 4th row | 1 |

#### Common Values

| Value | Count | Frequency (%) |
| --- | --- | --- |
| 1 | 4 | 100.0% |

#### Length

xml version="1.0" encoding="utf-8" standalone="no"?2023-10-31T16:39:02.598174image/svg+xmlMatplotlib v3.6.0, https://matplotlib.org/ 

Histogram of lengths of the category

#### Common Values (Plot)

xml version="1.0" encoding="utf-8" standalone="no"?2023-10-31T16:39:03.228453image/svg+xmlMatplotlib v3.6.0, https://matplotlib.org/

| Value | Count | Frequency (%) |
| --- | --- | --- |
| 1 | 4 | 100.0% |

- Characters
- Categories
- Scripts
- Blocks

#### Most occurring characters

| Value | Count | Frequency (%) |
| --- | --- | --- |
| 1 | 4 | 100.0% |

#### Most occurring categories

| Value | Count | Frequency (%) |
| --- | --- | --- |
| Decimal Number | 4 | 100.0% |

#### Most frequent character per category

##### *Decimal Number*

| Value | Count | Frequency (%) |
| --- | --- | --- |
| 1 | 4 | 100.0% |

#### Most occurring scripts

| Value | Count | Frequency (%) |
| --- | --- | --- |
| Common | 4 | 100.0% |

#### Most frequent character per script

##### *Common*

| Value | Count | Frequency (%) |
| --- | --- | --- |
| 1 | 4 | 100.0% |

#### Most occurring blocks

| Value | Count | Frequency (%) |
| --- | --- | --- |
| ASCII | 4 | 100.0% |

#### Most frequent character per block

##### *ASCII*

| Value | Count | Frequency (%) |
| --- | --- | --- |
| 1 | 4 | 100.0% |

Cluster  
Categorical

|  |  |
| --- | --- |
| Distinct | 1 |
| Distinct (%) | 25.0% |
| Missing | 0 |
| Missing (%) | 0.0% |
| Memory size | 64.0 B |

|  |  |
| --- | --- |
| 1 | 4 |

More details

- Overview
- Categories
- Words
- Characters

Length

|  |  |
| --- | --- |
| Max length | 1 |
| Median length | 1 |
| Mean length | 1 |
| Min length | 1 |

Characters and Unicode

|  |  |
| --- | --- |
| Total characters | 4 |
| Distinct characters | 1 |
| Distinct categories | 1 ? |
| Distinct scripts | 1 ? |
| Distinct blocks | 1 ? |

The Unicode Standard assigns character properties to each code point, which can be used to analyse textual variables.

Unique

|  |  |
| --- | --- |
| Unique | 0 ? |
| Unique (%) | 0.0% |

Sample

|  |  |
| --- | --- |
| 1st row | 1 |
| 2nd row | 1 |
| 3rd row | 1 |
| 4th row | 1 |

#### Common Values

| Value | Count | Frequency (%) |
| --- | --- | --- |
| 1 | 4 | 100.0% |

#### Length

xml version="1.0" encoding="utf-8" standalone="no"?2023-10-31T16:39:03.335687image/svg+xmlMatplotlib v3.6.0, https://matplotlib.org/ 

Histogram of lengths of the category

#### Common Values (Plot)

xml version="1.0" encoding="utf-8" standalone="no"?2023-10-31T16:39:03.473168image/svg+xmlMatplotlib v3.6.0, https://matplotlib.org/

| Value | Count | Frequency (%) |
| --- | --- | --- |
| 1 | 4 | 100.0% |

- Characters
- Categories
- Scripts
- Blocks

#### Most occurring characters

| Value | Count | Frequency (%) |
| --- | --- | --- |
| 1 | 4 | 100.0% |

#### Most occurring categories

| Value | Count | Frequency (%) |
| --- | --- | --- |
| Decimal Number | 4 | 100.0% |

#### Most frequent character per category

##### *Decimal Number*

| Value | Count | Frequency (%) |
| --- | --- | --- |
| 1 | 4 | 100.0% |

#### Most occurring scripts

| Value | Count | Frequency (%) |
| --- | --- | --- |
| Common | 4 | 100.0% |

#### Most frequent character per script

##### *Common*

| Value | Count | Frequency (%) |
| --- | --- | --- |
| 1 | 4 | 100.0% |

#### Most occurring blocks

| Value | Count | Frequency (%) |
| --- | --- | --- |
| ASCII | 4 | 100.0% |

#### Most frequent character per block

##### *ASCII*

| Value | Count | Frequency (%) |
| --- | --- | --- |
| 1 | 4 | 100.0% |

Probabilidade  
Categorical

`HIGH CORRELATION`  `UNIFORM`  `UNIQUE`

|  |  |
| --- | --- |
| Distinct | 4 |
| Distinct (%) | 100.0% |
| Missing | 0 |
| Missing (%) | 0.0% |
| Memory size | 64.0 B |

|  |  |
| --- | --- |
| 0.28968234395794423 | 1 |
| 0.3027694902809071 | 1 |
| 0.2013750686572229 | 1 |
| 0.21357264163761477 | 1 |

More details

- Overview
- Categories
- Words
- Characters

Length

|  |  |
| --- | --- |
| Max length | 19 |
| Median length | 18.5 |
| Mean length | 18.5 |
| Min length | 18 |

Characters and Unicode

|  |  |
| --- | --- |
| Total characters | 74 |
| Distinct characters | 11 |
| Distinct categories | 2 ? |
| Distinct scripts | 1 ? |
| Distinct blocks | 1 ? |

The Unicode Standard assigns character properties to each code point, which can be used to analyse textual variables.

Unique

|  |  |
| --- | --- |
| Unique | 4 ? |
| Unique (%) | 100.0% |

Sample

|  |  |
| --- | --- |
| 1st row | 0.28968234395794423 |
| 2nd row | 0.3027694902809071 |
| 3rd row | 0.2013750686572229 |
| 4th row | 0.21357264163761477 |

#### Common Values

| Value | Count | Frequency (%) |
| --- | --- | --- |
| 0.28968234395794423 | 1 | 25.0% |
| 0.3027694902809071 | 1 | 25.0% |
| 0.2013750686572229 | 1 | 25.0% |
| 0.21357264163761477 | 1 | 25.0% |

#### Length

xml version="1.0" encoding="utf-8" standalone="no"?2023-10-31T16:39:03.584148image/svg+xmlMatplotlib v3.6.0, https://matplotlib.org/ 

Histogram of lengths of the category

#### Common Values (Plot)

xml version="1.0" encoding="utf-8" standalone="no"?2023-10-31T16:39:03.744673image/svg+xmlMatplotlib v3.6.0, https://matplotlib.org/

| Value | Count | Frequency (%) |
| --- | --- | --- |
| 0.28968234395794423 | 1 | 25.0% |
| 0.3027694902809071 | 1 | 25.0% |
| 0.2013750686572229 | 1 | 25.0% |
| 0.21357264163761477 | 1 | 25.0% |

- Characters
- Categories
- Scripts
- Blocks

#### Most occurring characters

| Value | Count | Frequency (%) |
| --- | --- | --- |
| 2 | 11 | 14.9% |
| 0 | 10 | 13.5% |
| 7 | 9 | 12.2% |
| 9 | 7 | 9.5% |
| 6 | 7 | 9.5% |
| 3 | 7 | 9.5% |
| 4 | 6 | 8.1% |
| 1 | 5 | 6.8% |
| . | 4 | 5.4% |
| 8 | 4 | 5.4% |

#### Most occurring categories

| Value | Count | Frequency (%) |
| --- | --- | --- |
| Decimal Number | 70 | 94.6% |
| Other Punctuation | 4 | 5.4% |

#### Most frequent character per category

##### *Decimal Number*

| Value | Count | Frequency (%) |
| --- | --- | --- |
| 2 | 11 | 15.7% |
| 0 | 10 | 14.3% |
| 7 | 9 | 12.9% |
| 9 | 7 | 10.0% |
| 6 | 7 | 10.0% |
| 3 | 7 | 10.0% |
| 4 | 6 | 8.6% |
| 1 | 5 | 7.1% |
| 8 | 4 | 5.7% |
| 5 | 4 | 5.7% |

##### *Other Punctuation*

| Value | Count | Frequency (%) |
| --- | --- | --- |
| . | 4 | 100.0% |

#### Most occurring scripts

| Value | Count | Frequency (%) |
| --- | --- | --- |
| Common | 74 | 100.0% |

#### Most frequent character per script

##### *Common*

| Value | Count | Frequency (%) |
| --- | --- | --- |
| 2 | 11 | 14.9% |
| 0 | 10 | 13.5% |
| 7 | 9 | 12.2% |
| 9 | 7 | 9.5% |
| 6 | 7 | 9.5% |
| 3 | 7 | 9.5% |
| 4 | 6 | 8.1% |
| 1 | 5 | 6.8% |
| . | 4 | 5.4% |
| 8 | 4 | 5.4% |

#### Most occurring blocks

| Value | Count | Frequency (%) |
| --- | --- | --- |
| ASCII | 74 | 100.0% |

#### Most frequent character per block

##### *ASCII*

| Value | Count | Frequency (%) |
| --- | --- | --- |
| 2 | 11 | 14.9% |
| 0 | 10 | 13.5% |
| 7 | 9 | 12.2% |
| 9 | 7 | 9.5% |
| 6 | 7 | 9.5% |
| 3 | 7 | 9.5% |
| 4 | 6 | 8.1% |
| 1 | 5 | 6.8% |
| . | 4 | 5.4% |
| 8 | 4 | 5.4% |

# Correlations

- Auto

- Heatmap
- Table

xml version="1.0" encoding="utf-8" standalone="no"?2023-10-31T16:39:03.881243image/svg+xmlMatplotlib v3.6.0, https://matplotlib.org/

|  | faixaEtaria | sexo | ESCOLARID | TIPOCUP | tipoCaso | descoberta | cultEsc | RX | DROGADICAO | motMudEsquema | idade | Probabilidade |
| --- | --- | --- | --- | --- | --- | --- | --- | --- | --- | --- | --- | --- |
| faixaEtaria | 1.000 | 0.707 | 1.000 | 0.707 | 0.707 | 0.000 | 0.000 | 0.000 | 0.707 | 0.000 | 1.000 | 1.000 |
| sexo | 0.707 | 1.000 | 1.000 | 0.000 | 0.000 | 0.000 | 0.000 | 0.000 | 0.000 | 0.000 | 0.707 | 1.000 |
| ESCOLARID | 1.000 | 1.000 | 1.000 | 1.000 | 1.000 | 1.000 | 1.000 | 1.000 | 1.000 | 1.000 | 1.000 | 1.000 |
| TIPOCUP | 0.707 | 0.000 | 1.000 | 1.000 | 0.000 | 0.707 | 0.000 | 0.000 | 0.000 | 0.000 | 0.707 | 1.000 |
| tipoCaso | 0.707 | 0.000 | 1.000 | 0.000 | 1.000 | 0.000 | 0.000 | 0.000 | 0.000 | 0.000 | 0.707 | 1.000 |
| descoberta | 0.000 | 0.000 | 1.000 | 0.707 | 0.000 | 1.000 | 0.000 | 0.707 | 0.707 | 0.707 | 0.000 | 1.000 |
| cultEsc | 0.000 | 0.000 | 1.000 | 0.000 | 0.000 | 0.000 | 1.000 | 0.000 | 0.000 | 0.000 | 0.000 | 1.000 |
| RX | 0.000 | 0.000 | 1.000 | 0.000 | 0.000 | 0.707 | 0.000 | 1.000 | 0.000 | 0.000 | 0.000 | 1.000 |
| DROGADICAO | 0.707 | 0.000 | 1.000 | 0.000 | 0.000 | 0.707 | 0.000 | 0.000 | 1.000 | 0.000 | 0.707 | 1.000 |
| motMudEsquema | 0.000 | 0.000 | 1.000 | 0.000 | 0.000 | 0.707 | 0.000 | 0.000 | 0.000 | 1.000 | 0.000 | 1.000 |
| idade | 1.000 | 0.707 | 1.000 | 0.707 | 0.707 | 0.000 | 0.000 | 0.000 | 0.707 | 0.000 | 1.000 | 1.000 |
| Probabilidade | 1.000 | 1.000 | 1.000 | 1.000 | 1.000 | 1.000 | 1.000 | 1.000 | 1.000 | 1.000 | 1.000 | 1.000 |

# Missing values

- Count
- Matrix

xml version="1.0" encoding="utf-8" standalone="no"?2023-10-31T16:38:55.860847image/svg+xmlMatplotlib v3.6.0, https://matplotlib.org/ 

A simple visualization of nullity by column.

xml version="1.0" encoding="utf-8" standalone="no"?2023-10-31T16:38:56.401112image/svg+xmlMatplotlib v3.6.0, https://matplotlib.org/ 

Nullity matrix is a data-dense display which lets you quickly visually pick out patterns in data completion.

# Sample

- First rows
- Last rows

|  | faixaEtaria | sexo | ESCOLARID | TIPOCUP | sitAtual | tipoCaso | FORMACLIN1 | classif | descoberta | bac | BACOUTRO | cultEsc | RX | NECROP | hiv | aids | DIABETES | ALCOOLISMO | MENTAL | DROGADICAO | TABAGISMO | motMudEsquema | tipoTrat | idade | HISTOPATOL | Status\_Resistencia | Cluster | Probabilidade |
| --- | --- | --- | --- | --- | --- | --- | --- | --- | --- | --- | --- | --- | --- | --- | --- | --- | --- | --- | --- | --- | --- | --- | --- | --- | --- | --- | --- | --- |
| 1054 | 20\_29 | F | De 4 a 7 anos | Desempregado | Cura | Novo | Pul | Pul | Elucidacao Diagn. em Internacao | Pos | N/realiz | N/realiz | Susp TB | N/realiz | Neg | N | N | N | N | S | N | Nulo | Supervisionado | 23\_39 | N/realiz | 1 | 1 | 0.289682 |
| 1459 | 40\_49 | F | De 8 a 11 anos | Outra | Cura | Novo | Pul | Pul | Urgencia / Emergencia | Pos | N/realiz | Neg | Susp TB | N/realiz | Neg | N | N | N | N | N | N | Nulo | Supervisionado | 40\_54 | N/realiz | 1 | 1 | 0.302769 |
| 528 | 40\_49 | F | De 1 a 3 anos | Outra | Cura | Novo | Pul | Pul | Investigacao de Contatos | Pos | N/realiz | N/realiz | N/realiz | N/realiz | Neg | N | N | N | N | N | N | Intolerancia/Toxicidade | Supervisionado | 40\_54 | N/realiz | 1 | 1 | 0.201375 |
| 218 | 50\_59 | M | De 12 a 14 anos | Outra | Cura | Recidiva | Pul | Pul | Urgencia / Emergencia | Pos | N/realiz | N/realiz | Susp TB | N/realiz | Neg | N | N | N | N | N | N | Nulo | Supervisionado | Mais de 54 | N/realiz | 1 | 1 | 0.213573 |

|  | faixaEtaria | sexo | ESCOLARID | TIPOCUP | sitAtual | tipoCaso | FORMACLIN1 | classif | descoberta | bac | BACOUTRO | cultEsc | RX | NECROP | hiv | aids | DIABETES | ALCOOLISMO | MENTAL | DROGADICAO | TABAGISMO | motMudEsquema | tipoTrat | idade | HISTOPATOL | Status\_Resistencia | Cluster | Probabilidade |
| --- | --- | --- | --- | --- | --- | --- | --- | --- | --- | --- | --- | --- | --- | --- | --- | --- | --- | --- | --- | --- | --- | --- | --- | --- | --- | --- | --- | --- |
| 1054 | 20\_29 | F | De 4 a 7 anos | Desempregado | Cura | Novo | Pul | Pul | Elucidacao Diagn. em Internacao | Pos | N/realiz | N/realiz | Susp TB | N/realiz | Neg | N | N | N | N | S | N | Nulo | Supervisionado | 23\_39 | N/realiz | 1 | 1 | 0.289682 |
| 1459 | 40\_49 | F | De 8 a 11 anos | Outra | Cura | Novo | Pul | Pul | Urgencia / Emergencia | Pos | N/realiz | Neg | Susp TB | N/realiz | Neg | N | N | N | N | N | N | Nulo | Supervisionado | 40\_54 | N/realiz | 1 | 1 | 0.302769 |
| 528 | 40\_49 | F | De 1 a 3 anos | Outra | Cura | Novo | Pul | Pul | Investigacao de Contatos | Pos | N/realiz | N/realiz | N/realiz | N/realiz | Neg | N | N | N | N | N | N | Intolerancia/Toxicidade | Supervisionado | 40\_54 | N/realiz | 1 | 1 | 0.201375 |
| 218 | 50\_59 | M | De 12 a 14 anos | Outra | Cura | Recidiva | Pul | Pul | Urgencia / Emergencia | Pos | N/realiz | N/realiz | Susp TB | N/realiz | Neg | N | N | N | N | N | N | Nulo | Supervisionado | Mais de 54 | N/realiz | 1 | 1 | 0.213573 |

Report generated by YData.

 
