# Supplementary material for: Model for predicting drug resistance based on the clinical profile of tuberculosis patients using machine learning techniques
Source: PeerJ Comput Sci. 2024 Oct 14;10:e2246. doi: 10.7717/peerj-cs.2246 (PMC11623081; doi:10.7717/peerj-cs.2246)
Supplement: Supplemental Information 2 [file peerj-cs-10-2246-s002.zip › code/EDA/false_negative_3.html]

Pandas Profiling Report 

Toggle navigationPandas Profiling Report

- Overview
- Variables
- Correlations
- Missing values
- Sample

# Overview

- Overview
- Alerts 33
- Reproduction

Dataset statistics

|  |  |
| --- | --- |
| Number of variables | 28 |
| Number of observations | 4 |
| Missing cells | 0 |
| Missing cells (%) | 0.0% |
| Duplicate rows | 0 |
| Duplicate rows (%) | 0.0% |
| Total size in memory | 1000.0 B |
| Average record size in memory | 250.0 B |

Variable types

|  |  |
| --- | --- |
| Categorical | 25 |
| Boolean | 3 |

Alerts

| `sexo` has constant value "M" | Constant |
| `TIPOCUP` has constant value "Outra" | Constant |
| `tipoCaso` has constant value "Novo" | Constant |
| `cultEsc` has constant value "N/realiz" | Constant |
| `NECROP` has constant value "N/realiz" | Constant |
| `aids` has constant value "False" | Constant |
| `DIABETES` has constant value "False" | Constant |
| `MENTAL` has constant value "False" | Constant |
| `motMudEsquema` has constant value "Nulo" | Constant |
| `tipoTrat` has constant value "Supervisionado" | Constant |
| `HISTOPATOL` has constant value "N/realiz" | Constant |
| `Status_Resistencia` has constant value "1" | Constant |
| `Cluster` has constant value "2" | Constant |
| `faixaEtaria` is highly overall correlated with `bac` and 4 other fields | High correlation |
| `ESCOLARID` is highly overall correlated with `sitAtual` and 3 other fields | High correlation |
| `sitAtual` is highly overall correlated with `ESCOLARID` and 2 other fields | High correlation |
| `FORMACLIN1` is highly overall correlated with `classif` and 3 other fields | High correlation |
| `classif` is highly overall correlated with `FORMACLIN1` and 3 other fields | High correlation |
| `descoberta` is highly overall correlated with `sitAtual` and 4 other fields | High correlation |
| `bac` is highly overall correlated with `faixaEtaria` and 1 other fields | High correlation |
| `BACOUTRO` is highly overall correlated with `DROGADICAO` and 2 other fields | High correlation |
| `RX` is highly overall correlated with `FORMACLIN1` and 3 other fields | High correlation |
| `hiv` is highly overall correlated with `faixaEtaria` and 5 other fields | High correlation |
| `ALCOOLISMO` is highly overall correlated with `faixaEtaria` and 1 other fields | High correlation |
| `DROGADICAO` is highly overall correlated with `descoberta` and 2 other fields | High correlation |
| `TABAGISMO` is highly overall correlated with `ESCOLARID` and 2 other fields | High correlation |
| `idade` is highly overall correlated with `faixaEtaria` and 3 other fields | High correlation |
| `Probabilidade` is highly overall correlated with `faixaEtaria` and 13 other fields | High correlation |
| `bac` is uniformly distributed | Uniform |
| `ALCOOLISMO` is uniformly distributed | Uniform |
| `DROGADICAO` is uniformly distributed | Uniform |
| `Probabilidade` is uniformly distributed | Uniform |
| `Probabilidade` has unique values | Unique |

Reproduction

|  |  |
| --- | --- |
| Analysis started | 2023-10-31 19:44:32.874711 |
| Analysis finished | 2023-10-31 19:44:35.297205 |
| Duration | 2.42 seconds |
| Software version | pandas-profiling v3.6.6 |
| Download configuration | config.json |

# Variables

Select ColumnsfaixaEtariasexoESCOLARIDTIPOCUPsitAtualtipoCasoFORMACLIN1classifdescobertabacBACOUTROcultEscRXNECROPhivaidsDIABETESALCOOLISMOMENTALDROGADICAOTABAGISMOmotMudEsquematipoTratidadeHISTOPATOLStatus\_ResistenciaClusterProbabilidade

faixaEtaria  
Categorical

|  |  |
| --- | --- |
| Distinct | 3 |
| Distinct (%) | 75.0% |
| Missing | 0 |
| Missing (%) | 0.0% |
| Memory size | 64.0 B |

|  |  |
| --- | --- |
| 30\_39 | 2 |
| 60\_69 | 1 |
| 20\_29 | 1 |

More details

- Overview
- Categories
- Words
- Characters

Length

|  |  |
| --- | --- |
| Max length | 5 |
| Median length | 5 |
| Mean length | 5 |
| Min length | 5 |

Characters and Unicode

|  |  |
| --- | --- |
| Total characters | 20 |
| Distinct characters | 6 |
| Distinct categories | 2 ? |
| Distinct scripts | 1 ? |
| Distinct blocks | 1 ? |

The Unicode Standard assigns character properties to each code point, which can be used to analyse textual variables.

Unique

|  |  |
| --- | --- |
| Unique | 2 ? |
| Unique (%) | 50.0% |

Sample

|  |  |
| --- | --- |
| 1st row | 60\_69 |
| 2nd row | 30\_39 |
| 3rd row | 20\_29 |
| 4th row | 30\_39 |

#### Common Values

| Value | Count | Frequency (%) |
| --- | --- | --- |
| 30\_39 | 2 | 50.0% |
| 60\_69 | 1 | 25.0% |
| 20\_29 | 1 | 25.0% |

#### Length

xml version="1.0" encoding="utf-8" standalone="no"?2023-10-31T16:44:35.370350image/svg+xmlMatplotlib v3.6.0, https://matplotlib.org/ 

Histogram of lengths of the category

#### Common Values (Plot)

xml version="1.0" encoding="utf-8" standalone="no"?2023-10-31T16:44:35.522906image/svg+xmlMatplotlib v3.6.0, https://matplotlib.org/

| Value | Count | Frequency (%) |
| --- | --- | --- |
| 30\_39 | 2 | 50.0% |
| 60\_69 | 1 | 25.0% |
| 20\_29 | 1 | 25.0% |

- Characters
- Categories
- Scripts
- Blocks

#### Most occurring characters

| Value | Count | Frequency (%) |
| --- | --- | --- |
| 3 | 4 | 20.0% |
| 0 | 4 | 20.0% |
| \_ | 4 | 20.0% |
| 9 | 4 | 20.0% |
| 6 | 2 | 10.0% |
| 2 | 2 | 10.0% |

#### Most occurring categories

| Value | Count | Frequency (%) |
| --- | --- | --- |
| Decimal Number | 16 | 80.0% |
| Connector Punctuation | 4 | 20.0% |

#### Most frequent character per category

##### *Decimal Number*

| Value | Count | Frequency (%) |
| --- | --- | --- |
| 3 | 4 | 25.0% |
| 0 | 4 | 25.0% |
| 9 | 4 | 25.0% |
| 6 | 2 | 12.5% |
| 2 | 2 | 12.5% |

##### *Connector Punctuation*

| Value | Count | Frequency (%) |
| --- | --- | --- |
| \_ | 4 | 100.0% |

#### Most occurring scripts

| Value | Count | Frequency (%) |
| --- | --- | --- |
| Common | 20 | 100.0% |

#### Most frequent character per script

##### *Common*

| Value | Count | Frequency (%) |
| --- | --- | --- |
| 3 | 4 | 20.0% |
| 0 | 4 | 20.0% |
| \_ | 4 | 20.0% |
| 9 | 4 | 20.0% |
| 6 | 2 | 10.0% |
| 2 | 2 | 10.0% |

#### Most occurring blocks

| Value | Count | Frequency (%) |
| --- | --- | --- |
| ASCII | 20 | 100.0% |

#### Most frequent character per block

##### *ASCII*

| Value | Count | Frequency (%) |
| --- | --- | --- |
| 3 | 4 | 20.0% |
| 0 | 4 | 20.0% |
| \_ | 4 | 20.0% |
| 9 | 4 | 20.0% |
| 6 | 2 | 10.0% |
| 2 | 2 | 10.0% |

sexo  
Categorical

|  |  |
| --- | --- |
| Distinct | 1 |
| Distinct (%) | 25.0% |
| Missing | 0 |
| Missing (%) | 0.0% |
| Memory size | 160.0 B |

|  |  |
| --- | --- |
| M | 4 |

More details

- Overview
- Categories
- Words
- Characters

Length

|  |  |
| --- | --- |
| Max length | 1 |
| Median length | 1 |
| Mean length | 1 |
| Min length | 1 |

Characters and Unicode

|  |  |
| --- | --- |
| Total characters | 4 |
| Distinct characters | 1 |
| Distinct categories | 1 ? |
| Distinct scripts | 1 ? |
| Distinct blocks | 1 ? |

The Unicode Standard assigns character properties to each code point, which can be used to analyse textual variables.

Unique

|  |  |
| --- | --- |
| Unique | 0 ? |
| Unique (%) | 0.0% |

Sample

|  |  |
| --- | --- |
| 1st row | M |
| 2nd row | M |
| 3rd row | M |
| 4th row | M |

#### Common Values

| Value | Count | Frequency (%) |
| --- | --- | --- |
| M | 4 | 100.0% |

#### Length

xml version="1.0" encoding="utf-8" standalone="no"?2023-10-31T16:44:35.645820image/svg+xmlMatplotlib v3.6.0, https://matplotlib.org/ 

Histogram of lengths of the category

#### Common Values (Plot)

xml version="1.0" encoding="utf-8" standalone="no"?2023-10-31T16:44:35.781514image/svg+xmlMatplotlib v3.6.0, https://matplotlib.org/

| Value | Count | Frequency (%) |
| --- | --- | --- |
| m | 4 | 100.0% |

- Characters
- Categories
- Scripts
- Blocks

#### Most occurring characters

| Value | Count | Frequency (%) |
| --- | --- | --- |
| M | 4 | 100.0% |

#### Most occurring categories

| Value | Count | Frequency (%) |
| --- | --- | --- |
| Uppercase Letter | 4 | 100.0% |

#### Most frequent character per category

##### *Uppercase Letter*

| Value | Count | Frequency (%) |
| --- | --- | --- |
| M | 4 | 100.0% |

#### Most occurring scripts

| Value | Count | Frequency (%) |
| --- | --- | --- |
| Latin | 4 | 100.0% |

#### Most frequent character per script

##### *Latin*

| Value | Count | Frequency (%) |
| --- | --- | --- |
| M | 4 | 100.0% |

#### Most occurring blocks

| Value | Count | Frequency (%) |
| --- | --- | --- |
| ASCII | 4 | 100.0% |

#### Most frequent character per block

##### *ASCII*

| Value | Count | Frequency (%) |
| --- | --- | --- |
| M | 4 | 100.0% |

ESCOLARID  
Categorical

|  |  |
| --- | --- |
| Distinct | 3 |
| Distinct (%) | 75.0% |
| Missing | 0 |
| Missing (%) | 0.0% |
| Memory size | 64.0 B |

|  |  |
| --- | --- |
| De 8 a 11 anos | 2 |
| Nenhuma | 1 |
| De 4 a 7 anos | 1 |

More details

- Overview
- Categories
- Words
- Characters

Length

|  |  |
| --- | --- |
| Max length | 14 |
| Median length | 13.5 |
| Mean length | 12 |
| Min length | 7 |

Characters and Unicode

|  |  |
| --- | --- |
| Total characters | 48 |
| Distinct characters | 15 |
| Distinct categories | 4 ? |
| Distinct scripts | 2 ? |
| Distinct blocks | 1 ? |

The Unicode Standard assigns character properties to each code point, which can be used to analyse textual variables.

Unique

|  |  |
| --- | --- |
| Unique | 2 ? |
| Unique (%) | 50.0% |

Sample

|  |  |
| --- | --- |
| 1st row | Nenhuma |
| 2nd row | De 4 a 7 anos |
| 3rd row | De 8 a 11 anos |
| 4th row | De 8 a 11 anos |

#### Common Values

| Value | Count | Frequency (%) |
| --- | --- | --- |
| De 8 a 11 anos | 2 | 50.0% |
| Nenhuma | 1 | 25.0% |
| De 4 a 7 anos | 1 | 25.0% |

#### Length

xml version="1.0" encoding="utf-8" standalone="no"?2023-10-31T16:44:35.900285image/svg+xmlMatplotlib v3.6.0, https://matplotlib.org/ 

Histogram of lengths of the category

#### Common Values (Plot)

xml version="1.0" encoding="utf-8" standalone="no"?2023-10-31T16:44:36.062652image/svg+xmlMatplotlib v3.6.0, https://matplotlib.org/

| Value | Count | Frequency (%) |
| --- | --- | --- |
| de | 3 | 18.8% |
| a | 3 | 18.8% |
| anos | 3 | 18.8% |
| 8 | 2 | 12.5% |
| 11 | 2 | 12.5% |
| nenhuma | 1 | 6.2% |
| 4 | 1 | 6.2% |
| 7 | 1 | 6.2% |

- Characters
- Categories
- Scripts
- Blocks

#### Most occurring characters

| Value | Count | Frequency (%) |
| --- | --- | --- |
|  | 12 | 25.0% |
| a | 7 | 14.6% |
| e | 4 | 8.3% |
| 1 | 4 | 8.3% |
| n | 4 | 8.3% |
| D | 3 | 6.2% |
| o | 3 | 6.2% |
| s | 3 | 6.2% |
| 8 | 2 | 4.2% |
| N | 1 | 2.1% |
| Other values (5) | 5 | 10.4% |

#### Most occurring categories

| Value | Count | Frequency (%) |
| --- | --- | --- |
| Lowercase Letter | 24 | 50.0% |
| Space Separator | 12 | 25.0% |
| Decimal Number | 8 | 16.7% |
| Uppercase Letter | 4 | 8.3% |

#### Most frequent character per category

##### *Lowercase Letter*

| Value | Count | Frequency (%) |
| --- | --- | --- |
| a | 7 | 29.2% |
| e | 4 | 16.7% |
| n | 4 | 16.7% |
| o | 3 | 12.5% |
| s | 3 | 12.5% |
| h | 1 | 4.2% |
| u | 1 | 4.2% |
| m | 1 | 4.2% |

##### *Decimal Number*

| Value | Count | Frequency (%) |
| --- | --- | --- |
| 1 | 4 | 50.0% |
| 8 | 2 | 25.0% |
| 4 | 1 | 12.5% |
| 7 | 1 | 12.5% |

##### *Uppercase Letter*

| Value | Count | Frequency (%) |
| --- | --- | --- |
| D | 3 | 75.0% |
| N | 1 | 25.0% |

##### *Space Separator*

| Value | Count | Frequency (%) |
| --- | --- | --- |
|  | 12 | 100.0% |

#### Most occurring scripts

| Value | Count | Frequency (%) |
| --- | --- | --- |
| Latin | 28 | 58.3% |
| Common | 20 | 41.7% |

#### Most frequent character per script

##### *Latin*

| Value | Count | Frequency (%) |
| --- | --- | --- |
| a | 7 | 25.0% |
| e | 4 | 14.3% |
| n | 4 | 14.3% |
| D | 3 | 10.7% |
| o | 3 | 10.7% |
| s | 3 | 10.7% |
| N | 1 | 3.6% |
| h | 1 | 3.6% |
| u | 1 | 3.6% |
| m | 1 | 3.6% |

##### *Common*

| Value | Count | Frequency (%) |
| --- | --- | --- |
|  | 12 | 60.0% |
| 1 | 4 | 20.0% |
| 8 | 2 | 10.0% |
| 4 | 1 | 5.0% |
| 7 | 1 | 5.0% |

#### Most occurring blocks

| Value | Count | Frequency (%) |
| --- | --- | --- |
| ASCII | 48 | 100.0% |

#### Most frequent character per block

##### *ASCII*

| Value | Count | Frequency (%) |
| --- | --- | --- |
|  | 12 | 25.0% |
| a | 7 | 14.6% |
| e | 4 | 8.3% |
| 1 | 4 | 8.3% |
| n | 4 | 8.3% |
| D | 3 | 6.2% |
| o | 3 | 6.2% |
| s | 3 | 6.2% |
| 8 | 2 | 4.2% |
| N | 1 | 2.1% |
| Other values (5) | 5 | 10.4% |

TIPOCUP  
Categorical

|  |  |
| --- | --- |
| Distinct | 1 |
| Distinct (%) | 25.0% |
| Missing | 0 |
| Missing (%) | 0.0% |
| Memory size | 64.0 B |

|  |  |
| --- | --- |
| Outra | 4 |

More details

- Overview
- Categories
- Words
- Characters

Length

|  |  |
| --- | --- |
| Max length | 5 |
| Median length | 5 |
| Mean length | 5 |
| Min length | 5 |

Characters and Unicode

|  |  |
| --- | --- |
| Total characters | 20 |
| Distinct characters | 5 |
| Distinct categories | 2 ? |
| Distinct scripts | 1 ? |
| Distinct blocks | 1 ? |

The Unicode Standard assigns character properties to each code point, which can be used to analyse textual variables.

Unique

|  |  |
| --- | --- |
| Unique | 0 ? |
| Unique (%) | 0.0% |

Sample

|  |  |
| --- | --- |
| 1st row | Outra |
| 2nd row | Outra |
| 3rd row | Outra |
| 4th row | Outra |

#### Common Values

| Value | Count | Frequency (%) |
| --- | --- | --- |
| Outra | 4 | 100.0% |

#### Length

xml version="1.0" encoding="utf-8" standalone="no"?2023-10-31T16:44:36.187508image/svg+xmlMatplotlib v3.6.0, https://matplotlib.org/ 

Histogram of lengths of the category

#### Common Values (Plot)

xml version="1.0" encoding="utf-8" standalone="no"?2023-10-31T16:44:36.323086image/svg+xmlMatplotlib v3.6.0, https://matplotlib.org/

| Value | Count | Frequency (%) |
| --- | --- | --- |
| outra | 4 | 100.0% |

- Characters
- Categories
- Scripts
- Blocks

#### Most occurring characters

| Value | Count | Frequency (%) |
| --- | --- | --- |
| O | 4 | 20.0% |
| u | 4 | 20.0% |
| t | 4 | 20.0% |
| r | 4 | 20.0% |
| a | 4 | 20.0% |

#### Most occurring categories

| Value | Count | Frequency (%) |
| --- | --- | --- |
| Lowercase Letter | 16 | 80.0% |
| Uppercase Letter | 4 | 20.0% |

#### Most frequent character per category

##### *Lowercase Letter*

| Value | Count | Frequency (%) |
| --- | --- | --- |
| u | 4 | 25.0% |
| t | 4 | 25.0% |
| r | 4 | 25.0% |
| a | 4 | 25.0% |

##### *Uppercase Letter*

| Value | Count | Frequency (%) |
| --- | --- | --- |
| O | 4 | 100.0% |

#### Most occurring scripts

| Value | Count | Frequency (%) |
| --- | --- | --- |
| Latin | 20 | 100.0% |

#### Most frequent character per script

##### *Latin*

| Value | Count | Frequency (%) |
| --- | --- | --- |
| O | 4 | 20.0% |
| u | 4 | 20.0% |
| t | 4 | 20.0% |
| r | 4 | 20.0% |
| a | 4 | 20.0% |

#### Most occurring blocks

| Value | Count | Frequency (%) |
| --- | --- | --- |
| ASCII | 20 | 100.0% |

#### Most frequent character per block

##### *ASCII*

| Value | Count | Frequency (%) |
| --- | --- | --- |
| O | 4 | 20.0% |
| u | 4 | 20.0% |
| t | 4 | 20.0% |
| r | 4 | 20.0% |
| a | 4 | 20.0% |

sitAtual  
Categorical

|  |  |
| --- | --- |
| Distinct | 2 |
| Distinct (%) | 50.0% |
| Missing | 0 |
| Missing (%) | 0.0% |
| Memory size | 64.0 B |

|  |  |
| --- | --- |
| Cura | 3 |
| Abandono | 1 |

More details

- Overview
- Categories
- Words
- Characters

Length

|  |  |
| --- | --- |
| Max length | 8 |
| Median length | 4 |
| Mean length | 5 |
| Min length | 4 |

Characters and Unicode

|  |  |
| --- | --- |
| Total characters | 20 |
| Distinct characters | 9 |
| Distinct categories | 2 ? |
| Distinct scripts | 1 ? |
| Distinct blocks | 1 ? |

The Unicode Standard assigns character properties to each code point, which can be used to analyse textual variables.

Unique

|  |  |
| --- | --- |
| Unique | 1 ? |
| Unique (%) | 25.0% |

Sample

|  |  |
| --- | --- |
| 1st row | Cura |
| 2nd row | Abandono |
| 3rd row | Cura |
| 4th row | Cura |

#### Common Values

| Value | Count | Frequency (%) |
| --- | --- | --- |
| Cura | 3 | 75.0% |
| Abandono | 1 | 25.0% |

#### Length

xml version="1.0" encoding="utf-8" standalone="no"?2023-10-31T16:44:36.452979image/svg+xmlMatplotlib v3.6.0, https://matplotlib.org/ 

Histogram of lengths of the category

#### Common Values (Plot)

xml version="1.0" encoding="utf-8" standalone="no"?2023-10-31T16:44:36.616083image/svg+xmlMatplotlib v3.6.0, https://matplotlib.org/

| Value | Count | Frequency (%) |
| --- | --- | --- |
| cura | 3 | 75.0% |
| abandono | 1 | 25.0% |

- Characters
- Categories
- Scripts
- Blocks

#### Most occurring characters

| Value | Count | Frequency (%) |
| --- | --- | --- |
| a | 4 | 20.0% |
| C | 3 | 15.0% |
| u | 3 | 15.0% |
| r | 3 | 15.0% |
| n | 2 | 10.0% |
| o | 2 | 10.0% |
| A | 1 | 5.0% |
| b | 1 | 5.0% |
| d | 1 | 5.0% |

#### Most occurring categories

| Value | Count | Frequency (%) |
| --- | --- | --- |
| Lowercase Letter | 16 | 80.0% |
| Uppercase Letter | 4 | 20.0% |

#### Most frequent character per category

##### *Lowercase Letter*

| Value | Count | Frequency (%) |
| --- | --- | --- |
| a | 4 | 25.0% |
| u | 3 | 18.8% |
| r | 3 | 18.8% |
| n | 2 | 12.5% |
| o | 2 | 12.5% |
| b | 1 | 6.2% |
| d | 1 | 6.2% |

##### *Uppercase Letter*

| Value | Count | Frequency (%) |
| --- | --- | --- |
| C | 3 | 75.0% |
| A | 1 | 25.0% |

#### Most occurring scripts

| Value | Count | Frequency (%) |
| --- | --- | --- |
| Latin | 20 | 100.0% |

#### Most frequent character per script

##### *Latin*

| Value | Count | Frequency (%) |
| --- | --- | --- |
| a | 4 | 20.0% |
| C | 3 | 15.0% |
| u | 3 | 15.0% |
| r | 3 | 15.0% |
| n | 2 | 10.0% |
| o | 2 | 10.0% |
| A | 1 | 5.0% |
| b | 1 | 5.0% |
| d | 1 | 5.0% |

#### Most occurring blocks

| Value | Count | Frequency (%) |
| --- | --- | --- |
| ASCII | 20 | 100.0% |

#### Most frequent character per block

##### *ASCII*

| Value | Count | Frequency (%) |
| --- | --- | --- |
| a | 4 | 20.0% |
| C | 3 | 15.0% |
| u | 3 | 15.0% |
| r | 3 | 15.0% |
| n | 2 | 10.0% |
| o | 2 | 10.0% |
| A | 1 | 5.0% |
| b | 1 | 5.0% |
| d | 1 | 5.0% |

tipoCaso  
Categorical

|  |  |
| --- | --- |
| Distinct | 1 |
| Distinct (%) | 25.0% |
| Missing | 0 |
| Missing (%) | 0.0% |
| Memory size | 64.0 B |

|  |  |
| --- | --- |
| Novo | 4 |

More details

- Overview
- Categories
- Words
- Characters

Length

|  |  |
| --- | --- |
| Max length | 4 |
| Median length | 4 |
| Mean length | 4 |
| Min length | 4 |

Characters and Unicode

|  |  |
| --- | --- |
| Total characters | 16 |
| Distinct characters | 3 |
| Distinct categories | 2 ? |
| Distinct scripts | 1 ? |
| Distinct blocks | 1 ? |

The Unicode Standard assigns character properties to each code point, which can be used to analyse textual variables.

Unique

|  |  |
| --- | --- |
| Unique | 0 ? |
| Unique (%) | 0.0% |

Sample

|  |  |
| --- | --- |
| 1st row | Novo |
| 2nd row | Novo |
| 3rd row | Novo |
| 4th row | Novo |

#### Common Values

| Value | Count | Frequency (%) |
| --- | --- | --- |
| Novo | 4 | 100.0% |

#### Length

xml version="1.0" encoding="utf-8" standalone="no"?2023-10-31T16:44:36.734805image/svg+xmlMatplotlib v3.6.0, https://matplotlib.org/ 

Histogram of lengths of the category

#### Common Values (Plot)

xml version="1.0" encoding="utf-8" standalone="no"?2023-10-31T16:44:36.920382image/svg+xmlMatplotlib v3.6.0, https://matplotlib.org/

| Value | Count | Frequency (%) |
| --- | --- | --- |
| novo | 4 | 100.0% |

- Characters
- Categories
- Scripts
- Blocks

#### Most occurring characters

| Value | Count | Frequency (%) |
| --- | --- | --- |
| o | 8 | 50.0% |
| N | 4 | 25.0% |
| v | 4 | 25.0% |

#### Most occurring categories

| Value | Count | Frequency (%) |
| --- | --- | --- |
| Lowercase Letter | 12 | 75.0% |
| Uppercase Letter | 4 | 25.0% |

#### Most frequent character per category

##### *Lowercase Letter*

| Value | Count | Frequency (%) |
| --- | --- | --- |
| o | 8 | 66.7% |
| v | 4 | 33.3% |

##### *Uppercase Letter*

| Value | Count | Frequency (%) |
| --- | --- | --- |
| N | 4 | 100.0% |

#### Most occurring scripts

| Value | Count | Frequency (%) |
| --- | --- | --- |
| Latin | 16 | 100.0% |

#### Most frequent character per script

##### *Latin*

| Value | Count | Frequency (%) |
| --- | --- | --- |
| o | 8 | 50.0% |
| N | 4 | 25.0% |
| v | 4 | 25.0% |

#### Most occurring blocks

| Value | Count | Frequency (%) |
| --- | --- | --- |
| ASCII | 16 | 100.0% |

#### Most frequent character per block

##### *ASCII*

| Value | Count | Frequency (%) |
| --- | --- | --- |
| o | 8 | 50.0% |
| N | 4 | 25.0% |
| v | 4 | 25.0% |

FORMACLIN1  
Categorical

|  |  |
| --- | --- |
| Distinct | 3 |
| Distinct (%) | 75.0% |
| Missing | 0 |
| Missing (%) | 0.0% |
| Memory size | 64.0 B |

|  |  |
| --- | --- |
| Pul | 2 |
| Multiplos Orgaos | 1 |
| Pleural | 1 |

More details

- Overview
- Categories
- Words
- Characters

Length

|  |  |
| --- | --- |
| Max length | 16 |
| Median length | 11.5 |
| Mean length | 7.25 |
| Min length | 3 |

Characters and Unicode

|  |  |
| --- | --- |
| Total characters | 29 |
| Distinct characters | 15 |
| Distinct categories | 3 ? |
| Distinct scripts | 2 ? |
| Distinct blocks | 1 ? |

The Unicode Standard assigns character properties to each code point, which can be used to analyse textual variables.

Unique

|  |  |
| --- | --- |
| Unique | 2 ? |
| Unique (%) | 50.0% |

Sample

|  |  |
| --- | --- |
| 1st row | Pul |
| 2nd row | Pul |
| 3rd row | Multiplos Orgaos |
| 4th row | Pleural |

#### Common Values

| Value | Count | Frequency (%) |
| --- | --- | --- |
| Pul | 2 | 50.0% |
| Multiplos Orgaos | 1 | 25.0% |
| Pleural | 1 | 25.0% |

#### Length

xml version="1.0" encoding="utf-8" standalone="no"?2023-10-31T16:44:37.037403image/svg+xmlMatplotlib v3.6.0, https://matplotlib.org/ 

Histogram of lengths of the category

#### Common Values (Plot)

xml version="1.0" encoding="utf-8" standalone="no"?2023-10-31T16:44:37.197592image/svg+xmlMatplotlib v3.6.0, https://matplotlib.org/

| Value | Count | Frequency (%) |
| --- | --- | --- |
| pul | 2 | 40.0% |
| multiplos | 1 | 20.0% |
| orgaos | 1 | 20.0% |
| pleural | 1 | 20.0% |

- Characters
- Categories
- Scripts
- Blocks

#### Most occurring characters

| Value | Count | Frequency (%) |
| --- | --- | --- |
| l | 6 | 20.7% |
| u | 4 | 13.8% |
| P | 3 | 10.3% |
| o | 2 | 6.9% |
| s | 2 | 6.9% |
| r | 2 | 6.9% |
| a | 2 | 6.9% |
| M | 1 | 3.4% |
| t | 1 | 3.4% |
| i | 1 | 3.4% |
| Other values (5) | 5 | 17.2% |

#### Most occurring categories

| Value | Count | Frequency (%) |
| --- | --- | --- |
| Lowercase Letter | 23 | 79.3% |
| Uppercase Letter | 5 | 17.2% |
| Space Separator | 1 | 3.4% |

#### Most frequent character per category

##### *Lowercase Letter*

| Value | Count | Frequency (%) |
| --- | --- | --- |
| l | 6 | 26.1% |
| u | 4 | 17.4% |
| o | 2 | 8.7% |
| s | 2 | 8.7% |
| r | 2 | 8.7% |
| a | 2 | 8.7% |
| t | 1 | 4.3% |
| i | 1 | 4.3% |
| p | 1 | 4.3% |
| g | 1 | 4.3% |

##### *Uppercase Letter*

| Value | Count | Frequency (%) |
| --- | --- | --- |
| P | 3 | 60.0% |
| M | 1 | 20.0% |
| O | 1 | 20.0% |

##### *Space Separator*

| Value | Count | Frequency (%) |
| --- | --- | --- |
|  | 1 | 100.0% |

#### Most occurring scripts

| Value | Count | Frequency (%) |
| --- | --- | --- |
| Latin | 28 | 96.6% |
| Common | 1 | 3.4% |

#### Most frequent character per script

##### *Latin*

| Value | Count | Frequency (%) |
| --- | --- | --- |
| l | 6 | 21.4% |
| u | 4 | 14.3% |
| P | 3 | 10.7% |
| o | 2 | 7.1% |
| s | 2 | 7.1% |
| r | 2 | 7.1% |
| a | 2 | 7.1% |
| M | 1 | 3.6% |
| t | 1 | 3.6% |
| i | 1 | 3.6% |
| Other values (4) | 4 | 14.3% |

##### *Common*

| Value | Count | Frequency (%) |
| --- | --- | --- |
|  | 1 | 100.0% |

#### Most occurring blocks

| Value | Count | Frequency (%) |
| --- | --- | --- |
| ASCII | 29 | 100.0% |

#### Most frequent character per block

##### *ASCII*

| Value | Count | Frequency (%) |
| --- | --- | --- |
| l | 6 | 20.7% |
| u | 4 | 13.8% |
| P | 3 | 10.3% |
| o | 2 | 6.9% |
| s | 2 | 6.9% |
| r | 2 | 6.9% |
| a | 2 | 6.9% |
| M | 1 | 3.4% |
| t | 1 | 3.4% |
| i | 1 | 3.4% |
| Other values (5) | 5 | 17.2% |

classif  
Categorical

|  |  |
| --- | --- |
| Distinct | 3 |
| Distinct (%) | 75.0% |
| Missing | 0 |
| Missing (%) | 0.0% |
| Memory size | 64.0 B |

|  |  |
| --- | --- |
| Pul | 2 |
| Dissem | 1 |
| Ext | 1 |

More details

- Overview
- Categories
- Words
- Characters

Length

|  |  |
| --- | --- |
| Max length | 6 |
| Median length | 3 |
| Mean length | 3.75 |
| Min length | 3 |

Characters and Unicode

|  |  |
| --- | --- |
| Total characters | 15 |
| Distinct characters | 11 |
| Distinct categories | 2 ? |
| Distinct scripts | 1 ? |
| Distinct blocks | 1 ? |

The Unicode Standard assigns character properties to each code point, which can be used to analyse textual variables.

Unique

|  |  |
| --- | --- |
| Unique | 2 ? |
| Unique (%) | 50.0% |

Sample

|  |  |
| --- | --- |
| 1st row | Pul |
| 2nd row | Pul |
| 3rd row | Dissem |
| 4th row | Ext |

#### Common Values

| Value | Count | Frequency (%) |
| --- | --- | --- |
| Pul | 2 | 50.0% |
| Dissem | 1 | 25.0% |
| Ext | 1 | 25.0% |

#### Length

xml version="1.0" encoding="utf-8" standalone="no"?2023-10-31T16:44:37.330713image/svg+xmlMatplotlib v3.6.0, https://matplotlib.org/ 

Histogram of lengths of the category

#### Common Values (Plot)

xml version="1.0" encoding="utf-8" standalone="no"?2023-10-31T16:44:37.491467image/svg+xmlMatplotlib v3.6.0, https://matplotlib.org/

| Value | Count | Frequency (%) |
| --- | --- | --- |
| pul | 2 | 50.0% |
| dissem | 1 | 25.0% |
| ext | 1 | 25.0% |

- Characters
- Categories
- Scripts
- Blocks

#### Most occurring characters

| Value | Count | Frequency (%) |
| --- | --- | --- |
| P | 2 | 13.3% |
| u | 2 | 13.3% |
| l | 2 | 13.3% |
| s | 2 | 13.3% |
| D | 1 | 6.7% |
| i | 1 | 6.7% |
| e | 1 | 6.7% |
| m | 1 | 6.7% |
| E | 1 | 6.7% |
| x | 1 | 6.7% |

#### Most occurring categories

| Value | Count | Frequency (%) |
| --- | --- | --- |
| Lowercase Letter | 11 | 73.3% |
| Uppercase Letter | 4 | 26.7% |

#### Most frequent character per category

##### *Lowercase Letter*

| Value | Count | Frequency (%) |
| --- | --- | --- |
| u | 2 | 18.2% |
| l | 2 | 18.2% |
| s | 2 | 18.2% |
| i | 1 | 9.1% |
| e | 1 | 9.1% |
| m | 1 | 9.1% |
| x | 1 | 9.1% |
| t | 1 | 9.1% |

##### *Uppercase Letter*

| Value | Count | Frequency (%) |
| --- | --- | --- |
| P | 2 | 50.0% |
| D | 1 | 25.0% |
| E | 1 | 25.0% |

#### Most occurring scripts

| Value | Count | Frequency (%) |
| --- | --- | --- |
| Latin | 15 | 100.0% |

#### Most frequent character per script

##### *Latin*

| Value | Count | Frequency (%) |
| --- | --- | --- |
| P | 2 | 13.3% |
| u | 2 | 13.3% |
| l | 2 | 13.3% |
| s | 2 | 13.3% |
| D | 1 | 6.7% |
| i | 1 | 6.7% |
| e | 1 | 6.7% |
| m | 1 | 6.7% |
| E | 1 | 6.7% |
| x | 1 | 6.7% |

#### Most occurring blocks

| Value | Count | Frequency (%) |
| --- | --- | --- |
| ASCII | 15 | 100.0% |

#### Most frequent character per block

##### *ASCII*

| Value | Count | Frequency (%) |
| --- | --- | --- |
| P | 2 | 13.3% |
| u | 2 | 13.3% |
| l | 2 | 13.3% |
| s | 2 | 13.3% |
| D | 1 | 6.7% |
| i | 1 | 6.7% |
| e | 1 | 6.7% |
| m | 1 | 6.7% |
| E | 1 | 6.7% |
| x | 1 | 6.7% |

descoberta  
Categorical

|  |  |
| --- | --- |
| Distinct | 3 |
| Distinct (%) | 75.0% |
| Missing | 0 |
| Missing (%) | 0.0% |
| Memory size | 64.0 B |

|  |  |
| --- | --- |
| Elucidacao Diagn. em Internacao | 2 |
| Demanda Ambulatorial | 1 |
| Urgencia / Emergencia | 1 |

More details

- Overview
- Categories
- Words
- Characters

Length

|  |  |
| --- | --- |
| Max length | 31 |
| Median length | 26 |
| Mean length | 25.75 |
| Min length | 20 |

Characters and Unicode

|  |  |
| --- | --- |
| Total characters | 103 |
| Distinct characters | 22 |
| Distinct categories | 4 ? |
| Distinct scripts | 2 ? |
| Distinct blocks | 1 ? |

The Unicode Standard assigns character properties to each code point, which can be used to analyse textual variables.

Unique

|  |  |
| --- | --- |
| Unique | 2 ? |
| Unique (%) | 50.0% |

Sample

|  |  |
| --- | --- |
| 1st row | Elucidacao Diagn. em Internacao |
| 2nd row | Demanda Ambulatorial |
| 3rd row | Urgencia / Emergencia |
| 4th row | Elucidacao Diagn. em Internacao |

#### Common Values

| Value | Count | Frequency (%) |
| --- | --- | --- |
| Elucidacao Diagn. em Internacao | 2 | 50.0% |
| Demanda Ambulatorial | 1 | 25.0% |
| Urgencia / Emergencia | 1 | 25.0% |

#### Length

xml version="1.0" encoding="utf-8" standalone="no"?2023-10-31T16:44:37.618405image/svg+xmlMatplotlib v3.6.0, https://matplotlib.org/ 

Histogram of lengths of the category

#### Common Values (Plot)

xml version="1.0" encoding="utf-8" standalone="no"?2023-10-31T16:44:37.769667image/svg+xmlMatplotlib v3.6.0, https://matplotlib.org/

| Value | Count | Frequency (%) |
| --- | --- | --- |
| elucidacao | 2 | 15.4% |
| diagn | 2 | 15.4% |
| em | 2 | 15.4% |
| internacao | 2 | 15.4% |
| demanda | 1 | 7.7% |
| ambulatorial | 1 | 7.7% |
| urgencia | 1 | 7.7% |
|  | 1 | 7.7% |
| emergencia | 1 | 7.7% |

- Characters
- Categories
- Scripts
- Blocks

#### Most occurring characters

| Value | Count | Frequency (%) |
| --- | --- | --- |
| a | 16 | 15.5% |
| n | 9 | 8.7% |
|  | 9 | 8.7% |
| e | 8 | 7.8% |
| c | 8 | 7.8% |
| i | 7 | 6.8% |
| r | 5 | 4.9% |
| o | 5 | 4.9% |
| m | 5 | 4.9% |
| g | 4 | 3.9% |
| Other values (12) | 27 | 26.2% |

#### Most occurring categories

| Value | Count | Frequency (%) |
| --- | --- | --- |
| Lowercase Letter | 81 | 78.6% |
| Uppercase Letter | 10 | 9.7% |
| Space Separator | 9 | 8.7% |
| Other Punctuation | 3 | 2.9% |

#### Most frequent character per category

##### *Lowercase Letter*

| Value | Count | Frequency (%) |
| --- | --- | --- |
| a | 16 | 19.8% |
| n | 9 | 11.1% |
| e | 8 | 9.9% |
| c | 8 | 9.9% |
| i | 7 | 8.6% |
| r | 5 | 6.2% |
| o | 5 | 6.2% |
| m | 5 | 6.2% |
| g | 4 | 4.9% |
| l | 4 | 4.9% |
| Other values (4) | 10 | 12.3% |

##### *Uppercase Letter*

| Value | Count | Frequency (%) |
| --- | --- | --- |
| E | 3 | 30.0% |
| D | 3 | 30.0% |
| I | 2 | 20.0% |
| A | 1 | 10.0% |
| U | 1 | 10.0% |

##### *Other Punctuation*

| Value | Count | Frequency (%) |
| --- | --- | --- |
| . | 2 | 66.7% |
| / | 1 | 33.3% |

##### *Space Separator*

| Value | Count | Frequency (%) |
| --- | --- | --- |
|  | 9 | 100.0% |

#### Most occurring scripts

| Value | Count | Frequency (%) |
| --- | --- | --- |
| Latin | 91 | 88.3% |
| Common | 12 | 11.7% |

#### Most frequent character per script

##### *Latin*

| Value | Count | Frequency (%) |
| --- | --- | --- |
| a | 16 | 17.6% |
| n | 9 | 9.9% |
| e | 8 | 8.8% |
| c | 8 | 8.8% |
| i | 7 | 7.7% |
| r | 5 | 5.5% |
| o | 5 | 5.5% |
| m | 5 | 5.5% |
| g | 4 | 4.4% |
| l | 4 | 4.4% |
| Other values (9) | 20 | 22.0% |

##### *Common*

| Value | Count | Frequency (%) |
| --- | --- | --- |
|  | 9 | 75.0% |
| . | 2 | 16.7% |
| / | 1 | 8.3% |

#### Most occurring blocks

| Value | Count | Frequency (%) |
| --- | --- | --- |
| ASCII | 103 | 100.0% |

#### Most frequent character per block

##### *ASCII*

| Value | Count | Frequency (%) |
| --- | --- | --- |
| a | 16 | 15.5% |
| n | 9 | 8.7% |
|  | 9 | 8.7% |
| e | 8 | 7.8% |
| c | 8 | 7.8% |
| i | 7 | 6.8% |
| r | 5 | 4.9% |
| o | 5 | 4.9% |
| m | 5 | 4.9% |
| g | 4 | 3.9% |
| Other values (12) | 27 | 26.2% |

bac  
Categorical

`HIGH CORRELATION`  `UNIFORM`

|  |  |
| --- | --- |
| Distinct | 2 |
| Distinct (%) | 50.0% |
| Missing | 0 |
| Missing (%) | 0.0% |
| Memory size | 64.0 B |

|  |  |
| --- | --- |
| N/realiz | 2 |
| Neg | 2 |

More details

- Overview
- Categories
- Words
- Characters

Length

|  |  |
| --- | --- |
| Max length | 8 |
| Median length | 5.5 |
| Mean length | 5.5 |
| Min length | 3 |

Characters and Unicode

|  |  |
| --- | --- |
| Total characters | 22 |
| Distinct characters | 9 |
| Distinct categories | 3 ? |
| Distinct scripts | 2 ? |
| Distinct blocks | 1 ? |

The Unicode Standard assigns character properties to each code point, which can be used to analyse textual variables.

Unique

|  |  |
| --- | --- |
| Unique | 0 ? |
| Unique (%) | 0.0% |

Sample

|  |  |
| --- | --- |
| 1st row | N/realiz |
| 2nd row | Neg |
| 3rd row | N/realiz |
| 4th row | Neg |

#### Common Values

| Value | Count | Frequency (%) |
| --- | --- | --- |
| N/realiz | 2 | 50.0% |
| Neg | 2 | 50.0% |

#### Length

xml version="1.0" encoding="utf-8" standalone="no"?2023-10-31T16:44:37.901405image/svg+xmlMatplotlib v3.6.0, https://matplotlib.org/ 

Histogram of lengths of the category

#### Common Values (Plot)

xml version="1.0" encoding="utf-8" standalone="no"?2023-10-31T16:44:38.045345image/svg+xmlMatplotlib v3.6.0, https://matplotlib.org/

| Value | Count | Frequency (%) |
| --- | --- | --- |
| n/realiz | 2 | 50.0% |
| neg | 2 | 50.0% |

- Characters
- Categories
- Scripts
- Blocks

#### Most occurring characters

| Value | Count | Frequency (%) |
| --- | --- | --- |
| N | 4 | 18.2% |
| e | 4 | 18.2% |
| / | 2 | 9.1% |
| r | 2 | 9.1% |
| a | 2 | 9.1% |
| l | 2 | 9.1% |
| i | 2 | 9.1% |
| z | 2 | 9.1% |
| g | 2 | 9.1% |

#### Most occurring categories

| Value | Count | Frequency (%) |
| --- | --- | --- |
| Lowercase Letter | 16 | 72.7% |
| Uppercase Letter | 4 | 18.2% |
| Other Punctuation | 2 | 9.1% |

#### Most frequent character per category

##### *Lowercase Letter*

| Value | Count | Frequency (%) |
| --- | --- | --- |
| e | 4 | 25.0% |
| r | 2 | 12.5% |
| a | 2 | 12.5% |
| l | 2 | 12.5% |
| i | 2 | 12.5% |
| z | 2 | 12.5% |
| g | 2 | 12.5% |

##### *Uppercase Letter*

| Value | Count | Frequency (%) |
| --- | --- | --- |
| N | 4 | 100.0% |

##### *Other Punctuation*

| Value | Count | Frequency (%) |
| --- | --- | --- |
| / | 2 | 100.0% |

#### Most occurring scripts

| Value | Count | Frequency (%) |
| --- | --- | --- |
| Latin | 20 | 90.9% |
| Common | 2 | 9.1% |

#### Most frequent character per script

##### *Latin*

| Value | Count | Frequency (%) |
| --- | --- | --- |
| N | 4 | 20.0% |
| e | 4 | 20.0% |
| r | 2 | 10.0% |
| a | 2 | 10.0% |
| l | 2 | 10.0% |
| i | 2 | 10.0% |
| z | 2 | 10.0% |
| g | 2 | 10.0% |

##### *Common*

| Value | Count | Frequency (%) |
| --- | --- | --- |
| / | 2 | 100.0% |

#### Most occurring blocks

| Value | Count | Frequency (%) |
| --- | --- | --- |
| ASCII | 22 | 100.0% |

#### Most frequent character per block

##### *ASCII*

| Value | Count | Frequency (%) |
| --- | --- | --- |
| N | 4 | 18.2% |
| e | 4 | 18.2% |
| / | 2 | 9.1% |
| r | 2 | 9.1% |
| a | 2 | 9.1% |
| l | 2 | 9.1% |
| i | 2 | 9.1% |
| z | 2 | 9.1% |
| g | 2 | 9.1% |

BACOUTRO  
Categorical

|  |  |
| --- | --- |
| Distinct | 3 |
| Distinct (%) | 75.0% |
| Missing | 0 |
| Missing (%) | 0.0% |
| Memory size | 64.0 B |

|  |  |
| --- | --- |
| N/realiz | 2 |
| Neg | 1 |
| Pos | 1 |

More details

- Overview
- Categories
- Words
- Characters

Length

|  |  |
| --- | --- |
| Max length | 8 |
| Median length | 5.5 |
| Mean length | 5.5 |
| Min length | 3 |

Characters and Unicode

|  |  |
| --- | --- |
| Total characters | 22 |
| Distinct characters | 12 |
| Distinct categories | 3 ? |
| Distinct scripts | 2 ? |
| Distinct blocks | 1 ? |

The Unicode Standard assigns character properties to each code point, which can be used to analyse textual variables.

Unique

|  |  |
| --- | --- |
| Unique | 2 ? |
| Unique (%) | 50.0% |

Sample

|  |  |
| --- | --- |
| 1st row | Neg |
| 2nd row | N/realiz |
| 3rd row | N/realiz |
| 4th row | Pos |

#### Common Values

| Value | Count | Frequency (%) |
| --- | --- | --- |
| N/realiz | 2 | 50.0% |
| Neg | 1 | 25.0% |
| Pos | 1 | 25.0% |

#### Length

xml version="1.0" encoding="utf-8" standalone="no"?2023-10-31T16:44:38.164270image/svg+xmlMatplotlib v3.6.0, https://matplotlib.org/ 

Histogram of lengths of the category

#### Common Values (Plot)

xml version="1.0" encoding="utf-8" standalone="no"?2023-10-31T16:44:38.314281image/svg+xmlMatplotlib v3.6.0, https://matplotlib.org/

| Value | Count | Frequency (%) |
| --- | --- | --- |
| n/realiz | 2 | 50.0% |
| neg | 1 | 25.0% |
| pos | 1 | 25.0% |

- Characters
- Categories
- Scripts
- Blocks

#### Most occurring characters

| Value | Count | Frequency (%) |
| --- | --- | --- |
| N | 3 | 13.6% |
| e | 3 | 13.6% |
| / | 2 | 9.1% |
| r | 2 | 9.1% |
| a | 2 | 9.1% |
| l | 2 | 9.1% |
| i | 2 | 9.1% |
| z | 2 | 9.1% |
| g | 1 | 4.5% |
| P | 1 | 4.5% |
| Other values (2) | 2 | 9.1% |

#### Most occurring categories

| Value | Count | Frequency (%) |
| --- | --- | --- |
| Lowercase Letter | 16 | 72.7% |
| Uppercase Letter | 4 | 18.2% |
| Other Punctuation | 2 | 9.1% |

#### Most frequent character per category

##### *Lowercase Letter*

| Value | Count | Frequency (%) |
| --- | --- | --- |
| e | 3 | 18.8% |
| r | 2 | 12.5% |
| a | 2 | 12.5% |
| l | 2 | 12.5% |
| i | 2 | 12.5% |
| z | 2 | 12.5% |
| g | 1 | 6.2% |
| o | 1 | 6.2% |
| s | 1 | 6.2% |

##### *Uppercase Letter*

| Value | Count | Frequency (%) |
| --- | --- | --- |
| N | 3 | 75.0% |
| P | 1 | 25.0% |

##### *Other Punctuation*

| Value | Count | Frequency (%) |
| --- | --- | --- |
| / | 2 | 100.0% |

#### Most occurring scripts

| Value | Count | Frequency (%) |
| --- | --- | --- |
| Latin | 20 | 90.9% |
| Common | 2 | 9.1% |

#### Most frequent character per script

##### *Latin*

| Value | Count | Frequency (%) |
| --- | --- | --- |
| N | 3 | 15.0% |
| e | 3 | 15.0% |
| r | 2 | 10.0% |
| a | 2 | 10.0% |
| l | 2 | 10.0% |
| i | 2 | 10.0% |
| z | 2 | 10.0% |
| g | 1 | 5.0% |
| P | 1 | 5.0% |
| o | 1 | 5.0% |

##### *Common*

| Value | Count | Frequency (%) |
| --- | --- | --- |
| / | 2 | 100.0% |

#### Most occurring blocks

| Value | Count | Frequency (%) |
| --- | --- | --- |
| ASCII | 22 | 100.0% |

#### Most frequent character per block

##### *ASCII*

| Value | Count | Frequency (%) |
| --- | --- | --- |
| N | 3 | 13.6% |
| e | 3 | 13.6% |
| / | 2 | 9.1% |
| r | 2 | 9.1% |
| a | 2 | 9.1% |
| l | 2 | 9.1% |
| i | 2 | 9.1% |
| z | 2 | 9.1% |
| g | 1 | 4.5% |
| P | 1 | 4.5% |
| Other values (2) | 2 | 9.1% |

cultEsc  
Categorical

|  |  |
| --- | --- |
| Distinct | 1 |
| Distinct (%) | 25.0% |
| Missing | 0 |
| Missing (%) | 0.0% |
| Memory size | 64.0 B |

|  |  |
| --- | --- |
| N/realiz | 4 |

More details

- Overview
- Categories
- Words
- Characters

Length

|  |  |
| --- | --- |
| Max length | 8 |
| Median length | 8 |
| Mean length | 8 |
| Min length | 8 |

Characters and Unicode

|  |  |
| --- | --- |
| Total characters | 32 |
| Distinct characters | 8 |
| Distinct categories | 3 ? |
| Distinct scripts | 2 ? |
| Distinct blocks | 1 ? |

The Unicode Standard assigns character properties to each code point, which can be used to analyse textual variables.

Unique

|  |  |
| --- | --- |
| Unique | 0 ? |
| Unique (%) | 0.0% |

Sample

|  |  |
| --- | --- |
| 1st row | N/realiz |
| 2nd row | N/realiz |
| 3rd row | N/realiz |
| 4th row | N/realiz |

#### Common Values

| Value | Count | Frequency (%) |
| --- | --- | --- |
| N/realiz | 4 | 100.0% |

#### Length

xml version="1.0" encoding="utf-8" standalone="no"?2023-10-31T16:44:38.437132image/svg+xmlMatplotlib v3.6.0, https://matplotlib.org/ 

Histogram of lengths of the category

#### Common Values (Plot)

xml version="1.0" encoding="utf-8" standalone="no"?2023-10-31T16:44:38.570976image/svg+xmlMatplotlib v3.6.0, https://matplotlib.org/

| Value | Count | Frequency (%) |
| --- | --- | --- |
| n/realiz | 4 | 100.0% |

- Characters
- Categories
- Scripts
- Blocks

#### Most occurring characters

| Value | Count | Frequency (%) |
| --- | --- | --- |
| N | 4 | 12.5% |
| / | 4 | 12.5% |
| r | 4 | 12.5% |
| e | 4 | 12.5% |
| a | 4 | 12.5% |
| l | 4 | 12.5% |
| i | 4 | 12.5% |
| z | 4 | 12.5% |

#### Most occurring categories

| Value | Count | Frequency (%) |
| --- | --- | --- |
| Lowercase Letter | 24 | 75.0% |
| Uppercase Letter | 4 | 12.5% |
| Other Punctuation | 4 | 12.5% |

#### Most frequent character per category

##### *Lowercase Letter*

| Value | Count | Frequency (%) |
| --- | --- | --- |
| r | 4 | 16.7% |
| e | 4 | 16.7% |
| a | 4 | 16.7% |
| l | 4 | 16.7% |
| i | 4 | 16.7% |
| z | 4 | 16.7% |

##### *Uppercase Letter*

| Value | Count | Frequency (%) |
| --- | --- | --- |
| N | 4 | 100.0% |

##### *Other Punctuation*

| Value | Count | Frequency (%) |
| --- | --- | --- |
| / | 4 | 100.0% |

#### Most occurring scripts

| Value | Count | Frequency (%) |
| --- | --- | --- |
| Latin | 28 | 87.5% |
| Common | 4 | 12.5% |

#### Most frequent character per script

##### *Latin*

| Value | Count | Frequency (%) |
| --- | --- | --- |
| N | 4 | 14.3% |
| r | 4 | 14.3% |
| e | 4 | 14.3% |
| a | 4 | 14.3% |
| l | 4 | 14.3% |
| i | 4 | 14.3% |
| z | 4 | 14.3% |

##### *Common*

| Value | Count | Frequency (%) |
| --- | --- | --- |
| / | 4 | 100.0% |

#### Most occurring blocks

| Value | Count | Frequency (%) |
| --- | --- | --- |
| ASCII | 32 | 100.0% |

#### Most frequent character per block

##### *ASCII*

| Value | Count | Frequency (%) |
| --- | --- | --- |
| N | 4 | 12.5% |
| / | 4 | 12.5% |
| r | 4 | 12.5% |
| e | 4 | 12.5% |
| a | 4 | 12.5% |
| l | 4 | 12.5% |
| i | 4 | 12.5% |
| z | 4 | 12.5% |

RX  
Categorical

|  |  |
| --- | --- |
| Distinct | 3 |
| Distinct (%) | 75.0% |
| Missing | 0 |
| Missing (%) | 0.0% |
| Memory size | 64.0 B |

|  |  |
| --- | --- |
| Susp TB | 2 |
| Normal | 1 |
| N/realiz | 1 |

More details

- Overview
- Categories
- Words
- Characters

Length

|  |  |
| --- | --- |
| Max length | 8 |
| Median length | 7.5 |
| Mean length | 7 |
| Min length | 6 |

Characters and Unicode

|  |  |
| --- | --- |
| Total characters | 28 |
| Distinct characters | 17 |
| Distinct categories | 4 ? |
| Distinct scripts | 2 ? |
| Distinct blocks | 1 ? |

The Unicode Standard assigns character properties to each code point, which can be used to analyse textual variables.

Unique

|  |  |
| --- | --- |
| Unique | 2 ? |
| Unique (%) | 50.0% |

Sample

|  |  |
| --- | --- |
| 1st row | Susp TB |
| 2nd row | Susp TB |
| 3rd row | Normal |
| 4th row | N/realiz |

#### Common Values

| Value | Count | Frequency (%) |
| --- | --- | --- |
| Susp TB | 2 | 50.0% |
| Normal | 1 | 25.0% |
| N/realiz | 1 | 25.0% |

#### Length

xml version="1.0" encoding="utf-8" standalone="no"?2023-10-31T16:44:38.688542image/svg+xmlMatplotlib v3.6.0, https://matplotlib.org/ 

Histogram of lengths of the category

#### Common Values (Plot)

xml version="1.0" encoding="utf-8" standalone="no"?2023-10-31T16:44:38.854586image/svg+xmlMatplotlib v3.6.0, https://matplotlib.org/

| Value | Count | Frequency (%) |
| --- | --- | --- |
| susp | 2 | 33.3% |
| tb | 2 | 33.3% |
| normal | 1 | 16.7% |
| n/realiz | 1 | 16.7% |

- Characters
- Categories
- Scripts
- Blocks

#### Most occurring characters

| Value | Count | Frequency (%) |
| --- | --- | --- |
| S | 2 | 7.1% |
| s | 2 | 7.1% |
| p | 2 | 7.1% |
|  | 2 | 7.1% |
| T | 2 | 7.1% |
| B | 2 | 7.1% |
| N | 2 | 7.1% |
| u | 2 | 7.1% |
| r | 2 | 7.1% |
| a | 2 | 7.1% |
| Other values (7) | 8 | 28.6% |

#### Most occurring categories

| Value | Count | Frequency (%) |
| --- | --- | --- |
| Lowercase Letter | 17 | 60.7% |
| Uppercase Letter | 8 | 28.6% |
| Space Separator | 2 | 7.1% |
| Other Punctuation | 1 | 3.6% |

#### Most frequent character per category

##### *Lowercase Letter*

| Value | Count | Frequency (%) |
| --- | --- | --- |
| s | 2 | 11.8% |
| p | 2 | 11.8% |
| u | 2 | 11.8% |
| r | 2 | 11.8% |
| a | 2 | 11.8% |
| l | 2 | 11.8% |
| e | 1 | 5.9% |
| i | 1 | 5.9% |
| o | 1 | 5.9% |
| m | 1 | 5.9% |

##### *Uppercase Letter*

| Value | Count | Frequency (%) |
| --- | --- | --- |
| S | 2 | 25.0% |
| T | 2 | 25.0% |
| B | 2 | 25.0% |
| N | 2 | 25.0% |

##### *Space Separator*

| Value | Count | Frequency (%) |
| --- | --- | --- |
|  | 2 | 100.0% |

##### *Other Punctuation*

| Value | Count | Frequency (%) |
| --- | --- | --- |
| / | 1 | 100.0% |

#### Most occurring scripts

| Value | Count | Frequency (%) |
| --- | --- | --- |
| Latin | 25 | 89.3% |
| Common | 3 | 10.7% |

#### Most frequent character per script

##### *Latin*

| Value | Count | Frequency (%) |
| --- | --- | --- |
| S | 2 | 8.0% |
| s | 2 | 8.0% |
| p | 2 | 8.0% |
| T | 2 | 8.0% |
| B | 2 | 8.0% |
| N | 2 | 8.0% |
| u | 2 | 8.0% |
| r | 2 | 8.0% |
| a | 2 | 8.0% |
| l | 2 | 8.0% |
| Other values (5) | 5 | 20.0% |

##### *Common*

| Value | Count | Frequency (%) |
| --- | --- | --- |
|  | 2 | 66.7% |
| / | 1 | 33.3% |

#### Most occurring blocks

| Value | Count | Frequency (%) |
| --- | --- | --- |
| ASCII | 28 | 100.0% |

#### Most frequent character per block

##### *ASCII*

| Value | Count | Frequency (%) |
| --- | --- | --- |
| S | 2 | 7.1% |
| s | 2 | 7.1% |
| p | 2 | 7.1% |
|  | 2 | 7.1% |
| T | 2 | 7.1% |
| B | 2 | 7.1% |
| N | 2 | 7.1% |
| u | 2 | 7.1% |
| r | 2 | 7.1% |
| a | 2 | 7.1% |
| Other values (7) | 8 | 28.6% |

NECROP  
Categorical

|  |  |
| --- | --- |
| Distinct | 1 |
| Distinct (%) | 25.0% |
| Missing | 0 |
| Missing (%) | 0.0% |
| Memory size | 64.0 B |

|  |  |
| --- | --- |
| N/realiz | 4 |

More details

- Overview
- Categories
- Words
- Characters

Length

|  |  |
| --- | --- |
| Max length | 8 |
| Median length | 8 |
| Mean length | 8 |
| Min length | 8 |

Characters and Unicode

|  |  |
| --- | --- |
| Total characters | 32 |
| Distinct characters | 8 |
| Distinct categories | 3 ? |
| Distinct scripts | 2 ? |
| Distinct blocks | 1 ? |

The Unicode Standard assigns character properties to each code point, which can be used to analyse textual variables.

Unique

|  |  |
| --- | --- |
| Unique | 0 ? |
| Unique (%) | 0.0% |

Sample

|  |  |
| --- | --- |
| 1st row | N/realiz |
| 2nd row | N/realiz |
| 3rd row | N/realiz |
| 4th row | N/realiz |

#### Common Values

| Value | Count | Frequency (%) |
| --- | --- | --- |
| N/realiz | 4 | 100.0% |

#### Length

xml version="1.0" encoding="utf-8" standalone="no"?2023-10-31T16:44:38.977117image/svg+xmlMatplotlib v3.6.0, https://matplotlib.org/ 

Histogram of lengths of the category

#### Common Values (Plot)

xml version="1.0" encoding="utf-8" standalone="no"?2023-10-31T16:44:39.110209image/svg+xmlMatplotlib v3.6.0, https://matplotlib.org/

| Value | Count | Frequency (%) |
| --- | --- | --- |
| n/realiz | 4 | 100.0% |

- Characters
- Categories
- Scripts
- Blocks

#### Most occurring characters

| Value | Count | Frequency (%) |
| --- | --- | --- |
| N | 4 | 12.5% |
| / | 4 | 12.5% |
| r | 4 | 12.5% |
| e | 4 | 12.5% |
| a | 4 | 12.5% |
| l | 4 | 12.5% |
| i | 4 | 12.5% |
| z | 4 | 12.5% |

#### Most occurring categories

| Value | Count | Frequency (%) |
| --- | --- | --- |
| Lowercase Letter | 24 | 75.0% |
| Uppercase Letter | 4 | 12.5% |
| Other Punctuation | 4 | 12.5% |

#### Most frequent character per category

##### *Lowercase Letter*

| Value | Count | Frequency (%) |
| --- | --- | --- |
| r | 4 | 16.7% |
| e | 4 | 16.7% |
| a | 4 | 16.7% |
| l | 4 | 16.7% |
| i | 4 | 16.7% |
| z | 4 | 16.7% |

##### *Uppercase Letter*

| Value | Count | Frequency (%) |
| --- | --- | --- |
| N | 4 | 100.0% |

##### *Other Punctuation*

| Value | Count | Frequency (%) |
| --- | --- | --- |
| / | 4 | 100.0% |

#### Most occurring scripts

| Value | Count | Frequency (%) |
| --- | --- | --- |
| Latin | 28 | 87.5% |
| Common | 4 | 12.5% |

#### Most frequent character per script

##### *Latin*

| Value | Count | Frequency (%) |
| --- | --- | --- |
| N | 4 | 14.3% |
| r | 4 | 14.3% |
| e | 4 | 14.3% |
| a | 4 | 14.3% |
| l | 4 | 14.3% |
| i | 4 | 14.3% |
| z | 4 | 14.3% |

##### *Common*

| Value | Count | Frequency (%) |
| --- | --- | --- |
| / | 4 | 100.0% |

#### Most occurring blocks

| Value | Count | Frequency (%) |
| --- | --- | --- |
| ASCII | 32 | 100.0% |

#### Most frequent character per block

##### *ASCII*

| Value | Count | Frequency (%) |
| --- | --- | --- |
| N | 4 | 12.5% |
| / | 4 | 12.5% |
| r | 4 | 12.5% |
| e | 4 | 12.5% |
| a | 4 | 12.5% |
| l | 4 | 12.5% |
| i | 4 | 12.5% |
| z | 4 | 12.5% |

hiv  
Categorical

|  |  |
| --- | --- |
| Distinct | 2 |
| Distinct (%) | 50.0% |
| Missing | 0 |
| Missing (%) | 0.0% |
| Memory size | 64.0 B |

|  |  |
| --- | --- |
| Neg | 3 |
| Pos | 1 |

More details

- Overview
- Categories
- Words
- Characters

Length

|  |  |
| --- | --- |
| Max length | 3 |
| Median length | 3 |
| Mean length | 3 |
| Min length | 3 |

Characters and Unicode

|  |  |
| --- | --- |
| Total characters | 12 |
| Distinct characters | 6 |
| Distinct categories | 2 ? |
| Distinct scripts | 1 ? |
| Distinct blocks | 1 ? |

The Unicode Standard assigns character properties to each code point, which can be used to analyse textual variables.

Unique

|  |  |
| --- | --- |
| Unique | 1 ? |
| Unique (%) | 25.0% |

Sample

|  |  |
| --- | --- |
| 1st row | Neg |
| 2nd row | Neg |
| 3rd row | Pos |
| 4th row | Neg |

#### Common Values

| Value | Count | Frequency (%) |
| --- | --- | --- |
| Neg | 3 | 75.0% |
| Pos | 1 | 25.0% |

#### Length

xml version="1.0" encoding="utf-8" standalone="no"?2023-10-31T16:44:39.217992image/svg+xmlMatplotlib v3.6.0, https://matplotlib.org/ 

Histogram of lengths of the category

#### Common Values (Plot)

xml version="1.0" encoding="utf-8" standalone="no"?2023-10-31T16:44:39.358011image/svg+xmlMatplotlib v3.6.0, https://matplotlib.org/

| Value | Count | Frequency (%) |
| --- | --- | --- |
| neg | 3 | 75.0% |
| pos | 1 | 25.0% |

- Characters
- Categories
- Scripts
- Blocks

#### Most occurring characters

| Value | Count | Frequency (%) |
| --- | --- | --- |
| N | 3 | 25.0% |
| e | 3 | 25.0% |
| g | 3 | 25.0% |
| P | 1 | 8.3% |
| o | 1 | 8.3% |
| s | 1 | 8.3% |

#### Most occurring categories

| Value | Count | Frequency (%) |
| --- | --- | --- |
| Lowercase Letter | 8 | 66.7% |
| Uppercase Letter | 4 | 33.3% |

#### Most frequent character per category

##### *Lowercase Letter*

| Value | Count | Frequency (%) |
| --- | --- | --- |
| e | 3 | 37.5% |
| g | 3 | 37.5% |
| o | 1 | 12.5% |
| s | 1 | 12.5% |

##### *Uppercase Letter*

| Value | Count | Frequency (%) |
| --- | --- | --- |
| N | 3 | 75.0% |
| P | 1 | 25.0% |

#### Most occurring scripts

| Value | Count | Frequency (%) |
| --- | --- | --- |
| Latin | 12 | 100.0% |

#### Most frequent character per script

##### *Latin*

| Value | Count | Frequency (%) |
| --- | --- | --- |
| N | 3 | 25.0% |
| e | 3 | 25.0% |
| g | 3 | 25.0% |
| P | 1 | 8.3% |
| o | 1 | 8.3% |
| s | 1 | 8.3% |

#### Most occurring blocks

| Value | Count | Frequency (%) |
| --- | --- | --- |
| ASCII | 12 | 100.0% |

#### Most frequent character per block

##### *ASCII*

| Value | Count | Frequency (%) |
| --- | --- | --- |
| N | 3 | 25.0% |
| e | 3 | 25.0% |
| g | 3 | 25.0% |
| P | 1 | 8.3% |
| o | 1 | 8.3% |
| s | 1 | 8.3% |

aids  
Boolean

|  |  |
| --- | --- |
| Distinct | 1 |
| Distinct (%) | 25.0% |
| Missing | 0 |
| Missing (%) | 0.0% |
| Memory size | 36.0 B |

|  |  |
| --- | --- |
| False | 4 |

More details

- Common Values (Table)
- Common Values (Plot)

| Value | Count | Frequency (%) |
| --- | --- | --- |
| False | 4 | 100.0% |

xml version="1.0" encoding="utf-8" standalone="no"?2023-10-31T16:44:39.489603image/svg+xmlMatplotlib v3.6.0, https://matplotlib.org/

DIABETES  
Boolean

|  |  |
| --- | --- |
| Distinct | 1 |
| Distinct (%) | 25.0% |
| Missing | 0 |
| Missing (%) | 0.0% |
| Memory size | 36.0 B |

|  |  |
| --- | --- |
| False | 4 |

More details

- Common Values (Table)
- Common Values (Plot)

| Value | Count | Frequency (%) |
| --- | --- | --- |
| False | 4 | 100.0% |

xml version="1.0" encoding="utf-8" standalone="no"?2023-10-31T16:44:39.610372image/svg+xmlMatplotlib v3.6.0, https://matplotlib.org/

ALCOOLISMO  
Categorical

`HIGH CORRELATION`  `UNIFORM`

|  |  |
| --- | --- |
| Distinct | 2 |
| Distinct (%) | 50.0% |
| Missing | 0 |
| Missing (%) | 0.0% |
| Memory size | 64.0 B |

|  |  |
| --- | --- |
| N | 2 |
| S | 2 |

More details

- Overview
- Categories
- Words
- Characters

Length

|  |  |
| --- | --- |
| Max length | 1 |
| Median length | 1 |
| Mean length | 1 |
| Min length | 1 |

Characters and Unicode

|  |  |
| --- | --- |
| Total characters | 4 |
| Distinct characters | 2 |
| Distinct categories | 1 ? |
| Distinct scripts | 1 ? |
| Distinct blocks | 1 ? |

The Unicode Standard assigns character properties to each code point, which can be used to analyse textual variables.

Unique

|  |  |
| --- | --- |
| Unique | 0 ? |
| Unique (%) | 0.0% |

Sample

|  |  |
| --- | --- |
| 1st row | N |
| 2nd row | S |
| 3rd row | N |
| 4th row | S |

#### Common Values

| Value | Count | Frequency (%) |
| --- | --- | --- |
| N | 2 | 50.0% |
| S | 2 | 50.0% |

#### Length

xml version="1.0" encoding="utf-8" standalone="no"?2023-10-31T16:44:39.720149image/svg+xmlMatplotlib v3.6.0, https://matplotlib.org/ 

Histogram of lengths of the category

#### Common Values (Plot)

xml version="1.0" encoding="utf-8" standalone="no"?2023-10-31T16:44:39.857757image/svg+xmlMatplotlib v3.6.0, https://matplotlib.org/

| Value | Count | Frequency (%) |
| --- | --- | --- |
| n | 2 | 50.0% |
| s | 2 | 50.0% |

- Characters
- Categories
- Scripts
- Blocks

#### Most occurring characters

| Value | Count | Frequency (%) |
| --- | --- | --- |
| N | 2 | 50.0% |
| S | 2 | 50.0% |

#### Most occurring categories

| Value | Count | Frequency (%) |
| --- | --- | --- |
| Uppercase Letter | 4 | 100.0% |

#### Most frequent character per category

##### *Uppercase Letter*

| Value | Count | Frequency (%) |
| --- | --- | --- |
| N | 2 | 50.0% |
| S | 2 | 50.0% |

#### Most occurring scripts

| Value | Count | Frequency (%) |
| --- | --- | --- |
| Latin | 4 | 100.0% |

#### Most frequent character per script

##### *Latin*

| Value | Count | Frequency (%) |
| --- | --- | --- |
| N | 2 | 50.0% |
| S | 2 | 50.0% |

#### Most occurring blocks

| Value | Count | Frequency (%) |
| --- | --- | --- |
| ASCII | 4 | 100.0% |

#### Most frequent character per block

##### *ASCII*

| Value | Count | Frequency (%) |
| --- | --- | --- |
| N | 2 | 50.0% |
| S | 2 | 50.0% |

MENTAL  
Boolean

|  |  |
| --- | --- |
| Distinct | 1 |
| Distinct (%) | 25.0% |
| Missing | 0 |
| Missing (%) | 0.0% |
| Memory size | 36.0 B |

|  |  |
| --- | --- |
| False | 4 |

More details

- Common Values (Table)
- Common Values (Plot)

| Value | Count | Frequency (%) |
| --- | --- | --- |
| False | 4 | 100.0% |

xml version="1.0" encoding="utf-8" standalone="no"?2023-10-31T16:44:39.987603image/svg+xmlMatplotlib v3.6.0, https://matplotlib.org/

DROGADICAO  
Categorical

`HIGH CORRELATION`  `UNIFORM`

|  |  |
| --- | --- |
| Distinct | 2 |
| Distinct (%) | 50.0% |
| Missing | 0 |
| Missing (%) | 0.0% |
| Memory size | 64.0 B |

|  |  |
| --- | --- |
| N | 2 |
| S | 2 |

More details

- Overview
- Categories
- Words
- Characters

Length

|  |  |
| --- | --- |
| Max length | 1 |
| Median length | 1 |
| Mean length | 1 |
| Min length | 1 |

Characters and Unicode

|  |  |
| --- | --- |
| Total characters | 4 |
| Distinct characters | 2 |
| Distinct categories | 1 ? |
| Distinct scripts | 1 ? |
| Distinct blocks | 1 ? |

The Unicode Standard assigns character properties to each code point, which can be used to analyse textual variables.

Unique

|  |  |
| --- | --- |
| Unique | 0 ? |
| Unique (%) | 0.0% |

Sample

|  |  |
| --- | --- |
| 1st row | N |
| 2nd row | S |
| 3rd row | S |
| 4th row | N |

#### Common Values

| Value | Count | Frequency (%) |
| --- | --- | --- |
| N | 2 | 50.0% |
| S | 2 | 50.0% |

#### Length

xml version="1.0" encoding="utf-8" standalone="no"?2023-10-31T16:44:40.092239image/svg+xmlMatplotlib v3.6.0, https://matplotlib.org/ 

Histogram of lengths of the category

#### Common Values (Plot)

xml version="1.0" encoding="utf-8" standalone="no"?2023-10-31T16:44:40.230785image/svg+xmlMatplotlib v3.6.0, https://matplotlib.org/

| Value | Count | Frequency (%) |
| --- | --- | --- |
| n | 2 | 50.0% |
| s | 2 | 50.0% |

- Characters
- Categories
- Scripts
- Blocks

#### Most occurring characters

| Value | Count | Frequency (%) |
| --- | --- | --- |
| N | 2 | 50.0% |
| S | 2 | 50.0% |

#### Most occurring categories

| Value | Count | Frequency (%) |
| --- | --- | --- |
| Uppercase Letter | 4 | 100.0% |

#### Most frequent character per category

##### *Uppercase Letter*

| Value | Count | Frequency (%) |
| --- | --- | --- |
| N | 2 | 50.0% |
| S | 2 | 50.0% |

#### Most occurring scripts

| Value | Count | Frequency (%) |
| --- | --- | --- |
| Latin | 4 | 100.0% |

#### Most frequent character per script

##### *Latin*

| Value | Count | Frequency (%) |
| --- | --- | --- |
| N | 2 | 50.0% |
| S | 2 | 50.0% |

#### Most occurring blocks

| Value | Count | Frequency (%) |
| --- | --- | --- |
| ASCII | 4 | 100.0% |

#### Most frequent character per block

##### *ASCII*

| Value | Count | Frequency (%) |
| --- | --- | --- |
| N | 2 | 50.0% |
| S | 2 | 50.0% |

TABAGISMO  
Categorical

|  |  |
| --- | --- |
| Distinct | 2 |
| Distinct (%) | 50.0% |
| Missing | 0 |
| Missing (%) | 0.0% |
| Memory size | 64.0 B |

|  |  |
| --- | --- |
| N | 3 |
| S | 1 |

More details

- Overview
- Categories
- Words
- Characters

Length

|  |  |
| --- | --- |
| Max length | 1 |
| Median length | 1 |
| Mean length | 1 |
| Min length | 1 |

Characters and Unicode

|  |  |
| --- | --- |
| Total characters | 4 |
| Distinct characters | 2 |
| Distinct categories | 1 ? |
| Distinct scripts | 1 ? |
| Distinct blocks | 1 ? |

The Unicode Standard assigns character properties to each code point, which can be used to analyse textual variables.

Unique

|  |  |
| --- | --- |
| Unique | 1 ? |
| Unique (%) | 25.0% |

Sample

|  |  |
| --- | --- |
| 1st row | N |
| 2nd row | S |
| 3rd row | N |
| 4th row | N |

#### Common Values

| Value | Count | Frequency (%) |
| --- | --- | --- |
| N | 3 | 75.0% |
| S | 1 | 25.0% |

#### Length

xml version="1.0" encoding="utf-8" standalone="no"?2023-10-31T16:44:40.842239image/svg+xmlMatplotlib v3.6.0, https://matplotlib.org/ 

Histogram of lengths of the category

#### Common Values (Plot)

xml version="1.0" encoding="utf-8" standalone="no"?2023-10-31T16:44:40.982656image/svg+xmlMatplotlib v3.6.0, https://matplotlib.org/

| Value | Count | Frequency (%) |
| --- | --- | --- |
| n | 3 | 75.0% |
| s | 1 | 25.0% |

- Characters
- Categories
- Scripts
- Blocks

#### Most occurring characters

| Value | Count | Frequency (%) |
| --- | --- | --- |
| N | 3 | 75.0% |
| S | 1 | 25.0% |

#### Most occurring categories

| Value | Count | Frequency (%) |
| --- | --- | --- |
| Uppercase Letter | 4 | 100.0% |

#### Most frequent character per category

##### *Uppercase Letter*

| Value | Count | Frequency (%) |
| --- | --- | --- |
| N | 3 | 75.0% |
| S | 1 | 25.0% |

#### Most occurring scripts

| Value | Count | Frequency (%) |
| --- | --- | --- |
| Latin | 4 | 100.0% |

#### Most frequent character per script

##### *Latin*

| Value | Count | Frequency (%) |
| --- | --- | --- |
| N | 3 | 75.0% |
| S | 1 | 25.0% |

#### Most occurring blocks

| Value | Count | Frequency (%) |
| --- | --- | --- |
| ASCII | 4 | 100.0% |

#### Most frequent character per block

##### *ASCII*

| Value | Count | Frequency (%) |
| --- | --- | --- |
| N | 3 | 75.0% |
| S | 1 | 25.0% |

motMudEsquema  
Categorical

|  |  |
| --- | --- |
| Distinct | 1 |
| Distinct (%) | 25.0% |
| Missing | 0 |
| Missing (%) | 0.0% |
| Memory size | 64.0 B |

|  |  |
| --- | --- |
| Nulo | 4 |

More details

- Overview
- Categories
- Words
- Characters

Length

|  |  |
| --- | --- |
| Max length | 4 |
| Median length | 4 |
| Mean length | 4 |
| Min length | 4 |

Characters and Unicode

|  |  |
| --- | --- |
| Total characters | 16 |
| Distinct characters | 4 |
| Distinct categories | 2 ? |
| Distinct scripts | 1 ? |
| Distinct blocks | 1 ? |

The Unicode Standard assigns character properties to each code point, which can be used to analyse textual variables.

Unique

|  |  |
| --- | --- |
| Unique | 0 ? |
| Unique (%) | 0.0% |

Sample

|  |  |
| --- | --- |
| 1st row | Nulo |
| 2nd row | Nulo |
| 3rd row | Nulo |
| 4th row | Nulo |

#### Common Values

| Value | Count | Frequency (%) |
| --- | --- | --- |
| Nulo | 4 | 100.0% |

#### Length

xml version="1.0" encoding="utf-8" standalone="no"?2023-10-31T16:44:41.097539image/svg+xmlMatplotlib v3.6.0, https://matplotlib.org/ 

Histogram of lengths of the category

#### Common Values (Plot)

xml version="1.0" encoding="utf-8" standalone="no"?2023-10-31T16:44:41.230231image/svg+xmlMatplotlib v3.6.0, https://matplotlib.org/

| Value | Count | Frequency (%) |
| --- | --- | --- |
| nulo | 4 | 100.0% |

- Characters
- Categories
- Scripts
- Blocks

#### Most occurring characters

| Value | Count | Frequency (%) |
| --- | --- | --- |
| N | 4 | 25.0% |
| u | 4 | 25.0% |
| l | 4 | 25.0% |
| o | 4 | 25.0% |

#### Most occurring categories

| Value | Count | Frequency (%) |
| --- | --- | --- |
| Lowercase Letter | 12 | 75.0% |
| Uppercase Letter | 4 | 25.0% |

#### Most frequent character per category

##### *Lowercase Letter*

| Value | Count | Frequency (%) |
| --- | --- | --- |
| u | 4 | 33.3% |
| l | 4 | 33.3% |
| o | 4 | 33.3% |

##### *Uppercase Letter*

| Value | Count | Frequency (%) |
| --- | --- | --- |
| N | 4 | 100.0% |

#### Most occurring scripts

| Value | Count | Frequency (%) |
| --- | --- | --- |
| Latin | 16 | 100.0% |

#### Most frequent character per script

##### *Latin*

| Value | Count | Frequency (%) |
| --- | --- | --- |
| N | 4 | 25.0% |
| u | 4 | 25.0% |
| l | 4 | 25.0% |
| o | 4 | 25.0% |

#### Most occurring blocks

| Value | Count | Frequency (%) |
| --- | --- | --- |
| ASCII | 16 | 100.0% |

#### Most frequent character per block

##### *ASCII*

| Value | Count | Frequency (%) |
| --- | --- | --- |
| N | 4 | 25.0% |
| u | 4 | 25.0% |
| l | 4 | 25.0% |
| o | 4 | 25.0% |

tipoTrat  
Categorical

|  |  |
| --- | --- |
| Distinct | 1 |
| Distinct (%) | 25.0% |
| Missing | 0 |
| Missing (%) | 0.0% |
| Memory size | 64.0 B |

|  |  |
| --- | --- |
| Supervisionado | 4 |

More details

- Overview
- Categories
- Words
- Characters

Length

|  |  |
| --- | --- |
| Max length | 14 |
| Median length | 14 |
| Mean length | 14 |
| Min length | 14 |

Characters and Unicode

|  |  |
| --- | --- |
| Total characters | 56 |
| Distinct characters | 12 |
| Distinct categories | 2 ? |
| Distinct scripts | 1 ? |
| Distinct blocks | 1 ? |

The Unicode Standard assigns character properties to each code point, which can be used to analyse textual variables.

Unique

|  |  |
| --- | --- |
| Unique | 0 ? |
| Unique (%) | 0.0% |

Sample

|  |  |
| --- | --- |
| 1st row | Supervisionado |
| 2nd row | Supervisionado |
| 3rd row | Supervisionado |
| 4th row | Supervisionado |

#### Common Values

| Value | Count | Frequency (%) |
| --- | --- | --- |
| Supervisionado | 4 | 100.0% |

#### Length

xml version="1.0" encoding="utf-8" standalone="no"?2023-10-31T16:44:41.338132image/svg+xmlMatplotlib v3.6.0, https://matplotlib.org/ 

Histogram of lengths of the category

#### Common Values (Plot)

xml version="1.0" encoding="utf-8" standalone="no"?2023-10-31T16:44:41.474094image/svg+xmlMatplotlib v3.6.0, https://matplotlib.org/

| Value | Count | Frequency (%) |
| --- | --- | --- |
| supervisionado | 4 | 100.0% |

- Characters
- Categories
- Scripts
- Blocks

#### Most occurring characters

| Value | Count | Frequency (%) |
| --- | --- | --- |
| i | 8 | 14.3% |
| o | 8 | 14.3% |
| S | 4 | 7.1% |
| u | 4 | 7.1% |
| p | 4 | 7.1% |
| e | 4 | 7.1% |
| r | 4 | 7.1% |
| v | 4 | 7.1% |
| s | 4 | 7.1% |
| n | 4 | 7.1% |
| Other values (2) | 8 | 14.3% |

#### Most occurring categories

| Value | Count | Frequency (%) |
| --- | --- | --- |
| Lowercase Letter | 52 | 92.9% |
| Uppercase Letter | 4 | 7.1% |

#### Most frequent character per category

##### *Lowercase Letter*

| Value | Count | Frequency (%) |
| --- | --- | --- |
| i | 8 | 15.4% |
| o | 8 | 15.4% |
| u | 4 | 7.7% |
| p | 4 | 7.7% |
| e | 4 | 7.7% |
| r | 4 | 7.7% |
| v | 4 | 7.7% |
| s | 4 | 7.7% |
| n | 4 | 7.7% |
| a | 4 | 7.7% |

##### *Uppercase Letter*

| Value | Count | Frequency (%) |
| --- | --- | --- |
| S | 4 | 100.0% |

#### Most occurring scripts

| Value | Count | Frequency (%) |
| --- | --- | --- |
| Latin | 56 | 100.0% |

#### Most frequent character per script

##### *Latin*

| Value | Count | Frequency (%) |
| --- | --- | --- |
| i | 8 | 14.3% |
| o | 8 | 14.3% |
| S | 4 | 7.1% |
| u | 4 | 7.1% |
| p | 4 | 7.1% |
| e | 4 | 7.1% |
| r | 4 | 7.1% |
| v | 4 | 7.1% |
| s | 4 | 7.1% |
| n | 4 | 7.1% |
| Other values (2) | 8 | 14.3% |

#### Most occurring blocks

| Value | Count | Frequency (%) |
| --- | --- | --- |
| ASCII | 56 | 100.0% |

#### Most frequent character per block

##### *ASCII*

| Value | Count | Frequency (%) |
| --- | --- | --- |
| i | 8 | 14.3% |
| o | 8 | 14.3% |
| S | 4 | 7.1% |
| u | 4 | 7.1% |
| p | 4 | 7.1% |
| e | 4 | 7.1% |
| r | 4 | 7.1% |
| v | 4 | 7.1% |
| s | 4 | 7.1% |
| n | 4 | 7.1% |
| Other values (2) | 8 | 14.3% |

idade  
Categorical

|  |  |
| --- | --- |
| Distinct | 2 |
| Distinct (%) | 50.0% |
| Missing | 0 |
| Missing (%) | 0.0% |
| Memory size | 64.0 B |

|  |  |
| --- | --- |
| 23\_39 | 3 |
| Mais de 54 | 1 |

More details

- Overview
- Categories
- Words
- Characters

Length

|  |  |
| --- | --- |
| Max length | 10 |
| Median length | 5 |
| Mean length | 6.25 |
| Min length | 5 |

Characters and Unicode

|  |  |
| --- | --- |
| Total characters | 25 |
| Distinct characters | 13 |
| Distinct categories | 5 ? |
| Distinct scripts | 2 ? |
| Distinct blocks | 1 ? |

The Unicode Standard assigns character properties to each code point, which can be used to analyse textual variables.

Unique

|  |  |
| --- | --- |
| Unique | 1 ? |
| Unique (%) | 25.0% |

Sample

|  |  |
| --- | --- |
| 1st row | Mais de 54 |
| 2nd row | 23\_39 |
| 3rd row | 23\_39 |
| 4th row | 23\_39 |

#### Common Values

| Value | Count | Frequency (%) |
| --- | --- | --- |
| 23\_39 | 3 | 75.0% |
| Mais de 54 | 1 | 25.0% |

#### Length

xml version="1.0" encoding="utf-8" standalone="no"?2023-10-31T16:44:41.589330image/svg+xmlMatplotlib v3.6.0, https://matplotlib.org/ 

Histogram of lengths of the category

#### Common Values (Plot)

xml version="1.0" encoding="utf-8" standalone="no"?2023-10-31T16:44:41.737835image/svg+xmlMatplotlib v3.6.0, https://matplotlib.org/

| Value | Count | Frequency (%) |
| --- | --- | --- |
| 23\_39 | 3 | 50.0% |
| mais | 1 | 16.7% |
| de | 1 | 16.7% |
| 54 | 1 | 16.7% |

- Characters
- Categories
- Scripts
- Blocks

#### Most occurring characters

| Value | Count | Frequency (%) |
| --- | --- | --- |
| 3 | 6 | 24.0% |
| 2 | 3 | 12.0% |
| \_ | 3 | 12.0% |
| 9 | 3 | 12.0% |
|  | 2 | 8.0% |
| M | 1 | 4.0% |
| a | 1 | 4.0% |
| i | 1 | 4.0% |
| s | 1 | 4.0% |
| d | 1 | 4.0% |
| Other values (3) | 3 | 12.0% |

#### Most occurring categories

| Value | Count | Frequency (%) |
| --- | --- | --- |
| Decimal Number | 14 | 56.0% |
| Lowercase Letter | 5 | 20.0% |
| Connector Punctuation | 3 | 12.0% |
| Space Separator | 2 | 8.0% |
| Uppercase Letter | 1 | 4.0% |

#### Most frequent character per category

##### *Decimal Number*

| Value | Count | Frequency (%) |
| --- | --- | --- |
| 3 | 6 | 42.9% |
| 2 | 3 | 21.4% |
| 9 | 3 | 21.4% |
| 5 | 1 | 7.1% |
| 4 | 1 | 7.1% |

##### *Lowercase Letter*

| Value | Count | Frequency (%) |
| --- | --- | --- |
| a | 1 | 20.0% |
| i | 1 | 20.0% |
| s | 1 | 20.0% |
| d | 1 | 20.0% |
| e | 1 | 20.0% |

##### *Connector Punctuation*

| Value | Count | Frequency (%) |
| --- | --- | --- |
| \_ | 3 | 100.0% |

##### *Space Separator*

| Value | Count | Frequency (%) |
| --- | --- | --- |
|  | 2 | 100.0% |

##### *Uppercase Letter*

| Value | Count | Frequency (%) |
| --- | --- | --- |
| M | 1 | 100.0% |

#### Most occurring scripts

| Value | Count | Frequency (%) |
| --- | --- | --- |
| Common | 19 | 76.0% |
| Latin | 6 | 24.0% |

#### Most frequent character per script

##### *Common*

| Value | Count | Frequency (%) |
| --- | --- | --- |
| 3 | 6 | 31.6% |
| 2 | 3 | 15.8% |
| \_ | 3 | 15.8% |
| 9 | 3 | 15.8% |
|  | 2 | 10.5% |
| 5 | 1 | 5.3% |
| 4 | 1 | 5.3% |

##### *Latin*

| Value | Count | Frequency (%) |
| --- | --- | --- |
| M | 1 | 16.7% |
| a | 1 | 16.7% |
| i | 1 | 16.7% |
| s | 1 | 16.7% |
| d | 1 | 16.7% |
| e | 1 | 16.7% |

#### Most occurring blocks

| Value | Count | Frequency (%) |
| --- | --- | --- |
| ASCII | 25 | 100.0% |

#### Most frequent character per block

##### *ASCII*

| Value | Count | Frequency (%) |
| --- | --- | --- |
| 3 | 6 | 24.0% |
| 2 | 3 | 12.0% |
| \_ | 3 | 12.0% |
| 9 | 3 | 12.0% |
|  | 2 | 8.0% |
| M | 1 | 4.0% |
| a | 1 | 4.0% |
| i | 1 | 4.0% |
| s | 1 | 4.0% |
| d | 1 | 4.0% |
| Other values (3) | 3 | 12.0% |

HISTOPATOL  
Categorical

|  |  |
| --- | --- |
| Distinct | 1 |
| Distinct (%) | 25.0% |
| Missing | 0 |
| Missing (%) | 0.0% |
| Memory size | 64.0 B |

|  |  |
| --- | --- |
| N/realiz | 4 |

More details

- Overview
- Categories
- Words
- Characters

Length

|  |  |
| --- | --- |
| Max length | 8 |
| Median length | 8 |
| Mean length | 8 |
| Min length | 8 |

Characters and Unicode

|  |  |
| --- | --- |
| Total characters | 32 |
| Distinct characters | 8 |
| Distinct categories | 3 ? |
| Distinct scripts | 2 ? |
| Distinct blocks | 1 ? |

The Unicode Standard assigns character properties to each code point, which can be used to analyse textual variables.

Unique

|  |  |
| --- | --- |
| Unique | 0 ? |
| Unique (%) | 0.0% |

Sample

|  |  |
| --- | --- |
| 1st row | N/realiz |
| 2nd row | N/realiz |
| 3rd row | N/realiz |
| 4th row | N/realiz |

#### Common Values

| Value | Count | Frequency (%) |
| --- | --- | --- |
| N/realiz | 4 | 100.0% |

#### Length

xml version="1.0" encoding="utf-8" standalone="no"?2023-10-31T16:44:41.853847image/svg+xmlMatplotlib v3.6.0, https://matplotlib.org/ 

Histogram of lengths of the category

#### Common Values (Plot)

xml version="1.0" encoding="utf-8" standalone="no"?2023-10-31T16:44:41.986487image/svg+xmlMatplotlib v3.6.0, https://matplotlib.org/

| Value | Count | Frequency (%) |
| --- | --- | --- |
| n/realiz | 4 | 100.0% |

- Characters
- Categories
- Scripts
- Blocks

#### Most occurring characters

| Value | Count | Frequency (%) |
| --- | --- | --- |
| N | 4 | 12.5% |
| / | 4 | 12.5% |
| r | 4 | 12.5% |
| e | 4 | 12.5% |
| a | 4 | 12.5% |
| l | 4 | 12.5% |
| i | 4 | 12.5% |
| z | 4 | 12.5% |

#### Most occurring categories

| Value | Count | Frequency (%) |
| --- | --- | --- |
| Lowercase Letter | 24 | 75.0% |
| Uppercase Letter | 4 | 12.5% |
| Other Punctuation | 4 | 12.5% |

#### Most frequent character per category

##### *Lowercase Letter*

| Value | Count | Frequency (%) |
| --- | --- | --- |
| r | 4 | 16.7% |
| e | 4 | 16.7% |
| a | 4 | 16.7% |
| l | 4 | 16.7% |
| i | 4 | 16.7% |
| z | 4 | 16.7% |

##### *Uppercase Letter*

| Value | Count | Frequency (%) |
| --- | --- | --- |
| N | 4 | 100.0% |

##### *Other Punctuation*

| Value | Count | Frequency (%) |
| --- | --- | --- |
| / | 4 | 100.0% |

#### Most occurring scripts

| Value | Count | Frequency (%) |
| --- | --- | --- |
| Latin | 28 | 87.5% |
| Common | 4 | 12.5% |

#### Most frequent character per script

##### *Latin*

| Value | Count | Frequency (%) |
| --- | --- | --- |
| N | 4 | 14.3% |
| r | 4 | 14.3% |
| e | 4 | 14.3% |
| a | 4 | 14.3% |
| l | 4 | 14.3% |
| i | 4 | 14.3% |
| z | 4 | 14.3% |

##### *Common*

| Value | Count | Frequency (%) |
| --- | --- | --- |
| / | 4 | 100.0% |

#### Most occurring blocks

| Value | Count | Frequency (%) |
| --- | --- | --- |
| ASCII | 32 | 100.0% |

#### Most frequent character per block

##### *ASCII*

| Value | Count | Frequency (%) |
| --- | --- | --- |
| N | 4 | 12.5% |
| / | 4 | 12.5% |
| r | 4 | 12.5% |
| e | 4 | 12.5% |
| a | 4 | 12.5% |
| l | 4 | 12.5% |
| i | 4 | 12.5% |
| z | 4 | 12.5% |

Status\_Resistencia  
Categorical

|  |  |
| --- | --- |
| Distinct | 1 |
| Distinct (%) | 25.0% |
| Missing | 0 |
| Missing (%) | 0.0% |
| Memory size | 64.0 B |

|  |  |
| --- | --- |
| 1 | 4 |

More details

- Overview
- Categories
- Words
- Characters

Length

|  |  |
| --- | --- |
| Max length | 1 |
| Median length | 1 |
| Mean length | 1 |
| Min length | 1 |

Characters and Unicode

|  |  |
| --- | --- |
| Total characters | 4 |
| Distinct characters | 1 |
| Distinct categories | 1 ? |
| Distinct scripts | 1 ? |
| Distinct blocks | 1 ? |

The Unicode Standard assigns character properties to each code point, which can be used to analyse textual variables.

Unique

|  |  |
| --- | --- |
| Unique | 0 ? |
| Unique (%) | 0.0% |

Sample

|  |  |
| --- | --- |
| 1st row | 1 |
| 2nd row | 1 |
| 3rd row | 1 |
| 4th row | 1 |

#### Common Values

| Value | Count | Frequency (%) |
| --- | --- | --- |
| 1 | 4 | 100.0% |

#### Length

xml version="1.0" encoding="utf-8" standalone="no"?2023-10-31T16:44:42.100149image/svg+xmlMatplotlib v3.6.0, https://matplotlib.org/ 

Histogram of lengths of the category

#### Common Values (Plot)

xml version="1.0" encoding="utf-8" standalone="no"?2023-10-31T16:44:42.246159image/svg+xmlMatplotlib v3.6.0, https://matplotlib.org/

| Value | Count | Frequency (%) |
| --- | --- | --- |
| 1 | 4 | 100.0% |

- Characters
- Categories
- Scripts
- Blocks

#### Most occurring characters

| Value | Count | Frequency (%) |
| --- | --- | --- |
| 1 | 4 | 100.0% |

#### Most occurring categories

| Value | Count | Frequency (%) |
| --- | --- | --- |
| Decimal Number | 4 | 100.0% |

#### Most frequent character per category

##### *Decimal Number*

| Value | Count | Frequency (%) |
| --- | --- | --- |
| 1 | 4 | 100.0% |

#### Most occurring scripts

| Value | Count | Frequency (%) |
| --- | --- | --- |
| Common | 4 | 100.0% |

#### Most frequent character per script

##### *Common*

| Value | Count | Frequency (%) |
| --- | --- | --- |
| 1 | 4 | 100.0% |

#### Most occurring blocks

| Value | Count | Frequency (%) |
| --- | --- | --- |
| ASCII | 4 | 100.0% |

#### Most frequent character per block

##### *ASCII*

| Value | Count | Frequency (%) |
| --- | --- | --- |
| 1 | 4 | 100.0% |

Cluster  
Categorical

|  |  |
| --- | --- |
| Distinct | 1 |
| Distinct (%) | 25.0% |
| Missing | 0 |
| Missing (%) | 0.0% |
| Memory size | 64.0 B |

|  |  |
| --- | --- |
| 2 | 4 |

More details

- Overview
- Categories
- Words
- Characters

Length

|  |  |
| --- | --- |
| Max length | 1 |
| Median length | 1 |
| Mean length | 1 |
| Min length | 1 |

Characters and Unicode

|  |  |
| --- | --- |
| Total characters | 4 |
| Distinct characters | 1 |
| Distinct categories | 1 ? |
| Distinct scripts | 1 ? |
| Distinct blocks | 1 ? |

The Unicode Standard assigns character properties to each code point, which can be used to analyse textual variables.

Unique

|  |  |
| --- | --- |
| Unique | 0 ? |
| Unique (%) | 0.0% |

Sample

|  |  |
| --- | --- |
| 1st row | 2 |
| 2nd row | 2 |
| 3rd row | 2 |
| 4th row | 2 |

#### Common Values

| Value | Count | Frequency (%) |
| --- | --- | --- |
| 2 | 4 | 100.0% |

#### Length

xml version="1.0" encoding="utf-8" standalone="no"?2023-10-31T16:44:42.352175image/svg+xmlMatplotlib v3.6.0, https://matplotlib.org/ 

Histogram of lengths of the category

#### Common Values (Plot)

xml version="1.0" encoding="utf-8" standalone="no"?2023-10-31T16:44:42.487037image/svg+xmlMatplotlib v3.6.0, https://matplotlib.org/

| Value | Count | Frequency (%) |
| --- | --- | --- |
| 2 | 4 | 100.0% |

- Characters
- Categories
- Scripts
- Blocks

#### Most occurring characters

| Value | Count | Frequency (%) |
| --- | --- | --- |
| 2 | 4 | 100.0% |

#### Most occurring categories

| Value | Count | Frequency (%) |
| --- | --- | --- |
| Decimal Number | 4 | 100.0% |

#### Most frequent character per category

##### *Decimal Number*

| Value | Count | Frequency (%) |
| --- | --- | --- |
| 2 | 4 | 100.0% |

#### Most occurring scripts

| Value | Count | Frequency (%) |
| --- | --- | --- |
| Common | 4 | 100.0% |

#### Most frequent character per script

##### *Common*

| Value | Count | Frequency (%) |
| --- | --- | --- |
| 2 | 4 | 100.0% |

#### Most occurring blocks

| Value | Count | Frequency (%) |
| --- | --- | --- |
| ASCII | 4 | 100.0% |

#### Most frequent character per block

##### *ASCII*

| Value | Count | Frequency (%) |
| --- | --- | --- |
| 2 | 4 | 100.0% |

Probabilidade  
Categorical

`HIGH CORRELATION`  `UNIFORM`  `UNIQUE`

|  |  |
| --- | --- |
| Distinct | 4 |
| Distinct (%) | 100.0% |
| Missing | 0 |
| Missing (%) | 0.0% |
| Memory size | 64.0 B |

|  |  |
| --- | --- |
| 0.3571453191116193 | 1 |
| 0.3319027214101606 | 1 |
| 0.2761522297340586 | 1 |
| 0.27454173409707605 | 1 |

More details

- Overview
- Categories
- Words
- Characters

Length

|  |  |
| --- | --- |
| Max length | 19 |
| Median length | 18 |
| Mean length | 18.25 |
| Min length | 18 |

Characters and Unicode

|  |  |
| --- | --- |
| Total characters | 73 |
| Distinct characters | 11 |
| Distinct categories | 2 ? |
| Distinct scripts | 1 ? |
| Distinct blocks | 1 ? |

The Unicode Standard assigns character properties to each code point, which can be used to analyse textual variables.

Unique

|  |  |
| --- | --- |
| Unique | 4 ? |
| Unique (%) | 100.0% |

Sample

|  |  |
| --- | --- |
| 1st row | 0.3571453191116193 |
| 2nd row | 0.3319027214101606 |
| 3rd row | 0.2761522297340586 |
| 4th row | 0.27454173409707605 |

#### Common Values

| Value | Count | Frequency (%) |
| --- | --- | --- |
| 0.3571453191116193 | 1 | 25.0% |
| 0.3319027214101606 | 1 | 25.0% |
| 0.2761522297340586 | 1 | 25.0% |
| 0.27454173409707605 | 1 | 25.0% |

#### Length

xml version="1.0" encoding="utf-8" standalone="no"?2023-10-31T16:44:42.600326image/svg+xmlMatplotlib v3.6.0, https://matplotlib.org/ 

Histogram of lengths of the category

#### Common Values (Plot)

xml version="1.0" encoding="utf-8" standalone="no"?2023-10-31T16:44:42.760738image/svg+xmlMatplotlib v3.6.0, https://matplotlib.org/

| Value | Count | Frequency (%) |
| --- | --- | --- |
| 0.3571453191116193 | 1 | 25.0% |
| 0.3319027214101606 | 1 | 25.0% |
| 0.2761522297340586 | 1 | 25.0% |
| 0.27454173409707605 | 1 | 25.0% |

- Characters
- Categories
- Scripts
- Blocks

#### Most occurring characters

| Value | Count | Frequency (%) |
| --- | --- | --- |
| 1 | 12 | 16.4% |
| 0 | 11 | 15.1% |
| 7 | 8 | 11.0% |
| 3 | 7 | 9.6% |
| 2 | 7 | 9.6% |
| 5 | 6 | 8.2% |
| 4 | 6 | 8.2% |
| 6 | 6 | 8.2% |
| 9 | 5 | 6.8% |
| . | 4 | 5.5% |

#### Most occurring categories

| Value | Count | Frequency (%) |
| --- | --- | --- |
| Decimal Number | 69 | 94.5% |
| Other Punctuation | 4 | 5.5% |

#### Most frequent character per category

##### *Decimal Number*

| Value | Count | Frequency (%) |
| --- | --- | --- |
| 1 | 12 | 17.4% |
| 0 | 11 | 15.9% |
| 7 | 8 | 11.6% |
| 3 | 7 | 10.1% |
| 2 | 7 | 10.1% |
| 5 | 6 | 8.7% |
| 4 | 6 | 8.7% |
| 6 | 6 | 8.7% |
| 9 | 5 | 7.2% |
| 8 | 1 | 1.4% |

##### *Other Punctuation*

| Value | Count | Frequency (%) |
| --- | --- | --- |
| . | 4 | 100.0% |

#### Most occurring scripts

| Value | Count | Frequency (%) |
| --- | --- | --- |
| Common | 73 | 100.0% |

#### Most frequent character per script

##### *Common*

| Value | Count | Frequency (%) |
| --- | --- | --- |
| 1 | 12 | 16.4% |
| 0 | 11 | 15.1% |
| 7 | 8 | 11.0% |
| 3 | 7 | 9.6% |
| 2 | 7 | 9.6% |
| 5 | 6 | 8.2% |
| 4 | 6 | 8.2% |
| 6 | 6 | 8.2% |
| 9 | 5 | 6.8% |
| . | 4 | 5.5% |

#### Most occurring blocks

| Value | Count | Frequency (%) |
| --- | --- | --- |
| ASCII | 73 | 100.0% |

#### Most frequent character per block

##### *ASCII*

| Value | Count | Frequency (%) |
| --- | --- | --- |
| 1 | 12 | 16.4% |
| 0 | 11 | 15.1% |
| 7 | 8 | 11.0% |
| 3 | 7 | 9.6% |
| 2 | 7 | 9.6% |
| 5 | 6 | 8.2% |
| 4 | 6 | 8.2% |
| 6 | 6 | 8.2% |
| 9 | 5 | 6.8% |
| . | 4 | 5.5% |

# Correlations

- Auto

- Heatmap
- Table

xml version="1.0" encoding="utf-8" standalone="no"?2023-10-31T16:44:42.902609image/svg+xmlMatplotlib v3.6.0, https://matplotlib.org/

|  | faixaEtaria | ESCOLARID | sitAtual | FORMACLIN1 | classif | descoberta | bac | BACOUTRO | RX | hiv | ALCOOLISMO | DROGADICAO | TABAGISMO | idade | Probabilidade |
| --- | --- | --- | --- | --- | --- | --- | --- | --- | --- | --- | --- | --- | --- | --- | --- |
| faixaEtaria | 1.000 | 0.000 | 0.000 | 0.000 | 0.000 | 0.000 | 0.707 | 0.000 | 0.000 | 0.707 | 0.707 | 0.000 | 0.000 | 0.707 | 1.000 |
| ESCOLARID | 0.000 | 1.000 | 0.707 | 0.000 | 0.000 | 0.000 | 0.000 | 0.000 | 0.000 | 0.000 | 0.000 | 0.000 | 0.707 | 0.707 | 1.000 |
| sitAtual | 0.000 | 0.707 | 1.000 | 0.000 | 0.000 | 0.707 | 0.000 | 0.000 | 0.000 | 0.000 | 0.000 | 0.000 | 0.000 | 0.000 | 1.000 |
| FORMACLIN1 | 0.000 | 0.000 | 0.000 | 1.000 | 1.000 | 0.000 | 0.000 | 0.000 | 1.000 | 0.707 | 0.000 | 0.000 | 0.000 | 0.000 | 1.000 |
| classif | 0.000 | 0.000 | 0.000 | 1.000 | 1.000 | 0.000 | 0.000 | 0.000 | 1.000 | 0.707 | 0.000 | 0.000 | 0.000 | 0.000 | 1.000 |
| descoberta | 0.000 | 0.000 | 0.707 | 0.000 | 0.000 | 1.000 | 0.000 | 0.000 | 0.000 | 0.707 | 0.000 | 0.707 | 0.707 | 0.000 | 1.000 |
| bac | 0.707 | 0.000 | 0.000 | 0.000 | 0.000 | 0.000 | 1.000 | 0.000 | 0.000 | 0.000 | 0.000 | 0.000 | 0.000 | 0.000 | 1.000 |
| BACOUTRO | 0.000 | 0.000 | 0.000 | 0.000 | 0.000 | 0.000 | 0.000 | 1.000 | 0.000 | 0.000 | 0.000 | 0.707 | 0.000 | 0.707 | 1.000 |
| RX | 0.000 | 0.000 | 0.000 | 1.000 | 1.000 | 0.000 | 0.000 | 0.000 | 1.000 | 0.707 | 0.000 | 0.000 | 0.000 | 0.000 | 1.000 |
| hiv | 0.707 | 0.000 | 0.000 | 0.707 | 0.707 | 0.707 | 0.000 | 0.000 | 0.707 | 1.000 | 0.000 | 0.000 | 0.000 | 0.000 | 1.000 |
| ALCOOLISMO | 0.707 | 0.000 | 0.000 | 0.000 | 0.000 | 0.000 | 0.000 | 0.000 | 0.000 | 0.000 | 1.000 | 0.000 | 0.000 | 0.000 | 1.000 |
| DROGADICAO | 0.000 | 0.000 | 0.000 | 0.000 | 0.000 | 0.707 | 0.000 | 0.707 | 0.000 | 0.000 | 0.000 | 1.000 | 0.000 | 0.000 | 1.000 |
| TABAGISMO | 0.000 | 0.707 | 0.000 | 0.000 | 0.000 | 0.707 | 0.000 | 0.000 | 0.000 | 0.000 | 0.000 | 0.000 | 1.000 | 0.000 | 1.000 |
| idade | 0.707 | 0.707 | 0.000 | 0.000 | 0.000 | 0.000 | 0.000 | 0.707 | 0.000 | 0.000 | 0.000 | 0.000 | 0.000 | 1.000 | 1.000 |
| Probabilidade | 1.000 | 1.000 | 1.000 | 1.000 | 1.000 | 1.000 | 1.000 | 1.000 | 1.000 | 1.000 | 1.000 | 1.000 | 1.000 | 1.000 | 1.000 |

# Missing values

- Count
- Matrix

xml version="1.0" encoding="utf-8" standalone="no"?2023-10-31T16:44:34.581146image/svg+xmlMatplotlib v3.6.0, https://matplotlib.org/ 

A simple visualization of nullity by column.

xml version="1.0" encoding="utf-8" standalone="no"?2023-10-31T16:44:35.091970image/svg+xmlMatplotlib v3.6.0, https://matplotlib.org/ 

Nullity matrix is a data-dense display which lets you quickly visually pick out patterns in data completion.

# Sample

- First rows
- Last rows

|  | faixaEtaria | sexo | ESCOLARID | TIPOCUP | sitAtual | tipoCaso | FORMACLIN1 | classif | descoberta | bac | BACOUTRO | cultEsc | RX | NECROP | hiv | aids | DIABETES | ALCOOLISMO | MENTAL | DROGADICAO | TABAGISMO | motMudEsquema | tipoTrat | idade | HISTOPATOL | Status\_Resistencia | Cluster | Probabilidade |
| --- | --- | --- | --- | --- | --- | --- | --- | --- | --- | --- | --- | --- | --- | --- | --- | --- | --- | --- | --- | --- | --- | --- | --- | --- | --- | --- | --- | --- |
| 1490 | 60\_69 | M | Nenhuma | Outra | Cura | Novo | Pul | Pul | Elucidacao Diagn. em Internacao | N/realiz | Neg | N/realiz | Susp TB | N/realiz | Neg | N | N | N | N | N | N | Nulo | Supervisionado | Mais de 54 | N/realiz | 1 | 2 | 0.357145 |
| 794 | 30\_39 | M | De 4 a 7 anos | Outra | Abandono | Novo | Pul | Pul | Demanda Ambulatorial | Neg | N/realiz | N/realiz | Susp TB | N/realiz | Neg | N | N | S | N | S | S | Nulo | Supervisionado | 23\_39 | N/realiz | 1 | 2 | 0.331903 |
| 1416 | 20\_29 | M | De 8 a 11 anos | Outra | Cura | Novo | Multiplos Orgaos | Dissem | Urgencia / Emergencia | N/realiz | N/realiz | N/realiz | Normal | N/realiz | Pos | N | N | N | N | S | N | Nulo | Supervisionado | 23\_39 | N/realiz | 1 | 2 | 0.276152 |
| 1310 | 30\_39 | M | De 8 a 11 anos | Outra | Cura | Novo | Pleural | Ext | Elucidacao Diagn. em Internacao | Neg | Pos | N/realiz | N/realiz | N/realiz | Neg | N | N | S | N | N | N | Nulo | Supervisionado | 23\_39 | N/realiz | 1 | 2 | 0.274542 |

|  | faixaEtaria | sexo | ESCOLARID | TIPOCUP | sitAtual | tipoCaso | FORMACLIN1 | classif | descoberta | bac | BACOUTRO | cultEsc | RX | NECROP | hiv | aids | DIABETES | ALCOOLISMO | MENTAL | DROGADICAO | TABAGISMO | motMudEsquema | tipoTrat | idade | HISTOPATOL | Status\_Resistencia | Cluster | Probabilidade |
| --- | --- | --- | --- | --- | --- | --- | --- | --- | --- | --- | --- | --- | --- | --- | --- | --- | --- | --- | --- | --- | --- | --- | --- | --- | --- | --- | --- | --- |
| 1490 | 60\_69 | M | Nenhuma | Outra | Cura | Novo | Pul | Pul | Elucidacao Diagn. em Internacao | N/realiz | Neg | N/realiz | Susp TB | N/realiz | Neg | N | N | N | N | N | N | Nulo | Supervisionado | Mais de 54 | N/realiz | 1 | 2 | 0.357145 |
| 794 | 30\_39 | M | De 4 a 7 anos | Outra | Abandono | Novo | Pul | Pul | Demanda Ambulatorial | Neg | N/realiz | N/realiz | Susp TB | N/realiz | Neg | N | N | S | N | S | S | Nulo | Supervisionado | 23\_39 | N/realiz | 1 | 2 | 0.331903 |
| 1416 | 20\_29 | M | De 8 a 11 anos | Outra | Cura | Novo | Multiplos Orgaos | Dissem | Urgencia / Emergencia | N/realiz | N/realiz | N/realiz | Normal | N/realiz | Pos | N | N | N | N | S | N | Nulo | Supervisionado | 23\_39 | N/realiz | 1 | 2 | 0.276152 |
| 1310 | 30\_39 | M | De 8 a 11 anos | Outra | Cura | Novo | Pleural | Ext | Elucidacao Diagn. em Internacao | Neg | Pos | N/realiz | N/realiz | N/realiz | Neg | N | N | S | N | N | N | Nulo | Supervisionado | 23\_39 | N/realiz | 1 | 2 | 0.274542 |

Report generated by YData.

 
