# Supplementary material for: Model for predicting drug resistance based on the clinical profile of tuberculosis patients using machine learning techniques
Source: PeerJ Comput Sci. 2024 Oct 14;10:e2246. doi: 10.7717/peerj-cs.2246 (PMC11623081; doi:10.7717/peerj-cs.2246)
Supplement: Supplemental Information 2 [file peerj-cs-10-2246-s002.zip › code/EDA/false_positive_1.html]

Pandas Profiling Report 

Toggle navigationPandas Profiling Report

- Overview
- Variables
- Correlations
- Missing values
- Sample

# Overview

- Overview
- Alerts 33
- Reproduction

Dataset statistics

|  |  |
| --- | --- |
| Number of variables | 28 |
| Number of observations | 4 |
| Missing cells | 0 |
| Missing cells (%) | 0.0% |
| Duplicate rows | 0 |
| Duplicate rows (%) | 0.0% |
| Total size in memory | 1000.0 B |
| Average record size in memory | 250.0 B |

Variable types

|  |  |
| --- | --- |
| Categorical | 25 |
| Boolean | 3 |

Alerts

| `bac` has constant value "Neg" | Constant |
| `NECROP` has constant value "N/realiz" | Constant |
| `DIABETES` has constant value "False" | Constant |
| `ALCOOLISMO` has constant value "False" | Constant |
| `MENTAL` has constant value "False" | Constant |
| `motMudEsquema` has constant value "Nulo" | Constant |
| `HISTOPATOL` has constant value "N/realiz" | Constant |
| `Status_Resistencia` has constant value "0" | Constant |
| `Cluster` has constant value "0" | Constant |
| `faixaEtaria` is highly overall correlated with `FORMACLIN1` and 8 other fields | High correlation |
| `sexo` is highly overall correlated with `ESCOLARID` and 3 other fields | High correlation |
| `ESCOLARID` is highly overall correlated with `sexo` and 5 other fields | High correlation |
| `TIPOCUP` is highly overall correlated with `ESCOLARID` and 3 other fields | High correlation |
| `sitAtual` is highly overall correlated with `BACOUTRO` and 1 other fields | High correlation |
| `tipoCaso` is highly overall correlated with `ESCOLARID` and 3 other fields | High correlation |
| `FORMACLIN1` is highly overall correlated with `faixaEtaria` and 3 other fields | High correlation |
| `classif` is highly overall correlated with `tipoCaso` and 5 other fields | High correlation |
| `descoberta` is highly overall correlated with `sexo` and 5 other fields | High correlation |
| `BACOUTRO` is highly overall correlated with `sexo` and 8 other fields | High correlation |
| `cultEsc` is highly overall correlated with `faixaEtaria` and 3 other fields | High correlation |
| `RX` is highly overall correlated with `BACOUTRO` and 1 other fields | High correlation |
| `hiv` is highly overall correlated with `faixaEtaria` and 1 other fields | High correlation |
| `aids` is highly overall correlated with `faixaEtaria` and 1 other fields | High correlation |
| `DROGADICAO` is highly overall correlated with `faixaEtaria` and 3 other fields | High correlation |
| `TABAGISMO` is highly overall correlated with `faixaEtaria` and 3 other fields | High correlation |
| `tipoTrat` is highly overall correlated with `faixaEtaria` and 3 other fields | High correlation |
| `idade` is highly overall correlated with `faixaEtaria` and 1 other fields | High correlation |
| `Probabilidade` is highly overall correlated with `faixaEtaria` and 17 other fields | High correlation |
| `sitAtual` is uniformly distributed | Uniform |
| `RX` is uniformly distributed | Uniform |
| `tipoTrat` is uniformly distributed | Uniform |
| `Probabilidade` is uniformly distributed | Uniform |
| `Probabilidade` has unique values | Unique |

Reproduction

|  |  |
| --- | --- |
| Analysis started | 2023-10-31 19:42:27.127663 |
| Analysis finished | 2023-10-31 19:42:30.296644 |
| Duration | 3.17 seconds |
| Software version | pandas-profiling v3.6.6 |
| Download configuration | config.json |

# Variables

Select ColumnsfaixaEtariasexoESCOLARIDTIPOCUPsitAtualtipoCasoFORMACLIN1classifdescobertabacBACOUTROcultEscRXNECROPhivaidsDIABETESALCOOLISMOMENTALDROGADICAOTABAGISMOmotMudEsquematipoTratidadeHISTOPATOLStatus\_ResistenciaClusterProbabilidade

faixaEtaria  
Categorical

|  |  |
| --- | --- |
| Distinct | 3 |
| Distinct (%) | 75.0% |
| Missing | 0 |
| Missing (%) | 0.0% |
| Memory size | 64.0 B |

|  |  |
| --- | --- |
| 30\_39 | 2 |
| 40\_49 | 1 |
| 20\_29 | 1 |

More details

- Overview
- Categories
- Words
- Characters

Length

|  |  |
| --- | --- |
| Max length | 5 |
| Median length | 5 |
| Mean length | 5 |
| Min length | 5 |

Characters and Unicode

|  |  |
| --- | --- |
| Total characters | 20 |
| Distinct characters | 6 |
| Distinct categories | 2 ? |
| Distinct scripts | 1 ? |
| Distinct blocks | 1 ? |

The Unicode Standard assigns character properties to each code point, which can be used to analyse textual variables.

Unique

|  |  |
| --- | --- |
| Unique | 2 ? |
| Unique (%) | 50.0% |

Sample

|  |  |
| --- | --- |
| 1st row | 40\_49 |
| 2nd row | 30\_39 |
| 3rd row | 30\_39 |
| 4th row | 20\_29 |

#### Common Values

| Value | Count | Frequency (%) |
| --- | --- | --- |
| 30\_39 | 2 | 50.0% |
| 40\_49 | 1 | 25.0% |
| 20\_29 | 1 | 25.0% |

#### Length

xml version="1.0" encoding="utf-8" standalone="no"?2023-10-31T16:42:30.372026image/svg+xmlMatplotlib v3.6.0, https://matplotlib.org/ 

Histogram of lengths of the category

#### Common Values (Plot)

xml version="1.0" encoding="utf-8" standalone="no"?2023-10-31T16:42:30.523283image/svg+xmlMatplotlib v3.6.0, https://matplotlib.org/

| Value | Count | Frequency (%) |
| --- | --- | --- |
| 30\_39 | 2 | 50.0% |
| 40\_49 | 1 | 25.0% |
| 20\_29 | 1 | 25.0% |

- Characters
- Categories
- Scripts
- Blocks

#### Most occurring characters

| Value | Count | Frequency (%) |
| --- | --- | --- |
| 3 | 4 | 20.0% |
| 0 | 4 | 20.0% |
| \_ | 4 | 20.0% |
| 9 | 4 | 20.0% |
| 4 | 2 | 10.0% |
| 2 | 2 | 10.0% |

#### Most occurring categories

| Value | Count | Frequency (%) |
| --- | --- | --- |
| Decimal Number | 16 | 80.0% |
| Connector Punctuation | 4 | 20.0% |

#### Most frequent character per category

##### *Decimal Number*

| Value | Count | Frequency (%) |
| --- | --- | --- |
| 3 | 4 | 25.0% |
| 0 | 4 | 25.0% |
| 9 | 4 | 25.0% |
| 4 | 2 | 12.5% |
| 2 | 2 | 12.5% |

##### *Connector Punctuation*

| Value | Count | Frequency (%) |
| --- | --- | --- |
| \_ | 4 | 100.0% |

#### Most occurring scripts

| Value | Count | Frequency (%) |
| --- | --- | --- |
| Common | 20 | 100.0% |

#### Most frequent character per script

##### *Common*

| Value | Count | Frequency (%) |
| --- | --- | --- |
| 3 | 4 | 20.0% |
| 0 | 4 | 20.0% |
| \_ | 4 | 20.0% |
| 9 | 4 | 20.0% |
| 4 | 2 | 10.0% |
| 2 | 2 | 10.0% |

#### Most occurring blocks

| Value | Count | Frequency (%) |
| --- | --- | --- |
| ASCII | 20 | 100.0% |

#### Most frequent character per block

##### *ASCII*

| Value | Count | Frequency (%) |
| --- | --- | --- |
| 3 | 4 | 20.0% |
| 0 | 4 | 20.0% |
| \_ | 4 | 20.0% |
| 9 | 4 | 20.0% |
| 4 | 2 | 10.0% |
| 2 | 2 | 10.0% |

sexo  
Categorical

|  |  |
| --- | --- |
| Distinct | 2 |
| Distinct (%) | 50.0% |
| Missing | 0 |
| Missing (%) | 0.0% |
| Memory size | 160.0 B |

|  |  |
| --- | --- |
| M | 3 |
| F | 1 |

More details

- Overview
- Categories
- Words
- Characters

Length

|  |  |
| --- | --- |
| Max length | 1 |
| Median length | 1 |
| Mean length | 1 |
| Min length | 1 |

Characters and Unicode

|  |  |
| --- | --- |
| Total characters | 4 |
| Distinct characters | 2 |
| Distinct categories | 1 ? |
| Distinct scripts | 1 ? |
| Distinct blocks | 1 ? |

The Unicode Standard assigns character properties to each code point, which can be used to analyse textual variables.

Unique

|  |  |
| --- | --- |
| Unique | 1 ? |
| Unique (%) | 25.0% |

Sample

|  |  |
| --- | --- |
| 1st row | M |
| 2nd row | F |
| 3rd row | M |
| 4th row | M |

#### Common Values

| Value | Count | Frequency (%) |
| --- | --- | --- |
| M | 3 | 75.0% |
| F | 1 | 25.0% |

#### Length

xml version="1.0" encoding="utf-8" standalone="no"?2023-10-31T16:42:30.646971image/svg+xmlMatplotlib v3.6.0, https://matplotlib.org/ 

Histogram of lengths of the category

#### Common Values (Plot)

xml version="1.0" encoding="utf-8" standalone="no"?2023-10-31T16:42:30.789038image/svg+xmlMatplotlib v3.6.0, https://matplotlib.org/

| Value | Count | Frequency (%) |
| --- | --- | --- |
| m | 3 | 75.0% |
| f | 1 | 25.0% |

- Characters
- Categories
- Scripts
- Blocks

#### Most occurring characters

| Value | Count | Frequency (%) |
| --- | --- | --- |
| M | 3 | 75.0% |
| F | 1 | 25.0% |

#### Most occurring categories

| Value | Count | Frequency (%) |
| --- | --- | --- |
| Uppercase Letter | 4 | 100.0% |

#### Most frequent character per category

##### *Uppercase Letter*

| Value | Count | Frequency (%) |
| --- | --- | --- |
| M | 3 | 75.0% |
| F | 1 | 25.0% |

#### Most occurring scripts

| Value | Count | Frequency (%) |
| --- | --- | --- |
| Latin | 4 | 100.0% |

#### Most frequent character per script

##### *Latin*

| Value | Count | Frequency (%) |
| --- | --- | --- |
| M | 3 | 75.0% |
| F | 1 | 25.0% |

#### Most occurring blocks

| Value | Count | Frequency (%) |
| --- | --- | --- |
| ASCII | 4 | 100.0% |

#### Most frequent character per block

##### *ASCII*

| Value | Count | Frequency (%) |
| --- | --- | --- |
| M | 3 | 75.0% |
| F | 1 | 25.0% |

ESCOLARID  
Categorical

|  |  |
| --- | --- |
| Distinct | 3 |
| Distinct (%) | 75.0% |
| Missing | 0 |
| Missing (%) | 0.0% |
| Memory size | 64.0 B |

|  |  |
| --- | --- |
| De 8 a 11 anos | 2 |
| De 4 a 7 anos | 1 |
| De 12 a 14 anos | 1 |

More details

- Overview
- Categories
- Words
- Characters

Length

|  |  |
| --- | --- |
| Max length | 15 |
| Median length | 14.5 |
| Mean length | 14 |
| Min length | 13 |

Characters and Unicode

|  |  |
| --- | --- |
| Total characters | 56 |
| Distinct characters | 12 |
| Distinct categories | 4 ? |
| Distinct scripts | 2 ? |
| Distinct blocks | 1 ? |

The Unicode Standard assigns character properties to each code point, which can be used to analyse textual variables.

Unique

|  |  |
| --- | --- |
| Unique | 2 ? |
| Unique (%) | 50.0% |

Sample

|  |  |
| --- | --- |
| 1st row | De 8 a 11 anos |
| 2nd row | De 4 a 7 anos |
| 3rd row | De 12 a 14 anos |
| 4th row | De 8 a 11 anos |

#### Common Values

| Value | Count | Frequency (%) |
| --- | --- | --- |
| De 8 a 11 anos | 2 | 50.0% |
| De 4 a 7 anos | 1 | 25.0% |
| De 12 a 14 anos | 1 | 25.0% |

#### Length

xml version="1.0" encoding="utf-8" standalone="no"?2023-10-31T16:42:30.920337image/svg+xmlMatplotlib v3.6.0, https://matplotlib.org/ 

Histogram of lengths of the category

#### Common Values (Plot)

xml version="1.0" encoding="utf-8" standalone="no"?2023-10-31T16:42:31.088773image/svg+xmlMatplotlib v3.6.0, https://matplotlib.org/

| Value | Count | Frequency (%) |
| --- | --- | --- |
| de | 4 | 20.0% |
| a | 4 | 20.0% |
| anos | 4 | 20.0% |
| 8 | 2 | 10.0% |
| 11 | 2 | 10.0% |
| 4 | 1 | 5.0% |
| 7 | 1 | 5.0% |
| 12 | 1 | 5.0% |
| 14 | 1 | 5.0% |

- Characters
- Categories
- Scripts
- Blocks

#### Most occurring characters

| Value | Count | Frequency (%) |
| --- | --- | --- |
|  | 16 | 28.6% |
| a | 8 | 14.3% |
| 1 | 6 | 10.7% |
| D | 4 | 7.1% |
| e | 4 | 7.1% |
| n | 4 | 7.1% |
| o | 4 | 7.1% |
| s | 4 | 7.1% |
| 8 | 2 | 3.6% |
| 4 | 2 | 3.6% |
| Other values (2) | 2 | 3.6% |

#### Most occurring categories

| Value | Count | Frequency (%) |
| --- | --- | --- |
| Lowercase Letter | 24 | 42.9% |
| Space Separator | 16 | 28.6% |
| Decimal Number | 12 | 21.4% |
| Uppercase Letter | 4 | 7.1% |

#### Most frequent character per category

##### *Lowercase Letter*

| Value | Count | Frequency (%) |
| --- | --- | --- |
| a | 8 | 33.3% |
| e | 4 | 16.7% |
| n | 4 | 16.7% |
| o | 4 | 16.7% |
| s | 4 | 16.7% |

##### *Decimal Number*

| Value | Count | Frequency (%) |
| --- | --- | --- |
| 1 | 6 | 50.0% |
| 8 | 2 | 16.7% |
| 4 | 2 | 16.7% |
| 7 | 1 | 8.3% |
| 2 | 1 | 8.3% |

##### *Space Separator*

| Value | Count | Frequency (%) |
| --- | --- | --- |
|  | 16 | 100.0% |

##### *Uppercase Letter*

| Value | Count | Frequency (%) |
| --- | --- | --- |
| D | 4 | 100.0% |

#### Most occurring scripts

| Value | Count | Frequency (%) |
| --- | --- | --- |
| Common | 28 | 50.0% |
| Latin | 28 | 50.0% |

#### Most frequent character per script

##### *Common*

| Value | Count | Frequency (%) |
| --- | --- | --- |
|  | 16 | 57.1% |
| 1 | 6 | 21.4% |
| 8 | 2 | 7.1% |
| 4 | 2 | 7.1% |
| 7 | 1 | 3.6% |
| 2 | 1 | 3.6% |

##### *Latin*

| Value | Count | Frequency (%) |
| --- | --- | --- |
| a | 8 | 28.6% |
| D | 4 | 14.3% |
| e | 4 | 14.3% |
| n | 4 | 14.3% |
| o | 4 | 14.3% |
| s | 4 | 14.3% |

#### Most occurring blocks

| Value | Count | Frequency (%) |
| --- | --- | --- |
| ASCII | 56 | 100.0% |

#### Most frequent character per block

##### *ASCII*

| Value | Count | Frequency (%) |
| --- | --- | --- |
|  | 16 | 28.6% |
| a | 8 | 14.3% |
| 1 | 6 | 10.7% |
| D | 4 | 7.1% |
| e | 4 | 7.1% |
| n | 4 | 7.1% |
| o | 4 | 7.1% |
| s | 4 | 7.1% |
| 8 | 2 | 3.6% |
| 4 | 2 | 3.6% |
| Other values (2) | 2 | 3.6% |

TIPOCUP  
Categorical

|  |  |
| --- | --- |
| Distinct | 2 |
| Distinct (%) | 50.0% |
| Missing | 0 |
| Missing (%) | 0.0% |
| Memory size | 64.0 B |

|  |  |
| --- | --- |
| Outra | 3 |
| Dona de Casa | 1 |

More details

- Overview
- Categories
- Words
- Characters

Length

|  |  |
| --- | --- |
| Max length | 12 |
| Median length | 5 |
| Mean length | 6.75 |
| Min length | 5 |

Characters and Unicode

|  |  |
| --- | --- |
| Total characters | 27 |
| Distinct characters | 13 |
| Distinct categories | 3 ? |
| Distinct scripts | 2 ? |
| Distinct blocks | 1 ? |

The Unicode Standard assigns character properties to each code point, which can be used to analyse textual variables.

Unique

|  |  |
| --- | --- |
| Unique | 1 ? |
| Unique (%) | 25.0% |

Sample

|  |  |
| --- | --- |
| 1st row | Outra |
| 2nd row | Dona de Casa |
| 3rd row | Outra |
| 4th row | Outra |

#### Common Values

| Value | Count | Frequency (%) |
| --- | --- | --- |
| Outra | 3 | 75.0% |
| Dona de Casa | 1 | 25.0% |

#### Length

xml version="1.0" encoding="utf-8" standalone="no"?2023-10-31T16:42:31.229246image/svg+xmlMatplotlib v3.6.0, https://matplotlib.org/ 

Histogram of lengths of the category

#### Common Values (Plot)

xml version="1.0" encoding="utf-8" standalone="no"?2023-10-31T16:42:31.397339image/svg+xmlMatplotlib v3.6.0, https://matplotlib.org/

| Value | Count | Frequency (%) |
| --- | --- | --- |
| outra | 3 | 50.0% |
| dona | 1 | 16.7% |
| de | 1 | 16.7% |
| casa | 1 | 16.7% |

- Characters
- Categories
- Scripts
- Blocks

#### Most occurring characters

| Value | Count | Frequency (%) |
| --- | --- | --- |
| a | 6 | 22.2% |
| O | 3 | 11.1% |
| u | 3 | 11.1% |
| t | 3 | 11.1% |
| r | 3 | 11.1% |
|  | 2 | 7.4% |
| D | 1 | 3.7% |
| o | 1 | 3.7% |
| n | 1 | 3.7% |
| d | 1 | 3.7% |
| Other values (3) | 3 | 11.1% |

#### Most occurring categories

| Value | Count | Frequency (%) |
| --- | --- | --- |
| Lowercase Letter | 20 | 74.1% |
| Uppercase Letter | 5 | 18.5% |
| Space Separator | 2 | 7.4% |

#### Most frequent character per category

##### *Lowercase Letter*

| Value | Count | Frequency (%) |
| --- | --- | --- |
| a | 6 | 30.0% |
| u | 3 | 15.0% |
| t | 3 | 15.0% |
| r | 3 | 15.0% |
| o | 1 | 5.0% |
| n | 1 | 5.0% |
| d | 1 | 5.0% |
| e | 1 | 5.0% |
| s | 1 | 5.0% |

##### *Uppercase Letter*

| Value | Count | Frequency (%) |
| --- | --- | --- |
| O | 3 | 60.0% |
| D | 1 | 20.0% |
| C | 1 | 20.0% |

##### *Space Separator*

| Value | Count | Frequency (%) |
| --- | --- | --- |
|  | 2 | 100.0% |

#### Most occurring scripts

| Value | Count | Frequency (%) |
| --- | --- | --- |
| Latin | 25 | 92.6% |
| Common | 2 | 7.4% |

#### Most frequent character per script

##### *Latin*

| Value | Count | Frequency (%) |
| --- | --- | --- |
| a | 6 | 24.0% |
| O | 3 | 12.0% |
| u | 3 | 12.0% |
| t | 3 | 12.0% |
| r | 3 | 12.0% |
| D | 1 | 4.0% |
| o | 1 | 4.0% |
| n | 1 | 4.0% |
| d | 1 | 4.0% |
| e | 1 | 4.0% |
| Other values (2) | 2 | 8.0% |

##### *Common*

| Value | Count | Frequency (%) |
| --- | --- | --- |
|  | 2 | 100.0% |

#### Most occurring blocks

| Value | Count | Frequency (%) |
| --- | --- | --- |
| ASCII | 27 | 100.0% |

#### Most frequent character per block

##### *ASCII*

| Value | Count | Frequency (%) |
| --- | --- | --- |
| a | 6 | 22.2% |
| O | 3 | 11.1% |
| u | 3 | 11.1% |
| t | 3 | 11.1% |
| r | 3 | 11.1% |
|  | 2 | 7.4% |
| D | 1 | 3.7% |
| o | 1 | 3.7% |
| n | 1 | 3.7% |
| d | 1 | 3.7% |
| Other values (3) | 3 | 11.1% |

sitAtual  
Categorical

`HIGH CORRELATION`  `UNIFORM`

|  |  |
| --- | --- |
| Distinct | 2 |
| Distinct (%) | 50.0% |
| Missing | 0 |
| Missing (%) | 0.0% |
| Memory size | 64.0 B |

|  |  |
| --- | --- |
| Abandono | 2 |
| Cura | 2 |

More details

- Overview
- Categories
- Words
- Characters

Length

|  |  |
| --- | --- |
| Max length | 8 |
| Median length | 6 |
| Mean length | 6 |
| Min length | 4 |

Characters and Unicode

|  |  |
| --- | --- |
| Total characters | 24 |
| Distinct characters | 9 |
| Distinct categories | 2 ? |
| Distinct scripts | 1 ? |
| Distinct blocks | 1 ? |

The Unicode Standard assigns character properties to each code point, which can be used to analyse textual variables.

Unique

|  |  |
| --- | --- |
| Unique | 0 ? |
| Unique (%) | 0.0% |

Sample

|  |  |
| --- | --- |
| 1st row | Abandono |
| 2nd row | Abandono |
| 3rd row | Cura |
| 4th row | Cura |

#### Common Values

| Value | Count | Frequency (%) |
| --- | --- | --- |
| Abandono | 2 | 50.0% |
| Cura | 2 | 50.0% |

#### Length

xml version="1.0" encoding="utf-8" standalone="no"?2023-10-31T16:42:31.542806image/svg+xmlMatplotlib v3.6.0, https://matplotlib.org/ 

Histogram of lengths of the category

#### Common Values (Plot)

xml version="1.0" encoding="utf-8" standalone="no"?2023-10-31T16:42:31.702801image/svg+xmlMatplotlib v3.6.0, https://matplotlib.org/

| Value | Count | Frequency (%) |
| --- | --- | --- |
| abandono | 2 | 50.0% |
| cura | 2 | 50.0% |

- Characters
- Categories
- Scripts
- Blocks

#### Most occurring characters

| Value | Count | Frequency (%) |
| --- | --- | --- |
| a | 4 | 16.7% |
| n | 4 | 16.7% |
| o | 4 | 16.7% |
| A | 2 | 8.3% |
| b | 2 | 8.3% |
| d | 2 | 8.3% |
| C | 2 | 8.3% |
| u | 2 | 8.3% |
| r | 2 | 8.3% |

#### Most occurring categories

| Value | Count | Frequency (%) |
| --- | --- | --- |
| Lowercase Letter | 20 | 83.3% |
| Uppercase Letter | 4 | 16.7% |

#### Most frequent character per category

##### *Lowercase Letter*

| Value | Count | Frequency (%) |
| --- | --- | --- |
| a | 4 | 20.0% |
| n | 4 | 20.0% |
| o | 4 | 20.0% |
| b | 2 | 10.0% |
| d | 2 | 10.0% |
| u | 2 | 10.0% |
| r | 2 | 10.0% |

##### *Uppercase Letter*

| Value | Count | Frequency (%) |
| --- | --- | --- |
| A | 2 | 50.0% |
| C | 2 | 50.0% |

#### Most occurring scripts

| Value | Count | Frequency (%) |
| --- | --- | --- |
| Latin | 24 | 100.0% |

#### Most frequent character per script

##### *Latin*

| Value | Count | Frequency (%) |
| --- | --- | --- |
| a | 4 | 16.7% |
| n | 4 | 16.7% |
| o | 4 | 16.7% |
| A | 2 | 8.3% |
| b | 2 | 8.3% |
| d | 2 | 8.3% |
| C | 2 | 8.3% |
| u | 2 | 8.3% |
| r | 2 | 8.3% |

#### Most occurring blocks

| Value | Count | Frequency (%) |
| --- | --- | --- |
| ASCII | 24 | 100.0% |

#### Most frequent character per block

##### *ASCII*

| Value | Count | Frequency (%) |
| --- | --- | --- |
| a | 4 | 16.7% |
| n | 4 | 16.7% |
| o | 4 | 16.7% |
| A | 2 | 8.3% |
| b | 2 | 8.3% |
| d | 2 | 8.3% |
| C | 2 | 8.3% |
| u | 2 | 8.3% |
| r | 2 | 8.3% |

tipoCaso  
Categorical

|  |  |
| --- | --- |
| Distinct | 2 |
| Distinct (%) | 50.0% |
| Missing | 0 |
| Missing (%) | 0.0% |
| Memory size | 64.0 B |

|  |  |
| --- | --- |
| Novo | 3 |
| Recidiva | 1 |

More details

- Overview
- Categories
- Words
- Characters

Length

|  |  |
| --- | --- |
| Max length | 8 |
| Median length | 4 |
| Mean length | 5 |
| Min length | 4 |

Characters and Unicode

|  |  |
| --- | --- |
| Total characters | 20 |
| Distinct characters | 9 |
| Distinct categories | 2 ? |
| Distinct scripts | 1 ? |
| Distinct blocks | 1 ? |

The Unicode Standard assigns character properties to each code point, which can be used to analyse textual variables.

Unique

|  |  |
| --- | --- |
| Unique | 1 ? |
| Unique (%) | 25.0% |

Sample

|  |  |
| --- | --- |
| 1st row | Novo |
| 2nd row | Novo |
| 3rd row | Recidiva |
| 4th row | Novo |

#### Common Values

| Value | Count | Frequency (%) |
| --- | --- | --- |
| Novo | 3 | 75.0% |
| Recidiva | 1 | 25.0% |

#### Length

xml version="1.0" encoding="utf-8" standalone="no"?2023-10-31T16:42:31.833852image/svg+xmlMatplotlib v3.6.0, https://matplotlib.org/ 

Histogram of lengths of the category

#### Common Values (Plot)

xml version="1.0" encoding="utf-8" standalone="no"?2023-10-31T16:42:31.993625image/svg+xmlMatplotlib v3.6.0, https://matplotlib.org/

| Value | Count | Frequency (%) |
| --- | --- | --- |
| novo | 3 | 75.0% |
| recidiva | 1 | 25.0% |

- Characters
- Categories
- Scripts
- Blocks

#### Most occurring characters

| Value | Count | Frequency (%) |
| --- | --- | --- |
| o | 6 | 30.0% |
| v | 4 | 20.0% |
| N | 3 | 15.0% |
| i | 2 | 10.0% |
| R | 1 | 5.0% |
| e | 1 | 5.0% |
| c | 1 | 5.0% |
| d | 1 | 5.0% |
| a | 1 | 5.0% |

#### Most occurring categories

| Value | Count | Frequency (%) |
| --- | --- | --- |
| Lowercase Letter | 16 | 80.0% |
| Uppercase Letter | 4 | 20.0% |

#### Most frequent character per category

##### *Lowercase Letter*

| Value | Count | Frequency (%) |
| --- | --- | --- |
| o | 6 | 37.5% |
| v | 4 | 25.0% |
| i | 2 | 12.5% |
| e | 1 | 6.2% |
| c | 1 | 6.2% |
| d | 1 | 6.2% |
| a | 1 | 6.2% |

##### *Uppercase Letter*

| Value | Count | Frequency (%) |
| --- | --- | --- |
| N | 3 | 75.0% |
| R | 1 | 25.0% |

#### Most occurring scripts

| Value | Count | Frequency (%) |
| --- | --- | --- |
| Latin | 20 | 100.0% |

#### Most frequent character per script

##### *Latin*

| Value | Count | Frequency (%) |
| --- | --- | --- |
| o | 6 | 30.0% |
| v | 4 | 20.0% |
| N | 3 | 15.0% |
| i | 2 | 10.0% |
| R | 1 | 5.0% |
| e | 1 | 5.0% |
| c | 1 | 5.0% |
| d | 1 | 5.0% |
| a | 1 | 5.0% |

#### Most occurring blocks

| Value | Count | Frequency (%) |
| --- | --- | --- |
| ASCII | 20 | 100.0% |

#### Most frequent character per block

##### *ASCII*

| Value | Count | Frequency (%) |
| --- | --- | --- |
| o | 6 | 30.0% |
| v | 4 | 20.0% |
| N | 3 | 15.0% |
| i | 2 | 10.0% |
| R | 1 | 5.0% |
| e | 1 | 5.0% |
| c | 1 | 5.0% |
| d | 1 | 5.0% |
| a | 1 | 5.0% |

FORMACLIN1  
Categorical

|  |  |
| --- | --- |
| Distinct | 2 |
| Distinct (%) | 50.0% |
| Missing | 0 |
| Missing (%) | 0.0% |
| Memory size | 64.0 B |

|  |  |
| --- | --- |
| Pul | 3 |
| Ganglionar Periferica | 1 |

More details

- Overview
- Categories
- Words
- Characters

Length

|  |  |
| --- | --- |
| Max length | 21 |
| Median length | 3 |
| Mean length | 7.5 |
| Min length | 3 |

Characters and Unicode

|  |  |
| --- | --- |
| Total characters | 30 |
| Distinct characters | 14 |
| Distinct categories | 3 ? |
| Distinct scripts | 2 ? |
| Distinct blocks | 1 ? |

The Unicode Standard assigns character properties to each code point, which can be used to analyse textual variables.

Unique

|  |  |
| --- | --- |
| Unique | 1 ? |
| Unique (%) | 25.0% |

Sample

|  |  |
| --- | --- |
| 1st row | Ganglionar Periferica |
| 2nd row | Pul |
| 3rd row | Pul |
| 4th row | Pul |

#### Common Values

| Value | Count | Frequency (%) |
| --- | --- | --- |
| Pul | 3 | 75.0% |
| Ganglionar Periferica | 1 | 25.0% |

#### Length

xml version="1.0" encoding="utf-8" standalone="no"?2023-10-31T16:42:32.124856image/svg+xmlMatplotlib v3.6.0, https://matplotlib.org/ 

Histogram of lengths of the category

#### Common Values (Plot)

xml version="1.0" encoding="utf-8" standalone="no"?2023-10-31T16:42:32.294657image/svg+xmlMatplotlib v3.6.0, https://matplotlib.org/

| Value | Count | Frequency (%) |
| --- | --- | --- |
| pul | 3 | 60.0% |
| ganglionar | 1 | 20.0% |
| periferica | 1 | 20.0% |

- Characters
- Categories
- Scripts
- Blocks

#### Most occurring characters

| Value | Count | Frequency (%) |
| --- | --- | --- |
| P | 4 | 13.3% |
| l | 4 | 13.3% |
| u | 3 | 10.0% |
| a | 3 | 10.0% |
| i | 3 | 10.0% |
| r | 3 | 10.0% |
| n | 2 | 6.7% |
| e | 2 | 6.7% |
| G | 1 | 3.3% |
| g | 1 | 3.3% |
| Other values (4) | 4 | 13.3% |

#### Most occurring categories

| Value | Count | Frequency (%) |
| --- | --- | --- |
| Lowercase Letter | 24 | 80.0% |
| Uppercase Letter | 5 | 16.7% |
| Space Separator | 1 | 3.3% |

#### Most frequent character per category

##### *Lowercase Letter*

| Value | Count | Frequency (%) |
| --- | --- | --- |
| l | 4 | 16.7% |
| u | 3 | 12.5% |
| a | 3 | 12.5% |
| i | 3 | 12.5% |
| r | 3 | 12.5% |
| n | 2 | 8.3% |
| e | 2 | 8.3% |
| g | 1 | 4.2% |
| o | 1 | 4.2% |
| f | 1 | 4.2% |

##### *Uppercase Letter*

| Value | Count | Frequency (%) |
| --- | --- | --- |
| P | 4 | 80.0% |
| G | 1 | 20.0% |

##### *Space Separator*

| Value | Count | Frequency (%) |
| --- | --- | --- |
|  | 1 | 100.0% |

#### Most occurring scripts

| Value | Count | Frequency (%) |
| --- | --- | --- |
| Latin | 29 | 96.7% |
| Common | 1 | 3.3% |

#### Most frequent character per script

##### *Latin*

| Value | Count | Frequency (%) |
| --- | --- | --- |
| P | 4 | 13.8% |
| l | 4 | 13.8% |
| u | 3 | 10.3% |
| a | 3 | 10.3% |
| i | 3 | 10.3% |
| r | 3 | 10.3% |
| n | 2 | 6.9% |
| e | 2 | 6.9% |
| G | 1 | 3.4% |
| g | 1 | 3.4% |
| Other values (3) | 3 | 10.3% |

##### *Common*

| Value | Count | Frequency (%) |
| --- | --- | --- |
|  | 1 | 100.0% |

#### Most occurring blocks

| Value | Count | Frequency (%) |
| --- | --- | --- |
| ASCII | 30 | 100.0% |

#### Most frequent character per block

##### *ASCII*

| Value | Count | Frequency (%) |
| --- | --- | --- |
| P | 4 | 13.3% |
| l | 4 | 13.3% |
| u | 3 | 10.0% |
| a | 3 | 10.0% |
| i | 3 | 10.0% |
| r | 3 | 10.0% |
| n | 2 | 6.7% |
| e | 2 | 6.7% |
| G | 1 | 3.3% |
| g | 1 | 3.3% |
| Other values (4) | 4 | 13.3% |

classif  
Categorical

|  |  |
| --- | --- |
| Distinct | 3 |
| Distinct (%) | 75.0% |
| Missing | 0 |
| Missing (%) | 0.0% |
| Memory size | 64.0 B |

|  |  |
| --- | --- |
| Pul | 2 |
| Ext | 1 |
| P+E | 1 |

More details

- Overview
- Categories
- Words
- Characters

Length

|  |  |
| --- | --- |
| Max length | 3 |
| Median length | 3 |
| Mean length | 3 |
| Min length | 3 |

Characters and Unicode

|  |  |
| --- | --- |
| Total characters | 12 |
| Distinct characters | 7 |
| Distinct categories | 3 ? |
| Distinct scripts | 2 ? |
| Distinct blocks | 1 ? |

The Unicode Standard assigns character properties to each code point, which can be used to analyse textual variables.

Unique

|  |  |
| --- | --- |
| Unique | 2 ? |
| Unique (%) | 50.0% |

Sample

|  |  |
| --- | --- |
| 1st row | Ext |
| 2nd row | Pul |
| 3rd row | P+E |
| 4th row | Pul |

#### Common Values

| Value | Count | Frequency (%) |
| --- | --- | --- |
| Pul | 2 | 50.0% |
| Ext | 1 | 25.0% |
| P+E | 1 | 25.0% |

#### Length

xml version="1.0" encoding="utf-8" standalone="no"?2023-10-31T16:42:32.415141image/svg+xmlMatplotlib v3.6.0, https://matplotlib.org/ 

Histogram of lengths of the category

#### Common Values (Plot)

xml version="1.0" encoding="utf-8" standalone="no"?2023-10-31T16:42:32.562304image/svg+xmlMatplotlib v3.6.0, https://matplotlib.org/

| Value | Count | Frequency (%) |
| --- | --- | --- |
| pul | 2 | 50.0% |
| ext | 1 | 25.0% |
| p+e | 1 | 25.0% |

- Characters
- Categories
- Scripts
- Blocks

#### Most occurring characters

| Value | Count | Frequency (%) |
| --- | --- | --- |
| P | 3 | 25.0% |
| u | 2 | 16.7% |
| l | 2 | 16.7% |
| E | 2 | 16.7% |
| x | 1 | 8.3% |
| t | 1 | 8.3% |
| + | 1 | 8.3% |

#### Most occurring categories

| Value | Count | Frequency (%) |
| --- | --- | --- |
| Lowercase Letter | 6 | 50.0% |
| Uppercase Letter | 5 | 41.7% |
| Math Symbol | 1 | 8.3% |

#### Most frequent character per category

##### *Lowercase Letter*

| Value | Count | Frequency (%) |
| --- | --- | --- |
| u | 2 | 33.3% |
| l | 2 | 33.3% |
| x | 1 | 16.7% |
| t | 1 | 16.7% |

##### *Uppercase Letter*

| Value | Count | Frequency (%) |
| --- | --- | --- |
| P | 3 | 60.0% |
| E | 2 | 40.0% |

##### *Math Symbol*

| Value | Count | Frequency (%) |
| --- | --- | --- |
| + | 1 | 100.0% |

#### Most occurring scripts

| Value | Count | Frequency (%) |
| --- | --- | --- |
| Latin | 11 | 91.7% |
| Common | 1 | 8.3% |

#### Most frequent character per script

##### *Latin*

| Value | Count | Frequency (%) |
| --- | --- | --- |
| P | 3 | 27.3% |
| u | 2 | 18.2% |
| l | 2 | 18.2% |
| E | 2 | 18.2% |
| x | 1 | 9.1% |
| t | 1 | 9.1% |

##### *Common*

| Value | Count | Frequency (%) |
| --- | --- | --- |
| + | 1 | 100.0% |

#### Most occurring blocks

| Value | Count | Frequency (%) |
| --- | --- | --- |
| ASCII | 12 | 100.0% |

#### Most frequent character per block

##### *ASCII*

| Value | Count | Frequency (%) |
| --- | --- | --- |
| P | 3 | 25.0% |
| u | 2 | 16.7% |
| l | 2 | 16.7% |
| E | 2 | 16.7% |
| x | 1 | 8.3% |
| t | 1 | 8.3% |
| + | 1 | 8.3% |

descoberta  
Categorical

|  |  |
| --- | --- |
| Distinct | 3 |
| Distinct (%) | 75.0% |
| Missing | 0 |
| Missing (%) | 0.0% |
| Memory size | 64.0 B |

|  |  |
| --- | --- |
| Elucidacao Diagn. em Internacao | 2 |
| Investigacao de Contatos | 1 |
| Demanda Ambulatorial | 1 |

More details

- Overview
- Categories
- Words
- Characters

Length

|  |  |
| --- | --- |
| Max length | 31 |
| Median length | 27.5 |
| Mean length | 26.5 |
| Min length | 20 |

Characters and Unicode

|  |  |
| --- | --- |
| Total characters | 106 |
| Distinct characters | 23 |
| Distinct categories | 4 ? |
| Distinct scripts | 2 ? |
| Distinct blocks | 1 ? |

The Unicode Standard assigns character properties to each code point, which can be used to analyse textual variables.

Unique

|  |  |
| --- | --- |
| Unique | 2 ? |
| Unique (%) | 50.0% |

Sample

|  |  |
| --- | --- |
| 1st row | Elucidacao Diagn. em Internacao |
| 2nd row | Investigacao de Contatos |
| 3rd row | Demanda Ambulatorial |
| 4th row | Elucidacao Diagn. em Internacao |

#### Common Values

| Value | Count | Frequency (%) |
| --- | --- | --- |
| Elucidacao Diagn. em Internacao | 2 | 50.0% |
| Investigacao de Contatos | 1 | 25.0% |
| Demanda Ambulatorial | 1 | 25.0% |

#### Length

xml version="1.0" encoding="utf-8" standalone="no"?2023-10-31T16:42:32.694953image/svg+xmlMatplotlib v3.6.0, https://matplotlib.org/ 

Histogram of lengths of the category

#### Common Values (Plot)

xml version="1.0" encoding="utf-8" standalone="no"?2023-10-31T16:42:32.854390image/svg+xmlMatplotlib v3.6.0, https://matplotlib.org/

| Value | Count | Frequency (%) |
| --- | --- | --- |
| elucidacao | 2 | 15.4% |
| diagn | 2 | 15.4% |
| em | 2 | 15.4% |
| internacao | 2 | 15.4% |
| investigacao | 1 | 7.7% |
| de | 1 | 7.7% |
| contatos | 1 | 7.7% |
| demanda | 1 | 7.7% |
| ambulatorial | 1 | 7.7% |

- Characters
- Categories
- Scripts
- Blocks

#### Most occurring characters

| Value | Count | Frequency (%) |
| --- | --- | --- |
| a | 17 | 16.0% |
| n | 9 | 8.5% |
|  | 9 | 8.5% |
| o | 8 | 7.5% |
| c | 7 | 6.6% |
| e | 7 | 6.6% |
| i | 6 | 5.7% |
| t | 6 | 5.7% |
| d | 4 | 3.8% |
| l | 4 | 3.8% |
| Other values (13) | 29 | 27.4% |

#### Most occurring categories

| Value | Count | Frequency (%) |
| --- | --- | --- |
| Lowercase Letter | 85 | 80.2% |
| Uppercase Letter | 10 | 9.4% |
| Space Separator | 9 | 8.5% |
| Other Punctuation | 2 | 1.9% |

#### Most frequent character per category

##### *Lowercase Letter*

| Value | Count | Frequency (%) |
| --- | --- | --- |
| a | 17 | 20.0% |
| n | 9 | 10.6% |
| o | 8 | 9.4% |
| c | 7 | 8.2% |
| e | 7 | 8.2% |
| i | 6 | 7.1% |
| t | 6 | 7.1% |
| d | 4 | 4.7% |
| l | 4 | 4.7% |
| m | 4 | 4.7% |
| Other values (6) | 13 | 15.3% |

##### *Uppercase Letter*

| Value | Count | Frequency (%) |
| --- | --- | --- |
| D | 3 | 30.0% |
| I | 3 | 30.0% |
| E | 2 | 20.0% |
| C | 1 | 10.0% |
| A | 1 | 10.0% |

##### *Space Separator*

| Value | Count | Frequency (%) |
| --- | --- | --- |
|  | 9 | 100.0% |

##### *Other Punctuation*

| Value | Count | Frequency (%) |
| --- | --- | --- |
| . | 2 | 100.0% |

#### Most occurring scripts

| Value | Count | Frequency (%) |
| --- | --- | --- |
| Latin | 95 | 89.6% |
| Common | 11 | 10.4% |

#### Most frequent character per script

##### *Latin*

| Value | Count | Frequency (%) |
| --- | --- | --- |
| a | 17 | 17.9% |
| n | 9 | 9.5% |
| o | 8 | 8.4% |
| c | 7 | 7.4% |
| e | 7 | 7.4% |
| i | 6 | 6.3% |
| t | 6 | 6.3% |
| d | 4 | 4.2% |
| l | 4 | 4.2% |
| m | 4 | 4.2% |
| Other values (11) | 23 | 24.2% |

##### *Common*

| Value | Count | Frequency (%) |
| --- | --- | --- |
|  | 9 | 81.8% |
| . | 2 | 18.2% |

#### Most occurring blocks

| Value | Count | Frequency (%) |
| --- | --- | --- |
| ASCII | 106 | 100.0% |

#### Most frequent character per block

##### *ASCII*

| Value | Count | Frequency (%) |
| --- | --- | --- |
| a | 17 | 16.0% |
| n | 9 | 8.5% |
|  | 9 | 8.5% |
| o | 8 | 7.5% |
| c | 7 | 6.6% |
| e | 7 | 6.6% |
| i | 6 | 5.7% |
| t | 6 | 5.7% |
| d | 4 | 3.8% |
| l | 4 | 3.8% |
| Other values (13) | 29 | 27.4% |

bac  
Categorical

|  |  |
| --- | --- |
| Distinct | 1 |
| Distinct (%) | 25.0% |
| Missing | 0 |
| Missing (%) | 0.0% |
| Memory size | 64.0 B |

|  |  |
| --- | --- |
| Neg | 4 |

More details

- Overview
- Categories
- Words
- Characters

Length

|  |  |
| --- | --- |
| Max length | 3 |
| Median length | 3 |
| Mean length | 3 |
| Min length | 3 |

Characters and Unicode

|  |  |
| --- | --- |
| Total characters | 12 |
| Distinct characters | 3 |
| Distinct categories | 2 ? |
| Distinct scripts | 1 ? |
| Distinct blocks | 1 ? |

The Unicode Standard assigns character properties to each code point, which can be used to analyse textual variables.

Unique

|  |  |
| --- | --- |
| Unique | 0 ? |
| Unique (%) | 0.0% |

Sample

|  |  |
| --- | --- |
| 1st row | Neg |
| 2nd row | Neg |
| 3rd row | Neg |
| 4th row | Neg |

#### Common Values

| Value | Count | Frequency (%) |
| --- | --- | --- |
| Neg | 4 | 100.0% |

#### Length

xml version="1.0" encoding="utf-8" standalone="no"?2023-10-31T16:42:32.997370image/svg+xmlMatplotlib v3.6.0, https://matplotlib.org/ 

Histogram of lengths of the category

#### Common Values (Plot)

xml version="1.0" encoding="utf-8" standalone="no"?2023-10-31T16:42:33.138061image/svg+xmlMatplotlib v3.6.0, https://matplotlib.org/

| Value | Count | Frequency (%) |
| --- | --- | --- |
| neg | 4 | 100.0% |

- Characters
- Categories
- Scripts
- Blocks

#### Most occurring characters

| Value | Count | Frequency (%) |
| --- | --- | --- |
| N | 4 | 33.3% |
| e | 4 | 33.3% |
| g | 4 | 33.3% |

#### Most occurring categories

| Value | Count | Frequency (%) |
| --- | --- | --- |
| Lowercase Letter | 8 | 66.7% |
| Uppercase Letter | 4 | 33.3% |

#### Most frequent character per category

##### *Lowercase Letter*

| Value | Count | Frequency (%) |
| --- | --- | --- |
| e | 4 | 50.0% |
| g | 4 | 50.0% |

##### *Uppercase Letter*

| Value | Count | Frequency (%) |
| --- | --- | --- |
| N | 4 | 100.0% |

#### Most occurring scripts

| Value | Count | Frequency (%) |
| --- | --- | --- |
| Latin | 12 | 100.0% |

#### Most frequent character per script

##### *Latin*

| Value | Count | Frequency (%) |
| --- | --- | --- |
| N | 4 | 33.3% |
| e | 4 | 33.3% |
| g | 4 | 33.3% |

#### Most occurring blocks

| Value | Count | Frequency (%) |
| --- | --- | --- |
| ASCII | 12 | 100.0% |

#### Most frequent character per block

##### *ASCII*

| Value | Count | Frequency (%) |
| --- | --- | --- |
| N | 4 | 33.3% |
| e | 4 | 33.3% |
| g | 4 | 33.3% |

BACOUTRO  
Categorical

|  |  |
| --- | --- |
| Distinct | 3 |
| Distinct (%) | 75.0% |
| Missing | 0 |
| Missing (%) | 0.0% |
| Memory size | 64.0 B |

|  |  |
| --- | --- |
| Neg | 2 |
| Pos | 1 |
| N/realiz | 1 |

More details

- Overview
- Categories
- Words
- Characters

Length

|  |  |
| --- | --- |
| Max length | 8 |
| Median length | 3 |
| Mean length | 4.25 |
| Min length | 3 |

Characters and Unicode

|  |  |
| --- | --- |
| Total characters | 17 |
| Distinct characters | 12 |
| Distinct categories | 3 ? |
| Distinct scripts | 2 ? |
| Distinct blocks | 1 ? |

The Unicode Standard assigns character properties to each code point, which can be used to analyse textual variables.

Unique

|  |  |
| --- | --- |
| Unique | 2 ? |
| Unique (%) | 50.0% |

Sample

|  |  |
| --- | --- |
| 1st row | Pos |
| 2nd row | N/realiz |
| 3rd row | Neg |
| 4th row | Neg |

#### Common Values

| Value | Count | Frequency (%) |
| --- | --- | --- |
| Neg | 2 | 50.0% |
| Pos | 1 | 25.0% |
| N/realiz | 1 | 25.0% |

#### Length

xml version="1.0" encoding="utf-8" standalone="no"?2023-10-31T16:42:33.257395image/svg+xmlMatplotlib v3.6.0, https://matplotlib.org/ 

Histogram of lengths of the category

#### Common Values (Plot)

xml version="1.0" encoding="utf-8" standalone="no"?2023-10-31T16:42:33.416284image/svg+xmlMatplotlib v3.6.0, https://matplotlib.org/

| Value | Count | Frequency (%) |
| --- | --- | --- |
| neg | 2 | 50.0% |
| pos | 1 | 25.0% |
| n/realiz | 1 | 25.0% |

- Characters
- Categories
- Scripts
- Blocks

#### Most occurring characters

| Value | Count | Frequency (%) |
| --- | --- | --- |
| N | 3 | 17.6% |
| e | 3 | 17.6% |
| g | 2 | 11.8% |
| P | 1 | 5.9% |
| o | 1 | 5.9% |
| s | 1 | 5.9% |
| / | 1 | 5.9% |
| r | 1 | 5.9% |
| a | 1 | 5.9% |
| l | 1 | 5.9% |
| Other values (2) | 2 | 11.8% |

#### Most occurring categories

| Value | Count | Frequency (%) |
| --- | --- | --- |
| Lowercase Letter | 12 | 70.6% |
| Uppercase Letter | 4 | 23.5% |
| Other Punctuation | 1 | 5.9% |

#### Most frequent character per category

##### *Lowercase Letter*

| Value | Count | Frequency (%) |
| --- | --- | --- |
| e | 3 | 25.0% |
| g | 2 | 16.7% |
| o | 1 | 8.3% |
| s | 1 | 8.3% |
| r | 1 | 8.3% |
| a | 1 | 8.3% |
| l | 1 | 8.3% |
| i | 1 | 8.3% |
| z | 1 | 8.3% |

##### *Uppercase Letter*

| Value | Count | Frequency (%) |
| --- | --- | --- |
| N | 3 | 75.0% |
| P | 1 | 25.0% |

##### *Other Punctuation*

| Value | Count | Frequency (%) |
| --- | --- | --- |
| / | 1 | 100.0% |

#### Most occurring scripts

| Value | Count | Frequency (%) |
| --- | --- | --- |
| Latin | 16 | 94.1% |
| Common | 1 | 5.9% |

#### Most frequent character per script

##### *Latin*

| Value | Count | Frequency (%) |
| --- | --- | --- |
| N | 3 | 18.8% |
| e | 3 | 18.8% |
| g | 2 | 12.5% |
| P | 1 | 6.2% |
| o | 1 | 6.2% |
| s | 1 | 6.2% |
| r | 1 | 6.2% |
| a | 1 | 6.2% |
| l | 1 | 6.2% |
| i | 1 | 6.2% |

##### *Common*

| Value | Count | Frequency (%) |
| --- | --- | --- |
| / | 1 | 100.0% |

#### Most occurring blocks

| Value | Count | Frequency (%) |
| --- | --- | --- |
| ASCII | 17 | 100.0% |

#### Most frequent character per block

##### *ASCII*

| Value | Count | Frequency (%) |
| --- | --- | --- |
| N | 3 | 17.6% |
| e | 3 | 17.6% |
| g | 2 | 11.8% |
| P | 1 | 5.9% |
| o | 1 | 5.9% |
| s | 1 | 5.9% |
| / | 1 | 5.9% |
| r | 1 | 5.9% |
| a | 1 | 5.9% |
| l | 1 | 5.9% |
| Other values (2) | 2 | 11.8% |

cultEsc  
Categorical

|  |  |
| --- | --- |
| Distinct | 2 |
| Distinct (%) | 50.0% |
| Missing | 0 |
| Missing (%) | 0.0% |
| Memory size | 64.0 B |

|  |  |
| --- | --- |
| Pos | 3 |
| Neg | 1 |

More details

- Overview
- Categories
- Words
- Characters

Length

|  |  |
| --- | --- |
| Max length | 3 |
| Median length | 3 |
| Mean length | 3 |
| Min length | 3 |

Characters and Unicode

|  |  |
| --- | --- |
| Total characters | 12 |
| Distinct characters | 6 |
| Distinct categories | 2 ? |
| Distinct scripts | 1 ? |
| Distinct blocks | 1 ? |

The Unicode Standard assigns character properties to each code point, which can be used to analyse textual variables.

Unique

|  |  |
| --- | --- |
| Unique | 1 ? |
| Unique (%) | 25.0% |

Sample

|  |  |
| --- | --- |
| 1st row | Neg |
| 2nd row | Pos |
| 3rd row | Pos |
| 4th row | Pos |

#### Common Values

| Value | Count | Frequency (%) |
| --- | --- | --- |
| Pos | 3 | 75.0% |
| Neg | 1 | 25.0% |

#### Length

xml version="1.0" encoding="utf-8" standalone="no"?2023-10-31T16:42:33.546379image/svg+xmlMatplotlib v3.6.0, https://matplotlib.org/ 

Histogram of lengths of the category

#### Common Values (Plot)

xml version="1.0" encoding="utf-8" standalone="no"?2023-10-31T16:42:33.696142image/svg+xmlMatplotlib v3.6.0, https://matplotlib.org/

| Value | Count | Frequency (%) |
| --- | --- | --- |
| pos | 3 | 75.0% |
| neg | 1 | 25.0% |

- Characters
- Categories
- Scripts
- Blocks

#### Most occurring characters

| Value | Count | Frequency (%) |
| --- | --- | --- |
| P | 3 | 25.0% |
| o | 3 | 25.0% |
| s | 3 | 25.0% |
| N | 1 | 8.3% |
| e | 1 | 8.3% |
| g | 1 | 8.3% |

#### Most occurring categories

| Value | Count | Frequency (%) |
| --- | --- | --- |
| Lowercase Letter | 8 | 66.7% |
| Uppercase Letter | 4 | 33.3% |

#### Most frequent character per category

##### *Lowercase Letter*

| Value | Count | Frequency (%) |
| --- | --- | --- |
| o | 3 | 37.5% |
| s | 3 | 37.5% |
| e | 1 | 12.5% |
| g | 1 | 12.5% |

##### *Uppercase Letter*

| Value | Count | Frequency (%) |
| --- | --- | --- |
| P | 3 | 75.0% |
| N | 1 | 25.0% |

#### Most occurring scripts

| Value | Count | Frequency (%) |
| --- | --- | --- |
| Latin | 12 | 100.0% |

#### Most frequent character per script

##### *Latin*

| Value | Count | Frequency (%) |
| --- | --- | --- |
| P | 3 | 25.0% |
| o | 3 | 25.0% |
| s | 3 | 25.0% |
| N | 1 | 8.3% |
| e | 1 | 8.3% |
| g | 1 | 8.3% |

#### Most occurring blocks

| Value | Count | Frequency (%) |
| --- | --- | --- |
| ASCII | 12 | 100.0% |

#### Most frequent character per block

##### *ASCII*

| Value | Count | Frequency (%) |
| --- | --- | --- |
| P | 3 | 25.0% |
| o | 3 | 25.0% |
| s | 3 | 25.0% |
| N | 1 | 8.3% |
| e | 1 | 8.3% |
| g | 1 | 8.3% |

RX  
Categorical

`HIGH CORRELATION`  `UNIFORM`

|  |  |
| --- | --- |
| Distinct | 2 |
| Distinct (%) | 50.0% |
| Missing | 0 |
| Missing (%) | 0.0% |
| Memory size | 64.0 B |

|  |  |
| --- | --- |
| Normal | 2 |
| Susp TB | 2 |

More details

- Overview
- Categories
- Words
- Characters

Length

|  |  |
| --- | --- |
| Max length | 7 |
| Median length | 6.5 |
| Mean length | 6.5 |
| Min length | 6 |

Characters and Unicode

|  |  |
| --- | --- |
| Total characters | 26 |
| Distinct characters | 13 |
| Distinct categories | 3 ? |
| Distinct scripts | 2 ? |
| Distinct blocks | 1 ? |

The Unicode Standard assigns character properties to each code point, which can be used to analyse textual variables.

Unique

|  |  |
| --- | --- |
| Unique | 0 ? |
| Unique (%) | 0.0% |

Sample

|  |  |
| --- | --- |
| 1st row | Normal |
| 2nd row | Normal |
| 3rd row | Susp TB |
| 4th row | Susp TB |

#### Common Values

| Value | Count | Frequency (%) |
| --- | --- | --- |
| Normal | 2 | 50.0% |
| Susp TB | 2 | 50.0% |

#### Length

xml version="1.0" encoding="utf-8" standalone="no"?2023-10-31T16:42:33.820443image/svg+xmlMatplotlib v3.6.0, https://matplotlib.org/ 

Histogram of lengths of the category

#### Common Values (Plot)

xml version="1.0" encoding="utf-8" standalone="no"?2023-10-31T16:42:33.971776image/svg+xmlMatplotlib v3.6.0, https://matplotlib.org/

| Value | Count | Frequency (%) |
| --- | --- | --- |
| normal | 2 | 33.3% |
| susp | 2 | 33.3% |
| tb | 2 | 33.3% |

- Characters
- Categories
- Scripts
- Blocks

#### Most occurring characters

| Value | Count | Frequency (%) |
| --- | --- | --- |
| N | 2 | 7.7% |
| o | 2 | 7.7% |
| r | 2 | 7.7% |
| m | 2 | 7.7% |
| a | 2 | 7.7% |
| l | 2 | 7.7% |
| S | 2 | 7.7% |
| u | 2 | 7.7% |
| s | 2 | 7.7% |
| p | 2 | 7.7% |
| Other values (3) | 6 | 23.1% |

#### Most occurring categories

| Value | Count | Frequency (%) |
| --- | --- | --- |
| Lowercase Letter | 16 | 61.5% |
| Uppercase Letter | 8 | 30.8% |
| Space Separator | 2 | 7.7% |

#### Most frequent character per category

##### *Lowercase Letter*

| Value | Count | Frequency (%) |
| --- | --- | --- |
| o | 2 | 12.5% |
| r | 2 | 12.5% |
| m | 2 | 12.5% |
| a | 2 | 12.5% |
| l | 2 | 12.5% |
| u | 2 | 12.5% |
| s | 2 | 12.5% |
| p | 2 | 12.5% |

##### *Uppercase Letter*

| Value | Count | Frequency (%) |
| --- | --- | --- |
| N | 2 | 25.0% |
| S | 2 | 25.0% |
| T | 2 | 25.0% |
| B | 2 | 25.0% |

##### *Space Separator*

| Value | Count | Frequency (%) |
| --- | --- | --- |
|  | 2 | 100.0% |

#### Most occurring scripts

| Value | Count | Frequency (%) |
| --- | --- | --- |
| Latin | 24 | 92.3% |
| Common | 2 | 7.7% |

#### Most frequent character per script

##### *Latin*

| Value | Count | Frequency (%) |
| --- | --- | --- |
| N | 2 | 8.3% |
| o | 2 | 8.3% |
| r | 2 | 8.3% |
| m | 2 | 8.3% |
| a | 2 | 8.3% |
| l | 2 | 8.3% |
| S | 2 | 8.3% |
| u | 2 | 8.3% |
| s | 2 | 8.3% |
| p | 2 | 8.3% |
| Other values (2) | 4 | 16.7% |

##### *Common*

| Value | Count | Frequency (%) |
| --- | --- | --- |
|  | 2 | 100.0% |

#### Most occurring blocks

| Value | Count | Frequency (%) |
| --- | --- | --- |
| ASCII | 26 | 100.0% |

#### Most frequent character per block

##### *ASCII*

| Value | Count | Frequency (%) |
| --- | --- | --- |
| N | 2 | 7.7% |
| o | 2 | 7.7% |
| r | 2 | 7.7% |
| m | 2 | 7.7% |
| a | 2 | 7.7% |
| l | 2 | 7.7% |
| S | 2 | 7.7% |
| u | 2 | 7.7% |
| s | 2 | 7.7% |
| p | 2 | 7.7% |
| Other values (3) | 6 | 23.1% |

NECROP  
Categorical

|  |  |
| --- | --- |
| Distinct | 1 |
| Distinct (%) | 25.0% |
| Missing | 0 |
| Missing (%) | 0.0% |
| Memory size | 64.0 B |

|  |  |
| --- | --- |
| N/realiz | 4 |

More details

- Overview
- Categories
- Words
- Characters

Length

|  |  |
| --- | --- |
| Max length | 8 |
| Median length | 8 |
| Mean length | 8 |
| Min length | 8 |

Characters and Unicode

|  |  |
| --- | --- |
| Total characters | 32 |
| Distinct characters | 8 |
| Distinct categories | 3 ? |
| Distinct scripts | 2 ? |
| Distinct blocks | 1 ? |

The Unicode Standard assigns character properties to each code point, which can be used to analyse textual variables.

Unique

|  |  |
| --- | --- |
| Unique | 0 ? |
| Unique (%) | 0.0% |

Sample

|  |  |
| --- | --- |
| 1st row | N/realiz |
| 2nd row | N/realiz |
| 3rd row | N/realiz |
| 4th row | N/realiz |

#### Common Values

| Value | Count | Frequency (%) |
| --- | --- | --- |
| N/realiz | 4 | 100.0% |

#### Length

xml version="1.0" encoding="utf-8" standalone="no"?2023-10-31T16:42:34.092538image/svg+xmlMatplotlib v3.6.0, https://matplotlib.org/ 

Histogram of lengths of the category

#### Common Values (Plot)

xml version="1.0" encoding="utf-8" standalone="no"?2023-10-31T16:42:34.243486image/svg+xmlMatplotlib v3.6.0, https://matplotlib.org/

| Value | Count | Frequency (%) |
| --- | --- | --- |
| n/realiz | 4 | 100.0% |

- Characters
- Categories
- Scripts
- Blocks

#### Most occurring characters

| Value | Count | Frequency (%) |
| --- | --- | --- |
| N | 4 | 12.5% |
| / | 4 | 12.5% |
| r | 4 | 12.5% |
| e | 4 | 12.5% |
| a | 4 | 12.5% |
| l | 4 | 12.5% |
| i | 4 | 12.5% |
| z | 4 | 12.5% |

#### Most occurring categories

| Value | Count | Frequency (%) |
| --- | --- | --- |
| Lowercase Letter | 24 | 75.0% |
| Uppercase Letter | 4 | 12.5% |
| Other Punctuation | 4 | 12.5% |

#### Most frequent character per category

##### *Lowercase Letter*

| Value | Count | Frequency (%) |
| --- | --- | --- |
| r | 4 | 16.7% |
| e | 4 | 16.7% |
| a | 4 | 16.7% |
| l | 4 | 16.7% |
| i | 4 | 16.7% |
| z | 4 | 16.7% |

##### *Uppercase Letter*

| Value | Count | Frequency (%) |
| --- | --- | --- |
| N | 4 | 100.0% |

##### *Other Punctuation*

| Value | Count | Frequency (%) |
| --- | --- | --- |
| / | 4 | 100.0% |

#### Most occurring scripts

| Value | Count | Frequency (%) |
| --- | --- | --- |
| Latin | 28 | 87.5% |
| Common | 4 | 12.5% |

#### Most frequent character per script

##### *Latin*

| Value | Count | Frequency (%) |
| --- | --- | --- |
| N | 4 | 14.3% |
| r | 4 | 14.3% |
| e | 4 | 14.3% |
| a | 4 | 14.3% |
| l | 4 | 14.3% |
| i | 4 | 14.3% |
| z | 4 | 14.3% |

##### *Common*

| Value | Count | Frequency (%) |
| --- | --- | --- |
| / | 4 | 100.0% |

#### Most occurring blocks

| Value | Count | Frequency (%) |
| --- | --- | --- |
| ASCII | 32 | 100.0% |

#### Most frequent character per block

##### *ASCII*

| Value | Count | Frequency (%) |
| --- | --- | --- |
| N | 4 | 12.5% |
| / | 4 | 12.5% |
| r | 4 | 12.5% |
| e | 4 | 12.5% |
| a | 4 | 12.5% |
| l | 4 | 12.5% |
| i | 4 | 12.5% |
| z | 4 | 12.5% |

hiv  
Categorical

|  |  |
| --- | --- |
| Distinct | 2 |
| Distinct (%) | 50.0% |
| Missing | 0 |
| Missing (%) | 0.0% |
| Memory size | 64.0 B |

|  |  |
| --- | --- |
| Pos | 3 |
| Neg | 1 |

More details

- Overview
- Categories
- Words
- Characters

Length

|  |  |
| --- | --- |
| Max length | 3 |
| Median length | 3 |
| Mean length | 3 |
| Min length | 3 |

Characters and Unicode

|  |  |
| --- | --- |
| Total characters | 12 |
| Distinct characters | 6 |
| Distinct categories | 2 ? |
| Distinct scripts | 1 ? |
| Distinct blocks | 1 ? |

The Unicode Standard assigns character properties to each code point, which can be used to analyse textual variables.

Unique

|  |  |
| --- | --- |
| Unique | 1 ? |
| Unique (%) | 25.0% |

Sample

|  |  |
| --- | --- |
| 1st row | Pos |
| 2nd row | Pos |
| 3rd row | Pos |
| 4th row | Neg |

#### Common Values

| Value | Count | Frequency (%) |
| --- | --- | --- |
| Pos | 3 | 75.0% |
| Neg | 1 | 25.0% |

#### Length

xml version="1.0" encoding="utf-8" standalone="no"?2023-10-31T16:42:34.353326image/svg+xmlMatplotlib v3.6.0, https://matplotlib.org/ 

Histogram of lengths of the category

#### Common Values (Plot)

xml version="1.0" encoding="utf-8" standalone="no"?2023-10-31T16:42:34.494861image/svg+xmlMatplotlib v3.6.0, https://matplotlib.org/

| Value | Count | Frequency (%) |
| --- | --- | --- |
| pos | 3 | 75.0% |
| neg | 1 | 25.0% |

- Characters
- Categories
- Scripts
- Blocks

#### Most occurring characters

| Value | Count | Frequency (%) |
| --- | --- | --- |
| P | 3 | 25.0% |
| o | 3 | 25.0% |
| s | 3 | 25.0% |
| N | 1 | 8.3% |
| e | 1 | 8.3% |
| g | 1 | 8.3% |

#### Most occurring categories

| Value | Count | Frequency (%) |
| --- | --- | --- |
| Lowercase Letter | 8 | 66.7% |
| Uppercase Letter | 4 | 33.3% |

#### Most frequent character per category

##### *Lowercase Letter*

| Value | Count | Frequency (%) |
| --- | --- | --- |
| o | 3 | 37.5% |
| s | 3 | 37.5% |
| e | 1 | 12.5% |
| g | 1 | 12.5% |

##### *Uppercase Letter*

| Value | Count | Frequency (%) |
| --- | --- | --- |
| P | 3 | 75.0% |
| N | 1 | 25.0% |

#### Most occurring scripts

| Value | Count | Frequency (%) |
| --- | --- | --- |
| Latin | 12 | 100.0% |

#### Most frequent character per script

##### *Latin*

| Value | Count | Frequency (%) |
| --- | --- | --- |
| P | 3 | 25.0% |
| o | 3 | 25.0% |
| s | 3 | 25.0% |
| N | 1 | 8.3% |
| e | 1 | 8.3% |
| g | 1 | 8.3% |

#### Most occurring blocks

| Value | Count | Frequency (%) |
| --- | --- | --- |
| ASCII | 12 | 100.0% |

#### Most frequent character per block

##### *ASCII*

| Value | Count | Frequency (%) |
| --- | --- | --- |
| P | 3 | 25.0% |
| o | 3 | 25.0% |
| s | 3 | 25.0% |
| N | 1 | 8.3% |
| e | 1 | 8.3% |
| g | 1 | 8.3% |

aids  
Categorical

|  |  |
| --- | --- |
| Distinct | 2 |
| Distinct (%) | 50.0% |
| Missing | 0 |
| Missing (%) | 0.0% |
| Memory size | 64.0 B |

|  |  |
| --- | --- |
| S | 3 |
| N | 1 |

More details

- Overview
- Categories
- Words
- Characters

Length

|  |  |
| --- | --- |
| Max length | 1 |
| Median length | 1 |
| Mean length | 1 |
| Min length | 1 |

Characters and Unicode

|  |  |
| --- | --- |
| Total characters | 4 |
| Distinct characters | 2 |
| Distinct categories | 1 ? |
| Distinct scripts | 1 ? |
| Distinct blocks | 1 ? |

The Unicode Standard assigns character properties to each code point, which can be used to analyse textual variables.

Unique

|  |  |
| --- | --- |
| Unique | 1 ? |
| Unique (%) | 25.0% |

Sample

|  |  |
| --- | --- |
| 1st row | S |
| 2nd row | S |
| 3rd row | S |
| 4th row | N |

#### Common Values

| Value | Count | Frequency (%) |
| --- | --- | --- |
| S | 3 | 75.0% |
| N | 1 | 25.0% |

#### Length

xml version="1.0" encoding="utf-8" standalone="no"?2023-10-31T16:42:34.613436image/svg+xmlMatplotlib v3.6.0, https://matplotlib.org/ 

Histogram of lengths of the category

#### Common Values (Plot)

xml version="1.0" encoding="utf-8" standalone="no"?2023-10-31T16:42:34.762550image/svg+xmlMatplotlib v3.6.0, https://matplotlib.org/

| Value | Count | Frequency (%) |
| --- | --- | --- |
| s | 3 | 75.0% |
| n | 1 | 25.0% |

- Characters
- Categories
- Scripts
- Blocks

#### Most occurring characters

| Value | Count | Frequency (%) |
| --- | --- | --- |
| S | 3 | 75.0% |
| N | 1 | 25.0% |

#### Most occurring categories

| Value | Count | Frequency (%) |
| --- | --- | --- |
| Uppercase Letter | 4 | 100.0% |

#### Most frequent character per category

##### *Uppercase Letter*

| Value | Count | Frequency (%) |
| --- | --- | --- |
| S | 3 | 75.0% |
| N | 1 | 25.0% |

#### Most occurring scripts

| Value | Count | Frequency (%) |
| --- | --- | --- |
| Latin | 4 | 100.0% |

#### Most frequent character per script

##### *Latin*

| Value | Count | Frequency (%) |
| --- | --- | --- |
| S | 3 | 75.0% |
| N | 1 | 25.0% |

#### Most occurring blocks

| Value | Count | Frequency (%) |
| --- | --- | --- |
| ASCII | 4 | 100.0% |

#### Most frequent character per block

##### *ASCII*

| Value | Count | Frequency (%) |
| --- | --- | --- |
| S | 3 | 75.0% |
| N | 1 | 25.0% |

DIABETES  
Boolean

|  |  |
| --- | --- |
| Distinct | 1 |
| Distinct (%) | 25.0% |
| Missing | 0 |
| Missing (%) | 0.0% |
| Memory size | 36.0 B |

|  |  |
| --- | --- |
| False | 4 |

More details

- Common Values (Table)
- Common Values (Plot)

| Value | Count | Frequency (%) |
| --- | --- | --- |
| False | 4 | 100.0% |

xml version="1.0" encoding="utf-8" standalone="no"?2023-10-31T16:42:34.896108image/svg+xmlMatplotlib v3.6.0, https://matplotlib.org/

ALCOOLISMO  
Boolean

|  |  |
| --- | --- |
| Distinct | 1 |
| Distinct (%) | 25.0% |
| Missing | 0 |
| Missing (%) | 0.0% |
| Memory size | 36.0 B |

|  |  |
| --- | --- |
| False | 4 |

More details

- Common Values (Table)
- Common Values (Plot)

| Value | Count | Frequency (%) |
| --- | --- | --- |
| False | 4 | 100.0% |

xml version="1.0" encoding="utf-8" standalone="no"?2023-10-31T16:42:35.021482image/svg+xmlMatplotlib v3.6.0, https://matplotlib.org/

MENTAL  
Boolean

|  |  |
| --- | --- |
| Distinct | 1 |
| Distinct (%) | 25.0% |
| Missing | 0 |
| Missing (%) | 0.0% |
| Memory size | 36.0 B |

|  |  |
| --- | --- |
| False | 4 |

More details

- Common Values (Table)
- Common Values (Plot)

| Value | Count | Frequency (%) |
| --- | --- | --- |
| False | 4 | 100.0% |

xml version="1.0" encoding="utf-8" standalone="no"?2023-10-31T16:42:35.149768image/svg+xmlMatplotlib v3.6.0, https://matplotlib.org/

DROGADICAO  
Categorical

|  |  |
| --- | --- |
| Distinct | 2 |
| Distinct (%) | 50.0% |
| Missing | 0 |
| Missing (%) | 0.0% |
| Memory size | 64.0 B |

|  |  |
| --- | --- |
| N | 3 |
| S | 1 |

More details

- Overview
- Categories
- Words
- Characters

Length

|  |  |
| --- | --- |
| Max length | 1 |
| Median length | 1 |
| Mean length | 1 |
| Min length | 1 |

Characters and Unicode

|  |  |
| --- | --- |
| Total characters | 4 |
| Distinct characters | 2 |
| Distinct categories | 1 ? |
| Distinct scripts | 1 ? |
| Distinct blocks | 1 ? |

The Unicode Standard assigns character properties to each code point, which can be used to analyse textual variables.

Unique

|  |  |
| --- | --- |
| Unique | 1 ? |
| Unique (%) | 25.0% |

Sample

|  |  |
| --- | --- |
| 1st row | S |
| 2nd row | N |
| 3rd row | N |
| 4th row | N |

#### Common Values

| Value | Count | Frequency (%) |
| --- | --- | --- |
| N | 3 | 75.0% |
| S | 1 | 25.0% |

#### Length

xml version="1.0" encoding="utf-8" standalone="no"?2023-10-31T16:42:35.273251image/svg+xmlMatplotlib v3.6.0, https://matplotlib.org/ 

Histogram of lengths of the category

#### Common Values (Plot)

xml version="1.0" encoding="utf-8" standalone="no"?2023-10-31T16:42:35.465683image/svg+xmlMatplotlib v3.6.0, https://matplotlib.org/

| Value | Count | Frequency (%) |
| --- | --- | --- |
| n | 3 | 75.0% |
| s | 1 | 25.0% |

- Characters
- Categories
- Scripts
- Blocks

#### Most occurring characters

| Value | Count | Frequency (%) |
| --- | --- | --- |
| N | 3 | 75.0% |
| S | 1 | 25.0% |

#### Most occurring categories

| Value | Count | Frequency (%) |
| --- | --- | --- |
| Uppercase Letter | 4 | 100.0% |

#### Most frequent character per category

##### *Uppercase Letter*

| Value | Count | Frequency (%) |
| --- | --- | --- |
| N | 3 | 75.0% |
| S | 1 | 25.0% |

#### Most occurring scripts

| Value | Count | Frequency (%) |
| --- | --- | --- |
| Latin | 4 | 100.0% |

#### Most frequent character per script

##### *Latin*

| Value | Count | Frequency (%) |
| --- | --- | --- |
| N | 3 | 75.0% |
| S | 1 | 25.0% |

#### Most occurring blocks

| Value | Count | Frequency (%) |
| --- | --- | --- |
| ASCII | 4 | 100.0% |

#### Most frequent character per block

##### *ASCII*

| Value | Count | Frequency (%) |
| --- | --- | --- |
| N | 3 | 75.0% |
| S | 1 | 25.0% |

TABAGISMO  
Categorical

|  |  |
| --- | --- |
| Distinct | 2 |
| Distinct (%) | 50.0% |
| Missing | 0 |
| Missing (%) | 0.0% |
| Memory size | 64.0 B |

|  |  |
| --- | --- |
| N | 3 |
| S | 1 |

More details

- Overview
- Categories
- Words
- Characters

Length

|  |  |
| --- | --- |
| Max length | 1 |
| Median length | 1 |
| Mean length | 1 |
| Min length | 1 |

Characters and Unicode

|  |  |
| --- | --- |
| Total characters | 4 |
| Distinct characters | 2 |
| Distinct categories | 1 ? |
| Distinct scripts | 1 ? |
| Distinct blocks | 1 ? |

The Unicode Standard assigns character properties to each code point, which can be used to analyse textual variables.

Unique

|  |  |
| --- | --- |
| Unique | 1 ? |
| Unique (%) | 25.0% |

Sample

|  |  |
| --- | --- |
| 1st row | S |
| 2nd row | N |
| 3rd row | N |
| 4th row | N |

#### Common Values

| Value | Count | Frequency (%) |
| --- | --- | --- |
| N | 3 | 75.0% |
| S | 1 | 25.0% |

#### Length

xml version="1.0" encoding="utf-8" standalone="no"?2023-10-31T16:42:35.592490image/svg+xmlMatplotlib v3.6.0, https://matplotlib.org/ 

Histogram of lengths of the category

#### Common Values (Plot)

xml version="1.0" encoding="utf-8" standalone="no"?2023-10-31T16:42:35.756448image/svg+xmlMatplotlib v3.6.0, https://matplotlib.org/

| Value | Count | Frequency (%) |
| --- | --- | --- |
| n | 3 | 75.0% |
| s | 1 | 25.0% |

- Characters
- Categories
- Scripts
- Blocks

#### Most occurring characters

| Value | Count | Frequency (%) |
| --- | --- | --- |
| N | 3 | 75.0% |
| S | 1 | 25.0% |

#### Most occurring categories

| Value | Count | Frequency (%) |
| --- | --- | --- |
| Uppercase Letter | 4 | 100.0% |

#### Most frequent character per category

##### *Uppercase Letter*

| Value | Count | Frequency (%) |
| --- | --- | --- |
| N | 3 | 75.0% |
| S | 1 | 25.0% |

#### Most occurring scripts

| Value | Count | Frequency (%) |
| --- | --- | --- |
| Latin | 4 | 100.0% |

#### Most frequent character per script

##### *Latin*

| Value | Count | Frequency (%) |
| --- | --- | --- |
| N | 3 | 75.0% |
| S | 1 | 25.0% |

#### Most occurring blocks

| Value | Count | Frequency (%) |
| --- | --- | --- |
| ASCII | 4 | 100.0% |

#### Most frequent character per block

##### *ASCII*

| Value | Count | Frequency (%) |
| --- | --- | --- |
| N | 3 | 75.0% |
| S | 1 | 25.0% |

motMudEsquema  
Categorical

|  |  |
| --- | --- |
| Distinct | 1 |
| Distinct (%) | 25.0% |
| Missing | 0 |
| Missing (%) | 0.0% |
| Memory size | 64.0 B |

|  |  |
| --- | --- |
| Nulo | 4 |

More details

- Overview
- Categories
- Words
- Characters

Length

|  |  |
| --- | --- |
| Max length | 4 |
| Median length | 4 |
| Mean length | 4 |
| Min length | 4 |

Characters and Unicode

|  |  |
| --- | --- |
| Total characters | 16 |
| Distinct characters | 4 |
| Distinct categories | 2 ? |
| Distinct scripts | 1 ? |
| Distinct blocks | 1 ? |

The Unicode Standard assigns character properties to each code point, which can be used to analyse textual variables.

Unique

|  |  |
| --- | --- |
| Unique | 0 ? |
| Unique (%) | 0.0% |

Sample

|  |  |
| --- | --- |
| 1st row | Nulo |
| 2nd row | Nulo |
| 3rd row | Nulo |
| 4th row | Nulo |

#### Common Values

| Value | Count | Frequency (%) |
| --- | --- | --- |
| Nulo | 4 | 100.0% |

#### Length

xml version="1.0" encoding="utf-8" standalone="no"?2023-10-31T16:42:35.886027image/svg+xmlMatplotlib v3.6.0, https://matplotlib.org/ 

Histogram of lengths of the category

#### Common Values (Plot)

xml version="1.0" encoding="utf-8" standalone="no"?2023-10-31T16:42:36.041305image/svg+xmlMatplotlib v3.6.0, https://matplotlib.org/

| Value | Count | Frequency (%) |
| --- | --- | --- |
| nulo | 4 | 100.0% |

- Characters
- Categories
- Scripts
- Blocks

#### Most occurring characters

| Value | Count | Frequency (%) |
| --- | --- | --- |
| N | 4 | 25.0% |
| u | 4 | 25.0% |
| l | 4 | 25.0% |
| o | 4 | 25.0% |

#### Most occurring categories

| Value | Count | Frequency (%) |
| --- | --- | --- |
| Lowercase Letter | 12 | 75.0% |
| Uppercase Letter | 4 | 25.0% |

#### Most frequent character per category

##### *Lowercase Letter*

| Value | Count | Frequency (%) |
| --- | --- | --- |
| u | 4 | 33.3% |
| l | 4 | 33.3% |
| o | 4 | 33.3% |

##### *Uppercase Letter*

| Value | Count | Frequency (%) |
| --- | --- | --- |
| N | 4 | 100.0% |

#### Most occurring scripts

| Value | Count | Frequency (%) |
| --- | --- | --- |
| Latin | 16 | 100.0% |

#### Most frequent character per script

##### *Latin*

| Value | Count | Frequency (%) |
| --- | --- | --- |
| N | 4 | 25.0% |
| u | 4 | 25.0% |
| l | 4 | 25.0% |
| o | 4 | 25.0% |

#### Most occurring blocks

| Value | Count | Frequency (%) |
| --- | --- | --- |
| ASCII | 16 | 100.0% |

#### Most frequent character per block

##### *ASCII*

| Value | Count | Frequency (%) |
| --- | --- | --- |
| N | 4 | 25.0% |
| u | 4 | 25.0% |
| l | 4 | 25.0% |
| o | 4 | 25.0% |

tipoTrat  
Categorical

`HIGH CORRELATION`  `UNIFORM`

|  |  |
| --- | --- |
| Distinct | 2 |
| Distinct (%) | 50.0% |
| Missing | 0 |
| Missing (%) | 0.0% |
| Memory size | 64.0 B |

|  |  |
| --- | --- |
| Auto-Administrado | 2 |
| Supervisionado | 2 |

More details

- Overview
- Categories
- Words
- Characters

Length

|  |  |
| --- | --- |
| Max length | 17 |
| Median length | 15.5 |
| Mean length | 15.5 |
| Min length | 14 |

Characters and Unicode

|  |  |
| --- | --- |
| Total characters | 62 |
| Distinct characters | 16 |
| Distinct categories | 3 ? |
| Distinct scripts | 2 ? |
| Distinct blocks | 1 ? |

The Unicode Standard assigns character properties to each code point, which can be used to analyse textual variables.

Unique

|  |  |
| --- | --- |
| Unique | 0 ? |
| Unique (%) | 0.0% |

Sample

|  |  |
| --- | --- |
| 1st row | Auto-Administrado |
| 2nd row | Supervisionado |
| 3rd row | Supervisionado |
| 4th row | Auto-Administrado |

#### Common Values

| Value | Count | Frequency (%) |
| --- | --- | --- |
| Auto-Administrado | 2 | 50.0% |
| Supervisionado | 2 | 50.0% |

#### Length

xml version="1.0" encoding="utf-8" standalone="no"?2023-10-31T16:42:36.170774image/svg+xmlMatplotlib v3.6.0, https://matplotlib.org/ 

Histogram of lengths of the category

#### Common Values (Plot)

xml version="1.0" encoding="utf-8" standalone="no"?2023-10-31T16:42:37.186168image/svg+xmlMatplotlib v3.6.0, https://matplotlib.org/

| Value | Count | Frequency (%) |
| --- | --- | --- |
| auto-administrado | 2 | 50.0% |
| supervisionado | 2 | 50.0% |

- Characters
- Categories
- Scripts
- Blocks

#### Most occurring characters

| Value | Count | Frequency (%) |
| --- | --- | --- |
| o | 8 | 12.9% |
| i | 8 | 12.9% |
| d | 6 | 9.7% |
| A | 4 | 6.5% |
| u | 4 | 6.5% |
| t | 4 | 6.5% |
| n | 4 | 6.5% |
| s | 4 | 6.5% |
| r | 4 | 6.5% |
| a | 4 | 6.5% |
| Other values (6) | 12 | 19.4% |

#### Most occurring categories

| Value | Count | Frequency (%) |
| --- | --- | --- |
| Lowercase Letter | 54 | 87.1% |
| Uppercase Letter | 6 | 9.7% |
| Dash Punctuation | 2 | 3.2% |

#### Most frequent character per category

##### *Lowercase Letter*

| Value | Count | Frequency (%) |
| --- | --- | --- |
| o | 8 | 14.8% |
| i | 8 | 14.8% |
| d | 6 | 11.1% |
| u | 4 | 7.4% |
| t | 4 | 7.4% |
| n | 4 | 7.4% |
| s | 4 | 7.4% |
| r | 4 | 7.4% |
| a | 4 | 7.4% |
| m | 2 | 3.7% |
| Other values (3) | 6 | 11.1% |

##### *Uppercase Letter*

| Value | Count | Frequency (%) |
| --- | --- | --- |
| A | 4 | 66.7% |
| S | 2 | 33.3% |

##### *Dash Punctuation*

| Value | Count | Frequency (%) |
| --- | --- | --- |
| - | 2 | 100.0% |

#### Most occurring scripts

| Value | Count | Frequency (%) |
| --- | --- | --- |
| Latin | 60 | 96.8% |
| Common | 2 | 3.2% |

#### Most frequent character per script

##### *Latin*

| Value | Count | Frequency (%) |
| --- | --- | --- |
| o | 8 | 13.3% |
| i | 8 | 13.3% |
| d | 6 | 10.0% |
| A | 4 | 6.7% |
| u | 4 | 6.7% |
| t | 4 | 6.7% |
| n | 4 | 6.7% |
| s | 4 | 6.7% |
| r | 4 | 6.7% |
| a | 4 | 6.7% |
| Other values (5) | 10 | 16.7% |

##### *Common*

| Value | Count | Frequency (%) |
| --- | --- | --- |
| - | 2 | 100.0% |

#### Most occurring blocks

| Value | Count | Frequency (%) |
| --- | --- | --- |
| ASCII | 62 | 100.0% |

#### Most frequent character per block

##### *ASCII*

| Value | Count | Frequency (%) |
| --- | --- | --- |
| o | 8 | 12.9% |
| i | 8 | 12.9% |
| d | 6 | 9.7% |
| A | 4 | 6.5% |
| u | 4 | 6.5% |
| t | 4 | 6.5% |
| n | 4 | 6.5% |
| s | 4 | 6.5% |
| r | 4 | 6.5% |
| a | 4 | 6.5% |
| Other values (6) | 12 | 19.4% |

idade  
Categorical

|  |  |
| --- | --- |
| Distinct | 2 |
| Distinct (%) | 50.0% |
| Missing | 0 |
| Missing (%) | 0.0% |
| Memory size | 64.0 B |

|  |  |
| --- | --- |
| 40\_54 | 3 |
| 23\_39 | 1 |

More details

- Overview
- Categories
- Words
- Characters

Length

|  |  |
| --- | --- |
| Max length | 5 |
| Median length | 5 |
| Mean length | 5 |
| Min length | 5 |

Characters and Unicode

|  |  |
| --- | --- |
| Total characters | 20 |
| Distinct characters | 7 |
| Distinct categories | 2 ? |
| Distinct scripts | 1 ? |
| Distinct blocks | 1 ? |

The Unicode Standard assigns character properties to each code point, which can be used to analyse textual variables.

Unique

|  |  |
| --- | --- |
| Unique | 1 ? |
| Unique (%) | 25.0% |

Sample

|  |  |
| --- | --- |
| 1st row | 40\_54 |
| 2nd row | 40\_54 |
| 3rd row | 40\_54 |
| 4th row | 23\_39 |

#### Common Values

| Value | Count | Frequency (%) |
| --- | --- | --- |
| 40\_54 | 3 | 75.0% |
| 23\_39 | 1 | 25.0% |

#### Length

xml version="1.0" encoding="utf-8" standalone="no"?2023-10-31T16:42:37.358382image/svg+xmlMatplotlib v3.6.0, https://matplotlib.org/ 

Histogram of lengths of the category

#### Common Values (Plot)

xml version="1.0" encoding="utf-8" standalone="no"?2023-10-31T16:42:37.533615image/svg+xmlMatplotlib v3.6.0, https://matplotlib.org/

| Value | Count | Frequency (%) |
| --- | --- | --- |
| 40\_54 | 3 | 75.0% |
| 23\_39 | 1 | 25.0% |

- Characters
- Categories
- Scripts
- Blocks

#### Most occurring characters

| Value | Count | Frequency (%) |
| --- | --- | --- |
| 4 | 6 | 30.0% |
| \_ | 4 | 20.0% |
| 0 | 3 | 15.0% |
| 5 | 3 | 15.0% |
| 3 | 2 | 10.0% |
| 2 | 1 | 5.0% |
| 9 | 1 | 5.0% |

#### Most occurring categories

| Value | Count | Frequency (%) |
| --- | --- | --- |
| Decimal Number | 16 | 80.0% |
| Connector Punctuation | 4 | 20.0% |

#### Most frequent character per category

##### *Decimal Number*

| Value | Count | Frequency (%) |
| --- | --- | --- |
| 4 | 6 | 37.5% |
| 0 | 3 | 18.8% |
| 5 | 3 | 18.8% |
| 3 | 2 | 12.5% |
| 2 | 1 | 6.2% |
| 9 | 1 | 6.2% |

##### *Connector Punctuation*

| Value | Count | Frequency (%) |
| --- | --- | --- |
| \_ | 4 | 100.0% |

#### Most occurring scripts

| Value | Count | Frequency (%) |
| --- | --- | --- |
| Common | 20 | 100.0% |

#### Most frequent character per script

##### *Common*

| Value | Count | Frequency (%) |
| --- | --- | --- |
| 4 | 6 | 30.0% |
| \_ | 4 | 20.0% |
| 0 | 3 | 15.0% |
| 5 | 3 | 15.0% |
| 3 | 2 | 10.0% |
| 2 | 1 | 5.0% |
| 9 | 1 | 5.0% |

#### Most occurring blocks

| Value | Count | Frequency (%) |
| --- | --- | --- |
| ASCII | 20 | 100.0% |

#### Most frequent character per block

##### *ASCII*

| Value | Count | Frequency (%) |
| --- | --- | --- |
| 4 | 6 | 30.0% |
| \_ | 4 | 20.0% |
| 0 | 3 | 15.0% |
| 5 | 3 | 15.0% |
| 3 | 2 | 10.0% |
| 2 | 1 | 5.0% |
| 9 | 1 | 5.0% |

HISTOPATOL  
Categorical

|  |  |
| --- | --- |
| Distinct | 1 |
| Distinct (%) | 25.0% |
| Missing | 0 |
| Missing (%) | 0.0% |
| Memory size | 64.0 B |

|  |  |
| --- | --- |
| N/realiz | 4 |

More details

- Overview
- Categories
- Words
- Characters

Length

|  |  |
| --- | --- |
| Max length | 8 |
| Median length | 8 |
| Mean length | 8 |
| Min length | 8 |

Characters and Unicode

|  |  |
| --- | --- |
| Total characters | 32 |
| Distinct characters | 8 |
| Distinct categories | 3 ? |
| Distinct scripts | 2 ? |
| Distinct blocks | 1 ? |

The Unicode Standard assigns character properties to each code point, which can be used to analyse textual variables.

Unique

|  |  |
| --- | --- |
| Unique | 0 ? |
| Unique (%) | 0.0% |

Sample

|  |  |
| --- | --- |
| 1st row | N/realiz |
| 2nd row | N/realiz |
| 3rd row | N/realiz |
| 4th row | N/realiz |

#### Common Values

| Value | Count | Frequency (%) |
| --- | --- | --- |
| N/realiz | 4 | 100.0% |

#### Length

xml version="1.0" encoding="utf-8" standalone="no"?2023-10-31T16:42:37.681323image/svg+xmlMatplotlib v3.6.0, https://matplotlib.org/ 

Histogram of lengths of the category

#### Common Values (Plot)

xml version="1.0" encoding="utf-8" standalone="no"?2023-10-31T16:42:37.878417image/svg+xmlMatplotlib v3.6.0, https://matplotlib.org/

| Value | Count | Frequency (%) |
| --- | --- | --- |
| n/realiz | 4 | 100.0% |

- Characters
- Categories
- Scripts
- Blocks

#### Most occurring characters

| Value | Count | Frequency (%) |
| --- | --- | --- |
| N | 4 | 12.5% |
| / | 4 | 12.5% |
| r | 4 | 12.5% |
| e | 4 | 12.5% |
| a | 4 | 12.5% |
| l | 4 | 12.5% |
| i | 4 | 12.5% |
| z | 4 | 12.5% |

#### Most occurring categories

| Value | Count | Frequency (%) |
| --- | --- | --- |
| Lowercase Letter | 24 | 75.0% |
| Uppercase Letter | 4 | 12.5% |
| Other Punctuation | 4 | 12.5% |

#### Most frequent character per category

##### *Lowercase Letter*

| Value | Count | Frequency (%) |
| --- | --- | --- |
| r | 4 | 16.7% |
| e | 4 | 16.7% |
| a | 4 | 16.7% |
| l | 4 | 16.7% |
| i | 4 | 16.7% |
| z | 4 | 16.7% |

##### *Uppercase Letter*

| Value | Count | Frequency (%) |
| --- | --- | --- |
| N | 4 | 100.0% |

##### *Other Punctuation*

| Value | Count | Frequency (%) |
| --- | --- | --- |
| / | 4 | 100.0% |

#### Most occurring scripts

| Value | Count | Frequency (%) |
| --- | --- | --- |
| Latin | 28 | 87.5% |
| Common | 4 | 12.5% |

#### Most frequent character per script

##### *Latin*

| Value | Count | Frequency (%) |
| --- | --- | --- |
| N | 4 | 14.3% |
| r | 4 | 14.3% |
| e | 4 | 14.3% |
| a | 4 | 14.3% |
| l | 4 | 14.3% |
| i | 4 | 14.3% |
| z | 4 | 14.3% |

##### *Common*

| Value | Count | Frequency (%) |
| --- | --- | --- |
| / | 4 | 100.0% |

#### Most occurring blocks

| Value | Count | Frequency (%) |
| --- | --- | --- |
| ASCII | 32 | 100.0% |

#### Most frequent character per block

##### *ASCII*

| Value | Count | Frequency (%) |
| --- | --- | --- |
| N | 4 | 12.5% |
| / | 4 | 12.5% |
| r | 4 | 12.5% |
| e | 4 | 12.5% |
| a | 4 | 12.5% |
| l | 4 | 12.5% |
| i | 4 | 12.5% |
| z | 4 | 12.5% |

Status\_Resistencia  
Categorical

|  |  |
| --- | --- |
| Distinct | 1 |
| Distinct (%) | 25.0% |
| Missing | 0 |
| Missing (%) | 0.0% |
| Memory size | 64.0 B |

|  |  |
| --- | --- |
| 0 | 4 |

More details

- Overview
- Categories
- Words
- Characters

Length

|  |  |
| --- | --- |
| Max length | 1 |
| Median length | 1 |
| Mean length | 1 |
| Min length | 1 |

Characters and Unicode

|  |  |
| --- | --- |
| Total characters | 4 |
| Distinct characters | 1 |
| Distinct categories | 1 ? |
| Distinct scripts | 1 ? |
| Distinct blocks | 1 ? |

The Unicode Standard assigns character properties to each code point, which can be used to analyse textual variables.

Unique

|  |  |
| --- | --- |
| Unique | 0 ? |
| Unique (%) | 0.0% |

Sample

|  |  |
| --- | --- |
| 1st row | 0 |
| 2nd row | 0 |
| 3rd row | 0 |
| 4th row | 0 |

#### Common Values

| Value | Count | Frequency (%) |
| --- | --- | --- |
| 0 | 4 | 100.0% |

#### Length

xml version="1.0" encoding="utf-8" standalone="no"?2023-10-31T16:42:38.035605image/svg+xmlMatplotlib v3.6.0, https://matplotlib.org/ 

Histogram of lengths of the category

#### Common Values (Plot)

xml version="1.0" encoding="utf-8" standalone="no"?2023-10-31T16:42:38.183478image/svg+xmlMatplotlib v3.6.0, https://matplotlib.org/

| Value | Count | Frequency (%) |
| --- | --- | --- |
| 0 | 4 | 100.0% |

- Characters
- Categories
- Scripts
- Blocks

#### Most occurring characters

| Value | Count | Frequency (%) |
| --- | --- | --- |
| 0 | 4 | 100.0% |

#### Most occurring categories

| Value | Count | Frequency (%) |
| --- | --- | --- |
| Decimal Number | 4 | 100.0% |

#### Most frequent character per category

##### *Decimal Number*

| Value | Count | Frequency (%) |
| --- | --- | --- |
| 0 | 4 | 100.0% |

#### Most occurring scripts

| Value | Count | Frequency (%) |
| --- | --- | --- |
| Common | 4 | 100.0% |

#### Most frequent character per script

##### *Common*

| Value | Count | Frequency (%) |
| --- | --- | --- |
| 0 | 4 | 100.0% |

#### Most occurring blocks

| Value | Count | Frequency (%) |
| --- | --- | --- |
| ASCII | 4 | 100.0% |

#### Most frequent character per block

##### *ASCII*

| Value | Count | Frequency (%) |
| --- | --- | --- |
| 0 | 4 | 100.0% |

Cluster  
Categorical

|  |  |
| --- | --- |
| Distinct | 1 |
| Distinct (%) | 25.0% |
| Missing | 0 |
| Missing (%) | 0.0% |
| Memory size | 64.0 B |

|  |  |
| --- | --- |
| 0 | 4 |

More details

- Overview
- Categories
- Words
- Characters

Length

|  |  |
| --- | --- |
| Max length | 1 |
| Median length | 1 |
| Mean length | 1 |
| Min length | 1 |

Characters and Unicode

|  |  |
| --- | --- |
| Total characters | 4 |
| Distinct characters | 1 |
| Distinct categories | 1 ? |
| Distinct scripts | 1 ? |
| Distinct blocks | 1 ? |

The Unicode Standard assigns character properties to each code point, which can be used to analyse textual variables.

Unique

|  |  |
| --- | --- |
| Unique | 0 ? |
| Unique (%) | 0.0% |

Sample

|  |  |
| --- | --- |
| 1st row | 0 |
| 2nd row | 0 |
| 3rd row | 0 |
| 4th row | 0 |

#### Common Values

| Value | Count | Frequency (%) |
| --- | --- | --- |
| 0 | 4 | 100.0% |

#### Length

xml version="1.0" encoding="utf-8" standalone="no"?2023-10-31T16:42:38.300368image/svg+xmlMatplotlib v3.6.0, https://matplotlib.org/ 

Histogram of lengths of the category

#### Common Values (Plot)

xml version="1.0" encoding="utf-8" standalone="no"?2023-10-31T16:42:38.439322image/svg+xmlMatplotlib v3.6.0, https://matplotlib.org/

| Value | Count | Frequency (%) |
| --- | --- | --- |
| 0 | 4 | 100.0% |

- Characters
- Categories
- Scripts
- Blocks

#### Most occurring characters

| Value | Count | Frequency (%) |
| --- | --- | --- |
| 0 | 4 | 100.0% |

#### Most occurring categories

| Value | Count | Frequency (%) |
| --- | --- | --- |
| Decimal Number | 4 | 100.0% |

#### Most frequent character per category

##### *Decimal Number*

| Value | Count | Frequency (%) |
| --- | --- | --- |
| 0 | 4 | 100.0% |

#### Most occurring scripts

| Value | Count | Frequency (%) |
| --- | --- | --- |
| Common | 4 | 100.0% |

#### Most frequent character per script

##### *Common*

| Value | Count | Frequency (%) |
| --- | --- | --- |
| 0 | 4 | 100.0% |

#### Most occurring blocks

| Value | Count | Frequency (%) |
| --- | --- | --- |
| ASCII | 4 | 100.0% |

#### Most frequent character per block

##### *ASCII*

| Value | Count | Frequency (%) |
| --- | --- | --- |
| 0 | 4 | 100.0% |

Probabilidade  
Categorical

`HIGH CORRELATION`  `UNIFORM`  `UNIQUE`

|  |  |
| --- | --- |
| Distinct | 4 |
| Distinct (%) | 100.0% |
| Missing | 0 |
| Missing (%) | 0.0% |
| Memory size | 64.0 B |

|  |  |
| --- | --- |
| 0.6120194388542883 | 1 |
| 0.6292858855467693 | 1 |
| 0.717662238501694 | 1 |
| 0.6855705311276303 | 1 |

More details

- Overview
- Categories
- Words
- Characters

Length

|  |  |
| --- | --- |
| Max length | 18 |
| Median length | 18 |
| Mean length | 17.75 |
| Min length | 17 |

Characters and Unicode

|  |  |
| --- | --- |
| Total characters | 71 |
| Distinct characters | 11 |
| Distinct categories | 2 ? |
| Distinct scripts | 1 ? |
| Distinct blocks | 1 ? |

The Unicode Standard assigns character properties to each code point, which can be used to analyse textual variables.

Unique

|  |  |
| --- | --- |
| Unique | 4 ? |
| Unique (%) | 100.0% |

Sample

|  |  |
| --- | --- |
| 1st row | 0.6120194388542883 |
| 2nd row | 0.6292858855467693 |
| 3rd row | 0.717662238501694 |
| 4th row | 0.6855705311276303 |

#### Common Values

| Value | Count | Frequency (%) |
| --- | --- | --- |
| 0.6120194388542883 | 1 | 25.0% |
| 0.6292858855467693 | 1 | 25.0% |
| 0.717662238501694 | 1 | 25.0% |
| 0.6855705311276303 | 1 | 25.0% |

#### Length

xml version="1.0" encoding="utf-8" standalone="no"?2023-10-31T16:42:38.561725image/svg+xmlMatplotlib v3.6.0, https://matplotlib.org/ 

Histogram of lengths of the category

#### Common Values (Plot)

xml version="1.0" encoding="utf-8" standalone="no"?2023-10-31T16:42:38.738831image/svg+xmlMatplotlib v3.6.0, https://matplotlib.org/

| Value | Count | Frequency (%) |
| --- | --- | --- |
| 0.6120194388542883 | 1 | 25.0% |
| 0.6292858855467693 | 1 | 25.0% |
| 0.717662238501694 | 1 | 25.0% |
| 0.6855705311276303 | 1 | 25.0% |

- Characters
- Categories
- Scripts
- Blocks

#### Most occurring characters

| Value | Count | Frequency (%) |
| --- | --- | --- |
| 6 | 9 | 12.7% |
| 8 | 9 | 12.7% |
| 0 | 8 | 11.3% |
| 5 | 8 | 11.3% |
| 2 | 7 | 9.9% |
| 3 | 7 | 9.9% |
| 1 | 6 | 8.5% |
| 7 | 5 | 7.0% |
| . | 4 | 5.6% |
| 9 | 4 | 5.6% |

#### Most occurring categories

| Value | Count | Frequency (%) |
| --- | --- | --- |
| Decimal Number | 67 | 94.4% |
| Other Punctuation | 4 | 5.6% |

#### Most frequent character per category

##### *Decimal Number*

| Value | Count | Frequency (%) |
| --- | --- | --- |
| 6 | 9 | 13.4% |
| 8 | 9 | 13.4% |
| 0 | 8 | 11.9% |
| 5 | 8 | 11.9% |
| 2 | 7 | 10.4% |
| 3 | 7 | 10.4% |
| 1 | 6 | 9.0% |
| 7 | 5 | 7.5% |
| 9 | 4 | 6.0% |
| 4 | 4 | 6.0% |

##### *Other Punctuation*

| Value | Count | Frequency (%) |
| --- | --- | --- |
| . | 4 | 100.0% |

#### Most occurring scripts

| Value | Count | Frequency (%) |
| --- | --- | --- |
| Common | 71 | 100.0% |

#### Most frequent character per script

##### *Common*

| Value | Count | Frequency (%) |
| --- | --- | --- |
| 6 | 9 | 12.7% |
| 8 | 9 | 12.7% |
| 0 | 8 | 11.3% |
| 5 | 8 | 11.3% |
| 2 | 7 | 9.9% |
| 3 | 7 | 9.9% |
| 1 | 6 | 8.5% |
| 7 | 5 | 7.0% |
| . | 4 | 5.6% |
| 9 | 4 | 5.6% |

#### Most occurring blocks

| Value | Count | Frequency (%) |
| --- | --- | --- |
| ASCII | 71 | 100.0% |

#### Most frequent character per block

##### *ASCII*

| Value | Count | Frequency (%) |
| --- | --- | --- |
| 6 | 9 | 12.7% |
| 8 | 9 | 12.7% |
| 0 | 8 | 11.3% |
| 5 | 8 | 11.3% |
| 2 | 7 | 9.9% |
| 3 | 7 | 9.9% |
| 1 | 6 | 8.5% |
| 7 | 5 | 7.0% |
| . | 4 | 5.6% |
| 9 | 4 | 5.6% |

# Correlations

- Auto

- Heatmap
- Table

xml version="1.0" encoding="utf-8" standalone="no"?2023-10-31T16:42:38.907470image/svg+xmlMatplotlib v3.6.0, https://matplotlib.org/

|  | faixaEtaria | sexo | ESCOLARID | TIPOCUP | sitAtual | tipoCaso | FORMACLIN1 | classif | descoberta | BACOUTRO | cultEsc | RX | hiv | aids | DROGADICAO | TABAGISMO | tipoTrat | idade | Probabilidade |
| --- | --- | --- | --- | --- | --- | --- | --- | --- | --- | --- | --- | --- | --- | --- | --- | --- | --- | --- | --- |
| faixaEtaria | 1.000 | 0.000 | 0.000 | 0.000 | 0.000 | 0.000 | 0.707 | 0.000 | 0.000 | 0.000 | 0.707 | 0.000 | 0.707 | 0.707 | 0.707 | 0.707 | 0.707 | 0.707 | 1.000 |
| sexo | 0.000 | 1.000 | 0.707 | 0.000 | 0.000 | 0.000 | 0.000 | 0.000 | 0.707 | 0.707 | 0.000 | 0.000 | 0.000 | 0.000 | 0.000 | 0.000 | 0.000 | 0.000 | 1.000 |
| ESCOLARID | 0.000 | 0.707 | 1.000 | 0.707 | 0.000 | 0.707 | 0.000 | 0.000 | 1.000 | 0.000 | 0.000 | 0.000 | 0.000 | 0.000 | 0.000 | 0.000 | 0.707 | 0.000 | 1.000 |
| TIPOCUP | 0.000 | 0.000 | 0.707 | 1.000 | 0.000 | 0.000 | 0.000 | 0.000 | 0.707 | 0.707 | 0.000 | 0.000 | 0.000 | 0.000 | 0.000 | 0.000 | 0.000 | 0.000 | 1.000 |
| sitAtual | 0.000 | 0.000 | 0.000 | 0.000 | 1.000 | 0.000 | 0.000 | 0.000 | 0.000 | 0.707 | 0.000 | 0.000 | 0.000 | 0.000 | 0.000 | 0.000 | 0.000 | 0.000 | 1.000 |
| tipoCaso | 0.000 | 0.000 | 0.707 | 0.000 | 0.000 | 1.000 | 0.000 | 0.707 | 0.707 | 0.000 | 0.000 | 0.000 | 0.000 | 0.000 | 0.000 | 0.000 | 0.000 | 0.000 | 1.000 |
| FORMACLIN1 | 0.707 | 0.000 | 0.000 | 0.000 | 0.000 | 0.000 | 1.000 | 0.707 | 0.000 | 0.707 | 0.000 | 0.000 | 0.000 | 0.000 | 0.000 | 0.000 | 0.000 | 0.000 | 1.000 |
| classif | 0.000 | 0.000 | 0.000 | 0.000 | 0.000 | 0.707 | 0.707 | 1.000 | 0.000 | 0.000 | 0.707 | 0.000 | 0.000 | 0.000 | 0.707 | 0.707 | 0.000 | 0.000 | 1.000 |
| descoberta | 0.000 | 0.707 | 1.000 | 0.707 | 0.000 | 0.707 | 0.000 | 0.000 | 1.000 | 0.000 | 0.000 | 0.000 | 0.000 | 0.000 | 0.000 | 0.000 | 0.707 | 0.000 | 1.000 |
| BACOUTRO | 0.000 | 0.707 | 0.000 | 0.707 | 0.707 | 0.000 | 0.707 | 0.000 | 0.000 | 1.000 | 0.707 | 0.707 | 0.000 | 0.000 | 0.707 | 0.707 | 0.000 | 0.000 | 1.000 |
| cultEsc | 0.707 | 0.000 | 0.000 | 0.000 | 0.000 | 0.000 | 0.000 | 0.707 | 0.000 | 0.707 | 1.000 | 0.000 | 0.000 | 0.000 | 0.000 | 0.000 | 0.000 | 0.000 | 1.000 |
| RX | 0.000 | 0.000 | 0.000 | 0.000 | 0.000 | 0.000 | 0.000 | 0.000 | 0.000 | 0.707 | 0.000 | 1.000 | 0.000 | 0.000 | 0.000 | 0.000 | 0.000 | 0.000 | 1.000 |
| hiv | 0.707 | 0.000 | 0.000 | 0.000 | 0.000 | 0.000 | 0.000 | 0.000 | 0.000 | 0.000 | 0.000 | 0.000 | 1.000 | 0.000 | 0.000 | 0.000 | 0.000 | 0.000 | 1.000 |
| aids | 0.707 | 0.000 | 0.000 | 0.000 | 0.000 | 0.000 | 0.000 | 0.000 | 0.000 | 0.000 | 0.000 | 0.000 | 0.000 | 1.000 | 0.000 | 0.000 | 0.000 | 0.000 | 1.000 |
| DROGADICAO | 0.707 | 0.000 | 0.000 | 0.000 | 0.000 | 0.000 | 0.000 | 0.707 | 0.000 | 0.707 | 0.000 | 0.000 | 0.000 | 0.000 | 1.000 | 0.000 | 0.000 | 0.000 | 1.000 |
| TABAGISMO | 0.707 | 0.000 | 0.000 | 0.000 | 0.000 | 0.000 | 0.000 | 0.707 | 0.000 | 0.707 | 0.000 | 0.000 | 0.000 | 0.000 | 0.000 | 1.000 | 0.000 | 0.000 | 1.000 |
| tipoTrat | 0.707 | 0.000 | 0.707 | 0.000 | 0.000 | 0.000 | 0.000 | 0.000 | 0.707 | 0.000 | 0.000 | 0.000 | 0.000 | 0.000 | 0.000 | 0.000 | 1.000 | 0.000 | 1.000 |
| idade | 0.707 | 0.000 | 0.000 | 0.000 | 0.000 | 0.000 | 0.000 | 0.000 | 0.000 | 0.000 | 0.000 | 0.000 | 0.000 | 0.000 | 0.000 | 0.000 | 0.000 | 1.000 | 1.000 |
| Probabilidade | 1.000 | 1.000 | 1.000 | 1.000 | 1.000 | 1.000 | 1.000 | 1.000 | 1.000 | 1.000 | 1.000 | 1.000 | 1.000 | 1.000 | 1.000 | 1.000 | 1.000 | 1.000 | 1.000 |

# Missing values

- Count
- Matrix

xml version="1.0" encoding="utf-8" standalone="no"?2023-10-31T16:42:29.561507image/svg+xmlMatplotlib v3.6.0, https://matplotlib.org/ 

A simple visualization of nullity by column.

xml version="1.0" encoding="utf-8" standalone="no"?2023-10-31T16:42:30.089571image/svg+xmlMatplotlib v3.6.0, https://matplotlib.org/ 

Nullity matrix is a data-dense display which lets you quickly visually pick out patterns in data completion.

# Sample

- First rows
- Last rows

|  | faixaEtaria | sexo | ESCOLARID | TIPOCUP | sitAtual | tipoCaso | FORMACLIN1 | classif | descoberta | bac | BACOUTRO | cultEsc | RX | NECROP | hiv | aids | DIABETES | ALCOOLISMO | MENTAL | DROGADICAO | TABAGISMO | motMudEsquema | tipoTrat | idade | HISTOPATOL | Status\_Resistencia | Cluster | Probabilidade |
| --- | --- | --- | --- | --- | --- | --- | --- | --- | --- | --- | --- | --- | --- | --- | --- | --- | --- | --- | --- | --- | --- | --- | --- | --- | --- | --- | --- | --- |
| 374 | 40\_49 | M | De 8 a 11 anos | Outra | Abandono | Novo | Ganglionar Periferica | Ext | Elucidacao Diagn. em Internacao | Neg | Pos | Neg | Normal | N/realiz | Pos | S | N | N | N | S | S | Nulo | Auto-Administrado | 40\_54 | N/realiz | 0 | 0 | 0.612019 |
| 969 | 30\_39 | F | De 4 a 7 anos | Dona de Casa | Abandono | Novo | Pul | Pul | Investigacao de Contatos | Neg | N/realiz | Pos | Normal | N/realiz | Pos | S | N | N | N | N | N | Nulo | Supervisionado | 40\_54 | N/realiz | 0 | 0 | 0.629286 |
| 258 | 30\_39 | M | De 12 a 14 anos | Outra | Cura | Recidiva | Pul | P+E | Demanda Ambulatorial | Neg | Neg | Pos | Susp TB | N/realiz | Pos | S | N | N | N | N | N | Nulo | Supervisionado | 40\_54 | N/realiz | 0 | 0 | 0.717662 |
| 1243 | 20\_29 | M | De 8 a 11 anos | Outra | Cura | Novo | Pul | Pul | Elucidacao Diagn. em Internacao | Neg | Neg | Pos | Susp TB | N/realiz | Neg | N | N | N | N | N | N | Nulo | Auto-Administrado | 23\_39 | N/realiz | 0 | 0 | 0.685571 |

|  | faixaEtaria | sexo | ESCOLARID | TIPOCUP | sitAtual | tipoCaso | FORMACLIN1 | classif | descoberta | bac | BACOUTRO | cultEsc | RX | NECROP | hiv | aids | DIABETES | ALCOOLISMO | MENTAL | DROGADICAO | TABAGISMO | motMudEsquema | tipoTrat | idade | HISTOPATOL | Status\_Resistencia | Cluster | Probabilidade |
| --- | --- | --- | --- | --- | --- | --- | --- | --- | --- | --- | --- | --- | --- | --- | --- | --- | --- | --- | --- | --- | --- | --- | --- | --- | --- | --- | --- | --- |
| 374 | 40\_49 | M | De 8 a 11 anos | Outra | Abandono | Novo | Ganglionar Periferica | Ext | Elucidacao Diagn. em Internacao | Neg | Pos | Neg | Normal | N/realiz | Pos | S | N | N | N | S | S | Nulo | Auto-Administrado | 40\_54 | N/realiz | 0 | 0 | 0.612019 |
| 969 | 30\_39 | F | De 4 a 7 anos | Dona de Casa | Abandono | Novo | Pul | Pul | Investigacao de Contatos | Neg | N/realiz | Pos | Normal | N/realiz | Pos | S | N | N | N | N | N | Nulo | Supervisionado | 40\_54 | N/realiz | 0 | 0 | 0.629286 |
| 258 | 30\_39 | M | De 12 a 14 anos | Outra | Cura | Recidiva | Pul | P+E | Demanda Ambulatorial | Neg | Neg | Pos | Susp TB | N/realiz | Pos | S | N | N | N | N | N | Nulo | Supervisionado | 40\_54 | N/realiz | 0 | 0 | 0.717662 |
| 1243 | 20\_29 | M | De 8 a 11 anos | Outra | Cura | Novo | Pul | Pul | Elucidacao Diagn. em Internacao | Neg | Neg | Pos | Susp TB | N/realiz | Neg | N | N | N | N | N | N | Nulo | Auto-Administrado | 23\_39 | N/realiz | 0 | 0 | 0.685571 |

Report generated by YData.

 
