# Supplementary material for: Model for predicting drug resistance based on the clinical profile of tuberculosis patients using machine learning techniques
Source: PeerJ Comput Sci. 2024 Oct 14;10:e2246. doi: 10.7717/peerj-cs.2246 (PMC11623081; doi:10.7717/peerj-cs.2246)
Supplement: Supplemental Information 2 [file peerj-cs-10-2246-s002.zip › code/EDA/tuberculosis_analysis.html]

Tuberculosis Analysis 

Toggle navigationTuberculosis Analysis

- Overview
- Variables

# Overview

- Overview
- Alerts 11
- Reproduction

Dataset statistics

|  |  |
| --- | --- |
| Number of variables | 26 |
| Number of observations | 1536 |
| Missing cells | 1516 |
| Missing cells (%) | 3.8% |
| Total size in memory | 312.1 KiB |
| Average record size in memory | 208.1 B |

Variable types

|  |  |
| --- | --- |
| Categorical | 25 |
| Numeric | 1 |

Alerts

| `tipoCaso` is highly imbalanced (86.1%) | Imbalance |
| `FORMACLIN1` is highly imbalanced (81.2%) | Imbalance |
| `classif` is highly imbalanced (58.8%) | Imbalance |
| `BACOUTRO` is highly imbalanced (69.2%) | Imbalance |
| `NECROP` is highly imbalanced (97.4%) | Imbalance |
| `hiv` is highly imbalanced (51.9%) | Imbalance |
| `DIABETES` is highly imbalanced (66.0%) | Imbalance |
| `MENTAL` is highly imbalanced (88.0%) | Imbalance |
| `HISTOPATOL` is highly imbalanced (75.4%) | Imbalance |
| `motMudEsquema` has 1516 (98.7%) missing values | Missing |
| `Status_Resistencia` has 768 (50.0%) zeros | Zeros |

Reproduction

|  |  |
| --- | --- |
| Analysis started | 2023-10-31 18:15:45.547735 |
| Analysis finished | 2023-10-31 18:15:45.745820 |
| Duration | 0.2 seconds |
| Software version | pandas-profiling v3.6.6 |
| Download configuration | config.json |

# Variables

Select ColumnsfaixaEtariasexoESCOLARIDTIPOCUPsitAtualtipoCasoFORMACLIN1classifdescobertabacBACOUTROcultEscRXNECROPhivaidsDIABETESALCOOLISMOMENTALDROGADICAOTABAGISMOmotMudEsquematipoTratidadeHISTOPATOLStatus\_Resistencia

faixaEtaria  
Categorical

|  |  |
| --- | --- |
| Distinct | 12 |
| Distinct (%) | 0.8% |
| Missing | 0 |
| Missing (%) | 0.0% |
| Memory size | 12.1 KiB |

|  |  |
| --- | --- |
| 20\_29 | 387 |
| 30\_39 | 372 |
| 40\_49 | 323 |
| 50\_59 | 191 |
| 60\_69 | 90 |
| Other values (7) | 173 |

More details

- Overview
- Categories

Unique

|  |  |
| --- | --- |
| Unique | 1 ? |
| Unique (%) | 0.1% |

Sample

|  |  |
| --- | --- |
| 1st row | 20\_29 |
| 2nd row | 40\_49 |
| 3rd row | 40\_49 |
| 4th row | 50\_59 |
| 5th row | 30\_39 |

#### Common Values

| Value | Count | Frequency (%) |
| --- | --- | --- |
| 20\_29 | 387 | 25.2% |
| 30\_39 | 372 | 24.2% |
| 40\_49 | 323 | 21.0% |
| 50\_59 | 191 | 12.4% |
| 60\_69 | 90 | 5.9% |
| 15\_19 | 87 | 5.7% |
| 70\_79 | 35 | 2.3% |
| 10\_14 | 22 | 1.4% |
| 05\_09 | 11 | 0.7% |
| Maior de 80 anos | 9 | 0.6% |
| Other values (2) | 9 | 0.6% |

sexo  
Categorical

|  |  |
| --- | --- |
| Distinct | 2 |
| Distinct (%) | 0.1% |
| Missing | 0 |
| Missing (%) | 0.0% |
| Memory size | 12.1 KiB |

|  |  |
| --- | --- |
| M | 1065 |
| F | 471 |

More details

- Overview
- Categories

Unique

|  |  |
| --- | --- |
| Unique | 0 ? |
| Unique (%) | 0.0% |

Sample

|  |  |
| --- | --- |
| 1st row | M |
| 2nd row | M |
| 3rd row | M |
| 4th row | M |
| 5th row | M |

#### Common Values

| Value | Count | Frequency (%) |
| --- | --- | --- |
| M | 1065 | 69.3% |
| F | 471 | 30.7% |

#### Common Values (Plot)

xml version="1.0" encoding="utf-8" standalone="no"?2023-10-31T15:15:45.925329image/svg+xmlMatplotlib v3.6.0, https://matplotlib.org/

ESCOLARID  
Categorical

|  |  |
| --- | --- |
| Distinct | 6 |
| Distinct (%) | 0.4% |
| Missing | 0 |
| Missing (%) | 0.0% |
| Memory size | 12.1 KiB |

|  |  |
| --- | --- |
| De 8 a 11 anos | 595 |
| De 4 a 7 anos | 524 |
| De 1 a 3 anos | 185 |
| De 12 a 14 anos | 119 |
| Nenhuma | 60 |

More details

- Overview
- Categories

Unique

|  |  |
| --- | --- |
| Unique | 0 ? |
| Unique (%) | 0.0% |

Sample

|  |  |
| --- | --- |
| 1st row | De 4 a 7 anos |
| 2nd row | De 4 a 7 anos |
| 3rd row | De 8 a 11 anos |
| 4th row | De 4 a 7 anos |
| 5th row | De 4 a 7 anos |

#### Common Values

| Value | Count | Frequency (%) |
| --- | --- | --- |
| De 8 a 11 anos | 595 | 38.7% |
| De 4 a 7 anos | 524 | 34.1% |
| De 1 a 3 anos | 185 | 12.0% |
| De 12 a 14 anos | 119 | 7.7% |
| Nenhuma | 60 | 3.9% |
| 15 anos e mais | 53 | 3.5% |

#### Common Values (Plot)

xml version="1.0" encoding="utf-8" standalone="no"?2023-10-31T15:15:46.079583image/svg+xmlMatplotlib v3.6.0, https://matplotlib.org/

TIPOCUP  
Categorical

|  |  |
| --- | --- |
| Distinct | 5 |
| Distinct (%) | 0.3% |
| Missing | 0 |
| Missing (%) | 0.0% |
| Memory size | 12.1 KiB |

|  |  |
| --- | --- |
| Outra | 1007 |
| Desempregado | 307 |
| Dona de Casa | 108 |
| Aposentado | 93 |
| Profissional de Saude | 21 |

More details

- Overview
- Categories

Unique

|  |  |
| --- | --- |
| Unique | 0 ? |
| Unique (%) | 0.0% |

Sample

|  |  |
| --- | --- |
| 1st row | Outra |
| 2nd row | Desempregado |
| 3rd row | Outra |
| 4th row | Outra |
| 5th row | Outra |

#### Common Values

| Value | Count | Frequency (%) |
| --- | --- | --- |
| Outra | 1007 | 65.6% |
| Desempregado | 307 | 20.0% |
| Dona de Casa | 108 | 7.0% |
| Aposentado | 93 | 6.1% |
| Profissional de Saude | 21 | 1.4% |

#### Common Values (Plot)

xml version="1.0" encoding="utf-8" standalone="no"?2023-10-31T15:15:46.253719image/svg+xmlMatplotlib v3.6.0, https://matplotlib.org/

sitAtual  
Categorical

|  |  |
| --- | --- |
| Distinct | 2 |
| Distinct (%) | 0.1% |
| Missing | 0 |
| Missing (%) | 0.0% |
| Memory size | 12.1 KiB |

|  |  |
| --- | --- |
| Cura | 1308 |
| Abandono | 228 |

More details

- Overview
- Categories

Unique

|  |  |
| --- | --- |
| Unique | 0 ? |
| Unique (%) | 0.0% |

Sample

|  |  |
| --- | --- |
| 1st row | Cura |
| 2nd row | Cura |
| 3rd row | Cura |
| 4th row | Cura |
| 5th row | Cura |

#### Common Values

| Value | Count | Frequency (%) |
| --- | --- | --- |
| Cura | 1308 | 85.2% |
| Abandono | 228 | 14.8% |

#### Common Values (Plot)

xml version="1.0" encoding="utf-8" standalone="no"?2023-10-31T15:15:46.407372image/svg+xmlMatplotlib v3.6.0, https://matplotlib.org/

tipoCaso  
Categorical

|  |  |
| --- | --- |
| Distinct | 4 |
| Distinct (%) | 0.3% |
| Missing | 0 |
| Missing (%) | 0.0% |
| Memory size | 12.1 KiB |

|  |  |
| --- | --- |
| Novo | 1477 |
| Recidiva | 32 |
| Retr Aband | 26 |
| Retrat apos falencia/resistencia | 1 |

More details

- Overview
- Categories

Unique

|  |  |
| --- | --- |
| Unique | 1 ? |
| Unique (%) | 0.1% |

Sample

|  |  |
| --- | --- |
| 1st row | Novo |
| 2nd row | Novo |
| 3rd row | Novo |
| 4th row | Novo |
| 5th row | Novo |

#### Common Values

| Value | Count | Frequency (%) |
| --- | --- | --- |
| Novo | 1477 | 96.2% |
| Recidiva | 32 | 2.1% |
| Retr Aband | 26 | 1.7% |
| Retrat apos falencia/resistencia | 1 | 0.1% |

#### Common Values (Plot)

xml version="1.0" encoding="utf-8" standalone="no"?2023-10-31T15:15:46.551510image/svg+xmlMatplotlib v3.6.0, https://matplotlib.org/

FORMACLIN1  
Categorical

|  |  |
| --- | --- |
| Distinct | 12 |
| Distinct (%) | 0.8% |
| Missing | 0 |
| Missing (%) | 0.0% |
| Memory size | 12.1 KiB |

|  |  |
| --- | --- |
| Pul | 1387 |
| Pleural | 85 |
| Ganglionar Periferica | 24 |
| Meningea | 8 |
| Oftalmica | 7 |
| Other values (7) | 25 |

More details

- Overview
- Categories

Unique

|  |  |
| --- | --- |
| Unique | 1 ? |
| Unique (%) | 0.1% |

Sample

|  |  |
| --- | --- |
| 1st row | Pul |
| 2nd row | Pul |
| 3rd row | Pul |
| 4th row | Pul |
| 5th row | Pul |

#### Common Values

| Value | Count | Frequency (%) |
| --- | --- | --- |
| Pul | 1387 | 90.3% |
| Pleural | 85 | 5.5% |
| Ganglionar Periferica | 24 | 1.6% |
| Meningea | 8 | 0.5% |
| Oftalmica | 7 | 0.5% |
| Outras | 6 | 0.4% |
| Pele | 5 | 0.3% |
| Miliar | 5 | 0.3% |
| Multiplos Orgaos | 3 | 0.2% |
| Ossea | 3 | 0.2% |
| Other values (2) | 3 | 0.2% |

classif  
Categorical

|  |  |
| --- | --- |
| Distinct | 4 |
| Distinct (%) | 0.3% |
| Missing | 0 |
| Missing (%) | 0.0% |
| Memory size | 12.1 KiB |

|  |  |
| --- | --- |
| Pul | 1281 |
| Ext | 146 |
| P+E | 106 |
| Dissem | 3 |

More details

- Overview
- Categories

Unique

|  |  |
| --- | --- |
| Unique | 0 ? |
| Unique (%) | 0.0% |

Sample

|  |  |
| --- | --- |
| 1st row | Pul |
| 2nd row | P+E |
| 3rd row | P+E |
| 4th row | P+E |
| 5th row | P+E |

#### Common Values

| Value | Count | Frequency (%) |
| --- | --- | --- |
| Pul | 1281 | 83.4% |
| Ext | 146 | 9.5% |
| P+E | 106 | 6.9% |
| Dissem | 3 | 0.2% |

#### Common Values (Plot)

xml version="1.0" encoding="utf-8" standalone="no"?2023-10-31T15:15:46.702881image/svg+xmlMatplotlib v3.6.0, https://matplotlib.org/

descoberta  
Categorical

|  |  |
| --- | --- |
| Distinct | 6 |
| Distinct (%) | 0.4% |
| Missing | 0 |
| Missing (%) | 0.0% |
| Memory size | 12.1 KiB |

|  |  |
| --- | --- |
| Demanda Ambulatorial | 701 |
| Urgencia / Emergencia | 376 |
| Elucidacao Diagn. em Internacao | 344 |
| Busca Ativa na Comunidade | 46 |
| Investigacao de Contatos | 41 |

More details

- Overview
- Categories

Unique

|  |  |
| --- | --- |
| Unique | 0 ? |
| Unique (%) | 0.0% |

Sample

|  |  |
| --- | --- |
| 1st row | Elucidacao Diagn. em Internacao |
| 2nd row | Demanda Ambulatorial |
| 3rd row | Elucidacao Diagn. em Internacao |
| 4th row | Demanda Ambulatorial |
| 5th row | Urgencia / Emergencia |

#### Common Values

| Value | Count | Frequency (%) |
| --- | --- | --- |
| Demanda Ambulatorial | 701 | 45.6% |
| Urgencia / Emergencia | 376 | 24.5% |
| Elucidacao Diagn. em Internacao | 344 | 22.4% |
| Busca Ativa na Comunidade | 46 | 3.0% |
| Investigacao de Contatos | 41 | 2.7% |
| Busca Ativa em Instituicao | 28 | 1.8% |

#### Common Values (Plot)

xml version="1.0" encoding="utf-8" standalone="no"?2023-10-31T15:15:46.874431image/svg+xmlMatplotlib v3.6.0, https://matplotlib.org/

bac  
Categorical

|  |  |
| --- | --- |
| Distinct | 3 |
| Distinct (%) | 0.2% |
| Missing | 0 |
| Missing (%) | 0.0% |
| Memory size | 12.1 KiB |

|  |  |
| --- | --- |
| Pos | 925 |
| Neg | 352 |
| N/realiz | 259 |

More details

- Overview
- Categories

Unique

|  |  |
| --- | --- |
| Unique | 0 ? |
| Unique (%) | 0.0% |

Sample

|  |  |
| --- | --- |
| 1st row | Pos |
| 2nd row | Pos |
| 3rd row | N/realiz |
| 4th row | Pos |
| 5th row | Pos |

#### Common Values

| Value | Count | Frequency (%) |
| --- | --- | --- |
| Pos | 925 | 60.2% |
| Neg | 352 | 22.9% |
| N/realiz | 259 | 16.9% |

#### Common Values (Plot)

xml version="1.0" encoding="utf-8" standalone="no"?2023-10-31T15:15:47.103148image/svg+xmlMatplotlib v3.6.0, https://matplotlib.org/

BACOUTRO  
Categorical

|  |  |
| --- | --- |
| Distinct | 4 |
| Distinct (%) | 0.3% |
| Missing | 0 |
| Missing (%) | 0.0% |
| Memory size | 12.1 KiB |

|  |  |
| --- | --- |
| N/realiz | 1365 |
| Neg | 103 |
| Pos | 67 |
| And | 1 |

More details

- Overview
- Categories

Unique

|  |  |
| --- | --- |
| Unique | 1 ? |
| Unique (%) | 0.1% |

Sample

|  |  |
| --- | --- |
| 1st row | N/realiz |
| 2nd row | N/realiz |
| 3rd row | Pos |
| 4th row | N/realiz |
| 5th row | N/realiz |

#### Common Values

| Value | Count | Frequency (%) |
| --- | --- | --- |
| N/realiz | 1365 | 88.9% |
| Neg | 103 | 6.7% |
| Pos | 67 | 4.4% |
| And | 1 | 0.1% |

#### Common Values (Plot)

xml version="1.0" encoding="utf-8" standalone="no"?2023-10-31T15:15:47.244841image/svg+xmlMatplotlib v3.6.0, https://matplotlib.org/

cultEsc  
Categorical

|  |  |
| --- | --- |
| Distinct | 4 |
| Distinct (%) | 0.3% |
| Missing | 0 |
| Missing (%) | 0.0% |
| Memory size | 12.1 KiB |

|  |  |
| --- | --- |
| Pos | 858 |
| N/realiz | 529 |
| Neg | 146 |
| And | 3 |

More details

- Overview
- Categories

Unique

|  |  |
| --- | --- |
| Unique | 0 ? |
| Unique (%) | 0.0% |

Sample

|  |  |
| --- | --- |
| 1st row | Pos |
| 2nd row | Pos |
| 3rd row | N/realiz |
| 4th row | Pos |
| 5th row | N/realiz |

#### Common Values

| Value | Count | Frequency (%) |
| --- | --- | --- |
| Pos | 858 | 55.9% |
| N/realiz | 529 | 34.4% |
| Neg | 146 | 9.5% |
| And | 3 | 0.2% |

#### Common Values (Plot)

xml version="1.0" encoding="utf-8" standalone="no"?2023-10-31T15:15:47.388900image/svg+xmlMatplotlib v3.6.0, https://matplotlib.org/

RX  
Categorical

|  |  |
| --- | --- |
| Distinct | 5 |
| Distinct (%) | 0.3% |
| Missing | 0 |
| Missing (%) | 0.0% |
| Memory size | 12.1 KiB |

|  |  |
| --- | --- |
| Susp TB | 941 |
| Susp c/cavid | 291 |
| N/realiz | 208 |
| Normal | 79 |
| Outra Patologia | 17 |

More details

- Overview
- Categories

Unique

|  |  |
| --- | --- |
| Unique | 0 ? |
| Unique (%) | 0.0% |

Sample

|  |  |
| --- | --- |
| 1st row | Susp c/cavid |
| 2nd row | Susp TB |
| 3rd row | Susp TB |
| 4th row | Susp TB |
| 5th row | Susp TB |

#### Common Values

| Value | Count | Frequency (%) |
| --- | --- | --- |
| Susp TB | 941 | 61.3% |
| Susp c/cavid | 291 | 18.9% |
| N/realiz | 208 | 13.5% |
| Normal | 79 | 5.1% |
| Outra Patologia | 17 | 1.1% |

#### Common Values (Plot)

xml version="1.0" encoding="utf-8" standalone="no"?2023-10-31T15:15:47.543314image/svg+xmlMatplotlib v3.6.0, https://matplotlib.org/

NECROP  
Categorical

|  |  |
| --- | --- |
| Distinct | 3 |
| Distinct (%) | 0.2% |
| Missing | 0 |
| Missing (%) | 0.0% |
| Memory size | 12.1 KiB |

|  |  |
| --- | --- |
| N/realiz | 1530 |
| Sugestivo TB | 4 |
| BAAR pos | 2 |

More details

- Overview
- Categories

Unique

|  |  |
| --- | --- |
| Unique | 0 ? |
| Unique (%) | 0.0% |

Sample

|  |  |
| --- | --- |
| 1st row | N/realiz |
| 2nd row | N/realiz |
| 3rd row | N/realiz |
| 4th row | N/realiz |
| 5th row | N/realiz |

#### Common Values

| Value | Count | Frequency (%) |
| --- | --- | --- |
| N/realiz | 1530 | 99.6% |
| Sugestivo TB | 4 | 0.3% |
| BAAR pos | 2 | 0.1% |

#### Common Values (Plot)

xml version="1.0" encoding="utf-8" standalone="no"?2023-10-31T15:15:47.697709image/svg+xmlMatplotlib v3.6.0, https://matplotlib.org/

hiv  
Categorical

|  |  |
| --- | --- |
| Distinct | 4 |
| Distinct (%) | 0.3% |
| Missing | 0 |
| Missing (%) | 0.0% |
| Memory size | 12.1 KiB |

|  |  |
| --- | --- |
| Neg | 1200 |
| Pos | 226 |
| N/realiz | 109 |
| And | 1 |

More details

- Overview
- Categories

Unique

|  |  |
| --- | --- |
| Unique | 1 ? |
| Unique (%) | 0.1% |

Sample

|  |  |
| --- | --- |
| 1st row | Neg |
| 2nd row | Neg |
| 3rd row | Pos |
| 4th row | Pos |
| 5th row | Pos |

#### Common Values

| Value | Count | Frequency (%) |
| --- | --- | --- |
| Neg | 1200 | 78.1% |
| Pos | 226 | 14.7% |
| N/realiz | 109 | 7.1% |
| And | 1 | 0.1% |

#### Common Values (Plot)

xml version="1.0" encoding="utf-8" standalone="no"?2023-10-31T15:15:47.836173image/svg+xmlMatplotlib v3.6.0, https://matplotlib.org/

aids  
Categorical

|  |  |
| --- | --- |
| Distinct | 2 |
| Distinct (%) | 0.1% |
| Missing | 0 |
| Missing (%) | 0.0% |
| Memory size | 12.1 KiB |

|  |  |
| --- | --- |
| N | 1329 |
| S | 207 |

More details

- Overview
- Categories

Unique

|  |  |
| --- | --- |
| Unique | 0 ? |
| Unique (%) | 0.0% |

Sample

|  |  |
| --- | --- |
| 1st row | N |
| 2nd row | N |
| 3rd row | S |
| 4th row | S |
| 5th row | S |

#### Common Values

| Value | Count | Frequency (%) |
| --- | --- | --- |
| N | 1329 | 86.5% |
| S | 207 | 13.5% |

#### Common Values (Plot)

xml version="1.0" encoding="utf-8" standalone="no"?2023-10-31T15:15:47.971738image/svg+xmlMatplotlib v3.6.0, https://matplotlib.org/

DIABETES  
Categorical

|  |  |
| --- | --- |
| Distinct | 2 |
| Distinct (%) | 0.1% |
| Missing | 0 |
| Missing (%) | 0.0% |
| Memory size | 12.1 KiB |

|  |  |
| --- | --- |
| N | 1439 |
| S | 97 |

More details

- Overview
- Categories

Unique

|  |  |
| --- | --- |
| Unique | 0 ? |
| Unique (%) | 0.0% |

Sample

|  |  |
| --- | --- |
| 1st row | N |
| 2nd row | N |
| 3rd row | N |
| 4th row | N |
| 5th row | N |

#### Common Values

| Value | Count | Frequency (%) |
| --- | --- | --- |
| N | 1439 | 93.7% |
| S | 97 | 6.3% |

#### Common Values (Plot)

xml version="1.0" encoding="utf-8" standalone="no"?2023-10-31T15:15:48.096592image/svg+xmlMatplotlib v3.6.0, https://matplotlib.org/

ALCOOLISMO  
Categorical

|  |  |
| --- | --- |
| Distinct | 2 |
| Distinct (%) | 0.1% |
| Missing | 0 |
| Missing (%) | 0.0% |
| Memory size | 12.1 KiB |

|  |  |
| --- | --- |
| N | 1198 |
| S | 338 |

More details

- Overview
- Categories

Unique

|  |  |
| --- | --- |
| Unique | 0 ? |
| Unique (%) | 0.0% |

Sample

|  |  |
| --- | --- |
| 1st row | N |
| 2nd row | S |
| 3rd row | N |
| 4th row | N |
| 5th row | N |

#### Common Values

| Value | Count | Frequency (%) |
| --- | --- | --- |
| N | 1198 | 78.0% |
| S | 338 | 22.0% |

#### Common Values (Plot)

xml version="1.0" encoding="utf-8" standalone="no"?2023-10-31T15:15:48.220095image/svg+xmlMatplotlib v3.6.0, https://matplotlib.org/

MENTAL  
Categorical

|  |  |
| --- | --- |
| Distinct | 2 |
| Distinct (%) | 0.1% |
| Missing | 0 |
| Missing (%) | 0.0% |
| Memory size | 12.1 KiB |

|  |  |
| --- | --- |
| N | 1511 |
| S | 25 |

More details

- Overview
- Categories

Unique

|  |  |
| --- | --- |
| Unique | 0 ? |
| Unique (%) | 0.0% |

Sample

|  |  |
| --- | --- |
| 1st row | N |
| 2nd row | N |
| 3rd row | N |
| 4th row | N |
| 5th row | N |

#### Common Values

| Value | Count | Frequency (%) |
| --- | --- | --- |
| N | 1511 | 98.4% |
| S | 25 | 1.6% |

#### Common Values (Plot)

xml version="1.0" encoding="utf-8" standalone="no"?2023-10-31T15:15:48.343957image/svg+xmlMatplotlib v3.6.0, https://matplotlib.org/

DROGADICAO  
Categorical

|  |  |
| --- | --- |
| Distinct | 2 |
| Distinct (%) | 0.1% |
| Missing | 0 |
| Missing (%) | 0.0% |
| Memory size | 12.1 KiB |

|  |  |
| --- | --- |
| N | 1241 |
| S | 295 |

More details

- Overview
- Categories

Unique

|  |  |
| --- | --- |
| Unique | 0 ? |
| Unique (%) | 0.0% |

Sample

|  |  |
| --- | --- |
| 1st row | N |
| 2nd row | N |
| 3rd row | N |
| 4th row | N |
| 5th row | S |

#### Common Values

| Value | Count | Frequency (%) |
| --- | --- | --- |
| N | 1241 | 80.8% |
| S | 295 | 19.2% |

#### Common Values (Plot)

xml version="1.0" encoding="utf-8" standalone="no"?2023-10-31T15:15:48.606416image/svg+xmlMatplotlib v3.6.0, https://matplotlib.org/

TABAGISMO  
Categorical

|  |  |
| --- | --- |
| Distinct | 2 |
| Distinct (%) | 0.1% |
| Missing | 0 |
| Missing (%) | 0.0% |
| Memory size | 12.1 KiB |

|  |  |
| --- | --- |
| N | 1231 |
| S | 305 |

More details

- Overview
- Categories

Unique

|  |  |
| --- | --- |
| Unique | 0 ? |
| Unique (%) | 0.0% |

Sample

|  |  |
| --- | --- |
| 1st row | N |
| 2nd row | S |
| 3rd row | N |
| 4th row | N |
| 5th row | N |

#### Common Values

| Value | Count | Frequency (%) |
| --- | --- | --- |
| N | 1231 | 80.1% |
| S | 305 | 19.9% |

#### Common Values (Plot)

xml version="1.0" encoding="utf-8" standalone="no"?2023-10-31T15:15:48.732538image/svg+xmlMatplotlib v3.6.0, https://matplotlib.org/

motMudEsquema  
Categorical

|  |  |
| --- | --- |
| Distinct | 3 |
| Distinct (%) | 15.0% |
| Missing | 1516 |
| Missing (%) | 98.7% |
| Memory size | 12.1 KiB |

|  |  |
| --- | --- |
| Intolerancia/Toxicidade | 13 |
| Resistencia Medicamentosa | 5 |
| Outro Motivo | 2 |

More details

- Overview
- Categories

Unique

|  |  |
| --- | --- |
| Unique | 0 ? |
| Unique (%) | 0.0% |

Sample

|  |  |
| --- | --- |
| 1st row | Resistencia Medicamentosa |
| 2nd row | Resistencia Medicamentosa |
| 3rd row | Resistencia Medicamentosa |
| 4th row | Resistencia Medicamentosa |
| 5th row | Intolerancia/Toxicidade |

#### Common Values

| Value | Count | Frequency (%) |
| --- | --- | --- |
| Intolerancia/Toxicidade | 13 | 0.8% |
| Resistencia Medicamentosa | 5 | 0.3% |
| Outro Motivo | 2 | 0.1% |
| (Missing) | 1516 | 98.7% |

#### Common Values (Plot)

xml version="1.0" encoding="utf-8" standalone="no"?2023-10-31T15:15:48.869056image/svg+xmlMatplotlib v3.6.0, https://matplotlib.org/

tipoTrat  
Categorical

|  |  |
| --- | --- |
| Distinct | 2 |
| Distinct (%) | 0.1% |
| Missing | 0 |
| Missing (%) | 0.0% |
| Memory size | 12.1 KiB |

|  |  |
| --- | --- |
| Supervisionado | 1130 |
| Auto-Administrado | 406 |

More details

- Overview
- Categories

Unique

|  |  |
| --- | --- |
| Unique | 0 ? |
| Unique (%) | 0.0% |

Sample

|  |  |
| --- | --- |
| 1st row | Supervisionado |
| 2nd row | Supervisionado |
| 3rd row | Auto-Administrado |
| 4th row | Supervisionado |
| 5th row | Supervisionado |

#### Common Values

| Value | Count | Frequency (%) |
| --- | --- | --- |
| Supervisionado | 1130 | 73.6% |
| Auto-Administrado | 406 | 26.4% |

#### Common Values (Plot)

xml version="1.0" encoding="utf-8" standalone="no"?2023-10-31T15:15:49.014518image/svg+xmlMatplotlib v3.6.0, https://matplotlib.org/

idade  
Categorical

|  |  |
| --- | --- |
| Distinct | 4 |
| Distinct (%) | 0.3% |
| Missing | 0 |
| Missing (%) | 0.0% |
| Memory size | 12.1 KiB |

|  |  |
| --- | --- |
| 40\_54 | 436 |
| 23\_39 | 420 |
| 0\_22 | 355 |
| Mais de 54 | 325 |

More details

- Overview
- Categories

Unique

|  |  |
| --- | --- |
| Unique | 0 ? |
| Unique (%) | 0.0% |

Sample

|  |  |
| --- | --- |
| 1st row | 23\_39 |
| 2nd row | 40\_54 |
| 3rd row | 40\_54 |
| 4th row | Mais de 54 |
| 5th row | 23\_39 |

#### Common Values

| Value | Count | Frequency (%) |
| --- | --- | --- |
| 40\_54 | 436 | 28.4% |
| 23\_39 | 420 | 27.3% |
| 0\_22 | 355 | 23.1% |
| Mais de 54 | 325 | 21.2% |

#### Common Values (Plot)

xml version="1.0" encoding="utf-8" standalone="no"?2023-10-31T15:15:49.161096image/svg+xmlMatplotlib v3.6.0, https://matplotlib.org/

HISTOPATOL  
Categorical

|  |  |
| --- | --- |
| Distinct | 3 |
| Distinct (%) | 0.2% |
| Missing | 0 |
| Missing (%) | 0.0% |
| Memory size | 12.1 KiB |

|  |  |
| --- | --- |
| N/realiz | 1441 |
| Sugestivo TB | 65 |
| BAAR pos | 30 |

More details

- Overview
- Categories

Unique

|  |  |
| --- | --- |
| Unique | 0 ? |
| Unique (%) | 0.0% |

Sample

|  |  |
| --- | --- |
| 1st row | N/realiz |
| 2nd row | N/realiz |
| 3rd row | BAAR pos |
| 4th row | N/realiz |
| 5th row | Sugestivo TB |

#### Common Values

| Value | Count | Frequency (%) |
| --- | --- | --- |
| N/realiz | 1441 | 93.8% |
| Sugestivo TB | 65 | 4.2% |
| BAAR pos | 30 | 2.0% |

#### Common Values (Plot)

xml version="1.0" encoding="utf-8" standalone="no"?2023-10-31T15:15:49.305419image/svg+xmlMatplotlib v3.6.0, https://matplotlib.org/

Status\_Resistencia  
Real number (ℝ)

|  |  |
| --- | --- |
| Distinct | 2 |
| Distinct (%) | 0.1% |
| Missing | 0 |
| Missing (%) | 0.0% |
| Infinite | 0 |
| Infinite (%) | 0.0% |
| Mean | 0.5 |

|  |  |
| --- | --- |
| Minimum | 0 |
| Maximum | 1 |
| Zeros | 768 |
| Zeros (%) | 50.0% |
| Negative | 0 |
| Negative (%) | 0.0% |
| Memory size | 12.1 KiB |

xml version="1.0" encoding="utf-8" standalone="no"?2023-10-31T15:15:49.414781image/svg+xmlMatplotlib v3.6.0, https://matplotlib.org/

More details

- Statistics
- Histogram
- Common values
- Extreme values

Quantile statistics

|  |  |
| --- | --- |
| Minimum | 0 |
| 5-th percentile | 0 |
| Q1 | 0 |
| median | 0.5 |
| Q3 | 1 |
| 95-th percentile | 1 |
| Maximum | 1 |
| Range | 1 |
| Interquartile range (IQR) | 1 |

Descriptive statistics

|  |  |
| --- | --- |
| Standard deviation | 0.5001628399 |
| Coefficient of variation (CV) | 1.00032568 |
| Kurtosis | -2.002609263 |
| Mean | 0.5 |
| Median Absolute Deviation (MAD) | 0.5 |
| Skewness | 0 |
| Sum | 768 |
| Variance | 0.2501628664 |
| Monotonicity | Not monotonic |

xml version="1.0" encoding="utf-8" standalone="no"?2023-10-31T15:15:49.523416image/svg+xmlMatplotlib v3.6.0, https://matplotlib.org/ 

**Histogram with fixed size bins** (bins=2)

| Value | Count | Frequency (%) |
| --- | --- | --- |
| 1 | 768 | 50.0% |
| 0 | 768 | 50.0% |

- Minimum 5 values
- Maximum 5 values

| Value | Count | Frequency (%) |
| --- | --- | --- |
| 0 | 768 | 50.0% |
| 1 | 768 | 50.0% |

| Value | Count | Frequency (%) |
| --- | --- | --- |
| 1 | 768 | 50.0% |
| 0 | 768 | 50.0% |

Report generated by YData.

 
