# Supplementary material for: Model for predicting drug resistance based on the clinical profile of tuberculosis patients using machine learning techniques
Source: PeerJ Comput Sci. 2024 Oct 14;10:e2246. doi: 10.7717/peerj-cs.2246 (PMC11623081; doi:10.7717/peerj-cs.2246)
Supplement: Supplemental Information 2 [file peerj-cs-10-2246-s002.zip › code/EDA/Tuberculosis_Clusters_Comparison.html]

Comparing Cluster 1, Cluster 2 and Cluster 3 

Toggle navigation*Comparing* Cluster 1, Cluster 2 *and* Cluster 3

- Overview
- Variables
- Correlations
- Missing values
- Sample
- Duplicate rows

# Overview

- Overview
- Alerts 89
- Reproduction

Dataset statistics

|  | Cluster 1 | Cluster 2 | Cluster 3 |
| --- | --- | --- | --- |
| Number of variables | 28 | 28 | 28 |
| Number of observations | 857 | 511 | 168 |
| Missing cells | 0 | 0 | 0 |
| Missing cells (%) | 0.0% | 0.0% | 0.0% |
| Duplicate rows | 256 | 6912 | 0 |
| Duplicate rows (%) | 29.9% | 1352.6% | 0.0% |
| Total size in memory | 183.4 KiB | 109.4 KiB | 36.1 KiB |
| Average record size in memory | 219.1 B | 219.2 B | 219.7 B |

Variable types

|  | Cluster 1 | Cluster 2 | Cluster 3 |
| --- | --- | --- | --- |
| Categorical | 28 | 28 | 28 |

Alerts

| Cluster 1 | Cluster 2 | Cluster 3 |  |
| --- | --- | --- | --- |
| `Cluster` has constant value "0" | `Cluster` has constant value "1" | `Cluster` has constant value "2" | Constant |
| Dataset has 256 (29.9%) duplicate rows | Dataset has 6912 (1352.6%) duplicate rows | *Alert not present in* | Duplicates |
| `faixaEtaria` is highly overall correlated with `idade` | `faixaEtaria` is highly overall correlated with `idade` | `faixaEtaria` is highly overall correlated with `DIABETES` and 1 other fields | High Correlation |
| `FORMACLIN1` is highly overall correlated with `classif` | `FORMACLIN1` is highly overall correlated with `classif` | `FORMACLIN1` is highly overall correlated with `classif` | High Correlation |
| `classif` is highly overall correlated with `FORMACLIN1` | `classif` is highly overall correlated with `FORMACLIN1` | `classif` is highly overall correlated with `FORMACLIN1` | High Correlation |
| `hiv` is highly overall correlated with `aids` | `hiv` is highly overall correlated with `aids` | `hiv` is highly overall correlated with `aids` | High Correlation |
| `aids` is highly overall correlated with `hiv` | `aids` is highly overall correlated with `hiv` | `aids` is highly overall correlated with `hiv` | High Correlation |
| `idade` is highly overall correlated with `faixaEtaria` | `idade` is highly overall correlated with `faixaEtaria` | `idade` is highly overall correlated with `faixaEtaria` | High Correlation |
| `tipoCaso` is highly imbalanced (82.4%) | `tipoCaso` is highly imbalanced (89.2%) | `tipoCaso` is highly imbalanced (73.9%) | Imbalance |
| `FORMACLIN1` is highly imbalanced (88.7%) | `FORMACLIN1` is highly imbalanced (72.5%) | `FORMACLIN1` is highly imbalanced (60.2%) | Imbalance |
| `classif` is highly imbalanced (74.6%) | `classif` is highly imbalanced (55.3%) | *Alert not present in* | Imbalance |
| `BACOUTRO` is highly imbalanced (76.1%) | `BACOUTRO` is highly imbalanced (73.3%) | *Alert not present in* | Imbalance |
| `cultEsc` is highly imbalanced (55.8%) | *Alert not present in* | *Alert not present in* | Imbalance |
| `NECROP` is highly imbalanced (96.6%) | `NECROP` is highly imbalanced (96.3%) | `NECROP` is highly imbalanced (94.7%) | Imbalance |
| `hiv` is highly imbalanced (54.6%) | `hiv` is highly imbalanced (68.5%) | `hiv` is highly imbalanced (59.3%) | Imbalance |
| `aids` is highly imbalanced (67.9%) | `aids` is highly imbalanced (82.9%) | *Alert not present in* | Imbalance |
| `DIABETES` is highly imbalanced (61.7%) | `DIABETES` is highly imbalanced (67.8%) | `DIABETES` is highly imbalanced (87.1%) | Imbalance |
| `MENTAL` is highly imbalanced (87.3%) | `MENTAL` is highly imbalanced (88.4%) | `MENTAL` is highly imbalanced (90.7%) | Imbalance |
| `motMudEsquema` is highly imbalanced (97.7%) | `motMudEsquema` is highly imbalanced (93.2%) | `motMudEsquema` is highly imbalanced (82.3%) | Imbalance |
| `HISTOPATOL` is highly imbalanced (84.5%) | `HISTOPATOL` is highly imbalanced (70.2%) | `HISTOPATOL` is highly imbalanced (53.7%) | Imbalance |
| *Alert not present in* | `sitAtual` is highly imbalanced (59.7%) | *Alert not present in* | Imbalance |
| *Alert not present in* | `ALCOOLISMO` is highly imbalanced (51.3%) | *Alert not present in* | Imbalance |
| *Alert not present in* | `DROGADICAO` is highly imbalanced (60.4%) | *Alert not present in* | Imbalance |
| *Alert not present in* | `TABAGISMO` is highly imbalanced (55.1%) | `TABAGISMO` is highly imbalanced (58.6%) | Imbalance |
| *Alert not present in* | `Status_Resistencia` is highly imbalanced (51.9%) | *Alert not present in* | Imbalance |
| *Alert not present in* | *Alert not present in* | `TIPOCUP` is highly overall correlated with `motMudEsquema` | High Correlation |
| *Alert not present in* | *Alert not present in* | `DIABETES` is highly overall correlated with `faixaEtaria` | High Correlation |
| *Alert not present in* | *Alert not present in* | `motMudEsquema` is highly overall correlated with `TIPOCUP` | High Correlation |

Reproduction

|  | Cluster 1 | Cluster 2 | Cluster 3 |
| --- | --- | --- | --- |
| Analysis started | 2023-08-25 16:07:23.199784 | 2023-08-25 16:07:28.703041 | 2023-08-25 16:07:33.680331 |
| Analysis finished | 2023-08-25 16:07:28.697762 | 2023-08-25 16:07:33.674186 | 2023-08-25 16:07:38.026250 |
| Duration | 5.5 seconds | 4.97 seconds | 4.35 seconds |
| Software version | pandas-profiling v3.6.6 | pandas-profiling v3.6.6 | pandas-profiling v3.6.6 |
| Download configuration | config.json | config.json | config.json |

# Variables

Select ColumnsracaCorfaixaEtariasexoESCOLARIDTIPOCUPsitAtualtipoCasoFORMACLIN1classifdescobertabacBACOUTROcultEscRXNECROPhivaidsDIABETESALCOOLISMOMENTALDROGADICAOTABAGISMOmotMudEsquematipoTratidadeHISTOPATOLStatus\_ResistenciaCluster

racaCor  
Categorical

|
|  |
|
|  |

|  | Cluster 1 | Cluster 2 | Cluster 3 |
| --- | --- | --- | --- |
| Distinct | 5 | 5 | 4 |
| Distinct (%) | 0.6% | 1.0% | 2.4% |
| Missing | 0 | 0 | 0 |
| Missing (%) | 0.0% | 0.0% | 0.0% |
| Memory size | 13.4 KiB | 8.0 KiB | 2.6 KiB |

|  |  |
| --- | --- |
| Pardo | 371 |
| Branco | 326 |
| Preto | 145 |
| Indigena | 8 |
| Amarelo | 7 |

|  |  |
| --- | --- |
| Branco | 351 |
| Pardo | 118 |
| Preto | 32 |
| Indigena | 5 |
| Amarelo | 5 |

|  |  |
| --- | --- |
| Branco | 93 |
| Pardo | 54 |
| Preto | 20 |
| Amarelo | 1 |

More details

- Overview
- Categories
- Words
- Characters

Length

|  | Cluster 1 | Cluster 2 | Cluster 3 |
| --- | --- | --- | --- |
| Max length | 8 | 8 | 7 |
| Median length | 5 | 6 | 6 |
| Mean length | 5.4247375 | 5.7358121 | 5.5654762 |
| Min length | 5 | 5 | 5 |

Characters and Unicode

|  | Cluster 1 | Cluster 2 | Cluster 3 |
| --- | --- | --- | --- |
| Total characters | 4649 | 2931 | 935 |
| Distinct characters | 16 | 16 | 13 |
| Distinct categories | 2 | 2 | 2 ? |
| Distinct scripts | 1 | 1 | 1 ? |
| Distinct blocks | 1 | 1 | 1 ? |

The Unicode Standard assigns character properties to each code point, which can be used to analyse textual variables.

Unique

|  | Cluster 1 | Cluster 2 | Cluster 3 |
| --- | --- | --- | --- |
| Unique | 0 | 0 | 1 ? |
| Unique (%) | 0.0% | 0.0% | 0.6% |

Sample

|  | Cluster 1 | Cluster 2 | Cluster 3 |
| --- | --- | --- | --- |
| 1st row | Branco | Branco | Pardo |
| 2nd row | Pardo | Branco | Branco |
| 3rd row | Branco | Branco | Branco |
| 4th row | Pardo | Branco | Branco |
| 5th row | Branco | Pardo | Preto |

#### Common Values

| Value | Count | Frequency (%) |
| --- | --- | --- |
| Pardo | 371 | 43.3% |
| Branco | 326 | 38.0% |
| Preto | 145 | 16.9% |
| Indigena | 8 | 0.9% |
| Amarelo | 7 | 0.8% |

| Value | Count | Frequency (%) |
| --- | --- | --- |
| Branco | 351 | 68.7% |
| Pardo | 118 | 23.1% |
| Preto | 32 | 6.3% |
| Indigena | 5 | 1.0% |
| Amarelo | 5 | 1.0% |

| Value | Count | Frequency (%) |
| --- | --- | --- |
| Branco | 93 | 55.4% |
| Pardo | 54 | 32.1% |
| Preto | 20 | 11.9% |
| Amarelo | 1 | 0.6% |

#### Length

xml version="1.0" encoding="utf-8" standalone="no"?2023-08-25T13:07:38.143283image/svg+xmlMatplotlib v3.6.0, https://matplotlib.org/ 

Histogram of lengths of the category

#### Common Values (Plot)

#### Cluster 1

xml version="1.0" encoding="utf-8" standalone="no"?2023-08-25T13:07:38.320772image/svg+xmlMatplotlib v3.6.0, https://matplotlib.org/

#### Cluster 2

xml version="1.0" encoding="utf-8" standalone="no"?2023-08-25T13:07:38.475564image/svg+xmlMatplotlib v3.6.0, https://matplotlib.org/

#### Cluster 3

xml version="1.0" encoding="utf-8" standalone="no"?2023-08-25T13:07:38.625988image/svg+xmlMatplotlib v3.6.0, https://matplotlib.org/

| Value | Count | Frequency (%) |
| --- | --- | --- |
| pardo | 371 | 43.3% |
| branco | 326 | 38.0% |
| preto | 145 | 16.9% |
| indigena | 8 | 0.9% |
| amarelo | 7 | 0.8% |

| Value | Count | Frequency (%) |
| --- | --- | --- |
| branco | 351 | 68.7% |
| pardo | 118 | 23.1% |
| preto | 32 | 6.3% |
| indigena | 5 | 1.0% |
| amarelo | 5 | 1.0% |

| Value | Count | Frequency (%) |
| --- | --- | --- |
| branco | 93 | 55.4% |
| pardo | 54 | 32.1% |
| preto | 20 | 11.9% |
| amarelo | 1 | 0.6% |

- Characters
- Categories
- Scripts
- Blocks

#### Most occurring characters

| Value | Count | Frequency (%) |
| --- | --- | --- |
| r | 849 | 18.3% |
| o | 849 | 18.3% |
| a | 712 | 15.3% |
| P | 516 | 11.1% |
| d | 379 | 8.2% |
| n | 342 | 7.4% |
| B | 326 | 7.0% |
| c | 326 | 7.0% |
| e | 160 | 3.4% |
| t | 145 | 3.1% |
| Other values (6) | 45 | 1.0% |

| Value | Count | Frequency (%) |
| --- | --- | --- |
| r | 506 | 17.3% |
| o | 506 | 17.3% |
| a | 479 | 16.3% |
| n | 361 | 12.3% |
| B | 351 | 12.0% |
| c | 351 | 12.0% |
| P | 150 | 5.1% |
| d | 123 | 4.2% |
| e | 42 | 1.4% |
| t | 32 | 1.1% |
| Other values (6) | 30 | 1.0% |

| Value | Count | Frequency (%) |
| --- | --- | --- |
| r | 168 | 18.0% |
| o | 168 | 18.0% |
| a | 148 | 15.8% |
| B | 93 | 9.9% |
| n | 93 | 9.9% |
| c | 93 | 9.9% |
| P | 74 | 7.9% |
| d | 54 | 5.8% |
| e | 21 | 2.2% |
| t | 20 | 2.1% |
| Other values (3) | 3 | 0.3% |

#### Most occurring categories

| Value | Count | Frequency (%) |
| --- | --- | --- |
| Lowercase Letter | 3792 | 81.6% |
| Uppercase Letter | 857 | 18.4% |

| Value | Count | Frequency (%) |
| --- | --- | --- |
| Lowercase Letter | 2420 | 82.6% |
| Uppercase Letter | 511 | 17.4% |

| Value | Count | Frequency (%) |
| --- | --- | --- |
| Lowercase Letter | 767 | 82.0% |
| Uppercase Letter | 168 | 18.0% |

#### Most frequent character per category

##### *Lowercase Letter*

| Value | Count | Frequency (%) |
| --- | --- | --- |
| r | 849 | 22.4% |
| o | 849 | 22.4% |
| a | 712 | 18.8% |
| d | 379 | 10.0% |
| n | 342 | 9.0% |
| c | 326 | 8.6% |
| e | 160 | 4.2% |
| t | 145 | 3.8% |
| i | 8 | 0.2% |
| g | 8 | 0.2% |
| Other values (2) | 14 | 0.4% |

| Value | Count | Frequency (%) |
| --- | --- | --- |
| r | 506 | 20.9% |
| o | 506 | 20.9% |
| a | 479 | 19.8% |
| n | 361 | 14.9% |
| c | 351 | 14.5% |
| d | 123 | 5.1% |
| e | 42 | 1.7% |
| t | 32 | 1.3% |
| i | 5 | 0.2% |
| g | 5 | 0.2% |
| Other values (2) | 10 | 0.4% |

| Value | Count | Frequency (%) |
| --- | --- | --- |
| r | 168 | 21.9% |
| o | 168 | 21.9% |
| a | 148 | 19.3% |
| n | 93 | 12.1% |
| c | 93 | 12.1% |
| d | 54 | 7.0% |
| e | 21 | 2.7% |
| t | 20 | 2.6% |
| m | 1 | 0.1% |
| l | 1 | 0.1% |

##### *Uppercase Letter*

| Value | Count | Frequency (%) |
| --- | --- | --- |
| P | 516 | 60.2% |
| B | 326 | 38.0% |
| I | 8 | 0.9% |
| A | 7 | 0.8% |

| Value | Count | Frequency (%) |
| --- | --- | --- |
| B | 351 | 68.7% |
| P | 150 | 29.4% |
| I | 5 | 1.0% |
| A | 5 | 1.0% |

| Value | Count | Frequency (%) |
| --- | --- | --- |
| B | 93 | 55.4% |
| P | 74 | 44.0% |
| A | 1 | 0.6% |

#### Most occurring scripts

| Value | Count | Frequency (%) |
| --- | --- | --- |
| Latin | 4649 | 100.0% |

| Value | Count | Frequency (%) |
| --- | --- | --- |
| Latin | 2931 | 100.0% |

| Value | Count | Frequency (%) |
| --- | --- | --- |
| Latin | 935 | 100.0% |

#### Most frequent character per script

##### *Latin*

| Value | Count | Frequency (%) |
| --- | --- | --- |
| r | 849 | 18.3% |
| o | 849 | 18.3% |
| a | 712 | 15.3% |
| P | 516 | 11.1% |
| d | 379 | 8.2% |
| n | 342 | 7.4% |
| B | 326 | 7.0% |
| c | 326 | 7.0% |
| e | 160 | 3.4% |
| t | 145 | 3.1% |
| Other values (6) | 45 | 1.0% |

| Value | Count | Frequency (%) |
| --- | --- | --- |
| r | 506 | 17.3% |
| o | 506 | 17.3% |
| a | 479 | 16.3% |
| n | 361 | 12.3% |
| B | 351 | 12.0% |
| c | 351 | 12.0% |
| P | 150 | 5.1% |
| d | 123 | 4.2% |
| e | 42 | 1.4% |
| t | 32 | 1.1% |
| Other values (6) | 30 | 1.0% |

| Value | Count | Frequency (%) |
| --- | --- | --- |
| r | 168 | 18.0% |
| o | 168 | 18.0% |
| a | 148 | 15.8% |
| B | 93 | 9.9% |
| n | 93 | 9.9% |
| c | 93 | 9.9% |
| P | 74 | 7.9% |
| d | 54 | 5.8% |
| e | 21 | 2.2% |
| t | 20 | 2.1% |
| Other values (3) | 3 | 0.3% |

#### Most occurring blocks

| Value | Count | Frequency (%) |
| --- | --- | --- |
| ASCII | 4649 | 100.0% |

| Value | Count | Frequency (%) |
| --- | --- | --- |
| ASCII | 2931 | 100.0% |

| Value | Count | Frequency (%) |
| --- | --- | --- |
| ASCII | 935 | 100.0% |

#### Most frequent character per block

##### *ASCII*

| Value | Count | Frequency (%) |
| --- | --- | --- |
| r | 849 | 18.3% |
| o | 849 | 18.3% |
| a | 712 | 15.3% |
| P | 516 | 11.1% |
| d | 379 | 8.2% |
| n | 342 | 7.4% |
| B | 326 | 7.0% |
| c | 326 | 7.0% |
| e | 160 | 3.4% |
| t | 145 | 3.1% |
| Other values (6) | 45 | 1.0% |

| Value | Count | Frequency (%) |
| --- | --- | --- |
| r | 506 | 17.3% |
| o | 506 | 17.3% |
| a | 479 | 16.3% |
| n | 361 | 12.3% |
| B | 351 | 12.0% |
| c | 351 | 12.0% |
| P | 150 | 5.1% |
| d | 123 | 4.2% |
| e | 42 | 1.4% |
| t | 32 | 1.1% |
| Other values (6) | 30 | 1.0% |

| Value | Count | Frequency (%) |
| --- | --- | --- |
| r | 168 | 18.0% |
| o | 168 | 18.0% |
| a | 148 | 15.8% |
| B | 93 | 9.9% |
| n | 93 | 9.9% |
| c | 93 | 9.9% |
| P | 74 | 7.9% |
| d | 54 | 5.8% |
| e | 21 | 2.2% |
| t | 20 | 2.1% |
| Other values (3) | 3 | 0.3% |

faixaEtaria  
Categorical

|
|  |
|
|  |

|  | Cluster 1 | Cluster 2 | Cluster 3 |
| --- | --- | --- | --- |
| Distinct | 10 | 12 | 9 |
| Distinct (%) | 1.2% | 2.3% | 5.4% |
| Missing | 0 | 0 | 0 |
| Missing (%) | 0.0% | 0.0% | 0.0% |
| Memory size | 13.4 KiB | 8.0 KiB | 2.6 KiB |

|  |  |
| --- | --- |
| 20\_29 | 215 |
| 30\_39 | 203 |
| 40\_49 | 185 |
| 50\_59 | 114 |
| 60\_69 | 51 |
| Other values (5) | 89 |

|  |  |
| --- | --- |
| 20\_29 | 139 |
| 30\_39 | 101 |
| 40\_49 | 95 |
| 50\_59 | 63 |
| 15\_19 | 39 |
| Other values (7) | 74 |

|  |  |
| --- | --- |
| 30\_39 | 68 |
| 40\_49 | 43 |
| 20\_29 | 33 |
| 50\_59 | 14 |
| 60\_69 | 5 |
| Other values (4) | 5 |

More details

- Overview
- Categories
- Words
- Characters

Length

|  | Cluster 1 | Cluster 2 | Cluster 3 |
| --- | --- | --- | --- |
| Max length | 16 | 16 | 16 |
| Median length | 5 | 5 | 5 |
| Mean length | 5.0641774 | 5.0821918 | 5.0654762 |
| Min length | 5 | 5 | 5 |

Characters and Unicode

|  | Cluster 1 | Cluster 2 | Cluster 3 |
| --- | --- | --- | --- |
| Total characters | 4340 | 2597 | 851 |
| Distinct characters | 21 | 21 | 21 |
| Distinct categories | 5 | 5 | 5 ? |
| Distinct scripts | 2 | 2 | 2 ? |
| Distinct blocks | 1 | 1 | 1 ? |

The Unicode Standard assigns character properties to each code point, which can be used to analyse textual variables.

Unique

|  | Cluster 1 | Cluster 2 | Cluster 3 |
| --- | --- | --- | --- |
| Unique | 0 | 1 | 3 ? |
| Unique (%) | 0.0% | 0.2% | 1.8% |

Sample

|  | Cluster 1 | Cluster 2 | Cluster 3 |
| --- | --- | --- | --- |
| 1st row | 20\_29 | 40\_49 | 40\_49 |
| 2nd row | 40\_49 | 40\_49 | 30\_39 |
| 3rd row | 50\_59 | 40\_49 | 20\_29 |
| 4th row | 20\_29 | 50\_59 | 20\_29 |
| 5th row | 30\_39 | 10\_14 | 30\_39 |

#### Common Values

| Value | Count | Frequency (%) |
| --- | --- | --- |
| 20\_29 | 215 | 25.1% |
| 30\_39 | 203 | 23.7% |
| 40\_49 | 185 | 21.6% |
| 50\_59 | 114 | 13.3% |
| 60\_69 | 51 | 6.0% |
| 15\_19 | 48 | 5.6% |
| 70\_79 | 21 | 2.5% |
| 10\_14 | 11 | 1.3% |
| Maior de 80 anos | 5 | 0.6% |
| 05\_09 | 4 | 0.5% |

| Value | Count | Frequency (%) |
| --- | --- | --- |
| 20\_29 | 139 | 27.2% |
| 30\_39 | 101 | 19.8% |
| 40\_49 | 95 | 18.6% |
| 50\_59 | 63 | 12.3% |
| 15\_19 | 39 | 7.6% |
| 60\_69 | 34 | 6.7% |
| 70\_79 | 12 | 2.3% |
| 10\_14 | 11 | 2.2% |
| 01\_04 | 7 | 1.4% |
| 05\_09 | 6 | 1.2% |
| Other values (2) | 4 | 0.8% |

| Value | Count | Frequency (%) |
| --- | --- | --- |
| 30\_39 | 68 | 40.5% |
| 40\_49 | 43 | 25.6% |
| 20\_29 | 33 | 19.6% |
| 50\_59 | 14 | 8.3% |
| 60\_69 | 5 | 3.0% |
| 70\_79 | 2 | 1.2% |
| 05\_09 | 1 | 0.6% |
| Maior de 80 anos | 1 | 0.6% |
| 01\_04 | 1 | 0.6% |

#### Length

xml version="1.0" encoding="utf-8" standalone="no"?2023-08-25T13:07:38.761399image/svg+xmlMatplotlib v3.6.0, https://matplotlib.org/ 

Histogram of lengths of the category

#### Common Values (Plot)

#### Cluster 1

xml version="1.0" encoding="utf-8" standalone="no"?2023-08-25T13:07:38.947247image/svg+xmlMatplotlib v3.6.0, https://matplotlib.org/

#### Cluster 2

  
*Number of variable categories passes threshold (`config.plot.cat_freq.max_unique`)*

#### Cluster 3

xml version="1.0" encoding="utf-8" standalone="no"?2023-08-25T13:07:39.164224image/svg+xmlMatplotlib v3.6.0, https://matplotlib.org/

| Value | Count | Frequency (%) |
| --- | --- | --- |
| 20\_29 | 215 | 24.7% |
| 30\_39 | 203 | 23.3% |
| 40\_49 | 185 | 21.2% |
| 50\_59 | 114 | 13.1% |
| 60\_69 | 51 | 5.8% |
| 15\_19 | 48 | 5.5% |
| 70\_79 | 21 | 2.4% |
| 10\_14 | 11 | 1.3% |
| maior | 5 | 0.6% |
| de | 5 | 0.6% |
| Other values (3) | 14 | 1.6% |

| Value | Count | Frequency (%) |
| --- | --- | --- |
| 20\_29 | 139 | 26.6% |
| 30\_39 | 101 | 19.3% |
| 40\_49 | 95 | 18.2% |
| 50\_59 | 63 | 12.0% |
| 15\_19 | 39 | 7.5% |
| 60\_69 | 34 | 6.5% |
| 70\_79 | 12 | 2.3% |
| 10\_14 | 11 | 2.1% |
| 01\_04 | 7 | 1.3% |
| 05\_09 | 6 | 1.1% |
| Other values (7) | 16 | 3.1% |

| Value | Count | Frequency (%) |
| --- | --- | --- |
| 30\_39 | 68 | 39.8% |
| 40\_49 | 43 | 25.1% |
| 20\_29 | 33 | 19.3% |
| 50\_59 | 14 | 8.2% |
| 60\_69 | 5 | 2.9% |
| 70\_79 | 2 | 1.2% |
| 05\_09 | 1 | 0.6% |
| maior | 1 | 0.6% |
| de | 1 | 0.6% |
| 80 | 1 | 0.6% |
| Other values (2) | 2 | 1.2% |

- Characters
- Categories
- Scripts
- Blocks

#### Most occurring characters

| Value | Count | Frequency (%) |
| --- | --- | --- |
| \_ | 852 | 19.6% |
| 9 | 841 | 19.4% |
| 0 | 813 | 18.7% |
| 2 | 430 | 9.9% |
| 3 | 406 | 9.4% |
| 4 | 381 | 8.8% |
| 5 | 280 | 6.5% |
| 1 | 118 | 2.7% |
| 6 | 102 | 2.4% |
| 7 | 42 | 1.0% |
| Other values (11) | 75 | 1.7% |

| Value | Count | Frequency (%) |
| --- | --- | --- |
| \_ | 507 | 19.5% |
| 9 | 489 | 18.8% |
| 0 | 484 | 18.6% |
| 2 | 278 | 10.7% |
| 4 | 208 | 8.0% |
| 3 | 202 | 7.8% |
| 5 | 171 | 6.6% |
| 1 | 108 | 4.2% |
| 6 | 68 | 2.6% |
| 7 | 24 | 0.9% |
| Other values (11) | 58 | 2.2% |

| Value | Count | Frequency (%) |
| --- | --- | --- |
| 0 | 170 | 20.0% |
| \_ | 167 | 19.6% |
| 9 | 166 | 19.5% |
| 3 | 136 | 16.0% |
| 4 | 87 | 10.2% |
| 2 | 66 | 7.8% |
| 5 | 29 | 3.4% |
| 6 | 10 | 1.2% |
| 7 | 4 | 0.5% |
|  | 3 | 0.4% |
| Other values (11) | 13 | 1.5% |

#### Most occurring categories

| Value | Count | Frequency (%) |
| --- | --- | --- |
| Decimal Number | 3418 | 78.8% |
| Connector Punctuation | 852 | 19.6% |
| Lowercase Letter | 50 | 1.2% |
| Space Separator | 15 | 0.3% |
| Uppercase Letter | 5 | 0.1% |

| Value | Count | Frequency (%) |
| --- | --- | --- |
| Decimal Number | 2035 | 78.4% |
| Connector Punctuation | 507 | 19.5% |
| Lowercase Letter | 39 | 1.5% |
| Space Separator | 12 | 0.5% |
| Uppercase Letter | 4 | 0.2% |

| Value | Count | Frequency (%) |
| --- | --- | --- |
| Decimal Number | 670 | 78.7% |
| Connector Punctuation | 167 | 19.6% |
| Lowercase Letter | 10 | 1.2% |
| Space Separator | 3 | 0.4% |
| Uppercase Letter | 1 | 0.1% |

#### Most frequent character per category

##### *Connector Punctuation*

| Value | Count | Frequency (%) |
| --- | --- | --- |
| \_ | 852 | 100.0% |

| Value | Count | Frequency (%) |
| --- | --- | --- |
| \_ | 507 | 100.0% |

| Value | Count | Frequency (%) |
| --- | --- | --- |
| \_ | 167 | 100.0% |

##### *Decimal Number*

| Value | Count | Frequency (%) |
| --- | --- | --- |
| 9 | 841 | 24.6% |
| 0 | 813 | 23.8% |
| 2 | 430 | 12.6% |
| 3 | 406 | 11.9% |
| 4 | 381 | 11.1% |
| 5 | 280 | 8.2% |
| 1 | 118 | 3.5% |
| 6 | 102 | 3.0% |
| 7 | 42 | 1.2% |
| 8 | 5 | 0.1% |

| Value | Count | Frequency (%) |
| --- | --- | --- |
| 9 | 489 | 24.0% |
| 0 | 484 | 23.8% |
| 2 | 278 | 13.7% |
| 4 | 208 | 10.2% |
| 3 | 202 | 9.9% |
| 5 | 171 | 8.4% |
| 1 | 108 | 5.3% |
| 6 | 68 | 3.3% |
| 7 | 24 | 1.2% |
| 8 | 3 | 0.1% |

| Value | Count | Frequency (%) |
| --- | --- | --- |
| 0 | 170 | 25.4% |
| 9 | 166 | 24.8% |
| 3 | 136 | 20.3% |
| 4 | 87 | 13.0% |
| 2 | 66 | 9.9% |
| 5 | 29 | 4.3% |
| 6 | 10 | 1.5% |
| 7 | 4 | 0.6% |
| 8 | 1 | 0.1% |
| 1 | 1 | 0.1% |

##### *Space Separator*

| Value | Count | Frequency (%) |
| --- | --- | --- |
|  | 15 | 100.0% |

| Value | Count | Frequency (%) |
| --- | --- | --- |
|  | 12 | 100.0% |

| Value | Count | Frequency (%) |
| --- | --- | --- |
|  | 3 | 100.0% |

##### *Lowercase Letter*

| Value | Count | Frequency (%) |
| --- | --- | --- |
| a | 10 | 20.0% |
| o | 10 | 20.0% |
| e | 5 | 10.0% |
| n | 5 | 10.0% |
| d | 5 | 10.0% |
| r | 5 | 10.0% |
| i | 5 | 10.0% |
| s | 5 | 10.0% |

| Value | Count | Frequency (%) |
| --- | --- | --- |
| o | 8 | 20.5% |
| a | 7 | 17.9% |
| n | 5 | 12.8% |
| e | 5 | 12.8% |
| d | 4 | 10.3% |
| r | 4 | 10.3% |
| i | 3 | 7.7% |
| s | 3 | 7.7% |

| Value | Count | Frequency (%) |
| --- | --- | --- |
| o | 2 | 20.0% |
| a | 2 | 20.0% |
| i | 1 | 10.0% |
| r | 1 | 10.0% |
| d | 1 | 10.0% |
| e | 1 | 10.0% |
| n | 1 | 10.0% |
| s | 1 | 10.0% |

##### *Uppercase Letter*

| Value | Count | Frequency (%) |
| --- | --- | --- |
| M | 5 | 100.0% |

| Value | Count | Frequency (%) |
| --- | --- | --- |
| M | 4 | 100.0% |

| Value | Count | Frequency (%) |
| --- | --- | --- |
| M | 1 | 100.0% |

#### Most occurring scripts

| Value | Count | Frequency (%) |
| --- | --- | --- |
| Common | 4285 | 98.7% |
| Latin | 55 | 1.3% |

| Value | Count | Frequency (%) |
| --- | --- | --- |
| Common | 2554 | 98.3% |
| Latin | 43 | 1.7% |

| Value | Count | Frequency (%) |
| --- | --- | --- |
| Common | 840 | 98.7% |
| Latin | 11 | 1.3% |

#### Most frequent character per script

##### *Common*

| Value | Count | Frequency (%) |
| --- | --- | --- |
| \_ | 852 | 19.9% |
| 9 | 841 | 19.6% |
| 0 | 813 | 19.0% |
| 2 | 430 | 10.0% |
| 3 | 406 | 9.5% |
| 4 | 381 | 8.9% |
| 5 | 280 | 6.5% |
| 1 | 118 | 2.8% |
| 6 | 102 | 2.4% |
| 7 | 42 | 1.0% |
| Other values (2) | 20 | 0.5% |

| Value | Count | Frequency (%) |
| --- | --- | --- |
| \_ | 507 | 19.9% |
| 9 | 489 | 19.1% |
| 0 | 484 | 19.0% |
| 2 | 278 | 10.9% |
| 4 | 208 | 8.1% |
| 3 | 202 | 7.9% |
| 5 | 171 | 6.7% |
| 1 | 108 | 4.2% |
| 6 | 68 | 2.7% |
| 7 | 24 | 0.9% |
| Other values (2) | 15 | 0.6% |

| Value | Count | Frequency (%) |
| --- | --- | --- |
| 0 | 170 | 20.2% |
| \_ | 167 | 19.9% |
| 9 | 166 | 19.8% |
| 3 | 136 | 16.2% |
| 4 | 87 | 10.4% |
| 2 | 66 | 7.9% |
| 5 | 29 | 3.5% |
| 6 | 10 | 1.2% |
| 7 | 4 | 0.5% |
|  | 3 | 0.4% |
| Other values (2) | 2 | 0.2% |

##### *Latin*

| Value | Count | Frequency (%) |
| --- | --- | --- |
| a | 10 | 18.2% |
| o | 10 | 18.2% |
| e | 5 | 9.1% |
| n | 5 | 9.1% |
| M | 5 | 9.1% |
| d | 5 | 9.1% |
| r | 5 | 9.1% |
| i | 5 | 9.1% |
| s | 5 | 9.1% |

| Value | Count | Frequency (%) |
| --- | --- | --- |
| o | 8 | 18.6% |
| a | 7 | 16.3% |
| n | 5 | 11.6% |
| e | 5 | 11.6% |
| d | 4 | 9.3% |
| M | 4 | 9.3% |
| r | 4 | 9.3% |
| i | 3 | 7.0% |
| s | 3 | 7.0% |

| Value | Count | Frequency (%) |
| --- | --- | --- |
| o | 2 | 18.2% |
| a | 2 | 18.2% |
| i | 1 | 9.1% |
| r | 1 | 9.1% |
| M | 1 | 9.1% |
| d | 1 | 9.1% |
| e | 1 | 9.1% |
| n | 1 | 9.1% |
| s | 1 | 9.1% |

#### Most occurring blocks

| Value | Count | Frequency (%) |
| --- | --- | --- |
| ASCII | 4340 | 100.0% |

| Value | Count | Frequency (%) |
| --- | --- | --- |
| ASCII | 2597 | 100.0% |

| Value | Count | Frequency (%) |
| --- | --- | --- |
| ASCII | 851 | 100.0% |

#### Most frequent character per block

##### *ASCII*

| Value | Count | Frequency (%) |
| --- | --- | --- |
| \_ | 852 | 19.6% |
| 9 | 841 | 19.4% |
| 0 | 813 | 18.7% |
| 2 | 430 | 9.9% |
| 3 | 406 | 9.4% |
| 4 | 381 | 8.8% |
| 5 | 280 | 6.5% |
| 1 | 118 | 2.7% |
| 6 | 102 | 2.4% |
| 7 | 42 | 1.0% |
| Other values (11) | 75 | 1.7% |

| Value | Count | Frequency (%) |
| --- | --- | --- |
| \_ | 507 | 19.5% |
| 9 | 489 | 18.8% |
| 0 | 484 | 18.6% |
| 2 | 278 | 10.7% |
| 4 | 208 | 8.0% |
| 3 | 202 | 7.8% |
| 5 | 171 | 6.6% |
| 1 | 108 | 4.2% |
| 6 | 68 | 2.6% |
| 7 | 24 | 0.9% |
| Other values (11) | 58 | 2.2% |

| Value | Count | Frequency (%) |
| --- | --- | --- |
| 0 | 170 | 20.0% |
| \_ | 167 | 19.6% |
| 9 | 166 | 19.5% |
| 3 | 136 | 16.0% |
| 4 | 87 | 10.2% |
| 2 | 66 | 7.8% |
| 5 | 29 | 3.4% |
| 6 | 10 | 1.2% |
| 7 | 4 | 0.5% |
|  | 3 | 0.4% |
| Other values (11) | 13 | 1.5% |

sexo  
Categorical

|
|  |
|
|  |

|  | Cluster 1 | Cluster 2 | Cluster 3 |
| --- | --- | --- | --- |
| Distinct | 2 | 2 | 2 |
| Distinct (%) | 0.2% | 0.4% | 1.2% |
| Missing | 0 | 0 | 0 |
| Missing (%) | 0.0% | 0.0% | 0.0% |
| Memory size | 7.7 KiB | 4.6 KiB | 1.6 KiB |

|  |  |
| --- | --- |
| M | 720 |
| F | 137 |

|  |  |
| --- | --- |
| F | 293 |
| M | 218 |

|  |  |
| --- | --- |
| M | 127 |
| F | 41 |

More details

- Overview
- Categories
- Words
- Characters

Length

|  | Cluster 1 | Cluster 2 | Cluster 3 |
| --- | --- | --- | --- |
| Max length | 1 | 1 | 1 |
| Median length | 1 | 1 | 1 |
| Mean length | 1 | 1 | 1 |
| Min length | 1 | 1 | 1 |

Characters and Unicode

|  | Cluster 1 | Cluster 2 | Cluster 3 |
| --- | --- | --- | --- |
| Total characters | 857 | 511 | 168 |
| Distinct characters | 2 | 2 | 2 |
| Distinct categories | 1 | 1 | 1 ? |
| Distinct scripts | 1 | 1 | 1 ? |
| Distinct blocks | 1 | 1 | 1 ? |

The Unicode Standard assigns character properties to each code point, which can be used to analyse textual variables.

Unique

|  | Cluster 1 | Cluster 2 | Cluster 3 |
| --- | --- | --- | --- |
| Unique | 0 | 0 | 0 ? |
| Unique (%) | 0.0% | 0.0% | 0.0% |

Sample

|  | Cluster 1 | Cluster 2 | Cluster 3 |
| --- | --- | --- | --- |
| 1st row | M | F | M |
| 2nd row | M | M | M |
| 3rd row | M | F | M |
| 4th row | F | F | M |
| 5th row | M | F | M |

#### Common Values

| Value | Count | Frequency (%) |
| --- | --- | --- |
| M | 720 | 84.0% |
| F | 137 | 16.0% |

| Value | Count | Frequency (%) |
| --- | --- | --- |
| F | 293 | 57.3% |
| M | 218 | 42.7% |

| Value | Count | Frequency (%) |
| --- | --- | --- |
| M | 127 | 75.6% |
| F | 41 | 24.4% |

#### Length

xml version="1.0" encoding="utf-8" standalone="no"?2023-08-25T13:07:39.326038image/svg+xmlMatplotlib v3.6.0, https://matplotlib.org/ 

Histogram of lengths of the category

#### Common Values (Plot)

#### Cluster 1

xml version="1.0" encoding="utf-8" standalone="no"?2023-08-25T13:07:39.471265image/svg+xmlMatplotlib v3.6.0, https://matplotlib.org/

#### Cluster 2

xml version="1.0" encoding="utf-8" standalone="no"?2023-08-25T13:07:39.619725image/svg+xmlMatplotlib v3.6.0, https://matplotlib.org/

#### Cluster 3

xml version="1.0" encoding="utf-8" standalone="no"?2023-08-25T13:07:39.750308image/svg+xmlMatplotlib v3.6.0, https://matplotlib.org/

| Value | Count | Frequency (%) |
| --- | --- | --- |
| m | 720 | 84.0% |
| f | 137 | 16.0% |

| Value | Count | Frequency (%) |
| --- | --- | --- |
| f | 293 | 57.3% |
| m | 218 | 42.7% |

| Value | Count | Frequency (%) |
| --- | --- | --- |
| m | 127 | 75.6% |
| f | 41 | 24.4% |

- Characters
- Categories
- Scripts
- Blocks

#### Most occurring characters

| Value | Count | Frequency (%) |
| --- | --- | --- |
| M | 720 | 84.0% |
| F | 137 | 16.0% |

| Value | Count | Frequency (%) |
| --- | --- | --- |
| F | 293 | 57.3% |
| M | 218 | 42.7% |

| Value | Count | Frequency (%) |
| --- | --- | --- |
| M | 127 | 75.6% |
| F | 41 | 24.4% |

#### Most occurring categories

| Value | Count | Frequency (%) |
| --- | --- | --- |
| Uppercase Letter | 857 | 100.0% |

| Value | Count | Frequency (%) |
| --- | --- | --- |
| Uppercase Letter | 511 | 100.0% |

| Value | Count | Frequency (%) |
| --- | --- | --- |
| Uppercase Letter | 168 | 100.0% |

#### Most frequent character per category

##### *Uppercase Letter*

| Value | Count | Frequency (%) |
| --- | --- | --- |
| M | 720 | 84.0% |
| F | 137 | 16.0% |

| Value | Count | Frequency (%) |
| --- | --- | --- |
| F | 293 | 57.3% |
| M | 218 | 42.7% |

| Value | Count | Frequency (%) |
| --- | --- | --- |
| M | 127 | 75.6% |
| F | 41 | 24.4% |

#### Most occurring scripts

| Value | Count | Frequency (%) |
| --- | --- | --- |
| Latin | 857 | 100.0% |

| Value | Count | Frequency (%) |
| --- | --- | --- |
| Latin | 511 | 100.0% |

| Value | Count | Frequency (%) |
| --- | --- | --- |
| Latin | 168 | 100.0% |

#### Most frequent character per script

##### *Latin*

| Value | Count | Frequency (%) |
| --- | --- | --- |
| M | 720 | 84.0% |
| F | 137 | 16.0% |

| Value | Count | Frequency (%) |
| --- | --- | --- |
| F | 293 | 57.3% |
| M | 218 | 42.7% |

| Value | Count | Frequency (%) |
| --- | --- | --- |
| M | 127 | 75.6% |
| F | 41 | 24.4% |

#### Most occurring blocks

| Value | Count | Frequency (%) |
| --- | --- | --- |
| ASCII | 857 | 100.0% |

| Value | Count | Frequency (%) |
| --- | --- | --- |
| ASCII | 511 | 100.0% |

| Value | Count | Frequency (%) |
| --- | --- | --- |
| ASCII | 168 | 100.0% |

#### Most frequent character per block

##### *ASCII*

| Value | Count | Frequency (%) |
| --- | --- | --- |
| M | 720 | 84.0% |
| F | 137 | 16.0% |

| Value | Count | Frequency (%) |
| --- | --- | --- |
| F | 293 | 57.3% |
| M | 218 | 42.7% |

| Value | Count | Frequency (%) |
| --- | --- | --- |
| M | 127 | 75.6% |
| F | 41 | 24.4% |

ESCOLARID  
Categorical

|
|  |
|
|  |

|  | Cluster 1 | Cluster 2 | Cluster 3 |
| --- | --- | --- | --- |
| Distinct | 6 | 6 | 6 |
| Distinct (%) | 0.7% | 1.2% | 3.6% |
| Missing | 0 | 0 | 0 |
| Missing (%) | 0.0% | 0.0% | 0.0% |
| Memory size | 13.4 KiB | 8.0 KiB | 2.6 KiB |

|  |  |
| --- | --- |
| De 4 a 7 anos | 322 |
| De 8 a 11 anos | 316 |
| De 1 a 3 anos | 116 |
| De 12 a 14 anos | 52 |
| Nenhuma | 35 |

|  |  |
| --- | --- |
| De 8 a 11 anos | 198 |
| De 4 a 7 anos | 160 |
| De 1 a 3 anos | 56 |
| De 12 a 14 anos | 50 |
| 15 anos e mais | 28 |

|  |  |
| --- | --- |
| De 8 a 11 anos | 81 |
| De 4 a 7 anos | 42 |
| De 12 a 14 anos | 17 |
| De 1 a 3 anos | 13 |
| 15 anos e mais | 9 |

More details

- Overview
- Categories
- Words
- Characters

Length

|  | Cluster 1 | Cluster 2 | Cluster 3 |
| --- | --- | --- | --- |
| Max length | 15 | 15 | 15 |
| Median length | 13 | 14 | 14 |
| Mean length | 13.263711 | 13.414873 | 13.52381 |
| Min length | 7 | 7 | 7 |

Characters and Unicode

|  | Cluster 1 | Cluster 2 | Cluster 3 |
| --- | --- | --- | --- |
| Total characters | 11367 | 6855 | 2272 |
| Distinct characters | 19 | 19 | 19 |
| Distinct categories | 4 | 4 | 4 ? |
| Distinct scripts | 2 | 2 | 2 ? |
| Distinct blocks | 1 | 1 | 1 ? |

The Unicode Standard assigns character properties to each code point, which can be used to analyse textual variables.

Unique

|  | Cluster 1 | Cluster 2 | Cluster 3 |
| --- | --- | --- | --- |
| Unique | 0 | 0 | 0 ? |
| Unique (%) | 0.0% | 0.0% | 0.0% |

Sample

|  | Cluster 1 | Cluster 2 | Cluster 3 |
| --- | --- | --- | --- |
| 1st row | De 4 a 7 anos | 15 anos e mais | De 8 a 11 anos |
| 2nd row | De 4 a 7 anos | De 1 a 3 anos | De 4 a 7 anos |
| 3rd row | De 4 a 7 anos | De 8 a 11 anos | De 8 a 11 anos |
| 4th row | De 8 a 11 anos | De 4 a 7 anos | De 1 a 3 anos |
| 5th row | De 8 a 11 anos | De 4 a 7 anos | De 8 a 11 anos |

#### Common Values

| Value | Count | Frequency (%) |
| --- | --- | --- |
| De 4 a 7 anos | 322 | 37.6% |
| De 8 a 11 anos | 316 | 36.9% |
| De 1 a 3 anos | 116 | 13.5% |
| De 12 a 14 anos | 52 | 6.1% |
| Nenhuma | 35 | 4.1% |
| 15 anos e mais | 16 | 1.9% |

| Value | Count | Frequency (%) |
| --- | --- | --- |
| De 8 a 11 anos | 198 | 38.7% |
| De 4 a 7 anos | 160 | 31.3% |
| De 1 a 3 anos | 56 | 11.0% |
| De 12 a 14 anos | 50 | 9.8% |
| 15 anos e mais | 28 | 5.5% |
| Nenhuma | 19 | 3.7% |

| Value | Count | Frequency (%) |
| --- | --- | --- |
| De 8 a 11 anos | 81 | 48.2% |
| De 4 a 7 anos | 42 | 25.0% |
| De 12 a 14 anos | 17 | 10.1% |
| De 1 a 3 anos | 13 | 7.7% |
| 15 anos e mais | 9 | 5.4% |
| Nenhuma | 6 | 3.6% |

#### Length

xml version="1.0" encoding="utf-8" standalone="no"?2023-08-25T13:07:39.885225image/svg+xmlMatplotlib v3.6.0, https://matplotlib.org/ 

Histogram of lengths of the category

#### Common Values (Plot)

#### Cluster 1

xml version="1.0" encoding="utf-8" standalone="no"?2023-08-25T13:07:40.072222image/svg+xmlMatplotlib v3.6.0, https://matplotlib.org/

#### Cluster 2

xml version="1.0" encoding="utf-8" standalone="no"?2023-08-25T13:07:40.245993image/svg+xmlMatplotlib v3.6.0, https://matplotlib.org/

#### Cluster 3

xml version="1.0" encoding="utf-8" standalone="no"?2023-08-25T13:07:40.422354image/svg+xmlMatplotlib v3.6.0, https://matplotlib.org/

| Value | Count | Frequency (%) |
| --- | --- | --- |
| anos | 822 | 19.9% |
| de | 806 | 19.5% |
| a | 806 | 19.5% |
| 4 | 322 | 7.8% |
| 7 | 322 | 7.8% |
| 8 | 316 | 7.7% |
| 11 | 316 | 7.7% |
| 1 | 116 | 2.8% |
| 3 | 116 | 2.8% |
| 12 | 52 | 1.3% |
| Other values (5) | 135 | 3.3% |

| Value | Count | Frequency (%) |
| --- | --- | --- |
| anos | 492 | 20.1% |
| de | 464 | 18.9% |
| a | 464 | 18.9% |
| 8 | 198 | 8.1% |
| 11 | 198 | 8.1% |
| 4 | 160 | 6.5% |
| 7 | 160 | 6.5% |
| 1 | 56 | 2.3% |
| 3 | 56 | 2.3% |
| 12 | 50 | 2.0% |
| Other values (5) | 153 | 6.2% |

| Value | Count | Frequency (%) |
| --- | --- | --- |
| anos | 162 | 20.1% |
| de | 153 | 19.0% |
| a | 153 | 19.0% |
| 8 | 81 | 10.0% |
| 11 | 81 | 10.0% |
| 4 | 42 | 5.2% |
| 7 | 42 | 5.2% |
| 12 | 17 | 2.1% |
| 14 | 17 | 2.1% |
| 1 | 13 | 1.6% |
| Other values (5) | 46 | 5.7% |

- Characters
- Categories
- Scripts
- Blocks

#### Most occurring characters

| Value | Count | Frequency (%) |
| --- | --- | --- |
|  | 3272 | 28.8% |
| a | 1679 | 14.8% |
| 1 | 868 | 7.6% |
| n | 857 | 7.5% |
| e | 857 | 7.5% |
| s | 838 | 7.4% |
| o | 822 | 7.2% |
| D | 806 | 7.1% |
| 4 | 374 | 3.3% |
| 7 | 322 | 2.8% |
| Other values (9) | 672 | 5.9% |

| Value | Count | Frequency (%) |
| --- | --- | --- |
|  | 1940 | 28.3% |
| a | 1003 | 14.6% |
| 1 | 580 | 8.5% |
| s | 520 | 7.6% |
| e | 511 | 7.5% |
| n | 511 | 7.5% |
| o | 492 | 7.2% |
| D | 464 | 6.8% |
| 4 | 210 | 3.1% |
| 8 | 198 | 2.9% |
| Other values (9) | 426 | 6.2% |

| Value | Count | Frequency (%) |
| --- | --- | --- |
|  | 639 | 28.1% |
| a | 330 | 14.5% |
| 1 | 218 | 9.6% |
| s | 171 | 7.5% |
| e | 168 | 7.4% |
| n | 168 | 7.4% |
| o | 162 | 7.1% |
| D | 153 | 6.7% |
| 8 | 81 | 3.6% |
| 4 | 59 | 2.6% |
| Other values (9) | 123 | 5.4% |

#### Most occurring categories

| Value | Count | Frequency (%) |
| --- | --- | --- |
| Lowercase Letter | 5190 | 45.7% |
| Space Separator | 3272 | 28.8% |
| Decimal Number | 2064 | 18.2% |
| Uppercase Letter | 841 | 7.4% |

| Value | Count | Frequency (%) |
| --- | --- | --- |
| Lowercase Letter | 3150 | 46.0% |
| Space Separator | 1940 | 28.3% |
| Decimal Number | 1282 | 18.7% |
| Uppercase Letter | 483 | 7.0% |

| Value | Count | Frequency (%) |
| --- | --- | --- |
| Lowercase Letter | 1035 | 45.6% |
| Space Separator | 639 | 28.1% |
| Decimal Number | 439 | 19.3% |
| Uppercase Letter | 159 | 7.0% |

#### Most frequent character per category

##### *Space Separator*

| Value | Count | Frequency (%) |
| --- | --- | --- |
|  | 3272 | 100.0% |

| Value | Count | Frequency (%) |
| --- | --- | --- |
|  | 1940 | 100.0% |

| Value | Count | Frequency (%) |
| --- | --- | --- |
|  | 639 | 100.0% |

##### *Lowercase Letter*

| Value | Count | Frequency (%) |
| --- | --- | --- |
| a | 1679 | 32.4% |
| n | 857 | 16.5% |
| e | 857 | 16.5% |
| s | 838 | 16.1% |
| o | 822 | 15.8% |
| m | 51 | 1.0% |
| h | 35 | 0.7% |
| u | 35 | 0.7% |
| i | 16 | 0.3% |

| Value | Count | Frequency (%) |
| --- | --- | --- |
| a | 1003 | 31.8% |
| s | 520 | 16.5% |
| e | 511 | 16.2% |
| n | 511 | 16.2% |
| o | 492 | 15.6% |
| m | 47 | 1.5% |
| i | 28 | 0.9% |
| h | 19 | 0.6% |
| u | 19 | 0.6% |

| Value | Count | Frequency (%) |
| --- | --- | --- |
| a | 330 | 31.9% |
| s | 171 | 16.5% |
| e | 168 | 16.2% |
| n | 168 | 16.2% |
| o | 162 | 15.7% |
| m | 15 | 1.4% |
| i | 9 | 0.9% |
| h | 6 | 0.6% |
| u | 6 | 0.6% |

##### *Decimal Number*

| Value | Count | Frequency (%) |
| --- | --- | --- |
| 1 | 868 | 42.1% |
| 4 | 374 | 18.1% |
| 7 | 322 | 15.6% |
| 8 | 316 | 15.3% |
| 3 | 116 | 5.6% |
| 2 | 52 | 2.5% |
| 5 | 16 | 0.8% |

| Value | Count | Frequency (%) |
| --- | --- | --- |
| 1 | 580 | 45.2% |
| 4 | 210 | 16.4% |
| 8 | 198 | 15.4% |
| 7 | 160 | 12.5% |
| 3 | 56 | 4.4% |
| 2 | 50 | 3.9% |
| 5 | 28 | 2.2% |

| Value | Count | Frequency (%) |
| --- | --- | --- |
| 1 | 218 | 49.7% |
| 8 | 81 | 18.5% |
| 4 | 59 | 13.4% |
| 7 | 42 | 9.6% |
| 2 | 17 | 3.9% |
| 3 | 13 | 3.0% |
| 5 | 9 | 2.1% |

##### *Uppercase Letter*

| Value | Count | Frequency (%) |
| --- | --- | --- |
| D | 806 | 95.8% |
| N | 35 | 4.2% |

| Value | Count | Frequency (%) |
| --- | --- | --- |
| D | 464 | 96.1% |
| N | 19 | 3.9% |

| Value | Count | Frequency (%) |
| --- | --- | --- |
| D | 153 | 96.2% |
| N | 6 | 3.8% |

#### Most occurring scripts

| Value | Count | Frequency (%) |
| --- | --- | --- |
| Latin | 6031 | 53.1% |
| Common | 5336 | 46.9% |

| Value | Count | Frequency (%) |
| --- | --- | --- |
| Latin | 3633 | 53.0% |
| Common | 3222 | 47.0% |

| Value | Count | Frequency (%) |
| --- | --- | --- |
| Latin | 1194 | 52.6% |
| Common | 1078 | 47.4% |

#### Most frequent character per script

##### *Common*

| Value | Count | Frequency (%) |
| --- | --- | --- |
|  | 3272 | 61.3% |
| 1 | 868 | 16.3% |
| 4 | 374 | 7.0% |
| 7 | 322 | 6.0% |
| 8 | 316 | 5.9% |
| 3 | 116 | 2.2% |
| 2 | 52 | 1.0% |
| 5 | 16 | 0.3% |

| Value | Count | Frequency (%) |
| --- | --- | --- |
|  | 1940 | 60.2% |
| 1 | 580 | 18.0% |
| 4 | 210 | 6.5% |
| 8 | 198 | 6.1% |
| 7 | 160 | 5.0% |
| 3 | 56 | 1.7% |
| 2 | 50 | 1.6% |
| 5 | 28 | 0.9% |

| Value | Count | Frequency (%) |
| --- | --- | --- |
|  | 639 | 59.3% |
| 1 | 218 | 20.2% |
| 8 | 81 | 7.5% |
| 4 | 59 | 5.5% |
| 7 | 42 | 3.9% |
| 2 | 17 | 1.6% |
| 3 | 13 | 1.2% |
| 5 | 9 | 0.8% |

##### *Latin*

| Value | Count | Frequency (%) |
| --- | --- | --- |
| a | 1679 | 27.8% |
| n | 857 | 14.2% |
| e | 857 | 14.2% |
| s | 838 | 13.9% |
| o | 822 | 13.6% |
| D | 806 | 13.4% |
| m | 51 | 0.8% |
| N | 35 | 0.6% |
| h | 35 | 0.6% |
| u | 35 | 0.6% |

| Value | Count | Frequency (%) |
| --- | --- | --- |
| a | 1003 | 27.6% |
| s | 520 | 14.3% |
| e | 511 | 14.1% |
| n | 511 | 14.1% |
| o | 492 | 13.5% |
| D | 464 | 12.8% |
| m | 47 | 1.3% |
| i | 28 | 0.8% |
| N | 19 | 0.5% |
| h | 19 | 0.5% |

| Value | Count | Frequency (%) |
| --- | --- | --- |
| a | 330 | 27.6% |
| s | 171 | 14.3% |
| e | 168 | 14.1% |
| n | 168 | 14.1% |
| o | 162 | 13.6% |
| D | 153 | 12.8% |
| m | 15 | 1.3% |
| i | 9 | 0.8% |
| N | 6 | 0.5% |
| h | 6 | 0.5% |

#### Most occurring blocks

| Value | Count | Frequency (%) |
| --- | --- | --- |
| ASCII | 11367 | 100.0% |

| Value | Count | Frequency (%) |
| --- | --- | --- |
| ASCII | 6855 | 100.0% |

| Value | Count | Frequency (%) |
| --- | --- | --- |
| ASCII | 2272 | 100.0% |

#### Most frequent character per block

##### *ASCII*

| Value | Count | Frequency (%) |
| --- | --- | --- |
|  | 3272 | 28.8% |
| a | 1679 | 14.8% |
| 1 | 868 | 7.6% |
| n | 857 | 7.5% |
| e | 857 | 7.5% |
| s | 838 | 7.4% |
| o | 822 | 7.2% |
| D | 806 | 7.1% |
| 4 | 374 | 3.3% |
| 7 | 322 | 2.8% |
| Other values (9) | 672 | 5.9% |

| Value | Count | Frequency (%) |
| --- | --- | --- |
|  | 1940 | 28.3% |
| a | 1003 | 14.6% |
| 1 | 580 | 8.5% |
| s | 520 | 7.6% |
| e | 511 | 7.5% |
| n | 511 | 7.5% |
| o | 492 | 7.2% |
| D | 464 | 6.8% |
| 4 | 210 | 3.1% |
| 8 | 198 | 2.9% |
| Other values (9) | 426 | 6.2% |

| Value | Count | Frequency (%) |
| --- | --- | --- |
|  | 639 | 28.1% |
| a | 330 | 14.5% |
| 1 | 218 | 9.6% |
| s | 171 | 7.5% |
| e | 168 | 7.4% |
| n | 168 | 7.4% |
| o | 162 | 7.1% |
| D | 153 | 6.7% |
| 8 | 81 | 3.6% |
| 4 | 59 | 2.6% |
| Other values (9) | 123 | 5.4% |

TIPOCUP  
Categorical

|
|  |
|
|  |

|  | Cluster 1 | Cluster 2 | Cluster 3 |
| --- | --- | --- | --- |
| Distinct | 5 | 5 | 5 |
| Distinct (%) | 0.6% | 1.0% | 3.0% |
| Missing | 0 | 0 | 0 |
| Missing (%) | 0.0% | 0.0% | 0.0% |
| Memory size | 13.4 KiB | 8.0 KiB | 2.6 KiB |

|  |  |
| --- | --- |
| Outra | 547 |
| Desempregado | 222 |
| Aposentado | 44 |
| Dona de Casa | 38 |
| Profissional de Saude | 6 |

|  |  |
| --- | --- |
| Outra | 337 |
| Dona de Casa | 59 |
| Desempregado | 57 |
| Aposentado | 44 |
| Profissional de Saude | 14 |

|  |  |
| --- | --- |
| Outra | 123 |
| Desempregado | 28 |
| Dona de Casa | 11 |
| Aposentado | 5 |
| Profissional de Saude | 1 |

More details

- Overview
- Categories
- Words
- Characters

Length

|  | Cluster 1 | Cluster 2 | Cluster 3 |
| --- | --- | --- | --- |
| Max length | 21 | 21 | 21 |
| Median length | 5 | 5 | 5 |
| Mean length | 7.4924154 | 7.4579256 | 6.8690476 |
| Min length | 5 | 5 | 5 |

Characters and Unicode

|  | Cluster 1 | Cluster 2 | Cluster 3 |
| --- | --- | --- | --- |
| Total characters | 6421 | 3811 | 1154 |
| Distinct characters | 22 | 22 | 22 |
| Distinct categories | 3 | 3 | 3 ? |
| Distinct scripts | 2 | 2 | 2 ? |
| Distinct blocks | 1 | 1 | 1 ? |

The Unicode Standard assigns character properties to each code point, which can be used to analyse textual variables.

Unique

|  | Cluster 1 | Cluster 2 | Cluster 3 |
| --- | --- | --- | --- |
| Unique | 0 | 0 | 1 ? |
| Unique (%) | 0.0% | 0.0% | 0.6% |

Sample

|  | Cluster 1 | Cluster 2 | Cluster 3 |
| --- | --- | --- | --- |
| 1st row | Outra | Outra | Outra |
| 2nd row | Desempregado | Outra | Outra |
| 3rd row | Outra | Dona de Casa | Desempregado |
| 4th row | Desempregado | Outra | Outra |
| 5th row | Outra | Outra | Outra |

#### Common Values

| Value | Count | Frequency (%) |
| --- | --- | --- |
| Outra | 547 | 63.8% |
| Desempregado | 222 | 25.9% |
| Aposentado | 44 | 5.1% |
| Dona de Casa | 38 | 4.4% |
| Profissional de Saude | 6 | 0.7% |

| Value | Count | Frequency (%) |
| --- | --- | --- |
| Outra | 337 | 65.9% |
| Dona de Casa | 59 | 11.5% |
| Desempregado | 57 | 11.2% |
| Aposentado | 44 | 8.6% |
| Profissional de Saude | 14 | 2.7% |

| Value | Count | Frequency (%) |
| --- | --- | --- |
| Outra | 123 | 73.2% |
| Desempregado | 28 | 16.7% |
| Dona de Casa | 11 | 6.5% |
| Aposentado | 5 | 3.0% |
| Profissional de Saude | 1 | 0.6% |

#### Length

xml version="1.0" encoding="utf-8" standalone="no"?2023-08-25T13:07:40.585436image/svg+xmlMatplotlib v3.6.0, https://matplotlib.org/ 

Histogram of lengths of the category

#### Common Values (Plot)

#### Cluster 1

xml version="1.0" encoding="utf-8" standalone="no"?2023-08-25T13:07:40.764725image/svg+xmlMatplotlib v3.6.0, https://matplotlib.org/

#### Cluster 2

xml version="1.0" encoding="utf-8" standalone="no"?2023-08-25T13:07:40.925444image/svg+xmlMatplotlib v3.6.0, https://matplotlib.org/

#### Cluster 3

xml version="1.0" encoding="utf-8" standalone="no"?2023-08-25T13:07:41.127775image/svg+xmlMatplotlib v3.6.0, https://matplotlib.org/

| Value | Count | Frequency (%) |
| --- | --- | --- |
| outra | 547 | 57.9% |
| desempregado | 222 | 23.5% |
| aposentado | 44 | 4.7% |
| de | 44 | 4.7% |
| dona | 38 | 4.0% |
| casa | 38 | 4.0% |
| profissional | 6 | 0.6% |
| saude | 6 | 0.6% |

| Value | Count | Frequency (%) |
| --- | --- | --- |
| outra | 337 | 51.3% |
| de | 73 | 11.1% |
| dona | 59 | 9.0% |
| casa | 59 | 9.0% |
| desempregado | 57 | 8.7% |
| aposentado | 44 | 6.7% |
| profissional | 14 | 2.1% |
| saude | 14 | 2.1% |

| Value | Count | Frequency (%) |
| --- | --- | --- |
| outra | 123 | 64.1% |
| desempregado | 28 | 14.6% |
| de | 12 | 6.2% |
| dona | 11 | 5.7% |
| casa | 11 | 5.7% |
| aposentado | 5 | 2.6% |
| profissional | 1 | 0.5% |
| saude | 1 | 0.5% |

- Characters
- Categories
- Scripts
- Blocks

#### Most occurring characters

| Value | Count | Frequency (%) |
| --- | --- | --- |
| a | 939 | 14.6% |
| r | 775 | 12.1% |
| e | 760 | 11.8% |
| t | 591 | 9.2% |
| u | 553 | 8.6% |
| O | 547 | 8.5% |
| o | 360 | 5.6% |
| d | 316 | 4.9% |
| s | 316 | 4.9% |
| p | 266 | 4.1% |
| Other values (12) | 998 | 15.5% |

| Value | Count | Frequency (%) |
| --- | --- | --- |
| a | 643 | 16.9% |
| r | 408 | 10.7% |
| t | 381 | 10.0% |
| u | 351 | 9.2% |
| O | 337 | 8.8% |
| e | 302 | 7.9% |
| o | 232 | 6.1% |
| d | 188 | 4.9% |
| s | 188 | 4.9% |
|  | 146 | 3.8% |
| Other values (12) | 635 | 16.7% |

| Value | Count | Frequency (%) |
| --- | --- | --- |
| a | 191 | 16.6% |
| r | 152 | 13.2% |
| t | 128 | 11.1% |
| u | 124 | 10.7% |
| O | 123 | 10.7% |
| e | 102 | 8.8% |
| o | 51 | 4.4% |
| d | 46 | 4.0% |
| s | 46 | 4.0% |
| D | 39 | 3.4% |
| Other values (12) | 152 | 13.2% |

#### Most occurring categories

| Value | Count | Frequency (%) |
| --- | --- | --- |
| Lowercase Letter | 5432 | 84.6% |
| Uppercase Letter | 901 | 14.0% |
| Space Separator | 88 | 1.4% |

| Value | Count | Frequency (%) |
| --- | --- | --- |
| Lowercase Letter | 3081 | 80.8% |
| Uppercase Letter | 584 | 15.3% |
| Space Separator | 146 | 3.8% |

| Value | Count | Frequency (%) |
| --- | --- | --- |
| Lowercase Letter | 950 | 82.3% |
| Uppercase Letter | 180 | 15.6% |
| Space Separator | 24 | 2.1% |

#### Most frequent character per category

##### *Lowercase Letter*

| Value | Count | Frequency (%) |
| --- | --- | --- |
| a | 939 | 17.3% |
| r | 775 | 14.3% |
| e | 760 | 14.0% |
| t | 591 | 10.9% |
| u | 553 | 10.2% |
| o | 360 | 6.6% |
| d | 316 | 5.8% |
| s | 316 | 5.8% |
| p | 266 | 4.9% |
| m | 222 | 4.1% |
| Other values (5) | 334 | 6.1% |

| Value | Count | Frequency (%) |
| --- | --- | --- |
| a | 643 | 20.9% |
| r | 408 | 13.2% |
| t | 381 | 12.4% |
| u | 351 | 11.4% |
| e | 302 | 9.8% |
| o | 232 | 7.5% |
| d | 188 | 6.1% |
| s | 188 | 6.1% |
| n | 117 | 3.8% |
| p | 101 | 3.3% |
| Other values (5) | 170 | 5.5% |

| Value | Count | Frequency (%) |
| --- | --- | --- |
| a | 191 | 20.1% |
| r | 152 | 16.0% |
| t | 128 | 13.5% |
| u | 124 | 13.1% |
| e | 102 | 10.7% |
| o | 51 | 5.4% |
| d | 46 | 4.8% |
| s | 46 | 4.8% |
| p | 33 | 3.5% |
| m | 28 | 2.9% |
| Other values (5) | 49 | 5.2% |

##### *Uppercase Letter*

| Value | Count | Frequency (%) |
| --- | --- | --- |
| O | 547 | 60.7% |
| D | 260 | 28.9% |
| A | 44 | 4.9% |
| C | 38 | 4.2% |
| P | 6 | 0.7% |
| S | 6 | 0.7% |

| Value | Count | Frequency (%) |
| --- | --- | --- |
| O | 337 | 57.7% |
| D | 116 | 19.9% |
| C | 59 | 10.1% |
| A | 44 | 7.5% |
| P | 14 | 2.4% |
| S | 14 | 2.4% |

| Value | Count | Frequency (%) |
| --- | --- | --- |
| O | 123 | 68.3% |
| D | 39 | 21.7% |
| C | 11 | 6.1% |
| A | 5 | 2.8% |
| P | 1 | 0.6% |
| S | 1 | 0.6% |

##### *Space Separator*

| Value | Count | Frequency (%) |
| --- | --- | --- |
|  | 88 | 100.0% |

| Value | Count | Frequency (%) |
| --- | --- | --- |
|  | 146 | 100.0% |

| Value | Count | Frequency (%) |
| --- | --- | --- |
|  | 24 | 100.0% |

#### Most occurring scripts

| Value | Count | Frequency (%) |
| --- | --- | --- |
| Latin | 6333 | 98.6% |
| Common | 88 | 1.4% |

| Value | Count | Frequency (%) |
| --- | --- | --- |
| Latin | 3665 | 96.2% |
| Common | 146 | 3.8% |

| Value | Count | Frequency (%) |
| --- | --- | --- |
| Latin | 1130 | 97.9% |
| Common | 24 | 2.1% |

#### Most frequent character per script

##### *Latin*

| Value | Count | Frequency (%) |
| --- | --- | --- |
| a | 939 | 14.8% |
| r | 775 | 12.2% |
| e | 760 | 12.0% |
| t | 591 | 9.3% |
| u | 553 | 8.7% |
| O | 547 | 8.6% |
| o | 360 | 5.7% |
| d | 316 | 5.0% |
| s | 316 | 5.0% |
| p | 266 | 4.2% |
| Other values (11) | 910 | 14.4% |

| Value | Count | Frequency (%) |
| --- | --- | --- |
| a | 643 | 17.5% |
| r | 408 | 11.1% |
| t | 381 | 10.4% |
| u | 351 | 9.6% |
| O | 337 | 9.2% |
| e | 302 | 8.2% |
| o | 232 | 6.3% |
| d | 188 | 5.1% |
| s | 188 | 5.1% |
| n | 117 | 3.2% |
| Other values (11) | 518 | 14.1% |

| Value | Count | Frequency (%) |
| --- | --- | --- |
| a | 191 | 16.9% |
| r | 152 | 13.5% |
| t | 128 | 11.3% |
| u | 124 | 11.0% |
| O | 123 | 10.9% |
| e | 102 | 9.0% |
| o | 51 | 4.5% |
| d | 46 | 4.1% |
| s | 46 | 4.1% |
| D | 39 | 3.5% |
| Other values (11) | 128 | 11.3% |

##### *Common*

| Value | Count | Frequency (%) |
| --- | --- | --- |
|  | 88 | 100.0% |

| Value | Count | Frequency (%) |
| --- | --- | --- |
|  | 146 | 100.0% |

| Value | Count | Frequency (%) |
| --- | --- | --- |
|  | 24 | 100.0% |

#### Most occurring blocks

| Value | Count | Frequency (%) |
| --- | --- | --- |
| ASCII | 6421 | 100.0% |

| Value | Count | Frequency (%) |
| --- | --- | --- |
| ASCII | 3811 | 100.0% |

| Value | Count | Frequency (%) |
| --- | --- | --- |
| ASCII | 1154 | 100.0% |

#### Most frequent character per block

##### *ASCII*

| Value | Count | Frequency (%) |
| --- | --- | --- |
| a | 939 | 14.6% |
| r | 775 | 12.1% |
| e | 760 | 11.8% |
| t | 591 | 9.2% |
| u | 553 | 8.6% |
| O | 547 | 8.5% |
| o | 360 | 5.6% |
| d | 316 | 4.9% |
| s | 316 | 4.9% |
| p | 266 | 4.1% |
| Other values (12) | 998 | 15.5% |

| Value | Count | Frequency (%) |
| --- | --- | --- |
| a | 643 | 16.9% |
| r | 408 | 10.7% |
| t | 381 | 10.0% |
| u | 351 | 9.2% |
| O | 337 | 8.8% |
| e | 302 | 7.9% |
| o | 232 | 6.1% |
| d | 188 | 4.9% |
| s | 188 | 4.9% |
|  | 146 | 3.8% |
| Other values (12) | 635 | 16.7% |

| Value | Count | Frequency (%) |
| --- | --- | --- |
| a | 191 | 16.6% |
| r | 152 | 13.2% |
| t | 128 | 11.1% |
| u | 124 | 10.7% |
| O | 123 | 10.7% |
| e | 102 | 8.8% |
| o | 51 | 4.4% |
| d | 46 | 4.0% |
| s | 46 | 4.0% |
| D | 39 | 3.4% |
| Other values (12) | 152 | 13.2% |

sitAtual  
Categorical

|
|  |
|
|  |

|  | Cluster 1 | Cluster 2 | Cluster 3 |
| --- | --- | --- | --- |
| Distinct | 2 | 2 | 2 |
| Distinct (%) | 0.2% | 0.4% | 1.2% |
| Missing | 0 | 0 | 0 |
| Missing (%) | 0.0% | 0.0% | 0.0% |
| Memory size | 13.4 KiB | 8.0 KiB | 2.6 KiB |

|  |  |
| --- | --- |
| Cura | 717 |
| Abandono | 140 |

|  |  |
| --- | --- |
| Cura | 470 |
| Abandono | 41 |

|  |  |
| --- | --- |
| Cura | 121 |
| Abandono | 47 |

More details

- Overview
- Categories
- Words
- Characters

Length

|  | Cluster 1 | Cluster 2 | Cluster 3 |
| --- | --- | --- | --- |
| Max length | 8 | 8 | 8 |
| Median length | 4 | 4 | 4 |
| Mean length | 4.6534422 | 4.3209393 | 5.1190476 |
| Min length | 4 | 4 | 4 |

Characters and Unicode

|  | Cluster 1 | Cluster 2 | Cluster 3 |
| --- | --- | --- | --- |
| Total characters | 3988 | 2208 | 860 |
| Distinct characters | 9 | 9 | 9 |
| Distinct categories | 2 | 2 | 2 ? |
| Distinct scripts | 1 | 1 | 1 ? |
| Distinct blocks | 1 | 1 | 1 ? |

The Unicode Standard assigns character properties to each code point, which can be used to analyse textual variables.

Unique

|  | Cluster 1 | Cluster 2 | Cluster 3 |
| --- | --- | --- | --- |
| Unique | 0 | 0 | 0 ? |
| Unique (%) | 0.0% | 0.0% | 0.0% |

Sample

|  | Cluster 1 | Cluster 2 | Cluster 3 |
| --- | --- | --- | --- |
| 1st row | Cura | Cura | Cura |
| 2nd row | Cura | Cura | Cura |
| 3rd row | Cura | Cura | Abandono |
| 4th row | Cura | Cura | Cura |
| 5th row | Cura | Cura | Cura |

#### Common Values

| Value | Count | Frequency (%) |
| --- | --- | --- |
| Cura | 717 | 83.7% |
| Abandono | 140 | 16.3% |

| Value | Count | Frequency (%) |
| --- | --- | --- |
| Cura | 470 | 92.0% |
| Abandono | 41 | 8.0% |

| Value | Count | Frequency (%) |
| --- | --- | --- |
| Cura | 121 | 72.0% |
| Abandono | 47 | 28.0% |

#### Length

xml version="1.0" encoding="utf-8" standalone="no"?2023-08-25T13:07:41.304873image/svg+xmlMatplotlib v3.6.0, https://matplotlib.org/ 

Histogram of lengths of the category

#### Common Values (Plot)

#### Cluster 1

xml version="1.0" encoding="utf-8" standalone="no"?2023-08-25T13:07:41.480583image/svg+xmlMatplotlib v3.6.0, https://matplotlib.org/

#### Cluster 2

xml version="1.0" encoding="utf-8" standalone="no"?2023-08-25T13:07:41.625492image/svg+xmlMatplotlib v3.6.0, https://matplotlib.org/

#### Cluster 3

xml version="1.0" encoding="utf-8" standalone="no"?2023-08-25T13:07:41.766824image/svg+xmlMatplotlib v3.6.0, https://matplotlib.org/

| Value | Count | Frequency (%) |
| --- | --- | --- |
| cura | 717 | 83.7% |
| abandono | 140 | 16.3% |

| Value | Count | Frequency (%) |
| --- | --- | --- |
| cura | 470 | 92.0% |
| abandono | 41 | 8.0% |

| Value | Count | Frequency (%) |
| --- | --- | --- |
| cura | 121 | 72.0% |
| abandono | 47 | 28.0% |

- Characters
- Categories
- Scripts
- Blocks

#### Most occurring characters

| Value | Count | Frequency (%) |
| --- | --- | --- |
| a | 857 | 21.5% |
| C | 717 | 18.0% |
| u | 717 | 18.0% |
| r | 717 | 18.0% |
| n | 280 | 7.0% |
| o | 280 | 7.0% |
| A | 140 | 3.5% |
| b | 140 | 3.5% |
| d | 140 | 3.5% |

| Value | Count | Frequency (%) |
| --- | --- | --- |
| a | 511 | 23.1% |
| C | 470 | 21.3% |
| u | 470 | 21.3% |
| r | 470 | 21.3% |
| n | 82 | 3.7% |
| o | 82 | 3.7% |
| A | 41 | 1.9% |
| b | 41 | 1.9% |
| d | 41 | 1.9% |

| Value | Count | Frequency (%) |
| --- | --- | --- |
| a | 168 | 19.5% |
| C | 121 | 14.1% |
| u | 121 | 14.1% |
| r | 121 | 14.1% |
| n | 94 | 10.9% |
| o | 94 | 10.9% |
| A | 47 | 5.5% |
| b | 47 | 5.5% |
| d | 47 | 5.5% |

#### Most occurring categories

| Value | Count | Frequency (%) |
| --- | --- | --- |
| Lowercase Letter | 3131 | 78.5% |
| Uppercase Letter | 857 | 21.5% |

| Value | Count | Frequency (%) |
| --- | --- | --- |
| Lowercase Letter | 1697 | 76.9% |
| Uppercase Letter | 511 | 23.1% |

| Value | Count | Frequency (%) |
| --- | --- | --- |
| Lowercase Letter | 692 | 80.5% |
| Uppercase Letter | 168 | 19.5% |

#### Most frequent character per category

##### *Lowercase Letter*

| Value | Count | Frequency (%) |
| --- | --- | --- |
| a | 857 | 27.4% |
| u | 717 | 22.9% |
| r | 717 | 22.9% |
| n | 280 | 8.9% |
| o | 280 | 8.9% |
| b | 140 | 4.5% |
| d | 140 | 4.5% |

| Value | Count | Frequency (%) |
| --- | --- | --- |
| a | 511 | 30.1% |
| u | 470 | 27.7% |
| r | 470 | 27.7% |
| n | 82 | 4.8% |
| o | 82 | 4.8% |
| b | 41 | 2.4% |
| d | 41 | 2.4% |

| Value | Count | Frequency (%) |
| --- | --- | --- |
| a | 168 | 24.3% |
| u | 121 | 17.5% |
| r | 121 | 17.5% |
| n | 94 | 13.6% |
| o | 94 | 13.6% |
| b | 47 | 6.8% |
| d | 47 | 6.8% |

##### *Uppercase Letter*

| Value | Count | Frequency (%) |
| --- | --- | --- |
| C | 717 | 83.7% |
| A | 140 | 16.3% |

| Value | Count | Frequency (%) |
| --- | --- | --- |
| C | 470 | 92.0% |
| A | 41 | 8.0% |

| Value | Count | Frequency (%) |
| --- | --- | --- |
| C | 121 | 72.0% |
| A | 47 | 28.0% |

#### Most occurring scripts

| Value | Count | Frequency (%) |
| --- | --- | --- |
| Latin | 3988 | 100.0% |

| Value | Count | Frequency (%) |
| --- | --- | --- |
| Latin | 2208 | 100.0% |

| Value | Count | Frequency (%) |
| --- | --- | --- |
| Latin | 860 | 100.0% |

#### Most frequent character per script

##### *Latin*

| Value | Count | Frequency (%) |
| --- | --- | --- |
| a | 857 | 21.5% |
| C | 717 | 18.0% |
| u | 717 | 18.0% |
| r | 717 | 18.0% |
| n | 280 | 7.0% |
| o | 280 | 7.0% |
| A | 140 | 3.5% |
| b | 140 | 3.5% |
| d | 140 | 3.5% |

| Value | Count | Frequency (%) |
| --- | --- | --- |
| a | 511 | 23.1% |
| C | 470 | 21.3% |
| u | 470 | 21.3% |
| r | 470 | 21.3% |
| n | 82 | 3.7% |
| o | 82 | 3.7% |
| A | 41 | 1.9% |
| b | 41 | 1.9% |
| d | 41 | 1.9% |

| Value | Count | Frequency (%) |
| --- | --- | --- |
| a | 168 | 19.5% |
| C | 121 | 14.1% |
| u | 121 | 14.1% |
| r | 121 | 14.1% |
| n | 94 | 10.9% |
| o | 94 | 10.9% |
| A | 47 | 5.5% |
| b | 47 | 5.5% |
| d | 47 | 5.5% |

#### Most occurring blocks

| Value | Count | Frequency (%) |
| --- | --- | --- |
| ASCII | 3988 | 100.0% |

| Value | Count | Frequency (%) |
| --- | --- | --- |
| ASCII | 2208 | 100.0% |

| Value | Count | Frequency (%) |
| --- | --- | --- |
| ASCII | 860 | 100.0% |

#### Most frequent character per block

##### *ASCII*

| Value | Count | Frequency (%) |
| --- | --- | --- |
| a | 857 | 21.5% |
| C | 717 | 18.0% |
| u | 717 | 18.0% |
| r | 717 | 18.0% |
| n | 280 | 7.0% |
| o | 280 | 7.0% |
| A | 140 | 3.5% |
| b | 140 | 3.5% |
| d | 140 | 3.5% |

| Value | Count | Frequency (%) |
| --- | --- | --- |
| a | 511 | 23.1% |
| C | 470 | 21.3% |
| u | 470 | 21.3% |
| r | 470 | 21.3% |
| n | 82 | 3.7% |
| o | 82 | 3.7% |
| A | 41 | 1.9% |
| b | 41 | 1.9% |
| d | 41 | 1.9% |

| Value | Count | Frequency (%) |
| --- | --- | --- |
| a | 168 | 19.5% |
| C | 121 | 14.1% |
| u | 121 | 14.1% |
| r | 121 | 14.1% |
| n | 94 | 10.9% |
| o | 94 | 10.9% |
| A | 47 | 5.5% |
| b | 47 | 5.5% |
| d | 47 | 5.5% |

tipoCaso  
Categorical

|
|  |
|
|  |

|  | Cluster 1 | Cluster 2 | Cluster 3 |
| --- | --- | --- | --- |
| Distinct | 3 | 3 | 4 |
| Distinct (%) | 0.4% | 0.6% | 2.4% |
| Missing | 0 | 0 | 0 |
| Missing (%) | 0.0% | 0.0% | 0.0% |
| Memory size | 13.4 KiB | 8.0 KiB | 2.6 KiB |

|  |  |
| --- | --- |
| Novo | 823 |
| Recidiva | 21 |
| Retr Aband | 13 |

|  |  |
| --- | --- |
| Novo | 500 |
| Retr Aband | 6 |
| Recidiva | 5 |

|  |  |
| --- | --- |
| Novo | 154 |
| Retr Aband | 7 |
| Recidiva | 6 |
| Retrat apos falencia/resistencia | 1 |

More details

- Overview
- Categories
- Words
- Characters

Length

|  | Cluster 1 | Cluster 2 | Cluster 3 |
| --- | --- | --- | --- |
| Max length | 10 | 10 | 32 |
| Median length | 4 | 4 | 4 |
| Mean length | 4.1890315 | 4.109589 | 4.5595238 |
| Min length | 4 | 4 | 4 |

Characters and Unicode

|  | Cluster 1 | Cluster 2 | Cluster 3 |
| --- | --- | --- | --- |
| Total characters | 3590 | 2100 | 766 |
| Distinct characters | 15 | 15 | 20 |
| Distinct categories | 3 | 3 | 4 ? |
| Distinct scripts | 2 | 2 | 2 ? |
| Distinct blocks | 1 | 1 | 1 ? |

The Unicode Standard assigns character properties to each code point, which can be used to analyse textual variables.

Unique

|  | Cluster 1 | Cluster 2 | Cluster 3 |
| --- | --- | --- | --- |
| Unique | 0 | 0 | 1 ? |
| Unique (%) | 0.0% | 0.0% | 0.6% |

Sample

|  | Cluster 1 | Cluster 2 | Cluster 3 |
| --- | --- | --- | --- |
| 1st row | Novo | Novo | Novo |
| 2nd row | Novo | Novo | Novo |
| 3rd row | Novo | Novo | Novo |
| 4th row | Novo | Novo | Novo |
| 5th row | Novo | Novo | Novo |

#### Common Values

| Value | Count | Frequency (%) |
| --- | --- | --- |
| Novo | 823 | 96.0% |
| Recidiva | 21 | 2.5% |
| Retr Aband | 13 | 1.5% |

| Value | Count | Frequency (%) |
| --- | --- | --- |
| Novo | 500 | 97.8% |
| Retr Aband | 6 | 1.2% |
| Recidiva | 5 | 1.0% |

| Value | Count | Frequency (%) |
| --- | --- | --- |
| Novo | 154 | 91.7% |
| Retr Aband | 7 | 4.2% |
| Recidiva | 6 | 3.6% |
| Retrat apos falencia/resistencia | 1 | 0.6% |

#### Length

xml version="1.0" encoding="utf-8" standalone="no"?2023-08-25T13:07:41.896419image/svg+xmlMatplotlib v3.6.0, https://matplotlib.org/ 

Histogram of lengths of the category

#### Common Values (Plot)

#### Cluster 1

xml version="1.0" encoding="utf-8" standalone="no"?2023-08-25T13:07:42.385781image/svg+xmlMatplotlib v3.6.0, https://matplotlib.org/

#### Cluster 2

xml version="1.0" encoding="utf-8" standalone="no"?2023-08-25T13:07:42.514139image/svg+xmlMatplotlib v3.6.0, https://matplotlib.org/

#### Cluster 3

xml version="1.0" encoding="utf-8" standalone="no"?2023-08-25T13:07:42.650715image/svg+xmlMatplotlib v3.6.0, https://matplotlib.org/

| Value | Count | Frequency (%) |
| --- | --- | --- |
| novo | 823 | 94.6% |
| recidiva | 21 | 2.4% |
| retr | 13 | 1.5% |
| aband | 13 | 1.5% |

| Value | Count | Frequency (%) |
| --- | --- | --- |
| novo | 500 | 96.7% |
| retr | 6 | 1.2% |
| aband | 6 | 1.2% |
| recidiva | 5 | 1.0% |

| Value | Count | Frequency (%) |
| --- | --- | --- |
| novo | 154 | 87.0% |
| retr | 7 | 4.0% |
| aband | 7 | 4.0% |
| recidiva | 6 | 3.4% |
| retrat | 1 | 0.6% |
| apos | 1 | 0.6% |
| falencia/resistencia | 1 | 0.6% |

- Characters
- Categories
- Scripts
- Blocks

#### Most occurring characters

| Value | Count | Frequency (%) |
| --- | --- | --- |
| o | 1646 | 45.8% |
| v | 844 | 23.5% |
| N | 823 | 22.9% |
| i | 42 | 1.2% |
| R | 34 | 0.9% |
| e | 34 | 0.9% |
| d | 34 | 0.9% |
| a | 34 | 0.9% |
| c | 21 | 0.6% |
| t | 13 | 0.4% |
| Other values (5) | 65 | 1.8% |

| Value | Count | Frequency (%) |
| --- | --- | --- |
| o | 1000 | 47.6% |
| v | 505 | 24.0% |
| N | 500 | 23.8% |
| R | 11 | 0.5% |
| e | 11 | 0.5% |
| a | 11 | 0.5% |
| d | 11 | 0.5% |
| i | 10 | 0.5% |
| t | 6 | 0.3% |
| r | 6 | 0.3% |
| Other values (5) | 29 | 1.4% |

| Value | Count | Frequency (%) |
| --- | --- | --- |
| o | 309 | 40.3% |
| v | 160 | 20.9% |
| N | 154 | 20.1% |
| a | 18 | 2.3% |
| e | 17 | 2.2% |
| i | 15 | 2.0% |
| R | 14 | 1.8% |
| d | 13 | 1.7% |
| t | 10 | 1.3% |
| r | 9 | 1.2% |
| Other values (10) | 47 | 6.1% |

#### Most occurring categories

| Value | Count | Frequency (%) |
| --- | --- | --- |
| Lowercase Letter | 2707 | 75.4% |
| Uppercase Letter | 870 | 24.2% |
| Space Separator | 13 | 0.4% |

| Value | Count | Frequency (%) |
| --- | --- | --- |
| Lowercase Letter | 1577 | 75.1% |
| Uppercase Letter | 517 | 24.6% |
| Space Separator | 6 | 0.3% |

| Value | Count | Frequency (%) |
| --- | --- | --- |
| Lowercase Letter | 581 | 75.8% |
| Uppercase Letter | 175 | 22.8% |
| Space Separator | 9 | 1.2% |
| Other Punctuation | 1 | 0.1% |

#### Most frequent character per category

##### *Lowercase Letter*

| Value | Count | Frequency (%) |
| --- | --- | --- |
| o | 1646 | 60.8% |
| v | 844 | 31.2% |
| i | 42 | 1.6% |
| e | 34 | 1.3% |
| d | 34 | 1.3% |
| a | 34 | 1.3% |
| c | 21 | 0.8% |
| t | 13 | 0.5% |
| r | 13 | 0.5% |
| b | 13 | 0.5% |

| Value | Count | Frequency (%) |
| --- | --- | --- |
| o | 1000 | 63.4% |
| v | 505 | 32.0% |
| e | 11 | 0.7% |
| a | 11 | 0.7% |
| d | 11 | 0.7% |
| i | 10 | 0.6% |
| t | 6 | 0.4% |
| r | 6 | 0.4% |
| b | 6 | 0.4% |
| n | 6 | 0.4% |

| Value | Count | Frequency (%) |
| --- | --- | --- |
| o | 309 | 53.2% |
| v | 160 | 27.5% |
| a | 18 | 3.1% |
| e | 17 | 2.9% |
| i | 15 | 2.6% |
| d | 13 | 2.2% |
| t | 10 | 1.7% |
| r | 9 | 1.5% |
| n | 9 | 1.5% |
| c | 8 | 1.4% |
| Other values (5) | 13 | 2.2% |

##### *Uppercase Letter*

| Value | Count | Frequency (%) |
| --- | --- | --- |
| N | 823 | 94.6% |
| R | 34 | 3.9% |
| A | 13 | 1.5% |

| Value | Count | Frequency (%) |
| --- | --- | --- |
| N | 500 | 96.7% |
| R | 11 | 2.1% |
| A | 6 | 1.2% |

| Value | Count | Frequency (%) |
| --- | --- | --- |
| N | 154 | 88.0% |
| R | 14 | 8.0% |
| A | 7 | 4.0% |

##### *Space Separator*

| Value | Count | Frequency (%) |
| --- | --- | --- |
|  | 13 | 100.0% |

| Value | Count | Frequency (%) |
| --- | --- | --- |
|  | 6 | 100.0% |

| Value | Count | Frequency (%) |
| --- | --- | --- |
|  | 9 | 100.0% |

##### *Other Punctuation*

| Value | Count | Frequency (%) |
| --- | --- | --- |
| / | 1 | 100.0% |

#### Most occurring scripts

| Value | Count | Frequency (%) |
| --- | --- | --- |
| Latin | 3577 | 99.6% |
| Common | 13 | 0.4% |

| Value | Count | Frequency (%) |
| --- | --- | --- |
| Latin | 2094 | 99.7% |
| Common | 6 | 0.3% |

| Value | Count | Frequency (%) |
| --- | --- | --- |
| Latin | 756 | 98.7% |
| Common | 10 | 1.3% |

#### Most frequent character per script

##### *Latin*

| Value | Count | Frequency (%) |
| --- | --- | --- |
| o | 1646 | 46.0% |
| v | 844 | 23.6% |
| N | 823 | 23.0% |
| i | 42 | 1.2% |
| R | 34 | 1.0% |
| e | 34 | 1.0% |
| d | 34 | 1.0% |
| a | 34 | 1.0% |
| c | 21 | 0.6% |
| t | 13 | 0.4% |
| Other values (4) | 52 | 1.5% |

| Value | Count | Frequency (%) |
| --- | --- | --- |
| o | 1000 | 47.8% |
| v | 505 | 24.1% |
| N | 500 | 23.9% |
| R | 11 | 0.5% |
| e | 11 | 0.5% |
| a | 11 | 0.5% |
| d | 11 | 0.5% |
| i | 10 | 0.5% |
| t | 6 | 0.3% |
| r | 6 | 0.3% |
| Other values (4) | 23 | 1.1% |

| Value | Count | Frequency (%) |
| --- | --- | --- |
| o | 309 | 40.9% |
| v | 160 | 21.2% |
| N | 154 | 20.4% |
| a | 18 | 2.4% |
| e | 17 | 2.2% |
| i | 15 | 2.0% |
| R | 14 | 1.9% |
| d | 13 | 1.7% |
| t | 10 | 1.3% |
| r | 9 | 1.2% |
| Other values (8) | 37 | 4.9% |

##### *Common*

| Value | Count | Frequency (%) |
| --- | --- | --- |
|  | 13 | 100.0% |

| Value | Count | Frequency (%) |
| --- | --- | --- |
|  | 6 | 100.0% |

| Value | Count | Frequency (%) |
| --- | --- | --- |
|  | 9 | 90.0% |
| / | 1 | 10.0% |

#### Most occurring blocks

| Value | Count | Frequency (%) |
| --- | --- | --- |
| ASCII | 3590 | 100.0% |

| Value | Count | Frequency (%) |
| --- | --- | --- |
| ASCII | 2100 | 100.0% |

| Value | Count | Frequency (%) |
| --- | --- | --- |
| ASCII | 766 | 100.0% |

#### Most frequent character per block

##### *ASCII*

| Value | Count | Frequency (%) |
| --- | --- | --- |
| o | 1646 | 45.8% |
| v | 844 | 23.5% |
| N | 823 | 22.9% |
| i | 42 | 1.2% |
| R | 34 | 0.9% |
| e | 34 | 0.9% |
| d | 34 | 0.9% |
| a | 34 | 0.9% |
| c | 21 | 0.6% |
| t | 13 | 0.4% |
| Other values (5) | 65 | 1.8% |

| Value | Count | Frequency (%) |
| --- | --- | --- |
| o | 1000 | 47.6% |
| v | 505 | 24.0% |
| N | 500 | 23.8% |
| R | 11 | 0.5% |
| e | 11 | 0.5% |
| a | 11 | 0.5% |
| d | 11 | 0.5% |
| i | 10 | 0.5% |
| t | 6 | 0.3% |
| r | 6 | 0.3% |
| Other values (5) | 29 | 1.4% |

| Value | Count | Frequency (%) |
| --- | --- | --- |
| o | 309 | 40.3% |
| v | 160 | 20.9% |
| N | 154 | 20.1% |
| a | 18 | 2.3% |
| e | 17 | 2.2% |
| i | 15 | 2.0% |
| R | 14 | 1.8% |
| d | 13 | 1.7% |
| t | 10 | 1.3% |
| r | 9 | 1.2% |
| Other values (10) | 47 | 6.1% |

FORMACLIN1  
Categorical

|
|  |
|
|  |

|  | Cluster 1 | Cluster 2 | Cluster 3 |
| --- | --- | --- | --- |
| Distinct | 8 | 10 | 8 |
| Distinct (%) | 0.9% | 2.0% | 4.8% |
| Missing | 0 | 0 | 0 |
| Missing (%) | 0.0% | 0.0% | 0.0% |
| Memory size | 13.4 KiB | 8.0 KiB | 2.6 KiB |

|  |  |
| --- | --- |
| Pul | 818 |
| Pleural | 27 |
| Ganglionar Periferica | 5 |
| Meningea | 3 |
| Vias Urinarias | 1 |
| Other values (3) | 3 |

|  |  |
| --- | --- |
| Pul | 435 |
| Pleural | 43 |
| Ganglionar Periferica | 13 |
| Oftalmica | 6 |
| Pele | 5 |
| Other values (5) | 9 |

|  |  |
| --- | --- |
| Pul | 134 |
| Pleural | 15 |
| Ganglionar Periferica | 6 |
| Meningea | 5 |
| Miliar | 3 |
| Other values (3) | 5 |

More details

- Overview
- Categories
- Words
- Characters

Length

|  | Cluster 1 | Cluster 2 | Cluster 3 |
| --- | --- | --- | --- |
| Max length | 21 | 21 | 21 |
| Median length | 3 | 3 | 3 |
| Mean length | 3.2823804 | 3.9412916 | 4.4285714 |
| Min length | 3 | 3 | 3 |

Characters and Unicode

|  | Cluster 1 | Cluster 2 | Cluster 3 |
| --- | --- | --- | --- |
| Total characters | 2813 | 2014 | 744 |
| Distinct characters | 21 | 21 | 20 |
| Distinct categories | 3 | 3 | 3 ? |
| Distinct scripts | 2 | 2 | 2 ? |
| Distinct blocks | 1 | 1 | 1 ? |

The Unicode Standard assigns character properties to each code point, which can be used to analyse textual variables.

Unique

|  | Cluster 1 | Cluster 2 | Cluster 3 |
| --- | --- | --- | --- |
| Unique | 4 | 2 | 1 ? |
| Unique (%) | 0.5% | 0.4% | 0.6% |

Sample

|  | Cluster 1 | Cluster 2 | Cluster 3 |
| --- | --- | --- | --- |
| 1st row | Pul | Ganglionar Periferica | Pul |
| 2nd row | Pul | Pul | Pul |
| 3rd row | Pul | Pleural | Pul |
| 4th row | Pul | Pul | Pleural |
| 5th row | Pul | Pul | Pul |

#### Common Values

| Value | Count | Frequency (%) |
| --- | --- | --- |
| Pul | 818 | 95.4% |
| Pleural | 27 | 3.2% |
| Ganglionar Periferica | 5 | 0.6% |
| Meningea | 3 | 0.4% |
| Vias Urinarias | 1 | 0.1% |
| Ossea | 1 | 0.1% |
| Outras | 1 | 0.1% |
| Multiplos Orgaos | 1 | 0.1% |

| Value | Count | Frequency (%) |
| --- | --- | --- |
| Pul | 435 | 85.1% |
| Pleural | 43 | 8.4% |
| Ganglionar Periferica | 13 | 2.5% |
| Oftalmica | 6 | 1.2% |
| Pele | 5 | 1.0% |
| Outras | 3 | 0.6% |
| Ossea | 2 | 0.4% |
| Miliar | 2 | 0.4% |
| Vias Urinarias | 1 | 0.2% |
| Genital | 1 | 0.2% |

| Value | Count | Frequency (%) |
| --- | --- | --- |
| Pul | 134 | 79.8% |
| Pleural | 15 | 8.9% |
| Ganglionar Periferica | 6 | 3.6% |
| Meningea | 5 | 3.0% |
| Miliar | 3 | 1.8% |
| Outras | 2 | 1.2% |
| Multiplos Orgaos | 2 | 1.2% |
| Oftalmica | 1 | 0.6% |

#### Length

xml version="1.0" encoding="utf-8" standalone="no"?2023-08-25T13:07:42.803830image/svg+xmlMatplotlib v3.6.0, https://matplotlib.org/ 

Histogram of lengths of the category

#### Common Values (Plot)

#### Cluster 1

xml version="1.0" encoding="utf-8" standalone="no"?2023-08-25T13:07:43.004199image/svg+xmlMatplotlib v3.6.0, https://matplotlib.org/

#### Cluster 2

xml version="1.0" encoding="utf-8" standalone="no"?2023-08-25T13:07:43.204636image/svg+xmlMatplotlib v3.6.0, https://matplotlib.org/

#### Cluster 3

xml version="1.0" encoding="utf-8" standalone="no"?2023-08-25T13:07:43.401469image/svg+xmlMatplotlib v3.6.0, https://matplotlib.org/

| Value | Count | Frequency (%) |
| --- | --- | --- |
| pul | 818 | 94.7% |
| pleural | 27 | 3.1% |
| ganglionar | 5 | 0.6% |
| periferica | 5 | 0.6% |
| meningea | 3 | 0.3% |
| vias | 1 | 0.1% |
| urinarias | 1 | 0.1% |
| ossea | 1 | 0.1% |
| outras | 1 | 0.1% |
| multiplos | 1 | 0.1% |

| Value | Count | Frequency (%) |
| --- | --- | --- |
| pul | 435 | 82.9% |
| pleural | 43 | 8.2% |
| ganglionar | 13 | 2.5% |
| periferica | 13 | 2.5% |
| oftalmica | 6 | 1.1% |
| pele | 5 | 1.0% |
| outras | 3 | 0.6% |
| ossea | 2 | 0.4% |
| miliar | 2 | 0.4% |
| vias | 1 | 0.2% |
| Other values (2) | 2 | 0.4% |

| Value | Count | Frequency (%) |
| --- | --- | --- |
| pul | 134 | 76.1% |
| pleural | 15 | 8.5% |
| ganglionar | 6 | 3.4% |
| periferica | 6 | 3.4% |
| meningea | 5 | 2.8% |
| miliar | 3 | 1.7% |
| outras | 2 | 1.1% |
| multiplos | 2 | 1.1% |
| orgaos | 2 | 1.1% |
| oftalmica | 1 | 0.6% |

- Characters
- Categories
- Scripts
- Blocks

#### Most occurring characters

| Value | Count | Frequency (%) |
| --- | --- | --- |
| l | 879 | 31.2% |
| P | 850 | 30.2% |
| u | 847 | 30.1% |
| a | 51 | 1.8% |
| r | 46 | 1.6% |
| e | 44 | 1.6% |
| i | 22 | 0.8% |
| n | 17 | 0.6% |
| g | 9 | 0.3% |
|  | 7 | 0.2% |
| Other values (11) | 41 | 1.5% |

| Value | Count | Frequency (%) |
| --- | --- | --- |
| l | 548 | 27.2% |
| P | 496 | 24.6% |
| u | 481 | 23.9% |
| a | 105 | 5.2% |
| r | 89 | 4.4% |
| e | 82 | 4.1% |
| i | 53 | 2.6% |
| n | 28 | 1.4% |
| f | 19 | 0.9% |
| c | 19 | 0.9% |
| Other values (11) | 94 | 4.7% |

| Value | Count | Frequency (%) |
| --- | --- | --- |
| l | 178 | 23.9% |
| P | 155 | 20.8% |
| u | 153 | 20.6% |
| a | 47 | 6.3% |
| r | 40 | 5.4% |
| e | 37 | 5.0% |
| i | 32 | 4.3% |
| n | 22 | 3.0% |
| g | 13 | 1.7% |
| M | 10 | 1.3% |
| Other values (10) | 57 | 7.7% |

#### Most occurring categories

| Value | Count | Frequency (%) |
| --- | --- | --- |
| Lowercase Letter | 1942 | 69.0% |
| Uppercase Letter | 864 | 30.7% |
| Space Separator | 7 | 0.2% |

| Value | Count | Frequency (%) |
| --- | --- | --- |
| Lowercase Letter | 1475 | 73.2% |
| Uppercase Letter | 525 | 26.1% |
| Space Separator | 14 | 0.7% |

| Value | Count | Frequency (%) |
| --- | --- | --- |
| Lowercase Letter | 560 | 75.3% |
| Uppercase Letter | 176 | 23.7% |
| Space Separator | 8 | 1.1% |

#### Most frequent character per category

##### *Lowercase Letter*

| Value | Count | Frequency (%) |
| --- | --- | --- |
| l | 879 | 45.3% |
| u | 847 | 43.6% |
| a | 51 | 2.6% |
| r | 46 | 2.4% |
| e | 44 | 2.3% |
| i | 22 | 1.1% |
| n | 17 | 0.9% |
| g | 9 | 0.5% |
| s | 7 | 0.4% |
| o | 7 | 0.4% |
| Other values (4) | 13 | 0.7% |

| Value | Count | Frequency (%) |
| --- | --- | --- |
| l | 548 | 37.2% |
| u | 481 | 32.6% |
| a | 105 | 7.1% |
| r | 89 | 6.0% |
| e | 82 | 5.6% |
| i | 53 | 3.6% |
| n | 28 | 1.9% |
| f | 19 | 1.3% |
| c | 19 | 1.3% |
| o | 13 | 0.9% |
| Other values (4) | 38 | 2.6% |

| Value | Count | Frequency (%) |
| --- | --- | --- |
| l | 178 | 31.8% |
| u | 153 | 27.3% |
| a | 47 | 8.4% |
| r | 40 | 7.1% |
| e | 37 | 6.6% |
| i | 32 | 5.7% |
| n | 22 | 3.9% |
| g | 13 | 2.3% |
| o | 10 | 1.8% |
| f | 7 | 1.2% |
| Other values (5) | 21 | 3.8% |

##### *Uppercase Letter*

| Value | Count | Frequency (%) |
| --- | --- | --- |
| P | 850 | 98.4% |
| G | 5 | 0.6% |
| M | 4 | 0.5% |
| O | 3 | 0.3% |
| V | 1 | 0.1% |
| U | 1 | 0.1% |

| Value | Count | Frequency (%) |
| --- | --- | --- |
| P | 496 | 94.5% |
| G | 14 | 2.7% |
| O | 11 | 2.1% |
| M | 2 | 0.4% |
| V | 1 | 0.2% |
| U | 1 | 0.2% |

| Value | Count | Frequency (%) |
| --- | --- | --- |
| P | 155 | 88.1% |
| M | 10 | 5.7% |
| G | 6 | 3.4% |
| O | 5 | 2.8% |

##### *Space Separator*

| Value | Count | Frequency (%) |
| --- | --- | --- |
|  | 7 | 100.0% |

| Value | Count | Frequency (%) |
| --- | --- | --- |
|  | 14 | 100.0% |

| Value | Count | Frequency (%) |
| --- | --- | --- |
|  | 8 | 100.0% |

#### Most occurring scripts

| Value | Count | Frequency (%) |
| --- | --- | --- |
| Latin | 2806 | 99.8% |
| Common | 7 | 0.2% |

| Value | Count | Frequency (%) |
| --- | --- | --- |
| Latin | 2000 | 99.3% |
| Common | 14 | 0.7% |

| Value | Count | Frequency (%) |
| --- | --- | --- |
| Latin | 736 | 98.9% |
| Common | 8 | 1.1% |

#### Most frequent character per script

##### *Latin*

| Value | Count | Frequency (%) |
| --- | --- | --- |
| l | 879 | 31.3% |
| P | 850 | 30.3% |
| u | 847 | 30.2% |
| a | 51 | 1.8% |
| r | 46 | 1.6% |
| e | 44 | 1.6% |
| i | 22 | 0.8% |
| n | 17 | 0.6% |
| g | 9 | 0.3% |
| s | 7 | 0.2% |
| Other values (10) | 34 | 1.2% |

| Value | Count | Frequency (%) |
| --- | --- | --- |
| l | 548 | 27.4% |
| P | 496 | 24.8% |
| u | 481 | 24.1% |
| a | 105 | 5.2% |
| r | 89 | 4.5% |
| e | 82 | 4.1% |
| i | 53 | 2.6% |
| n | 28 | 1.4% |
| f | 19 | 0.9% |
| c | 19 | 0.9% |
| Other values (10) | 80 | 4.0% |

| Value | Count | Frequency (%) |
| --- | --- | --- |
| l | 178 | 24.2% |
| P | 155 | 21.1% |
| u | 153 | 20.8% |
| a | 47 | 6.4% |
| r | 40 | 5.4% |
| e | 37 | 5.0% |
| i | 32 | 4.3% |
| n | 22 | 3.0% |
| g | 13 | 1.8% |
| M | 10 | 1.4% |
| Other values (9) | 49 | 6.7% |

##### *Common*

| Value | Count | Frequency (%) |
| --- | --- | --- |
|  | 7 | 100.0% |

| Value | Count | Frequency (%) |
| --- | --- | --- |
|  | 14 | 100.0% |

| Value | Count | Frequency (%) |
| --- | --- | --- |
|  | 8 | 100.0% |

#### Most occurring blocks

| Value | Count | Frequency (%) |
| --- | --- | --- |
| ASCII | 2813 | 100.0% |

| Value | Count | Frequency (%) |
| --- | --- | --- |
| ASCII | 2014 | 100.0% |

| Value | Count | Frequency (%) |
| --- | --- | --- |
| ASCII | 744 | 100.0% |

#### Most frequent character per block

##### *ASCII*

| Value | Count | Frequency (%) |
| --- | --- | --- |
| l | 879 | 31.2% |
| P | 850 | 30.2% |
| u | 847 | 30.1% |
| a | 51 | 1.8% |
| r | 46 | 1.6% |
| e | 44 | 1.6% |
| i | 22 | 0.8% |
| n | 17 | 0.6% |
| g | 9 | 0.3% |
|  | 7 | 0.2% |
| Other values (11) | 41 | 1.5% |

| Value | Count | Frequency (%) |
| --- | --- | --- |
| l | 548 | 27.2% |
| P | 496 | 24.6% |
| u | 481 | 23.9% |
| a | 105 | 5.2% |
| r | 89 | 4.4% |
| e | 82 | 4.1% |
| i | 53 | 2.6% |
| n | 28 | 1.4% |
| f | 19 | 0.9% |
| c | 19 | 0.9% |
| Other values (11) | 94 | 4.7% |

| Value | Count | Frequency (%) |
| --- | --- | --- |
| l | 178 | 23.9% |
| P | 155 | 20.8% |
| u | 153 | 20.6% |
| a | 47 | 6.3% |
| r | 40 | 5.4% |
| e | 37 | 5.0% |
| i | 32 | 4.3% |
| n | 22 | 3.0% |
| g | 13 | 1.7% |
| M | 10 | 1.3% |
| Other values (10) | 57 | 7.7% |

classif  
Categorical

|
|  |
|
|  |

|  | Cluster 1 | Cluster 2 | Cluster 3 |
| --- | --- | --- | --- |
| Distinct | 4 | 3 | 4 |
| Distinct (%) | 0.5% | 0.6% | 2.4% |
| Missing | 0 | 0 | 0 |
| Missing (%) | 0.0% | 0.0% | 0.0% |
| Memory size | 13.4 KiB | 8.0 KiB | 2.6 KiB |

|  |  |
| --- | --- |
| Pul | 785 |
| Ext | 38 |
| P+E | 33 |
| Dissem | 1 |

|  |  |
| --- | --- |
| Pul | 428 |
| Ext | 76 |
| P+E | 7 |

|  |  |
| --- | --- |
| Pul | 68 |
| P+E | 66 |
| Ext | 32 |
| Dissem | 2 |

More details

- Overview
- Categories
- Words
- Characters

Length

|  | Cluster 1 | Cluster 2 | Cluster 3 |
| --- | --- | --- | --- |
| Max length | 6 | 3 | 6 |
| Median length | 3 | 3 | 3 |
| Mean length | 3.0035006 | 3 | 3.0357143 |
| Min length | 3 | 3 | 3 |

Characters and Unicode

|  | Cluster 1 | Cluster 2 | Cluster 3 |
| --- | --- | --- | --- |
| Total characters | 2574 | 1533 | 510 |
| Distinct characters | 12 | 7 | 12 |
| Distinct categories | 3 | 3 | 3 ? |
| Distinct scripts | 2 | 2 | 2 ? |
| Distinct blocks | 1 | 1 | 1 ? |

The Unicode Standard assigns character properties to each code point, which can be used to analyse textual variables.

Unique

|  | Cluster 1 | Cluster 2 | Cluster 3 |
| --- | --- | --- | --- |
| Unique | 1 | 0 | 0 ? |
| Unique (%) | 0.1% | 0.0% | 0.0% |

Sample

|  | Cluster 1 | Cluster 2 | Cluster 3 |
| --- | --- | --- | --- |
| 1st row | Pul | Ext | P+E |
| 2nd row | P+E | Pul | P+E |
| 3rd row | P+E | Ext | Pul |
| 4th row | Pul | Pul | Ext |
| 5th row | Pul | Pul | P+E |

#### Common Values

| Value | Count | Frequency (%) |
| --- | --- | --- |
| Pul | 785 | 91.6% |
| Ext | 38 | 4.4% |
| P+E | 33 | 3.9% |
| Dissem | 1 | 0.1% |

| Value | Count | Frequency (%) |
| --- | --- | --- |
| Pul | 428 | 83.8% |
| Ext | 76 | 14.9% |
| P+E | 7 | 1.4% |

| Value | Count | Frequency (%) |
| --- | --- | --- |
| Pul | 68 | 40.5% |
| P+E | 66 | 39.3% |
| Ext | 32 | 19.0% |
| Dissem | 2 | 1.2% |

#### Length

xml version="1.0" encoding="utf-8" standalone="no"?2023-08-25T13:07:43.572407image/svg+xmlMatplotlib v3.6.0, https://matplotlib.org/ 

Histogram of lengths of the category

#### Common Values (Plot)

#### Cluster 1

xml version="1.0" encoding="utf-8" standalone="no"?2023-08-25T13:07:43.747164image/svg+xmlMatplotlib v3.6.0, https://matplotlib.org/

#### Cluster 2

xml version="1.0" encoding="utf-8" standalone="no"?2023-08-25T13:07:43.885697image/svg+xmlMatplotlib v3.6.0, https://matplotlib.org/

#### Cluster 3

xml version="1.0" encoding="utf-8" standalone="no"?2023-08-25T13:07:44.036200image/svg+xmlMatplotlib v3.6.0, https://matplotlib.org/

| Value | Count | Frequency (%) |
| --- | --- | --- |
| pul | 785 | 91.6% |
| ext | 38 | 4.4% |
| p+e | 33 | 3.9% |
| dissem | 1 | 0.1% |

| Value | Count | Frequency (%) |
| --- | --- | --- |
| pul | 428 | 83.8% |
| ext | 76 | 14.9% |
| p+e | 7 | 1.4% |

| Value | Count | Frequency (%) |
| --- | --- | --- |
| pul | 68 | 40.5% |
| p+e | 66 | 39.3% |
| ext | 32 | 19.0% |
| dissem | 2 | 1.2% |

- Characters
- Categories
- Scripts
- Blocks

#### Most occurring characters

| Value | Count | Frequency (%) |
| --- | --- | --- |
| P | 818 | 31.8% |
| u | 785 | 30.5% |
| l | 785 | 30.5% |
| E | 71 | 2.8% |
| x | 38 | 1.5% |
| t | 38 | 1.5% |
| + | 33 | 1.3% |
| s | 2 | 0.1% |
| D | 1 | < 0.1% |
| i | 1 | < 0.1% |
| Other values (2) | 2 | 0.1% |

| Value | Count | Frequency (%) |
| --- | --- | --- |
| P | 435 | 28.4% |
| u | 428 | 27.9% |
| l | 428 | 27.9% |
| E | 83 | 5.4% |
| x | 76 | 5.0% |
| t | 76 | 5.0% |
| + | 7 | 0.5% |

| Value | Count | Frequency (%) |
| --- | --- | --- |
| P | 134 | 26.3% |
| E | 98 | 19.2% |
| u | 68 | 13.3% |
| l | 68 | 13.3% |
| + | 66 | 12.9% |
| x | 32 | 6.3% |
| t | 32 | 6.3% |
| s | 4 | 0.8% |
| D | 2 | 0.4% |
| i | 2 | 0.4% |
| Other values (2) | 4 | 0.8% |

#### Most occurring categories

| Value | Count | Frequency (%) |
| --- | --- | --- |
| Lowercase Letter | 1651 | 64.1% |
| Uppercase Letter | 890 | 34.6% |
| Math Symbol | 33 | 1.3% |

| Value | Count | Frequency (%) |
| --- | --- | --- |
| Lowercase Letter | 1008 | 65.8% |
| Uppercase Letter | 518 | 33.8% |
| Math Symbol | 7 | 0.5% |

| Value | Count | Frequency (%) |
| --- | --- | --- |
| Uppercase Letter | 234 | 45.9% |
| Lowercase Letter | 210 | 41.2% |
| Math Symbol | 66 | 12.9% |

#### Most frequent character per category

##### *Uppercase Letter*

| Value | Count | Frequency (%) |
| --- | --- | --- |
| P | 818 | 91.9% |
| E | 71 | 8.0% |
| D | 1 | 0.1% |

| Value | Count | Frequency (%) |
| --- | --- | --- |
| P | 435 | 84.0% |
| E | 83 | 16.0% |

| Value | Count | Frequency (%) |
| --- | --- | --- |
| P | 134 | 57.3% |
| E | 98 | 41.9% |
| D | 2 | 0.9% |

##### *Lowercase Letter*

| Value | Count | Frequency (%) |
| --- | --- | --- |
| u | 785 | 47.5% |
| l | 785 | 47.5% |
| x | 38 | 2.3% |
| t | 38 | 2.3% |
| s | 2 | 0.1% |
| i | 1 | 0.1% |
| e | 1 | 0.1% |
| m | 1 | 0.1% |

| Value | Count | Frequency (%) |
| --- | --- | --- |
| u | 428 | 42.5% |
| l | 428 | 42.5% |
| x | 76 | 7.5% |
| t | 76 | 7.5% |

| Value | Count | Frequency (%) |
| --- | --- | --- |
| u | 68 | 32.4% |
| l | 68 | 32.4% |
| x | 32 | 15.2% |
| t | 32 | 15.2% |
| s | 4 | 1.9% |
| i | 2 | 1.0% |
| e | 2 | 1.0% |
| m | 2 | 1.0% |

##### *Math Symbol*

| Value | Count | Frequency (%) |
| --- | --- | --- |
| + | 33 | 100.0% |

| Value | Count | Frequency (%) |
| --- | --- | --- |
| + | 7 | 100.0% |

| Value | Count | Frequency (%) |
| --- | --- | --- |
| + | 66 | 100.0% |

#### Most occurring scripts

| Value | Count | Frequency (%) |
| --- | --- | --- |
| Latin | 2541 | 98.7% |
| Common | 33 | 1.3% |

| Value | Count | Frequency (%) |
| --- | --- | --- |
| Latin | 1526 | 99.5% |
| Common | 7 | 0.5% |

| Value | Count | Frequency (%) |
| --- | --- | --- |
| Latin | 444 | 87.1% |
| Common | 66 | 12.9% |

#### Most frequent character per script

##### *Latin*

| Value | Count | Frequency (%) |
| --- | --- | --- |
| P | 818 | 32.2% |
| u | 785 | 30.9% |
| l | 785 | 30.9% |
| E | 71 | 2.8% |
| x | 38 | 1.5% |
| t | 38 | 1.5% |
| s | 2 | 0.1% |
| D | 1 | < 0.1% |
| i | 1 | < 0.1% |
| e | 1 | < 0.1% |

| Value | Count | Frequency (%) |
| --- | --- | --- |
| P | 435 | 28.5% |
| u | 428 | 28.0% |
| l | 428 | 28.0% |
| E | 83 | 5.4% |
| x | 76 | 5.0% |
| t | 76 | 5.0% |

| Value | Count | Frequency (%) |
| --- | --- | --- |
| P | 134 | 30.2% |
| E | 98 | 22.1% |
| u | 68 | 15.3% |
| l | 68 | 15.3% |
| x | 32 | 7.2% |
| t | 32 | 7.2% |
| s | 4 | 0.9% |
| D | 2 | 0.5% |
| i | 2 | 0.5% |
| e | 2 | 0.5% |

##### *Common*

| Value | Count | Frequency (%) |
| --- | --- | --- |
| + | 33 | 100.0% |

| Value | Count | Frequency (%) |
| --- | --- | --- |
| + | 7 | 100.0% |

| Value | Count | Frequency (%) |
| --- | --- | --- |
| + | 66 | 100.0% |

#### Most occurring blocks

| Value | Count | Frequency (%) |
| --- | --- | --- |
| ASCII | 2574 | 100.0% |

| Value | Count | Frequency (%) |
| --- | --- | --- |
| ASCII | 1533 | 100.0% |

| Value | Count | Frequency (%) |
| --- | --- | --- |
| ASCII | 510 | 100.0% |

#### Most frequent character per block

##### *ASCII*

| Value | Count | Frequency (%) |
| --- | --- | --- |
| P | 818 | 31.8% |
| u | 785 | 30.5% |
| l | 785 | 30.5% |
| E | 71 | 2.8% |
| x | 38 | 1.5% |
| t | 38 | 1.5% |
| + | 33 | 1.3% |
| s | 2 | 0.1% |
| D | 1 | < 0.1% |
| i | 1 | < 0.1% |
| Other values (2) | 2 | 0.1% |

| Value | Count | Frequency (%) |
| --- | --- | --- |
| P | 435 | 28.4% |
| u | 428 | 27.9% |
| l | 428 | 27.9% |
| E | 83 | 5.4% |
| x | 76 | 5.0% |
| t | 76 | 5.0% |
| + | 7 | 0.5% |

| Value | Count | Frequency (%) |
| --- | --- | --- |
| P | 134 | 26.3% |
| E | 98 | 19.2% |
| u | 68 | 13.3% |
| l | 68 | 13.3% |
| + | 66 | 12.9% |
| x | 32 | 6.3% |
| t | 32 | 6.3% |
| s | 4 | 0.8% |
| D | 2 | 0.4% |
| i | 2 | 0.4% |
| Other values (2) | 4 | 0.8% |

descoberta  
Categorical

|
|  |
|
|  |

|  | Cluster 1 | Cluster 2 | Cluster 3 |
| --- | --- | --- | --- |
| Distinct | 6 | 6 | 6 |
| Distinct (%) | 0.7% | 1.2% | 3.6% |
| Missing | 0 | 0 | 0 |
| Missing (%) | 0.0% | 0.0% | 0.0% |
| Memory size | 13.4 KiB | 8.0 KiB | 2.6 KiB |

|  |  |
| --- | --- |
| Demanda Ambulatorial | 340 |
| Urgencia / Emergencia | 267 |
| Elucidacao Diagn. em Internacao | 168 |
| Busca Ativa na Comunidade | 34 |
| Investigacao de Contatos | 24 |

|  |  |
| --- | --- |
| Demanda Ambulatorial | 329 |
| Urgencia / Emergencia | 92 |
| Elucidacao Diagn. em Internacao | 61 |
| Investigacao de Contatos | 16 |
| Busca Ativa na Comunidade | 11 |

|  |  |
| --- | --- |
| Elucidacao Diagn. em Internacao | 115 |
| Demanda Ambulatorial | 32 |
| Urgencia / Emergencia | 17 |
| Busca Ativa em Instituicao | 2 |
| Busca Ativa na Comunidade | 1 |

More details

- Overview
- Categories
- Words
- Characters

Length

|  | Cluster 1 | Cluster 2 | Cluster 3 |
| --- | --- | --- | --- |
| Max length | 31 | 31 | 31 |
| Median length | 26 | 20 | 31 |
| Mean length | 22.946324 | 21.749511 | 27.755952 |
| Min length | 20 | 20 | 20 |

Characters and Unicode

|  | Cluster 1 | Cluster 2 | Cluster 3 |
| --- | --- | --- | --- |
| Total characters | 19665 | 11114 | 4663 |
| Distinct characters | 26 | 26 | 26 |
| Distinct categories | 4 | 4 | 4 ? |
| Distinct scripts | 2 | 2 | 2 ? |
| Distinct blocks | 1 | 1 | 1 ? |

The Unicode Standard assigns character properties to each code point, which can be used to analyse textual variables.

Unique

|  | Cluster 1 | Cluster 2 | Cluster 3 |
| --- | --- | --- | --- |
| Unique | 0 | 0 | 2 ? |
| Unique (%) | 0.0% | 0.0% | 1.2% |

Sample

|  | Cluster 1 | Cluster 2 | Cluster 3 |
| --- | --- | --- | --- |
| 1st row | Elucidacao Diagn. em Internacao | Demanda Ambulatorial | Elucidacao Diagn. em Internacao |
| 2nd row | Demanda Ambulatorial | Demanda Ambulatorial | Urgencia / Emergencia |
| 3rd row | Demanda Ambulatorial | Demanda Ambulatorial | Demanda Ambulatorial |
| 4th row | Demanda Ambulatorial | Demanda Ambulatorial | Elucidacao Diagn. em Internacao |
| 5th row | Demanda Ambulatorial | Demanda Ambulatorial | Elucidacao Diagn. em Internacao |

#### Common Values

| Value | Count | Frequency (%) |
| --- | --- | --- |
| Demanda Ambulatorial | 340 | 39.7% |
| Urgencia / Emergencia | 267 | 31.2% |
| Elucidacao Diagn. em Internacao | 168 | 19.6% |
| Busca Ativa na Comunidade | 34 | 4.0% |
| Investigacao de Contatos | 24 | 2.8% |
| Busca Ativa em Instituicao | 24 | 2.8% |

| Value | Count | Frequency (%) |
| --- | --- | --- |
| Demanda Ambulatorial | 329 | 64.4% |
| Urgencia / Emergencia | 92 | 18.0% |
| Elucidacao Diagn. em Internacao | 61 | 11.9% |
| Investigacao de Contatos | 16 | 3.1% |
| Busca Ativa na Comunidade | 11 | 2.2% |
| Busca Ativa em Instituicao | 2 | 0.4% |

| Value | Count | Frequency (%) |
| --- | --- | --- |
| Elucidacao Diagn. em Internacao | 115 | 68.5% |
| Demanda Ambulatorial | 32 | 19.0% |
| Urgencia / Emergencia | 17 | 10.1% |
| Busca Ativa em Instituicao | 2 | 1.2% |
| Busca Ativa na Comunidade | 1 | 0.6% |
| Investigacao de Contatos | 1 | 0.6% |

#### Length

xml version="1.0" encoding="utf-8" standalone="no"?2023-08-25T13:07:44.184253image/svg+xmlMatplotlib v3.6.0, https://matplotlib.org/ 

Histogram of lengths of the category

#### Common Values (Plot)

#### Cluster 1

xml version="1.0" encoding="utf-8" standalone="no"?2023-08-25T13:07:44.363346image/svg+xmlMatplotlib v3.6.0, https://matplotlib.org/

#### Cluster 2

xml version="1.0" encoding="utf-8" standalone="no"?2023-08-25T13:07:44.535900image/svg+xmlMatplotlib v3.6.0, https://matplotlib.org/

#### Cluster 3

xml version="1.0" encoding="utf-8" standalone="no"?2023-08-25T13:07:44.710381image/svg+xmlMatplotlib v3.6.0, https://matplotlib.org/

| Value | Count | Frequency (%) |
| --- | --- | --- |
| demanda | 340 | 13.8% |
| ambulatorial | 340 | 13.8% |
| urgencia | 267 | 10.9% |
|  | 267 | 10.9% |
| emergencia | 267 | 10.9% |
| em | 192 | 7.8% |
| internacao | 168 | 6.8% |
| diagn | 168 | 6.8% |
| elucidacao | 168 | 6.8% |
| busca | 58 | 2.4% |
| Other values (7) | 222 | 9.0% |

| Value | Count | Frequency (%) |
| --- | --- | --- |
| demanda | 329 | 25.7% |
| ambulatorial | 329 | 25.7% |
| urgencia | 92 | 7.2% |
|  | 92 | 7.2% |
| emergencia | 92 | 7.2% |
| em | 63 | 4.9% |
| internacao | 61 | 4.8% |
| diagn | 61 | 4.8% |
| elucidacao | 61 | 4.8% |
| investigacao | 16 | 1.3% |
| Other values (7) | 82 | 6.4% |

| Value | Count | Frequency (%) |
| --- | --- | --- |
| em | 117 | 19.8% |
| elucidacao | 115 | 19.5% |
| internacao | 115 | 19.5% |
| diagn | 115 | 19.5% |
| demanda | 32 | 5.4% |
| ambulatorial | 32 | 5.4% |
|  | 17 | 2.9% |
| emergencia | 17 | 2.9% |
| urgencia | 17 | 2.9% |
| busca | 3 | 0.5% |
| Other values (7) | 10 | 1.7% |

- Characters
- Categories
- Scripts
- Blocks

#### Most occurring characters

| Value | Count | Frequency (%) |
| --- | --- | --- |
| a | 3014 | 15.3% |
|  | 1600 | 8.1% |
| e | 1583 | 8.0% |
| n | 1518 | 7.7% |
| i | 1374 | 7.0% |
| m | 1173 | 6.0% |
| c | 1144 | 5.8% |
| r | 1042 | 5.3% |
| l | 848 | 4.3% |
| o | 806 | 4.1% |
| Other values (16) | 5563 | 28.3% |

| Value | Count | Frequency (%) |
| --- | --- | --- |
| a | 1903 | 17.1% |
| m | 824 | 7.4% |
| e | 772 | 6.9% |
|  | 767 | 6.9% |
| n | 752 | 6.8% |
| l | 719 | 6.5% |
| i | 679 | 6.1% |
| r | 574 | 5.2% |
| o | 512 | 4.6% |
| t | 455 | 4.1% |
| Other values (16) | 3157 | 28.4% |

| Value | Count | Frequency (%) |
| --- | --- | --- |
| a | 750 | 16.1% |
|  | 422 | 9.0% |
| n | 417 | 8.9% |
| c | 385 | 8.3% |
| e | 318 | 6.8% |
| i | 305 | 6.5% |
| o | 268 | 5.7% |
| m | 199 | 4.3% |
| r | 181 | 3.9% |
| l | 179 | 3.8% |
| Other values (16) | 1239 | 26.6% |

#### Most occurring categories

| Value | Count | Frequency (%) |
| --- | --- | --- |
| Lowercase Letter | 15690 | 79.8% |
| Uppercase Letter | 1940 | 9.9% |
| Space Separator | 1600 | 8.1% |
| Other Punctuation | 435 | 2.2% |

| Value | Count | Frequency (%) |
| --- | --- | --- |
| Lowercase Letter | 9098 | 81.9% |
| Uppercase Letter | 1096 | 9.9% |
| Space Separator | 767 | 6.9% |
| Other Punctuation | 153 | 1.4% |

| Value | Count | Frequency (%) |
| --- | --- | --- |
| Lowercase Letter | 3655 | 78.4% |
| Uppercase Letter | 454 | 9.7% |
| Space Separator | 422 | 9.0% |
| Other Punctuation | 132 | 2.8% |

#### Most frequent character per category

##### *Lowercase Letter*

| Value | Count | Frequency (%) |
| --- | --- | --- |
| a | 3014 | 19.2% |
| e | 1583 | 10.1% |
| n | 1518 | 9.7% |
| i | 1374 | 8.8% |
| m | 1173 | 7.5% |
| c | 1144 | 7.3% |
| r | 1042 | 6.6% |
| l | 848 | 5.4% |
| o | 806 | 5.1% |
| g | 726 | 4.6% |
| Other values (6) | 2462 | 15.7% |

| Value | Count | Frequency (%) |
| --- | --- | --- |
| a | 1903 | 20.9% |
| m | 824 | 9.1% |
| e | 772 | 8.5% |
| n | 752 | 8.3% |
| l | 719 | 7.9% |
| i | 679 | 7.5% |
| r | 574 | 6.3% |
| o | 512 | 5.6% |
| t | 455 | 5.0% |
| d | 428 | 4.7% |
| Other values (6) | 1480 | 16.3% |

| Value | Count | Frequency (%) |
| --- | --- | --- |
| a | 750 | 20.5% |
| n | 417 | 11.4% |
| c | 385 | 10.5% |
| e | 318 | 8.7% |
| i | 305 | 8.3% |
| o | 268 | 7.3% |
| m | 199 | 5.4% |
| r | 181 | 5.0% |
| l | 179 | 4.9% |
| t | 157 | 4.3% |
| Other values (6) | 496 | 13.6% |

##### *Space Separator*

| Value | Count | Frequency (%) |
| --- | --- | --- |
|  | 1600 | 100.0% |

| Value | Count | Frequency (%) |
| --- | --- | --- |
|  | 767 | 100.0% |

| Value | Count | Frequency (%) |
| --- | --- | --- |
|  | 422 | 100.0% |

##### *Uppercase Letter*

| Value | Count | Frequency (%) |
| --- | --- | --- |
| D | 508 | 26.2% |
| E | 435 | 22.4% |
| A | 398 | 20.5% |
| U | 267 | 13.8% |
| I | 216 | 11.1% |
| B | 58 | 3.0% |
| C | 58 | 3.0% |

| Value | Count | Frequency (%) |
| --- | --- | --- |
| D | 390 | 35.6% |
| A | 342 | 31.2% |
| E | 153 | 14.0% |
| U | 92 | 8.4% |
| I | 79 | 7.2% |
| C | 27 | 2.5% |
| B | 13 | 1.2% |

| Value | Count | Frequency (%) |
| --- | --- | --- |
| D | 147 | 32.4% |
| E | 132 | 29.1% |
| I | 118 | 26.0% |
| A | 35 | 7.7% |
| U | 17 | 3.7% |
| B | 3 | 0.7% |
| C | 2 | 0.4% |

##### *Other Punctuation*

| Value | Count | Frequency (%) |
| --- | --- | --- |
| / | 267 | 61.4% |
| . | 168 | 38.6% |

| Value | Count | Frequency (%) |
| --- | --- | --- |
| / | 92 | 60.1% |
| . | 61 | 39.9% |

| Value | Count | Frequency (%) |
| --- | --- | --- |
| . | 115 | 87.1% |
| / | 17 | 12.9% |

#### Most occurring scripts

| Value | Count | Frequency (%) |
| --- | --- | --- |
| Latin | 17630 | 89.7% |
| Common | 2035 | 10.3% |

| Value | Count | Frequency (%) |
| --- | --- | --- |
| Latin | 10194 | 91.7% |
| Common | 920 | 8.3% |

| Value | Count | Frequency (%) |
| --- | --- | --- |
| Latin | 4109 | 88.1% |
| Common | 554 | 11.9% |

#### Most frequent character per script

##### *Latin*

| Value | Count | Frequency (%) |
| --- | --- | --- |
| a | 3014 | 17.1% |
| e | 1583 | 9.0% |
| n | 1518 | 8.6% |
| i | 1374 | 7.8% |
| m | 1173 | 6.7% |
| c | 1144 | 6.5% |
| r | 1042 | 5.9% |
| l | 848 | 4.8% |
| o | 806 | 4.6% |
| g | 726 | 4.1% |
| Other values (13) | 4402 | 25.0% |

| Value | Count | Frequency (%) |
| --- | --- | --- |
| a | 1903 | 18.7% |
| m | 824 | 8.1% |
| e | 772 | 7.6% |
| n | 752 | 7.4% |
| l | 719 | 7.1% |
| i | 679 | 6.7% |
| r | 574 | 5.6% |
| o | 512 | 5.0% |
| t | 455 | 4.5% |
| d | 428 | 4.2% |
| Other values (13) | 2576 | 25.3% |

| Value | Count | Frequency (%) |
| --- | --- | --- |
| a | 750 | 18.3% |
| n | 417 | 10.1% |
| c | 385 | 9.4% |
| e | 318 | 7.7% |
| i | 305 | 7.4% |
| o | 268 | 6.5% |
| m | 199 | 4.8% |
| r | 181 | 4.4% |
| l | 179 | 4.4% |
| t | 157 | 3.8% |
| Other values (13) | 950 | 23.1% |

##### *Common*

| Value | Count | Frequency (%) |
| --- | --- | --- |
|  | 1600 | 78.6% |
| / | 267 | 13.1% |
| . | 168 | 8.3% |

| Value | Count | Frequency (%) |
| --- | --- | --- |
|  | 767 | 83.4% |
| / | 92 | 10.0% |
| . | 61 | 6.6% |

| Value | Count | Frequency (%) |
| --- | --- | --- |
|  | 422 | 76.2% |
| . | 115 | 20.8% |
| / | 17 | 3.1% |

#### Most occurring blocks

| Value | Count | Frequency (%) |
| --- | --- | --- |
| ASCII | 19665 | 100.0% |

| Value | Count | Frequency (%) |
| --- | --- | --- |
| ASCII | 11114 | 100.0% |

| Value | Count | Frequency (%) |
| --- | --- | --- |
| ASCII | 4663 | 100.0% |

#### Most frequent character per block

##### *ASCII*

| Value | Count | Frequency (%) |
| --- | --- | --- |
| a | 3014 | 15.3% |
|  | 1600 | 8.1% |
| e | 1583 | 8.0% |
| n | 1518 | 7.7% |
| i | 1374 | 7.0% |
| m | 1173 | 6.0% |
| c | 1144 | 5.8% |
| r | 1042 | 5.3% |
| l | 848 | 4.3% |
| o | 806 | 4.1% |
| Other values (16) | 5563 | 28.3% |

| Value | Count | Frequency (%) |
| --- | --- | --- |
| a | 1903 | 17.1% |
| m | 824 | 7.4% |
| e | 772 | 6.9% |
|  | 767 | 6.9% |
| n | 752 | 6.8% |
| l | 719 | 6.5% |
| i | 679 | 6.1% |
| r | 574 | 5.2% |
| o | 512 | 4.6% |
| t | 455 | 4.1% |
| Other values (16) | 3157 | 28.4% |

| Value | Count | Frequency (%) |
| --- | --- | --- |
| a | 750 | 16.1% |
|  | 422 | 9.0% |
| n | 417 | 8.9% |
| c | 385 | 8.3% |
| e | 318 | 6.8% |
| i | 305 | 6.5% |
| o | 268 | 5.7% |
| m | 199 | 4.3% |
| r | 181 | 3.9% |
| l | 179 | 3.8% |
| Other values (16) | 1239 | 26.6% |

bac  
Categorical

|
|  |
|
|  |

|  | Cluster 1 | Cluster 2 | Cluster 3 |
| --- | --- | --- | --- |
| Distinct | 3 | 3 | 3 |
| Distinct (%) | 0.4% | 0.6% | 1.8% |
| Missing | 0 | 0 | 0 |
| Missing (%) | 0.0% | 0.0% | 0.0% |
| Memory size | 13.4 KiB | 8.0 KiB | 2.6 KiB |

|  |  |
| --- | --- |
| Pos | 580 |
| Neg | 150 |
| N/realiz | 127 |

|  |  |
| --- | --- |
| Pos | 303 |
| Neg | 110 |
| N/realiz | 98 |

|  |  |
| --- | --- |
| Neg | 92 |
| Pos | 42 |
| N/realiz | 34 |

More details

- Overview
- Categories
- Words
- Characters

Length

|  | Cluster 1 | Cluster 2 | Cluster 3 |
| --- | --- | --- | --- |
| Max length | 8 | 8 | 8 |
| Median length | 3 | 3 | 3 |
| Mean length | 3.7409568 | 3.9589041 | 4.0119048 |
| Min length | 3 | 3 | 3 |

Characters and Unicode

|  | Cluster 1 | Cluster 2 | Cluster 3 |
| --- | --- | --- | --- |
| Total characters | 3206 | 2023 | 674 |
| Distinct characters | 12 | 12 | 12 |
| Distinct categories | 3 | 3 | 3 ? |
| Distinct scripts | 2 | 2 | 2 ? |
| Distinct blocks | 1 | 1 | 1 ? |

The Unicode Standard assigns character properties to each code point, which can be used to analyse textual variables.

Unique

|  | Cluster 1 | Cluster 2 | Cluster 3 |
| --- | --- | --- | --- |
| Unique | 0 | 0 | 0 ? |
| Unique (%) | 0.0% | 0.0% | 0.0% |

Sample

|  | Cluster 1 | Cluster 2 | Cluster 3 |
| --- | --- | --- | --- |
| 1st row | Pos | Neg | N/realiz |
| 2nd row | Pos | Pos | Pos |
| 3rd row | Pos | N/realiz | Neg |
| 4th row | Pos | Pos | Neg |
| 5th row | Neg | Neg | N/realiz |

#### Common Values

| Value | Count | Frequency (%) |
| --- | --- | --- |
| Pos | 580 | 67.7% |
| Neg | 150 | 17.5% |
| N/realiz | 127 | 14.8% |

| Value | Count | Frequency (%) |
| --- | --- | --- |
| Pos | 303 | 59.3% |
| Neg | 110 | 21.5% |
| N/realiz | 98 | 19.2% |

| Value | Count | Frequency (%) |
| --- | --- | --- |
| Neg | 92 | 54.8% |
| Pos | 42 | 25.0% |
| N/realiz | 34 | 20.2% |

#### Length

xml version="1.0" encoding="utf-8" standalone="no"?2023-08-25T13:07:44.869025image/svg+xmlMatplotlib v3.6.0, https://matplotlib.org/ 

Histogram of lengths of the category

#### Common Values (Plot)

#### Cluster 1

xml version="1.0" encoding="utf-8" standalone="no"?2023-08-25T13:07:45.027720image/svg+xmlMatplotlib v3.6.0, https://matplotlib.org/

#### Cluster 2

xml version="1.0" encoding="utf-8" standalone="no"?2023-08-25T13:07:45.162129image/svg+xmlMatplotlib v3.6.0, https://matplotlib.org/

#### Cluster 3

xml version="1.0" encoding="utf-8" standalone="no"?2023-08-25T13:07:45.297994image/svg+xmlMatplotlib v3.6.0, https://matplotlib.org/

| Value | Count | Frequency (%) |
| --- | --- | --- |
| pos | 580 | 67.7% |
| neg | 150 | 17.5% |
| n/realiz | 127 | 14.8% |

| Value | Count | Frequency (%) |
| --- | --- | --- |
| pos | 303 | 59.3% |
| neg | 110 | 21.5% |
| n/realiz | 98 | 19.2% |

| Value | Count | Frequency (%) |
| --- | --- | --- |
| neg | 92 | 54.8% |
| pos | 42 | 25.0% |
| n/realiz | 34 | 20.2% |

- Characters
- Categories
- Scripts
- Blocks

#### Most occurring characters

| Value | Count | Frequency (%) |
| --- | --- | --- |
| P | 580 | 18.1% |
| o | 580 | 18.1% |
| s | 580 | 18.1% |
| N | 277 | 8.6% |
| e | 277 | 8.6% |
| g | 150 | 4.7% |
| / | 127 | 4.0% |
| r | 127 | 4.0% |
| a | 127 | 4.0% |
| l | 127 | 4.0% |
| Other values (2) | 254 | 7.9% |

| Value | Count | Frequency (%) |
| --- | --- | --- |
| P | 303 | 15.0% |
| o | 303 | 15.0% |
| s | 303 | 15.0% |
| N | 208 | 10.3% |
| e | 208 | 10.3% |
| g | 110 | 5.4% |
| / | 98 | 4.8% |
| r | 98 | 4.8% |
| a | 98 | 4.8% |
| l | 98 | 4.8% |
| Other values (2) | 196 | 9.7% |

| Value | Count | Frequency (%) |
| --- | --- | --- |
| N | 126 | 18.7% |
| e | 126 | 18.7% |
| g | 92 | 13.6% |
| P | 42 | 6.2% |
| o | 42 | 6.2% |
| s | 42 | 6.2% |
| / | 34 | 5.0% |
| r | 34 | 5.0% |
| a | 34 | 5.0% |
| l | 34 | 5.0% |
| Other values (2) | 68 | 10.1% |

#### Most occurring categories

| Value | Count | Frequency (%) |
| --- | --- | --- |
| Lowercase Letter | 2222 | 69.3% |
| Uppercase Letter | 857 | 26.7% |
| Other Punctuation | 127 | 4.0% |

| Value | Count | Frequency (%) |
| --- | --- | --- |
| Lowercase Letter | 1414 | 69.9% |
| Uppercase Letter | 511 | 25.3% |
| Other Punctuation | 98 | 4.8% |

| Value | Count | Frequency (%) |
| --- | --- | --- |
| Lowercase Letter | 472 | 70.0% |
| Uppercase Letter | 168 | 24.9% |
| Other Punctuation | 34 | 5.0% |

#### Most frequent character per category

##### *Uppercase Letter*

| Value | Count | Frequency (%) |
| --- | --- | --- |
| P | 580 | 67.7% |
| N | 277 | 32.3% |

| Value | Count | Frequency (%) |
| --- | --- | --- |
| P | 303 | 59.3% |
| N | 208 | 40.7% |

| Value | Count | Frequency (%) |
| --- | --- | --- |
| N | 126 | 75.0% |
| P | 42 | 25.0% |

##### *Lowercase Letter*

| Value | Count | Frequency (%) |
| --- | --- | --- |
| o | 580 | 26.1% |
| s | 580 | 26.1% |
| e | 277 | 12.5% |
| g | 150 | 6.8% |
| r | 127 | 5.7% |
| a | 127 | 5.7% |
| l | 127 | 5.7% |
| i | 127 | 5.7% |
| z | 127 | 5.7% |

| Value | Count | Frequency (%) |
| --- | --- | --- |
| o | 303 | 21.4% |
| s | 303 | 21.4% |
| e | 208 | 14.7% |
| g | 110 | 7.8% |
| r | 98 | 6.9% |
| a | 98 | 6.9% |
| l | 98 | 6.9% |
| i | 98 | 6.9% |
| z | 98 | 6.9% |

| Value | Count | Frequency (%) |
| --- | --- | --- |
| e | 126 | 26.7% |
| g | 92 | 19.5% |
| o | 42 | 8.9% |
| s | 42 | 8.9% |
| r | 34 | 7.2% |
| a | 34 | 7.2% |
| l | 34 | 7.2% |
| i | 34 | 7.2% |
| z | 34 | 7.2% |

##### *Other Punctuation*

| Value | Count | Frequency (%) |
| --- | --- | --- |
| / | 127 | 100.0% |

| Value | Count | Frequency (%) |
| --- | --- | --- |
| / | 98 | 100.0% |

| Value | Count | Frequency (%) |
| --- | --- | --- |
| / | 34 | 100.0% |

#### Most occurring scripts

| Value | Count | Frequency (%) |
| --- | --- | --- |
| Latin | 3079 | 96.0% |
| Common | 127 | 4.0% |

| Value | Count | Frequency (%) |
| --- | --- | --- |
| Latin | 1925 | 95.2% |
| Common | 98 | 4.8% |

| Value | Count | Frequency (%) |
| --- | --- | --- |
| Latin | 640 | 95.0% |
| Common | 34 | 5.0% |

#### Most frequent character per script

##### *Latin*

| Value | Count | Frequency (%) |
| --- | --- | --- |
| P | 580 | 18.8% |
| o | 580 | 18.8% |
| s | 580 | 18.8% |
| N | 277 | 9.0% |
| e | 277 | 9.0% |
| g | 150 | 4.9% |
| r | 127 | 4.1% |
| a | 127 | 4.1% |
| l | 127 | 4.1% |
| i | 127 | 4.1% |

| Value | Count | Frequency (%) |
| --- | --- | --- |
| P | 303 | 15.7% |
| o | 303 | 15.7% |
| s | 303 | 15.7% |
| N | 208 | 10.8% |
| e | 208 | 10.8% |
| g | 110 | 5.7% |
| r | 98 | 5.1% |
| a | 98 | 5.1% |
| l | 98 | 5.1% |
| i | 98 | 5.1% |

| Value | Count | Frequency (%) |
| --- | --- | --- |
| N | 126 | 19.7% |
| e | 126 | 19.7% |
| g | 92 | 14.4% |
| P | 42 | 6.6% |
| o | 42 | 6.6% |
| s | 42 | 6.6% |
| r | 34 | 5.3% |
| a | 34 | 5.3% |
| l | 34 | 5.3% |
| i | 34 | 5.3% |

##### *Common*

| Value | Count | Frequency (%) |
| --- | --- | --- |
| / | 127 | 100.0% |

| Value | Count | Frequency (%) |
| --- | --- | --- |
| / | 98 | 100.0% |

| Value | Count | Frequency (%) |
| --- | --- | --- |
| / | 34 | 100.0% |

#### Most occurring blocks

| Value | Count | Frequency (%) |
| --- | --- | --- |
| ASCII | 3206 | 100.0% |

| Value | Count | Frequency (%) |
| --- | --- | --- |
| ASCII | 2023 | 100.0% |

| Value | Count | Frequency (%) |
| --- | --- | --- |
| ASCII | 674 | 100.0% |

#### Most frequent character per block

##### *ASCII*

| Value | Count | Frequency (%) |
| --- | --- | --- |
| P | 580 | 18.1% |
| o | 580 | 18.1% |
| s | 580 | 18.1% |
| N | 277 | 8.6% |
| e | 277 | 8.6% |
| g | 150 | 4.7% |
| / | 127 | 4.0% |
| r | 127 | 4.0% |
| a | 127 | 4.0% |
| l | 127 | 4.0% |
| Other values (2) | 254 | 7.9% |

| Value | Count | Frequency (%) |
| --- | --- | --- |
| P | 303 | 15.0% |
| o | 303 | 15.0% |
| s | 303 | 15.0% |
| N | 208 | 10.3% |
| e | 208 | 10.3% |
| g | 110 | 5.4% |
| / | 98 | 4.8% |
| r | 98 | 4.8% |
| a | 98 | 4.8% |
| l | 98 | 4.8% |
| Other values (2) | 196 | 9.7% |

| Value | Count | Frequency (%) |
| --- | --- | --- |
| N | 126 | 18.7% |
| e | 126 | 18.7% |
| g | 92 | 13.6% |
| P | 42 | 6.2% |
| o | 42 | 6.2% |
| s | 42 | 6.2% |
| / | 34 | 5.0% |
| r | 34 | 5.0% |
| a | 34 | 5.0% |
| l | 34 | 5.0% |
| Other values (2) | 68 | 10.1% |

BACOUTRO  
Categorical

|
|  |
|
|  |

|  | Cluster 1 | Cluster 2 | Cluster 3 |
| --- | --- | --- | --- |
| Distinct | 4 | 3 | 3 |
| Distinct (%) | 0.5% | 0.6% | 1.8% |
| Missing | 0 | 0 | 0 |
| Missing (%) | 0.0% | 0.0% | 0.0% |
| Memory size | 13.4 KiB | 8.0 KiB | 2.6 KiB |

|  |  |
| --- | --- |
| N/realiz | 790 |
| Neg | 40 |
| Pos | 26 |
| And | 1 |

|  |  |
| --- | --- |
| N/realiz | 476 |
| Neg | 23 |
| Pos | 12 |

|  |  |
| --- | --- |
| N/realiz | 99 |
| Neg | 40 |
| Pos | 29 |

More details

- Overview
- Categories
- Words
- Characters

Length

|  | Cluster 1 | Cluster 2 | Cluster 3 |
| --- | --- | --- | --- |
| Max length | 8 | 8 | 8 |
| Median length | 8 | 8 | 8 |
| Mean length | 7.6091015 | 7.6575342 | 5.9464286 |
| Min length | 3 | 3 | 3 |

Characters and Unicode

|  | Cluster 1 | Cluster 2 | Cluster 3 |
| --- | --- | --- | --- |
| Total characters | 6521 | 3913 | 999 |
| Distinct characters | 15 | 12 | 12 |
| Distinct categories | 3 | 3 | 3 ? |
| Distinct scripts | 2 | 2 | 2 ? |
| Distinct blocks | 1 | 1 | 1 ? |

The Unicode Standard assigns character properties to each code point, which can be used to analyse textual variables.

Unique

|  | Cluster 1 | Cluster 2 | Cluster 3 |
| --- | --- | --- | --- |
| Unique | 1 | 0 | 0 ? |
| Unique (%) | 0.1% | 0.0% | 0.0% |

Sample

|  | Cluster 1 | Cluster 2 | Cluster 3 |
| --- | --- | --- | --- |
| 1st row | N/realiz | N/realiz | Pos |
| 2nd row | N/realiz | N/realiz | N/realiz |
| 3rd row | N/realiz | N/realiz | N/realiz |
| 4th row | N/realiz | N/realiz | N/realiz |
| 5th row | N/realiz | N/realiz | Pos |

#### Common Values

| Value | Count | Frequency (%) |
| --- | --- | --- |
| N/realiz | 790 | 92.2% |
| Neg | 40 | 4.7% |
| Pos | 26 | 3.0% |
| And | 1 | 0.1% |

| Value | Count | Frequency (%) |
| --- | --- | --- |
| N/realiz | 476 | 93.2% |
| Neg | 23 | 4.5% |
| Pos | 12 | 2.3% |

| Value | Count | Frequency (%) |
| --- | --- | --- |
| N/realiz | 99 | 58.9% |
| Neg | 40 | 23.8% |
| Pos | 29 | 17.3% |

#### Length

xml version="1.0" encoding="utf-8" standalone="no"?2023-08-25T13:07:45.425755image/svg+xmlMatplotlib v3.6.0, https://matplotlib.org/ 

Histogram of lengths of the category

#### Common Values (Plot)

#### Cluster 1

xml version="1.0" encoding="utf-8" standalone="no"?2023-08-25T13:07:45.588745image/svg+xmlMatplotlib v3.6.0, https://matplotlib.org/

#### Cluster 2

xml version="1.0" encoding="utf-8" standalone="no"?2023-08-25T13:07:45.736080image/svg+xmlMatplotlib v3.6.0, https://matplotlib.org/

#### Cluster 3

xml version="1.0" encoding="utf-8" standalone="no"?2023-08-25T13:07:45.869520image/svg+xmlMatplotlib v3.6.0, https://matplotlib.org/

| Value | Count | Frequency (%) |
| --- | --- | --- |
| n/realiz | 790 | 92.2% |
| neg | 40 | 4.7% |
| pos | 26 | 3.0% |
| and | 1 | 0.1% |

| Value | Count | Frequency (%) |
| --- | --- | --- |
| n/realiz | 476 | 93.2% |
| neg | 23 | 4.5% |
| pos | 12 | 2.3% |

| Value | Count | Frequency (%) |
| --- | --- | --- |
| n/realiz | 99 | 58.9% |
| neg | 40 | 23.8% |
| pos | 29 | 17.3% |

- Characters
- Categories
- Scripts
- Blocks

#### Most occurring characters

| Value | Count | Frequency (%) |
| --- | --- | --- |
| N | 830 | 12.7% |
| e | 830 | 12.7% |
| / | 790 | 12.1% |
| r | 790 | 12.1% |
| a | 790 | 12.1% |
| l | 790 | 12.1% |
| i | 790 | 12.1% |
| z | 790 | 12.1% |
| g | 40 | 0.6% |
| P | 26 | 0.4% |
| Other values (5) | 55 | 0.8% |

| Value | Count | Frequency (%) |
| --- | --- | --- |
| N | 499 | 12.8% |
| e | 499 | 12.8% |
| / | 476 | 12.2% |
| r | 476 | 12.2% |
| a | 476 | 12.2% |
| l | 476 | 12.2% |
| i | 476 | 12.2% |
| z | 476 | 12.2% |
| g | 23 | 0.6% |
| P | 12 | 0.3% |
| Other values (2) | 24 | 0.6% |

| Value | Count | Frequency (%) |
| --- | --- | --- |
| N | 139 | 13.9% |
| e | 139 | 13.9% |
| / | 99 | 9.9% |
| r | 99 | 9.9% |
| a | 99 | 9.9% |
| l | 99 | 9.9% |
| i | 99 | 9.9% |
| z | 99 | 9.9% |
| g | 40 | 4.0% |
| P | 29 | 2.9% |
| Other values (2) | 58 | 5.8% |

#### Most occurring categories

| Value | Count | Frequency (%) |
| --- | --- | --- |
| Lowercase Letter | 4874 | 74.7% |
| Uppercase Letter | 857 | 13.1% |
| Other Punctuation | 790 | 12.1% |

| Value | Count | Frequency (%) |
| --- | --- | --- |
| Lowercase Letter | 2926 | 74.8% |
| Uppercase Letter | 511 | 13.1% |
| Other Punctuation | 476 | 12.2% |

| Value | Count | Frequency (%) |
| --- | --- | --- |
| Lowercase Letter | 732 | 73.3% |
| Uppercase Letter | 168 | 16.8% |
| Other Punctuation | 99 | 9.9% |

#### Most frequent character per category

##### *Uppercase Letter*

| Value | Count | Frequency (%) |
| --- | --- | --- |
| N | 830 | 96.8% |
| P | 26 | 3.0% |
| A | 1 | 0.1% |

| Value | Count | Frequency (%) |
| --- | --- | --- |
| N | 499 | 97.7% |
| P | 12 | 2.3% |

| Value | Count | Frequency (%) |
| --- | --- | --- |
| N | 139 | 82.7% |
| P | 29 | 17.3% |

##### *Lowercase Letter*

| Value | Count | Frequency (%) |
| --- | --- | --- |
| e | 830 | 17.0% |
| r | 790 | 16.2% |
| a | 790 | 16.2% |
| l | 790 | 16.2% |
| i | 790 | 16.2% |
| z | 790 | 16.2% |
| g | 40 | 0.8% |
| o | 26 | 0.5% |
| s | 26 | 0.5% |
| n | 1 | < 0.1% |

| Value | Count | Frequency (%) |
| --- | --- | --- |
| e | 499 | 17.1% |
| r | 476 | 16.3% |
| a | 476 | 16.3% |
| l | 476 | 16.3% |
| i | 476 | 16.3% |
| z | 476 | 16.3% |
| g | 23 | 0.8% |
| o | 12 | 0.4% |
| s | 12 | 0.4% |

| Value | Count | Frequency (%) |
| --- | --- | --- |
| e | 139 | 19.0% |
| r | 99 | 13.5% |
| a | 99 | 13.5% |
| l | 99 | 13.5% |
| i | 99 | 13.5% |
| z | 99 | 13.5% |
| g | 40 | 5.5% |
| o | 29 | 4.0% |
| s | 29 | 4.0% |

##### *Other Punctuation*

| Value | Count | Frequency (%) |
| --- | --- | --- |
| / | 790 | 100.0% |

| Value | Count | Frequency (%) |
| --- | --- | --- |
| / | 476 | 100.0% |

| Value | Count | Frequency (%) |
| --- | --- | --- |
| / | 99 | 100.0% |

#### Most occurring scripts

| Value | Count | Frequency (%) |
| --- | --- | --- |
| Latin | 5731 | 87.9% |
| Common | 790 | 12.1% |

| Value | Count | Frequency (%) |
| --- | --- | --- |
| Latin | 3437 | 87.8% |
| Common | 476 | 12.2% |

| Value | Count | Frequency (%) |
| --- | --- | --- |
| Latin | 900 | 90.1% |
| Common | 99 | 9.9% |

#### Most frequent character per script

##### *Latin*

| Value | Count | Frequency (%) |
| --- | --- | --- |
| N | 830 | 14.5% |
| e | 830 | 14.5% |
| r | 790 | 13.8% |
| a | 790 | 13.8% |
| l | 790 | 13.8% |
| i | 790 | 13.8% |
| z | 790 | 13.8% |
| g | 40 | 0.7% |
| P | 26 | 0.5% |
| o | 26 | 0.5% |
| Other values (4) | 29 | 0.5% |

| Value | Count | Frequency (%) |
| --- | --- | --- |
| N | 499 | 14.5% |
| e | 499 | 14.5% |
| r | 476 | 13.8% |
| a | 476 | 13.8% |
| l | 476 | 13.8% |
| i | 476 | 13.8% |
| z | 476 | 13.8% |
| g | 23 | 0.7% |
| P | 12 | 0.3% |
| o | 12 | 0.3% |

| Value | Count | Frequency (%) |
| --- | --- | --- |
| N | 139 | 15.4% |
| e | 139 | 15.4% |
| r | 99 | 11.0% |
| a | 99 | 11.0% |
| l | 99 | 11.0% |
| i | 99 | 11.0% |
| z | 99 | 11.0% |
| g | 40 | 4.4% |
| P | 29 | 3.2% |
| o | 29 | 3.2% |

##### *Common*

| Value | Count | Frequency (%) |
| --- | --- | --- |
| / | 790 | 100.0% |

| Value | Count | Frequency (%) |
| --- | --- | --- |
| / | 476 | 100.0% |

| Value | Count | Frequency (%) |
| --- | --- | --- |
| / | 99 | 100.0% |

#### Most occurring blocks

| Value | Count | Frequency (%) |
| --- | --- | --- |
| ASCII | 6521 | 100.0% |

| Value | Count | Frequency (%) |
| --- | --- | --- |
| ASCII | 3913 | 100.0% |

| Value | Count | Frequency (%) |
| --- | --- | --- |
| ASCII | 999 | 100.0% |

#### Most frequent character per block

##### *ASCII*

| Value | Count | Frequency (%) |
| --- | --- | --- |
| N | 830 | 12.7% |
| e | 830 | 12.7% |
| / | 790 | 12.1% |
| r | 790 | 12.1% |
| a | 790 | 12.1% |
| l | 790 | 12.1% |
| i | 790 | 12.1% |
| z | 790 | 12.1% |
| g | 40 | 0.6% |
| P | 26 | 0.4% |
| Other values (5) | 55 | 0.8% |

| Value | Count | Frequency (%) |
| --- | --- | --- |
| N | 499 | 12.8% |
| e | 499 | 12.8% |
| / | 476 | 12.2% |
| r | 476 | 12.2% |
| a | 476 | 12.2% |
| l | 476 | 12.2% |
| i | 476 | 12.2% |
| z | 476 | 12.2% |
| g | 23 | 0.6% |
| P | 12 | 0.3% |
| Other values (2) | 24 | 0.6% |

| Value | Count | Frequency (%) |
| --- | --- | --- |
| N | 139 | 13.9% |
| e | 139 | 13.9% |
| / | 99 | 9.9% |
| r | 99 | 9.9% |
| a | 99 | 9.9% |
| l | 99 | 9.9% |
| i | 99 | 9.9% |
| z | 99 | 9.9% |
| g | 40 | 4.0% |
| P | 29 | 2.9% |
| Other values (2) | 58 | 5.8% |

cultEsc  
Categorical

|
|  |
|
|  |

|  | Cluster 1 | Cluster 2 | Cluster 3 |
| --- | --- | --- | --- |
| Distinct | 4 | 4 | 3 |
| Distinct (%) | 0.5% | 0.8% | 1.8% |
| Missing | 0 | 0 | 0 |
| Missing (%) | 0.0% | 0.0% | 0.0% |
| Memory size | 13.4 KiB | 8.0 KiB | 2.6 KiB |

|  |  |
| --- | --- |
| Pos | 697 |
| N/realiz | 97 |
| Neg | 62 |
| And | 1 |

|  |  |
| --- | --- |
| N/realiz | 372 |
| Pos | 80 |
| Neg | 57 |
| And | 2 |

|  |  |
| --- | --- |
| Pos | 81 |
| N/realiz | 60 |
| Neg | 27 |

More details

- Overview
- Categories
- Words
- Characters

Length

|  | Cluster 1 | Cluster 2 | Cluster 3 |
| --- | --- | --- | --- |
| Max length | 8 | 8 | 8 |
| Median length | 3 | 8 | 3 |
| Mean length | 3.5659277 | 6.6399217 | 4.7857143 |
| Min length | 3 | 3 | 3 |

Characters and Unicode

|  | Cluster 1 | Cluster 2 | Cluster 3 |
| --- | --- | --- | --- |
| Total characters | 3056 | 3393 | 804 |
| Distinct characters | 15 | 15 | 12 |
| Distinct categories | 3 | 3 | 3 ? |
| Distinct scripts | 2 | 2 | 2 ? |
| Distinct blocks | 1 | 1 | 1 ? |

The Unicode Standard assigns character properties to each code point, which can be used to analyse textual variables.

Unique

|  | Cluster 1 | Cluster 2 | Cluster 3 |
| --- | --- | --- | --- |
| Unique | 1 | 0 | 0 ? |
| Unique (%) | 0.1% | 0.0% | 0.0% |

Sample

|  | Cluster 1 | Cluster 2 | Cluster 3 |
| --- | --- | --- | --- |
| 1st row | Pos | N/realiz | N/realiz |
| 2nd row | Pos | N/realiz | N/realiz |
| 3rd row | Pos | N/realiz | Neg |
| 4th row | Pos | Pos | Neg |
| 5th row | Pos | Neg | N/realiz |

#### Common Values

| Value | Count | Frequency (%) |
| --- | --- | --- |
| Pos | 697 | 81.3% |
| N/realiz | 97 | 11.3% |
| Neg | 62 | 7.2% |
| And | 1 | 0.1% |

| Value | Count | Frequency (%) |
| --- | --- | --- |
| N/realiz | 372 | 72.8% |
| Pos | 80 | 15.7% |
| Neg | 57 | 11.2% |
| And | 2 | 0.4% |

| Value | Count | Frequency (%) |
| --- | --- | --- |
| Pos | 81 | 48.2% |
| N/realiz | 60 | 35.7% |
| Neg | 27 | 16.1% |

#### Length

xml version="1.0" encoding="utf-8" standalone="no"?2023-08-25T13:07:46.023917image/svg+xmlMatplotlib v3.6.0, https://matplotlib.org/ 

Histogram of lengths of the category

#### Common Values (Plot)

#### Cluster 1

xml version="1.0" encoding="utf-8" standalone="no"?2023-08-25T13:07:46.192693image/svg+xmlMatplotlib v3.6.0, https://matplotlib.org/

#### Cluster 2

xml version="1.0" encoding="utf-8" standalone="no"?2023-08-25T13:07:46.348244image/svg+xmlMatplotlib v3.6.0, https://matplotlib.org/

#### Cluster 3

xml version="1.0" encoding="utf-8" standalone="no"?2023-08-25T13:07:46.491478image/svg+xmlMatplotlib v3.6.0, https://matplotlib.org/

| Value | Count | Frequency (%) |
| --- | --- | --- |
| pos | 697 | 81.3% |
| n/realiz | 97 | 11.3% |
| neg | 62 | 7.2% |
| and | 1 | 0.1% |

| Value | Count | Frequency (%) |
| --- | --- | --- |
| n/realiz | 372 | 72.8% |
| pos | 80 | 15.7% |
| neg | 57 | 11.2% |
| and | 2 | 0.4% |

| Value | Count | Frequency (%) |
| --- | --- | --- |
| pos | 81 | 48.2% |
| n/realiz | 60 | 35.7% |
| neg | 27 | 16.1% |

- Characters
- Categories
- Scripts
- Blocks

#### Most occurring characters

| Value | Count | Frequency (%) |
| --- | --- | --- |
| P | 697 | 22.8% |
| o | 697 | 22.8% |
| s | 697 | 22.8% |
| N | 159 | 5.2% |
| e | 159 | 5.2% |
| / | 97 | 3.2% |
| r | 97 | 3.2% |
| a | 97 | 3.2% |
| l | 97 | 3.2% |
| i | 97 | 3.2% |
| Other values (5) | 162 | 5.3% |

| Value | Count | Frequency (%) |
| --- | --- | --- |
| N | 429 | 12.6% |
| e | 429 | 12.6% |
| / | 372 | 11.0% |
| r | 372 | 11.0% |
| a | 372 | 11.0% |
| l | 372 | 11.0% |
| i | 372 | 11.0% |
| z | 372 | 11.0% |
| P | 80 | 2.4% |
| o | 80 | 2.4% |
| Other values (5) | 143 | 4.2% |

| Value | Count | Frequency (%) |
| --- | --- | --- |
| N | 87 | 10.8% |
| e | 87 | 10.8% |
| P | 81 | 10.1% |
| o | 81 | 10.1% |
| s | 81 | 10.1% |
| / | 60 | 7.5% |
| r | 60 | 7.5% |
| a | 60 | 7.5% |
| l | 60 | 7.5% |
| i | 60 | 7.5% |
| Other values (2) | 87 | 10.8% |

#### Most occurring categories

| Value | Count | Frequency (%) |
| --- | --- | --- |
| Lowercase Letter | 2102 | 68.8% |
| Uppercase Letter | 857 | 28.0% |
| Other Punctuation | 97 | 3.2% |

| Value | Count | Frequency (%) |
| --- | --- | --- |
| Lowercase Letter | 2510 | 74.0% |
| Uppercase Letter | 511 | 15.1% |
| Other Punctuation | 372 | 11.0% |

| Value | Count | Frequency (%) |
| --- | --- | --- |
| Lowercase Letter | 576 | 71.6% |
| Uppercase Letter | 168 | 20.9% |
| Other Punctuation | 60 | 7.5% |

#### Most frequent character per category

##### *Uppercase Letter*

| Value | Count | Frequency (%) |
| --- | --- | --- |
| P | 697 | 81.3% |
| N | 159 | 18.6% |
| A | 1 | 0.1% |

| Value | Count | Frequency (%) |
| --- | --- | --- |
| N | 429 | 84.0% |
| P | 80 | 15.7% |
| A | 2 | 0.4% |

| Value | Count | Frequency (%) |
| --- | --- | --- |
| N | 87 | 51.8% |
| P | 81 | 48.2% |

##### *Lowercase Letter*

| Value | Count | Frequency (%) |
| --- | --- | --- |
| o | 697 | 33.2% |
| s | 697 | 33.2% |
| e | 159 | 7.6% |
| r | 97 | 4.6% |
| a | 97 | 4.6% |
| l | 97 | 4.6% |
| i | 97 | 4.6% |
| z | 97 | 4.6% |
| g | 62 | 2.9% |
| n | 1 | < 0.1% |

| Value | Count | Frequency (%) |
| --- | --- | --- |
| e | 429 | 17.1% |
| r | 372 | 14.8% |
| a | 372 | 14.8% |
| l | 372 | 14.8% |
| i | 372 | 14.8% |
| z | 372 | 14.8% |
| o | 80 | 3.2% |
| s | 80 | 3.2% |
| g | 57 | 2.3% |
| n | 2 | 0.1% |

| Value | Count | Frequency (%) |
| --- | --- | --- |
| e | 87 | 15.1% |
| o | 81 | 14.1% |
| s | 81 | 14.1% |
| r | 60 | 10.4% |
| a | 60 | 10.4% |
| l | 60 | 10.4% |
| i | 60 | 10.4% |
| z | 60 | 10.4% |
| g | 27 | 4.7% |

##### *Other Punctuation*

| Value | Count | Frequency (%) |
| --- | --- | --- |
| / | 97 | 100.0% |

| Value | Count | Frequency (%) |
| --- | --- | --- |
| / | 372 | 100.0% |

| Value | Count | Frequency (%) |
| --- | --- | --- |
| / | 60 | 100.0% |

#### Most occurring scripts

| Value | Count | Frequency (%) |
| --- | --- | --- |
| Latin | 2959 | 96.8% |
| Common | 97 | 3.2% |

| Value | Count | Frequency (%) |
| --- | --- | --- |
| Latin | 3021 | 89.0% |
| Common | 372 | 11.0% |

| Value | Count | Frequency (%) |
| --- | --- | --- |
| Latin | 744 | 92.5% |
| Common | 60 | 7.5% |

#### Most frequent character per script

##### *Latin*

| Value | Count | Frequency (%) |
| --- | --- | --- |
| P | 697 | 23.6% |
| o | 697 | 23.6% |
| s | 697 | 23.6% |
| N | 159 | 5.4% |
| e | 159 | 5.4% |
| r | 97 | 3.3% |
| a | 97 | 3.3% |
| l | 97 | 3.3% |
| i | 97 | 3.3% |
| z | 97 | 3.3% |
| Other values (4) | 65 | 2.2% |

| Value | Count | Frequency (%) |
| --- | --- | --- |
| N | 429 | 14.2% |
| e | 429 | 14.2% |
| r | 372 | 12.3% |
| a | 372 | 12.3% |
| l | 372 | 12.3% |
| i | 372 | 12.3% |
| z | 372 | 12.3% |
| P | 80 | 2.6% |
| o | 80 | 2.6% |
| s | 80 | 2.6% |
| Other values (4) | 63 | 2.1% |

| Value | Count | Frequency (%) |
| --- | --- | --- |
| N | 87 | 11.7% |
| e | 87 | 11.7% |
| P | 81 | 10.9% |
| o | 81 | 10.9% |
| s | 81 | 10.9% |
| r | 60 | 8.1% |
| a | 60 | 8.1% |
| l | 60 | 8.1% |
| i | 60 | 8.1% |
| z | 60 | 8.1% |

##### *Common*

| Value | Count | Frequency (%) |
| --- | --- | --- |
| / | 97 | 100.0% |

| Value | Count | Frequency (%) |
| --- | --- | --- |
| / | 372 | 100.0% |

| Value | Count | Frequency (%) |
| --- | --- | --- |
| / | 60 | 100.0% |

#### Most occurring blocks

| Value | Count | Frequency (%) |
| --- | --- | --- |
| ASCII | 3056 | 100.0% |

| Value | Count | Frequency (%) |
| --- | --- | --- |
| ASCII | 3393 | 100.0% |

| Value | Count | Frequency (%) |
| --- | --- | --- |
| ASCII | 804 | 100.0% |

#### Most frequent character per block

##### *ASCII*

| Value | Count | Frequency (%) |
| --- | --- | --- |
| P | 697 | 22.8% |
| o | 697 | 22.8% |
| s | 697 | 22.8% |
| N | 159 | 5.2% |
| e | 159 | 5.2% |
| / | 97 | 3.2% |
| r | 97 | 3.2% |
| a | 97 | 3.2% |
| l | 97 | 3.2% |
| i | 97 | 3.2% |
| Other values (5) | 162 | 5.3% |

| Value | Count | Frequency (%) |
| --- | --- | --- |
| N | 429 | 12.6% |
| e | 429 | 12.6% |
| / | 372 | 11.0% |
| r | 372 | 11.0% |
| a | 372 | 11.0% |
| l | 372 | 11.0% |
| i | 372 | 11.0% |
| z | 372 | 11.0% |
| P | 80 | 2.4% |
| o | 80 | 2.4% |
| Other values (5) | 143 | 4.2% |

| Value | Count | Frequency (%) |
| --- | --- | --- |
| N | 87 | 10.8% |
| e | 87 | 10.8% |
| P | 81 | 10.1% |
| o | 81 | 10.1% |
| s | 81 | 10.1% |
| / | 60 | 7.5% |
| r | 60 | 7.5% |
| a | 60 | 7.5% |
| l | 60 | 7.5% |
| i | 60 | 7.5% |
| Other values (2) | 87 | 10.8% |

RX  
Categorical

|
|  |
|
|  |

|  | Cluster 1 | Cluster 2 | Cluster 3 |
| --- | --- | --- | --- |
| Distinct | 5 | 5 | 5 |
| Distinct (%) | 0.6% | 1.0% | 3.0% |
| Missing | 0 | 0 | 0 |
| Missing (%) | 0.0% | 0.0% | 0.0% |
| Memory size | 13.4 KiB | 8.0 KiB | 2.6 KiB |

|  |  |
| --- | --- |
| Susp TB | 517 |
| Susp c/cavid | 179 |
| N/realiz | 125 |
| Normal | 30 |
| Outra Patologia | 6 |

|  |  |
| --- | --- |
| Susp TB | 304 |
| Susp c/cavid | 109 |
| N/realiz | 62 |
| Normal | 29 |
| Outra Patologia | 7 |

|  |  |
| --- | --- |
| Susp TB | 120 |
| N/realiz | 21 |
| Normal | 20 |
| Outra Patologia | 4 |
| Susp c/cavid | 3 |

More details

- Overview
- Categories
- Words
- Characters

Length

|  | Cluster 1 | Cluster 2 | Cluster 3 |
| --- | --- | --- | --- |
| Max length | 15 | 15 | 15 |
| Median length | 7 | 7 | 7 |
| Mean length | 8.2112019 | 8.2407045 | 7.2857143 |
| Min length | 6 | 6 | 6 |

Characters and Unicode

|  | Cluster 1 | Cluster 2 | Cluster 3 |
| --- | --- | --- | --- |
| Total characters | 7037 | 4211 | 1224 |
| Distinct characters | 24 | 24 | 24 |
| Distinct categories | 4 | 4 | 4 ? |
| Distinct scripts | 2 | 2 | 2 ? |
| Distinct blocks | 1 | 1 | 1 ? |

The Unicode Standard assigns character properties to each code point, which can be used to analyse textual variables.

Unique

|  | Cluster 1 | Cluster 2 | Cluster 3 |
| --- | --- | --- | --- |
| Unique | 0 | 0 | 0 ? |
| Unique (%) | 0.0% | 0.0% | 0.0% |

Sample

|  | Cluster 1 | Cluster 2 | Cluster 3 |
| --- | --- | --- | --- |
| 1st row | Susp c/cavid | Susp TB | Susp TB |
| 2nd row | Susp TB | N/realiz | Susp TB |
| 3rd row | Susp TB | N/realiz | Susp c/cavid |
| 4th row | Susp TB | Susp TB | Susp TB |
| 5th row | Susp TB | Susp c/cavid | Susp TB |

#### Common Values

| Value | Count | Frequency (%) |
| --- | --- | --- |
| Susp TB | 517 | 60.3% |
| Susp c/cavid | 179 | 20.9% |
| N/realiz | 125 | 14.6% |
| Normal | 30 | 3.5% |
| Outra Patologia | 6 | 0.7% |

| Value | Count | Frequency (%) |
| --- | --- | --- |
| Susp TB | 304 | 59.5% |
| Susp c/cavid | 109 | 21.3% |
| N/realiz | 62 | 12.1% |
| Normal | 29 | 5.7% |
| Outra Patologia | 7 | 1.4% |

| Value | Count | Frequency (%) |
| --- | --- | --- |
| Susp TB | 120 | 71.4% |
| N/realiz | 21 | 12.5% |
| Normal | 20 | 11.9% |
| Outra Patologia | 4 | 2.4% |
| Susp c/cavid | 3 | 1.8% |

#### Length

xml version="1.0" encoding="utf-8" standalone="no"?2023-08-25T13:07:46.630690image/svg+xmlMatplotlib v3.6.0, https://matplotlib.org/ 

Histogram of lengths of the category

#### Common Values (Plot)

#### Cluster 1

xml version="1.0" encoding="utf-8" standalone="no"?2023-08-25T13:07:46.809987image/svg+xmlMatplotlib v3.6.0, https://matplotlib.org/

#### Cluster 2

xml version="1.0" encoding="utf-8" standalone="no"?2023-08-25T13:07:46.963906image/svg+xmlMatplotlib v3.6.0, https://matplotlib.org/

#### Cluster 3

xml version="1.0" encoding="utf-8" standalone="no"?2023-08-25T13:07:47.121193image/svg+xmlMatplotlib v3.6.0, https://matplotlib.org/

| Value | Count | Frequency (%) |
| --- | --- | --- |
| susp | 696 | 44.6% |
| tb | 517 | 33.2% |
| c/cavid | 179 | 11.5% |
| n/realiz | 125 | 8.0% |
| normal | 30 | 1.9% |
| outra | 6 | 0.4% |
| patologia | 6 | 0.4% |

| Value | Count | Frequency (%) |
| --- | --- | --- |
| susp | 413 | 44.4% |
| tb | 304 | 32.7% |
| c/cavid | 109 | 11.7% |
| n/realiz | 62 | 6.7% |
| normal | 29 | 3.1% |
| outra | 7 | 0.8% |
| patologia | 7 | 0.8% |

| Value | Count | Frequency (%) |
| --- | --- | --- |
| susp | 123 | 41.7% |
| tb | 120 | 40.7% |
| n/realiz | 21 | 7.1% |
| normal | 20 | 6.8% |
| outra | 4 | 1.4% |
| patologia | 4 | 1.4% |
| c/cavid | 3 | 1.0% |

- Characters
- Categories
- Scripts
- Blocks

#### Most occurring characters

| Value | Count | Frequency (%) |
| --- | --- | --- |
|  | 702 | 10.0% |
| u | 702 | 10.0% |
| S | 696 | 9.9% |
| s | 696 | 9.9% |
| p | 696 | 9.9% |
| T | 517 | 7.3% |
| B | 517 | 7.3% |
| c | 358 | 5.1% |
| a | 352 | 5.0% |
| i | 310 | 4.4% |
| Other values (14) | 1491 | 21.2% |

| Value | Count | Frequency (%) |
| --- | --- | --- |
|  | 420 | 10.0% |
| u | 420 | 10.0% |
| S | 413 | 9.8% |
| s | 413 | 9.8% |
| p | 413 | 9.8% |
| T | 304 | 7.2% |
| B | 304 | 7.2% |
| a | 221 | 5.2% |
| c | 218 | 5.2% |
| i | 178 | 4.2% |
| Other values (14) | 907 | 21.5% |

| Value | Count | Frequency (%) |
| --- | --- | --- |
|  | 127 | 10.4% |
| u | 127 | 10.4% |
| S | 123 | 10.0% |
| s | 123 | 10.0% |
| p | 123 | 10.0% |
| T | 120 | 9.8% |
| B | 120 | 9.8% |
| a | 56 | 4.6% |
| r | 45 | 3.7% |
| l | 45 | 3.7% |
| Other values (14) | 215 | 17.6% |

#### Most occurring categories

| Value | Count | Frequency (%) |
| --- | --- | --- |
| Lowercase Letter | 4134 | 58.7% |
| Uppercase Letter | 1897 | 27.0% |
| Space Separator | 702 | 10.0% |
| Other Punctuation | 304 | 4.3% |

| Value | Count | Frequency (%) |
| --- | --- | --- |
| Lowercase Letter | 2494 | 59.2% |
| Uppercase Letter | 1126 | 26.7% |
| Space Separator | 420 | 10.0% |
| Other Punctuation | 171 | 4.1% |

| Value | Count | Frequency (%) |
| --- | --- | --- |
| Lowercase Letter | 661 | 54.0% |
| Uppercase Letter | 412 | 33.7% |
| Space Separator | 127 | 10.4% |
| Other Punctuation | 24 | 2.0% |

#### Most frequent character per category

##### *Space Separator*

| Value | Count | Frequency (%) |
| --- | --- | --- |
|  | 702 | 100.0% |

| Value | Count | Frequency (%) |
| --- | --- | --- |
|  | 420 | 100.0% |

| Value | Count | Frequency (%) |
| --- | --- | --- |
|  | 127 | 100.0% |

##### *Lowercase Letter*

| Value | Count | Frequency (%) |
| --- | --- | --- |
| u | 702 | 17.0% |
| s | 696 | 16.8% |
| p | 696 | 16.8% |
| c | 358 | 8.7% |
| a | 352 | 8.5% |
| i | 310 | 7.5% |
| d | 179 | 4.3% |
| v | 179 | 4.3% |
| r | 161 | 3.9% |
| l | 161 | 3.9% |
| Other values (6) | 340 | 8.2% |

| Value | Count | Frequency (%) |
| --- | --- | --- |
| u | 420 | 16.8% |
| s | 413 | 16.6% |
| p | 413 | 16.6% |
| a | 221 | 8.9% |
| c | 218 | 8.7% |
| i | 178 | 7.1% |
| d | 109 | 4.4% |
| v | 109 | 4.4% |
| r | 98 | 3.9% |
| l | 98 | 3.9% |
| Other values (6) | 217 | 8.7% |

| Value | Count | Frequency (%) |
| --- | --- | --- |
| u | 127 | 19.2% |
| s | 123 | 18.6% |
| p | 123 | 18.6% |
| a | 56 | 8.5% |
| r | 45 | 6.8% |
| l | 45 | 6.8% |
| i | 28 | 4.2% |
| o | 28 | 4.2% |
| e | 21 | 3.2% |
| z | 21 | 3.2% |
| Other values (6) | 44 | 6.7% |

##### *Uppercase Letter*

| Value | Count | Frequency (%) |
| --- | --- | --- |
| S | 696 | 36.7% |
| T | 517 | 27.3% |
| B | 517 | 27.3% |
| N | 155 | 8.2% |
| O | 6 | 0.3% |
| P | 6 | 0.3% |

| Value | Count | Frequency (%) |
| --- | --- | --- |
| S | 413 | 36.7% |
| T | 304 | 27.0% |
| B | 304 | 27.0% |
| N | 91 | 8.1% |
| O | 7 | 0.6% |
| P | 7 | 0.6% |

| Value | Count | Frequency (%) |
| --- | --- | --- |
| S | 123 | 29.9% |
| T | 120 | 29.1% |
| B | 120 | 29.1% |
| N | 41 | 10.0% |
| O | 4 | 1.0% |
| P | 4 | 1.0% |

##### *Other Punctuation*

| Value | Count | Frequency (%) |
| --- | --- | --- |
| / | 304 | 100.0% |

| Value | Count | Frequency (%) |
| --- | --- | --- |
| / | 171 | 100.0% |

| Value | Count | Frequency (%) |
| --- | --- | --- |
| / | 24 | 100.0% |

#### Most occurring scripts

| Value | Count | Frequency (%) |
| --- | --- | --- |
| Latin | 6031 | 85.7% |
| Common | 1006 | 14.3% |

| Value | Count | Frequency (%) |
| --- | --- | --- |
| Latin | 3620 | 86.0% |
| Common | 591 | 14.0% |

| Value | Count | Frequency (%) |
| --- | --- | --- |
| Latin | 1073 | 87.7% |
| Common | 151 | 12.3% |

#### Most frequent character per script

##### *Common*

| Value | Count | Frequency (%) |
| --- | --- | --- |
|  | 702 | 69.8% |
| / | 304 | 30.2% |

| Value | Count | Frequency (%) |
| --- | --- | --- |
|  | 420 | 71.1% |
| / | 171 | 28.9% |

| Value | Count | Frequency (%) |
| --- | --- | --- |
|  | 127 | 84.1% |
| / | 24 | 15.9% |

##### *Latin*

| Value | Count | Frequency (%) |
| --- | --- | --- |
| u | 702 | 11.6% |
| S | 696 | 11.5% |
| s | 696 | 11.5% |
| p | 696 | 11.5% |
| T | 517 | 8.6% |
| B | 517 | 8.6% |
| c | 358 | 5.9% |
| a | 352 | 5.8% |
| i | 310 | 5.1% |
| d | 179 | 3.0% |
| Other values (12) | 1008 | 16.7% |

| Value | Count | Frequency (%) |
| --- | --- | --- |
| u | 420 | 11.6% |
| S | 413 | 11.4% |
| s | 413 | 11.4% |
| p | 413 | 11.4% |
| T | 304 | 8.4% |
| B | 304 | 8.4% |
| a | 221 | 6.1% |
| c | 218 | 6.0% |
| i | 178 | 4.9% |
| d | 109 | 3.0% |
| Other values (12) | 627 | 17.3% |

| Value | Count | Frequency (%) |
| --- | --- | --- |
| u | 127 | 11.8% |
| S | 123 | 11.5% |
| s | 123 | 11.5% |
| p | 123 | 11.5% |
| T | 120 | 11.2% |
| B | 120 | 11.2% |
| a | 56 | 5.2% |
| r | 45 | 4.2% |
| l | 45 | 4.2% |
| N | 41 | 3.8% |
| Other values (12) | 150 | 14.0% |

#### Most occurring blocks

| Value | Count | Frequency (%) |
| --- | --- | --- |
| ASCII | 7037 | 100.0% |

| Value | Count | Frequency (%) |
| --- | --- | --- |
| ASCII | 4211 | 100.0% |

| Value | Count | Frequency (%) |
| --- | --- | --- |
| ASCII | 1224 | 100.0% |

#### Most frequent character per block

##### *ASCII*

| Value | Count | Frequency (%) |
| --- | --- | --- |
|  | 702 | 10.0% |
| u | 702 | 10.0% |
| S | 696 | 9.9% |
| s | 696 | 9.9% |
| p | 696 | 9.9% |
| T | 517 | 7.3% |
| B | 517 | 7.3% |
| c | 358 | 5.1% |
| a | 352 | 5.0% |
| i | 310 | 4.4% |
| Other values (14) | 1491 | 21.2% |

| Value | Count | Frequency (%) |
| --- | --- | --- |
|  | 420 | 10.0% |
| u | 420 | 10.0% |
| S | 413 | 9.8% |
| s | 413 | 9.8% |
| p | 413 | 9.8% |
| T | 304 | 7.2% |
| B | 304 | 7.2% |
| a | 221 | 5.2% |
| c | 218 | 5.2% |
| i | 178 | 4.2% |
| Other values (14) | 907 | 21.5% |

| Value | Count | Frequency (%) |
| --- | --- | --- |
|  | 127 | 10.4% |
| u | 127 | 10.4% |
| S | 123 | 10.0% |
| s | 123 | 10.0% |
| p | 123 | 10.0% |
| T | 120 | 9.8% |
| B | 120 | 9.8% |
| a | 56 | 4.6% |
| r | 45 | 3.7% |
| l | 45 | 3.7% |
| Other values (14) | 215 | 17.6% |

NECROP  
Categorical

|
|  |
|
|  |

|  | Cluster 1 | Cluster 2 | Cluster 3 |
| --- | --- | --- | --- |
| Distinct | 2 | 2 | 2 |
| Distinct (%) | 0.2% | 0.4% | 1.2% |
| Missing | 0 | 0 | 0 |
| Missing (%) | 0.0% | 0.0% | 0.0% |
| Memory size | 13.4 KiB | 8.0 KiB | 2.6 KiB |

|  |  |
| --- | --- |
| N/realiz | 854 |
| Sugestivo TB | 3 |

|  |  |
| --- | --- |
| N/realiz | 509 |
| BAAR pos | 2 |

|  |  |
| --- | --- |
| N/realiz | 167 |
| Sugestivo TB | 1 |

More details

- Overview
- Categories
- Words
- Characters

Length

|  | Cluster 1 | Cluster 2 | Cluster 3 |
| --- | --- | --- | --- |
| Max length | 12 | 8 | 12 |
| Median length | 8 | 8 | 8 |
| Mean length | 8.0140023 | 8 | 8.0238095 |
| Min length | 8 | 8 | 8 |

Characters and Unicode

|  | Cluster 1 | Cluster 2 | Cluster 3 |
| --- | --- | --- | --- |
| Total characters | 6868 | 4088 | 1348 |
| Distinct characters | 18 | 15 | 18 |
| Distinct categories | 4 | 4 | 4 ? |
| Distinct scripts | 2 | 2 | 2 ? |
| Distinct blocks | 1 | 1 | 1 ? |

The Unicode Standard assigns character properties to each code point, which can be used to analyse textual variables.

Unique

|  | Cluster 1 | Cluster 2 | Cluster 3 |
| --- | --- | --- | --- |
| Unique | 0 | 0 | 1 ? |
| Unique (%) | 0.0% | 0.0% | 0.6% |

Sample

|  | Cluster 1 | Cluster 2 | Cluster 3 |
| --- | --- | --- | --- |
| 1st row | N/realiz | N/realiz | N/realiz |
| 2nd row | N/realiz | N/realiz | N/realiz |
| 3rd row | N/realiz | N/realiz | N/realiz |
| 4th row | N/realiz | N/realiz | N/realiz |
| 5th row | N/realiz | N/realiz | N/realiz |

#### Common Values

| Value | Count | Frequency (%) |
| --- | --- | --- |
| N/realiz | 854 | 99.6% |
| Sugestivo TB | 3 | 0.4% |

| Value | Count | Frequency (%) |
| --- | --- | --- |
| N/realiz | 509 | 99.6% |
| BAAR pos | 2 | 0.4% |

| Value | Count | Frequency (%) |
| --- | --- | --- |
| N/realiz | 167 | 99.4% |
| Sugestivo TB | 1 | 0.6% |

#### Length

xml version="1.0" encoding="utf-8" standalone="no"?2023-08-25T13:07:47.258181image/svg+xmlMatplotlib v3.6.0, https://matplotlib.org/ 

Histogram of lengths of the category

#### Common Values (Plot)

#### Cluster 1

xml version="1.0" encoding="utf-8" standalone="no"?2023-08-25T13:07:47.403401image/svg+xmlMatplotlib v3.6.0, https://matplotlib.org/

#### Cluster 2

xml version="1.0" encoding="utf-8" standalone="no"?2023-08-25T13:07:47.520702image/svg+xmlMatplotlib v3.6.0, https://matplotlib.org/

#### Cluster 3

xml version="1.0" encoding="utf-8" standalone="no"?2023-08-25T13:07:47.641593image/svg+xmlMatplotlib v3.6.0, https://matplotlib.org/

| Value | Count | Frequency (%) |
| --- | --- | --- |
| n/realiz | 854 | 99.3% |
| sugestivo | 3 | 0.3% |
| tb | 3 | 0.3% |

| Value | Count | Frequency (%) |
| --- | --- | --- |
| n/realiz | 509 | 99.2% |
| baar | 2 | 0.4% |
| pos | 2 | 0.4% |

| Value | Count | Frequency (%) |
| --- | --- | --- |
| n/realiz | 167 | 98.8% |
| sugestivo | 1 | 0.6% |
| tb | 1 | 0.6% |

- Characters
- Categories
- Scripts
- Blocks

#### Most occurring characters

| Value | Count | Frequency (%) |
| --- | --- | --- |
| e | 857 | 12.5% |
| i | 857 | 12.5% |
| N | 854 | 12.4% |
| r | 854 | 12.4% |
| a | 854 | 12.4% |
| l | 854 | 12.4% |
| z | 854 | 12.4% |
| / | 854 | 12.4% |
| v | 3 | < 0.1% |
| T | 3 | < 0.1% |
| Other values (8) | 24 | 0.3% |

| Value | Count | Frequency (%) |
| --- | --- | --- |
| N | 509 | 12.5% |
| / | 509 | 12.5% |
| r | 509 | 12.5% |
| e | 509 | 12.5% |
| a | 509 | 12.5% |
| l | 509 | 12.5% |
| i | 509 | 12.5% |
| z | 509 | 12.5% |
| A | 4 | 0.1% |
| B | 2 | < 0.1% |
| Other values (5) | 10 | 0.2% |

| Value | Count | Frequency (%) |
| --- | --- | --- |
| e | 168 | 12.5% |
| i | 168 | 12.5% |
| N | 167 | 12.4% |
| r | 167 | 12.4% |
| a | 167 | 12.4% |
| l | 167 | 12.4% |
| z | 167 | 12.4% |
| / | 167 | 12.4% |
| v | 1 | 0.1% |
| T | 1 | 0.1% |
| Other values (8) | 8 | 0.6% |

#### Most occurring categories

| Value | Count | Frequency (%) |
| --- | --- | --- |
| Lowercase Letter | 5148 | 75.0% |
| Uppercase Letter | 863 | 12.6% |
| Other Punctuation | 854 | 12.4% |
| Space Separator | 3 | < 0.1% |

| Value | Count | Frequency (%) |
| --- | --- | --- |
| Lowercase Letter | 3060 | 74.9% |
| Uppercase Letter | 517 | 12.6% |
| Other Punctuation | 509 | 12.5% |
| Space Separator | 2 | < 0.1% |

| Value | Count | Frequency (%) |
| --- | --- | --- |
| Lowercase Letter | 1010 | 74.9% |
| Uppercase Letter | 170 | 12.6% |
| Other Punctuation | 167 | 12.4% |
| Space Separator | 1 | 0.1% |

#### Most frequent character per category

##### *Lowercase Letter*

| Value | Count | Frequency (%) |
| --- | --- | --- |
| e | 857 | 16.6% |
| i | 857 | 16.6% |
| r | 854 | 16.6% |
| a | 854 | 16.6% |
| l | 854 | 16.6% |
| z | 854 | 16.6% |
| v | 3 | 0.1% |
| o | 3 | 0.1% |
| u | 3 | 0.1% |
| t | 3 | 0.1% |
| Other values (2) | 6 | 0.1% |

| Value | Count | Frequency (%) |
| --- | --- | --- |
| r | 509 | 16.6% |
| e | 509 | 16.6% |
| a | 509 | 16.6% |
| l | 509 | 16.6% |
| i | 509 | 16.6% |
| z | 509 | 16.6% |
| p | 2 | 0.1% |
| o | 2 | 0.1% |
| s | 2 | 0.1% |

| Value | Count | Frequency (%) |
| --- | --- | --- |
| e | 168 | 16.6% |
| i | 168 | 16.6% |
| r | 167 | 16.5% |
| a | 167 | 16.5% |
| l | 167 | 16.5% |
| z | 167 | 16.5% |
| v | 1 | 0.1% |
| o | 1 | 0.1% |
| u | 1 | 0.1% |
| t | 1 | 0.1% |
| Other values (2) | 2 | 0.2% |

##### *Uppercase Letter*

| Value | Count | Frequency (%) |
| --- | --- | --- |
| N | 854 | 99.0% |
| T | 3 | 0.3% |
| S | 3 | 0.3% |
| B | 3 | 0.3% |

| Value | Count | Frequency (%) |
| --- | --- | --- |
| N | 509 | 98.5% |
| A | 4 | 0.8% |
| B | 2 | 0.4% |
| R | 2 | 0.4% |

| Value | Count | Frequency (%) |
| --- | --- | --- |
| N | 167 | 98.2% |
| T | 1 | 0.6% |
| S | 1 | 0.6% |
| B | 1 | 0.6% |

##### *Other Punctuation*

| Value | Count | Frequency (%) |
| --- | --- | --- |
| / | 854 | 100.0% |

| Value | Count | Frequency (%) |
| --- | --- | --- |
| / | 509 | 100.0% |

| Value | Count | Frequency (%) |
| --- | --- | --- |
| / | 167 | 100.0% |

##### *Space Separator*

| Value | Count | Frequency (%) |
| --- | --- | --- |
|  | 3 | 100.0% |

| Value | Count | Frequency (%) |
| --- | --- | --- |
|  | 2 | 100.0% |

| Value | Count | Frequency (%) |
| --- | --- | --- |
|  | 1 | 100.0% |

#### Most occurring scripts

| Value | Count | Frequency (%) |
| --- | --- | --- |
| Latin | 6011 | 87.5% |
| Common | 857 | 12.5% |

| Value | Count | Frequency (%) |
| --- | --- | --- |
| Latin | 3577 | 87.5% |
| Common | 511 | 12.5% |

| Value | Count | Frequency (%) |
| --- | --- | --- |
| Latin | 1180 | 87.5% |
| Common | 168 | 12.5% |

#### Most frequent character per script

##### *Latin*

| Value | Count | Frequency (%) |
| --- | --- | --- |
| e | 857 | 14.3% |
| i | 857 | 14.3% |
| N | 854 | 14.2% |
| r | 854 | 14.2% |
| a | 854 | 14.2% |
| l | 854 | 14.2% |
| z | 854 | 14.2% |
| v | 3 | < 0.1% |
| T | 3 | < 0.1% |
| o | 3 | < 0.1% |
| Other values (6) | 18 | 0.3% |

| Value | Count | Frequency (%) |
| --- | --- | --- |
| N | 509 | 14.2% |
| r | 509 | 14.2% |
| e | 509 | 14.2% |
| a | 509 | 14.2% |
| l | 509 | 14.2% |
| i | 509 | 14.2% |
| z | 509 | 14.2% |
| A | 4 | 0.1% |
| B | 2 | 0.1% |
| R | 2 | 0.1% |
| Other values (3) | 6 | 0.2% |

| Value | Count | Frequency (%) |
| --- | --- | --- |
| e | 168 | 14.2% |
| i | 168 | 14.2% |
| N | 167 | 14.2% |
| r | 167 | 14.2% |
| a | 167 | 14.2% |
| l | 167 | 14.2% |
| z | 167 | 14.2% |
| v | 1 | 0.1% |
| T | 1 | 0.1% |
| o | 1 | 0.1% |
| Other values (6) | 6 | 0.5% |

##### *Common*

| Value | Count | Frequency (%) |
| --- | --- | --- |
| / | 854 | 99.6% |
|  | 3 | 0.4% |

| Value | Count | Frequency (%) |
| --- | --- | --- |
| / | 509 | 99.6% |
|  | 2 | 0.4% |

| Value | Count | Frequency (%) |
| --- | --- | --- |
| / | 167 | 99.4% |
|  | 1 | 0.6% |

#### Most occurring blocks

| Value | Count | Frequency (%) |
| --- | --- | --- |
| ASCII | 6868 | 100.0% |

| Value | Count | Frequency (%) |
| --- | --- | --- |
| ASCII | 4088 | 100.0% |

| Value | Count | Frequency (%) |
| --- | --- | --- |
| ASCII | 1348 | 100.0% |

#### Most frequent character per block

##### *ASCII*

| Value | Count | Frequency (%) |
| --- | --- | --- |
| e | 857 | 12.5% |
| i | 857 | 12.5% |
| N | 854 | 12.4% |
| r | 854 | 12.4% |
| a | 854 | 12.4% |
| l | 854 | 12.4% |
| z | 854 | 12.4% |
| / | 854 | 12.4% |
| v | 3 | < 0.1% |
| T | 3 | < 0.1% |
| Other values (8) | 24 | 0.3% |

| Value | Count | Frequency (%) |
| --- | --- | --- |
| N | 509 | 12.5% |
| / | 509 | 12.5% |
| r | 509 | 12.5% |
| e | 509 | 12.5% |
| a | 509 | 12.5% |
| l | 509 | 12.5% |
| i | 509 | 12.5% |
| z | 509 | 12.5% |
| A | 4 | 0.1% |
| B | 2 | < 0.1% |
| Other values (5) | 10 | 0.2% |

| Value | Count | Frequency (%) |
| --- | --- | --- |
| e | 168 | 12.5% |
| i | 168 | 12.5% |
| N | 167 | 12.4% |
| r | 167 | 12.4% |
| a | 167 | 12.4% |
| l | 167 | 12.4% |
| z | 167 | 12.4% |
| / | 167 | 12.4% |
| v | 1 | 0.1% |
| T | 1 | 0.1% |
| Other values (8) | 8 | 0.6% |

hiv  
Categorical

|
|  |
|
|  |

|  | Cluster 1 | Cluster 2 | Cluster 3 |
| --- | --- | --- | --- |
| Distinct | 3 | 4 | 3 |
| Distinct (%) | 0.4% | 0.8% | 1.8% |
| Missing | 0 | 0 | 0 |
| Missing (%) | 0.0% | 0.0% | 0.0% |
| Memory size | 13.4 KiB | 8.0 KiB | 2.6 KiB |

|  |  |
| --- | --- |
| Neg | 738 |
| Pos | 63 |
| N/realiz | 56 |

|  |  |
| --- | --- |
| Neg | 451 |
| N/realiz | 44 |
| Pos | 15 |
| And | 1 |

|  |  |
| --- | --- |
| Pos | 148 |
| Neg | 11 |
| N/realiz | 9 |

More details

- Overview
- Categories
- Words
- Characters

Length

|  | Cluster 1 | Cluster 2 | Cluster 3 |
| --- | --- | --- | --- |
| Max length | 8 | 8 | 8 |
| Median length | 3 | 3 | 3 |
| Mean length | 3.3267211 | 3.4305284 | 3.2678571 |
| Min length | 3 | 3 | 3 |

Characters and Unicode

|  | Cluster 1 | Cluster 2 | Cluster 3 |
| --- | --- | --- | --- |
| Total characters | 2851 | 1753 | 549 |
| Distinct characters | 12 | 15 | 12 |
| Distinct categories | 3 | 3 | 3 ? |
| Distinct scripts | 2 | 2 | 2 ? |
| Distinct blocks | 1 | 1 | 1 ? |

The Unicode Standard assigns character properties to each code point, which can be used to analyse textual variables.

Unique

|  | Cluster 1 | Cluster 2 | Cluster 3 |
| --- | --- | --- | --- |
| Unique | 0 | 1 | 0 ? |
| Unique (%) | 0.0% | 0.2% | 0.0% |

Sample

|  | Cluster 1 | Cluster 2 | Cluster 3 |
| --- | --- | --- | --- |
| 1st row | Neg | Neg | Pos |
| 2nd row | Neg | Neg | Pos |
| 3rd row | Pos | Neg | Pos |
| 4th row | Pos | Neg | N/realiz |
| 5th row | Neg | Neg | Pos |

#### Common Values

| Value | Count | Frequency (%) |
| --- | --- | --- |
| Neg | 738 | 86.1% |
| Pos | 63 | 7.4% |
| N/realiz | 56 | 6.5% |

| Value | Count | Frequency (%) |
| --- | --- | --- |
| Neg | 451 | 88.3% |
| N/realiz | 44 | 8.6% |
| Pos | 15 | 2.9% |
| And | 1 | 0.2% |

| Value | Count | Frequency (%) |
| --- | --- | --- |
| Pos | 148 | 88.1% |
| Neg | 11 | 6.5% |
| N/realiz | 9 | 5.4% |

#### Length

xml version="1.0" encoding="utf-8" standalone="no"?2023-08-25T13:07:47.760577image/svg+xmlMatplotlib v3.6.0, https://matplotlib.org/ 

Histogram of lengths of the category

#### Common Values (Plot)

#### Cluster 1

xml version="1.0" encoding="utf-8" standalone="no"?2023-08-25T13:07:47.911029image/svg+xmlMatplotlib v3.6.0, https://matplotlib.org/

#### Cluster 2

xml version="1.0" encoding="utf-8" standalone="no"?2023-08-25T13:07:48.044990image/svg+xmlMatplotlib v3.6.0, https://matplotlib.org/

#### Cluster 3

xml version="1.0" encoding="utf-8" standalone="no"?2023-08-25T13:07:48.194354image/svg+xmlMatplotlib v3.6.0, https://matplotlib.org/

| Value | Count | Frequency (%) |
| --- | --- | --- |
| neg | 738 | 86.1% |
| pos | 63 | 7.4% |
| n/realiz | 56 | 6.5% |

| Value | Count | Frequency (%) |
| --- | --- | --- |
| neg | 451 | 88.3% |
| n/realiz | 44 | 8.6% |
| pos | 15 | 2.9% |
| and | 1 | 0.2% |

| Value | Count | Frequency (%) |
| --- | --- | --- |
| pos | 148 | 88.1% |
| neg | 11 | 6.5% |
| n/realiz | 9 | 5.4% |

- Characters
- Categories
- Scripts
- Blocks

#### Most occurring characters

| Value | Count | Frequency (%) |
| --- | --- | --- |
| N | 794 | 27.8% |
| e | 794 | 27.8% |
| g | 738 | 25.9% |
| P | 63 | 2.2% |
| o | 63 | 2.2% |
| s | 63 | 2.2% |
| / | 56 | 2.0% |
| r | 56 | 2.0% |
| a | 56 | 2.0% |
| l | 56 | 2.0% |
| Other values (2) | 112 | 3.9% |

| Value | Count | Frequency (%) |
| --- | --- | --- |
| N | 495 | 28.2% |
| e | 495 | 28.2% |
| g | 451 | 25.7% |
| / | 44 | 2.5% |
| r | 44 | 2.5% |
| a | 44 | 2.5% |
| l | 44 | 2.5% |
| i | 44 | 2.5% |
| z | 44 | 2.5% |
| P | 15 | 0.9% |
| Other values (5) | 33 | 1.9% |

| Value | Count | Frequency (%) |
| --- | --- | --- |
| P | 148 | 27.0% |
| o | 148 | 27.0% |
| s | 148 | 27.0% |
| N | 20 | 3.6% |
| e | 20 | 3.6% |
| g | 11 | 2.0% |
| / | 9 | 1.6% |
| r | 9 | 1.6% |
| a | 9 | 1.6% |
| l | 9 | 1.6% |
| Other values (2) | 18 | 3.3% |

#### Most occurring categories

| Value | Count | Frequency (%) |
| --- | --- | --- |
| Lowercase Letter | 1938 | 68.0% |
| Uppercase Letter | 857 | 30.1% |
| Other Punctuation | 56 | 2.0% |

| Value | Count | Frequency (%) |
| --- | --- | --- |
| Lowercase Letter | 1198 | 68.3% |
| Uppercase Letter | 511 | 29.2% |
| Other Punctuation | 44 | 2.5% |

| Value | Count | Frequency (%) |
| --- | --- | --- |
| Lowercase Letter | 372 | 67.8% |
| Uppercase Letter | 168 | 30.6% |
| Other Punctuation | 9 | 1.6% |

#### Most frequent character per category

##### *Uppercase Letter*

| Value | Count | Frequency (%) |
| --- | --- | --- |
| N | 794 | 92.6% |
| P | 63 | 7.4% |

| Value | Count | Frequency (%) |
| --- | --- | --- |
| N | 495 | 96.9% |
| P | 15 | 2.9% |
| A | 1 | 0.2% |

| Value | Count | Frequency (%) |
| --- | --- | --- |
| P | 148 | 88.1% |
| N | 20 | 11.9% |

##### *Lowercase Letter*

| Value | Count | Frequency (%) |
| --- | --- | --- |
| e | 794 | 41.0% |
| g | 738 | 38.1% |
| o | 63 | 3.3% |
| s | 63 | 3.3% |
| r | 56 | 2.9% |
| a | 56 | 2.9% |
| l | 56 | 2.9% |
| i | 56 | 2.9% |
| z | 56 | 2.9% |

| Value | Count | Frequency (%) |
| --- | --- | --- |
| e | 495 | 41.3% |
| g | 451 | 37.6% |
| r | 44 | 3.7% |
| a | 44 | 3.7% |
| l | 44 | 3.7% |
| i | 44 | 3.7% |
| z | 44 | 3.7% |
| o | 15 | 1.3% |
| s | 15 | 1.3% |
| n | 1 | 0.1% |

| Value | Count | Frequency (%) |
| --- | --- | --- |
| o | 148 | 39.8% |
| s | 148 | 39.8% |
| e | 20 | 5.4% |
| g | 11 | 3.0% |
| r | 9 | 2.4% |
| a | 9 | 2.4% |
| l | 9 | 2.4% |
| i | 9 | 2.4% |
| z | 9 | 2.4% |

##### *Other Punctuation*

| Value | Count | Frequency (%) |
| --- | --- | --- |
| / | 56 | 100.0% |

| Value | Count | Frequency (%) |
| --- | --- | --- |
| / | 44 | 100.0% |

| Value | Count | Frequency (%) |
| --- | --- | --- |
| / | 9 | 100.0% |

#### Most occurring scripts

| Value | Count | Frequency (%) |
| --- | --- | --- |
| Latin | 2795 | 98.0% |
| Common | 56 | 2.0% |

| Value | Count | Frequency (%) |
| --- | --- | --- |
| Latin | 1709 | 97.5% |
| Common | 44 | 2.5% |

| Value | Count | Frequency (%) |
| --- | --- | --- |
| Latin | 540 | 98.4% |
| Common | 9 | 1.6% |

#### Most frequent character per script

##### *Latin*

| Value | Count | Frequency (%) |
| --- | --- | --- |
| N | 794 | 28.4% |
| e | 794 | 28.4% |
| g | 738 | 26.4% |
| P | 63 | 2.3% |
| o | 63 | 2.3% |
| s | 63 | 2.3% |
| r | 56 | 2.0% |
| a | 56 | 2.0% |
| l | 56 | 2.0% |
| i | 56 | 2.0% |

| Value | Count | Frequency (%) |
| --- | --- | --- |
| N | 495 | 29.0% |
| e | 495 | 29.0% |
| g | 451 | 26.4% |
| r | 44 | 2.6% |
| a | 44 | 2.6% |
| l | 44 | 2.6% |
| i | 44 | 2.6% |
| z | 44 | 2.6% |
| P | 15 | 0.9% |
| o | 15 | 0.9% |
| Other values (4) | 18 | 1.1% |

| Value | Count | Frequency (%) |
| --- | --- | --- |
| P | 148 | 27.4% |
| o | 148 | 27.4% |
| s | 148 | 27.4% |
| N | 20 | 3.7% |
| e | 20 | 3.7% |
| g | 11 | 2.0% |
| r | 9 | 1.7% |
| a | 9 | 1.7% |
| l | 9 | 1.7% |
| i | 9 | 1.7% |

##### *Common*

| Value | Count | Frequency (%) |
| --- | --- | --- |
| / | 56 | 100.0% |

| Value | Count | Frequency (%) |
| --- | --- | --- |
| / | 44 | 100.0% |

| Value | Count | Frequency (%) |
| --- | --- | --- |
| / | 9 | 100.0% |

#### Most occurring blocks

| Value | Count | Frequency (%) |
| --- | --- | --- |
| ASCII | 2851 | 100.0% |

| Value | Count | Frequency (%) |
| --- | --- | --- |
| ASCII | 1753 | 100.0% |

| Value | Count | Frequency (%) |
| --- | --- | --- |
| ASCII | 549 | 100.0% |

#### Most frequent character per block

##### *ASCII*

| Value | Count | Frequency (%) |
| --- | --- | --- |
| N | 794 | 27.8% |
| e | 794 | 27.8% |
| g | 738 | 25.9% |
| P | 63 | 2.2% |
| o | 63 | 2.2% |
| s | 63 | 2.2% |
| / | 56 | 2.0% |
| r | 56 | 2.0% |
| a | 56 | 2.0% |
| l | 56 | 2.0% |
| Other values (2) | 112 | 3.9% |

| Value | Count | Frequency (%) |
| --- | --- | --- |
| N | 495 | 28.2% |
| e | 495 | 28.2% |
| g | 451 | 25.7% |
| / | 44 | 2.5% |
| r | 44 | 2.5% |
| a | 44 | 2.5% |
| l | 44 | 2.5% |
| i | 44 | 2.5% |
| z | 44 | 2.5% |
| P | 15 | 0.9% |
| Other values (5) | 33 | 1.9% |

| Value | Count | Frequency (%) |
| --- | --- | --- |
| P | 148 | 27.0% |
| o | 148 | 27.0% |
| s | 148 | 27.0% |
| N | 20 | 3.6% |
| e | 20 | 3.6% |
| g | 11 | 2.0% |
| / | 9 | 1.6% |
| r | 9 | 1.6% |
| a | 9 | 1.6% |
| l | 9 | 1.6% |
| Other values (2) | 18 | 3.3% |

aids  
Categorical

|
|  |
|
|  |

|  | Cluster 1 | Cluster 2 | Cluster 3 |
| --- | --- | --- | --- |
| Distinct | 2 | 2 | 2 |
| Distinct (%) | 0.2% | 0.4% | 1.2% |
| Missing | 0 | 0 | 0 |
| Missing (%) | 0.0% | 0.0% | 0.0% |
| Memory size | 13.4 KiB | 8.0 KiB | 2.6 KiB |

|  |  |
| --- | --- |
| N | 807 |
| S | 50 |

|  |  |
| --- | --- |
| N | 498 |
| S | 13 |

|  |  |
| --- | --- |
| S | 144 |
| N | 24 |

More details

- Overview
- Categories
- Words
- Characters

Length

|  | Cluster 1 | Cluster 2 | Cluster 3 |
| --- | --- | --- | --- |
| Max length | 1 | 1 | 1 |
| Median length | 1 | 1 | 1 |
| Mean length | 1 | 1 | 1 |
| Min length | 1 | 1 | 1 |

Characters and Unicode

|  | Cluster 1 | Cluster 2 | Cluster 3 |
| --- | --- | --- | --- |
| Total characters | 857 | 511 | 168 |
| Distinct characters | 2 | 2 | 2 |
| Distinct categories | 1 | 1 | 1 ? |
| Distinct scripts | 1 | 1 | 1 ? |
| Distinct blocks | 1 | 1 | 1 ? |

The Unicode Standard assigns character properties to each code point, which can be used to analyse textual variables.

Unique

|  | Cluster 1 | Cluster 2 | Cluster 3 |
| --- | --- | --- | --- |
| Unique | 0 | 0 | 0 ? |
| Unique (%) | 0.0% | 0.0% | 0.0% |

Sample

|  | Cluster 1 | Cluster 2 | Cluster 3 |
| --- | --- | --- | --- |
| 1st row | N | N | S |
| 2nd row | N | N | S |
| 3rd row | S | N | S |
| 4th row | N | N | N |
| 5th row | N | N | S |

#### Common Values

| Value | Count | Frequency (%) |
| --- | --- | --- |
| N | 807 | 94.2% |
| S | 50 | 5.8% |

| Value | Count | Frequency (%) |
| --- | --- | --- |
| N | 498 | 97.5% |
| S | 13 | 2.5% |

| Value | Count | Frequency (%) |
| --- | --- | --- |
| S | 144 | 85.7% |
| N | 24 | 14.3% |

#### Length

xml version="1.0" encoding="utf-8" standalone="no"?2023-08-25T13:07:48.317600image/svg+xmlMatplotlib v3.6.0, https://matplotlib.org/ 

Histogram of lengths of the category

#### Common Values (Plot)

#### Cluster 1

xml version="1.0" encoding="utf-8" standalone="no"?2023-08-25T13:07:48.462224image/svg+xmlMatplotlib v3.6.0, https://matplotlib.org/

#### Cluster 2

xml version="1.0" encoding="utf-8" standalone="no"?2023-08-25T13:07:48.579883image/svg+xmlMatplotlib v3.6.0, https://matplotlib.org/

#### Cluster 3

xml version="1.0" encoding="utf-8" standalone="no"?2023-08-25T13:07:48.700035image/svg+xmlMatplotlib v3.6.0, https://matplotlib.org/

| Value | Count | Frequency (%) |
| --- | --- | --- |
| n | 807 | 94.2% |
| s | 50 | 5.8% |

| Value | Count | Frequency (%) |
| --- | --- | --- |
| n | 498 | 97.5% |
| s | 13 | 2.5% |

| Value | Count | Frequency (%) |
| --- | --- | --- |
| s | 144 | 85.7% |
| n | 24 | 14.3% |

- Characters
- Categories
- Scripts
- Blocks

#### Most occurring characters

| Value | Count | Frequency (%) |
| --- | --- | --- |
| N | 807 | 94.2% |
| S | 50 | 5.8% |

| Value | Count | Frequency (%) |
| --- | --- | --- |
| N | 498 | 97.5% |
| S | 13 | 2.5% |

| Value | Count | Frequency (%) |
| --- | --- | --- |
| S | 144 | 85.7% |
| N | 24 | 14.3% |

#### Most occurring categories

| Value | Count | Frequency (%) |
| --- | --- | --- |
| Uppercase Letter | 857 | 100.0% |

| Value | Count | Frequency (%) |
| --- | --- | --- |
| Uppercase Letter | 511 | 100.0% |

| Value | Count | Frequency (%) |
| --- | --- | --- |
| Uppercase Letter | 168 | 100.0% |

#### Most frequent character per category

##### *Uppercase Letter*

| Value | Count | Frequency (%) |
| --- | --- | --- |
| N | 807 | 94.2% |
| S | 50 | 5.8% |

| Value | Count | Frequency (%) |
| --- | --- | --- |
| N | 498 | 97.5% |
| S | 13 | 2.5% |

| Value | Count | Frequency (%) |
| --- | --- | --- |
| S | 144 | 85.7% |
| N | 24 | 14.3% |

#### Most occurring scripts

| Value | Count | Frequency (%) |
| --- | --- | --- |
| Latin | 857 | 100.0% |

| Value | Count | Frequency (%) |
| --- | --- | --- |
| Latin | 511 | 100.0% |

| Value | Count | Frequency (%) |
| --- | --- | --- |
| Latin | 168 | 100.0% |

#### Most frequent character per script

##### *Latin*

| Value | Count | Frequency (%) |
| --- | --- | --- |
| N | 807 | 94.2% |
| S | 50 | 5.8% |

| Value | Count | Frequency (%) |
| --- | --- | --- |
| N | 498 | 97.5% |
| S | 13 | 2.5% |

| Value | Count | Frequency (%) |
| --- | --- | --- |
| S | 144 | 85.7% |
| N | 24 | 14.3% |

#### Most occurring blocks

| Value | Count | Frequency (%) |
| --- | --- | --- |
| ASCII | 857 | 100.0% |

| Value | Count | Frequency (%) |
| --- | --- | --- |
| ASCII | 511 | 100.0% |

| Value | Count | Frequency (%) |
| --- | --- | --- |
| ASCII | 168 | 100.0% |

#### Most frequent character per block

##### *ASCII*

| Value | Count | Frequency (%) |
| --- | --- | --- |
| N | 807 | 94.2% |
| S | 50 | 5.8% |

| Value | Count | Frequency (%) |
| --- | --- | --- |
| N | 498 | 97.5% |
| S | 13 | 2.5% |

| Value | Count | Frequency (%) |
| --- | --- | --- |
| S | 144 | 85.7% |
| N | 24 | 14.3% |

DIABETES  
Categorical

|
|  |
|
|  |

|  | Cluster 1 | Cluster 2 | Cluster 3 |
| --- | --- | --- | --- |
| Distinct | 2 | 2 | 2 |
| Distinct (%) | 0.2% | 0.4% | 1.2% |
| Missing | 0 | 0 | 0 |
| Missing (%) | 0.0% | 0.0% | 0.0% |
| Memory size | 13.4 KiB | 8.0 KiB | 2.6 KiB |

|  |  |
| --- | --- |
| N | 793 |
| S | 64 |

|  |  |
| --- | --- |
| N | 481 |
| S | 30 |

|  |  |
| --- | --- |
| N | 165 |
| S | 3 |

More details

- Overview
- Categories
- Words
- Characters

Length

|  | Cluster 1 | Cluster 2 | Cluster 3 |
| --- | --- | --- | --- |
| Max length | 1 | 1 | 1 |
| Median length | 1 | 1 | 1 |
| Mean length | 1 | 1 | 1 |
| Min length | 1 | 1 | 1 |

Characters and Unicode

|  | Cluster 1 | Cluster 2 | Cluster 3 |
| --- | --- | --- | --- |
| Total characters | 857 | 511 | 168 |
| Distinct characters | 2 | 2 | 2 |
| Distinct categories | 1 | 1 | 1 ? |
| Distinct scripts | 1 | 1 | 1 ? |
| Distinct blocks | 1 | 1 | 1 ? |

The Unicode Standard assigns character properties to each code point, which can be used to analyse textual variables.

Unique

|  | Cluster 1 | Cluster 2 | Cluster 3 |
| --- | --- | --- | --- |
| Unique | 0 | 0 | 0 ? |
| Unique (%) | 0.0% | 0.0% | 0.0% |

Sample

|  | Cluster 1 | Cluster 2 | Cluster 3 |
| --- | --- | --- | --- |
| 1st row | N | N | N |
| 2nd row | N | N | N |
| 3rd row | N | N | N |
| 4th row | N | N | N |
| 5th row | N | N | N |

#### Common Values

| Value | Count | Frequency (%) |
| --- | --- | --- |
| N | 793 | 92.5% |
| S | 64 | 7.5% |

| Value | Count | Frequency (%) |
| --- | --- | --- |
| N | 481 | 94.1% |
| S | 30 | 5.9% |

| Value | Count | Frequency (%) |
| --- | --- | --- |
| N | 165 | 98.2% |
| S | 3 | 1.8% |

#### Length

xml version="1.0" encoding="utf-8" standalone="no"?2023-08-25T13:07:48.816504image/svg+xmlMatplotlib v3.6.0, https://matplotlib.org/ 

Histogram of lengths of the category

#### Common Values (Plot)

#### Cluster 1

xml version="1.0" encoding="utf-8" standalone="no"?2023-08-25T13:07:48.969680image/svg+xmlMatplotlib v3.6.0, https://matplotlib.org/

#### Cluster 2

xml version="1.0" encoding="utf-8" standalone="no"?2023-08-25T13:07:49.097142image/svg+xmlMatplotlib v3.6.0, https://matplotlib.org/

#### Cluster 3

xml version="1.0" encoding="utf-8" standalone="no"?2023-08-25T13:07:49.218898image/svg+xmlMatplotlib v3.6.0, https://matplotlib.org/

| Value | Count | Frequency (%) |
| --- | --- | --- |
| n | 793 | 92.5% |
| s | 64 | 7.5% |

| Value | Count | Frequency (%) |
| --- | --- | --- |
| n | 481 | 94.1% |
| s | 30 | 5.9% |

| Value | Count | Frequency (%) |
| --- | --- | --- |
| n | 165 | 98.2% |
| s | 3 | 1.8% |

- Characters
- Categories
- Scripts
- Blocks

#### Most occurring characters

| Value | Count | Frequency (%) |
| --- | --- | --- |
| N | 793 | 92.5% |
| S | 64 | 7.5% |

| Value | Count | Frequency (%) |
| --- | --- | --- |
| N | 481 | 94.1% |
| S | 30 | 5.9% |

| Value | Count | Frequency (%) |
| --- | --- | --- |
| N | 165 | 98.2% |
| S | 3 | 1.8% |

#### Most occurring categories

| Value | Count | Frequency (%) |
| --- | --- | --- |
| Uppercase Letter | 857 | 100.0% |

| Value | Count | Frequency (%) |
| --- | --- | --- |
| Uppercase Letter | 511 | 100.0% |

| Value | Count | Frequency (%) |
| --- | --- | --- |
| Uppercase Letter | 168 | 100.0% |

#### Most frequent character per category

##### *Uppercase Letter*

| Value | Count | Frequency (%) |
| --- | --- | --- |
| N | 793 | 92.5% |
| S | 64 | 7.5% |

| Value | Count | Frequency (%) |
| --- | --- | --- |
| N | 481 | 94.1% |
| S | 30 | 5.9% |

| Value | Count | Frequency (%) |
| --- | --- | --- |
| N | 165 | 98.2% |
| S | 3 | 1.8% |

#### Most occurring scripts

| Value | Count | Frequency (%) |
| --- | --- | --- |
| Latin | 857 | 100.0% |

| Value | Count | Frequency (%) |
| --- | --- | --- |
| Latin | 511 | 100.0% |

| Value | Count | Frequency (%) |
| --- | --- | --- |
| Latin | 168 | 100.0% |

#### Most frequent character per script

##### *Latin*

| Value | Count | Frequency (%) |
| --- | --- | --- |
| N | 793 | 92.5% |
| S | 64 | 7.5% |

| Value | Count | Frequency (%) |
| --- | --- | --- |
| N | 481 | 94.1% |
| S | 30 | 5.9% |

| Value | Count | Frequency (%) |
| --- | --- | --- |
| N | 165 | 98.2% |
| S | 3 | 1.8% |

#### Most occurring blocks

| Value | Count | Frequency (%) |
| --- | --- | --- |
| ASCII | 857 | 100.0% |

| Value | Count | Frequency (%) |
| --- | --- | --- |
| ASCII | 511 | 100.0% |

| Value | Count | Frequency (%) |
| --- | --- | --- |
| ASCII | 168 | 100.0% |

#### Most frequent character per block

##### *ASCII*

| Value | Count | Frequency (%) |
| --- | --- | --- |
| N | 793 | 92.5% |
| S | 64 | 7.5% |

| Value | Count | Frequency (%) |
| --- | --- | --- |
| N | 481 | 94.1% |
| S | 30 | 5.9% |

| Value | Count | Frequency (%) |
| --- | --- | --- |
| N | 165 | 98.2% |
| S | 3 | 1.8% |

ALCOOLISMO  
Categorical

|
|  |
|
|  |

|  | Cluster 1 | Cluster 2 | Cluster 3 |
| --- | --- | --- | --- |
| Distinct | 2 | 2 | 2 |
| Distinct (%) | 0.2% | 0.4% | 1.2% |
| Missing | 0 | 0 | 0 |
| Missing (%) | 0.0% | 0.0% | 0.0% |
| Memory size | 13.4 KiB | 8.0 KiB | 2.6 KiB |

|  |  |
| --- | --- |
| N | 597 |
| S | 260 |

|  |  |
| --- | --- |
| N | 457 |
| S | 54 |

|  |  |
| --- | --- |
| N | 144 |
| S | 24 |

More details

- Overview
- Categories
- Words
- Characters

Length

|  | Cluster 1 | Cluster 2 | Cluster 3 |
| --- | --- | --- | --- |
| Max length | 1 | 1 | 1 |
| Median length | 1 | 1 | 1 |
| Mean length | 1 | 1 | 1 |
| Min length | 1 | 1 | 1 |

Characters and Unicode

|  | Cluster 1 | Cluster 2 | Cluster 3 |
| --- | --- | --- | --- |
| Total characters | 857 | 511 | 168 |
| Distinct characters | 2 | 2 | 2 |
| Distinct categories | 1 | 1 | 1 ? |
| Distinct scripts | 1 | 1 | 1 ? |
| Distinct blocks | 1 | 1 | 1 ? |

The Unicode Standard assigns character properties to each code point, which can be used to analyse textual variables.

Unique

|  | Cluster 1 | Cluster 2 | Cluster 3 |
| --- | --- | --- | --- |
| Unique | 0 | 0 | 0 ? |
| Unique (%) | 0.0% | 0.0% | 0.0% |

Sample

|  | Cluster 1 | Cluster 2 | Cluster 3 |
| --- | --- | --- | --- |
| 1st row | N | N | N |
| 2nd row | S | S | N |
| 3rd row | N | N | S |
| 4th row | N | N | N |
| 5th row | N | N | N |

#### Common Values

| Value | Count | Frequency (%) |
| --- | --- | --- |
| N | 597 | 69.7% |
| S | 260 | 30.3% |

| Value | Count | Frequency (%) |
| --- | --- | --- |
| N | 457 | 89.4% |
| S | 54 | 10.6% |

| Value | Count | Frequency (%) |
| --- | --- | --- |
| N | 144 | 85.7% |
| S | 24 | 14.3% |

#### Length

xml version="1.0" encoding="utf-8" standalone="no"?2023-08-25T13:07:49.828921image/svg+xmlMatplotlib v3.6.0, https://matplotlib.org/ 

Histogram of lengths of the category

#### Common Values (Plot)

#### Cluster 1

xml version="1.0" encoding="utf-8" standalone="no"?2023-08-25T13:07:49.972078image/svg+xmlMatplotlib v3.6.0, https://matplotlib.org/

#### Cluster 2

xml version="1.0" encoding="utf-8" standalone="no"?2023-08-25T13:07:50.095965image/svg+xmlMatplotlib v3.6.0, https://matplotlib.org/

#### Cluster 3

xml version="1.0" encoding="utf-8" standalone="no"?2023-08-25T13:07:50.218851image/svg+xmlMatplotlib v3.6.0, https://matplotlib.org/

| Value | Count | Frequency (%) |
| --- | --- | --- |
| n | 597 | 69.7% |
| s | 260 | 30.3% |

| Value | Count | Frequency (%) |
| --- | --- | --- |
| n | 457 | 89.4% |
| s | 54 | 10.6% |

| Value | Count | Frequency (%) |
| --- | --- | --- |
| n | 144 | 85.7% |
| s | 24 | 14.3% |

- Characters
- Categories
- Scripts
- Blocks

#### Most occurring characters

| Value | Count | Frequency (%) |
| --- | --- | --- |
| N | 597 | 69.7% |
| S | 260 | 30.3% |

| Value | Count | Frequency (%) |
| --- | --- | --- |
| N | 457 | 89.4% |
| S | 54 | 10.6% |

| Value | Count | Frequency (%) |
| --- | --- | --- |
| N | 144 | 85.7% |
| S | 24 | 14.3% |

#### Most occurring categories

| Value | Count | Frequency (%) |
| --- | --- | --- |
| Uppercase Letter | 857 | 100.0% |

| Value | Count | Frequency (%) |
| --- | --- | --- |
| Uppercase Letter | 511 | 100.0% |

| Value | Count | Frequency (%) |
| --- | --- | --- |
| Uppercase Letter | 168 | 100.0% |

#### Most frequent character per category

##### *Uppercase Letter*

| Value | Count | Frequency (%) |
| --- | --- | --- |
| N | 597 | 69.7% |
| S | 260 | 30.3% |

| Value | Count | Frequency (%) |
| --- | --- | --- |
| N | 457 | 89.4% |
| S | 54 | 10.6% |

| Value | Count | Frequency (%) |
| --- | --- | --- |
| N | 144 | 85.7% |
| S | 24 | 14.3% |

#### Most occurring scripts

| Value | Count | Frequency (%) |
| --- | --- | --- |
| Latin | 857 | 100.0% |

| Value | Count | Frequency (%) |
| --- | --- | --- |
| Latin | 511 | 100.0% |

| Value | Count | Frequency (%) |
| --- | --- | --- |
| Latin | 168 | 100.0% |

#### Most frequent character per script

##### *Latin*

| Value | Count | Frequency (%) |
| --- | --- | --- |
| N | 597 | 69.7% |
| S | 260 | 30.3% |

| Value | Count | Frequency (%) |
| --- | --- | --- |
| N | 457 | 89.4% |
| S | 54 | 10.6% |

| Value | Count | Frequency (%) |
| --- | --- | --- |
| N | 144 | 85.7% |
| S | 24 | 14.3% |

#### Most occurring blocks

| Value | Count | Frequency (%) |
| --- | --- | --- |
| ASCII | 857 | 100.0% |

| Value | Count | Frequency (%) |
| --- | --- | --- |
| ASCII | 511 | 100.0% |

| Value | Count | Frequency (%) |
| --- | --- | --- |
| ASCII | 168 | 100.0% |

#### Most frequent character per block

##### *ASCII*

| Value | Count | Frequency (%) |
| --- | --- | --- |
| N | 597 | 69.7% |
| S | 260 | 30.3% |

| Value | Count | Frequency (%) |
| --- | --- | --- |
| N | 457 | 89.4% |
| S | 54 | 10.6% |

| Value | Count | Frequency (%) |
| --- | --- | --- |
| N | 144 | 85.7% |
| S | 24 | 14.3% |

MENTAL  
Categorical

|
|  |
|
|  |

|  | Cluster 1 | Cluster 2 | Cluster 3 |
| --- | --- | --- | --- |
| Distinct | 2 | 2 | 2 |
| Distinct (%) | 0.2% | 0.4% | 1.2% |
| Missing | 0 | 0 | 0 |
| Missing (%) | 0.0% | 0.0% | 0.0% |
| Memory size | 13.4 KiB | 8.0 KiB | 2.6 KiB |

|  |  |
| --- | --- |
| N | 842 |
| S | 15 |

|  |  |
| --- | --- |
| N | 503 |
| S | 8 |

|  |  |
| --- | --- |
| N | 166 |
| S | 2 |

More details

- Overview
- Categories
- Words
- Characters

Length

|  | Cluster 1 | Cluster 2 | Cluster 3 |
| --- | --- | --- | --- |
| Max length | 1 | 1 | 1 |
| Median length | 1 | 1 | 1 |
| Mean length | 1 | 1 | 1 |
| Min length | 1 | 1 | 1 |

Characters and Unicode

|  | Cluster 1 | Cluster 2 | Cluster 3 |
| --- | --- | --- | --- |
| Total characters | 857 | 511 | 168 |
| Distinct characters | 2 | 2 | 2 |
| Distinct categories | 1 | 1 | 1 ? |
| Distinct scripts | 1 | 1 | 1 ? |
| Distinct blocks | 1 | 1 | 1 ? |

The Unicode Standard assigns character properties to each code point, which can be used to analyse textual variables.

Unique

|  | Cluster 1 | Cluster 2 | Cluster 3 |
| --- | --- | --- | --- |
| Unique | 0 | 0 | 0 ? |
| Unique (%) | 0.0% | 0.0% | 0.0% |

Sample

|  | Cluster 1 | Cluster 2 | Cluster 3 |
| --- | --- | --- | --- |
| 1st row | N | N | N |
| 2nd row | N | N | N |
| 3rd row | N | N | N |
| 4th row | N | N | N |
| 5th row | N | N | N |

#### Common Values

| Value | Count | Frequency (%) |
| --- | --- | --- |
| N | 842 | 98.2% |
| S | 15 | 1.8% |

| Value | Count | Frequency (%) |
| --- | --- | --- |
| N | 503 | 98.4% |
| S | 8 | 1.6% |

| Value | Count | Frequency (%) |
| --- | --- | --- |
| N | 166 | 98.8% |
| S | 2 | 1.2% |

#### Length

xml version="1.0" encoding="utf-8" standalone="no"?2023-08-25T13:07:50.339978image/svg+xmlMatplotlib v3.6.0, https://matplotlib.org/ 

Histogram of lengths of the category

#### Common Values (Plot)

#### Cluster 1

xml version="1.0" encoding="utf-8" standalone="no"?2023-08-25T13:07:50.512551image/svg+xmlMatplotlib v3.6.0, https://matplotlib.org/

#### Cluster 2

xml version="1.0" encoding="utf-8" standalone="no"?2023-08-25T13:07:50.647249image/svg+xmlMatplotlib v3.6.0, https://matplotlib.org/

#### Cluster 3

xml version="1.0" encoding="utf-8" standalone="no"?2023-08-25T13:07:50.778746image/svg+xmlMatplotlib v3.6.0, https://matplotlib.org/

| Value | Count | Frequency (%) |
| --- | --- | --- |
| n | 842 | 98.2% |
| s | 15 | 1.8% |

| Value | Count | Frequency (%) |
| --- | --- | --- |
| n | 503 | 98.4% |
| s | 8 | 1.6% |

| Value | Count | Frequency (%) |
| --- | --- | --- |
| n | 166 | 98.8% |
| s | 2 | 1.2% |

- Characters
- Categories
- Scripts
- Blocks

#### Most occurring characters

| Value | Count | Frequency (%) |
| --- | --- | --- |
| N | 842 | 98.2% |
| S | 15 | 1.8% |

| Value | Count | Frequency (%) |
| --- | --- | --- |
| N | 503 | 98.4% |
| S | 8 | 1.6% |

| Value | Count | Frequency (%) |
| --- | --- | --- |
| N | 166 | 98.8% |
| S | 2 | 1.2% |

#### Most occurring categories

| Value | Count | Frequency (%) |
| --- | --- | --- |
| Uppercase Letter | 857 | 100.0% |

| Value | Count | Frequency (%) |
| --- | --- | --- |
| Uppercase Letter | 511 | 100.0% |

| Value | Count | Frequency (%) |
| --- | --- | --- |
| Uppercase Letter | 168 | 100.0% |

#### Most frequent character per category

##### *Uppercase Letter*

| Value | Count | Frequency (%) |
| --- | --- | --- |
| N | 842 | 98.2% |
| S | 15 | 1.8% |

| Value | Count | Frequency (%) |
| --- | --- | --- |
| N | 503 | 98.4% |
| S | 8 | 1.6% |

| Value | Count | Frequency (%) |
| --- | --- | --- |
| N | 166 | 98.8% |
| S | 2 | 1.2% |

#### Most occurring scripts

| Value | Count | Frequency (%) |
| --- | --- | --- |
| Latin | 857 | 100.0% |

| Value | Count | Frequency (%) |
| --- | --- | --- |
| Latin | 511 | 100.0% |

| Value | Count | Frequency (%) |
| --- | --- | --- |
| Latin | 168 | 100.0% |

#### Most frequent character per script

##### *Latin*

| Value | Count | Frequency (%) |
| --- | --- | --- |
| N | 842 | 98.2% |
| S | 15 | 1.8% |

| Value | Count | Frequency (%) |
| --- | --- | --- |
| N | 503 | 98.4% |
| S | 8 | 1.6% |

| Value | Count | Frequency (%) |
| --- | --- | --- |
| N | 166 | 98.8% |
| S | 2 | 1.2% |

#### Most occurring blocks

| Value | Count | Frequency (%) |
| --- | --- | --- |
| ASCII | 857 | 100.0% |

| Value | Count | Frequency (%) |
| --- | --- | --- |
| ASCII | 511 | 100.0% |

| Value | Count | Frequency (%) |
| --- | --- | --- |
| ASCII | 168 | 100.0% |

#### Most frequent character per block

##### *ASCII*

| Value | Count | Frequency (%) |
| --- | --- | --- |
| N | 842 | 98.2% |
| S | 15 | 1.8% |

| Value | Count | Frequency (%) |
| --- | --- | --- |
| N | 503 | 98.4% |
| S | 8 | 1.6% |

| Value | Count | Frequency (%) |
| --- | --- | --- |
| N | 166 | 98.8% |
| S | 2 | 1.2% |

DROGADICAO  
Categorical

|
|  |
|
|  |

|  | Cluster 1 | Cluster 2 | Cluster 3 |
| --- | --- | --- | --- |
| Distinct | 2 | 2 | 2 |
| Distinct (%) | 0.2% | 0.4% | 1.2% |
| Missing | 0 | 0 | 0 |
| Missing (%) | 0.0% | 0.0% | 0.0% |
| Memory size | 13.4 KiB | 8.0 KiB | 2.6 KiB |

|  |  |
| --- | --- |
| N | 634 |
| S | 223 |

|  |  |
| --- | --- |
| N | 471 |
| S | 40 |

|  |  |
| --- | --- |
| N | 136 |
| S | 32 |

More details

- Overview
- Categories
- Words
- Characters

Length

|  | Cluster 1 | Cluster 2 | Cluster 3 |
| --- | --- | --- | --- |
| Max length | 1 | 1 | 1 |
| Median length | 1 | 1 | 1 |
| Mean length | 1 | 1 | 1 |
| Min length | 1 | 1 | 1 |

Characters and Unicode

|  | Cluster 1 | Cluster 2 | Cluster 3 |
| --- | --- | --- | --- |
| Total characters | 857 | 511 | 168 |
| Distinct characters | 2 | 2 | 2 |
| Distinct categories | 1 | 1 | 1 ? |
| Distinct scripts | 1 | 1 | 1 ? |
| Distinct blocks | 1 | 1 | 1 ? |

The Unicode Standard assigns character properties to each code point, which can be used to analyse textual variables.

Unique

|  | Cluster 1 | Cluster 2 | Cluster 3 |
| --- | --- | --- | --- |
| Unique | 0 | 0 | 0 ? |
| Unique (%) | 0.0% | 0.0% | 0.0% |

Sample

|  | Cluster 1 | Cluster 2 | Cluster 3 |
| --- | --- | --- | --- |
| 1st row | N | N | N |
| 2nd row | N | S | S |
| 3rd row | N | N | S |
| 4th row | S | N | N |
| 5th row | N | N | N |

#### Common Values

| Value | Count | Frequency (%) |
| --- | --- | --- |
| N | 634 | 74.0% |
| S | 223 | 26.0% |

| Value | Count | Frequency (%) |
| --- | --- | --- |
| N | 471 | 92.2% |
| S | 40 | 7.8% |

| Value | Count | Frequency (%) |
| --- | --- | --- |
| N | 136 | 81.0% |
| S | 32 | 19.0% |

#### Length

xml version="1.0" encoding="utf-8" standalone="no"?2023-08-25T13:07:50.901144image/svg+xmlMatplotlib v3.6.0, https://matplotlib.org/ 

Histogram of lengths of the category

#### Common Values (Plot)

#### Cluster 1

xml version="1.0" encoding="utf-8" standalone="no"?2023-08-25T13:07:51.053028image/svg+xmlMatplotlib v3.6.0, https://matplotlib.org/

#### Cluster 2

xml version="1.0" encoding="utf-8" standalone="no"?2023-08-25T13:07:51.180691image/svg+xmlMatplotlib v3.6.0, https://matplotlib.org/

#### Cluster 3

xml version="1.0" encoding="utf-8" standalone="no"?2023-08-25T13:07:51.304111image/svg+xmlMatplotlib v3.6.0, https://matplotlib.org/

| Value | Count | Frequency (%) |
| --- | --- | --- |
| n | 634 | 74.0% |
| s | 223 | 26.0% |

| Value | Count | Frequency (%) |
| --- | --- | --- |
| n | 471 | 92.2% |
| s | 40 | 7.8% |

| Value | Count | Frequency (%) |
| --- | --- | --- |
| n | 136 | 81.0% |
| s | 32 | 19.0% |

- Characters
- Categories
- Scripts
- Blocks

#### Most occurring characters

| Value | Count | Frequency (%) |
| --- | --- | --- |
| N | 634 | 74.0% |
| S | 223 | 26.0% |

| Value | Count | Frequency (%) |
| --- | --- | --- |
| N | 471 | 92.2% |
| S | 40 | 7.8% |

| Value | Count | Frequency (%) |
| --- | --- | --- |
| N | 136 | 81.0% |
| S | 32 | 19.0% |

#### Most occurring categories

| Value | Count | Frequency (%) |
| --- | --- | --- |
| Uppercase Letter | 857 | 100.0% |

| Value | Count | Frequency (%) |
| --- | --- | --- |
| Uppercase Letter | 511 | 100.0% |

| Value | Count | Frequency (%) |
| --- | --- | --- |
| Uppercase Letter | 168 | 100.0% |

#### Most frequent character per category

##### *Uppercase Letter*

| Value | Count | Frequency (%) |
| --- | --- | --- |
| N | 634 | 74.0% |
| S | 223 | 26.0% |

| Value | Count | Frequency (%) |
| --- | --- | --- |
| N | 471 | 92.2% |
| S | 40 | 7.8% |

| Value | Count | Frequency (%) |
| --- | --- | --- |
| N | 136 | 81.0% |
| S | 32 | 19.0% |

#### Most occurring scripts

| Value | Count | Frequency (%) |
| --- | --- | --- |
| Latin | 857 | 100.0% |

| Value | Count | Frequency (%) |
| --- | --- | --- |
| Latin | 511 | 100.0% |

| Value | Count | Frequency (%) |
| --- | --- | --- |
| Latin | 168 | 100.0% |

#### Most frequent character per script

##### *Latin*

| Value | Count | Frequency (%) |
| --- | --- | --- |
| N | 634 | 74.0% |
| S | 223 | 26.0% |

| Value | Count | Frequency (%) |
| --- | --- | --- |
| N | 471 | 92.2% |
| S | 40 | 7.8% |

| Value | Count | Frequency (%) |
| --- | --- | --- |
| N | 136 | 81.0% |
| S | 32 | 19.0% |

#### Most occurring blocks

| Value | Count | Frequency (%) |
| --- | --- | --- |
| ASCII | 857 | 100.0% |

| Value | Count | Frequency (%) |
| --- | --- | --- |
| ASCII | 511 | 100.0% |

| Value | Count | Frequency (%) |
| --- | --- | --- |
| ASCII | 168 | 100.0% |

#### Most frequent character per block

##### *ASCII*

| Value | Count | Frequency (%) |
| --- | --- | --- |
| N | 634 | 74.0% |
| S | 223 | 26.0% |

| Value | Count | Frequency (%) |
| --- | --- | --- |
| N | 471 | 92.2% |
| S | 40 | 7.8% |

| Value | Count | Frequency (%) |
| --- | --- | --- |
| N | 136 | 81.0% |
| S | 32 | 19.0% |

TABAGISMO  
Categorical

|
|  |
|
|  |

|  | Cluster 1 | Cluster 2 | Cluster 3 |
| --- | --- | --- | --- |
| Distinct | 2 | 2 | 2 |
| Distinct (%) | 0.2% | 0.4% | 1.2% |
| Missing | 0 | 0 | 0 |
| Missing (%) | 0.0% | 0.0% | 0.0% |
| Memory size | 13.4 KiB | 8.0 KiB | 2.6 KiB |

|  |  |
| --- | --- |
| N | 614 |
| S | 243 |

|  |  |
| --- | --- |
| N | 463 |
| S | 48 |

|  |  |
| --- | --- |
| N | 154 |
| S | 14 |

More details

- Overview
- Categories
- Words
- Characters

Length

|  | Cluster 1 | Cluster 2 | Cluster 3 |
| --- | --- | --- | --- |
| Max length | 1 | 1 | 1 |
| Median length | 1 | 1 | 1 |
| Mean length | 1 | 1 | 1 |
| Min length | 1 | 1 | 1 |

Characters and Unicode

|  | Cluster 1 | Cluster 2 | Cluster 3 |
| --- | --- | --- | --- |
| Total characters | 857 | 511 | 168 |
| Distinct characters | 2 | 2 | 2 |
| Distinct categories | 1 | 1 | 1 ? |
| Distinct scripts | 1 | 1 | 1 ? |
| Distinct blocks | 1 | 1 | 1 ? |

The Unicode Standard assigns character properties to each code point, which can be used to analyse textual variables.

Unique

|  | Cluster 1 | Cluster 2 | Cluster 3 |
| --- | --- | --- | --- |
| Unique | 0 | 0 | 0 ? |
| Unique (%) | 0.0% | 0.0% | 0.0% |

Sample

|  | Cluster 1 | Cluster 2 | Cluster 3 |
| --- | --- | --- | --- |
| 1st row | N | N | N |
| 2nd row | S | S | N |
| 3rd row | N | N | N |
| 4th row | N | N | N |
| 5th row | S | N | N |

#### Common Values

| Value | Count | Frequency (%) |
| --- | --- | --- |
| N | 614 | 71.6% |
| S | 243 | 28.4% |

| Value | Count | Frequency (%) |
| --- | --- | --- |
| N | 463 | 90.6% |
| S | 48 | 9.4% |

| Value | Count | Frequency (%) |
| --- | --- | --- |
| N | 154 | 91.7% |
| S | 14 | 8.3% |

#### Length

xml version="1.0" encoding="utf-8" standalone="no"?2023-08-25T13:07:51.421243image/svg+xmlMatplotlib v3.6.0, https://matplotlib.org/ 

Histogram of lengths of the category

#### Common Values (Plot)

#### Cluster 1

xml version="1.0" encoding="utf-8" standalone="no"?2023-08-25T13:07:51.567830image/svg+xmlMatplotlib v3.6.0, https://matplotlib.org/

#### Cluster 2

xml version="1.0" encoding="utf-8" standalone="no"?2023-08-25T13:07:51.691633image/svg+xmlMatplotlib v3.6.0, https://matplotlib.org/

#### Cluster 3

xml version="1.0" encoding="utf-8" standalone="no"?2023-08-25T13:07:51.816295image/svg+xmlMatplotlib v3.6.0, https://matplotlib.org/

| Value | Count | Frequency (%) |
| --- | --- | --- |
| n | 614 | 71.6% |
| s | 243 | 28.4% |

| Value | Count | Frequency (%) |
| --- | --- | --- |
| n | 463 | 90.6% |
| s | 48 | 9.4% |

| Value | Count | Frequency (%) |
| --- | --- | --- |
| n | 154 | 91.7% |
| s | 14 | 8.3% |

- Characters
- Categories
- Scripts
- Blocks

#### Most occurring characters

| Value | Count | Frequency (%) |
| --- | --- | --- |
| N | 614 | 71.6% |
| S | 243 | 28.4% |

| Value | Count | Frequency (%) |
| --- | --- | --- |
| N | 463 | 90.6% |
| S | 48 | 9.4% |

| Value | Count | Frequency (%) |
| --- | --- | --- |
| N | 154 | 91.7% |
| S | 14 | 8.3% |

#### Most occurring categories

| Value | Count | Frequency (%) |
| --- | --- | --- |
| Uppercase Letter | 857 | 100.0% |

| Value | Count | Frequency (%) |
| --- | --- | --- |
| Uppercase Letter | 511 | 100.0% |

| Value | Count | Frequency (%) |
| --- | --- | --- |
| Uppercase Letter | 168 | 100.0% |

#### Most frequent character per category

##### *Uppercase Letter*

| Value | Count | Frequency (%) |
| --- | --- | --- |
| N | 614 | 71.6% |
| S | 243 | 28.4% |

| Value | Count | Frequency (%) |
| --- | --- | --- |
| N | 463 | 90.6% |
| S | 48 | 9.4% |

| Value | Count | Frequency (%) |
| --- | --- | --- |
| N | 154 | 91.7% |
| S | 14 | 8.3% |

#### Most occurring scripts

| Value | Count | Frequency (%) |
| --- | --- | --- |
| Latin | 857 | 100.0% |

| Value | Count | Frequency (%) |
| --- | --- | --- |
| Latin | 511 | 100.0% |

| Value | Count | Frequency (%) |
| --- | --- | --- |
| Latin | 168 | 100.0% |

#### Most frequent character per script

##### *Latin*

| Value | Count | Frequency (%) |
| --- | --- | --- |
| N | 614 | 71.6% |
| S | 243 | 28.4% |

| Value | Count | Frequency (%) |
| --- | --- | --- |
| N | 463 | 90.6% |
| S | 48 | 9.4% |

| Value | Count | Frequency (%) |
| --- | --- | --- |
| N | 154 | 91.7% |
| S | 14 | 8.3% |

#### Most occurring blocks

| Value | Count | Frequency (%) |
| --- | --- | --- |
| ASCII | 857 | 100.0% |

| Value | Count | Frequency (%) |
| --- | --- | --- |
| ASCII | 511 | 100.0% |

| Value | Count | Frequency (%) |
| --- | --- | --- |
| ASCII | 168 | 100.0% |

#### Most frequent character per block

##### *ASCII*

| Value | Count | Frequency (%) |
| --- | --- | --- |
| N | 614 | 71.6% |
| S | 243 | 28.4% |

| Value | Count | Frequency (%) |
| --- | --- | --- |
| N | 463 | 90.6% |
| S | 48 | 9.4% |

| Value | Count | Frequency (%) |
| --- | --- | --- |
| N | 154 | 91.7% |
| S | 14 | 8.3% |

motMudEsquema  
Categorical

|
|  |
|
|  |

|  | Cluster 1 | Cluster 2 | Cluster 3 |
| --- | --- | --- | --- |
| Distinct | 3 | 4 | 4 |
| Distinct (%) | 0.4% | 0.8% | 2.4% |
| Missing | 0 | 0 | 0 |
| Missing (%) | 0.0% | 0.0% | 0.0% |
| Memory size | 13.4 KiB | 8.0 KiB | 2.6 KiB |

|  |  |
| --- | --- |
| Nulo | 854 |
| Resistencia Medicamentosa | 2 |
| Intolerancia/Toxicidade | 1 |

|  |  |
| --- | --- |
| Nulo | 503 |
| Intolerancia/Toxicidade | 5 |
| Resistencia Medicamentosa | 2 |
| Outro Motivo | 1 |

|  |  |
| --- | --- |
| Nulo | 159 |
| Intolerancia/Toxicidade | 7 |
| Resistencia Medicamentosa | 1 |
| Outro Motivo | 1 |

More details

- Overview
- Categories
- Words
- Characters

Length

|  | Cluster 1 | Cluster 2 | Cluster 3 |
| --- | --- | --- | --- |
| Max length | 25 | 25 | 25 |
| Median length | 4 | 4 | 4 |
| Mean length | 4.0711785 | 4.2837573 | 4.9642857 |
| Min length | 4 | 4 | 4 |

Characters and Unicode

|  | Cluster 1 | Cluster 2 | Cluster 3 |
| --- | --- | --- | --- |
| Total characters | 3489 | 2189 | 834 |
| Distinct characters | 21 | 23 | 23 |
| Distinct categories | 4 | 4 | 4 ? |
| Distinct scripts | 2 | 2 | 2 ? |
| Distinct blocks | 1 | 1 | 1 ? |

The Unicode Standard assigns character properties to each code point, which can be used to analyse textual variables.

Unique

|  | Cluster 1 | Cluster 2 | Cluster 3 |
| --- | --- | --- | --- |
| Unique | 1 | 1 | 2 ? |
| Unique (%) | 0.1% | 0.2% | 1.2% |

Sample

|  | Cluster 1 | Cluster 2 | Cluster 3 |
| --- | --- | --- | --- |
| 1st row | Nulo | Nulo | Nulo |
| 2nd row | Nulo | Nulo | Nulo |
| 3rd row | Nulo | Nulo | Nulo |
| 4th row | Nulo | Nulo | Nulo |
| 5th row | Nulo | Nulo | Nulo |

#### Common Values

| Value | Count | Frequency (%) |
| --- | --- | --- |
| Nulo | 854 | 99.6% |
| Resistencia Medicamentosa | 2 | 0.2% |
| Intolerancia/Toxicidade | 1 | 0.1% |

| Value | Count | Frequency (%) |
| --- | --- | --- |
| Nulo | 503 | 98.4% |
| Intolerancia/Toxicidade | 5 | 1.0% |
| Resistencia Medicamentosa | 2 | 0.4% |
| Outro Motivo | 1 | 0.2% |

| Value | Count | Frequency (%) |
| --- | --- | --- |
| Nulo | 159 | 94.6% |
| Intolerancia/Toxicidade | 7 | 4.2% |
| Resistencia Medicamentosa | 1 | 0.6% |
| Outro Motivo | 1 | 0.6% |

#### Length

xml version="1.0" encoding="utf-8" standalone="no"?2023-08-25T13:07:51.939001image/svg+xmlMatplotlib v3.6.0, https://matplotlib.org/ 

Histogram of lengths of the category

#### Common Values (Plot)

#### Cluster 1

xml version="1.0" encoding="utf-8" standalone="no"?2023-08-25T13:07:52.090791image/svg+xmlMatplotlib v3.6.0, https://matplotlib.org/

#### Cluster 2

xml version="1.0" encoding="utf-8" standalone="no"?2023-08-25T13:07:52.227052image/svg+xmlMatplotlib v3.6.0, https://matplotlib.org/

#### Cluster 3

xml version="1.0" encoding="utf-8" standalone="no"?2023-08-25T13:07:52.372309image/svg+xmlMatplotlib v3.6.0, https://matplotlib.org/

| Value | Count | Frequency (%) |
| --- | --- | --- |
| nulo | 854 | 99.4% |
| resistencia | 2 | 0.2% |
| medicamentosa | 2 | 0.2% |
| intolerancia/toxicidade | 1 | 0.1% |

| Value | Count | Frequency (%) |
| --- | --- | --- |
| nulo | 503 | 97.9% |
| intolerancia/toxicidade | 5 | 1.0% |
| resistencia | 2 | 0.4% |
| medicamentosa | 2 | 0.4% |
| outro | 1 | 0.2% |
| motivo | 1 | 0.2% |

| Value | Count | Frequency (%) |
| --- | --- | --- |
| nulo | 159 | 93.5% |
| intolerancia/toxicidade | 7 | 4.1% |
| resistencia | 1 | 0.6% |
| medicamentosa | 1 | 0.6% |
| outro | 1 | 0.6% |
| motivo | 1 | 0.6% |

- Characters
- Categories
- Scripts
- Blocks

#### Most occurring characters

| Value | Count | Frequency (%) |
| --- | --- | --- |
| o | 858 | 24.6% |
| l | 855 | 24.5% |
| N | 854 | 24.5% |
| u | 854 | 24.5% |
| e | 10 | 0.3% |
| a | 9 | 0.3% |
| i | 9 | 0.3% |
| n | 6 | 0.2% |
| c | 6 | 0.2% |
| s | 6 | 0.2% |
| Other values (11) | 22 | 0.6% |

| Value | Count | Frequency (%) |
| --- | --- | --- |
| o | 518 | 23.7% |
| l | 508 | 23.2% |
| u | 504 | 23.0% |
| N | 503 | 23.0% |
| i | 22 | 1.0% |
| a | 21 | 1.0% |
| e | 18 | 0.8% |
| n | 14 | 0.6% |
| c | 14 | 0.6% |
| d | 12 | 0.5% |
| Other values (13) | 55 | 2.5% |

| Value | Count | Frequency (%) |
| --- | --- | --- |
| o | 177 | 21.2% |
| l | 166 | 19.9% |
| u | 160 | 19.2% |
| N | 159 | 19.1% |
| i | 25 | 3.0% |
| a | 24 | 2.9% |
| e | 18 | 2.2% |
| n | 16 | 1.9% |
| c | 16 | 1.9% |
| d | 15 | 1.8% |
| Other values (13) | 58 | 7.0% |

#### Most occurring categories

| Value | Count | Frequency (%) |
| --- | --- | --- |
| Lowercase Letter | 2626 | 75.3% |
| Uppercase Letter | 860 | 24.6% |
| Space Separator | 2 | 0.1% |
| Other Punctuation | 1 | < 0.1% |

| Value | Count | Frequency (%) |
| --- | --- | --- |
| Lowercase Letter | 1662 | 75.9% |
| Uppercase Letter | 519 | 23.7% |
| Other Punctuation | 5 | 0.2% |
| Space Separator | 3 | 0.1% |

| Value | Count | Frequency (%) |
| --- | --- | --- |
| Lowercase Letter | 648 | 77.7% |
| Uppercase Letter | 177 | 21.2% |
| Other Punctuation | 7 | 0.8% |
| Space Separator | 2 | 0.2% |

#### Most frequent character per category

##### *Lowercase Letter*

| Value | Count | Frequency (%) |
| --- | --- | --- |
| o | 858 | 32.7% |
| l | 855 | 32.6% |
| u | 854 | 32.5% |
| e | 10 | 0.4% |
| a | 9 | 0.3% |
| i | 9 | 0.3% |
| n | 6 | 0.2% |
| c | 6 | 0.2% |
| s | 6 | 0.2% |
| t | 5 | 0.2% |
| Other values (4) | 8 | 0.3% |

| Value | Count | Frequency (%) |
| --- | --- | --- |
| o | 518 | 31.2% |
| l | 508 | 30.6% |
| u | 504 | 30.3% |
| i | 22 | 1.3% |
| a | 21 | 1.3% |
| e | 18 | 1.1% |
| n | 14 | 0.8% |
| c | 14 | 0.8% |
| d | 12 | 0.7% |
| t | 11 | 0.7% |
| Other values (5) | 20 | 1.2% |

| Value | Count | Frequency (%) |
| --- | --- | --- |
| o | 177 | 27.3% |
| l | 166 | 25.6% |
| u | 160 | 24.7% |
| i | 25 | 3.9% |
| a | 24 | 3.7% |
| e | 18 | 2.8% |
| n | 16 | 2.5% |
| c | 16 | 2.5% |
| d | 15 | 2.3% |
| t | 11 | 1.7% |
| Other values (5) | 20 | 3.1% |

##### *Uppercase Letter*

| Value | Count | Frequency (%) |
| --- | --- | --- |
| N | 854 | 99.3% |
| R | 2 | 0.2% |
| M | 2 | 0.2% |
| I | 1 | 0.1% |
| T | 1 | 0.1% |

| Value | Count | Frequency (%) |
| --- | --- | --- |
| N | 503 | 96.9% |
| T | 5 | 1.0% |
| I | 5 | 1.0% |
| M | 3 | 0.6% |
| R | 2 | 0.4% |
| O | 1 | 0.2% |

| Value | Count | Frequency (%) |
| --- | --- | --- |
| N | 159 | 89.8% |
| T | 7 | 4.0% |
| I | 7 | 4.0% |
| M | 2 | 1.1% |
| R | 1 | 0.6% |
| O | 1 | 0.6% |

##### *Space Separator*

| Value | Count | Frequency (%) |
| --- | --- | --- |
|  | 2 | 100.0% |

| Value | Count | Frequency (%) |
| --- | --- | --- |
|  | 3 | 100.0% |

| Value | Count | Frequency (%) |
| --- | --- | --- |
|  | 2 | 100.0% |

##### *Other Punctuation*

| Value | Count | Frequency (%) |
| --- | --- | --- |
| / | 1 | 100.0% |

| Value | Count | Frequency (%) |
| --- | --- | --- |
| / | 5 | 100.0% |

| Value | Count | Frequency (%) |
| --- | --- | --- |
| / | 7 | 100.0% |

#### Most occurring scripts

| Value | Count | Frequency (%) |
| --- | --- | --- |
| Latin | 3486 | 99.9% |
| Common | 3 | 0.1% |

| Value | Count | Frequency (%) |
| --- | --- | --- |
| Latin | 2181 | 99.6% |
| Common | 8 | 0.4% |

| Value | Count | Frequency (%) |
| --- | --- | --- |
| Latin | 825 | 98.9% |
| Common | 9 | 1.1% |

#### Most frequent character per script

##### *Latin*

| Value | Count | Frequency (%) |
| --- | --- | --- |
| o | 858 | 24.6% |
| l | 855 | 24.5% |
| N | 854 | 24.5% |
| u | 854 | 24.5% |
| e | 10 | 0.3% |
| a | 9 | 0.3% |
| i | 9 | 0.3% |
| n | 6 | 0.2% |
| c | 6 | 0.2% |
| s | 6 | 0.2% |
| Other values (9) | 19 | 0.5% |

| Value | Count | Frequency (%) |
| --- | --- | --- |
| o | 518 | 23.8% |
| l | 508 | 23.3% |
| u | 504 | 23.1% |
| N | 503 | 23.1% |
| i | 22 | 1.0% |
| a | 21 | 1.0% |
| e | 18 | 0.8% |
| n | 14 | 0.6% |
| c | 14 | 0.6% |
| d | 12 | 0.6% |
| Other values (11) | 47 | 2.2% |

| Value | Count | Frequency (%) |
| --- | --- | --- |
| o | 177 | 21.5% |
| l | 166 | 20.1% |
| u | 160 | 19.4% |
| N | 159 | 19.3% |
| i | 25 | 3.0% |
| a | 24 | 2.9% |
| e | 18 | 2.2% |
| n | 16 | 1.9% |
| c | 16 | 1.9% |
| d | 15 | 1.8% |
| Other values (11) | 49 | 5.9% |

##### *Common*

| Value | Count | Frequency (%) |
| --- | --- | --- |
|  | 2 | 66.7% |
| / | 1 | 33.3% |

| Value | Count | Frequency (%) |
| --- | --- | --- |
| / | 5 | 62.5% |
|  | 3 | 37.5% |

| Value | Count | Frequency (%) |
| --- | --- | --- |
| / | 7 | 77.8% |
|  | 2 | 22.2% |

#### Most occurring blocks

| Value | Count | Frequency (%) |
| --- | --- | --- |
| ASCII | 3489 | 100.0% |

| Value | Count | Frequency (%) |
| --- | --- | --- |
| ASCII | 2189 | 100.0% |

| Value | Count | Frequency (%) |
| --- | --- | --- |
| ASCII | 834 | 100.0% |

#### Most frequent character per block

##### *ASCII*

| Value | Count | Frequency (%) |
| --- | --- | --- |
| o | 858 | 24.6% |
| l | 855 | 24.5% |
| N | 854 | 24.5% |
| u | 854 | 24.5% |
| e | 10 | 0.3% |
| a | 9 | 0.3% |
| i | 9 | 0.3% |
| n | 6 | 0.2% |
| c | 6 | 0.2% |
| s | 6 | 0.2% |
| Other values (11) | 22 | 0.6% |

| Value | Count | Frequency (%) |
| --- | --- | --- |
| o | 518 | 23.7% |
| l | 508 | 23.2% |
| u | 504 | 23.0% |
| N | 503 | 23.0% |
| i | 22 | 1.0% |
| a | 21 | 1.0% |
| e | 18 | 0.8% |
| n | 14 | 0.6% |
| c | 14 | 0.6% |
| d | 12 | 0.5% |
| Other values (13) | 55 | 2.5% |

| Value | Count | Frequency (%) |
| --- | --- | --- |
| o | 177 | 21.2% |
| l | 166 | 19.9% |
| u | 160 | 19.2% |
| N | 159 | 19.1% |
| i | 25 | 3.0% |
| a | 24 | 2.9% |
| e | 18 | 2.2% |
| n | 16 | 1.9% |
| c | 16 | 1.9% |
| d | 15 | 1.8% |
| Other values (13) | 58 | 7.0% |

tipoTrat  
Categorical

|
|  |
|
|  |

|  | Cluster 1 | Cluster 2 | Cluster 3 |
| --- | --- | --- | --- |
| Distinct | 2 | 2 | 2 |
| Distinct (%) | 0.2% | 0.4% | 1.2% |
| Missing | 0 | 0 | 0 |
| Missing (%) | 0.0% | 0.0% | 0.0% |
| Memory size | 13.4 KiB | 8.0 KiB | 2.6 KiB |

|  |  |
| --- | --- |
| Supervisionado | 724 |
| Auto-Administrado | 133 |

|  |  |
| --- | --- |
| Supervisionado | 363 |
| Auto-Administrado | 148 |

|  |  |
| --- | --- |
| Auto-Administrado | 125 |
| Supervisionado | 43 |

More details

- Overview
- Categories
- Words
- Characters

Length

|  | Cluster 1 | Cluster 2 | Cluster 3 |
| --- | --- | --- | --- |
| Max length | 17 | 17 | 17 |
| Median length | 14 | 14 | 17 |
| Mean length | 14.465578 | 14.868885 | 16.232143 |
| Min length | 14 | 14 | 14 |

Characters and Unicode

|  | Cluster 1 | Cluster 2 | Cluster 3 |
| --- | --- | --- | --- |
| Total characters | 12397 | 7598 | 2727 |
| Distinct characters | 16 | 16 | 16 |
| Distinct categories | 3 | 3 | 3 ? |
| Distinct scripts | 2 | 2 | 2 ? |
| Distinct blocks | 1 | 1 | 1 ? |

The Unicode Standard assigns character properties to each code point, which can be used to analyse textual variables.

Unique

|  | Cluster 1 | Cluster 2 | Cluster 3 |
| --- | --- | --- | --- |
| Unique | 0 | 0 | 0 ? |
| Unique (%) | 0.0% | 0.0% | 0.0% |

Sample

|  | Cluster 1 | Cluster 2 | Cluster 3 |
| --- | --- | --- | --- |
| 1st row | Supervisionado | Supervisionado | Auto-Administrado |
| 2nd row | Supervisionado | Supervisionado | Supervisionado |
| 3rd row | Supervisionado | Supervisionado | Supervisionado |
| 4th row | Supervisionado | Auto-Administrado | Auto-Administrado |
| 5th row | Supervisionado | Supervisionado | Auto-Administrado |

#### Common Values

| Value | Count | Frequency (%) |
| --- | --- | --- |
| Supervisionado | 724 | 84.5% |
| Auto-Administrado | 133 | 15.5% |

| Value | Count | Frequency (%) |
| --- | --- | --- |
| Supervisionado | 363 | 71.0% |
| Auto-Administrado | 148 | 29.0% |

| Value | Count | Frequency (%) |
| --- | --- | --- |
| Auto-Administrado | 125 | 74.4% |
| Supervisionado | 43 | 25.6% |

#### Length

xml version="1.0" encoding="utf-8" standalone="no"?2023-08-25T13:07:52.517426image/svg+xmlMatplotlib v3.6.0, https://matplotlib.org/ 

Histogram of lengths of the category

#### Common Values (Plot)

#### Cluster 1

xml version="1.0" encoding="utf-8" standalone="no"?2023-08-25T13:07:52.679481image/svg+xmlMatplotlib v3.6.0, https://matplotlib.org/

#### Cluster 2

xml version="1.0" encoding="utf-8" standalone="no"?2023-08-25T13:07:52.808814image/svg+xmlMatplotlib v3.6.0, https://matplotlib.org/

#### Cluster 3

xml version="1.0" encoding="utf-8" standalone="no"?2023-08-25T13:07:52.948515image/svg+xmlMatplotlib v3.6.0, https://matplotlib.org/

| Value | Count | Frequency (%) |
| --- | --- | --- |
| supervisionado | 724 | 84.5% |
| auto-administrado | 133 | 15.5% |

| Value | Count | Frequency (%) |
| --- | --- | --- |
| supervisionado | 363 | 71.0% |
| auto-administrado | 148 | 29.0% |

| Value | Count | Frequency (%) |
| --- | --- | --- |
| auto-administrado | 125 | 74.4% |
| supervisionado | 43 | 25.6% |

- Characters
- Categories
- Scripts
- Blocks

#### Most occurring characters

| Value | Count | Frequency (%) |
| --- | --- | --- |
| i | 1714 | 13.8% |
| o | 1714 | 13.8% |
| d | 990 | 8.0% |
| u | 857 | 6.9% |
| r | 857 | 6.9% |
| s | 857 | 6.9% |
| n | 857 | 6.9% |
| a | 857 | 6.9% |
| S | 724 | 5.8% |
| p | 724 | 5.8% |
| Other values (6) | 2246 | 18.1% |

| Value | Count | Frequency (%) |
| --- | --- | --- |
| i | 1022 | 13.5% |
| o | 1022 | 13.5% |
| d | 659 | 8.7% |
| u | 511 | 6.7% |
| r | 511 | 6.7% |
| s | 511 | 6.7% |
| n | 511 | 6.7% |
| a | 511 | 6.7% |
| S | 363 | 4.8% |
| p | 363 | 4.8% |
| Other values (6) | 1614 | 21.2% |

| Value | Count | Frequency (%) |
| --- | --- | --- |
| o | 336 | 12.3% |
| i | 336 | 12.3% |
| d | 293 | 10.7% |
| A | 250 | 9.2% |
| t | 250 | 9.2% |
| u | 168 | 6.2% |
| n | 168 | 6.2% |
| s | 168 | 6.2% |
| r | 168 | 6.2% |
| a | 168 | 6.2% |
| Other values (6) | 422 | 15.5% |

#### Most occurring categories

| Value | Count | Frequency (%) |
| --- | --- | --- |
| Lowercase Letter | 11274 | 90.9% |
| Uppercase Letter | 990 | 8.0% |
| Dash Punctuation | 133 | 1.1% |

| Value | Count | Frequency (%) |
| --- | --- | --- |
| Lowercase Letter | 6791 | 89.4% |
| Uppercase Letter | 659 | 8.7% |
| Dash Punctuation | 148 | 1.9% |

| Value | Count | Frequency (%) |
| --- | --- | --- |
| Lowercase Letter | 2309 | 84.7% |
| Uppercase Letter | 293 | 10.7% |
| Dash Punctuation | 125 | 4.6% |

#### Most frequent character per category

##### *Lowercase Letter*

| Value | Count | Frequency (%) |
| --- | --- | --- |
| i | 1714 | 15.2% |
| o | 1714 | 15.2% |
| d | 990 | 8.8% |
| u | 857 | 7.6% |
| r | 857 | 7.6% |
| s | 857 | 7.6% |
| n | 857 | 7.6% |
| a | 857 | 7.6% |
| p | 724 | 6.4% |
| e | 724 | 6.4% |
| Other values (3) | 1123 | 10.0% |

| Value | Count | Frequency (%) |
| --- | --- | --- |
| i | 1022 | 15.0% |
| o | 1022 | 15.0% |
| d | 659 | 9.7% |
| u | 511 | 7.5% |
| r | 511 | 7.5% |
| s | 511 | 7.5% |
| n | 511 | 7.5% |
| a | 511 | 7.5% |
| p | 363 | 5.3% |
| e | 363 | 5.3% |
| Other values (3) | 807 | 11.9% |

| Value | Count | Frequency (%) |
| --- | --- | --- |
| o | 336 | 14.6% |
| i | 336 | 14.6% |
| d | 293 | 12.7% |
| t | 250 | 10.8% |
| u | 168 | 7.3% |
| n | 168 | 7.3% |
| s | 168 | 7.3% |
| r | 168 | 7.3% |
| a | 168 | 7.3% |
| m | 125 | 5.4% |
| Other values (3) | 129 | 5.6% |

##### *Uppercase Letter*

| Value | Count | Frequency (%) |
| --- | --- | --- |
| S | 724 | 73.1% |
| A | 266 | 26.9% |

| Value | Count | Frequency (%) |
| --- | --- | --- |
| S | 363 | 55.1% |
| A | 296 | 44.9% |

| Value | Count | Frequency (%) |
| --- | --- | --- |
| A | 250 | 85.3% |
| S | 43 | 14.7% |

##### *Dash Punctuation*

| Value | Count | Frequency (%) |
| --- | --- | --- |
| - | 133 | 100.0% |

| Value | Count | Frequency (%) |
| --- | --- | --- |
| - | 148 | 100.0% |

| Value | Count | Frequency (%) |
| --- | --- | --- |
| - | 125 | 100.0% |

#### Most occurring scripts

| Value | Count | Frequency (%) |
| --- | --- | --- |
| Latin | 12264 | 98.9% |
| Common | 133 | 1.1% |

| Value | Count | Frequency (%) |
| --- | --- | --- |
| Latin | 7450 | 98.1% |
| Common | 148 | 1.9% |

| Value | Count | Frequency (%) |
| --- | --- | --- |
| Latin | 2602 | 95.4% |
| Common | 125 | 4.6% |

#### Most frequent character per script

##### *Latin*

| Value | Count | Frequency (%) |
| --- | --- | --- |
| i | 1714 | 14.0% |
| o | 1714 | 14.0% |
| d | 990 | 8.1% |
| u | 857 | 7.0% |
| r | 857 | 7.0% |
| s | 857 | 7.0% |
| n | 857 | 7.0% |
| a | 857 | 7.0% |
| S | 724 | 5.9% |
| p | 724 | 5.9% |
| Other values (5) | 2113 | 17.2% |

| Value | Count | Frequency (%) |
| --- | --- | --- |
| i | 1022 | 13.7% |
| o | 1022 | 13.7% |
| d | 659 | 8.8% |
| u | 511 | 6.9% |
| r | 511 | 6.9% |
| s | 511 | 6.9% |
| n | 511 | 6.9% |
| a | 511 | 6.9% |
| S | 363 | 4.9% |
| p | 363 | 4.9% |
| Other values (5) | 1466 | 19.7% |

| Value | Count | Frequency (%) |
| --- | --- | --- |
| o | 336 | 12.9% |
| i | 336 | 12.9% |
| d | 293 | 11.3% |
| A | 250 | 9.6% |
| t | 250 | 9.6% |
| u | 168 | 6.5% |
| n | 168 | 6.5% |
| s | 168 | 6.5% |
| r | 168 | 6.5% |
| a | 168 | 6.5% |
| Other values (5) | 297 | 11.4% |

##### *Common*

| Value | Count | Frequency (%) |
| --- | --- | --- |
| - | 133 | 100.0% |

| Value | Count | Frequency (%) |
| --- | --- | --- |
| - | 148 | 100.0% |

| Value | Count | Frequency (%) |
| --- | --- | --- |
| - | 125 | 100.0% |

#### Most occurring blocks

| Value | Count | Frequency (%) |
| --- | --- | --- |
| ASCII | 12397 | 100.0% |

| Value | Count | Frequency (%) |
| --- | --- | --- |
| ASCII | 7598 | 100.0% |

| Value | Count | Frequency (%) |
| --- | --- | --- |
| ASCII | 2727 | 100.0% |

#### Most frequent character per block

##### *ASCII*

| Value | Count | Frequency (%) |
| --- | --- | --- |
| i | 1714 | 13.8% |
| o | 1714 | 13.8% |
| d | 990 | 8.0% |
| u | 857 | 6.9% |
| r | 857 | 6.9% |
| s | 857 | 6.9% |
| n | 857 | 6.9% |
| a | 857 | 6.9% |
| S | 724 | 5.8% |
| p | 724 | 5.8% |
| Other values (6) | 2246 | 18.1% |

| Value | Count | Frequency (%) |
| --- | --- | --- |
| i | 1022 | 13.5% |
| o | 1022 | 13.5% |
| d | 659 | 8.7% |
| u | 511 | 6.7% |
| r | 511 | 6.7% |
| s | 511 | 6.7% |
| n | 511 | 6.7% |
| a | 511 | 6.7% |
| S | 363 | 4.8% |
| p | 363 | 4.8% |
| Other values (6) | 1614 | 21.2% |

| Value | Count | Frequency (%) |
| --- | --- | --- |
| o | 336 | 12.3% |
| i | 336 | 12.3% |
| d | 293 | 10.7% |
| A | 250 | 9.2% |
| t | 250 | 9.2% |
| u | 168 | 6.2% |
| n | 168 | 6.2% |
| s | 168 | 6.2% |
| r | 168 | 6.2% |
| a | 168 | 6.2% |
| Other values (6) | 422 | 15.5% |

idade  
Categorical

|
|  |
|
|  |

|  | Cluster 1 | Cluster 2 | Cluster 3 |
| --- | --- | --- | --- |
| Distinct | 4 | 4 | 4 |
| Distinct (%) | 0.5% | 0.8% | 2.4% |
| Missing | 0 | 0 | 0 |
| Missing (%) | 0.0% | 0.0% | 0.0% |
| Memory size | 13.4 KiB | 8.0 KiB | 2.6 KiB |

|  |  |
| --- | --- |
| 40\_54 | 251 |
| 23\_39 | 223 |
| 0\_22 | 192 |
| Mais de 54 | 191 |

|  |  |
| --- | --- |
| 0\_22 | 144 |
| 23\_39 | 135 |
| 40\_54 | 120 |
| Mais de 54 | 112 |

|  |  |
| --- | --- |
| 40\_54 | 65 |
| 23\_39 | 62 |
| Mais de 54 | 22 |
| 0\_22 | 19 |

More details

- Overview
- Categories
- Words
- Characters

Length

|  | Cluster 1 | Cluster 2 | Cluster 3 |
| --- | --- | --- | --- |
| Max length | 10 | 10 | 10 |
| Median length | 5 | 5 | 5 |
| Mean length | 5.8903151 | 5.81409 | 5.5416667 |
| Min length | 4 | 4 | 4 |

Characters and Unicode

|  | Cluster 1 | Cluster 2 | Cluster 3 |
| --- | --- | --- | --- |
| Total characters | 5048 | 2971 | 931 |
| Distinct characters | 14 | 14 | 14 |
| Distinct categories | 5 | 5 | 5 ? |
| Distinct scripts | 2 | 2 | 2 ? |
| Distinct blocks | 1 | 1 | 1 ? |

The Unicode Standard assigns character properties to each code point, which can be used to analyse textual variables.

Unique

|  | Cluster 1 | Cluster 2 | Cluster 3 |
| --- | --- | --- | --- |
| Unique | 0 | 0 | 0 ? |
| Unique (%) | 0.0% | 0.0% | 0.0% |

Sample

|  | Cluster 1 | Cluster 2 | Cluster 3 |
| --- | --- | --- | --- |
| 1st row | 23\_39 | 40\_54 | 40\_54 |
| 2nd row | 40\_54 | 40\_54 | 23\_39 |
| 3rd row | Mais de 54 | 40\_54 | 23\_39 |
| 4th row | 0\_22 | Mais de 54 | 0\_22 |
| 5th row | 23\_39 | 0\_22 | 23\_39 |

#### Common Values

| Value | Count | Frequency (%) |
| --- | --- | --- |
| 40\_54 | 251 | 29.3% |
| 23\_39 | 223 | 26.0% |
| 0\_22 | 192 | 22.4% |
| Mais de 54 | 191 | 22.3% |

| Value | Count | Frequency (%) |
| --- | --- | --- |
| 0\_22 | 144 | 28.2% |
| 23\_39 | 135 | 26.4% |
| 40\_54 | 120 | 23.5% |
| Mais de 54 | 112 | 21.9% |

| Value | Count | Frequency (%) |
| --- | --- | --- |
| 40\_54 | 65 | 38.7% |
| 23\_39 | 62 | 36.9% |
| Mais de 54 | 22 | 13.1% |
| 0\_22 | 19 | 11.3% |

#### Length

xml version="1.0" encoding="utf-8" standalone="no"?2023-08-25T13:07:53.083824image/svg+xmlMatplotlib v3.6.0, https://matplotlib.org/ 

Histogram of lengths of the category

#### Common Values (Plot)

#### Cluster 1

xml version="1.0" encoding="utf-8" standalone="no"?2023-08-25T13:07:53.255184image/svg+xmlMatplotlib v3.6.0, https://matplotlib.org/

#### Cluster 2

xml version="1.0" encoding="utf-8" standalone="no"?2023-08-25T13:07:53.409375image/svg+xmlMatplotlib v3.6.0, https://matplotlib.org/

#### Cluster 3

xml version="1.0" encoding="utf-8" standalone="no"?2023-08-25T13:07:53.564390image/svg+xmlMatplotlib v3.6.0, https://matplotlib.org/

| Value | Count | Frequency (%) |
| --- | --- | --- |
| 40\_54 | 251 | 20.3% |
| 23\_39 | 223 | 18.0% |
| 0\_22 | 192 | 15.5% |
| mais | 191 | 15.4% |
| de | 191 | 15.4% |
| 54 | 191 | 15.4% |

| Value | Count | Frequency (%) |
| --- | --- | --- |
| 0\_22 | 144 | 19.6% |
| 23\_39 | 135 | 18.4% |
| 40\_54 | 120 | 16.3% |
| mais | 112 | 15.2% |
| de | 112 | 15.2% |
| 54 | 112 | 15.2% |

| Value | Count | Frequency (%) |
| --- | --- | --- |
| 40\_54 | 65 | 30.7% |
| 23\_39 | 62 | 29.2% |
| mais | 22 | 10.4% |
| de | 22 | 10.4% |
| 54 | 22 | 10.4% |
| 0\_22 | 19 | 9.0% |

- Characters
- Categories
- Scripts
- Blocks

#### Most occurring characters

| Value | Count | Frequency (%) |
| --- | --- | --- |
| 4 | 693 | 13.7% |
| \_ | 666 | 13.2% |
| 2 | 607 | 12.0% |
| 3 | 446 | 8.8% |
| 0 | 443 | 8.8% |
| 5 | 442 | 8.8% |
|  | 382 | 7.6% |
| 9 | 223 | 4.4% |
| M | 191 | 3.8% |
| a | 191 | 3.8% |
| Other values (4) | 764 | 15.1% |

| Value | Count | Frequency (%) |
| --- | --- | --- |
| 2 | 423 | 14.2% |
| \_ | 399 | 13.4% |
| 4 | 352 | 11.8% |
| 3 | 270 | 9.1% |
| 0 | 264 | 8.9% |
| 5 | 232 | 7.8% |
|  | 224 | 7.5% |
| 9 | 135 | 4.5% |
| M | 112 | 3.8% |
| a | 112 | 3.8% |
| Other values (4) | 448 | 15.1% |

| Value | Count | Frequency (%) |
| --- | --- | --- |
| 4 | 152 | 16.3% |
| \_ | 146 | 15.7% |
| 3 | 124 | 13.3% |
| 2 | 100 | 10.7% |
| 5 | 87 | 9.3% |
| 0 | 84 | 9.0% |
| 9 | 62 | 6.7% |
|  | 44 | 4.7% |
| M | 22 | 2.4% |
| a | 22 | 2.4% |
| Other values (4) | 88 | 9.5% |

#### Most occurring categories

| Value | Count | Frequency (%) |
| --- | --- | --- |
| Decimal Number | 2854 | 56.5% |
| Lowercase Letter | 955 | 18.9% |
| Connector Punctuation | 666 | 13.2% |
| Space Separator | 382 | 7.6% |
| Uppercase Letter | 191 | 3.8% |

| Value | Count | Frequency (%) |
| --- | --- | --- |
| Decimal Number | 1676 | 56.4% |
| Lowercase Letter | 560 | 18.8% |
| Connector Punctuation | 399 | 13.4% |
| Space Separator | 224 | 7.5% |
| Uppercase Letter | 112 | 3.8% |

| Value | Count | Frequency (%) |
| --- | --- | --- |
| Decimal Number | 609 | 65.4% |
| Connector Punctuation | 146 | 15.7% |
| Lowercase Letter | 110 | 11.8% |
| Space Separator | 44 | 4.7% |
| Uppercase Letter | 22 | 2.4% |

#### Most frequent character per category

##### *Decimal Number*

| Value | Count | Frequency (%) |
| --- | --- | --- |
| 4 | 693 | 24.3% |
| 2 | 607 | 21.3% |
| 3 | 446 | 15.6% |
| 0 | 443 | 15.5% |
| 5 | 442 | 15.5% |
| 9 | 223 | 7.8% |

| Value | Count | Frequency (%) |
| --- | --- | --- |
| 2 | 423 | 25.2% |
| 4 | 352 | 21.0% |
| 3 | 270 | 16.1% |
| 0 | 264 | 15.8% |
| 5 | 232 | 13.8% |
| 9 | 135 | 8.1% |

| Value | Count | Frequency (%) |
| --- | --- | --- |
| 4 | 152 | 25.0% |
| 3 | 124 | 20.4% |
| 2 | 100 | 16.4% |
| 5 | 87 | 14.3% |
| 0 | 84 | 13.8% |
| 9 | 62 | 10.2% |

##### *Connector Punctuation*

| Value | Count | Frequency (%) |
| --- | --- | --- |
| \_ | 666 | 100.0% |

| Value | Count | Frequency (%) |
| --- | --- | --- |
| \_ | 399 | 100.0% |

| Value | Count | Frequency (%) |
| --- | --- | --- |
| \_ | 146 | 100.0% |

##### *Space Separator*

| Value | Count | Frequency (%) |
| --- | --- | --- |
|  | 382 | 100.0% |

| Value | Count | Frequency (%) |
| --- | --- | --- |
|  | 224 | 100.0% |

| Value | Count | Frequency (%) |
| --- | --- | --- |
|  | 44 | 100.0% |

##### *Uppercase Letter*

| Value | Count | Frequency (%) |
| --- | --- | --- |
| M | 191 | 100.0% |

| Value | Count | Frequency (%) |
| --- | --- | --- |
| M | 112 | 100.0% |

| Value | Count | Frequency (%) |
| --- | --- | --- |
| M | 22 | 100.0% |

##### *Lowercase Letter*

| Value | Count | Frequency (%) |
| --- | --- | --- |
| a | 191 | 20.0% |
| i | 191 | 20.0% |
| s | 191 | 20.0% |
| d | 191 | 20.0% |
| e | 191 | 20.0% |

| Value | Count | Frequency (%) |
| --- | --- | --- |
| a | 112 | 20.0% |
| i | 112 | 20.0% |
| s | 112 | 20.0% |
| d | 112 | 20.0% |
| e | 112 | 20.0% |

| Value | Count | Frequency (%) |
| --- | --- | --- |
| a | 22 | 20.0% |
| i | 22 | 20.0% |
| s | 22 | 20.0% |
| d | 22 | 20.0% |
| e | 22 | 20.0% |

#### Most occurring scripts

| Value | Count | Frequency (%) |
| --- | --- | --- |
| Common | 3902 | 77.3% |
| Latin | 1146 | 22.7% |

| Value | Count | Frequency (%) |
| --- | --- | --- |
| Common | 2299 | 77.4% |
| Latin | 672 | 22.6% |

| Value | Count | Frequency (%) |
| --- | --- | --- |
| Common | 799 | 85.8% |
| Latin | 132 | 14.2% |

#### Most frequent character per script

##### *Common*

| Value | Count | Frequency (%) |
| --- | --- | --- |
| 4 | 693 | 17.8% |
| \_ | 666 | 17.1% |
| 2 | 607 | 15.6% |
| 3 | 446 | 11.4% |
| 0 | 443 | 11.4% |
| 5 | 442 | 11.3% |
|  | 382 | 9.8% |
| 9 | 223 | 5.7% |

| Value | Count | Frequency (%) |
| --- | --- | --- |
| 2 | 423 | 18.4% |
| \_ | 399 | 17.4% |
| 4 | 352 | 15.3% |
| 3 | 270 | 11.7% |
| 0 | 264 | 11.5% |
| 5 | 232 | 10.1% |
|  | 224 | 9.7% |
| 9 | 135 | 5.9% |

| Value | Count | Frequency (%) |
| --- | --- | --- |
| 4 | 152 | 19.0% |
| \_ | 146 | 18.3% |
| 3 | 124 | 15.5% |
| 2 | 100 | 12.5% |
| 5 | 87 | 10.9% |
| 0 | 84 | 10.5% |
| 9 | 62 | 7.8% |
|  | 44 | 5.5% |

##### *Latin*

| Value | Count | Frequency (%) |
| --- | --- | --- |
| M | 191 | 16.7% |
| a | 191 | 16.7% |
| i | 191 | 16.7% |
| s | 191 | 16.7% |
| d | 191 | 16.7% |
| e | 191 | 16.7% |

| Value | Count | Frequency (%) |
| --- | --- | --- |
| M | 112 | 16.7% |
| a | 112 | 16.7% |
| i | 112 | 16.7% |
| s | 112 | 16.7% |
| d | 112 | 16.7% |
| e | 112 | 16.7% |

| Value | Count | Frequency (%) |
| --- | --- | --- |
| M | 22 | 16.7% |
| a | 22 | 16.7% |
| i | 22 | 16.7% |
| s | 22 | 16.7% |
| d | 22 | 16.7% |
| e | 22 | 16.7% |

#### Most occurring blocks

| Value | Count | Frequency (%) |
| --- | --- | --- |
| ASCII | 5048 | 100.0% |

| Value | Count | Frequency (%) |
| --- | --- | --- |
| ASCII | 2971 | 100.0% |

| Value | Count | Frequency (%) |
| --- | --- | --- |
| ASCII | 931 | 100.0% |

#### Most frequent character per block

##### *ASCII*

| Value | Count | Frequency (%) |
| --- | --- | --- |
| 4 | 693 | 13.7% |
| \_ | 666 | 13.2% |
| 2 | 607 | 12.0% |
| 3 | 446 | 8.8% |
| 0 | 443 | 8.8% |
| 5 | 442 | 8.8% |
|  | 382 | 7.6% |
| 9 | 223 | 4.4% |
| M | 191 | 3.8% |
| a | 191 | 3.8% |
| Other values (4) | 764 | 15.1% |

| Value | Count | Frequency (%) |
| --- | --- | --- |
| 2 | 423 | 14.2% |
| \_ | 399 | 13.4% |
| 4 | 352 | 11.8% |
| 3 | 270 | 9.1% |
| 0 | 264 | 8.9% |
| 5 | 232 | 7.8% |
|  | 224 | 7.5% |
| 9 | 135 | 4.5% |
| M | 112 | 3.8% |
| a | 112 | 3.8% |
| Other values (4) | 448 | 15.1% |

| Value | Count | Frequency (%) |
| --- | --- | --- |
| 4 | 152 | 16.3% |
| \_ | 146 | 15.7% |
| 3 | 124 | 13.3% |
| 2 | 100 | 10.7% |
| 5 | 87 | 9.3% |
| 0 | 84 | 9.0% |
| 9 | 62 | 6.7% |
|  | 44 | 4.7% |
| M | 22 | 2.4% |
| a | 22 | 2.4% |
| Other values (4) | 88 | 9.5% |

HISTOPATOL  
Categorical

|
|  |
|
|  |

|  | Cluster 1 | Cluster 2 | Cluster 3 |
| --- | --- | --- | --- |
| Distinct | 3 | 3 | 3 |
| Distinct (%) | 0.4% | 0.6% | 1.8% |
| Missing | 0 | 0 | 0 |
| Missing (%) | 0.0% | 0.0% | 0.0% |
| Memory size | 13.4 KiB | 8.0 KiB | 2.6 KiB |

|  |  |
| --- | --- |
| N/realiz | 828 |
| Sugestivo TB | 18 |
| BAAR pos | 11 |

|  |  |
| --- | --- |
| N/realiz | 470 |
| Sugestivo TB | 29 |
| BAAR pos | 12 |

|  |  |
| --- | --- |
| N/realiz | 143 |
| Sugestivo TB | 18 |
| BAAR pos | 7 |

More details

- Overview
- Categories
- Words
- Characters

Length

|  | Cluster 1 | Cluster 2 | Cluster 3 |
| --- | --- | --- | --- |
| Max length | 12 | 12 | 12 |
| Median length | 8 | 8 | 8 |
| Mean length | 8.084014 | 8.2270059 | 8.4285714 |
| Min length | 8 | 8 | 8 |

Characters and Unicode

|  | Cluster 1 | Cluster 2 | Cluster 3 |
| --- | --- | --- | --- |
| Total characters | 6928 | 4204 | 1416 |
| Distinct characters | 21 | 21 | 21 |
| Distinct categories | 4 | 4 | 4 ? |
| Distinct scripts | 2 | 2 | 2 ? |
| Distinct blocks | 1 | 1 | 1 ? |

The Unicode Standard assigns character properties to each code point, which can be used to analyse textual variables.

Unique

|  | Cluster 1 | Cluster 2 | Cluster 3 |
| --- | --- | --- | --- |
| Unique | 0 | 0 | 0 ? |
| Unique (%) | 0.0% | 0.0% | 0.0% |

Sample

|  | Cluster 1 | Cluster 2 | Cluster 3 |
| --- | --- | --- | --- |
| 1st row | N/realiz | N/realiz | BAAR pos |
| 2nd row | N/realiz | N/realiz | Sugestivo TB |
| 3rd row | N/realiz | BAAR pos | N/realiz |
| 4th row | N/realiz | N/realiz | Sugestivo TB |
| 5th row | N/realiz | N/realiz | N/realiz |

#### Common Values

| Value | Count | Frequency (%) |
| --- | --- | --- |
| N/realiz | 828 | 96.6% |
| Sugestivo TB | 18 | 2.1% |
| BAAR pos | 11 | 1.3% |

| Value | Count | Frequency (%) |
| --- | --- | --- |
| N/realiz | 470 | 92.0% |
| Sugestivo TB | 29 | 5.7% |
| BAAR pos | 12 | 2.3% |

| Value | Count | Frequency (%) |
| --- | --- | --- |
| N/realiz | 143 | 85.1% |
| Sugestivo TB | 18 | 10.7% |
| BAAR pos | 7 | 4.2% |

#### Length

xml version="1.0" encoding="utf-8" standalone="no"?2023-08-25T13:07:53.716221image/svg+xmlMatplotlib v3.6.0, https://matplotlib.org/ 

Histogram of lengths of the category

#### Common Values (Plot)

#### Cluster 1

xml version="1.0" encoding="utf-8" standalone="no"?2023-08-25T13:07:53.884694image/svg+xmlMatplotlib v3.6.0, https://matplotlib.org/

#### Cluster 2

xml version="1.0" encoding="utf-8" standalone="no"?2023-08-25T13:07:54.014536image/svg+xmlMatplotlib v3.6.0, https://matplotlib.org/

#### Cluster 3

xml version="1.0" encoding="utf-8" standalone="no"?2023-08-25T13:07:54.147165image/svg+xmlMatplotlib v3.6.0, https://matplotlib.org/

| Value | Count | Frequency (%) |
| --- | --- | --- |
| n/realiz | 828 | 93.5% |
| sugestivo | 18 | 2.0% |
| tb | 18 | 2.0% |
| baar | 11 | 1.2% |
| pos | 11 | 1.2% |

| Value | Count | Frequency (%) |
| --- | --- | --- |
| n/realiz | 470 | 85.1% |
| sugestivo | 29 | 5.3% |
| tb | 29 | 5.3% |
| baar | 12 | 2.2% |
| pos | 12 | 2.2% |

| Value | Count | Frequency (%) |
| --- | --- | --- |
| n/realiz | 143 | 74.1% |
| sugestivo | 18 | 9.3% |
| tb | 18 | 9.3% |
| baar | 7 | 3.6% |
| pos | 7 | 3.6% |

- Characters
- Categories
- Scripts
- Blocks

#### Most occurring characters

| Value | Count | Frequency (%) |
| --- | --- | --- |
| e | 846 | 12.2% |
| i | 846 | 12.2% |
| N | 828 | 12.0% |
| r | 828 | 12.0% |
| a | 828 | 12.0% |
| l | 828 | 12.0% |
| z | 828 | 12.0% |
| / | 828 | 12.0% |
| o | 29 | 0.4% |
| B | 29 | 0.4% |
| Other values (11) | 210 | 3.0% |

| Value | Count | Frequency (%) |
| --- | --- | --- |
| e | 499 | 11.9% |
| i | 499 | 11.9% |
| N | 470 | 11.2% |
| r | 470 | 11.2% |
| a | 470 | 11.2% |
| l | 470 | 11.2% |
| z | 470 | 11.2% |
| / | 470 | 11.2% |
| o | 41 | 1.0% |
| B | 41 | 1.0% |
| Other values (11) | 304 | 7.2% |

| Value | Count | Frequency (%) |
| --- | --- | --- |
| e | 161 | 11.4% |
| i | 161 | 11.4% |
| N | 143 | 10.1% |
| r | 143 | 10.1% |
| a | 143 | 10.1% |
| l | 143 | 10.1% |
| z | 143 | 10.1% |
| / | 143 | 10.1% |
| o | 25 | 1.8% |
| B | 25 | 1.8% |
| Other values (11) | 186 | 13.1% |

#### Most occurring categories

| Value | Count | Frequency (%) |
| --- | --- | --- |
| Lowercase Letter | 5145 | 74.3% |
| Uppercase Letter | 926 | 13.4% |
| Other Punctuation | 828 | 12.0% |
| Space Separator | 29 | 0.4% |

| Value | Count | Frequency (%) |
| --- | --- | --- |
| Lowercase Letter | 3088 | 73.5% |
| Uppercase Letter | 605 | 14.4% |
| Other Punctuation | 470 | 11.2% |
| Space Separator | 41 | 1.0% |

| Value | Count | Frequency (%) |
| --- | --- | --- |
| Lowercase Letter | 1023 | 72.2% |
| Uppercase Letter | 225 | 15.9% |
| Other Punctuation | 143 | 10.1% |
| Space Separator | 25 | 1.8% |

#### Most frequent character per category

##### *Lowercase Letter*

| Value | Count | Frequency (%) |
| --- | --- | --- |
| e | 846 | 16.4% |
| i | 846 | 16.4% |
| r | 828 | 16.1% |
| a | 828 | 16.1% |
| l | 828 | 16.1% |
| z | 828 | 16.1% |
| o | 29 | 0.6% |
| s | 29 | 0.6% |
| g | 18 | 0.3% |
| v | 18 | 0.3% |
| Other values (3) | 47 | 0.9% |

| Value | Count | Frequency (%) |
| --- | --- | --- |
| e | 499 | 16.2% |
| i | 499 | 16.2% |
| r | 470 | 15.2% |
| a | 470 | 15.2% |
| l | 470 | 15.2% |
| z | 470 | 15.2% |
| o | 41 | 1.3% |
| s | 41 | 1.3% |
| g | 29 | 0.9% |
| v | 29 | 0.9% |
| Other values (3) | 70 | 2.3% |

| Value | Count | Frequency (%) |
| --- | --- | --- |
| e | 161 | 15.7% |
| i | 161 | 15.7% |
| r | 143 | 14.0% |
| a | 143 | 14.0% |
| l | 143 | 14.0% |
| z | 143 | 14.0% |
| o | 25 | 2.4% |
| s | 25 | 2.4% |
| g | 18 | 1.8% |
| v | 18 | 1.8% |
| Other values (3) | 43 | 4.2% |

##### *Uppercase Letter*

| Value | Count | Frequency (%) |
| --- | --- | --- |
| N | 828 | 89.4% |
| B | 29 | 3.1% |
| A | 22 | 2.4% |
| T | 18 | 1.9% |
| S | 18 | 1.9% |
| R | 11 | 1.2% |

| Value | Count | Frequency (%) |
| --- | --- | --- |
| N | 470 | 77.7% |
| B | 41 | 6.8% |
| T | 29 | 4.8% |
| S | 29 | 4.8% |
| A | 24 | 4.0% |
| R | 12 | 2.0% |

| Value | Count | Frequency (%) |
| --- | --- | --- |
| N | 143 | 63.6% |
| B | 25 | 11.1% |
| T | 18 | 8.0% |
| S | 18 | 8.0% |
| A | 14 | 6.2% |
| R | 7 | 3.1% |

##### *Other Punctuation*

| Value | Count | Frequency (%) |
| --- | --- | --- |
| / | 828 | 100.0% |

| Value | Count | Frequency (%) |
| --- | --- | --- |
| / | 470 | 100.0% |

| Value | Count | Frequency (%) |
| --- | --- | --- |
| / | 143 | 100.0% |

##### *Space Separator*

| Value | Count | Frequency (%) |
| --- | --- | --- |
|  | 29 | 100.0% |

| Value | Count | Frequency (%) |
| --- | --- | --- |
|  | 41 | 100.0% |

| Value | Count | Frequency (%) |
| --- | --- | --- |
|  | 25 | 100.0% |

#### Most occurring scripts

| Value | Count | Frequency (%) |
| --- | --- | --- |
| Latin | 6071 | 87.6% |
| Common | 857 | 12.4% |

| Value | Count | Frequency (%) |
| --- | --- | --- |
| Latin | 3693 | 87.8% |
| Common | 511 | 12.2% |

| Value | Count | Frequency (%) |
| --- | --- | --- |
| Latin | 1248 | 88.1% |
| Common | 168 | 11.9% |

#### Most frequent character per script

##### *Latin*

| Value | Count | Frequency (%) |
| --- | --- | --- |
| e | 846 | 13.9% |
| i | 846 | 13.9% |
| N | 828 | 13.6% |
| r | 828 | 13.6% |
| a | 828 | 13.6% |
| l | 828 | 13.6% |
| z | 828 | 13.6% |
| o | 29 | 0.5% |
| B | 29 | 0.5% |
| s | 29 | 0.5% |
| Other values (9) | 152 | 2.5% |

| Value | Count | Frequency (%) |
| --- | --- | --- |
| e | 499 | 13.5% |
| i | 499 | 13.5% |
| N | 470 | 12.7% |
| r | 470 | 12.7% |
| a | 470 | 12.7% |
| l | 470 | 12.7% |
| z | 470 | 12.7% |
| o | 41 | 1.1% |
| B | 41 | 1.1% |
| s | 41 | 1.1% |
| Other values (9) | 222 | 6.0% |

| Value | Count | Frequency (%) |
| --- | --- | --- |
| e | 161 | 12.9% |
| i | 161 | 12.9% |
| N | 143 | 11.5% |
| r | 143 | 11.5% |
| a | 143 | 11.5% |
| l | 143 | 11.5% |
| z | 143 | 11.5% |
| o | 25 | 2.0% |
| B | 25 | 2.0% |
| s | 25 | 2.0% |
| Other values (9) | 136 | 10.9% |

##### *Common*

| Value | Count | Frequency (%) |
| --- | --- | --- |
| / | 828 | 96.6% |
|  | 29 | 3.4% |

| Value | Count | Frequency (%) |
| --- | --- | --- |
| / | 470 | 92.0% |
|  | 41 | 8.0% |

| Value | Count | Frequency (%) |
| --- | --- | --- |
| / | 143 | 85.1% |
|  | 25 | 14.9% |

#### Most occurring blocks

| Value | Count | Frequency (%) |
| --- | --- | --- |
| ASCII | 6928 | 100.0% |

| Value | Count | Frequency (%) |
| --- | --- | --- |
| ASCII | 4204 | 100.0% |

| Value | Count | Frequency (%) |
| --- | --- | --- |
| ASCII | 1416 | 100.0% |

#### Most frequent character per block

##### *ASCII*

| Value | Count | Frequency (%) |
| --- | --- | --- |
| e | 846 | 12.2% |
| i | 846 | 12.2% |
| N | 828 | 12.0% |
| r | 828 | 12.0% |
| a | 828 | 12.0% |
| l | 828 | 12.0% |
| z | 828 | 12.0% |
| / | 828 | 12.0% |
| o | 29 | 0.4% |
| B | 29 | 0.4% |
| Other values (11) | 210 | 3.0% |

| Value | Count | Frequency (%) |
| --- | --- | --- |
| e | 499 | 11.9% |
| i | 499 | 11.9% |
| N | 470 | 11.2% |
| r | 470 | 11.2% |
| a | 470 | 11.2% |
| l | 470 | 11.2% |
| z | 470 | 11.2% |
| / | 470 | 11.2% |
| o | 41 | 1.0% |
| B | 41 | 1.0% |
| Other values (11) | 304 | 7.2% |

| Value | Count | Frequency (%) |
| --- | --- | --- |
| e | 161 | 11.4% |
| i | 161 | 11.4% |
| N | 143 | 10.1% |
| r | 143 | 10.1% |
| a | 143 | 10.1% |
| l | 143 | 10.1% |
| z | 143 | 10.1% |
| / | 143 | 10.1% |
| o | 25 | 1.8% |
| B | 25 | 1.8% |
| Other values (11) | 186 | 13.1% |

Status\_Resistencia  
Categorical

|
|  |
|
|  |

|  | Cluster 1 | Cluster 2 | Cluster 3 |
| --- | --- | --- | --- |
| Distinct | 2 | 2 | 2 |
| Distinct (%) | 0.2% | 0.4% | 1.2% |
| Missing | 0 | 0 | 0 |
| Missing (%) | 0.0% | 0.0% | 0.0% |
| Memory size | 13.4 KiB | 8.0 KiB | 2.6 KiB |

|  |  |
| --- | --- |
| 1 | 613 |
| 0 | 244 |

|  |  |
| --- | --- |
| 0 | 458 |
| 1 | 53 |

|  |  |
| --- | --- |
| 1 | 102 |
| 0 | 66 |

More details

- Overview
- Categories
- Words
- Characters

Length

|  | Cluster 1 | Cluster 2 | Cluster 3 |
| --- | --- | --- | --- |
| Max length | 1 | 1 | 1 |
| Median length | 1 | 1 | 1 |
| Mean length | 1 | 1 | 1 |
| Min length | 1 | 1 | 1 |

Characters and Unicode

|  | Cluster 1 | Cluster 2 | Cluster 3 |
| --- | --- | --- | --- |
| Total characters | 857 | 511 | 168 |
| Distinct characters | 2 | 2 | 2 |
| Distinct categories | 1 | 1 | 1 ? |
| Distinct scripts | 1 | 1 | 1 ? |
| Distinct blocks | 1 | 1 | 1 ? |

The Unicode Standard assigns character properties to each code point, which can be used to analyse textual variables.

Unique

|  | Cluster 1 | Cluster 2 | Cluster 3 |
| --- | --- | --- | --- |
| Unique | 0 | 0 | 0 ? |
| Unique (%) | 0.0% | 0.0% | 0.0% |

Sample

|  | Cluster 1 | Cluster 2 | Cluster 3 |
| --- | --- | --- | --- |
| 1st row | 1 | 0 | 1 |
| 2nd row | 1 | 0 | 0 |
| 3rd row | 1 | 0 | 0 |
| 4th row | 1 | 0 | 0 |
| 5th row | 1 | 0 | 1 |

#### Common Values

| Value | Count | Frequency (%) |
| --- | --- | --- |
| 1 | 613 | 71.5% |
| 0 | 244 | 28.5% |

| Value | Count | Frequency (%) |
| --- | --- | --- |
| 0 | 458 | 89.6% |
| 1 | 53 | 10.4% |

| Value | Count | Frequency (%) |
| --- | --- | --- |
| 1 | 102 | 60.7% |
| 0 | 66 | 39.3% |

#### Length

xml version="1.0" encoding="utf-8" standalone="no"?2023-08-25T13:07:54.270730image/svg+xmlMatplotlib v3.6.0, https://matplotlib.org/ 

Histogram of lengths of the category

#### Common Values (Plot)

#### Cluster 1

xml version="1.0" encoding="utf-8" standalone="no"?2023-08-25T13:07:54.409960image/svg+xmlMatplotlib v3.6.0, https://matplotlib.org/

#### Cluster 2

xml version="1.0" encoding="utf-8" standalone="no"?2023-08-25T13:07:54.531758image/svg+xmlMatplotlib v3.6.0, https://matplotlib.org/

#### Cluster 3

xml version="1.0" encoding="utf-8" standalone="no"?2023-08-25T13:07:54.655586image/svg+xmlMatplotlib v3.6.0, https://matplotlib.org/

| Value | Count | Frequency (%) |
| --- | --- | --- |
| 1 | 613 | 71.5% |
| 0 | 244 | 28.5% |

| Value | Count | Frequency (%) |
| --- | --- | --- |
| 0 | 458 | 89.6% |
| 1 | 53 | 10.4% |

| Value | Count | Frequency (%) |
| --- | --- | --- |
| 1 | 102 | 60.7% |
| 0 | 66 | 39.3% |

- Characters
- Categories
- Scripts
- Blocks

#### Most occurring characters

| Value | Count | Frequency (%) |
| --- | --- | --- |
| 1 | 613 | 71.5% |
| 0 | 244 | 28.5% |

| Value | Count | Frequency (%) |
| --- | --- | --- |
| 0 | 458 | 89.6% |
| 1 | 53 | 10.4% |

| Value | Count | Frequency (%) |
| --- | --- | --- |
| 1 | 102 | 60.7% |
| 0 | 66 | 39.3% |

#### Most occurring categories

| Value | Count | Frequency (%) |
| --- | --- | --- |
| Decimal Number | 857 | 100.0% |

| Value | Count | Frequency (%) |
| --- | --- | --- |
| Decimal Number | 511 | 100.0% |

| Value | Count | Frequency (%) |
| --- | --- | --- |
| Decimal Number | 168 | 100.0% |

#### Most frequent character per category

##### *Decimal Number*

| Value | Count | Frequency (%) |
| --- | --- | --- |
| 1 | 613 | 71.5% |
| 0 | 244 | 28.5% |

| Value | Count | Frequency (%) |
| --- | --- | --- |
| 0 | 458 | 89.6% |
| 1 | 53 | 10.4% |

| Value | Count | Frequency (%) |
| --- | --- | --- |
| 1 | 102 | 60.7% |
| 0 | 66 | 39.3% |

#### Most occurring scripts

| Value | Count | Frequency (%) |
| --- | --- | --- |
| Common | 857 | 100.0% |

| Value | Count | Frequency (%) |
| --- | --- | --- |
| Common | 511 | 100.0% |

| Value | Count | Frequency (%) |
| --- | --- | --- |
| Common | 168 | 100.0% |

#### Most frequent character per script

##### *Common*

| Value | Count | Frequency (%) |
| --- | --- | --- |
| 1 | 613 | 71.5% |
| 0 | 244 | 28.5% |

| Value | Count | Frequency (%) |
| --- | --- | --- |
| 0 | 458 | 89.6% |
| 1 | 53 | 10.4% |

| Value | Count | Frequency (%) |
| --- | --- | --- |
| 1 | 102 | 60.7% |
| 0 | 66 | 39.3% |

#### Most occurring blocks

| Value | Count | Frequency (%) |
| --- | --- | --- |
| ASCII | 857 | 100.0% |

| Value | Count | Frequency (%) |
| --- | --- | --- |
| ASCII | 511 | 100.0% |

| Value | Count | Frequency (%) |
| --- | --- | --- |
| ASCII | 168 | 100.0% |

#### Most frequent character per block

##### *ASCII*

| Value | Count | Frequency (%) |
| --- | --- | --- |
| 1 | 613 | 71.5% |
| 0 | 244 | 28.5% |

| Value | Count | Frequency (%) |
| --- | --- | --- |
| 0 | 458 | 89.6% |
| 1 | 53 | 10.4% |

| Value | Count | Frequency (%) |
| --- | --- | --- |
| 1 | 102 | 60.7% |
| 0 | 66 | 39.3% |

Cluster  
Categorical

|
|  |
|
|  |

|  | Cluster 1 | Cluster 2 | Cluster 3 |
| --- | --- | --- | --- |
| Distinct | 1 | 1 | 1 |
| Distinct (%) | 0.1% | 0.2% | 0.6% |
| Missing | 0 | 0 | 0 |
| Missing (%) | 0.0% | 0.0% | 0.0% |
| Memory size | 13.4 KiB | 8.0 KiB | 2.6 KiB |

|  |  |
| --- | --- |
| 0 | 857 |

|  |  |
| --- | --- |
| 1 | 511 |

|  |  |
| --- | --- |
| 2 | 168 |

More details

- Overview
- Categories
- Words
- Characters

Length

|  | Cluster 1 | Cluster 2 | Cluster 3 |
| --- | --- | --- | --- |
| Max length | 1 | 1 | 1 |
| Median length | 1 | 1 | 1 |
| Mean length | 1 | 1 | 1 |
| Min length | 1 | 1 | 1 |

Characters and Unicode

|  | Cluster 1 | Cluster 2 | Cluster 3 |
| --- | --- | --- | --- |
| Total characters | 857 | 511 | 168 |
| Distinct characters | 1 | 1 | 1 |
| Distinct categories | 1 | 1 | 1 ? |
| Distinct scripts | 1 | 1 | 1 ? |
| Distinct blocks | 1 | 1 | 1 ? |

The Unicode Standard assigns character properties to each code point, which can be used to analyse textual variables.

Unique

|  | Cluster 1 | Cluster 2 | Cluster 3 |
| --- | --- | --- | --- |
| Unique | 0 | 0 | 0 ? |
| Unique (%) | 0.0% | 0.0% | 0.0% |

Sample

|  | Cluster 1 | Cluster 2 | Cluster 3 |
| --- | --- | --- | --- |
| 1st row | 0 | 1 | 2 |
| 2nd row | 0 | 1 | 2 |
| 3rd row | 0 | 1 | 2 |
| 4th row | 0 | 1 | 2 |
| 5th row | 0 | 1 | 2 |

#### Common Values

| Value | Count | Frequency (%) |
| --- | --- | --- |
| 0 | 857 | 100.0% |

| Value | Count | Frequency (%) |
| --- | --- | --- |
| 1 | 511 | 100.0% |

| Value | Count | Frequency (%) |
| --- | --- | --- |
| 2 | 168 | 100.0% |

#### Length

xml version="1.0" encoding="utf-8" standalone="no"?2023-08-25T13:07:54.770405image/svg+xmlMatplotlib v3.6.0, https://matplotlib.org/ 

Histogram of lengths of the category

#### Common Values (Plot)

#### Cluster 1

xml version="1.0" encoding="utf-8" standalone="no"?2023-08-25T13:07:54.906160image/svg+xmlMatplotlib v3.6.0, https://matplotlib.org/

#### Cluster 2

xml version="1.0" encoding="utf-8" standalone="no"?2023-08-25T13:07:55.013680image/svg+xmlMatplotlib v3.6.0, https://matplotlib.org/

#### Cluster 3

xml version="1.0" encoding="utf-8" standalone="no"?2023-08-25T13:07:55.122796image/svg+xmlMatplotlib v3.6.0, https://matplotlib.org/

| Value | Count | Frequency (%) |
| --- | --- | --- |
| 0 | 857 | 100.0% |

| Value | Count | Frequency (%) |
| --- | --- | --- |
| 1 | 511 | 100.0% |

| Value | Count | Frequency (%) |
| --- | --- | --- |
| 2 | 168 | 100.0% |

- Characters
- Categories
- Scripts
- Blocks

#### Most occurring characters

| Value | Count | Frequency (%) |
| --- | --- | --- |
| 0 | 857 | 100.0% |

| Value | Count | Frequency (%) |
| --- | --- | --- |
| 1 | 511 | 100.0% |

| Value | Count | Frequency (%) |
| --- | --- | --- |
| 2 | 168 | 100.0% |

#### Most occurring categories

| Value | Count | Frequency (%) |
| --- | --- | --- |
| Decimal Number | 857 | 100.0% |

| Value | Count | Frequency (%) |
| --- | --- | --- |
| Decimal Number | 511 | 100.0% |

| Value | Count | Frequency (%) |
| --- | --- | --- |
| Decimal Number | 168 | 100.0% |

#### Most frequent character per category

##### *Decimal Number*

| Value | Count | Frequency (%) |
| --- | --- | --- |
| 0 | 857 | 100.0% |

| Value | Count | Frequency (%) |
| --- | --- | --- |
| 1 | 511 | 100.0% |

| Value | Count | Frequency (%) |
| --- | --- | --- |
| 2 | 168 | 100.0% |

#### Most occurring scripts

| Value | Count | Frequency (%) |
| --- | --- | --- |
| Common | 857 | 100.0% |

| Value | Count | Frequency (%) |
| --- | --- | --- |
| Common | 511 | 100.0% |

| Value | Count | Frequency (%) |
| --- | --- | --- |
| Common | 168 | 100.0% |

#### Most frequent character per script

##### *Common*

| Value | Count | Frequency (%) |
| --- | --- | --- |
| 0 | 857 | 100.0% |

| Value | Count | Frequency (%) |
| --- | --- | --- |
| 1 | 511 | 100.0% |

| Value | Count | Frequency (%) |
| --- | --- | --- |
| 2 | 168 | 100.0% |

#### Most occurring blocks

| Value | Count | Frequency (%) |
| --- | --- | --- |
| ASCII | 857 | 100.0% |

| Value | Count | Frequency (%) |
| --- | --- | --- |
| ASCII | 511 | 100.0% |

| Value | Count | Frequency (%) |
| --- | --- | --- |
| ASCII | 168 | 100.0% |

#### Most frequent character per block

##### *ASCII*

| Value | Count | Frequency (%) |
| --- | --- | --- |
| 0 | 857 | 100.0% |

| Value | Count | Frequency (%) |
| --- | --- | --- |
| 1 | 511 | 100.0% |

| Value | Count | Frequency (%) |
| --- | --- | --- |
| 2 | 168 | 100.0% |

# Correlations

- Auto

- Heatmap
- Table

#### Cluster 1

xml version="1.0" encoding="utf-8" standalone="no"?2023-08-25T13:07:55.255007image/svg+xmlMatplotlib v3.6.0, https://matplotlib.org/

#### Cluster 2

xml version="1.0" encoding="utf-8" standalone="no"?2023-08-25T13:07:55.587618image/svg+xmlMatplotlib v3.6.0, https://matplotlib.org/

#### Cluster 3

xml version="1.0" encoding="utf-8" standalone="no"?2023-08-25T13:07:55.924348image/svg+xmlMatplotlib v3.6.0, https://matplotlib.org/

#### Cluster 1

|  | racaCor | faixaEtaria | sexo | ESCOLARID | TIPOCUP | sitAtual | tipoCaso | FORMACLIN1 | classif | descoberta | bac | BACOUTRO | cultEsc | RX | NECROP | hiv | aids | DIABETES | ALCOOLISMO | MENTAL | DROGADICAO | TABAGISMO | motMudEsquema | tipoTrat | idade | HISTOPATOL | Status\_Resistencia |
| --- | --- | --- | --- | --- | --- | --- | --- | --- | --- | --- | --- | --- | --- | --- | --- | --- | --- | --- | --- | --- | --- | --- | --- | --- | --- | --- | --- |
| racaCor | 1.000 | 0.090 | 0.174 | 0.063 | 0.081 | 0.106 | 0.053 | 0.000 | 0.000 | 0.013 | 0.036 | 0.000 | 0.082 | 0.065 | 0.000 | 0.015 | 0.046 | 0.000 | 0.042 | 0.000 | 0.000 | 0.058 | 0.000 | 0.000 | 0.056 | 0.000 | 0.128 |
| faixaEtaria | 0.090 | 1.000 | 0.097 | 0.128 | 0.325 | 0.132 | 0.000 | 0.120 | 0.088 | 0.072 | 0.033 | 0.111 | 0.015 | 0.000 | 0.000 | 0.099 | 0.059 | 0.235 | 0.251 | 0.000 | 0.302 | 0.168 | 0.000 | 0.060 | 0.836 | 0.136 | 0.000 |
| sexo | 0.174 | 0.097 | 1.000 | 0.000 | 0.472 | 0.000 | 0.025 | 0.030 | 0.086 | 0.100 | 0.000 | 0.000 | 0.184 | 0.000 | 0.000 | 0.069 | 0.051 | 0.000 | 0.149 | 0.094 | 0.000 | 0.016 | 0.000 | 0.000 | 0.084 | 0.024 | 0.220 |
| ESCOLARID | 0.063 | 0.128 | 0.000 | 1.000 | 0.124 | 0.000 | 0.000 | 0.048 | 0.045 | 0.000 | 0.000 | 0.000 | 0.040 | 0.038 | 0.000 | 0.039 | 0.000 | 0.000 | 0.159 | 0.139 | 0.079 | 0.092 | 0.000 | 0.100 | 0.103 | 0.047 | 0.036 |
| TIPOCUP | 0.081 | 0.325 | 0.472 | 0.124 | 1.000 | 0.141 | 0.028 | 0.070 | 0.013 | 0.000 | 0.039 | 0.078 | 0.076 | 0.000 | 0.000 | 0.060 | 0.116 | 0.104 | 0.182 | 0.122 | 0.251 | 0.046 | 0.000 | 0.080 | 0.221 | 0.078 | 0.138 |
| sitAtual | 0.106 | 0.132 | 0.000 | 0.000 | 0.141 | 1.000 | 0.092 | 0.048 | 0.000 | 0.069 | 0.000 | 0.057 | 0.083 | 0.057 | 0.000 | 0.183 | 0.047 | 0.010 | 0.075 | 0.000 | 0.214 | 0.000 | 0.000 | 0.078 | 0.135 | 0.000 | 0.000 |
| tipoCaso | 0.053 | 0.000 | 0.025 | 0.000 | 0.028 | 0.092 | 1.000 | 0.058 | 0.000 | 0.000 | 0.015 | 0.000 | 0.042 | 0.000 | 0.000 | 0.072 | 0.126 | 0.000 | 0.034 | 0.000 | 0.000 | 0.074 | 0.000 | 0.067 | 0.050 | 0.000 | 0.047 |
| FORMACLIN1 | 0.000 | 0.120 | 0.030 | 0.048 | 0.070 | 0.048 | 0.058 | 1.000 | 0.813 | 0.022 | 0.233 | 0.212 | 0.211 | 0.228 | 0.000 | 0.106 | 0.000 | 0.086 | 0.000 | 0.000 | 0.060 | 0.000 | 0.000 | 0.095 | 0.052 | 0.199 | 0.116 |
| classif | 0.000 | 0.088 | 0.086 | 0.045 | 0.013 | 0.000 | 0.000 | 0.813 | 1.000 | 0.104 | 0.234 | 0.213 | 0.211 | 0.170 | 0.000 | 0.080 | 0.046 | 0.000 | 0.000 | 0.000 | 0.129 | 0.066 | 0.000 | 0.000 | 0.000 | 0.249 | 0.116 |
| descoberta | 0.013 | 0.072 | 0.100 | 0.000 | 0.000 | 0.069 | 0.000 | 0.022 | 0.104 | 1.000 | 0.103 | 0.134 | 0.139 | 0.089 | 0.067 | 0.013 | 0.035 | 0.000 | 0.086 | 0.056 | 0.041 | 0.123 | 0.000 | 0.104 | 0.042 | 0.093 | 0.082 |
| bac | 0.036 | 0.033 | 0.000 | 0.000 | 0.039 | 0.000 | 0.015 | 0.233 | 0.234 | 0.103 | 1.000 | 0.175 | 0.169 | 0.055 | 0.000 | 0.062 | 0.097 | 0.037 | 0.000 | 0.000 | 0.117 | 0.059 | 0.000 | 0.000 | 0.000 | 0.097 | 0.162 |
| BACOUTRO | 0.000 | 0.111 | 0.000 | 0.000 | 0.078 | 0.057 | 0.000 | 0.212 | 0.213 | 0.134 | 0.175 | 1.000 | 0.135 | 0.099 | 0.000 | 0.000 | 0.000 | 0.113 | 0.092 | 0.000 | 0.089 | 0.000 | 0.000 | 0.089 | 0.063 | 0.258 | 0.018 |
| cultEsc | 0.082 | 0.015 | 0.184 | 0.040 | 0.076 | 0.083 | 0.042 | 0.211 | 0.211 | 0.139 | 0.169 | 0.135 | 1.000 | 0.000 | 0.000 | 0.032 | 0.055 | 0.000 | 0.099 | 0.046 | 0.120 | 0.124 | 0.037 | 0.098 | 0.000 | 0.063 | 0.432 |
| RX | 0.065 | 0.000 | 0.000 | 0.038 | 0.000 | 0.057 | 0.000 | 0.228 | 0.170 | 0.089 | 0.055 | 0.099 | 0.000 | 1.000 | 0.000 | 0.080 | 0.093 | 0.000 | 0.009 | 0.011 | 0.000 | 0.063 | 0.000 | 0.000 | 0.000 | 0.071 | 0.047 |
| NECROP | 0.000 | 0.000 | 0.000 | 0.000 | 0.000 | 0.000 | 0.000 | 0.000 | 0.000 | 0.067 | 0.000 | 0.000 | 0.000 | 0.000 | 1.000 | 0.000 | 0.000 | 0.000 | 0.000 | 0.000 | 0.000 | 0.000 | 0.000 | 0.000 | 0.038 | 0.000 | 0.000 |
| hiv | 0.015 | 0.099 | 0.069 | 0.039 | 0.060 | 0.183 | 0.072 | 0.106 | 0.080 | 0.013 | 0.062 | 0.000 | 0.032 | 0.080 | 0.000 | 1.000 | 0.883 | 0.064 | 0.018 | 0.000 | 0.065 | 0.083 | 0.037 | 0.168 | 0.080 | 0.000 | 0.043 |
| aids | 0.046 | 0.059 | 0.051 | 0.000 | 0.116 | 0.047 | 0.126 | 0.000 | 0.046 | 0.035 | 0.097 | 0.000 | 0.055 | 0.093 | 0.000 | 0.883 | 1.000 | 0.051 | 0.000 | 0.000 | 0.000 | 0.022 | 0.000 | 0.048 | 0.086 | 0.000 | 0.040 |
| DIABETES | 0.000 | 0.235 | 0.000 | 0.000 | 0.104 | 0.010 | 0.000 | 0.086 | 0.000 | 0.000 | 0.037 | 0.113 | 0.000 | 0.000 | 0.000 | 0.064 | 0.051 | 1.000 | 0.057 | 0.073 | 0.139 | 0.011 | 0.062 | 0.086 | 0.240 | 0.022 | 0.031 |
| ALCOOLISMO | 0.042 | 0.251 | 0.149 | 0.159 | 0.182 | 0.075 | 0.034 | 0.000 | 0.000 | 0.086 | 0.000 | 0.092 | 0.099 | 0.009 | 0.000 | 0.018 | 0.000 | 0.057 | 1.000 | 0.020 | 0.281 | 0.324 | 0.028 | 0.000 | 0.243 | 0.000 | 0.093 |
| MENTAL | 0.000 | 0.000 | 0.094 | 0.139 | 0.122 | 0.000 | 0.000 | 0.000 | 0.000 | 0.056 | 0.000 | 0.000 | 0.046 | 0.011 | 0.000 | 0.000 | 0.000 | 0.073 | 0.020 | 1.000 | 0.000 | 0.005 | 0.171 | 0.000 | 0.000 | 0.000 | 0.000 |
| DROGADICAO | 0.000 | 0.302 | 0.000 | 0.079 | 0.251 | 0.214 | 0.000 | 0.060 | 0.129 | 0.041 | 0.117 | 0.089 | 0.120 | 0.000 | 0.000 | 0.065 | 0.000 | 0.139 | 0.281 | 0.000 | 1.000 | 0.187 | 0.000 | 0.082 | 0.281 | 0.024 | 0.113 |
| TABAGISMO | 0.058 | 0.168 | 0.016 | 0.092 | 0.046 | 0.000 | 0.074 | 0.000 | 0.066 | 0.123 | 0.059 | 0.000 | 0.124 | 0.063 | 0.000 | 0.083 | 0.022 | 0.011 | 0.324 | 0.005 | 0.187 | 1.000 | 0.000 | 0.000 | 0.132 | 0.030 | 0.196 |
| motMudEsquema | 0.000 | 0.000 | 0.000 | 0.000 | 0.000 | 0.000 | 0.000 | 0.000 | 0.000 | 0.000 | 0.000 | 0.000 | 0.037 | 0.000 | 0.000 | 0.037 | 0.000 | 0.062 | 0.028 | 0.171 | 0.000 | 0.000 | 1.000 | 0.000 | 0.000 | 0.000 | 0.000 |
| tipoTrat | 0.000 | 0.060 | 0.000 | 0.100 | 0.080 | 0.078 | 0.067 | 0.095 | 0.000 | 0.104 | 0.000 | 0.089 | 0.098 | 0.000 | 0.000 | 0.168 | 0.048 | 0.086 | 0.000 | 0.000 | 0.082 | 0.000 | 0.000 | 1.000 | 0.000 | 0.020 | 0.000 |
| idade | 0.056 | 0.836 | 0.084 | 0.103 | 0.221 | 0.135 | 0.050 | 0.052 | 0.000 | 0.042 | 0.000 | 0.063 | 0.000 | 0.000 | 0.038 | 0.080 | 0.086 | 0.240 | 0.243 | 0.000 | 0.281 | 0.132 | 0.000 | 0.000 | 1.000 | 0.000 | 0.000 |
| HISTOPATOL | 0.000 | 0.136 | 0.024 | 0.047 | 0.078 | 0.000 | 0.000 | 0.199 | 0.249 | 0.093 | 0.097 | 0.258 | 0.063 | 0.071 | 0.000 | 0.000 | 0.000 | 0.022 | 0.000 | 0.000 | 0.024 | 0.030 | 0.000 | 0.020 | 0.000 | 1.000 | 0.000 |
| Status\_Resistencia | 0.128 | 0.000 | 0.220 | 0.036 | 0.138 | 0.000 | 0.047 | 0.116 | 0.116 | 0.082 | 0.162 | 0.018 | 0.432 | 0.047 | 0.000 | 0.043 | 0.040 | 0.031 | 0.093 | 0.000 | 0.113 | 0.196 | 0.000 | 0.000 | 0.000 | 0.000 | 1.000 |

#### Cluster 2

|  | racaCor | faixaEtaria | sexo | ESCOLARID | TIPOCUP | sitAtual | tipoCaso | FORMACLIN1 | classif | descoberta | bac | BACOUTRO | cultEsc | RX | NECROP | hiv | aids | DIABETES | ALCOOLISMO | MENTAL | DROGADICAO | TABAGISMO | motMudEsquema | tipoTrat | idade | HISTOPATOL | Status\_Resistencia |
| --- | --- | --- | --- | --- | --- | --- | --- | --- | --- | --- | --- | --- | --- | --- | --- | --- | --- | --- | --- | --- | --- | --- | --- | --- | --- | --- | --- |
| racaCor | 1.000 | 0.048 | 0.110 | 0.072 | 0.039 | 0.000 | 0.000 | 0.000 | 0.000 | 0.000 | 0.126 | 0.000 | 0.061 | 0.000 | 0.000 | 0.038 | 0.144 | 0.000 | 0.000 | 0.000 | 0.000 | 0.000 | 0.057 | 0.123 | 0.029 | 0.051 | 0.120 |
| faixaEtaria | 0.048 | 1.000 | 0.254 | 0.291 | 0.276 | 0.129 | 0.000 | 0.085 | 0.000 | 0.222 | 0.174 | 0.000 | 0.000 | 0.014 | 0.000 | 0.141 | 0.000 | 0.251 | 0.128 | 0.000 | 0.165 | 0.132 | 0.145 | 0.126 | 0.849 | 0.131 | 0.022 |
| sexo | 0.110 | 0.254 | 1.000 | 0.171 | 0.332 | 0.124 | 0.000 | 0.040 | 0.000 | 0.063 | 0.000 | 0.072 | 0.389 | 0.015 | 0.000 | 0.000 | 0.026 | 0.000 | 0.234 | 0.000 | 0.025 | 0.052 | 0.000 | 0.033 | 0.223 | 0.000 | 0.205 |
| ESCOLARID | 0.072 | 0.291 | 0.171 | 1.000 | 0.156 | 0.048 | 0.000 | 0.140 | 0.092 | 0.077 | 0.166 | 0.072 | 0.039 | 0.035 | 0.000 | 0.000 | 0.000 | 0.000 | 0.120 | 0.143 | 0.070 | 0.000 | 0.039 | 0.079 | 0.178 | 0.022 | 0.090 |
| TIPOCUP | 0.039 | 0.276 | 0.332 | 0.156 | 1.000 | 0.147 | 0.134 | 0.000 | 0.000 | 0.000 | 0.094 | 0.079 | 0.069 | 0.000 | 0.000 | 0.021 | 0.117 | 0.267 | 0.111 | 0.122 | 0.209 | 0.028 | 0.000 | 0.140 | 0.289 | 0.054 | 0.065 |
| sitAtual | 0.000 | 0.129 | 0.124 | 0.048 | 0.147 | 1.000 | 0.092 | 0.000 | 0.091 | 0.145 | 0.144 | 0.000 | 0.037 | 0.000 | 0.000 | 0.158 | 0.000 | 0.000 | 0.000 | 0.000 | 0.076 | 0.000 | 0.000 | 0.000 | 0.111 | 0.006 | 0.000 |
| tipoCaso | 0.000 | 0.000 | 0.000 | 0.000 | 0.134 | 0.092 | 1.000 | 0.000 | 0.000 | 0.000 | 0.000 | 0.034 | 0.000 | 0.106 | 0.277 | 0.000 | 0.077 | 0.000 | 0.192 | 0.118 | 0.162 | 0.000 | 0.000 | 0.000 | 0.064 | 0.000 | 0.000 |
| FORMACLIN1 | 0.000 | 0.085 | 0.040 | 0.140 | 0.000 | 0.000 | 0.000 | 1.000 | 0.696 | 0.000 | 0.414 | 0.292 | 0.000 | 0.329 | 0.000 | 0.000 | 0.000 | 0.093 | 0.000 | 0.000 | 0.000 | 0.000 | 0.000 | 0.119 | 0.041 | 0.361 | 0.119 |
| classif | 0.000 | 0.000 | 0.000 | 0.092 | 0.000 | 0.091 | 0.000 | 0.696 | 1.000 | 0.054 | 0.383 | 0.227 | 0.123 | 0.255 | 0.000 | 0.000 | 0.034 | 0.021 | 0.037 | 0.000 | 0.019 | 0.000 | 0.000 | 0.036 | 0.027 | 0.286 | 0.097 |
| descoberta | 0.000 | 0.222 | 0.063 | 0.077 | 0.000 | 0.145 | 0.000 | 0.000 | 0.054 | 1.000 | 0.080 | 0.000 | 0.112 | 0.075 | 0.000 | 0.000 | 0.000 | 0.000 | 0.024 | 0.024 | 0.000 | 0.000 | 0.000 | 0.030 | 0.098 | 0.047 | 0.134 |
| bac | 0.126 | 0.174 | 0.000 | 0.166 | 0.094 | 0.144 | 0.000 | 0.414 | 0.383 | 0.080 | 1.000 | 0.214 | 0.201 | 0.208 | 0.000 | 0.073 | 0.000 | 0.035 | 0.016 | 0.000 | 0.047 | 0.000 | 0.000 | 0.149 | 0.077 | 0.200 | 0.047 |
| BACOUTRO | 0.000 | 0.000 | 0.072 | 0.072 | 0.079 | 0.000 | 0.034 | 0.292 | 0.227 | 0.000 | 0.214 | 1.000 | 0.124 | 0.095 | 0.187 | 0.000 | 0.000 | 0.000 | 0.000 | 0.000 | 0.000 | 0.044 | 0.000 | 0.103 | 0.000 | 0.163 | 0.057 |
| cultEsc | 0.061 | 0.000 | 0.389 | 0.039 | 0.069 | 0.037 | 0.000 | 0.000 | 0.123 | 0.112 | 0.201 | 0.124 | 1.000 | 0.003 | 0.000 | 0.043 | 0.000 | 0.105 | 0.000 | 0.000 | 0.000 | 0.000 | 0.000 | 0.000 | 0.000 | 0.073 | 0.466 |
| RX | 0.000 | 0.014 | 0.015 | 0.035 | 0.000 | 0.000 | 0.106 | 0.329 | 0.255 | 0.075 | 0.208 | 0.095 | 0.003 | 1.000 | 0.000 | 0.000 | 0.048 | 0.000 | 0.010 | 0.000 | 0.000 | 0.057 | 0.217 | 0.044 | 0.008 | 0.118 | 0.056 |
| NECROP | 0.000 | 0.000 | 0.000 | 0.000 | 0.000 | 0.000 | 0.277 | 0.000 | 0.000 | 0.000 | 0.000 | 0.187 | 0.000 | 0.000 | 1.000 | 0.000 | 0.000 | 0.000 | 0.000 | 0.000 | 0.000 | 0.000 | 0.000 | 0.000 | 0.000 | 0.000 | 0.000 |
| hiv | 0.038 | 0.141 | 0.000 | 0.000 | 0.021 | 0.158 | 0.000 | 0.000 | 0.000 | 0.000 | 0.073 | 0.000 | 0.043 | 0.000 | 0.000 | 1.000 | 0.853 | 0.000 | 0.122 | 0.000 | 0.000 | 0.000 | 0.129 | 0.142 | 0.053 | 0.000 | 0.000 |
| aids | 0.144 | 0.000 | 0.026 | 0.000 | 0.117 | 0.000 | 0.077 | 0.000 | 0.034 | 0.000 | 0.000 | 0.000 | 0.000 | 0.048 | 0.000 | 0.853 | 1.000 | 0.000 | 0.011 | 0.000 | 0.000 | 0.000 | 0.264 | 0.044 | 0.000 | 0.000 | 0.000 |
| DIABETES | 0.000 | 0.251 | 0.000 | 0.000 | 0.267 | 0.000 | 0.000 | 0.093 | 0.021 | 0.000 | 0.035 | 0.000 | 0.105 | 0.000 | 0.000 | 0.000 | 0.000 | 1.000 | 0.000 | 0.053 | 0.000 | 0.000 | 0.093 | 0.117 | 0.257 | 0.000 | 0.000 |
| ALCOOLISMO | 0.000 | 0.128 | 0.234 | 0.120 | 0.111 | 0.000 | 0.192 | 0.000 | 0.037 | 0.024 | 0.016 | 0.000 | 0.000 | 0.010 | 0.000 | 0.122 | 0.011 | 0.000 | 1.000 | 0.000 | 0.264 | 0.110 | 0.000 | 0.000 | 0.181 | 0.000 | 0.000 |
| MENTAL | 0.000 | 0.000 | 0.000 | 0.143 | 0.122 | 0.000 | 0.118 | 0.000 | 0.000 | 0.024 | 0.000 | 0.000 | 0.000 | 0.000 | 0.000 | 0.000 | 0.000 | 0.053 | 0.000 | 1.000 | 0.000 | 0.000 | 0.000 | 0.000 | 0.000 | 0.000 | 0.000 |
| DROGADICAO | 0.000 | 0.165 | 0.025 | 0.070 | 0.209 | 0.076 | 0.162 | 0.000 | 0.019 | 0.000 | 0.047 | 0.000 | 0.000 | 0.000 | 0.000 | 0.000 | 0.000 | 0.000 | 0.264 | 0.000 | 1.000 | 0.110 | 0.000 | 0.000 | 0.203 | 0.000 | 0.000 |
| TABAGISMO | 0.000 | 0.132 | 0.052 | 0.000 | 0.028 | 0.000 | 0.000 | 0.000 | 0.000 | 0.000 | 0.000 | 0.044 | 0.000 | 0.057 | 0.000 | 0.000 | 0.000 | 0.000 | 0.110 | 0.000 | 0.110 | 1.000 | 0.057 | 0.024 | 0.134 | 0.000 | 0.089 |
| motMudEsquema | 0.057 | 0.145 | 0.000 | 0.039 | 0.000 | 0.000 | 0.000 | 0.000 | 0.000 | 0.000 | 0.000 | 0.000 | 0.000 | 0.217 | 0.000 | 0.129 | 0.264 | 0.093 | 0.000 | 0.000 | 0.000 | 0.057 | 1.000 | 0.000 | 0.065 | 0.000 | 0.000 |
| tipoTrat | 0.123 | 0.126 | 0.033 | 0.079 | 0.140 | 0.000 | 0.000 | 0.119 | 0.036 | 0.030 | 0.149 | 0.103 | 0.000 | 0.044 | 0.000 | 0.142 | 0.044 | 0.117 | 0.000 | 0.000 | 0.000 | 0.024 | 0.000 | 1.000 | 0.106 | 0.030 | 0.000 |
| idade | 0.029 | 0.849 | 0.223 | 0.178 | 0.289 | 0.111 | 0.064 | 0.041 | 0.027 | 0.098 | 0.077 | 0.000 | 0.000 | 0.008 | 0.000 | 0.053 | 0.000 | 0.257 | 0.181 | 0.000 | 0.203 | 0.134 | 0.065 | 0.106 | 1.000 | 0.059 | 0.000 |
| HISTOPATOL | 0.051 | 0.131 | 0.000 | 0.022 | 0.054 | 0.006 | 0.000 | 0.361 | 0.286 | 0.047 | 0.200 | 0.163 | 0.073 | 0.118 | 0.000 | 0.000 | 0.000 | 0.000 | 0.000 | 0.000 | 0.000 | 0.000 | 0.000 | 0.030 | 0.059 | 1.000 | 0.000 |
| Status\_Resistencia | 0.120 | 0.022 | 0.205 | 0.090 | 0.065 | 0.000 | 0.000 | 0.119 | 0.097 | 0.134 | 0.047 | 0.057 | 0.466 | 0.056 | 0.000 | 0.000 | 0.000 | 0.000 | 0.000 | 0.000 | 0.000 | 0.089 | 0.000 | 0.000 | 0.000 | 0.000 | 1.000 |

#### Cluster 3

|  | racaCor | faixaEtaria | sexo | ESCOLARID | TIPOCUP | sitAtual | tipoCaso | FORMACLIN1 | classif | descoberta | bac | BACOUTRO | cultEsc | RX | NECROP | hiv | aids | DIABETES | ALCOOLISMO | MENTAL | DROGADICAO | TABAGISMO | motMudEsquema | tipoTrat | idade | HISTOPATOL | Status\_Resistencia |
| --- | --- | --- | --- | --- | --- | --- | --- | --- | --- | --- | --- | --- | --- | --- | --- | --- | --- | --- | --- | --- | --- | --- | --- | --- | --- | --- | --- |
| racaCor | 1.000 | 0.000 | 0.000 | 0.000 | 0.000 | 0.000 | 0.000 | 0.000 | 0.000 | 0.098 | 0.000 | 0.052 | 0.101 | 0.000 | 0.000 | 0.200 | 0.174 | 0.000 | 0.000 | 0.000 | 0.000 | 0.000 | 0.000 | 0.000 | 0.000 | 0.000 | 0.000 |
| faixaEtaria | 0.000 | 1.000 | 0.000 | 0.203 | 0.367 | 0.222 | 0.000 | 0.000 | 0.000 | 0.000 | 0.111 | 0.000 | 0.081 | 0.000 | 0.000 | 0.398 | 0.301 | 0.554 | 0.000 | 0.000 | 0.030 | 0.000 | 0.238 | 0.000 | 0.786 | 0.123 | 0.080 |
| sexo | 0.000 | 0.000 | 1.000 | 0.144 | 0.442 | 0.098 | 0.000 | 0.000 | 0.000 | 0.000 | 0.143 | 0.000 | 0.084 | 0.000 | 0.000 | 0.178 | 0.198 | 0.000 | 0.052 | 0.000 | 0.171 | 0.000 | 0.000 | 0.000 | 0.000 | 0.000 | 0.000 |
| ESCOLARID | 0.000 | 0.203 | 0.144 | 1.000 | 0.083 | 0.000 | 0.188 | 0.000 | 0.000 | 0.000 | 0.087 | 0.000 | 0.000 | 0.074 | 0.000 | 0.098 | 0.088 | 0.000 | 0.142 | 0.269 | 0.203 | 0.160 | 0.000 | 0.000 | 0.191 | 0.000 | 0.000 |
| TIPOCUP | 0.000 | 0.367 | 0.442 | 0.083 | 1.000 | 0.042 | 0.000 | 0.000 | 0.138 | 0.000 | 0.102 | 0.041 | 0.000 | 0.122 | 0.000 | 0.000 | 0.000 | 0.202 | 0.000 | 0.000 | 0.215 | 0.078 | 0.565 | 0.088 | 0.117 | 0.000 | 0.000 |
| sitAtual | 0.000 | 0.222 | 0.098 | 0.000 | 0.042 | 1.000 | 0.132 | 0.127 | 0.046 | 0.129 | 0.033 | 0.097 | 0.164 | 0.265 | 0.000 | 0.159 | 0.140 | 0.000 | 0.000 | 0.000 | 0.314 | 0.000 | 0.000 | 0.074 | 0.203 | 0.102 | 0.022 |
| tipoCaso | 0.000 | 0.000 | 0.000 | 0.188 | 0.000 | 0.132 | 1.000 | 0.000 | 0.153 | 0.000 | 0.000 | 0.021 | 0.021 | 0.000 | 0.000 | 0.000 | 0.000 | 0.000 | 0.230 | 0.214 | 0.305 | 0.000 | 0.000 | 0.000 | 0.105 | 0.000 | 0.000 |
| FORMACLIN1 | 0.000 | 0.000 | 0.000 | 0.000 | 0.000 | 0.127 | 0.000 | 1.000 | 0.798 | 0.000 | 0.244 | 0.292 | 0.286 | 0.276 | 0.000 | 0.319 | 0.357 | 0.000 | 0.000 | 0.000 | 0.000 | 0.000 | 0.000 | 0.000 | 0.000 | 0.066 | 0.207 |
| classif | 0.000 | 0.000 | 0.000 | 0.000 | 0.138 | 0.046 | 0.153 | 0.798 | 1.000 | 0.090 | 0.237 | 0.316 | 0.287 | 0.176 | 0.000 | 0.222 | 0.282 | 0.094 | 0.094 | 0.000 | 0.077 | 0.066 | 0.054 | 0.000 | 0.085 | 0.245 | 0.330 |
| descoberta | 0.098 | 0.000 | 0.000 | 0.000 | 0.000 | 0.129 | 0.000 | 0.000 | 0.090 | 1.000 | 0.000 | 0.088 | 0.114 | 0.127 | 0.000 | 0.000 | 0.000 | 0.000 | 0.000 | 0.000 | 0.000 | 0.223 | 0.000 | 0.148 | 0.015 | 0.000 | 0.150 |
| bac | 0.000 | 0.111 | 0.143 | 0.087 | 0.102 | 0.033 | 0.000 | 0.244 | 0.237 | 0.000 | 1.000 | 0.121 | 0.339 | 0.172 | 0.078 | 0.134 | 0.217 | 0.077 | 0.131 | 0.000 | 0.073 | 0.028 | 0.038 | 0.228 | 0.054 | 0.000 | 0.113 |
| BACOUTRO | 0.052 | 0.000 | 0.000 | 0.000 | 0.041 | 0.097 | 0.021 | 0.292 | 0.316 | 0.088 | 0.121 | 1.000 | 0.148 | 0.057 | 0.000 | 0.083 | 0.078 | 0.026 | 0.000 | 0.000 | 0.000 | 0.146 | 0.094 | 0.000 | 0.000 | 0.166 | 0.000 |
| cultEsc | 0.101 | 0.081 | 0.084 | 0.000 | 0.000 | 0.164 | 0.021 | 0.286 | 0.287 | 0.114 | 0.339 | 0.148 | 1.000 | 0.000 | 0.000 | 0.295 | 0.304 | 0.071 | 0.000 | 0.099 | 0.000 | 0.089 | 0.117 | 0.000 | 0.000 | 0.115 | 0.451 |
| RX | 0.000 | 0.000 | 0.000 | 0.074 | 0.122 | 0.265 | 0.000 | 0.276 | 0.176 | 0.127 | 0.172 | 0.057 | 0.000 | 1.000 | 0.000 | 0.000 | 0.000 | 0.000 | 0.228 | 0.000 | 0.000 | 0.041 | 0.104 | 0.092 | 0.095 | 0.000 | 0.033 |
| NECROP | 0.000 | 0.000 | 0.000 | 0.000 | 0.000 | 0.000 | 0.000 | 0.000 | 0.000 | 0.000 | 0.078 | 0.000 | 0.000 | 0.000 | 1.000 | 0.000 | 0.000 | 0.000 | 0.000 | 0.000 | 0.000 | 0.000 | 0.000 | 0.000 | 0.000 | 0.000 | 0.000 |
| hiv | 0.200 | 0.398 | 0.178 | 0.098 | 0.000 | 0.159 | 0.000 | 0.319 | 0.222 | 0.000 | 0.134 | 0.083 | 0.295 | 0.000 | 0.000 | 1.000 | 0.896 | 0.130 | 0.000 | 0.000 | 0.088 | 0.018 | 0.196 | 0.186 | 0.284 | 0.207 | 0.293 |
| aids | 0.174 | 0.301 | 0.198 | 0.088 | 0.000 | 0.140 | 0.000 | 0.357 | 0.282 | 0.000 | 0.217 | 0.078 | 0.304 | 0.000 | 0.000 | 0.896 | 1.000 | 0.000 | 0.000 | 0.000 | 0.046 | 0.000 | 0.161 | 0.207 | 0.138 | 0.241 | 0.235 |
| DIABETES | 0.000 | 0.554 | 0.000 | 0.000 | 0.202 | 0.000 | 0.000 | 0.000 | 0.094 | 0.000 | 0.077 | 0.026 | 0.071 | 0.000 | 0.000 | 0.130 | 0.000 | 1.000 | 0.000 | 0.000 | 0.000 | 0.000 | 0.000 | 0.000 | 0.013 | 0.000 | 0.094 |
| ALCOOLISMO | 0.000 | 0.000 | 0.052 | 0.142 | 0.000 | 0.000 | 0.230 | 0.000 | 0.094 | 0.000 | 0.131 | 0.000 | 0.000 | 0.228 | 0.000 | 0.000 | 0.000 | 0.000 | 1.000 | 0.000 | 0.380 | 0.202 | 0.000 | 0.050 | 0.000 | 0.063 | 0.000 |
| MENTAL | 0.000 | 0.000 | 0.000 | 0.269 | 0.000 | 0.000 | 0.214 | 0.000 | 0.000 | 0.000 | 0.000 | 0.000 | 0.099 | 0.000 | 0.000 | 0.000 | 0.000 | 0.000 | 0.000 | 1.000 | 0.000 | 0.000 | 0.000 | 0.000 | 0.034 | 0.298 | 0.000 |
| DROGADICAO | 0.000 | 0.030 | 0.171 | 0.203 | 0.215 | 0.314 | 0.305 | 0.000 | 0.077 | 0.000 | 0.073 | 0.000 | 0.000 | 0.000 | 0.000 | 0.088 | 0.046 | 0.000 | 0.380 | 0.000 | 1.000 | 0.196 | 0.000 | 0.000 | 0.133 | 0.000 | 0.000 |
| TABAGISMO | 0.000 | 0.000 | 0.000 | 0.160 | 0.078 | 0.000 | 0.000 | 0.000 | 0.066 | 0.223 | 0.028 | 0.146 | 0.089 | 0.041 | 0.000 | 0.018 | 0.000 | 0.000 | 0.202 | 0.000 | 0.196 | 1.000 | 0.000 | 0.000 | 0.000 | 0.018 | 0.000 |
| motMudEsquema | 0.000 | 0.238 | 0.000 | 0.000 | 0.565 | 0.000 | 0.000 | 0.000 | 0.054 | 0.000 | 0.038 | 0.094 | 0.117 | 0.104 | 0.000 | 0.196 | 0.161 | 0.000 | 0.000 | 0.000 | 0.000 | 0.000 | 1.000 | 0.065 | 0.155 | 0.230 | 0.000 |
| tipoTrat | 0.000 | 0.000 | 0.000 | 0.000 | 0.088 | 0.074 | 0.000 | 0.000 | 0.000 | 0.148 | 0.228 | 0.000 | 0.000 | 0.092 | 0.000 | 0.186 | 0.207 | 0.000 | 0.050 | 0.000 | 0.000 | 0.000 | 0.065 | 1.000 | 0.108 | 0.000 | 0.000 |
| idade | 0.000 | 0.786 | 0.000 | 0.191 | 0.117 | 0.203 | 0.105 | 0.000 | 0.085 | 0.015 | 0.054 | 0.000 | 0.000 | 0.095 | 0.000 | 0.284 | 0.138 | 0.013 | 0.000 | 0.034 | 0.133 | 0.000 | 0.155 | 0.108 | 1.000 | 0.000 | 0.075 |
| HISTOPATOL | 0.000 | 0.123 | 0.000 | 0.000 | 0.000 | 0.102 | 0.000 | 0.066 | 0.245 | 0.000 | 0.000 | 0.166 | 0.115 | 0.000 | 0.000 | 0.207 | 0.241 | 0.000 | 0.063 | 0.298 | 0.000 | 0.018 | 0.230 | 0.000 | 0.000 | 1.000 | 0.106 |
| Status\_Resistencia | 0.000 | 0.080 | 0.000 | 0.000 | 0.000 | 0.022 | 0.000 | 0.207 | 0.330 | 0.150 | 0.113 | 0.000 | 0.451 | 0.033 | 0.000 | 0.293 | 0.235 | 0.094 | 0.000 | 0.000 | 0.000 | 0.000 | 0.000 | 0.000 | 0.075 | 0.106 | 1.000 |

# Missing values

- Count
- Matrix

#### Cluster 1

xml version="1.0" encoding="utf-8" standalone="no"?2023-08-25T13:07:27.990925image/svg+xmlMatplotlib v3.6.0, https://matplotlib.org/ 

A simple visualization of nullity by column.

#### Cluster 2

xml version="1.0" encoding="utf-8" standalone="no"?2023-08-25T13:07:32.893585image/svg+xmlMatplotlib v3.6.0, https://matplotlib.org/ 

A simple visualization of nullity by column.

#### Cluster 3

xml version="1.0" encoding="utf-8" standalone="no"?2023-08-25T13:07:37.326814image/svg+xmlMatplotlib v3.6.0, https://matplotlib.org/ 

A simple visualization of nullity by column.

#### Cluster 1

xml version="1.0" encoding="utf-8" standalone="no"?2023-08-25T13:07:28.501608image/svg+xmlMatplotlib v3.6.0, https://matplotlib.org/ 

Nullity matrix is a data-dense display which lets you quickly visually pick out patterns in data completion.

#### Cluster 2

xml version="1.0" encoding="utf-8" standalone="no"?2023-08-25T13:07:33.456274image/svg+xmlMatplotlib v3.6.0, https://matplotlib.org/ 

Nullity matrix is a data-dense display which lets you quickly visually pick out patterns in data completion.

#### Cluster 3

xml version="1.0" encoding="utf-8" standalone="no"?2023-08-25T13:07:37.837191image/svg+xmlMatplotlib v3.6.0, https://matplotlib.org/ 

Nullity matrix is a data-dense display which lets you quickly visually pick out patterns in data completion.

# Sample

- First rows
- Last rows

#### Cluster 1

|  | racaCor | faixaEtaria | sexo | ESCOLARID | TIPOCUP | sitAtual | tipoCaso | FORMACLIN1 | classif | descoberta | bac | BACOUTRO | cultEsc | RX | NECROP | hiv | aids | DIABETES | ALCOOLISMO | MENTAL | DROGADICAO | TABAGISMO | motMudEsquema | tipoTrat | idade | HISTOPATOL | Status\_Resistencia | Cluster |
| --- | --- | --- | --- | --- | --- | --- | --- | --- | --- | --- | --- | --- | --- | --- | --- | --- | --- | --- | --- | --- | --- | --- | --- | --- | --- | --- | --- | --- |
| 0 | Branco | 20\_29 | M | De 4 a 7 anos | Outra | Cura | Novo | Pul | Pul | Elucidacao Diagn. em Internacao | Pos | N/realiz | Pos | Susp c/cavid | N/realiz | Neg | N | N | N | N | N | N | Nulo | Supervisionado | 23\_39 | N/realiz | 1 | 0 |
| 1 | Pardo | 40\_49 | M | De 4 a 7 anos | Desempregado | Cura | Novo | Pul | P+E | Demanda Ambulatorial | Pos | N/realiz | Pos | Susp TB | N/realiz | Neg | N | N | S | N | N | S | Nulo | Supervisionado | 40\_54 | N/realiz | 1 | 0 |
| 3 | Branco | 50\_59 | M | De 4 a 7 anos | Outra | Cura | Novo | Pul | P+E | Demanda Ambulatorial | Pos | N/realiz | Pos | Susp TB | N/realiz | Pos | S | N | N | N | N | N | Nulo | Supervisionado | Mais de 54 | N/realiz | 1 | 0 |
| 5 | Pardo | 20\_29 | F | De 8 a 11 anos | Desempregado | Cura | Novo | Pul | Pul | Demanda Ambulatorial | Pos | N/realiz | Pos | Susp TB | N/realiz | Pos | N | N | N | N | S | N | Nulo | Supervisionado | 0\_22 | N/realiz | 1 | 0 |
| 6 | Branco | 30\_39 | M | De 8 a 11 anos | Outra | Cura | Novo | Pul | Pul | Demanda Ambulatorial | Neg | N/realiz | Pos | Susp TB | N/realiz | Neg | N | N | N | N | N | S | Nulo | Supervisionado | 23\_39 | N/realiz | 1 | 0 |
| 8 | Pardo | 60\_69 | M | De 8 a 11 anos | Aposentado | Cura | Novo | Pul | Pul | Elucidacao Diagn. em Internacao | Pos | N/realiz | Pos | Susp c/cavid | N/realiz | Neg | N | N | N | N | N | N | Nulo | Supervisionado | Mais de 54 | N/realiz | 1 | 0 |
| 11 | Preto | 20\_29 | M | De 8 a 11 anos | Outra | Cura | Novo | Pul | Pul | Demanda Ambulatorial | N/realiz | N/realiz | Neg | Susp TB | N/realiz | Neg | N | N | N | N | S | S | Nulo | Supervisionado | 0\_22 | N/realiz | 0 | 0 |
| 14 | Preto | 20\_29 | M | De 4 a 7 anos | Desempregado | Abandono | Novo | Pul | Pul | Urgencia / Emergencia | Pos | N/realiz | Pos | Susp c/cavid | N/realiz | Neg | N | N | N | N | N | S | Nulo | Supervisionado | 0\_22 | N/realiz | 1 | 0 |
| 15 | Branco | 20\_29 | F | De 4 a 7 anos | Outra | Cura | Novo | Pul | Pul | Urgencia / Emergencia | Pos | N/realiz | Pos | Susp TB | N/realiz | Neg | N | N | S | N | N | N | Nulo | Supervisionado | 23\_39 | N/realiz | 1 | 0 |
| 17 | Preto | 40\_49 | F | De 12 a 14 anos | Dona de Casa | Cura | Novo | Pul | Pul | Demanda Ambulatorial | Pos | N/realiz | Pos | Susp TB | N/realiz | Neg | N | N | N | N | N | N | Nulo | Supervisionado | 40\_54 | N/realiz | 1 | 0 |

#### Cluster 2

|  | racaCor | faixaEtaria | sexo | ESCOLARID | TIPOCUP | sitAtual | tipoCaso | FORMACLIN1 | classif | descoberta | bac | BACOUTRO | cultEsc | RX | NECROP | hiv | aids | DIABETES | ALCOOLISMO | MENTAL | DROGADICAO | TABAGISMO | motMudEsquema | tipoTrat | idade | HISTOPATOL | Status\_Resistencia | Cluster |
| --- | --- | --- | --- | --- | --- | --- | --- | --- | --- | --- | --- | --- | --- | --- | --- | --- | --- | --- | --- | --- | --- | --- | --- | --- | --- | --- | --- | --- |
| 12 | Branco | 40\_49 | F | 15 anos e mais | Outra | Cura | Novo | Ganglionar Periferica | Ext | Demanda Ambulatorial | Neg | N/realiz | N/realiz | Susp TB | N/realiz | Neg | N | N | N | N | N | N | Nulo | Supervisionado | 40\_54 | N/realiz | 0 | 1 |
| 13 | Branco | 40\_49 | M | De 1 a 3 anos | Outra | Cura | Novo | Pul | Pul | Demanda Ambulatorial | Pos | N/realiz | N/realiz | N/realiz | N/realiz | Neg | N | N | S | N | S | S | Nulo | Supervisionado | 40\_54 | N/realiz | 0 | 1 |
| 16 | Branco | 40\_49 | F | De 8 a 11 anos | Dona de Casa | Cura | Novo | Pleural | Ext | Demanda Ambulatorial | N/realiz | N/realiz | N/realiz | N/realiz | N/realiz | Neg | N | N | N | N | N | N | Nulo | Supervisionado | 40\_54 | BAAR pos | 0 | 1 |
| 23 | Branco | 50\_59 | F | De 4 a 7 anos | Outra | Cura | Novo | Pul | Pul | Demanda Ambulatorial | Pos | N/realiz | Pos | Susp TB | N/realiz | Neg | N | N | N | N | N | N | Nulo | Auto-Administrado | Mais de 54 | N/realiz | 0 | 1 |
| 25 | Pardo | 10\_14 | F | De 4 a 7 anos | Outra | Cura | Novo | Pul | Pul | Demanda Ambulatorial | Neg | N/realiz | Neg | Susp c/cavid | N/realiz | Neg | N | N | N | N | N | N | Nulo | Supervisionado | 0\_22 | N/realiz | 0 | 1 |
| 29 | Pardo | 50\_59 | M | De 1 a 3 anos | Outra | Abandono | Novo | Pul | Pul | Demanda Ambulatorial | Pos | N/realiz | N/realiz | Susp TB | N/realiz | Neg | N | N | N | N | N | N | Nulo | Supervisionado | Mais de 54 | N/realiz | 0 | 1 |
| 33 | Branco | 15\_19 | F | De 4 a 7 anos | Outra | Cura | Novo | Pul | Pul | Urgencia / Emergencia | Pos | N/realiz | Pos | Normal | N/realiz | Neg | N | N | S | N | S | N | Nulo | Supervisionado | 0\_22 | Sugestivo TB | 0 | 1 |
| 36 | Branco | 15\_19 | F | De 8 a 11 anos | Dona de Casa | Cura | Novo | Pul | Pul | Demanda Ambulatorial | Neg | N/realiz | Neg | Susp TB | N/realiz | Neg | N | N | N | N | N | N | Nulo | Supervisionado | 0\_22 | N/realiz | 0 | 1 |
| 41 | Branco | 40\_49 | M | De 4 a 7 anos | Outra | Cura | Novo | Pul | Pul | Demanda Ambulatorial | Pos | N/realiz | N/realiz | Susp TB | N/realiz | Neg | N | N | N | N | N | N | Nulo | Supervisionado | 40\_54 | N/realiz | 0 | 1 |
| 42 | Branco | 40\_49 | F | De 12 a 14 anos | Profissional de Saude | Cura | Recidiva | Pul | Pul | Demanda Ambulatorial | Pos | N/realiz | Pos | N/realiz | N/realiz | Neg | N | N | N | N | N | N | Nulo | Auto-Administrado | 40\_54 | N/realiz | 0 | 1 |

#### Cluster 3

|  | racaCor | faixaEtaria | sexo | ESCOLARID | TIPOCUP | sitAtual | tipoCaso | FORMACLIN1 | classif | descoberta | bac | BACOUTRO | cultEsc | RX | NECROP | hiv | aids | DIABETES | ALCOOLISMO | MENTAL | DROGADICAO | TABAGISMO | motMudEsquema | tipoTrat | idade | HISTOPATOL | Status\_Resistencia | Cluster |
| --- | --- | --- | --- | --- | --- | --- | --- | --- | --- | --- | --- | --- | --- | --- | --- | --- | --- | --- | --- | --- | --- | --- | --- | --- | --- | --- | --- | --- |
| 2 | Pardo | 40\_49 | M | De 8 a 11 anos | Outra | Cura | Novo | Pul | P+E | Elucidacao Diagn. em Internacao | N/realiz | Pos | N/realiz | Susp TB | N/realiz | Pos | S | N | N | N | N | N | Nulo | Auto-Administrado | 40\_54 | BAAR pos | 1 | 2 |
| 4 | Branco | 30\_39 | M | De 4 a 7 anos | Outra | Cura | Novo | Pul | P+E | Urgencia / Emergencia | Pos | N/realiz | N/realiz | Susp TB | N/realiz | Pos | S | N | N | N | S | N | Nulo | Supervisionado | 23\_39 | Sugestivo TB | 0 | 2 |
| 7 | Branco | 20\_29 | M | De 8 a 11 anos | Desempregado | Abandono | Novo | Pul | Pul | Demanda Ambulatorial | Neg | N/realiz | Neg | Susp c/cavid | N/realiz | Pos | S | N | S | N | S | N | Nulo | Supervisionado | 23\_39 | N/realiz | 0 | 2 |
| 9 | Branco | 20\_29 | M | De 1 a 3 anos | Outra | Cura | Novo | Pleural | Ext | Elucidacao Diagn. em Internacao | Neg | N/realiz | Neg | Susp TB | N/realiz | N/realiz | N | N | N | N | N | N | Nulo | Auto-Administrado | 0\_22 | Sugestivo TB | 0 | 2 |
| 10 | Preto | 30\_39 | M | De 8 a 11 anos | Outra | Cura | Novo | Pul | P+E | Elucidacao Diagn. em Internacao | N/realiz | Pos | N/realiz | Susp TB | N/realiz | Pos | S | N | N | N | N | N | Nulo | Auto-Administrado | 23\_39 | N/realiz | 1 | 2 |
| 18 | Branco | 30\_39 | M | 15 anos e mais | Outra | Cura | Novo | Pul | Pul | Demanda Ambulatorial | Neg | N/realiz | Pos | Susp TB | N/realiz | Pos | S | N | N | N | N | N | Nulo | Auto-Administrado | 40\_54 | N/realiz | 0 | 2 |
| 21 | Preto | 30\_39 | M | De 4 a 7 anos | Outra | Abandono | Novo | Pul | P+E | Elucidacao Diagn. em Internacao | Neg | N/realiz | Pos | Susp TB | N/realiz | Pos | S | N | N | N | N | N | Nulo | Auto-Administrado | 23\_39 | N/realiz | 1 | 2 |
| 24 | Pardo | 50\_59 | M | De 8 a 11 anos | Profissional de Saude | Cura | Novo | Pul | P+E | Elucidacao Diagn. em Internacao | Neg | N/realiz | Pos | N/realiz | N/realiz | Pos | S | N | N | N | N | N | Resistencia Medicamentosa | Supervisionado | Mais de 54 | N/realiz | 1 | 2 |
| 26 | Branco | 30\_39 | M | De 4 a 7 anos | Outra | Abandono | Novo | Pul | P+E | Elucidacao Diagn. em Internacao | Pos | Pos | Pos | Susp TB | N/realiz | Pos | S | N | N | N | N | N | Nulo | Auto-Administrado | 23\_39 | N/realiz | 1 | 2 |
| 28 | Branco | 30\_39 | M | De 8 a 11 anos | Outra | Cura | Novo | Pleural | Ext | Elucidacao Diagn. em Internacao | Neg | N/realiz | Neg | Susp TB | N/realiz | Neg | N | N | N | N | N | N | Nulo | Auto-Administrado | 23\_39 | N/realiz | 0 | 2 |

#### Cluster 1

|  | racaCor | faixaEtaria | sexo | ESCOLARID | TIPOCUP | sitAtual | tipoCaso | FORMACLIN1 | classif | descoberta | bac | BACOUTRO | cultEsc | RX | NECROP | hiv | aids | DIABETES | ALCOOLISMO | MENTAL | DROGADICAO | TABAGISMO | motMudEsquema | tipoTrat | idade | HISTOPATOL | Status\_Resistencia | Cluster |
| --- | --- | --- | --- | --- | --- | --- | --- | --- | --- | --- | --- | --- | --- | --- | --- | --- | --- | --- | --- | --- | --- | --- | --- | --- | --- | --- | --- | --- |
| 1512 | Pardo | 30\_39 | M | De 8 a 11 anos | Desempregado | Cura | Novo | Pul | Pul | Demanda Ambulatorial | Neg | N/realiz | Pos | Susp TB | N/realiz | Neg | N | N | S | N | N | N | Nulo | Supervisionado | 23\_39 | N/realiz | 1 | 0 |
| 1516 | Branco | 20\_29 | M | De 8 a 11 anos | Outra | Cura | Recidiva | Pul | Pul | Demanda Ambulatorial | Pos | N/realiz | Pos | Susp c/cavid | N/realiz | Neg | N | N | N | N | N | N | Nulo | Supervisionado | 0\_22 | N/realiz | 0 | 0 |
| 1517 | Preto | 60\_69 | M | De 4 a 7 anos | Aposentado | Cura | Novo | Pul | Pul | Demanda Ambulatorial | Neg | N/realiz | Pos | Susp TB | N/realiz | Neg | N | N | S | N | N | S | Nulo | Supervisionado | Mais de 54 | N/realiz | 1 | 0 |
| 1518 | Preto | 20\_29 | M | De 4 a 7 anos | Outra | Cura | Novo | Pul | Pul | Demanda Ambulatorial | Pos | N/realiz | Pos | Normal | N/realiz | Neg | N | N | N | N | N | N | Nulo | Supervisionado | 0\_22 | N/realiz | 0 | 0 |
| 1521 | Pardo | 30\_39 | M | De 4 a 7 anos | Outra | Cura | Novo | Pul | Pul | Demanda Ambulatorial | Neg | N/realiz | Pos | Susp TB | N/realiz | Neg | N | N | S | N | S | N | Nulo | Supervisionado | 23\_39 | N/realiz | 0 | 0 |
| 1526 | Pardo | 20\_29 | F | De 4 a 7 anos | Dona de Casa | Abandono | Novo | Pul | Pul | Urgencia / Emergencia | Pos | N/realiz | Pos | Susp TB | N/realiz | Neg | N | N | S | N | S | S | Nulo | Supervisionado | 0\_22 | N/realiz | 1 | 0 |
| 1527 | Branco | 50\_59 | M | De 4 a 7 anos | Outra | Abandono | Novo | Pul | Pul | Urgencia / Emergencia | Pos | N/realiz | Pos | Normal | N/realiz | N/realiz | N | S | N | N | N | N | Nulo | Supervisionado | Mais de 54 | N/realiz | 1 | 0 |
| 1531 | Indigena | 50\_59 | M | De 4 a 7 anos | Outra | Cura | Novo | Pul | Pul | Demanda Ambulatorial | Pos | N/realiz | Pos | N/realiz | N/realiz | Neg | N | N | N | N | N | N | Nulo | Supervisionado | Mais de 54 | N/realiz | 1 | 0 |
| 1533 | Pardo | 30\_39 | M | De 8 a 11 anos | Outra | Cura | Recidiva | Pul | Pul | Elucidacao Diagn. em Internacao | Pos | N/realiz | Pos | Susp TB | N/realiz | Neg | N | N | N | N | N | N | Nulo | Supervisionado | 23\_39 | N/realiz | 0 | 0 |
| 1534 | Pardo | 30\_39 | M | De 1 a 3 anos | Desempregado | Cura | Novo | Pul | Pul | Elucidacao Diagn. em Internacao | Pos | N/realiz | Pos | N/realiz | N/realiz | Pos | S | N | S | N | S | N | Nulo | Supervisionado | 23\_39 | N/realiz | 1 | 0 |

#### Cluster 2

|  | racaCor | faixaEtaria | sexo | ESCOLARID | TIPOCUP | sitAtual | tipoCaso | FORMACLIN1 | classif | descoberta | bac | BACOUTRO | cultEsc | RX | NECROP | hiv | aids | DIABETES | ALCOOLISMO | MENTAL | DROGADICAO | TABAGISMO | motMudEsquema | tipoTrat | idade | HISTOPATOL | Status\_Resistencia | Cluster |
| --- | --- | --- | --- | --- | --- | --- | --- | --- | --- | --- | --- | --- | --- | --- | --- | --- | --- | --- | --- | --- | --- | --- | --- | --- | --- | --- | --- | --- |
| 1507 | Branco | 40\_49 | F | 15 anos e mais | Profissional de Saude | Cura | Novo | Pul | Pul | Demanda Ambulatorial | Neg | N/realiz | Pos | Susp c/cavid | N/realiz | Neg | N | N | N | N | N | S | Nulo | Auto-Administrado | 40\_54 | N/realiz | 1 | 1 |
| 1509 | Branco | 20\_29 | M | De 4 a 7 anos | Outra | Cura | Novo | Pul | Pul | Demanda Ambulatorial | Pos | N/realiz | Neg | Susp TB | N/realiz | Neg | N | N | N | N | N | N | Nulo | Supervisionado | 23\_39 | N/realiz | 0 | 1 |
| 1511 | Branco | 50\_59 | F | De 4 a 7 anos | Outra | Cura | Novo | Pul | Pul | Demanda Ambulatorial | Pos | N/realiz | Pos | N/realiz | N/realiz | Neg | N | N | N | N | N | N | Nulo | Supervisionado | Mais de 54 | N/realiz | 1 | 1 |
| 1514 | Branco | 50\_59 | M | De 8 a 11 anos | Outra | Cura | Novo | Ganglionar Periferica | Ext | Demanda Ambulatorial | Neg | N/realiz | N/realiz | Susp TB | N/realiz | Neg | N | N | N | N | N | N | Nulo | Supervisionado | Mais de 54 | N/realiz | 0 | 1 |
| 1520 | Branco | 20\_29 | M | De 1 a 3 anos | Desempregado | Cura | Novo | Pul | Pul | Demanda Ambulatorial | Pos | N/realiz | N/realiz | Susp TB | N/realiz | Neg | N | N | N | N | N | N | Nulo | Supervisionado | 0\_22 | N/realiz | 0 | 1 |
| 1522 | Pardo | 40\_49 | M | De 4 a 7 anos | Outra | Cura | Novo | Pul | Pul | Demanda Ambulatorial | Pos | N/realiz | N/realiz | Susp TB | N/realiz | Neg | N | N | N | N | N | N | Nulo | Supervisionado | 40\_54 | N/realiz | 0 | 1 |
| 1524 | Branco | 50\_59 | M | De 4 a 7 anos | Outra | Cura | Novo | Pul | Pul | Elucidacao Diagn. em Internacao | Pos | N/realiz | N/realiz | Susp TB | N/realiz | Neg | N | N | S | N | N | N | Nulo | Supervisionado | Mais de 54 | N/realiz | 0 | 1 |
| 1529 | Branco | 40\_49 | M | De 1 a 3 anos | Outra | Cura | Novo | Pul | Pul | Demanda Ambulatorial | Neg | N/realiz | Neg | Susp TB | N/realiz | Neg | N | N | S | N | N | N | Nulo | Supervisionado | 40\_54 | N/realiz | 0 | 1 |
| 1530 | Branco | 70\_79 | F | De 4 a 7 anos | Aposentado | Cura | Novo | Pul | Pul | Demanda Ambulatorial | Pos | N/realiz | Pos | Susp TB | N/realiz | Neg | N | N | S | N | N | N | Nulo | Supervisionado | Mais de 54 | N/realiz | 0 | 1 |
| 1535 | Branco | 40\_49 | M | De 4 a 7 anos | Outra | Cura | Novo | Pul | Pul | Urgencia / Emergencia | Pos | N/realiz | N/realiz | Susp c/cavid | N/realiz | Neg | N | S | N | N | N | N | Nulo | Supervisionado | 40\_54 | N/realiz | 0 | 1 |

#### Cluster 3

|  | racaCor | faixaEtaria | sexo | ESCOLARID | TIPOCUP | sitAtual | tipoCaso | FORMACLIN1 | classif | descoberta | bac | BACOUTRO | cultEsc | RX | NECROP | hiv | aids | DIABETES | ALCOOLISMO | MENTAL | DROGADICAO | TABAGISMO | motMudEsquema | tipoTrat | idade | HISTOPATOL | Status\_Resistencia | Cluster |
| --- | --- | --- | --- | --- | --- | --- | --- | --- | --- | --- | --- | --- | --- | --- | --- | --- | --- | --- | --- | --- | --- | --- | --- | --- | --- | --- | --- | --- |
| 1505 | Branco | 30\_39 | M | De 1 a 3 anos | Outra | Cura | Novo | Ganglionar Periferica | Ext | Elucidacao Diagn. em Internacao | N/realiz | N/realiz | N/realiz | Normal | N/realiz | Neg | N | N | N | N | N | N | Nulo | Auto-Administrado | 40\_54 | Sugestivo TB | 0 | 2 |
| 1506 | Branco | 30\_39 | M | De 12 a 14 anos | Outra | Cura | Novo | Ganglionar Periferica | Ext | Demanda Ambulatorial | N/realiz | N/realiz | N/realiz | Normal | N/realiz | Pos | S | N | N | N | N | N | Nulo | Supervisionado | 23\_39 | N/realiz | 0 | 2 |
| 1510 | Branco | 40\_49 | M | De 4 a 7 anos | Outra | Cura | Novo | Pul | P+E | Demanda Ambulatorial | N/realiz | N/realiz | Pos | Susp TB | N/realiz | Pos | S | N | N | N | N | N | Nulo | Auto-Administrado | 40\_54 | N/realiz | 1 | 2 |
| 1513 | Pardo | 20\_29 | M | De 4 a 7 anos | Outra | Cura | Novo | Pul | P+E | Elucidacao Diagn. em Internacao | Neg | N/realiz | Pos | Susp TB | N/realiz | Pos | S | N | N | N | N | N | Nulo | Auto-Administrado | 0\_22 | N/realiz | 1 | 2 |
| 1515 | Branco | 40\_49 | F | De 4 a 7 anos | Outra | Cura | Novo | Pul | P+E | Demanda Ambulatorial | N/realiz | Pos | Pos | Susp TB | N/realiz | Pos | S | N | N | N | N | N | Nulo | Supervisionado | 40\_54 | BAAR pos | 1 | 2 |
| 1519 | Preto | 30\_39 | F | De 12 a 14 anos | Outra | Abandono | Novo | Pul | Pul | Elucidacao Diagn. em Internacao | Pos | N/realiz | Pos | Susp TB | N/realiz | Pos | S | N | S | N | N | N | Nulo | Auto-Administrado | 23\_39 | N/realiz | 1 | 2 |
| 1523 | Preto | 40\_49 | F | De 8 a 11 anos | Outra | Cura | Novo | Pul | Pul | Elucidacao Diagn. em Internacao | Pos | N/realiz | N/realiz | Susp TB | N/realiz | Pos | S | S | N | N | N | N | Nulo | Auto-Administrado | 40\_54 | N/realiz | 0 | 2 |
| 1525 | Pardo | 30\_39 | M | De 12 a 14 anos | Outra | Cura | Recidiva | Pul | P+E | Demanda Ambulatorial | Neg | N/realiz | Pos | Susp TB | N/realiz | Pos | S | N | N | N | N | N | Nulo | Auto-Administrado | 23\_39 | N/realiz | 1 | 2 |
| 1528 | Pardo | 20\_29 | F | De 4 a 7 anos | Outra | Abandono | Novo | Pul | P+E | Elucidacao Diagn. em Internacao | Neg | Neg | Pos | N/realiz | N/realiz | Pos | S | N | N | N | N | N | Nulo | Auto-Administrado | 0\_22 | N/realiz | 1 | 2 |
| 1532 | Preto | 01\_04 | M | Nenhuma | Outra | Cura | Novo | Meningea | Ext | Urgencia / Emergencia | Neg | Neg | Neg | Normal | N/realiz | N/realiz | N | N | N | N | N | N | Nulo | Auto-Administrado | 0\_22 | Sugestivo TB | 0 | 2 |

# Duplicate rows

#### Cluster 1

|  | racaCor | faixaEtaria | sexo | ESCOLARID | TIPOCUP | sitAtual | tipoCaso | FORMACLIN1 | classif | descoberta | bac | BACOUTRO | cultEsc | RX | NECROP | hiv | aids | DIABETES | ALCOOLISMO | MENTAL | DROGADICAO | TABAGISMO | motMudEsquema | tipoTrat | idade | HISTOPATOL | Status\_Resistencia | Cluster | # duplicates |
| --- | --- | --- | --- | --- | --- | --- | --- | --- | --- | --- | --- | --- | --- | --- | --- | --- | --- | --- | --- | --- | --- | --- | --- | --- | --- | --- | --- | --- | --- |
| 112 | Branco | 20\_29 | M | De 8 a 11 anos | Outra | Cura | Novo | Pul | Pul | Demanda Ambulatorial | Pos | N/realiz | Pos | Susp TB | N/realiz | Neg | N | N | N | N | N | N | Nulo | Supervisionado | 0\_22 | N/realiz | 1 | 0 | 2 |
| 207 | Branco | 30\_39 | M | De 4 a 7 anos | Desempregado | Cura | Novo | Pul | Pul | Elucidacao Diagn. em Internacao | Pos | N/realiz | Pos | Susp TB | N/realiz | Neg | N | N | S | N | S | N | Nulo | Supervisionado | 23\_39 | N/realiz | 1 | 0 | 2 |
| 0 | Branco | 20\_29 | F | De 4 a 7 anos | Desempregado | Cura | Novo | Pul | Pul | Demanda Ambulatorial | Pos | N/realiz | Pos | Susp TB | N/realiz | Neg | N | N | N | N | N | N | Nulo | Supervisionado | 0\_22 | N/realiz | 1 | 0 | 0 |
| 1 | Branco | 20\_29 | F | De 4 a 7 anos | Desempregado | Cura | Novo | Pul | Pul | Demanda Ambulatorial | Pos | N/realiz | Pos | Susp TB | N/realiz | Neg | N | N | N | N | N | N | Nulo | Supervisionado | 23\_39 | N/realiz | 1 | 0 | 0 |
| 2 | Branco | 20\_29 | F | De 4 a 7 anos | Desempregado | Cura | Novo | Pul | Pul | Demanda Ambulatorial | Pos | N/realiz | Pos | Susp TB | N/realiz | Neg | N | N | N | N | S | N | Nulo | Supervisionado | 0\_22 | N/realiz | 1 | 0 | 0 |
| 3 | Branco | 20\_29 | F | De 4 a 7 anos | Desempregado | Cura | Novo | Pul | Pul | Demanda Ambulatorial | Pos | N/realiz | Pos | Susp TB | N/realiz | Neg | N | N | N | N | S | N | Nulo | Supervisionado | 23\_39 | N/realiz | 1 | 0 | 0 |
| 4 | Branco | 20\_29 | F | De 4 a 7 anos | Desempregado | Cura | Novo | Pul | Pul | Demanda Ambulatorial | Pos | N/realiz | Pos | Susp TB | N/realiz | Neg | N | N | S | N | N | N | Nulo | Supervisionado | 0\_22 | N/realiz | 1 | 0 | 0 |
| 5 | Branco | 20\_29 | F | De 4 a 7 anos | Desempregado | Cura | Novo | Pul | Pul | Demanda Ambulatorial | Pos | N/realiz | Pos | Susp TB | N/realiz | Neg | N | N | S | N | N | N | Nulo | Supervisionado | 23\_39 | N/realiz | 1 | 0 | 0 |
| 6 | Branco | 20\_29 | F | De 4 a 7 anos | Desempregado | Cura | Novo | Pul | Pul | Demanda Ambulatorial | Pos | N/realiz | Pos | Susp TB | N/realiz | Neg | N | N | S | N | S | N | Nulo | Supervisionado | 0\_22 | N/realiz | 1 | 0 | 0 |
| 7 | Branco | 20\_29 | F | De 4 a 7 anos | Desempregado | Cura | Novo | Pul | Pul | Demanda Ambulatorial | Pos | N/realiz | Pos | Susp TB | N/realiz | Neg | N | N | S | N | S | N | Nulo | Supervisionado | 23\_39 | N/realiz | 1 | 0 | 0 |

#### Cluster 2

|  | racaCor | faixaEtaria | sexo | ESCOLARID | TIPOCUP | sitAtual | tipoCaso | FORMACLIN1 | classif | descoberta | bac | BACOUTRO | cultEsc | RX | NECROP | hiv | aids | DIABETES | ALCOOLISMO | MENTAL | DROGADICAO | TABAGISMO | motMudEsquema | tipoTrat | idade | HISTOPATOL | Status\_Resistencia | Cluster | # duplicates |
| --- | --- | --- | --- | --- | --- | --- | --- | --- | --- | --- | --- | --- | --- | --- | --- | --- | --- | --- | --- | --- | --- | --- | --- | --- | --- | --- | --- | --- | --- |
| 1272 | Branco | 20\_29 | F | De 8 a 11 anos | Outra | Cura | Novo | Pul | Pul | Urgencia / Emergencia | Pos | N/realiz | N/realiz | Susp TB | N/realiz | Neg | N | N | N | N | N | N | Nulo | Auto-Administrado | 0\_22 | N/realiz | 0 | 1 | 2 |
| 1623 | Branco | 20\_29 | M | De 8 a 11 anos | Outra | Cura | Novo | Pul | Pul | Demanda Ambulatorial | Pos | N/realiz | N/realiz | N/realiz | N/realiz | Neg | N | N | N | N | N | N | Nulo | Supervisionado | 0\_22 | N/realiz | 0 | 1 | 2 |
| 1671 | Branco | 20\_29 | M | De 8 a 11 anos | Outra | Cura | Novo | Pul | Pul | Elucidacao Diagn. em Internacao | Pos | N/realiz | N/realiz | Susp TB | N/realiz | Neg | N | N | N | N | N | N | Nulo | Supervisionado | 0\_22 | N/realiz | 0 | 1 | 2 |
| 2068 | Branco | 30\_39 | F | De 8 a 11 anos | Outra | Cura | Novo | Pul | Pul | Demanda Ambulatorial | Pos | N/realiz | N/realiz | Susp TB | N/realiz | Neg | N | N | N | N | N | N | Nulo | Supervisionado | 23\_39 | N/realiz | 0 | 1 | 2 |
| 4227 | Pardo | 15\_19 | M | De 8 a 11 anos | Outra | Cura | Novo | Pul | Pul | Demanda Ambulatorial | Pos | N/realiz | N/realiz | Susp TB | N/realiz | Neg | N | N | N | N | N | N | Nulo | Supervisionado | 0\_22 | N/realiz | 0 | 1 | 2 |
| 5404 | Pardo | 30\_39 | F | De 8 a 11 anos | Dona de Casa | Cura | Novo | Pul | Pul | Demanda Ambulatorial | Pos | N/realiz | N/realiz | N/realiz | N/realiz | Neg | N | N | N | N | N | N | Nulo | Supervisionado | 23\_39 | N/realiz | 0 | 1 | 2 |
| 5596 | Pardo | 30\_39 | F | De 8 a 11 anos | Outra | Cura | Novo | Pul | Pul | Urgencia / Emergencia | Pos | N/realiz | N/realiz | Susp TB | N/realiz | Neg | N | N | N | N | N | N | Nulo | Supervisionado | 23\_39 | N/realiz | 0 | 1 | 2 |
| 6623 | Pardo | 40\_49 | M | De 4 a 7 anos | Outra | Cura | Novo | Pul | Pul | Demanda Ambulatorial | Pos | N/realiz | N/realiz | Susp c/cavid | N/realiz | Neg | N | N | S | N | N | N | Nulo | Supervisionado | 40\_54 | N/realiz | 0 | 1 | 2 |
| 0 | Branco | 15\_19 | F | De 4 a 7 anos | Dona de Casa | Cura | Novo | Pul | Pul | Demanda Ambulatorial | Pos | N/realiz | N/realiz | N/realiz | N/realiz | Neg | N | N | N | N | N | N | Nulo | Auto-Administrado | 0\_22 | N/realiz | 0 | 1 | 0 |
| 1 | Branco | 15\_19 | F | De 4 a 7 anos | Dona de Casa | Cura | Novo | Pul | Pul | Demanda Ambulatorial | Pos | N/realiz | N/realiz | N/realiz | N/realiz | Neg | N | N | N | N | N | N | Nulo | Auto-Administrado | 23\_39 | N/realiz | 0 | 1 | 0 |

#### Cluster 3

|  | racaCor | faixaEtaria | sexo | ESCOLARID | TIPOCUP | sitAtual | tipoCaso | FORMACLIN1 | classif | descoberta | bac | BACOUTRO | cultEsc | RX | NECROP | hiv | aids | DIABETES | ALCOOLISMO | MENTAL | DROGADICAO | TABAGISMO | motMudEsquema | tipoTrat | idade | HISTOPATOL | Status\_Resistencia | Cluster | # duplicates |
| --- | --- | --- | --- | --- | --- | --- | --- | --- | --- | --- | --- | --- | --- | --- | --- | --- | --- | --- | --- | --- | --- | --- | --- | --- | --- | --- | --- | --- | --- |
| Dataset does not contain duplicate rows. | | | | | | | | | | | | | | | | | | | | | | | | | | | | | |

Report generated by YData.

 
